# Supplementary material for: A general strategy for diversifying complex natural products to polycyclic scaffolds with medium-sized rings
Source: Nat Commun. 2019 Sep 5;10:4015. doi: 10.1038/s41467-019-11976-2 (PMC6728360; doi:10.1038/s41467-019-11976-2)
Supplement: Supplementary file 1 — Supplementary Information [file 41467_2019_11976_MOESM1_ESM.pdf]

Supplementary Information

A general strategy for diversifying complex natural products to polycyclic scaffolds with medium-sized rings

Zhao et al

Table of Contents

I. Supplementary Discussion

Supplementary Figures 1-4 3

II. Supplementary Methods: Experimental procedures and characterization data

General remarks 8

General procedure 9

A-ring expansion of dehydroepiandrosterone (DHEA) and cholesterol 11

Supplementary Figure 5. Synthesis of **3a**, **3b**, **4a**, **5a-c**, **6a** and **6b** 11

D-ring expansion of isosteviol 16

Supplementary Figure 6. Syntheses of **8-10** 17

D-ring expansion of dehydroepiandrosterone (DHEA) and estrone 19

Supplementary Figure 7. Syntheses of medium ring scaffold **14-18** 19

C-H oxidation/ring expansion of dehydroepiandrosterone, cholesterol, isosteviol, estrone and diosgenin 24

Supplementary Figure 8. Electrochemical allylic oxidation/ring expansion of natural products ... 25

Supplementary Figure 9. Syntheses of **24** 29

Supplementary Figure 10. Copper-mediated C-H oxidation/C-ring expansion of DHEA and estrone 31

Supplementary Figure 11. Benzylic C-H oxidation 34

C-H oxidation/ring expansion of picfeltarraegenin and kirenol 35

Supplementary Figure 12. C-H oxidation/ring expansion of picfeltarraegenin and kirenol 35

Derivatization of medium ring scaffolds 40

Supplementary Figure 13. Formation of carbamates 40

Supplementary Figure 14. Synthesis of azide 66

Supplementary Figure 15. Synthesis of imides 68

Supplementary Figure 16. Ring-cleavage of **5b** and **5c** 84

Cheminformatic analysis of medium ring library 93

Supplementary Figure 17-20 94-96

X-Ray reports 100

Supplementary Figure 26-236 (Copies of NMR spectra) 119

Supplementary Figure 237-318 (Copies of LC-MS spectra) 324

III. Supplementary References 362

I. Supplementary Discussion

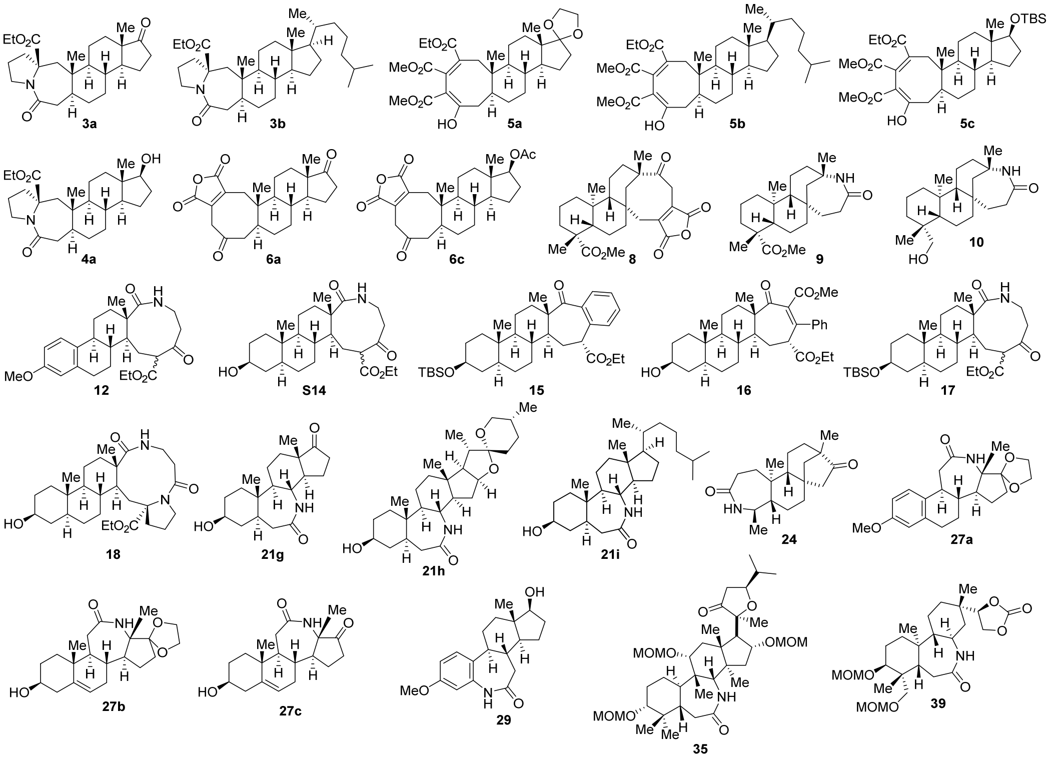

Supplementary Figure 1. Medium-sized ring scaffolds.

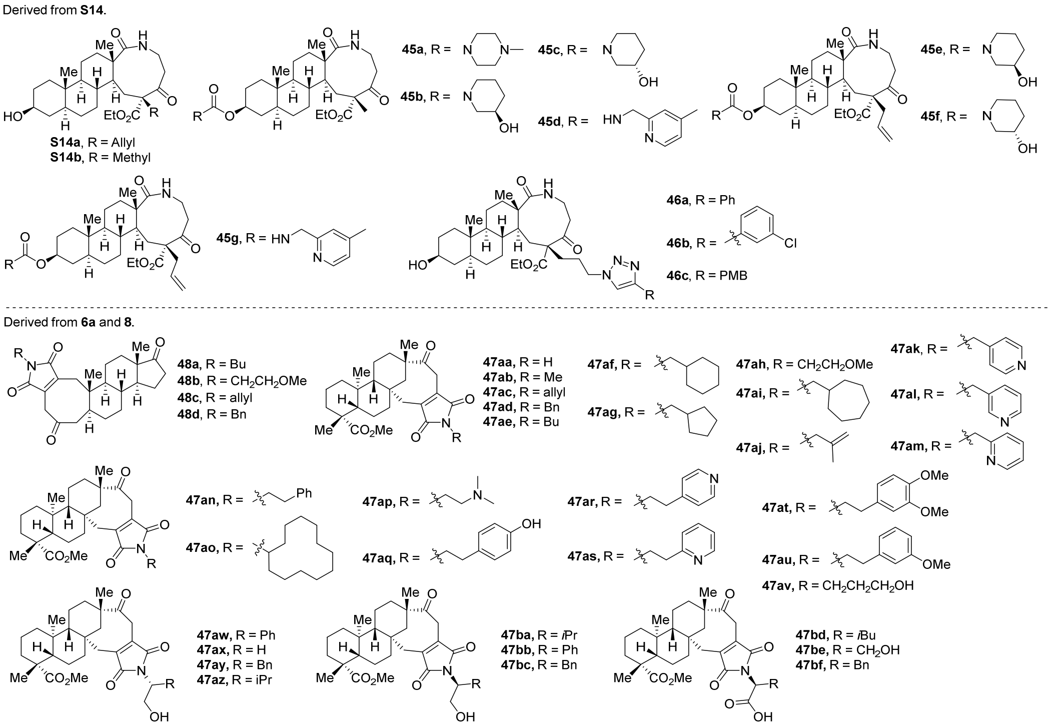

Supplementary Figure 2. Full compound set (continued on next page).

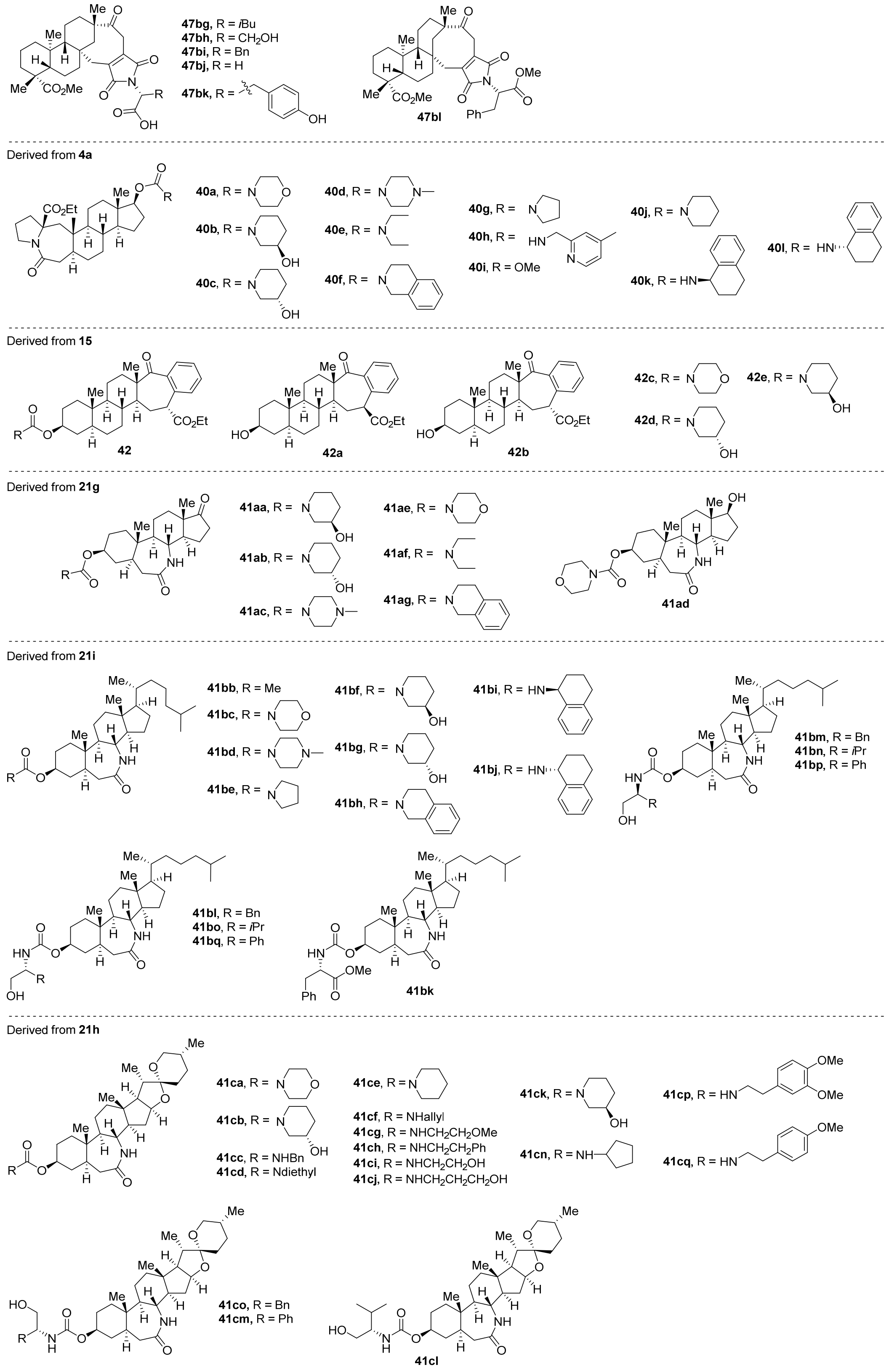

Supplementary Figure 2 (continued). Full compound set (continued on next page).

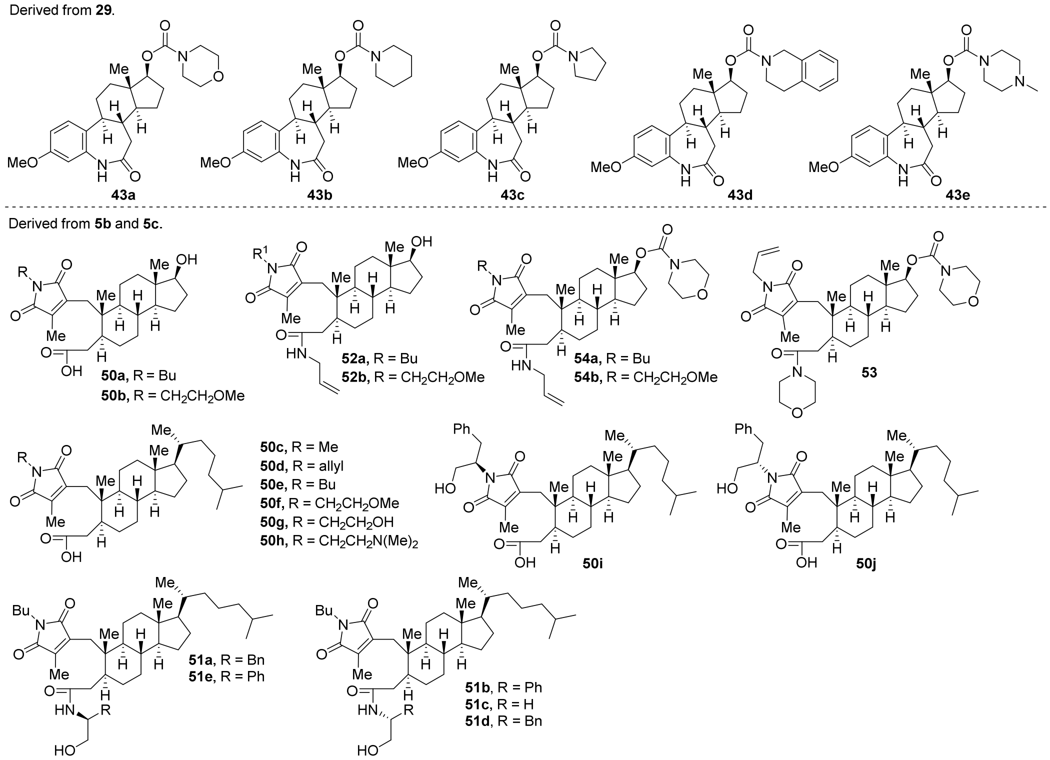

Supplementary Figure 2 (continued). Full compound set.

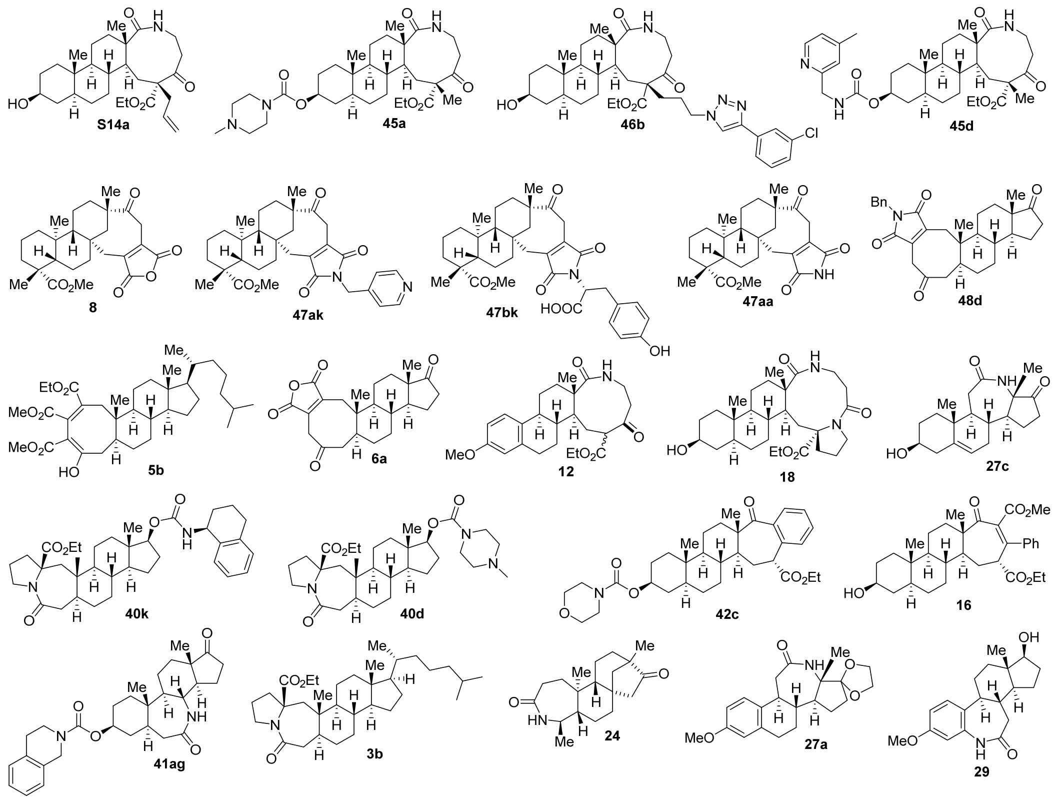

Supplementary Figure 3. Medium sized ring library used in PCA and PMI analyses (continued on next page) (30 structures).

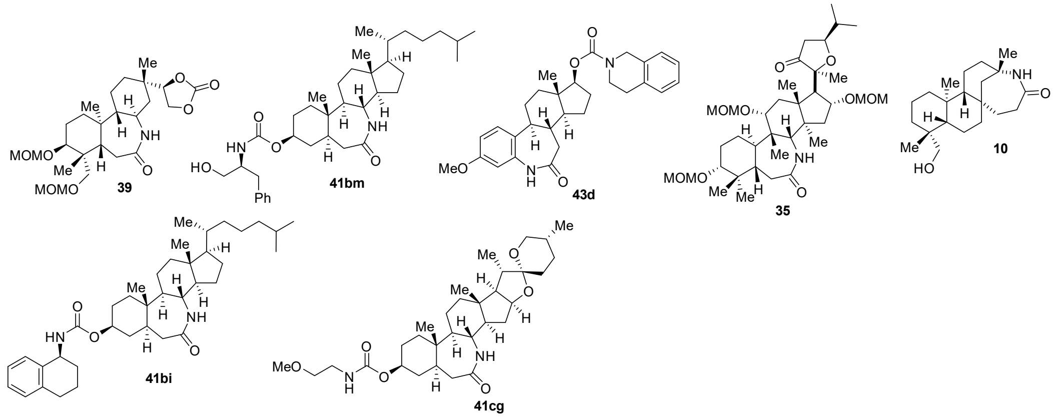

Supplementary Figure 3 (continued). Medium sized ring library used in PCA and PMI analyses (30 structures).

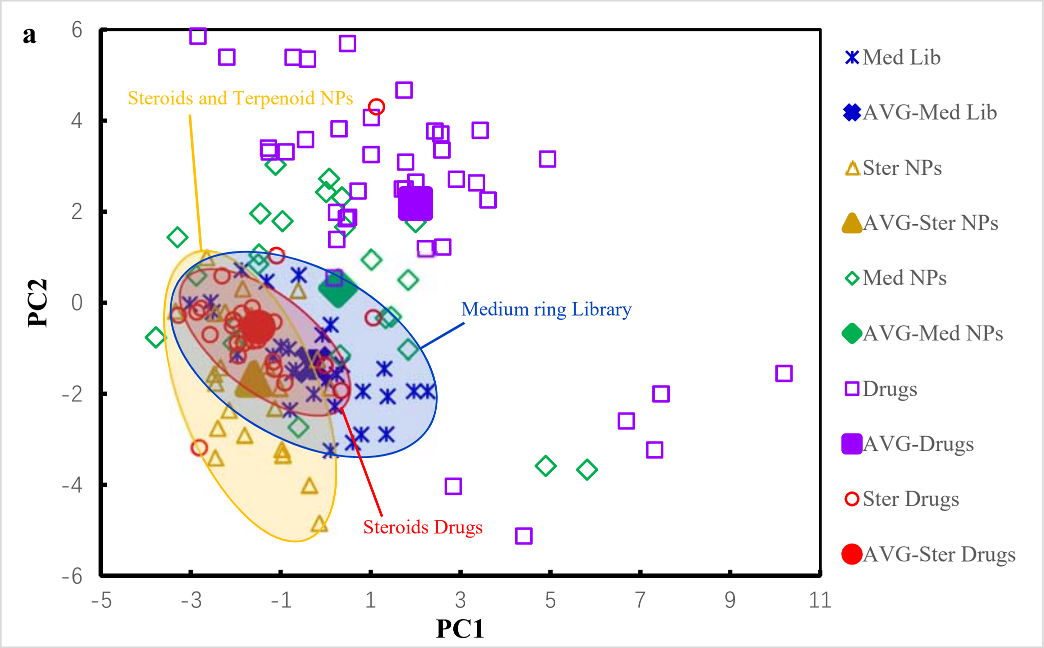

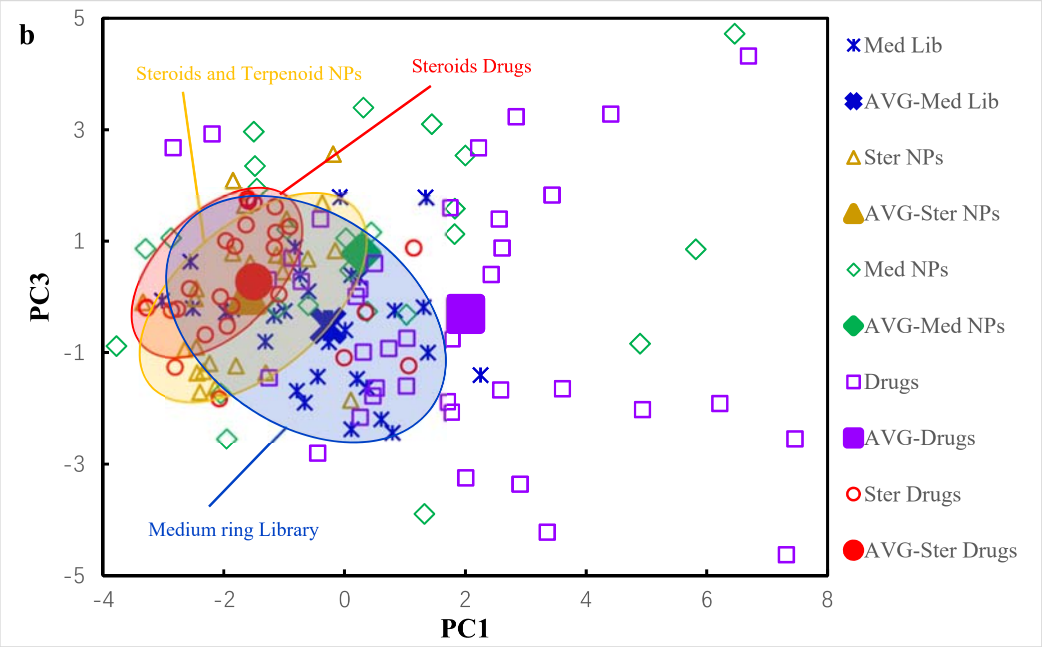
b
Steroids and Terpenoid NPs
Steroids Drugs
Medium ring Library
PC3
PC1
5
3
1
-1
-3
-5
-4
-2
0
2
4
6
8
Med Lib
AVG-Med Lib
Ster NPs
AVG-Ster NPs
Med NPs
AVG-Med NPs
Drugs
AVG-Drugs
Ster Drugs
AVG-Ster Drugs

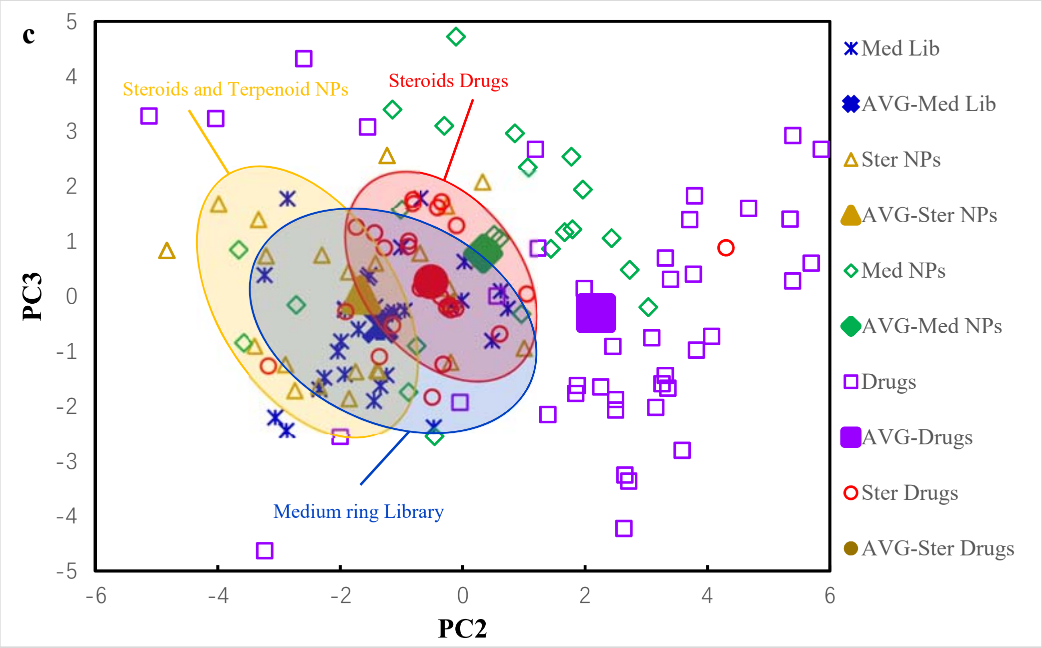
c
Steroids and Terpenoid NPs
Steroids Drugs
Medium ring Library
PC3
PC2
5
4
3
2
1
0
-1
-2
-3
-4
-5
-6
-4
-2
0
2
4
6
Med Lib
AVG-Med Lib
Ster NPs
AVG-Ster NPs
Med NPs
AVG-Med NPs
Drugs
AVG-Drugs
Ster Drugs
AVG-Ster Drugs

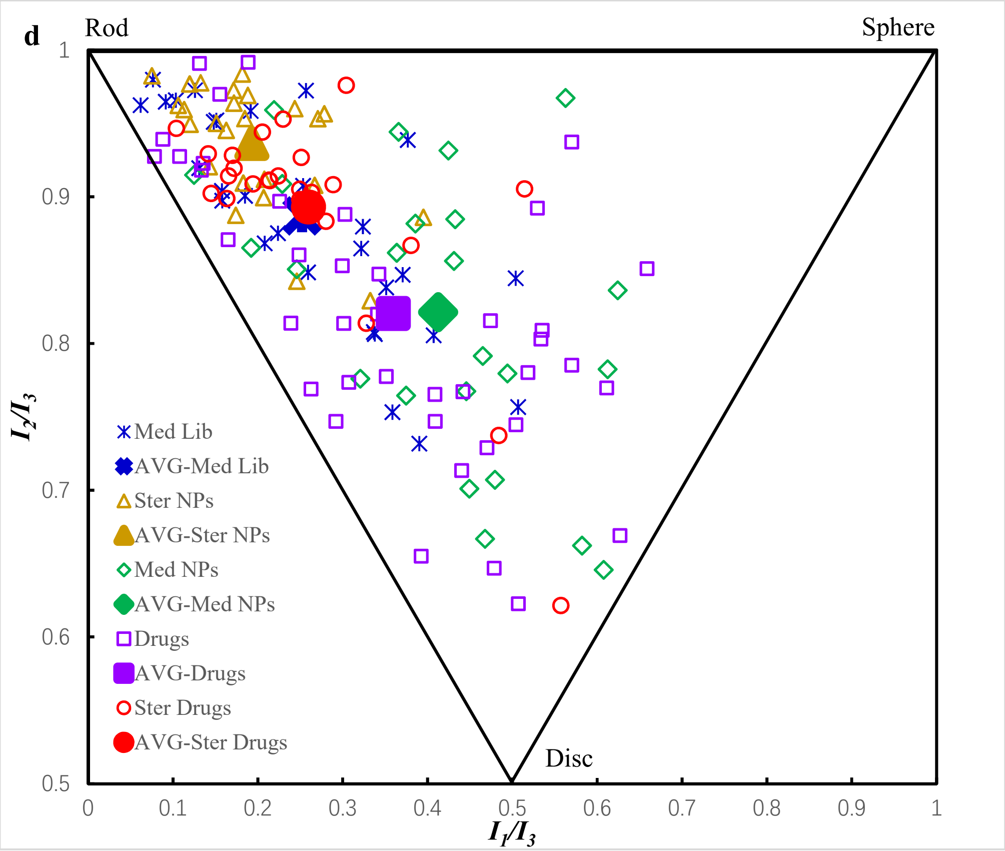

Supplementary Figure 4. Cheminformatic analyses of medium ring library. Expanded version of PCA and PMI plots of 30 medium ring library (Med Lib) members, established reference sets of 40 top selling drugs in 2016 (Drugs), 25 top steroid drugs by prescriptions and retail sales in 2016 (Ster Drugs), 25 diverse steroids and terpenoid natural products (NPs) and 25 diverse medium ring natural products (Med NPs). The hypothetical average structure for each series (-AVG) is also shown. **a**. PCA plot of PC1 versus PC2. **b.** PCA plot of PC1 versus PC3. **c.** PCA plot of PC2 versus PC3. **d.** PMI plot showing the three-dimensional shape of the lowest energy conformations of each compound. 72% of the total variation is represented in the first three principal components. There are significant overlaps between the synthesized medium ring scaffolds and steroid drugs and steroids and terpenoid natural products. See **Supplementary Data Set** for complete data and **Supplementary Methods** for details on parameter and compound selection.

II. Supplementary Methods: Experimental procedures and characterization data.

General remarks.

All reactions in non-aqueous media were conducted under a positive pressure of dry argon in glassware that had been dried in oven prior to use unless noted otherwise. Anhydrous solutions of reaction mixtures were transferred via an oven dried syringe or cannula. All solvents were dried prior to use unless noted otherwise. Thin layer chromatography was performed using precoated silica gel plates (EMD Chemical Inc. 60, F254). Flash column chromatography was performed with silica gel (Silicycle, 40-63 μm). Infrared spectra (IR) were obtained on a Bruker Equinox 55 Spectrophotometer. ^{1}H and ^{13}C nuclear magnetic resonance spectra (NMR) were obtained on a

Bruker 400 MHz or Varian Unity-Inova 500 MHz recorded in ppm (δ) downfield of TMS (δ = 0) in $CDCl_3$, DMSO-d_6, unless noted otherwise. Signal splitting patterns were described as singlet (s), doublet (d), triplet (t), quartet (q), quintet (quint), or multiplet (m), with coupling constants (*J*) in hertz. High resolution mass spectra (HRMS) were performed by Analytical Instrument Center at the School of Pharmacy. The liquid chromatography mass spectrometry LC-MS analysis of final products was processed on Agilent 1290 Infinity II LC system using Poroshell 120 EC-C18 column (5 cm × 2.1 mm, 1.9 μm) for chromatographic separation. Agilent 6120 Quadrupole LC/MS with multimode Electrospray Ionization plus atmospheric pressure chemical ionization (MM-ES+APCI) was used for detection. The mobile phases were 5.0% methanol and 0.1% formic acid in purified water (A) and 0.1% formic acid in methanol (B). The gradient was held at 5% (0-0.2 min), increased to 100% at 2.5 min, then held at isocratic 100% B for 0.4 min and then immediately stepped back down to 5% for 0.1 min re-equilibration. The flow rate was set at 0.8 mL/min. Column temperature was set at 40 °C. The purities of all the final compounds were determined to be over 95% by LC-MS.

General procedure.

Procedure A: General procedure for formation of β-keto ester

A solution of *N*-diisopropylamine (1.3 equiv) in tetrahydrofuan (THF) was cooled to −78 °C. To the above solution was added *n*-BuLi (1.25 equiv) dropwise. The resulting solution was stirred for 45 min under argon. To the above reaction mixture was added a solution of ketone (1.0 equiv) in THF at −78 °C. The reaction mixture was kept for 2 h at this temperature. Ethyl cyanoformate (1.3 eq) was added to the above solution. The resulting mixture was stirred at −78 °C for 1 h. The reaction was allowed to warm to room temperature. After quenching with NH_4Cl solution, the mixture was diluted with ethyl acetate and washed with brine (50 mL × 2). The combined organic phase was dried over sodium sulfate and concentrated under vacuum. Flash column chromatography over silica gel afforded the β-keto ester.

Procedure B: General procedure for alkylation of β-keto ester

To a solution of β-keto ester (1.0 equiv) and hexamethylphosphoramide (HMPA) (2.0 equiv) in THF (10 mL/mmol) was added NaH (1.3 equiv) under argon. The reaction mixture was stirred for 1 h. Then alkyl iodide (5.0 equiv) was added. The mixture was stirred for 24 h. After quenching with NH_4Cl solution, the mixture was diluted with ethyl acetate and washed with NaS_2O_3 and brine. The organic phase was dried over sodium sulfate and concentrated under vacuum to afford the crude product. Flash column chromatography over silica gel afforded the product.

Procedure C: General procedure of Backman rearrangement

To a solution of ketone (1.0 equiv) in EtOH (10 mL/mmol) was added hydroxylamine hydrochloride (10.0 equiv) and KOAc (10.0 equiv). The reaction mixture was heated at reflux for 3 h. The mixture was cooled to room temperature. The volatile EtOH was removed under vacuum and the resulting residue was diluted with ethyl acetate and washed with brine (20 mL × 2). The organic phase was dried over sodium sulfate and concentrated under vacuum to afford crude product which was directly used in the next step without further purification.

To a solution of the above crude product in pyridine (5.0 mL/mmol) was added *p*-

toluenesulfonyl chloride (2.0 equiv) and dimethylaminopyridine (0.1 equiv). The resulting reaction mixture was heated at 60 °C overnight, cooled to room temperature, diluted with ethyl acetate (100 mL), and washed with 2 N HCl, $NaHCO_3$ and brine sequentially. The organic phase was dried over sodium sulfate and concentrated under vacuum. Flash column chromatography over silica gel afforded the lactam product.

Procedure D: General procedure of removing TBS group

To a solution of the TBS ether in THF/H_2O (5:1) was added TsOH (0.1 equiv). The reaction mixture was stirred overnight. The mixture was diluted with EtOAc, and washed with brine (10 mL × 2). The organic phase was dried over sodium sulfate and concentrated under vacuum. Flash column chromatography over silica column afforded the alcohol.

Procedure E: General procedure of removing TBS group

A mixture of TBS ether and Pd/C (10%) in MeOH was hydrogenated at room temperature for 96 h under hydrogen atmosphere. The suspension was filtered through a pad of celite and the pad was washed with CH_2Cl_2. The combined filtrates were concentrated to dryness. Flash column chromatography over silica column afford of the alcohol.

Procedure F: General procedure for formation of carbamates

To a solution of alcohol (0.10 mol) in THF (2 mL) was added carbonyldiimidazole (32.4 mg, 0.20 mmol) and Et_3N (20.2 mg, 0.20 mmol). The reaction mixture was stirred at room temperature overnight. The volatile dichloromethane was removed under vacuum and the residue was dissolved in toluene (4 mL), amine (0.50 mmol), Et_3N (40.4 mg, 0.40 mmol) and dimethylaminopyridine (5.0 mg) were added to the reaction mixture. The reaction mixture was heated at 90 °C for 4 h. The mixture was cooled to room temperature then diluted with ethyl acetate (50 mL), and washed with brine (10 mL × 2). The organic phase was dried over sodium sulfate and concentrated under vacuum. Flash column chromatography over silica gel afforded the carbamates.

Procedure G: General procedure for formation of carbamates

To a solution of alcohol (0.10 mol) in dichloromethane (2 mL) was added *N*, *N*-dialkyl-1*H*-imidazole-1-carboxamide (0.20 mmol) and *t*-BuOK (22.4 mg, 0.20 mmol). The reaction mixture was stirred at room temperature overnight. The mixture was diluted with ethyl acetate (30 mL) and washed with brine (10 mL × 2). The organic phase was dried over sodium sulfate and concentrated under vacuum. Flash column chromatography over silica gel afforded the carbamates.

Procedure H: General procedure for formation of imides

To a solution of **8** (44.2 mg, 0.1 mol) in dichloromethane (0.5 mL) and toluene (3.0 mL) was added amine (0.5 mmol). The reaction mixture was stirred for 30 min and then heated at reflux for 2 h. The mixture was cooled to room temperature, and concentrated under vacuum. Flash column chromatography over silica gel afforded the imide. (Note, DMF was used as solvent for amino acids).

Procedure I: General procedure for formation of imides

A solution of **6a** (0.50 mmol) in aqueous NaOH (30%, 5 mL) and THF (5 mL) was stirred for 2 h at 50 °C. The reaction mixture was cooled to 0 °C and then $NaBH_4$ (76 mg, 2.00 mmol) was added

in one portion. The reaction mixture was allowed to warm to room temperature and stirred for 3 h. The reaction mixture was concentrated under vacuum, acidified by the slow addition of 1N HCl, extracted with EtOAc (3 × 30 mL). The combined organic extracts were washed with H_2O (30 mL), brine (30 mL), and dried over Na_2SO_4. The organic phase was concentrated under vacuum and used for the next step.

To a solution of the above crude product in dichloromethane (0.5 mL) and toluene (3.0 ml) was added amine (0.5 mmol). The reaction mixture was stirred for 30 min then heated at reflux for 2 h. The mixture was cooled to room temperature, and concentrated under vacuum. Flash column chromatography over silica gel afforded the imide.

To a solution of the above imide in dichloromethane (5 mL) was added Dess-Martin periodinane (848 mg, 2.0 mmol). The reaction mixture was stirred at room temperature for 3 h. The mixture was diluted with ethyl acetate (30 mL) and washed with NaS_2O_3 (10 mL) and brine (10 mL × 2). The organic phase was dried over sodium sulfate and concentrated under vacuum. Flash column chromatography over silica gel afforded the final imide product.

A-ring expansion of dehydroepiandrosterone (DHEA) and cholesterol.

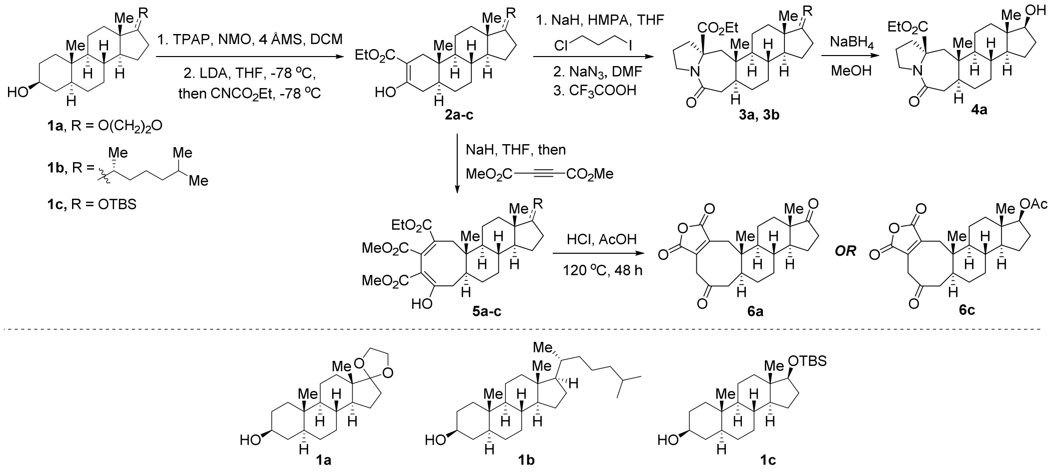

Supplementary Figure 5. Synthesis of 3a, 3b, 4a, 5a-c, 6a and 6b. The TBS ether **1c** was synthesized as reported[1]. **2a-c** were prepared following general procedure A. Alkylation of **2a** and **2b** was performed following general procedure B.

Syntheses of 2a-c.

Tetrapropylammonium perruthenate (526 mg, 1.5 mmol) was added to the solution of **1** (30.0 mmol), 4-methylmorpholine N-oxide (7.02 g, 30.0 mmol) and molecular sieves (1.5 g) in CH_2Cl_2 (150 mL). After stirring at rt for 1 h, the suspension was filtered through a pad of celite and the pad was washed with CH_2Cl_2, concentrated under reduced pressure. Flash column chromatography over short silica column afford off white solid of **S1a-c**.

S1a, yield: 95%. Its spectral data obtained were identical to those reported in literature.[2]
S1b, yield: 90%. Its spectral data obtained were identical to those reported in literature.[3]

S1c, yield: 92%. Its spectral data obtained were identical with those reported in literature.[1]

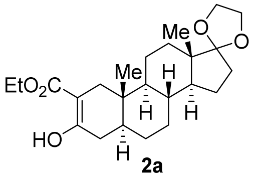

2a

2a, yield: (74%). **^{1}H NMR** (500 MHz, $CDCl_3$) δ 12.17 (s, 1H), 4.28 – 4.06 (m, 2H), 3.98 – 3.79 (m, 4H), 2.30 (d, *J* = 15.7 Hz, 1H), 2.11 (ddd, *J* = 18.6, 5.5, 1.4 Hz, 1H), 2.05 – 1.91 (m, 2H), 1.87 – 1.50 (m, 5H), 1.52 – 1.13 (m, 12H), 0.96 – 0.67 (m, 8H). **^{13}C NMR** (126 MHz, $CDCl_3$) δ 172.9, 170.7, 119.4, 96.4, 65.1, 64.5, 60.3, 60.1, 53.4, 50.2, 45.8, 40.8, 36.8, 35.6, 34.7, 34.1, 33.3, 30.7, 30.6, 27.9, 22.6, 20.6, 14.6, 14.3, 11.5. **IR**: $\bar{\upsilon}$ 3402, 2932, 2855, 1652, 1039, 1207, 1094, 1053, 912, 835, 779, 712 cm^{-1}. **HRMS** (ESI) m/z: anal. calculated for $[C_{24}H_{36}O_5 + H]^+$: 405.2636, found: 405.2631.

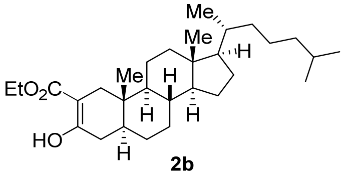

2b

2b, yield: (75%). **^{1}H NMR** (400 MHz, $CDCl_3$) δ 12.19 (s, 1H), 4.43 – 4.06 (m, 2H), 2.31 (d, *J* = 15.7 Hz, 1H), 2.13 (ddd, *J* = 18.6, 5.4, 1.3 Hz, 1H), 2.07 – 1.95 (m, 2H), 1.90 – 1.74 (m, 2H), 1.69 (dd, *J* = 12.8, 3.0 Hz, 1H), 1.64 – 0.94 (m, 25H), 0.92 (d, *J* = 6.5 Hz, 3H), 0.88 (dd, *J* = 6.6, 1.8 Hz, 6H), 0.75 (s, 3H), 0.68 (s, 3H). **^{13}C NMR** (101 MHz, $CDCl_3$) δ 173.0, 170.8, 96.5, 60.2, 56.4, 56.3, 53.6, 42.5, 40.9, 40.0, 39.5, 36.8, 36.2, 35.8, 35.4, 34.7, 33.4, 31.5, 28.2, 28.1, 28.0, 24.2, 23.8, 22.8, 22.6, 21.2, 18.7, 14.3, 12.0, 11.6. **IR**: $\bar{\upsilon}$ 3398, 2868, 1738, 1654, 1466, 1309, 1265, 1205, 1053, 941, 705 cm^{-1}. **HRMS** (ESI) m/z: anal. calculated for $[C_{30}H_{50}O_3 + Na]^+$: 481.3652, found: 481.3649.

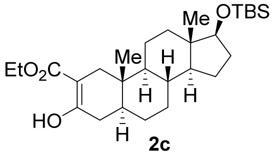

2c

2c, yield: (80%). **^{1}H NMR** (400 MHz, $CDCl_3$) δ 12.18 (s, 1H), 4.31 – 4.09 (m, 2H), 3.56 (t, *J* = 8.3 Hz, 1H), 2.32 (d, *J* = 15.7 Hz, 1H), 2.13 (ddd, *J* = 18.5, 5.5, 1.2 Hz, 1H), 2.08 – 1.95 (m, 1H), 1.88 (dtd, *J* = 13.2, 9.3, 5.7 Hz, 1H), 1.82 – 1.65 (m, 3H), 1.65 – 1.18 (m, 12H), 1.13 – 0.96 (m, 2H), 0.95 – 0.78 (m, 11H), 0.76 (s, 3H), 0.72 (s, 3H), 0.02 (s, 3H), 0.01 (s, 3H). **^{13}C NMR** (101 MHz, $CDCl_3$) δ 173.0, 170.7, 96.5, 81.8, 60.2, 54.0, 50.6, 43.2, 41.0, 37.2, 36.9, 35.5, 34.8, 33.4, 31.1, 30.9, 28.0, 25.9, 23.5, 20.9, 18.2, 14.3, 11.6, 11.3, -4.5, -4.8. **HRMS** (ESI) m/z: anal. calculated for $[C_{28}H_{48}O_4Si + H]^+$: 477.3395, found: 477.3397.

Syntheses of 3a, 3b and 4a.

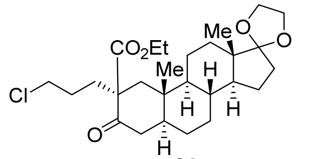

S2a

S2a, yield: (62%). **^{1}H NMR** (500 MHz, $CDCl_3$) δ 4.30 – 4.11 (m, 2H), 3.96 – 3.81 (m, 4H), 3.60 – 3.40 (m, 2H), 2.69 (t, J = 14.1 Hz, 1H), 2.62 (d, J = 13.8 Hz, 1H), 2.12 (dd, J = 14.1, 3.3 Hz, 1H), 2.00 (dddd, J = 28.7, 14.5, 10.5, 3.7 Hz, 2H), 1.79 (dtt, J = 14.1, 9.3, 5.2 Hz, 2H), 1.67 (dddd, J = 21.5, 15.0, 8.0, 3.8 Hz, 4H), 1.60 – 1.17 (m, 15H), 0.93 (s, 3H), 0.84 (s, 3H), 0.75 – 0.65 (m, 1H). **^{13}C NMR** (126 MHz, $CDCl_3$) δ 208.3, 173.3, 119.4, 65.3, 64.6, 61.5, 58.1, 54.5, 50.1, 50.0, 48.5, 46.0, 45.3, 44.4, 36.9, 35.3, 34.5, 34.2, 30.9, 30.6, 28.3, 28.0, 22.7, 21.1, 14.5, 14.1, 12.7. **IR**: $\bar{\upsilon}$ 3408, 2939, 1709, 1445, 1307, 1118, 1035, 952, 856, 736 cm^{-1}. **HRMS** (ESI) m/z: anal. calculated for $[C_{27}H_{41}ClO_5 + Na]^+$: 503.2535, found: 503.2537.

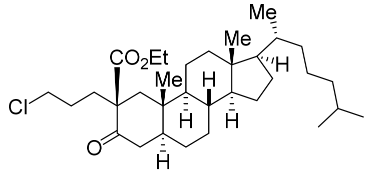

S2b

S2b, yield: (66%). **^{1}H NMR** (400 MHz, $CDCl_3$) δ 4.29 – 4.07 (m, 2H), 3.64 – 3.34 (m, 2H), 2.70 (t, J = 14.1 Hz, 1H), 2.61 (d, J = 13.8 Hz, 1H), 2.12 (dd, J = 14.1, 3.3 Hz, 1H), 2.09 – 1.93 (m, 2H), 1.89 – 1.74 (m, 2H), 1.74 – 1.62 (m, 2H), 1.59 – 1.40 (m, 6H), 1.42 – 0.96 (m, 21H), 0.93 (s, 3H), 0.91 (d, J = 6.5 Hz, 3H), 0.88 (d, J = 1.8 Hz, 3H), 0.86 (d, J = 1.8 Hz, 3H), 0.66 (s, 3H). **^{13}C NMR** (101 MHz, $CDCl_3$) δ 208.4, 173.2, 61.4, 58.1, 56.2, 56.2, 54.6, 49.9, 48.4, 45.2, 44.4, 42.6, 39.9, 39.5, 36.7, 36.1, 35.8, 34.9, 34.4, 31.6, 28.3, 28.2, 28.0, 27.9, 24.2, 23.8, 22.8, 22.5, 21.6, 18.6, 14.0, 12.6, 12.1. **IR**: $\bar{\upsilon}$ 3455, 2938, 2868, 1737, 1714, 1443, 1219, 1092, 1025, 736 cm^{-1}. **HRMS** (ESI) m/z: anal. calculated for $[C_{33}H_{55}ClO_3 + Na]^+$: 557.3732, found: 557.3721.

To a solution of **S2** (1.8 mol) in DMF (10 mL) was added NaN_3 (1.17 g, 18.0 mmol). The reaction mixture was heated at 80 ºC for 3 h. The mixture was cooled to room temperature, diluted with diethyl ether (150 mL), and washed with brine (50 mL × 3). The organic phase was dried over sodium sulfate and concentrated under vacuum to afford a crude product which was directly used in the next step without further purification.

The above keto azide was dissolved in trifluoroacetic acid (10.0 mL) and the solution was stirred at room temperature. Gas evolution occurred immediately. After 1 h, the reaction mixture was evaporated under vacuum to remove trifluoroacetic acid and diluted with 100 mL of ethyl acetate, and the solution was washed with saturated aqueous $NaHCO_3$ and brine (1 × 25 mL). The organic layer was dried over sodium sulfate and evaporated in vacuum to give an oil. The crude product was purified by flash chromatography to give the lactam **3a** or **3b** as yellow oil.

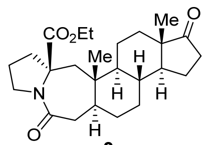

3a

3a, yield: 84%. **^{1}H NMR** (500 MHz, $CDCl_3$) δ 4.26 (dqd, *J* = 12.3, 7.1, 1.6 Hz, 1H), 4.20 – 4.08 (m, 1H), 3.76 (ddd, *J* = 11.3, 8.4, 2.9 Hz, 1H), 3.52 (ddd, *J* = 12.4, 9.5, 7.3 Hz, 1H), 2.85 – 2.69 (m, 2H), 2.44 (dd, *J* = 19.1, 8.6 Hz, 2H), 2.15 – 1.97 (m, 3H), 1.97 – 1.89 (m, 1H), 1.89 – 1.69 (m, 4H), 1.68 – 1.44 (m, 5H), 1.39 – 1.21 (m, 8H), 0.98 (qd, *J* = 12.7, 3.9 Hz, 1H), 0.86 (s, 3H), 0.82 (s, 3H), 0.73 (td, *J* = 11.3, 10.9, 3.6 Hz, 1H). **^{13}C NMR** (126 MHz, $CDCl_3$) δ 220.9, 174.4, 173.8, 67.8, 61.7, 55.0, 51.3, 50.5, 48.8, 47.4, 44.8, 42.8, 40.7, 39.7, 35.8, 33.9, 31.5, 30.6, 30.1, 21.7, 20.8, 20.7, 14.1, 13.8, 12.3. **IR**: *ν* 3456, 3002, 1665, 1441, 1266, 1102, 1082, 1043, 914, 734 cm^{-1}. **IR**: $\bar{\upsilon}$ 3451, 1736, 1631, 1439, 1365, 1266, 1227, 1205, 1123, 1048, 1024, 734 cm^{-1}. **HRMS** (ESI) m/z: anal. calculated for $[C_{25}H_{37}NO_4 + H]^+$: 416.2795, found: 416.2815.

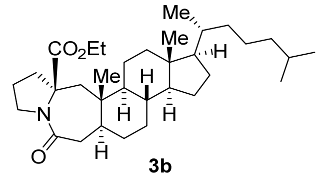

3b

3b, yield: 78%. **^{1}H NMR** (400 MHz, $CDCl_3$) δ 4.25 (dq, *J* = 10.8, 7.1 Hz, 1H), 4.13 (dq, *J* = 10.7, 7.1 Hz, 1H), 3.75 (ddd, *J* = 11.7, 8.3, 3.1 Hz, 1H), 3.51 (ddd, *J* = 11.8, 9.3, 7.1 Hz, 1H), 2.83 – 2.64 (m, 2H), 2.44 (ddd, *J* = 12.8, 6.5, 3.0 Hz, 1H), 2.15 – 1.93 (m, 3H), 1.78 (dtt, *J* = 16.6, 6.8, 3.8 Hz, 2H), 1.68 – 1.18 (m, 18H), 1.17 – 0.92 (m, 10H), 0.90 (d, *J* = 6.5 Hz, 3H), 0.87 (d, *J* = 1.9 Hz, 3H), 0.85 (d, *J* = 1.9 Hz, 3H), 0.78 (s, 3H), 0.64 (s, 3H). **^{13}C NMR** (101 MHz, $CDCl_3$) δ 174.5, 173.9, 67.8, 61.6, 56.4, 56.2, 55.0, 50.6, 48.8, 44.8, 42.8, 42.2, 40.9, 40.0, 39.5, 39.4, 36.1, 35.7, 34.3, 31.8, 30.4, 28.2, 28.0, 24.1, 23.8, 22.8, 22.5, 21.4, 20.8, 18.6, 14.0, 12.3, 12.0. **IR**: $\bar{\upsilon}$ 3454, 2946, 2868, 1632, 1444, 1366, 1151, 1121, 1025, 735, 701 cm^{-1}. **HRMS** (ESI) m/z: anal. calculated for $[C_{33}H_{55}NO_3 + H]^+$: 514.4255, found: 514.4256.

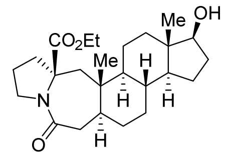

4a

Ketone **3a** (498 mg, 1.2 mmol) was dissolved in MeOH (10 ml) and cooled to −78 °C. $NaBH_4$ (67.2 mg, 2.4 mmol) was then added, and the solution was stirred for 3 h at this temperature. The reaction mixture was quenched with NH_4Cl solution, the mixture was diluted with ethyl acetate (100 mL), and washed with brine (50 mL × 2). The organic phase was dried over sodium sulfate and concentrated in vacuum. Flash column chromatography over silica column afford of **4a** as a white solid. Yield: (470 mg, 94%). **^{1}H NMR** (500 MHz, $CDCl_3$) δ 4.25 (dq, *J* = 10.8, 7.1 Hz, 1H), 4.13 (dq, *J* = 10.8, 7.1 Hz, 1H), 3.74 (ddd, *J* = 11.5, 8.1, 3.1 Hz, 1H), 3.62 (t, *J* = 8.6 Hz, 1H), 3.51 (ddd, *J* = 11.8, 9.3, 7.2 Hz, 1H), 2.87 – 2.65 (m, 2H), 2.44 (ddd, *J* = 12.8, 6.5, 3.0 Hz, 1H), 2.13 – 1.92 (m, 3H), 1.89 – 1.37 (m, 10H), 1.37 – 1.20 (m, 7H), 1.05 (td, *J* = 12.9, 4.2 Hz, 1H), 1.01 – 0.80 (m, 3H), 0.80 (s, 3H), 0.73 (s, 3H), 0.66 (ddd, *J* = 12.3, 10.2, 3.9 Hz, 1H). **^{13}C NMR** (126 MHz, $CDCl_3$) δ 174.5, 173.9, 81.8, 67.8, 61.6, 55.1, 50.9, 50.6, 48.8, 44.8, 42.9, 42.7, 40.8, 39.6, 36.8, 34.3, 31.3, 30.5, 30.3, 23.3, 21.0, 20.8, 14.1, 12.3, 11.1. **IR**: $\bar{\upsilon}$ 3395, 3052, 1731, 1622, 1445, 1265, 1066, 855, 702 cm^{-1}. **HRMS** (ESI) m/z: anal. calculated for $[C_{25}H_{39}NO_4 + H]^+$: 418.2952, found: 418.2960.

Syntheses of 5a-c.

To a solution of compound **2** (6.0 mol) in toluene (40 mL) was added NaH (288 mg, 7.2 mmol, 60%) under Ar. The reaction mixture was stirred for 2 h. Then DMAD (1.97 g, 12.0 mol) was added. The mixture was stirred for 30 mins. After quenching with NH_4Cl solution, the mixture was diluted with ethyl acetate (150 mL), and washed with brine (50 mL × 2). The organic phase was dried over sodium sulfate and concentrated in vacuum. Flash column chromatography over silica column afforded **5** as yellow oil.

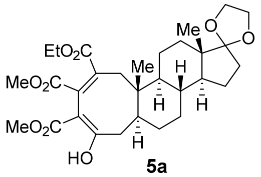

5a

5a, yield: (62%). **^{1}H NMR** (500 MHz, $CDCl_3$) δ 12.96 (d, J = 2.1 Hz, 1H), 4.34 – 4.06 (m, 2H), 3.98 – 3.82 (m, 4H), 3.71 (s, 6H), 3.33 (d, J = 13.0 Hz, 1H), 2.67 – 2.53 (m, 1H), 1.97 (ddd, J = 14.5, 11.6, 3.1 Hz, 1H), 1.88 (d, J = 12.9 Hz, 1H), 1.85 – 1.67 (m, 3H), 1.67 – 1.57 (m, 2H), 1.57 – 1.33 (m, 6H), 1.33 – 1.15 (m, 7H), 0.83 (s, 3H), 0.79 – 0.64 (m, 4H). **^{13}C NMR** (126 MHz, $CDCl_3$) δ 180.2, 171.6, 170.1, 168.2, 138.4, 133.5, 119.1, 98.2, 65.1, 64.5, 61.3, 52.2, 52.1, 52.0, 50.5, 46.7, 45.9, 41.5, 37.8, 36.8, 35.7, 34.3, 31.3, 30.8, 22.6, 22.6, 21.4, 14.5, 13.9, 11.4. **IR**: ῡ 3395, 2948, 1720, 1602, 1444, 1311, 1297, 1238, 1210, 1135, 1018, 950, 702, 673 cm^{-1}. **HRMS** (ESI) m/z: anal. calculated for $[C_{30}H_{42}O_9 + Na]^+$: 569.2721, found: 569.2707. LC-MS (t_R= 3.61 min, λ = 254 nm, purity >99%).

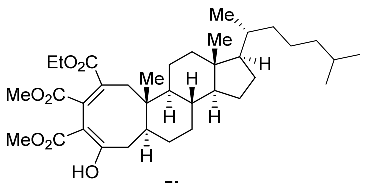

5b

5b, yield: (74%). **^{1}H NMR** (400 MHz, $CDCl_3$) δ 12.95 (t, J = 1.6 Hz, 2H), 4.34 – 4.04 (m, 2H), 3.71 (d, J = 1.2 Hz, 6H), 3.32 (d, J = 12.9 Hz, 1H), 2.58 (ddd, J = 12.7, 9.1, 2.1 Hz, 1H), 2.08 – 1.93 (m, 1H), 1.85 (dd, J = 21.3, 10.7 Hz, 2H), 1.80 – 1.41 (m, 8H), 1.41 – 1.19 (m, 11H), 1.19 – 0.93 (m, 9H), 0.92 – 0.82 (m, 9H), 0.71 (d, J = 1.3 Hz, 3H), 0.65 (d, J = 1.3 Hz, 3H). **^{13}C NMR** (101 MHz, $CDCl_3$) δ 180.3, 171.7, 170.1, 168.3, 138.7, 133.4, 98.2, 61.3, 56.7, 56.1, 52.4, 52.2, 51.9, 46.8, 42.5, 41.5, 40.1, 39.5, 37.7, 36.8, 36.1, 35.7, 35.4, 32.1, 31.4, 28.3, 28.0, 24.1, 23.8, 22.8, 22.5, 21.9, 18.6, 13.9, 12.2, 11.4. **IR**: ῡ 3433, 2936, 2867, 1654, 1600, 1358, 1296, 1235, 1214, 1173, 1018, 859, 675 cm^{-1}. **HRMS** (ESI) m/z: anal. calculated for $[C_{36}H_{56}O_7 + Na]^+$: 623.3918, found: 623.3904.

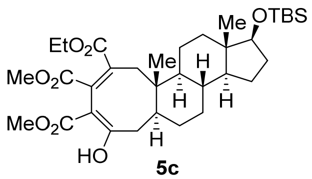

5c

5c, yield: (68%). **^{1}H NMR** (500 MHz, $CDCl_3$) δ 12.96 (d, J = 2.0 Hz, 1H), 4.25 (dq, J = 10.9, 7.2 Hz, 1H), 4.17 (dq, J = 10.7, 7.0 Hz, 1H), 3.71 (s, 6H), 3.54 (t, J = 8.3 Hz, 1H), 3.33 (d, J = 12.9 Hz,

1H), 2.59 (ddd, *J* = 12.8, 9.2, 2.2 Hz, 1H), 1.97 – 1.82 (m, 2H), 1.83 – 1.74 (m, 2H), 1.74 – 1.65 (m, 1H), 1.62 (d, *J* = 12.8 Hz, 1H), 1.57 – 1.14 (m, 11H), 1.02 – 0.79 (m, 13H), 0.71 (s, 3H), 0.69 (s, 3H), 0.00 (d, *J* = 4.3 Hz, 6H). **^{13}C NMR** (126 MHz, $CDCl_3$) δ 180.3, 171.7, 167.0, 168.3, 138.6, 133.5, 98.2, 81.6, 61.3, 52.7, 52.2, 52.0, 50.9, 46.9, 43.3, 41.5, 37.9, 37.3, 36.8, 35.5, 31.7, 31.3, 31.1, 25.8, 23.5, 21.6, 18.1, 13.9, 11.5, 11.4, -4.5, -4.8. **IR**: $\bar{\upsilon}$ 3371, 2953, 1722, 1654, 1312, 1212, 1173, 1020, 883, 797, 672 cm^{-1}. **HRMS** (ESI) m/z: anal. calculated for $[C_{34}H_{54}O_8Si + Na]^+$: 641.3480, found: 641.3457. LC-MS (t_R= 1.16 min, λ = 254 nm, purity >99%).

Syntheses of 6a and 6c.

5a or **5c** (4.0 mmol) was dissolved in AcOH (20 ml) and concentrated HCl (40 ml). The reaction mixture was heated at reflux for 48 h. The mixture was cooled to room temperature, diluted with ethyl acetate (200 mL), and washed with brine (50 mL × 2). The organic phase was dried over sodium sulfate and concentrated under vacuum. Flash column chromatography over silica column afforded **6a** or **6c**.

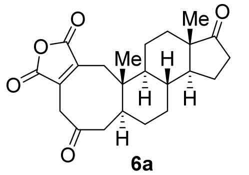

6a

6a, yield: (60%). **^{1}H NMR** (500 MHz, $CDCl_3$) δ 3.72 (dd, *J* = 18.4, 1.5 Hz, 1H), 3.39 (d, *J* = 18.3 Hz, 1H), 2.87 – 2.75 (m, 1H), 2.75 – 2.68 (m, 1H), 2.42 (ddd, *J* = 19.3, 8.9, 1.1 Hz, 1H), 2.26 (dd, *J* = 15.9, 2.2 Hz, 1H), 2.19 (ddd, *J* = 12.6, 6.3, 2.5 Hz, 2H), 2.04 (dt, *J* = 19.2, 9.0 Hz, 2H), 1.99 – 1.76 (m, 4H), 1.62 – 1.42 (m, 4H), 1.42 – 1.30 (m, 2H), 1.23 (tdd, *J* = 18.7, 7.8, 4.1 Hz, 3H), 0.98 (s, 3H), 0.85 (s, 3H), 0.67 (ddd, *J* = 12.1, 10.2, 3.6 Hz, 1H). **^{13}C NMR** (126 MHz, $CDCl_3$) δ 220.1, 206.4, 165.0, 164.1, 141.5, 140.7, 51.2, 50.0, 49.1, 47.5, 42.6, 40.2, 35.7, 35.0, 31.2, 31.0, 30.2, 29.3, 21.5, 20.9, 15.1, 13.8. **IR**: $\bar{\upsilon}$ 2924, 2852, 1768, 1454, 1366, 1264, 1216, 1049, 939, 913, 736 cm^{-1}. **HRMS** (ESI) m/z: anal. calculated for $[C_{23}H_{28}O_5 + H]^+$: 385.2010, found: 385.1999.

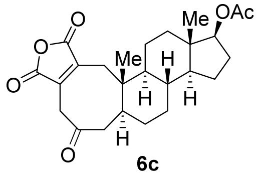

6c

6c, yield: (64%). **^{1}H NMR** (500 MHz, $CDCl_3$) δ 4.53 (dd, *J* = 9.2, 7.8 Hz, 1H), 3.71 (dd, *J* = 18.4, 1.5 Hz, 1H), 3.37 (d, *J* = 18.3 Hz, 1H), 2.79 (d, *J* = 14.6 Hz, 1H), 2.71 (dd, *J* = 15.9, 12.4 Hz, 1H), 2.24 (dd, *J* = 15.9, 2.2 Hz, 1H), 2.20 – 2.04 (m, 3H), 2.01 (s, 3H), 1.95 – 1.84 (m, 1H), 1.76 (dt, *J* = 12.9, 3.4 Hz, 1H), 1.68 (dq, *J* = 13.1, 3.5 Hz, 1H), 1.57 (dddd, *J* = 12.3, 9.2, 6.7, 3.5 Hz, 1H), 1.54 – 1.41 (m, 3H), 1.41 – 1.17 (m, 3H), 1.11 (td, *J* = 13.1, 3.9 Hz, 1H), 1.05 – 0.84 (m, 5H), 0.77 (s, 3H), 0.62 (ddd, *J* = 12.0, 10.2, 3.6 Hz, 1H). **^{13}C NMR** (126 MHz, $CDCl_3$) δ 206.7, 171.0, 165.0, 164.2, 141.6, 140.6, 82.4, 50.6, 49.9, 49.2, 42.6, 42.3 (two Carbon), 40.2, 36.5, 35.2, 31.0, 30.9, 29.4, 27.5, 23.3, 21.0, 21.0, 15.1, 12.1. **HRMS** (ESI) m/z: anal. calculated for $C_{25}H_{32}NaO_6$ $[M + H]^+$: 451.2091, found: 451.2099.

D-ring expansion of isosteviol.

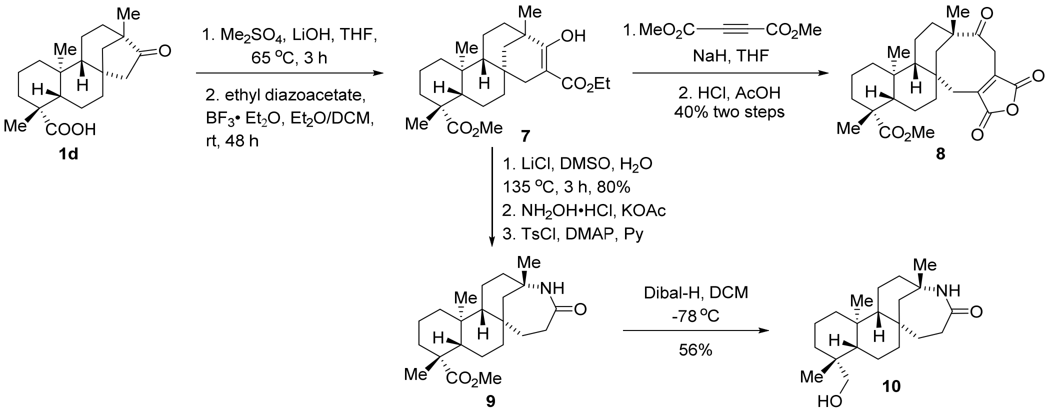

Supplementary Figure 6. Syntheses of 8-10. General procedure C was used for formation of **9**.

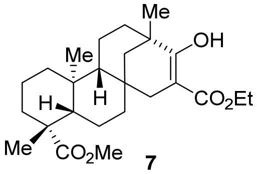

7

To a flame-dried 250 mL round-bottomed flask was added isosteviol **1d** (6.36 g, 20.0 mmol) and dry THF (100 mL). Upon dissolution, LiOH•H_2O (1.26 g, 30 mmol) was added and the reaction was stirred for 1 h at room temperature under an atmosphere of Ar. Me_2SO_4 (2.8 mL, 30.0 mmol) was slowly added, then a reflux condenser was fitted to the flask and the temperature was raised to 80 °C for 3 h. The mixture was cooled to room temperature, Et_3N (5.0 mL) was added and the mixture was stirred for 30 min. The reaction mixture was diluted with 200 mL of ethyl acetate, and the solution was washed with brine (3 × 50 mL). The organic layer was dried over sodium sulfate and evaporated under vacuum to give a white solid **S3**. Yield: (6.57 g, 99%).

Ketone **S3** (332 mg, 1.0 mol) was dissolved in dry DCM/Et_2O (1:1, 6.0 mL). BF_3•Et_2O (0.3 ml) and ethyl diazoethanoate (0.3 ml) was added. The reaction mixture was stirred for 48 h. The mixture was diluted with ethyl acetate (50 mL), and washed with $NaHCO_3$ (20 mL × 2) and brine (20 mL × 2). The organic phase was dried over sodium sulfate and concentrated under vacuum. Flash column chromatography over silica column afforded **7**. Yield: (264 mg, 63%). **^{1}H NMR** (500 MHz, $CDCl_3$) δ 12.31 (s, 1H), 4.22 (dddd, *J* = 17.9, 10.8, 7.1, 3.7 Hz, 2H), 3.66 (s, 3H), 2.86 (dd, *J* = 17.0, 1.9 Hz, 1H), 2.28 – 2.06 (m, 1H), 2.00 – 1.64 (m, 6H), 1.60 – 1.48 (m, 2H), 1.48 – 1.37 (m, 1H), 1.30 (t, *J* = 7.1 Hz, 3H), 1.27 – 1.22 (m, 3H), 1.18 (s, 3H), 1.13 – 0.96 (m, 7H), 0.85 (dd, *J* = 12.4, 3.9 Hz, 2H), 0.74 (s, 3H). **^{13}C NMR** (126 MHz, $CDCl_3$) δ 177.8, 175.9, 172.4, 97.5, 60.1, 58.3, 58.0, 51.2, 51.1, 45.5, 43.8, 40.0, 38.2, 37.9, 37.9, 37.1, 34.0, 32.0, 28.8, 23.8, 12.0, 19.7, 19.0, 14.4, 13.7. **HRMS** (ESI) m/z: anal. calculated for $[C_{25}H_{38}O_5 + Na]^+$: 441.2611, found: 441.2602.

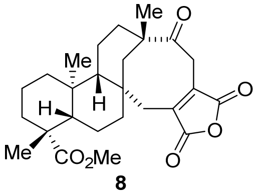

8

To a solution of compound **7** (4.18 g, 10.0 mol) in toluene (80 mL) was added NaH (480 mg, 12.0 mmol, 60%) under Ar. The reaction mixture was stirred for 2 h. Then DMAD (2.84 g, 2.0 mol) was added. The mixture was stirred for 20 min. After quenching with NH_4Cl solution, the mixture was diluted with ethyl acetate (200 mL), and washed with brine (50 mL × 2). The organic phase was dried over sodium sulfate and concentrated under vacuum. Flash column chromatography over short silica column afforded a yellow oil, which was directly used in the next step.

The above product was dissolved in AcOH (30 ml) and concentrated HCl (30 ml). The reaction mixture was heated at reflux for 48 h. The mixture was cooled to room temperature, diluted with ethyl acetate (200 mL), and washed with brine (50 mL × 2). The organic phase was dried over sodium sulfate and concentrated in vacuum. Flash column chromatography over silica column afforded **8**. Yield: (1.77 g, 40% for two steps). **^{1}H NMR** (500 MHz, $CDCl_3$) δ 3.91 (dd, *J* = 18.6, 1.7 Hz, 1H), 3.61 (s, 3H), 3.17 (dd, *J* = 18.6, 1.5 Hz, 1H), 2.84 (d, *J* = 14.1 Hz, 1H), 2.44 (dq, *J* = 13.6, 3.1 Hz, 1H), 2.27 – 1.96 (m, 3H), 1.87 (dd, *J* = 14.8, 2.7 Hz, 1H), 1.83 – 1.74 (m, 2H), 1.70 (dd, *J* = 13.0, 3.3 Hz, 1H), 1.58 (ddt, *J* = 14.1, 5.2, 2.8 Hz, 1H), 1.37 (ddt, *J* = 28.6, 13.5, 2.2 Hz, 3H), 1.12 (s, 3H), 1.10 (s, 3H), 1.06 – 1.00 (m, 2H), 1.00 – 0.86 (m, 4H), 0.81 (td, *J* = 13.1, 4.3 Hz, 1H), 0.61 (s, 3H). **^{13}C NMR** (126 MHz, $CDCl_3$) δ 209.9, 177.3, 165.4, 164.1, 142.9, 140.6, 58.4, 56.9, 51.0, 49.2, 48.2, 43.6, 40.0, 39.9, 39.4, 37.9, 37.2, 37.0, 34.1, 28.8, 28.3, 23.5, 19.2, 18.8, 18.0, 13.8. **IR**: $\bar{\upsilon}$ 3374, 3054, 1816, 1723, 1398, 1346, 1272, 1191, 1170, 1094, 1035, 974, 894, 853, 738 cm^{-1}. **HRMS** (ESI) m/z: anal. calculated for $[C_{26}H_{34}O_6 + Na]^+$: 465.2248, found: 465.2231.

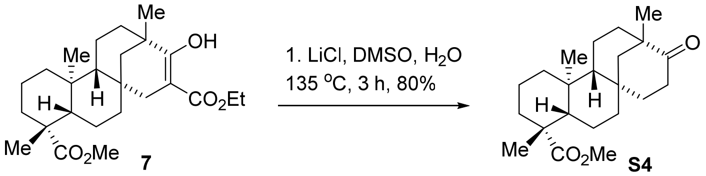

7 S4

To a solution of **7** (585 mg, 1.4 mol) in DMSO (10 ml) was added LiCl (616 mg, 14 mmol). The reaction mixture was heated at reflux for 3 h. The mixture was cooled to room temperature, diluted with ethyl acetate (150 mL), and washed with brine (50 mL × 3). The organic phase was dried over sodium sulfate and concentrated in vacuum. Flash column chromatography over silica column afforded **S4**. Yield: (388 mg, 80%). **^{1}H NMR** (500 MHz, $CDCl_3$) δ 3.64 (s, 3H), 2.55 – 2.38 (m, 1H), 2.29 – 2.11 (m, 3H), 1.82 (tdd, *J* = 19.7, 10.9, 5.2 Hz, 5H), 1.75 – 1.69 (m, 1H), 1.65 – 1.50 (m, 3H), 1.44 (dtd, *J* = 14.1, 4.5, 2.2 Hz, 1H), 1.32 – 1.16 (m, 5H), 1.16 – 1.08 (m, 2H), 1.08 – 0.92 (m, 6H), 0.87 (td, *J* = 13.2, 4.4 Hz, 1H), 0.73 (s, 3H). **^{13}C NMR** (126 MHz, $CDCl_3$) δ 219.3, 177.8, 57.8, 57.6, 51.3, 51.2, 45.4, 44.3, 43.8, 39.8, 38.5, 38.2, 38.0, 37.9, 34.3, 29.4, 28.7, 24.7, 20.6, 20.0, 18.9, 13.3. **IR**: $\bar{\upsilon}$ 2986, 1738, 1447, 1373, 1240, 121, 1047, 940, 747 cm^{-1}. **HRMS** (ESI) m/z: anal. calculated for $[C_{22}H_{34}O_3 + H]^+$: 347.2581, found: 347.2573.

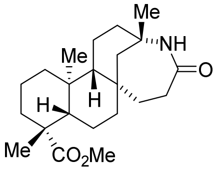

9

9, Yield: (78.0 mg, 60% for two steps). **^{1}H NMR** (500 MHz, $CDCl_3$) δ 5.20 (s, 1H), 3.64 (s, 3H),

2.55 (tt, J = 14.2, 2.2 Hz, 1H), 2.43 – 2.26 (m, 1H), 2.16 (td, J = 14.7, 14.1, 3.3 Hz, 2H), 2.04 – 1.70 (m, 5H), 1.70 – 1.49 (m, 5H), 1.49 – 1.40 (m, 1H), 1.33 (td, J = 12.6, 5.7 Hz, 1H), 1.17 (s, 3H), 1.16 (s, 3H), 1.13 – 0.91 (m, 4H), 0.85 (ddd, J = 22.3, 11.6, 4.0 Hz, 2H), 0.64 (s, 3H). **^{13}C NMR** (126 MHz, $CDCl_3$) δ 177.7, 175.9, 58.4, 57.2, 53.2, 51.2, 48.4, 43.8, 41.4, 41.4, 39.6, 38.1, 37.6, 35.3, 34.1, 32.5, 28.7, 28.3, 19.6, 18.9, 17.8, 13.8. **IR**: $\bar{\upsilon}$ 3055, 1736, 1640, 1422, 1265, 1046, 896 cm^{-1}. **HRMS** (ESI) m/z: anal. calculated for $[C_{22}H_{35}NO_3 + H]^+$: 362.2690, found: 362.2686. LC-MS (t_R= 2.46 min, λ = 210 nm, purity >99%).

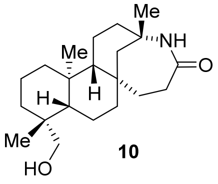

10

To a solution of lactam **9** (173 mg, 0.48 mol, 1.0 eq) in DCM (20 mL) was added DIBAL-H (1.0 ml, 1.0 mmol, 1 M) at -78 ºC under Ar. The reaction mixture was stirred for 30 min at this temperature. After quenching with 2 N HCl, the mixture was diluted with ethyl acetate (100 mL), and washed with brine (20 mL × 2). The organic phase was dried over sodium sulfate and concentrated in vacuum. Flash column chromatography over silica column afforded **10**. Yield: (89.5 mg, 56%). **^{1}H NMR** (500 MHz, $CDCl_3$) δ 5.65 (s, 1H), 3.72 (d, J = 10.8 Hz, 1H), 3.45 (d, J = 10.8 Hz, 1H), 2.89 (dd, J = 18.3, 2.4 Hz, 1H), 1.89 (d, J = 18.4 Hz, 1H), 1.84 – 1.70 (m, 3H), 1.70 – 1.20 (m, 15H), 1.18 (s, 3H), 1.04 – 0.95 (m, 4H), 0.95 – 0.88 (m, 4H), 0.84 (td, J = 13.1, 4.0 Hz, 1H). **^{13}C NMR** (126 MHz, $CDCl_3$) δ 173.7, 65.2, 57.7, 57.6, 51.8, 49.4, 44.6, 40.2, 39.8, 39.8, 38.6, 37.4, 35.6, 35.4, 28.9, 26.9, 18.6, 18.5, 18.0, 16.5. **IR**: $\bar{\upsilon}$ 3193, 2979, 2844, 1645, 1403, 1222, 1034, 953, 737 cm^{-1}. **HRMS** (ESI) m/z: anal. calculated for $[C_{21}H_{35}NO_2 + H]^+$: 334.2741, found: 334.2729.

D-ring expansion of dehydroepiandrosterone (DHEA) and estrone.

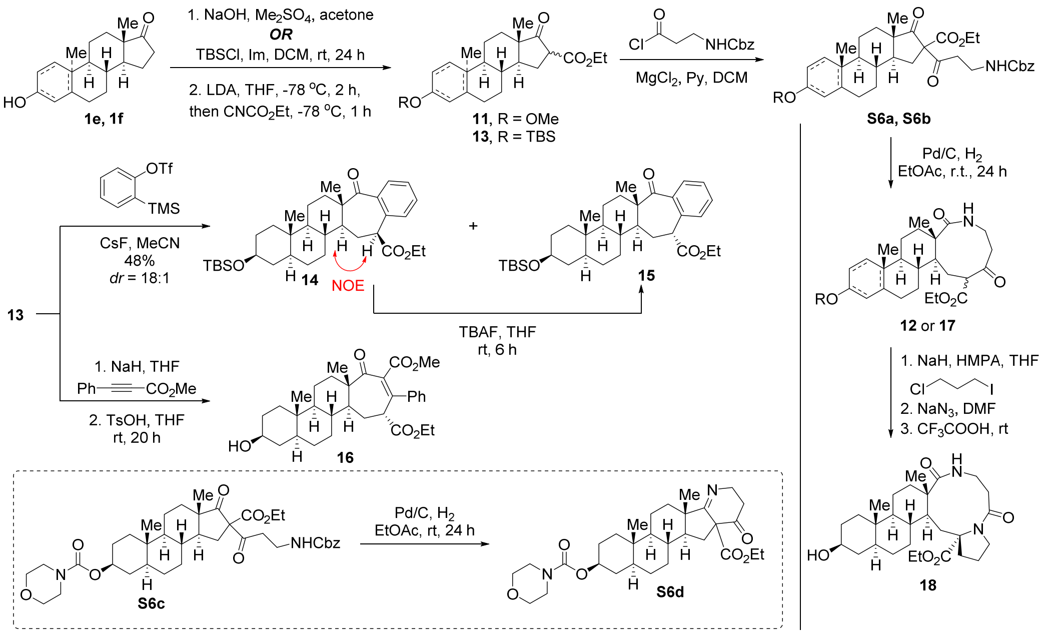

Supplementary Figure 7. Syntheses of medium ring scaffold 14-18. 11 and **13** were prepared as

general procedure A. Alkylation of **17** was performed as procedure B. The relative stereochemistry of **14** was confirmed by NOE experiment. The ring expansion failed to work when changing the TBS group of **S6b** to carbamate.

Syntheses of 12, 17 and 18.

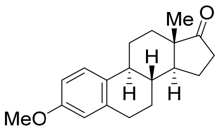

S5a

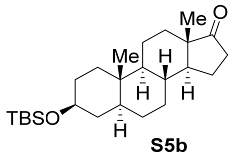

S5b

S5a[4] and **S5b**[5] were synthesized as reported by literature.

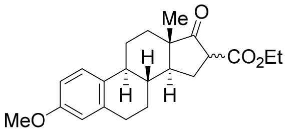

11

11, yield: (80%). **^{1}H NMR** (500 MHz, $CDCl_3$) δ 7.20 (d, *J* = 8.6 Hz, 1H), 6.72 (dd, *J* = 8.5, 2.7 Hz, 1H), 6.65 (d, *J* = 2.8 Hz, 1H), 4.21 (q, *J* = 7.1 Hz, 2H), 3.78 (s, 3H), 3.19 (dd, *J* = 9.9, 8.4 Hz, 1H), 2.91 (dd, *J* = 10.3, 6.2 Hz, 2H), 2.34 (dddd, *J* = 38.5, 22.8, 9.5, 3.7 Hz, 3H), 2.17 – 1.91 (m, 3H), 1.71 – 1.39 (m, 5H), 1.29 (t, *J* = 7.1 Hz, 3H), 0.99 (s, 3H). **^{13}C NMR** (126 MHz, $CDCl_3$) δ 212.2, 169.4, 157.6, 137.6, 131.7, 126.3, 113.8, 111.6, 61.4, 55.2, 54.3, 48.9, 48.0, 44.0, 37.9, 31.9, 29.6, 26.5, 26.4, 25.8, 14.2, 13.2. **IR**: $\bar{\upsilon}$ 3453, 2938, 1749, 1609, 1454, 1367, 1324, 1265, 1217, 1149, 1037, 900, 862, 785, 703 cm^{-1}. **HRMS** (ESI) m/z: anal. calculated for $[C_{22}H_{28}O_4 + Na]^+$: 379.1880, found: 379.1872.

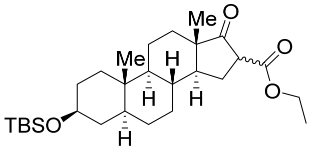

13

13, yield: (88%). **^{1}H NMR** (400 MHz, $CDCl_3$) δ 4.18 (q, *J* = 7.1 Hz, 2H), 3.68 – 3.34 (m, 1H), 3.10 (dd, *J* = 10.0, 8.4 Hz, 1H), 2.37 – 2.07 (m, 1H), 1.95 (td, *J* = 12.8, 10.0 Hz, 1H), 1.89 – 1.75 (m, 2H), 1.75 – 1.53 (m, 5H), 1.53 – 1.16 (m, 13H), 1.16 – 1.04 (m, 1H), 1.02 – 0.79 (m, 18H), 0.77 – 0.61 (m, 1H), 0.04 (s, 6H). **^{13}C NMR** (101 MHz, $CDCl_3$) δ 212.5, 169.5, 71.9, 61.3, 54.5, 54.3, 49.0, 48.7, 45.0, 38.5, 37.1, 35.7, 34.7, 32.0, 31.8, 30.9, 28.4, 26.6, 25.9, 20.4, 18.2, 14.1, 13.2, 12.3, -4.6. **IR**: $\bar{\upsilon}$ 3397, 2857, 1751, 1641, 1449, 1324, 1265, 1144, 1092, 1006, 868, 834, 736, 672 cm^{-1}. **HRMS** (ESI) m/z: anal. calculated for $[C_{28}H_{48}O_4Si + Na]^+$: 499.3214, found: 499.3195.

A mixture of **11** or **13** (5.28 mmol), $MgCl_2$ (1.03 g, 10.56 mmol) and pyridine (3.8 mL, 47.2 mmol) in DCM (30.0 mL) under an argon atmosphere was stirred at rt for 30 mins. Next, a solution of acid chloride (10.56 mmol) in DCM (15.0 mL) was added dropwise over 2 h and the resulting mixture was stirred for another two hours at rt. The mixture was then diluted with DCM (30 mL) and washed with 10% aq. HCl (30 mL). The aqueous layer was then extracted with DCM (2 × 30 mL) and the combined organic extracts dried over sodium sulfate and concentrated in vacuum. Flash

column chromatography over silica column afforded **S6** as yellow oil.

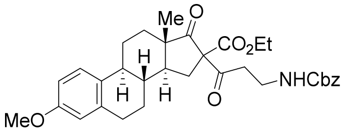

S6a

S6a, yield: (79%). **^{1}H NMR** (500 MHz, $CDCl_3$) δ 7.31 (dd, *J* = 20.9, 4.4 Hz, 5H), 7.15 (d, *J* = 8.6 Hz, 1H), 6.70 (dd, *J* = 8.6, 2.7 Hz, 1H), 6.63 (d, *J* = 2.8 Hz, 1H), 5.22 (t, *J* = 6.2 Hz, 1H), 5.07 (s, 2H), 4.20 (q, *J* = 6.9 Hz, 2H), 3.76 (s, 3H), 3.44 (q, *J* = 6.4 Hz, 2H), 3.03 (dt, *J* = 18.4, 5.7 Hz, 1H), 2.97 – 2.79 (m, 4H), 2.34 (dd, *J* = 12.6, 4.6 Hz, 1H), 2.21 (td, *J* = 10.9, 4.0 Hz, 1H), 2.13 – 1.80 (m, 3H), 1.69 – 1.37 (m, 5H), 1.25 (t, *J* = 7.1 Hz, 3H), 1.00 (s, 3H). **^{13}C NMR** (126 MHz, $CDCl_3$) δ 208.2, 199.8, 167.9, 157.5, 156.1, 137.4, 136.4, 131.5, 128.3, 127.9, 127.9, 126.1, 113.7, 111.5, 74.0, 66.5, 62.3, 55.0, 49.8, 47.2, 43.5, 40.1, 37.8, 35.9, 32.1, 30.5, 29.3, 26.3, 25.6, 14.2, 13.8. **IR**: $\bar{\upsilon}$ 3410, 1720, 1656, 1501, 1454, 1265, 1238, 1148, 1009, 736, 702 cm^{-1}. **HRMS** (ESI) m/z: anal. calculated for $[C_{33}H_{39}NO_7 + Na]^+$: 584.2619, found: 584.2609.

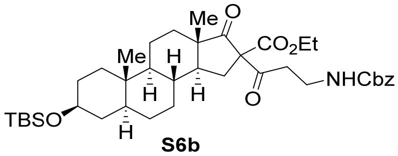

S6b

S6b, yield: (92%). **^{1}H NMR** (500 MHz, $CDCl_3$) δ 7.52 – 7.04 (m, 5H), 5.11 (d, *J* = 27.6 Hz, 3H), 4.20 (qd, *J* = 7.5, 4.4 Hz, 2H), 3.54 (tt, *J* = 10.3, 4.6 Hz, 1H), 3.44 (d, *J* = 7.3 Hz, 2H), 3.06 – 2.80 (m, 2H), 2.74 (dd, *J* = 13.0, 5.6 Hz, 1H), 1.93 (t, *J* = 13.1 Hz, 1H), 1.80 (ddd, *J* = 13.0, 10.1, 2.6 Hz, 2H), 1.75 – 1.59 (m, 3H), 1.59 – 1.13 (m, 13H), 1.15 – 1.01 (m, 2H), 0.96 (s, 3H), 0.89 (s, 9H), 0.81 (s, 3H), 0.68 (ddd, *J* = 14.8, 10.9, 4.1 Hz, 1H), 0.06 (s, 6H). **^{13}C NMR** (101 MHz, $CDCl_3$) δ 208.6, 200.1, 168.1, 156.1, 136.5, 128.4, 128.0, 74.0, 71.8, 66.5, 62.3, 54.2, 49.6, 48.2, 44.8, 40.1, 38.5, 37.0, 35.9, 35.6, 34.6, 32.2, 31.8, 30.9, 30.6, 28.2, 25.9, 20.3, 18.2, 14.2, 13.8, 12.2, -4.6. **IR**: $\bar{\upsilon}$ 3443, 2857, 1723, 1516, 1453, 1229, 1142, 863, 834, 775 cm^{-1}. **HRMS** (ESI) m/z: anal. calculated for $[C_{39}H_{59}NO_7Si + Na]^+$: 704.3953, found: 704.3943.

A mixture of compound **S6** (3.82 mmol) and Pd/C (0.30 g, 10%) in EtOAc (50 mL) was hydrogenated at rt for 24 h under hydrogen atmosphere. The suspension was filtered through a pad of celite and the pad was washed with CH_2Cl_2. The combined filtrates were concentrated to dryness. Flash column chromatography over silica column afforded **12** or **17** as a colorless oil.

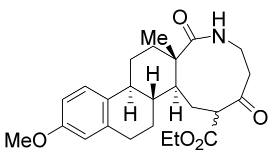

12

12, yield: (90%). **^{1}H NMR** (500 MHz, $CDCl_3$) δ 7.16 (d, *J* = 8.7 Hz, 1H), 6.72 (dd, *J* = 8.7, 2.7 Hz, 1H), 6.63 (d, *J* = 2.8 Hz, 1H), 5.67 (dd, *J* = 8.9, 4.8 Hz, 1H), 4.39 – 4.02 (m, 2H), 3.91 (ddd, *J* = 13.8, 8.9, 5.3 Hz, 1H), 3.78 (s, 3H), 3.54 (td, *J* = 13.2, 5.3 Hz, 1H), 3.44 – 3.23 (m, 2H), 2.87 (q, *J*

= 6.1, 5.1 Hz, 2H), 2.47 – 2.25 (m, 2H), 2.18 – 1.85 (m, 3H), 1.79 (dt, J = 13.1, 3.2 Hz, 1H), 1.67 – 1.29 (m, 5H), 1.25 (t, J = 7.2 Hz, 3H), 1.12 (s, 3H), 1.03 (dd, J = 10.5, 5.2 Hz, 1H). **^{13}C NMR** (126 MHz, $CDCl_3$) δ 209.8, 180.5, 168.6, 157.7, 137.3, 131.3, 126.2, 113.6, 111.9, 63.9, 61.6, 55.2, 48.7, 48.4, 43.5, 41.0, 38.8, 35.8, 34.0, 29.9, 27.4, 26.8, 25.7, 14.2, 14.1. **IR**: $\bar{\upsilon}$ 2990, 1666, 1502, 1442, 1366, 1266, 1228, 1216, 857, 743 cm^{-1}. **HRMS** (ESI) m/z: anal. calculated for $[C_{25}H_{33}NO_5 + H]^+$: 428.2432, found: 428.2427. LC-MS (t_R (major) = 2.52 min, t_R (minor) = 2.63 min, λ = 254 nm, purity 97%).

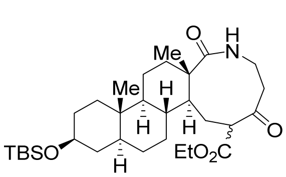

17

17, yield: (95%). **^{1}H NMR** (400 MHz, $CDCl_3$) δ 4.35 – 3.97 (m, 3H), 3.97 – 3.75 (m, 1H), 3.53 (tt, J = 10.5, 4.8 Hz, 1H), 2.79 (dd, J = 12.9, 6.4 Hz, 1H), 2.61 (ddd, J = 19.1, 5.6, 1.1 Hz, 1H), 2.22 (ddd, J = 19.1, 10.1, 7.1 Hz, 1H), 1.97 – 1.81 (m, 2H), 1.79 – 1.59 (m, 4H), 1.59 – 1.15 (m, 14H), 1.06 (s, 3H), 0.85 (d, J = 22.7 Hz, 15H), 0.68 – 0.57 (m, 1H), 0.04 (s, 6H). **^{13}C NMR** (101 MHz, $CDCl_3$) δ 202.3, 180.5, 167.0, 71.9, 66.2, 62.4, 54.4, 51.0, 46.9, 46.3, 44.9, 38.5, 37.1, 35.6, 34.7, 34.3, 33.6, 31.8, 31.1, 30.4, 28.4, 25.9, 20.6, 18.2, 14.5, 14.0, 12.3, -4.6. **HRMS** (ESI) m/z: anal. calculated for $[C_{31}H_{53}NO_5Si + H]^+$: 548.3766, found: 548.3750.

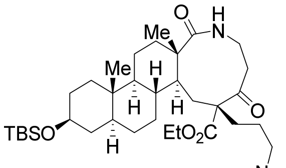

S7

To a solution of compound **17** (1.09 g, 2.0 mol) and HMPA (716 mg 4.0 mmol) in THF (20 mL) was added NaH (104 mg, 2.6 mmol, 60%) under Ar. The reaction mixture was stirred for 1 h. Then 1-Chloro-3-iodopropane (2.08 g, 10.0 mol) was added. The mixture was stirred for 24 h. After quenching with NH_4Cl solution, the mixture was diluted with ethyl acetate (60 mL), and washed with NaS_2O_3 (20 mL) and brine (20 mL × 2). The organic phase was dried over sodium sulfate and concentrated in vacuum to afford crude product which was directly used in the next step without further purification.

To a solution of the above product in DMF (10 mL) was added NaN_3 (1.17 g, 18.0 mmol). The reaction mixture was heated at 80 ºC for 3 h. The mixture was cooled to room temperature, diluted with diethyl ether (150 mL), and washed with brine (50 mL × 3), and the combined organic extracts was dried over sodium sulfate and concentrated in vacuum. Flash column chromatography over silica column afforded **S7** as a yellow oil. Yield: (756 mg, 60% for two steps). **^{1}H NMR** (500 MHz, $CDCl_3$) δ 5.57 (dd, J = 8.6, 5.0 Hz, 1H), 4.22 (dq, J = 10.9, 7.2 Hz, 1H), 4.18 – 4.03 (m, 2H), 3.73 (ddd, J = 13.4, 8.5, 5.0 Hz, 1H), 3.55 (tt, J = 10.4, 4.7 Hz, 1H), 3.47 (ddd, J = 15.3, 11.9, 5.2 Hz, 1H), 3.25 (dddd, J = 21.7, 18.9, 11.0, 5.7 Hz, 3H), 2.18 (dd, J = 15.3, 4.3 Hz, 1H), 2.11 – 1.98 (m, 1H), 1.95 – 1.74 (m, 3H), 1.74 – 1.57 (m, 4H), 1.57 – 1.15 (m, 15H), 1.15 – 1.04 (m, 4H), 1.00 – 0.84 (m, 10H), 0.84 – 0.72 (m, 4H), 0.05 (s, 6H). **^{13}C NMR** (126 MHz, $CDCl_3$) δ 210.5, 180.5, 171.9, 71.8, 65.1, 61.4, 53.5, 51.5, 49.0, 46.8, 44.6, 38.6, 38.3, 38.0, 37.0, 36.9, 36.2, 34.4, 33.1,

31.8, 31.8, 31.4, 28.7, 25.9, 25.0, 20.0, 18.2, 14.3, 14.1, 12.4, -4.6. **HRMS** (ESI) m/z: anal. calculated for $[C_{34}H_{58}N_4O_5Si + Na]^+$: 653.4069, found: 653.4061.

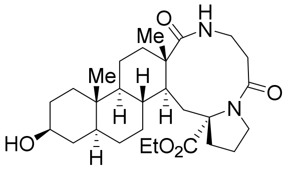

18

The keto azide **S7** (126 mg, 0.20 mmol) was dissolved in 5.0 mL of trifluoroacetic acid and the solution was stirred for 5 days. The reaction mixture was diluted with 30 mL of ethyl acetate, and the solution was washed with saturated aqueous $NaHCO_3$ and brine (1 × 25 mL). The organic layer was dried over sodium sulfate and evaporated under vacuum to give an oil. The crude product was purified by flash chromatography to give lactam **18** as a yellow oil. Yield: (33.2 mg, 34%). **^{1}H NMR** (500 MHz, $CDCl_3$) δ 5.88 (dd, *J* = 11.1, 2.3 Hz, 1H), 4.91 (tt, *J* = 11.5, 5.2 Hz, 1H), 4.38 (ddt, *J* = 13.7, 11.1, 9.1 Hz, 1H), 4.15 (dddd, *J* = 17.8, 10.7, 7.1, 3.7 Hz, 2H), 3.98 (td, *J* = 9.3, 8.3, 2.9 Hz, 1H), 3.50 (td, *J* = 9.5, 6.5 Hz, 1H), 3.00 (ddt, *J* = 13.7, 9.8, 1.9 Hz, 1H), 2.79 (ddd, *J* = 12.8, 9.0, 1.6 Hz, 1H), 2.64 (dd, *J* = 13.7, 3.3 Hz, 1H), 2.42 (dt, *J* = 12.8, 9.5 Hz, 1H), 2.20 – 2.00 (m, 3H), 1.91 (qt, *J* = 7.6, 4.1 Hz, 2H), 1.84 – 1.41 (m, 12H), 1.33 – 1.17 (m, 8H), 1.10 (s, 3H), 1.09 – 0.99 (m, 1H), 0.93 – 0.84 (m, 1H), 0.83 (s, 3H). **^{13}C NMR** (126 MHz, $CDCl_3$) δ 178.5, 172.9, 170.1, 78.5, 68.6, 60.6, 53.1, 49.9, 46.3, 44.7, 44.2, 41.1, 38.2, 37.8, 36.6, 36.4, 36.3, 35.9, 33.2, 32.2, 31.3, 28.9, 26.8, 23.9, 20.1, 15.2, 14.1, 12.1. **IR**: $\bar{\upsilon}$ 3427, 3055, 2855, 2099, 1738, 1664, 1366, 1265, 1134, 1019, 868, 703 cm^{-1}. **HRMS** (ESI) m/z: anal. calculated for $[C_{28}H_{44}N_2O_5 + H]^+$: 489.3323, found: 489.3310.

General protocol for syntheses of 14-16.

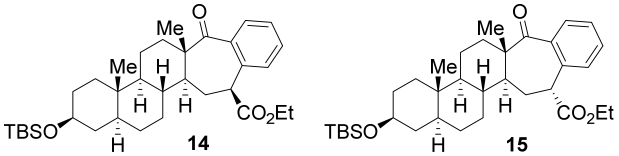

14 **15**

To a solution of **13** (514 mg, 1.08 mol,) in dry MeCN (10 mL) was added 2-(trimethylsilyl) phenyl trifluoromethanesulfonate (644 mg, 2.16 mmol) and CsF (328 mg, 2.16 mmol) under Ar. The reaction mixture was heated at 80 ºC for 3 h. The mixture was cooled to room temperature, diluted with ethyl acetate (50 mL), and washed with brine (20 mL × 2). The organic phase was dried over sodium sulfate and concentrated in vacuum. Flash column chromatography over silica column afforded **14** as white solid; Yield: (286 mg, 48%) and **15** as yellow oil. Yield: (16.1 mg, 2.7%). **14** can be converted to **15** by 1M TBAF in THF at room temperature for 6 h. **14**, **^{1}H NMR** (400 MHz, $CDCl_3$) δ 7.55 – 7.28 (m, 3H), 7.14 (dd, *J* = 7.6, 1.2 Hz, 1H), 4.17 – 3.94 (m, 2H), 3.87 (dd, *J* = 9.2, 4.6 Hz, 1H), 3.51 (td, *J* = 10.7, 5.4 Hz, 1H), 2.37 (ddd, *J* = 14.7, 9.2, 3.3 Hz, 1H), 2.03 (qd, *J* = 10.8, 10.4, 4.0 Hz, 2H), 1.76 – 1.53 (m, 5H), 1.53 – 1.08 (m, 16H), 1.04 – 0.81 (m, 11H), 0.78 (s, 3H), 0.68 – 0.55 (m, 1H), 0.04 (s, 6H). **^{13}C NMR** (101 MHz, $CDCl_3$) δ 213.8, 173.5, 140.2, 134.3, 130.5, 129.1, 128.0, 127.6, 71.9, 61.30, 53.4, 50.7, 49.5, 46.0, 44.2, 38.4, 37.6, 36.9, 35.6, 35.5, 31.7, 30.9, 28.6, 28.1, 25.9, 20.3, 18.2, 14.9, 13.9, 12.2, -4.6. **IR**: $\bar{\upsilon}$ 2944, 2865, 1447, 1366, 967, 902, 807, 836,

739 cm^{-1}. **HRMS** (ESI) m/z: anal. calculated for $[C_{34}H_{52}O_4Si + H]^+$: 553.3708, found: 553.3686. **15**, **^{1}H NMR** (500 MHz, $CDCl_3$) δ 7.39 (td, *J* = 7.6, 1.5 Hz, 1H), 7.30 (td, *J* = 7.4, 1.2 Hz, 1H), 7.28 – 7.18 (m, 1H), 7.09 (d, *J* = 7.6 Hz, 1H), 4.39 – 4.12 (m, 2H), 3.69 (dd, *J* = 12.6, 5.9 Hz, 1H), 3.47 (tt, *J* = 10.9, 4.7 Hz, 1H), 2.32 (td, *J* = 13.3, 5.4 Hz, 1H), 1.94 (dq, *J* = 12.9, 3.5 Hz, 1H), 1.72 (dd, *J* = 9.3, 3.0 Hz, 1H), 1.68 – 1.51 (m, 4H), 1.50 – 1.11 (m, 13H), 0.99 – 0.76 (m, 12H), 0.76 – 0.66 (m, 5H), 0.55 (td, *J* = 11.3, 3.5 Hz, 1H), 0.01 (s, 6H). **^{13}C NMR** (126 MHz, $CDCl_3$) δ 215.6, 172.7, 139.9, 134.3, 130.9, 127.3, 126.0, 124.3, 71.9, 61.0, 53.0, 49.0, 45.7, 44.1, 43.8, 38.3, 37.9, 36.9, 36.6, 35.5, 31.7, 31.3, 30.7, 28.5, 25.9, 20.5, 18.2, 14.1, 14.0, 12.2, -4.6. **HRMS** (ESI) m/z: anal. calculated for $[C_{34}H_{52}O_4Si + Na]^+$: 575.3527, found: 575.3505.

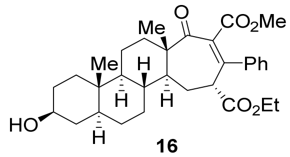

16

To a solution of compound **13** (143 mg, 0.3 mol,) in dry THF (10 mL) was added NaH (15.6 mg, 0.39 mmol, 60%) under Ar. The reaction mixture was stirred for 2 h. Then methyl phenylpropargylate (96 mg, 0.6 mol) was added. The mixture was heated at reflux for 24 h. The mixture was cooled to room temperature and 2 N HCl (2.0 ml) was added. The reaction mixture was heated at reflux for another 2 h. The mixture was cooled to room temperature, diluted with ethyl acetate (50 mL), and washed with $NaHCO_3$ (20 mL × 2) and brine (20 mL × 2). The organic phase was dried over sodium sulfate and concentrated in vacuum. Flash column chromatography over silica column afforded **16** as yellow oil; Yield: (76.2 mg, 46% for two steps). **^{1}H NMR** (500 MHz, $CDCl_3$) δ 7.40 (q, *J* = 5.2, 4.8 Hz, 3H), 7.26 – 7.09 (m, 2H), 3.94 – 3.84 (m, 1H), 3.84 – 3.73 (m, 1H), 3.68 (dq, *J* = 10.9, 5.2 Hz, 1H), 3.64 – 3.49 (m, 4H), 2.52 (td, *J* = 14.2, 13.4, 5.4 Hz, 1H), 2.29 – 2.14 (m, 1H), 2.03 – 1.60 (m, 10H), 1.60 – 1.17 (m, 11H), 1.13 – 0.95 (m, 5H), 0.89 – 0.86 (m, 4H). **^{13}C NMR** (126 MHz, $CDCl_3$) δ 210.6, 170.6, 163.8, 148.4, 136.5, 131.8, 128.3, 127.8, 127.8, 126.9, 71.1, 60.9, 53.2, 52.0, 49.3, 47.9, 44.4, 44.1, 37.9, 36.8, 36.7, 36.2, 35.6, 31.3, 30.9, 28.4, 27.9, 20.5, 13.7, 13.6, 12.2. **IR**: $\bar{\upsilon}$ 3456, 3016, 2944, 1435, 1229, 1046, 898, 737, 702 cm^{-1}. **HRMS** (ESI) m/z: anal. calculated for $[C_{32}H_{42}O_6 + H]^+$: 523.3054, found: 523.3042.

C-H oxidation/ring expansion of dehydroepiandrosterone, cholesterol, isosteviol, estrone and Diosgenin.

Syntheses of 14-16.

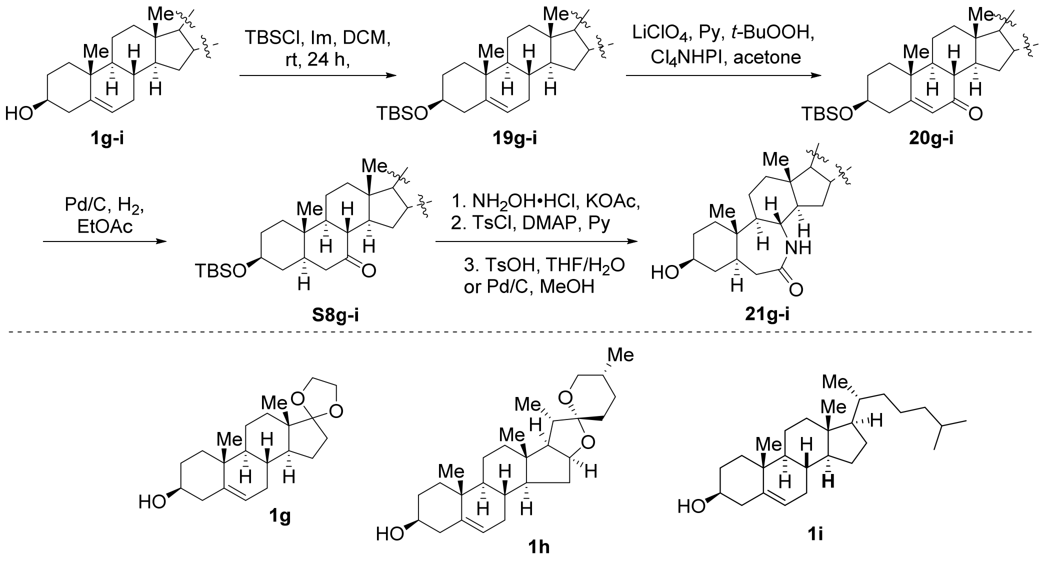

Supplementary Figure 8. Electrochemical allylic oxidation/ring expansion of natural products. **19g**,[6] **19h**[7] were synthesized as procedure reported by literature. The electrochemical allylic oxidation was performed by following procedures reported by Baran's group[8] using IKA ElectraSyn 2.0. General procedure C was used for Backman rearrangement of **S8**. TBS group of **21g** and **21i** was removed following procedure D, while TBS group of **21h** was removed following procedure E.

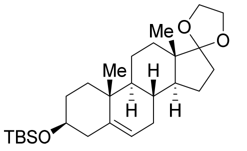

19g

19g, **^{1}H NMR** (500 MHz, $CDCl_3$) δ 5.32 (dt, J = 5.4, 2.0 Hz, 1H), 4.07 – 3.68 (m, 4H), 3.49 (tt, J = 10.9, 4.7 Hz, 2H), 2.36 – 2.22 (m, 1H), 2.17 (ddd, J = 13.3, 5.0, 2.3 Hz, 1H), 2.07 – 1.90 (m, 2H), 1.87 – 1.76 (m, 2H), 1.76 – 1.64 (m, 2H), 1.64 – 1.33 (m, 7H), 1.26 (qd, J = 11.9, 6.3 Hz, 1H), 1.13 – 0.93 (m, 5H), 0.90 (s, 9H), 0.87 (s, 3H), 0.06 (s, 6H). **^{13}C NMR** (126 MHz, $CDCl_3$) δ 141.5, 120.9, 119.5, 72.5, 65.2, 64.5, 50.63, 50.0, 45.7, 42.8, 37.4, 36.6, 34.2, 32.2, 32.1, 31.3, 30.6, 25.9, 22.8, 20.5, 19.4, 18.2, 14.2, -4.6. **HRMS** (ESI) m/z: anal. calculated for $C_{27}H_{46}O_3$ $[M + Na]^+$: 469.3108, found: 469.3118.

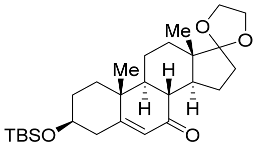

20g

20g, yield: 0.5 mmol scale, 68%, 2.5 mmol scale, 49%. **^{1}H NMR** (500 MHz, $CDCl_3$) δ 5.65 (d, J = 1.7 Hz, 1H), 4.01 – 3.78 (m, 4H), 3.68 – 3.52 (m, 1H), 2.53 – 2.32 (m, 3H), 2.30 – 2.16 (m, 1H), 2.00 – 1.87 (m, 2H), 1.87 – 1.70 (m, 3H), 1.69 – 1.34 (m, 8H), 1.18 (s, 3H), 0.88 (s, 9H), 0.85 (s, 3H), 0.05 (s, 6H). **^{13}C NMR** (126 MHz, $CDCl_3$) δ 201.6, 166.0, 125.7, 118.6, 71.2, 65.1, 64.4, 49.9, 46.1, 45.3, 44.3, 42.5, 38.3, 36.4, 34.1, 31.7, 29.6, 25.8, 25.0, 20.6, 18.1, 17.3, 14.4, -4.7, -4.7.

HRMS (ESI) m/z: anal. calculated for $[C_{27}H_{44}O_4Si + H]^+$: 461.3082, found: 461.3069.

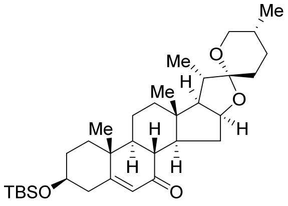

20h

20h, yield: 0.5 mmol scale, 58%. **^{1}H NMR** (500 MHz, $CDCl_3$) δ 5.75 – 5.52 (m, 1H), 4.48 (td, *J* = 8.0, 5.4 Hz, 1H), 3.60 (tt, *J* = 10.5, 4.8 Hz, 1H), 3.47 (ddd, *J* = 11.0, 4.5, 2.0 Hz, 1H), 3.40 (t, *J* = 10.9 Hz, 1H), 2.94 – 2.65 (m, 1H), 2.41 (dtd, *J* = 13.7, 10.8, 5.3 Hz, 3H), 1.96 – 1.78 (m, 3H), 1.77 – 1.35 (m, 13H), 1.21 (s, 3H), 1.15 (dt, *J* = 17.7, 5.2 Hz, 2H), 0.98 (d, *J* = 7.0 Hz, 3H), 0.89 (s, 9H), 0.80 (d, *J* = 7.0 Hz, 6H), 0.07 (s, 6H). **^{13}C NMR** (126 MHz, $CDCl_3$) δ 201.7, 166.0, 125.6, 109.2, 80.9, 71.2, 66.8, 61.1, 49.8, 49.5, 44.8, 42.6, 41.5, 40.9, 38.7, 38.5, 36.4, 33.7, 31.7, 31.4, 30.3, 28.8, 25.8, 20.9, 18.1, 17.3, 17.1, 16.4, 14.6, -4.7, -4.7. **IR**: $\bar{\upsilon}$ 3354, 1675, 1377, 1174, 1077, 981, 899, 851, 836, 773, 663 cm^{-1}. **HRMS** (ESI) m/z: anal. calculated for $[C_{33}H_{54}O_4Si + H]^+$: 543.3864, found: 543.3844.

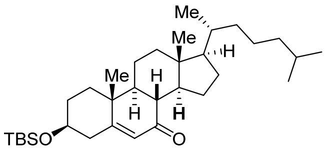

20i

20i, yield: 0.5 mmol scale, 70%. ^{1}H NMR (500 MHz, $CDCl_3$) δ 5.78 – 5.50 (m, 1H), 3.60 (tt, *J* = 10.5, 4.8 Hz, 1H), 2.39 (qdd, *J* = 14.0, 6.0, 2.0 Hz, 3H), 2.23 (dd, *J* = 12.5, 10.7 Hz, 1H), 2.03 (dt, *J* = 12.8, 3.5 Hz, 1H), 1.90 (dtt, *J* = 12.7, 7.1, 3.2 Hz, 2H), 1.82 (dq, *J* = 13.2, 3.7 Hz, 1H), 1.69 – 1.44 (m, 5H), 1.44 – 0.97 (m, 16H), 0.92 (d, *J* = 6.5 Hz, 3H), 0.91 – 0.88 (m, 9H), 0.87 (d, *J* = 2.4 Hz, 3H), 0.86 (d, *J* = 2.4 Hz, 3H), 0.68 (s, 3H), 0.06 (d, *J* = 0.8 Hz, 6H). ^{13}C NMR (126 MHz, $CDCl_3$) δ 202.4, 165.8, 125.8, 71.3, 54.8, 50.0, 49.92, 45.4, 43.0, 42.5, 39.4, 38.7, 38.3, 36.4, 36.2, 35.7, 31.7, 28.5, 28.0, 26.3, 25.8, 23.8, 22.8, 22.5, 21.2, 18.8, 18.1, 17.3, 11.9, -4.7, -4.7. **IR**: $\bar{\upsilon}$ 2977, 1739, 1422, 1365, 1265, 1228, 1216, 705 cm^{-1}. **HRMS** (ESI) m/z: anal. calculated for $[C_{33}H_{58}O_2Si + Na]^+$: 537.4098, found: 537.4089.

A mixture of compound **20** (6.0 mmol,) and Pd/C (100 mg, 10%) in EtOAc (100 mL) was hydrogenated at room temperature for 16 h under hydrogen atmosphere. The suspension was filtered through a pad of celite and the pad was washed with CH_2Cl_2. The combined filtrates were concentrated to dryness. Recrystallization of the crude reaction mixture with EtOAc give product **S8**.

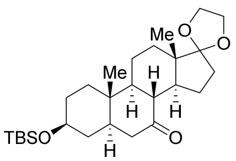

S8g

S8g, yield: 90%. **^{1}H NMR** (500 MHz, $CDCl_3$) δ 4.02 – 3.69 (m, 4H), 3.53 (tt, *J* = 9.7, 4.6 Hz, 1H),

2.42 – 2.20 (m, 3H), 2.11 – 1.95 (m, 1H), 1.97 – 1.68 (m, 5H), 1.63 (dt, *J* = 12.3, 3.6 Hz, 1H), 1.55 – 1.34 (m, 7H), 1.23 – 1.04 (m, 5H), 0.97 (td, *J* = 13.2, 3.8 Hz, 1H), 0.86 (s, 9H), 0.82 (s, 3H), 0.03 (d, *J* = 1.0 Hz, 6H). **^{13}C NMR** (126 MHz, $CDCl_3$) δ 211.3, 118.6, 71.4, 65.1, 64.4, 55.2, 50.0, 46.6, 46.0, 45.6, 43.3, 38.4, 36.3, 35.9, 34.0, 31.5, 29.7, 25.8, 23.7, 21.3, 18.1, 14.4, 11.8, -4.7. **IR**: $\bar{\upsilon}$ 3359, 1703, 1641, 1265, 1091, 948, 872, 835, 774, 735, 703 cm^{-1}. **HRMS** (ESI) m/z: anal. calculated for $[C_{27}H_{46}O_4Si + H]^+$: 463.3238, found: 463.3224.

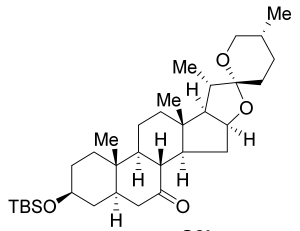

S8h

S8h, yield: 84%. **^{1}H NMR** (500 MHz, $CDCl_3$) δ 4.47 (td, *J* = 7.9, 6.2 Hz, 1H), 3.54 (tt, *J* = 10.4, 4.7 Hz, 1H), 3.46 (dt, *J* = 10.9, 3.2 Hz, 1H), 3.38 (t, *J* = 10.9 Hz, 1H), 2.58 (ddd, *J* = 12.8, 7.7, 5.5 Hz, 1H), 2.50 (t, *J* = 11.3 Hz, 1H), 2.43 – 2.26 (m, 1H), 2.01 (dd, *J* = 12.5, 2.3 Hz, 1H), 1.83 (p, *J* = 6.9 Hz, 1H), 1.79 – 1.34 (m, 16H), 1.21 – 1.02 (m, 6H), 1.02 – 0.92 (m, 4H), 0.87 (d, *J* = 1.1 Hz, 9H), 0.79 (d, *J* = 6.3 Hz, 3H), 0.75 (s, 3H), 0.04 (s, 6H). **^{13}C NMR** (126 MHz, $CDCl_3$) δ 211.5, 109.2, 80.8, 71.4, 66.8, 61.3, 55.3, 49.3, 48.5, 46.9, 46.0, 41.2, 40.4, 38.8, 38.4, 36.2, 36.1, 32.3, 31.5, 31.4, 30.3, 28.8, 25.8, 21.6, 18.2, 17.2, 16.5, 14.6, 11.9, -4.7. **IR**: $\bar{\upsilon}$ 2950, 1435, 1266, 1216, 1092, 1057, 981, 837 cm^{-1}. **HRMS** (ESI) m/z: anal. calculated for $[C_{33}H_{56}O_4Si + H]^+$: 545.4021, found: 545.4006.

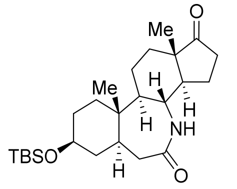

S9g

S9g, yield: 68%, **^{1}H NMR** (500 MHz, $CDCl_3$) δ 5.22 (s, 1H), 3.99 – 3.72 (m, 4H), 3.59 – 3.37 (m, 1H), 3.31 (ddd, *J* = 11.1, 9.0, 3.9 Hz, 1H), 2.65 (dd, *J* = 14.4, 10.1 Hz, 1H), 2.14 – 1.92 (m, 1H), 1.96 – 1.69 (m, 5H), 1.70 – 1.16 (m, 10H), 1.10 (ddd, *J* = 12.0, 9.2, 5.9 Hz, 1H), 1.07 – 0.97 (m, 1H), 0.97 (s, 3H), 0.85 (s, 9H), 0.83 (s, 3H), 0.02 (s, 6H). **^{13}C NMR** (126 MHz, $CDCl_3$) δ 177.5, 118.4, 70.9, 65.3, 64.5, 53.7, 52.3, 48.5, 45.6, 41.6, 41.2, 39.3, 39.3, 37.7, 33.6, 31.2, 29.4, 25.8, 23.4, 21.6, 18.1, 13.9, 12.8, -4.7, -4.7. **IR**: $\bar{\upsilon}$ 3225, 1447, 1337, 1228, 1051, 1007, 952, 875, 800, 775 cm^{-1}. **HRMS** (ESI) m/z: anal. calculated for $[C_{27}H_{47}NO_4Si + Na]^+$: 500.3167, found: 500.3148.

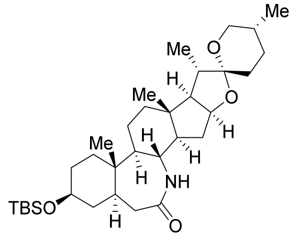

S9h

S9h, yield: 54%. **^{1}H NMR** (500 MHz, $CDCl_3$) δ 5.75 (d, *J* = 3.6 Hz, 1H), 4.38 (q, *J* = 7.3 Hz, 1H), 3.59 – 3.39 (m, 3H), 3.35 (t, *J* = 11.0 Hz, 1H), 2.69 (dd, *J* = 14.3, 10.3 Hz, 1H), 2.13 (ddd, *J* = 11.2, 7.7, 4.3 Hz, 1H), 2.00 – 1.52 (m, 11H), 1.50 – 1.25 (m, 9H), 1.20 – 1.06 (m, 2H), 0.99 (s, 3H), 0.95 (d, *J* = 6.4 Hz, 3H), 0.86 (s, 9H), 0.78 (d, *J* = 6.3 Hz, 3H), 0.76 (s, 3H), 0.02 (s, 6H). ^{13}C NMR (126 MHz, $CDCl_3$) δ 177.7, 109.2, 79.6, 70.9, 66.8, 61.8, 54.0, 53.5, 51.1, 41.7, 41.7, 41.2, 40.0, 39.4, 39.3, 38.8, 37.5, 32.4, 31.3, 31.2, 30.2, 28.7, 25.8, 22.1, 18.1, 17.0, 15.9, 14.4, 12.7, -4.6, -4.7. **IR**: $\bar{\upsilon}$ 3455, 3016, 1664, 1435, 1365, 1092, 981, 736 cm^{-1}. **HRMS** (ESI) m/z: anal. calculated for $[C_{33}H_{57}NO_4Si + Na]^+$: 582.3949, found: 582.3933.

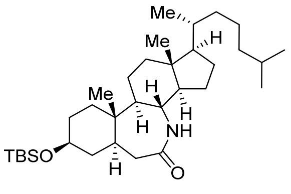

S9i

S9i, yield: 52%. **^{1}H NMR** (500 MHz, $CDCl_3$) δ 3.45 (ddd, *J* = 11.0, 6.3, 4.7 Hz, 1H), 3.25 (ddd, *J* = 10.7, 9.1, 4.1 Hz, 1H), 2.63 (dd, *J* = 14.3, 10.2 Hz, 1H), 1.95 – 1.77 (m, 3H), 1.77 – 1.54 (m, 8H), 1.54 – 0.89 (m, 21H), 0.86 (d, *J* = 6.5 Hz, 3H), 0.82 (d, *J* = 1.8 Hz, 11H), 0.81 (d, *J* = 2.6 Hz, 3H), 0.63 (s, 3H), -0.02 (s, 6H). **^{13}C NMR** (126 MHz, $CDCl_3$) δ 177.6, 70.9, 55.7, 54.2, 53.4, 52.0, 42.2, 41.5, 41.0, 39.3, 39.2, 39.1, 38.6, 37.6, 35.8, 35.4, 31.2, 27.8, 27.6, 25.8, 24.9, 23.6, 22.7, 22.4, 22.1, 18.4, 18.0, 12.7, 11.4, -4.7, -4.8. **IR**: $\bar{\upsilon}$ 3407, 2859, 1660, 1264, 1095, 1056, 1006, 874, 853, 799 cm^{-1}. **HRMS** (ESI) m/z: anal. calculated for $[C_{33}H_{61}NO_2Si + Na]^+$: 554.4364, found: 554.4379.

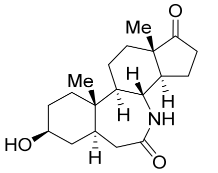

21g

21g, yield: 60%. **^{1}H NMR** (500 MHz, $CDCl_3$) δ 6.41 (s, 1H), 5.40 (dd, *J* = 5.1, 2.5 Hz, 1H), 3.54 (ddt, *J* = 16.0, 10.8, 4.7 Hz, 1H), 2.72 – 2.45 (m, 3H), 2.35 (ddd, *J* = 13.2, 5.0, 2.4 Hz, 1H), 2.31 – 2.09 (m, 5H), 2.11 – 1.84 (m, 5H), 1.69 (td, *J* = 12.1, 11.1, 6.3 Hz, 2H), 1.66 – 1.38 (m, 4H), 1.33 (s, 3H), 1.15 (td, *J* = 13.7, 3.6 Hz, 1H), 0.99 (s, 3H). **^{13}C NMR** (126 MHz, $CDCl_3$) δ 213.8, 175.0, 139.8, 120.4, 71.1, 61.5, 53.1, 46.8, 41.9, 37.7, 37.5, 36.5, 34.8, 32.1, 31.4, 31.0, 22.5, 18.5, 16.7. **IR**: $\bar{\upsilon}$ 3455, 2970, 1657, 1441, 1366, 1216, 1079, 1025, 897, 735 cm^{-1}. **HRMS** (ESI) m/z: anal. calculated for $C_{19}H_{30}NO_3$ $[M + H]^+$: 320.2200, found: 320.2209. LC-MS (t_R= 1.08 min, λ = 210 nm, purity >99%).

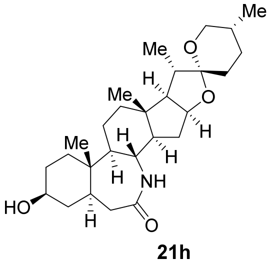

21h

21h, yellow solid. Yield: (860 mg, 84%). **^{1}H NMR** (500 MHz, $CDCl_3$) δ 5.31 (d, *J* = 6.4 Hz, 1H), 4.40 (q, *J* = 7.3 Hz, 1H), 3.58 (tt, *J* = 10.9, 4.7 Hz, 1H), 3.55 – 3.43 (m, 1H), 3.37 (t, *J* = 10.9 Hz, 1H), 2.72 (dd, *J* = 14.3, 10.6 Hz, 1H), 2.15 (ddd, *J* = 12.4, 7.5, 5.2 Hz, 1H), 1.98 – 1.73 (m, 7H), 1.65 (dddd, *J* = 35.2, 20.2, 10.3, 3.5 Hz, 4H), 1.54 – 1.22 (m, 9H), 1.21 – 1.00 (m, 7H), 0.97 (d, *J* = 6.5 Hz, 3H), 0.80 (d, *J* = 6.3 Hz, 3H), 0.78 (s, 3H). **^{13}C NMR** (126 MHz, $CDCl_3$) δ 177.3, 109.3, 79.6, 70.1, 66.9, 61.9, 54.2, 53.6, 51.1, 41.7, 41.5, 40.7, 40.1, 39.4, 39.3, 38.8, 37.4, 32.4, 31.3, 30.7, 30.2, 28.7, 22.1, 17.1, 16.0, 14.4, 12.7. **IR**: $\bar{\upsilon}$ 3016, 1649, 1267, 1092, 1060, 980, 896, 737 cm^{-1}. **HRMS** (ESI) m/z: anal. calculated for $[C_{27}H_{43}NO_4 + H]^+$: 446.3265, found: 446.3262.

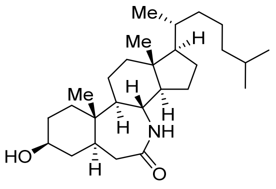

21i

21i, white solid, yield: (86%). **^{1}H NMR** ($CDCl_3$, 500 MHz) δ 5.33 (s, 1H), 3.60-3.54 (m, 1H), 3.31 (td, *J* = 9.8, 3.4 Hz, 1H), 2.69 (dd, *J* = 14.2, 10.9 Hz, 1H), 1.97-1.87 (m, 4H), 1.83-1.76 (m, 5H), 1.71-1.65 (m, 1H), 1.54-1.43 (m, 3H), 1.39-1.31 (m, 5H), 1.17-1.03 (m, 11H), 0.99 (s, 3H), 0.91 (d, *J* = 6.5 Hz, 3H), 0.87 (d, *J* = 2.5 Hz, 3H), 0.86 (d, *J* = 2.5 Hz, 3H), 0.69 (s, 3H). **^{13}C NMR** ($CDCl_3$, 125 MHz) δ 177.7, 70.2, 56.0, 54.6, 53.6, 52.2, 42.5, 41.6, 40.8, 39.5, 39.3, 38.8, 37.6, 36.0, 35.6, 30.8, 28.1, 27.8, 25.2, 23.8, 22.9, 22.6, 22.3, 18.6, 12.9, 11.7. **HRMS** (ESI): *m/z*: calculated for $C_{27}H_{48}NO_2$: $[M + H]^+$ 418.3680, found: 418.3687.

Procedure for synthesis of 24.

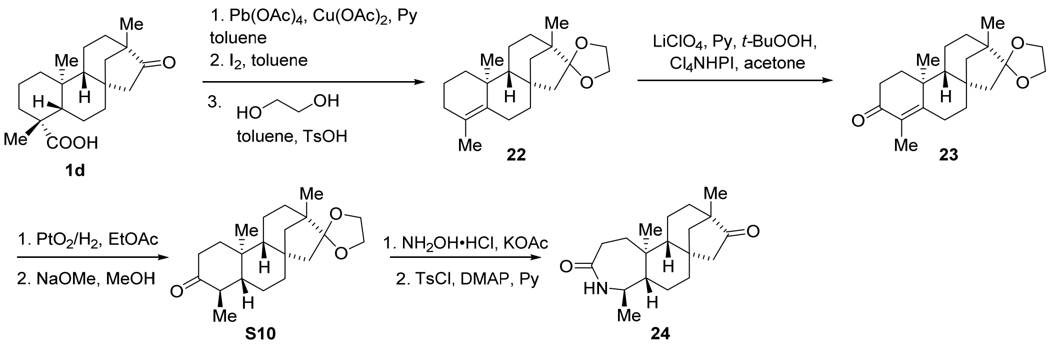

Supplementary Figure 9. Syntheses of 24. The relative stereochemistry of **S10** was confirmed by X-ray crystallography. The electrochemical allylic oxidation was performed following procedures reported by Baran's group[8] using IKA ElectraSyn 2.0. General procedure C was used for Backman rearrangement of **S10**.

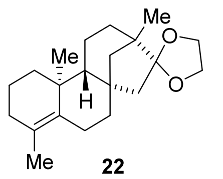

22

To a solution of **1d** (1.40 g, 4.44 mmol) in toluene (25 mL) were added lead (IV) acetate (2.56 g, 5.78 mmol), cooper(II) acetate (44 mg, 0.22 mmol), and pyridine (1.36 g, 16.9 mmol), and the

reaction mixture was stirred at 90 ºC for 15 min. Then it was diluted with ether (80 mL) and washed with 2 N HCl (3 × 20 mL), water (10 mL), sat. aq $NaHCO_3$ (2 × 10 mL), and brine, and the organic phase was dried over sodium sulfate. Removal of the solvent under vacuum afforded a crude product which was used in the next step without purification. To a stirred solution of this crude product in dry toluene (30 mL) was added iodine (16.6 mg, 0.05 mmol), and the reaction mixture was stirred at 90 ºC for 3 h. Then it was diluted with EtOAc (80 mL) and washed with $Na_2S_2O_3$ (20 mL) and brine, and the organic phase was dried over sodium sulfate. Removal of the solvent under vacuum afforded a crude product which was used in the next step without purification.

To a solution of above product and ethylene glycol (2.79 g, 45 mmol) in toluene (30 mL) was added p-toluenesulfonic acid (19.0 mg, 0.1 mmol). The reaction mixture was heated at reflux overnight with a Dean-Stark trap. The mixture was cooled to room temperature, diluted with ethyl acetate (60 mL), and washed with saturated aqueous sodium bicarbonate (10 mL × 2) and brine (10 mL × 2). The organic phase was dried over sodium sulfate and concentrated in vacuum. Flash column chromatography over silica column afforded **22**. Yield: (1.09 g, 78% for three steps). **^{1}H NMR** (500 MHz, $CDCl_3$) δ 4.09 – 3.63 (m, 4H), 2.57 – 2.23 (m, 2H), 1.97 (dt, *J* = 17.4, 8.8 Hz, 1H), 1.87 – 1.42 (m, 14H), 1.33 (td, *J* = 13.7, 3.9 Hz, 1H), 1.24 – 1.08 (m, 3H), 1.03 (s, 3H), 0.99 (d, *J* = 12.0 Hz, 1H), 0.86 (s, 3H). **^{13}C NMR** (126 MHz, $CDCl_3$) δ 136.9, 123.6, 118.5, 65.1, 64.0, 55.0, 54.46, 47.8, 45.2, 40.9, 40.7, 38.9, 38.0, 35.7, 32.7, 23.6, 21.3, 20.7, 20.1, 19.4, 18.7. **IR**: $\bar{\upsilon}$ 1737, 1440, 1365, 1265, 1228, 1216, 897, 742, 704 cm^{-1}. **HRMS** (ESI) m/z: anal. calculated for $[C_{21}H_{32}O_2 + H]^+$: 317.2475, found: 317.2466.

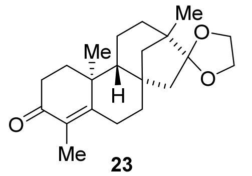

23

23, yield: 0.5 mmol scale, 68%. **^{1}H NMR** (500 MHz, $CDCl_3$) δ 4.04 – 3.71 (m, 4H), 2.62 (dt, *J* = 14.2, 3.5 Hz, 1H), 2.51 – 2.29 (m, 3H), 2.13 – 1.91 (m, 2H), 1.88 – 1.54 (m, 10H), 1.47 (td, *J* = 13.7, 3.4 Hz, 1H), 1.32 – 1.14 (m, 5H), 1.02 (dd, *J* = 11.5, 2.9 Hz, 1H), 0.88 (s, 3H). **^{13}C NMR** (126 MHz, $CDCl_3$) δ 198.9, 164.1, 128.0, 118.0, 65.2, 64.2, 54.6, 53.9, 47.7, 45.3, 40.6, 39.4, 39.1, 36.1, 35.2, 33.2, 26.0, 20.8, 19.9, 18.6, 11.0. **IR**: $\bar{\upsilon}$ 3457, 1665, 1449, 1366, 1275, 1228, 1054, 764, 663 cm^{-1}. **HRMS** (ESI) m/z: anal. calculated for $[C_{21}H_{30}O_3 + H]^+$: 331.2267, found: 331.2258.

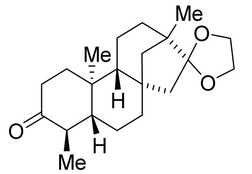

S10

A mixture of compound **23** (484 mg, 1.46 mmol,) and PtO_2 (100 mg) in EtOAc (20 mL) was hydrogenated at room temperature for 12 h under hydrogen atmosphere. The suspension was filtered through a pad of celite and the pad was washed with CH_2Cl_2. The combined filtrates were concentrated to dryness afforded of a crude product which was used in the next step without purification.

To a solution of above product in MeOH (10 mL) was added NaOMe (394 mg, 7.3 mmol)

under Ar atmosphere. The reaction mixture was stirred, diluted with ethyl acetate (80 mL), and washed with brine (10 mL × 2). The organic phase was dried over sodium sulfate and concentrated in vacuum. Flash column chromatography over silica column afforded **S10**. Yield: (349 mg, 72% for two steps). ^{1}**H NMR** (500 MHz, $CDCl_3$) δ 4.01 – 3.71 (m, 4H), 2.51 – 2.38 (m, 1H), 2.35 – 2.21 (m, 3H), 2.02 (ddd, *J* = 13.2, 6.7, 2.5 Hz, 1H), 1.75 (ddt, *J* = 12.7, 5.1, 2.7 Hz, 1H), 1.71 – 1.48 (m, 6H), 1.45 – 1.09 (m, 5H), 1.08 (s, 3H), 1.05 – 0.92 (m, 5H), 0.85 (s, 3H). ^{13}**C NMR** (126 MHz, $CDCl_3$) δ 213.5, 118.5, 65.1, 64.0, 54.9, 53.8, 53.7, 48.4, 45.1, 44.8, 40.3, 40.3, 39.4, 37.4, 36.9, 35.5, 23.6, 20.9, 20.0, 12.8, 11.7. **IR**: $\bar{\upsilon}$ 2948, 1453, 1153, 1098, 1044, 997, 950, 897, 837, 735, 707 cm^{-1}. **HRMS** (ESI) m/z: anal. calculated for $[C_{21}H_{32}O_3 + Na]^+$: 355.2244, found: 355.2230.

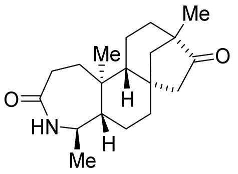

24

24, white solid. Yield: (100 mg, 40% for two steps). ^{1}**H NMR** (500 MHz, $CDCl_3$) δ 6.16 – 5.91 (m, 1H), 3.79 – 3.57 (m, 1H), 2.81 – 2.65 (m, 2H), 2.27 (ddt, *J* = 14.8, 7.6, 1.8 Hz, 1H), 2.02 – 1.84 (m, 3H), 1.77 – 1.53 (m, 5H), 1.54 – 1.22 (m, 7H), 1.21 (d, *J* = 6.8 Hz, 3H), 1.13 – 1.06 (m, 1H), 1.05 (d, *J* = 2.0 Hz, 2H), 1.01 (s, 3H). ^{13}**C NMR** (126 MHz, $CDCl_3$) δ 221.7, 177.0, 54.2, 53.9, 52.9, 48.5, 48.5, 46.8, 40.2, 39.6, 39.0, 37.1, 35.9, 30.8, 22.5, 20.6, 19.6, 19.4, 13.1. **IR**: $\bar{\upsilon}$ 3222, 1452, 1365, 1265, 1167, 1138, 1073, 977, 901, 731, 701 cm^{-1}. **HRMS** (ESI) m/z: anal. calculated for $[C_{19}H_{29}NO_2 + H]^+$: 304.2271, found: 304.2277.

Procedure for synthesis of 27a-c.

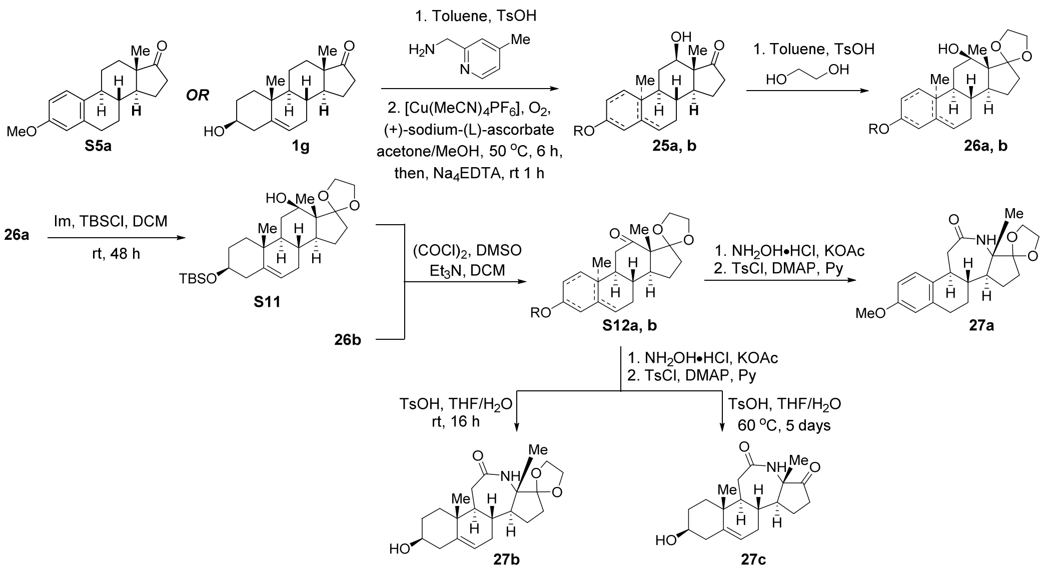

Supplementary Figure 10. Copper-mediated C-H oxidation/C-ring expansion of DHEA and estrone. 25a and **25b** were prepared following the procedures reported by Baran's group.[9] Protection the ketone group of **25** was realized using the standard ethylene glycol protection procedure. General procedure C was used for Backman rearrangement of **24.** The TBS of **27b** was

removed as procedure D. The ethylene glycol protection group of **27c** was removed by heating to reflux for 5 days.

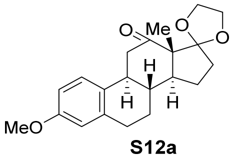

S12a

S12a, yield: (90% from **25a**). **^{1}H NMR** (500 MHz, $CDCl_3$) δ 7.05 (d, *J* = 8.7 Hz, 1H), 6.73 (dd, *J* = 8.6, 2.7 Hz, 1H), 6.68 (d, *J* = 2.9 Hz, 1H), 4.28 (q, *J* = 7.1 Hz, 1H), 4.13 (td, *J* = 6.7, 3.9 Hz, 1H), 4.01 (td, *J* = 6.8, 4.0 Hz, 1H), 3.90 (q, *J* = 7.0 Hz, 1H), 3.79 (s, 3H), 2.94 (dq, *J* = 9.8, 5.7, 4.8 Hz, 3H), 2.75 (td, *J* = 11.9, 5.0 Hz, 1H), 2.53 (dd, *J* = 15.2, 13.0 Hz, 1H), 2.17 (td, *J* = 11.9, 6.0 Hz, 1H), 2.12 – 2.00 (m, 2H), 1.94 – 1.73 (m, 3H), 1.58 – 1.39 (m, 2H), 1.13 (s, 3H). **^{13}C NMR** (126 MHz, $CDCl_3$) δ 209.3, 157.9, 137.7, 130.8, 126.1, 116.8, 114.0, 111.7, 65.8, 65.4, 58.2, 55.2, 48.8, 43.5, 43.4, 37.9, 34.6, 29.6, 26.4, 20.4, 15.0. **HRMS** (ESI) m/z: anal. calculated for $[C_{21}H_{26}O_4 + H]^+$: 343.1904, found: 343.1897.

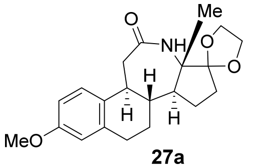

27a

27a, yield: (66% from **S12a**). **^{1}H NMR** (500 MHz, $CDCl_3$) δ 7.34 (d, *J* = 8.7 Hz, 1H), 6.75 (dd, *J* = 8.8, 2.8 Hz, 1H), 6.60 (d, *J* = 2.8 Hz, 1H), 6.10 (s, 1H), 4.12 (td, *J* = 7.3, 5.3 Hz, 1H), 4.05 – 3.88 (m, 3H), 3.78 (s, 3H), 3.20 – 3.00 (m, 1H), 2.80 (qd, *J* = 17.1, 16.5, 3.7 Hz, 4H), 2.12 (td, *J* = 11.2, 7.9 Hz, 1H), 2.04 – 1.67 (m, 5H), 1.56 – 1.13 (m, 5H). **^{13}C NMR** (126 MHz, $CDCl_3$) δ 175.7, 157.6, 138.6, 130.4, 128.8, 117.5, 113.5, 112.1, 64.8, 63.8, 61.1, 55.2, 51.2, 44.5, 43.6, 41.3, 30.7, 30.2, 26. 9, 23.7, 17.6. **IR**: $\bar{\upsilon}$ 3386, 3054, 1645, 1503, 1379, 1318, 1172, 1132, 1062, 1034, 917, 880, 812, 702 cm^{-1}. **HRMS** (ESI) m/z: anal. calculated for $[C_{21}H_{27}NO_4 + H]^+$: 358.2013, found: 358.2010. LC-MS (t_R= 1.85 min, λ = 254 nm, purity >99%).

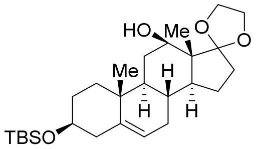

S11

S11, yield: (90% from **26a**). **^{1}H NMR** (500 MHz, $CDCl_3$) δ 5.41 – 5.19 (m, 1H), 3.99 (dq, *J* = 11.7, 6.0, 5.6 Hz, 4H), 3.88 (q, *J* = 6.0 Hz, 1H), 3.48 (tt, *J* = 10.5, 4.6 Hz, 1H), 2.39 – 2.09 (m, 2H), 2.09 – 1.87 (m, 2H), 1.88 – 1.63 (m, 6H), 1.63 – 1.30 (m, 6H), 1.13 – 0.97 (m, 5H), 0.93 (s, 3H), 0.89 (s, 9H), 0.06 (s, 6H). **^{13}C NMR** (126 MHz, $CDCl_3$) δ 141.5, 120.7, 119.1, 72.4, 71.3, 64.6, 64.1, 49.2, 49.1, 48.7, 42.7, 37.3, 36.7, 33.8, 32.0, 31.2, 30.8, 29.4, 25.9, 22.3, 19.4, 18.2, 8.8, -4.6, -4.6. **IR**: $\bar{\upsilon}$ 2857, 1436, 1306, 1092, 1034, 1001, 950, 887, 774, 704 cm^{-1}. **HRMS** (ESI) m/z: anal. calculated for $[C_{27}H_{46}O_4Si + Na]^+$: 485.3058, found: 485.3048.

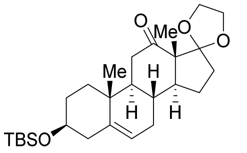

S12b

S12b, yield: (74% from **S11**). **^{1}H NMR** (500 MHz, $CDCl_3$) δ 5.37 (d, *J* = 5.1 Hz, 1H), 4.22 (q, *J* = 7.1 Hz, 1H), 4.11 (td, *J* = 6.6, 3.9 Hz, 1H), 3.98 (td, *J* = 6.8, 4.0 Hz, 1H), 3.87 (q, *J* = 7.0 Hz, 1H), 3.49 (tt, *J* = 10.6, 4.9 Hz, 1H), 2.45 (dd, *J* = 15.9, 12.8 Hz, 1H), 2.37 – 2.20 (m, 3H), 2.13 (dq, *J* = 17.1, 3.8, 2.8 Hz, 1H), 2.08 – 1.89 (m, 2H), 1.88 – 1.61 (m, 6H), 1.48 (dddd, *J* = 35.3, 17.3, 13.2, 8.1 Hz, 3H), 1.12 (s, 3H), 1.06 (s, 3H), 1.02 (dd, *J* = 13.6, 3.8 Hz, 1H), 0.90 (s, 9H), 0.07 (s, 6H). **^{13}C NMR** (126 MHz, $CDCl_3$) δ 210.7, 141.1, 120.6, 116.8, 72.1, 65.9, 65.4, 57.6, 50.7, 49.8, 42.6, 38.5, 36.9, 36.9, 34.4, 31.8, 31.2, 30.6, 25.9, 20.8, 19.0, 18.2, 15.1, -4.6. **IR**: $\bar{\upsilon}$ 3367, 2933, 1713, 1641, 1377, 1252, 1187, 1087, 954, 886, 834, 669 cm^{-1}. **HRMS** (ESI) m/z: anal. calculated for $[C_{27}H_{44}O_4Si + NH_4]^+$: 461.3082, found: 461.3066.

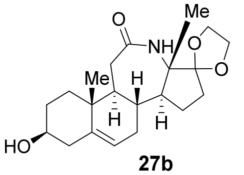

27b

27b, yield: (40% from **S11**). **^{1}H NMR** (500 MHz, $CDCl_3$) δ 6.16 (s, 1H), 5.36 (dt, *J* = 4.5, 2.2 Hz, 1H), 4.10 (tt, *J* = 4.8, 3.1 Hz, 1H), 4.05 – 3.82 (m, 3H), 3.53 (dt, *J* = 11.3, 6.2 Hz, 1H), 2.66 (t, *J* = 13.1 Hz, 1H), 2.52 – 2.38 (m, 1H), 2.32 (ddd, *J* = 13.1, 5.0, 2.4 Hz, 1H), 2.28 – 2.16 (m, 1H), 2.16 – 1.71 (m, 10H), 1.70 – 1.58 (m, 1H), 1.58 – 1.45 (m, 1H), 1.41 (s, 3H), 1.35 – 1.22 (m, 1H), 1.15 (td, *J* = 13.7, 3.7 Hz, 1H), 0.98 (s, 3H). **^{13}C NMR** (126 MHz, $CDCl_3$) δ 176.7, 139.6, 120.9, 117.7, 71.2, 64.7, 63.9, 60.4, 52.0, 46.7, 41.9, 37.6, 36.9, 36.6, 35.8, 31.5, 31.4, 30.2, 24.2, 18.4, 17.0. **IR**: $\bar{\upsilon}$ 3388, 2935, 1739, 1639, 1436, 1367, 1216, 1137, 1052, 951, 702 cm^{-1}. **HRMS** (ESI) m/z: anal. calculated for $[C_{21}H_{31}NO_4 + H]^+$: 362.2326, found: 362.2322.

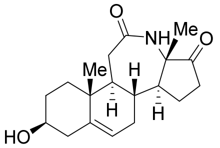

27c

27c, yield: (32% from **S11**). **^{1}H NMR** (500 MHz, $CDCl_3$) δ 6.41 (s, 1H), 5.40 (dd, *J* = 5.1, 2.5 Hz, 1H), 3.54 (ddt, *J* = 16.0, 10.9, 4.7 Hz, 1H), 2.73 – 2.46 (m, 3H), 2.35 (ddd, *J* = 13.2, 5.0, 2.4 Hz, 1H), 2.32 – 2.12 (m, 4H), 2.03 (dt, *J* = 13.4, 3.5 Hz, 1H), 1.93 (tdd, *J* = 18.9, 11.5, 2.8 Hz, 3H), 1.69 (td, *J* = 12.3, 11.1, 6.4 Hz, 2H), 1.64 – 1.48 (m, 2H), 1.43 (td, *J* = 10.7, 3.6 Hz, 1H), 1.33 (s, 3H), 1.15 (td, *J* = 13.7, 3.6 Hz, 1H), 0.99 (s, 3H). **^{13}C NMR** (126 MHz, $CDCl_3$) δ 213.8, 175.0, 139.8, 120.4, 71.1, 61.4, 53.1, 46.8, 41.9, 37.7, 37.5, 36.5, 34.8, 32.1, 31.4, 31.0, 22.5, 18.5, 16.7. **IR**: $\bar{\upsilon}$ 3318, 2928, 1656, 1449, 1372, 1265, 1167, 1120, 1024, 929, 823, 732, 662 cm^{-1}. **HRMS** (ESI) m/z: anal. calculated for $[C_{19}H_{27}NO_3 + H]^+$: 318.2064, found: 318.2056. LC-MS (t_R= 0.97 min, λ = 210 nm, purity >99%).

Procedure for synthesis of 29.

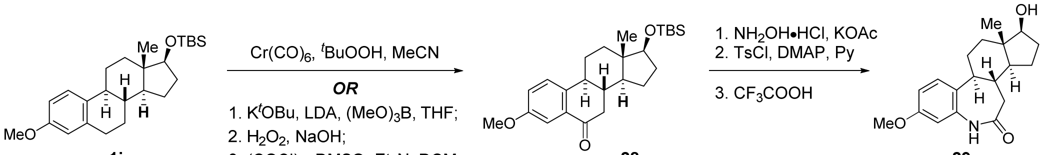

Supplementary Figure 11. Benzylic C-H oxidation.

1j was prepared following literature procedure.[10]

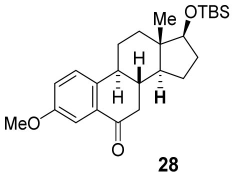

28

Procedure I:

To a solution of **1i** (1.42 g, 3.78 mmol) in acetonitrile (25 mL) was added tert-butyl hydroperoxide (1.14 mL, 11.40 mmol) and chromium hexacarbonyl (250 mg, 1.14 mmol). The mixture was boiled under reflux for 23 h and then cooled to room temperature. Water (100 mL) was added and the product was extracted with ether (3 × 20 mL). The extracts were washed with water, aqueous sodium hydrogen carbonate, and brine, dried ($MgSO_4$), and evaporated to give the crude product. Purification by flash chromatography afforded **28** as a white solid (469 mg, 30%).

Procedure II:

After a solution of *t*-BuOK (4.90 g, 44.5 mmol) in dry THF (50 ml) was cooled to −78 ºC, LDA (29.7 ml, 1.8 M, 44.5 mmol) was added and the mixture was stirred for 30 min at −78 ºC. Compound **1i** (4.4 g, 11.0 mmol) was then added to the reaction mixture and was stirred for 3h at −78 ºC. Trimethyl borate (15 ml) was added, and the reaction was warmed to 0 ºC, yielding a milky yellow suspension. After stirring for 2h, H_2O_2 (17 ml, 30% in water) was added, and the mixture was stirred for 1h at room temperatures. The reaction mixture was cooled to 0 ºC and 10% $Na_2S_2O_3$ (100 ml) was added. After extraction with ethyl acetate and drying over Na_2SO_4, flash column chromatography yielded the alcohol which was used directly for next step.

DMSO (2.48 g, 30.0 mmol) was dissolved in DCM (40 mL) and cooled to −78 ˚C. $(COCl)_2$ (7.5 ml, 2.0 M, 15.0 mmol) was then added dropwise, and the solution was stirred for 15 min, and the above product dissolved in DCM (20 mL) was added. The reaction was kept for 50 min at this temperature. Et_3N (6.06 g, 60 mmol) was added. The reaction was allowed to warm to rt. The mixture was diluted with ethyl acetate (100 mL), and washed with $NaHCO_3$ (30 ml × 2) and brine (50 mL × 2). The organic phase was dried over sodium sulfate and concentrated in vacuum. Flash column chromatography over silica column afforded **28**. Yield: (2.3 g, 50% for two steps). **^{1}H NMR** (400 MHz, $CDCl_3$) δ 7.56 (t, *J* = 2.3 Hz, 1H), 7.34 (d, *J* = 8.6 Hz, 1H), 7.11 (dt, *J* = 8.8, 2.3 Hz, 1H), 3.84 (s, 3H), 3.66 (t, *J* = 8.3 Hz, 1H), 2.74 (dt, *J* = 17.0, 2.4 Hz, 1H), 2.46 (td, *J* = 11.4, 4.6 Hz, 1H), 2.42 – 2.29 (m, 1H), 2.20 (ddd, *J* = 16.6, 13.6, 1.7 Hz, 1H), 2.03 – 1.81 (m, 3H), 1.74 – 1.41 (m, 3H), 1.30 (q, *J* = 13.0, 11.0 Hz, 3H), 0.90 (d, *J* = 1.7 Hz, 9H), 0.75 (d, *J* = 1.7 Hz, 3H), 0.04 (dd,

J = 5.5, 1.7 Hz, 6H). **^{13}C NMR** (101 MHz, $CDCl_3$) δ 198.2, 158.1, 139.8, 133.4, 126.6, 121.5, 109.5, 81.4, 55.5, 49.6, 44.1, 43.4, 43.1, 40.2, 36.6, 30.8, 25.8, 25.6, 23.0, 18.1, 11.2, -4.5, -4.8. **IR**: $\bar{\upsilon}$ 3372, 2956, 1737, 1608, 1418, 1319, 1170, 1070, 909, 867, 813, 770, 667 cm^{-1}. **HRMS** (ESI) m/z: anal. calculated for $[C_{25}H_{38}O_3Si + H]^+$: 415.2663, found: 415.2657.

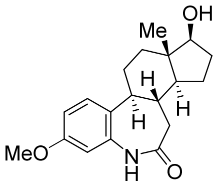

29

29, yield: 54% from **28**. **^{1}H NMR** (500 MHz, $CDCl_3$) δ 8.08 (s, 1H), 7.18 (d, J = 8.5 Hz, 1H), 6.76 (dd, J = 8.5, 2.6 Hz, 1H), 6.58 (d, J = 2.6 Hz, 1H), 4.87 (dd, J = 9.3, 7.6 Hz, 1H), 3.81 (s, 3H), 2.46 (dd, J = 12.7, 8.8 Hz, 2H), 2.37 – 2.24 (m, 1H), 2.15 (dd, J = 12.8, 2.1 Hz, 1H), 2.08 – 1.94 (m, 3H), 1.90 (dt, J = 12.9, 3.2 Hz, 1H), 1.83 – 1.62 (m, 3H), 1.42 (dtd, J = 40.7, 12.6, 5.1 Hz, 2H), 0.93 (s, 3H). **^{13}C NMR** (126 MHz, $CDCl_3$) δ 174.0, 158.6, 138.3, 129.2, 126.1, 112.0, 107.8, 86.2, 55.4, 48.8, 45.5, 44.0, 44.0, 36.4, 35.5, 26.9, 24.9, 23.5, 12.0. **IR**: $\bar{\upsilon}$ 3016, 1435, 1366, 1228, 1091, 899, 764 cm^{-1}. **HRMS** (ESI) m/z: anal. calculated for $[C_{19}H_{25}NO_3 + H]^+$: 316.1907, found: 316.1899. LC-MS (t_R= 2.92 min, λ = 254 nm, purity 95%).

C-H oxidation/ring expansion of picfeltarraegenin and kirenol.

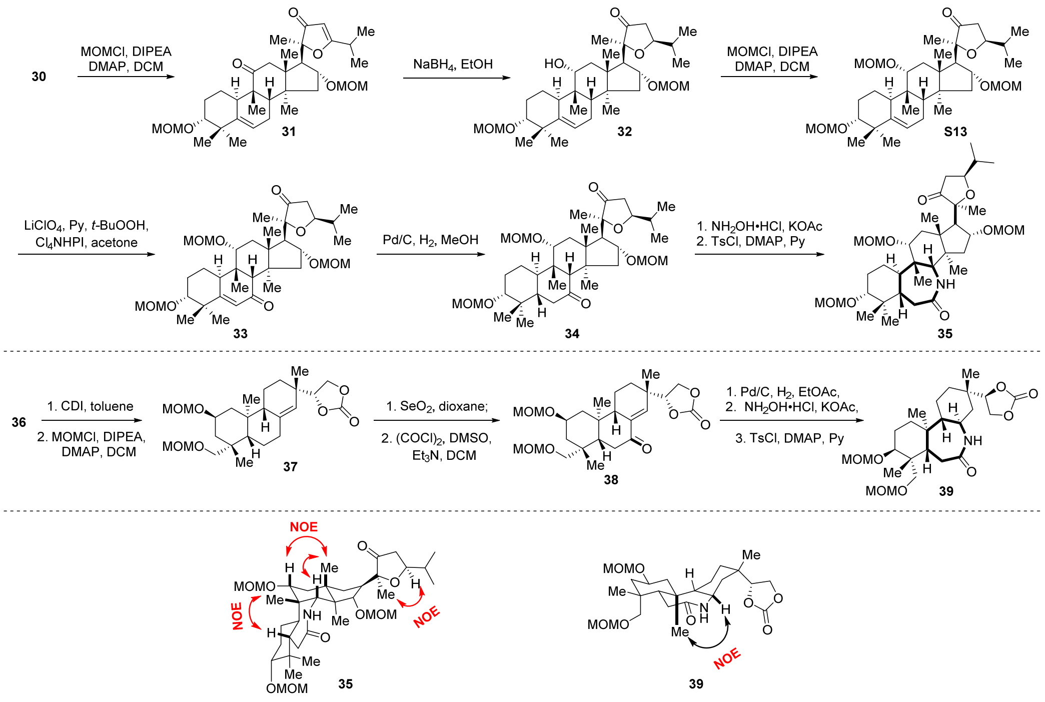

Supplementary Figure 12. C-H oxidation/ring expansion of picfeltarraegenin and kirenol. The electrochemical allylic oxidation was performed following procedure reported by Baran's group[8] using IKA ElectraSyn 2.0. General procedure C was used for Backman rearrangement. The relative stereochemistry of **35** and **39** was confirmed by NOE experiment.

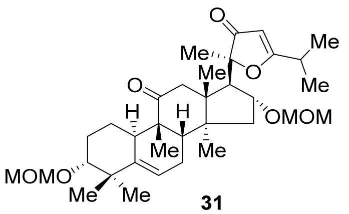

31

To a solution of **30** (2.42 g, 5.0 mol,) in DCM (60 mL) was added MOMCl (1.21 g, 15.0 mmol), DIPEA (2.58 g, 20.0 mmol) and DMAP (12.2 g, 0.10 mmol). The reaction mixture was heated at reflux for 48 h. The mixture was cooled to room temperature, diluted with ethyl acetate (80 mL), and washed with 2N HCl (20 mL × 2), $NaHCO_3$ (20 ml × 2) and brine (20 mL × 2). The organic phase was dried over sodium sulfate and concentrated in vacuum. Flash column chromatography over silica column afforded **31** as a yellow oil. Yield: (2.29 g, 80%). **^{1}H NMR** (500 MHz, $CDCl_3$) δ 5.66 (dt, *J* = 6.4, 2.2 Hz, 1H), 5.37 (s, 1H), 4.70 (d, *J* = 6.8 Hz, 1H), 4.57 (d, *J* = 6.8 Hz, 1H), 4.52 (d, *J* = 6.9 Hz, 1H), 4.40 (d, *J* = 6.9 Hz, 1H), 3.91 (t, *J* = 7.6 Hz, 1H), 3.36 (s, 3H), 3.32 (s, 3H), 3.16 (d, *J* = 14.6 Hz, 1H), 3.07 (dd, *J* = 11.5, 4.3 Hz, 1H), 2.83 – 2.71 (m, 1H), 2.69 (d, *J* = 6.9 Hz, 1H), 2.50 (d, *J* = 14.4 Hz, 1H), 2.35 (ddt, *J* = 19.1, 8.3, 2.7 Hz, 1H), 2.19 (d, *J* = 12.6 Hz, 1H), 1.98 – 1.82 (m, 4H), 1.76 – 1.59 (m, 3H), 1.55 (d, *J* = 13.5 Hz, 1H), 1.38 (s, 3H), 1.29 – 1.20 (m, 9H), 1.15 (s, 3H), 1.04 (s, 3H), 0.94 (s, 3H), 0.85 (s, 3H). **^{13}C NMR** (126 MHz, $CDCl_3$) δ 213.2, 206.5, 195.8, 142.1, 117.9, 100.3, 97.4, 95.8, 90.3, 82.4, 78.1, 56.5, 56.1, 55.5, 49.3, 48.7, 48.1, 47.6, 42.9, 42.5, 41.9, 35.3, 30.2, 27.4, 24.8, 24.4, 23.7, 22.1, 21.2, 20.1, 19.6, 19.6, 19.6, 19.5, 18.5. **IR**: $\bar{\upsilon}$ 3455, 2946, 1591, 1440, 1365, 1267, 1145, 1045, 1001, 916, 805, 734, 702 cm^{-1}. **HRMS** (ESI) m/z: anal. calculated for $[C_{34}H_{52}O_7 + H]^+$: 573.3786, found: 573.3768.

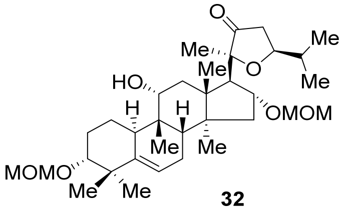

32

To a solution of **31** (2.29 g, 4.0 mol,) in EtOH (100 mL) was added $NaBH_4$ (2.28 g, 60.0 mmol) at 0 ºC. The reaction mixture was stirred at rt for 24 h. After quenching with NH_4Cl solution, EtOH was removed in vacuum then the reaction mixture was diluted with ethyl acetate (200 mL), and washed with brine (40 mL × 2). The organic phase was dried over sodium sulfate and concentrated in vacuum. Flash column chromatography over silica column afforded **32** as a yellow oil. Yield: (1.38 g, 60%). **^{1}H NMR** (500 MHz, $CDCl_3$) δ 5.68 (dt, *J* = 4.3, 2.1 Hz, 1H), 4.72 (dd, *J* = 8.4, 6.7 Hz, 2H), 4.66 (d, *J* = 6.5 Hz, 1H), 4.57 (td, *J* = 8.4, 7.6, 2.1 Hz, 3H), 4.02 – 3.84 (m, 1H), 3.36 – 3.43 (m, 7H), 3.12 – 2.99 (m, 2H), 2.68 – 2.54 (m, 2H), 2.37 (ddt, *J* = 19.0, 8.1, 2.6 Hz, 1H), 2.19 (ddd, *J* = 11.8, 7.4, 4.2 Hz, 2H), 1.97 – 1.81 (m, 3H), 1.80 – 1.54 (m, 4H), 1.41 (dt, *J* = 13.5, 10.2 Hz, 1H), 1.36 – 1.20 (m, 8H), 1.16 (s, 3H), 1.04 (s, 3H), 0.93 (d, *J* = 7.4 Hz, 6H), 0.88 (s, 3H), 0.86 (d, *J* = 6.7 Hz, 3H). **^{13}C NMR** (126 MHz, $CDCl_3$) δ 213.8, 142.1, 118.0, 97.0, 95.8, 85.0, 82.9, 82.4, 79.8, 79.0, 56.8, 55.5, 54.0, 49.8, 48.5, 48.4, 47.9, 42.7, 41.9, 41.5, 39.8, 35.3, 33.5, 27.5, 26.5, 24.8, 24.4, 23.7, 21.2, 20.1, 19.6, 18.8, 18.8, 18.7. **HRMS** (ESI) m/z: anal. calculated for $C_{34}H_{56}O_7$ $[M + Na]^+$: 599.3918, found: 599.3910.

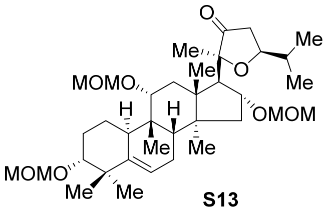

S13

To a solution of **32** (1.00 g, 1.74 mol,) in DCM (40 mL) was added MOMCl (431 mg, 5.22 mmol), DIPEA (1.12 g, 8.7 mmol) and DMAP (12.2 mg, 0.10 mmol). The reaction mixture was heated at reflux for 48 h. The mixture was cooled to room temperature, diluted with ethyl acetate (80 mL), and washed with 2N HCl (20 mL × 2), $NaHCO_3$ (20 ml × 2) and brine (20 mL × 2). The organic phase was dried over sodium sulfate and concentrated in vacuum. Flash column chromatography over silica column afforded **S13** as a yellow oil. Yield: (906 mg, 84%). **^{1}H NMR** (500 MHz, $CDCl_3$) δ 5.68 (dt, *J* = 6.2, 2.1 Hz, 1H), 4.73 – 4.50 (m, 6H), 4.41 (t, *J* = 7.4 Hz, 1H), 3.83 (dd, *J* = 6.1, 4.7 Hz, 1H), 3.45 (q, *J* = 7.6 Hz, 1H), 3.41 – 3.32 (m, 9H), 3.07 (dd, *J* = 11.6, 4.3 Hz, 1H), 3.01 (d, *J* = 14.6 Hz, 1H), 2.77 (d, *J* = 6.7 Hz, 1H), 2.46 (d, *J* = 14.6 Hz, 1H), 2.36 (ddt, *J* = 19.0, 8.0, 2.6 Hz, 1H), 2.20 (td, *J* = 13.4, 6.7 Hz, 2H), 1.94 (dd, *J* = 19.1, 6.1 Hz, 1H), 1.87 (dd, *J* = 9.7, 6.0 Hz, 2H), 1.83 – 1.63 (m, 5H), 1.60 (d, *J* = 13.2 Hz, 1H), 1.41 (dt, *J* = 13.2, 10.2 Hz, 1H), 1.26 (s, 3H), 1.23 (s, 3H), 1.15 (s, 3H), 1.03 (s, 3H), 0.95 (d, *J* = 6.8 Hz, 3H), 0.94 (s, 3H), 0.85 (d, *J* = 6.9 Hz, 3H), 0.84 (s, 3H). **^{13}C NMR** (126 MHz, $CDCl_3$) δ 214.6, 142.0, 118.2, 97.0, 96.7, 95.8, 86.1, 85.3, 82.5, 80.4, 79.1, 55.9, 55.7, 55.7, 55.5, 49.7, 48.7, 48.6, 47.1, 43.2, 43.0, 41.9, 36.2, 35.4, 33.8, 27.5, 25.7, 24.9, 24.4, 23.9, 21.2, 20.6, 20.2, 19.8, 19.0, 18.5. **IR**: $\bar{\upsilon}$ 3455, 2950, 1692, 1267, 1228, 1146, 1099, 1037, 917, 736 cm^{-1}. **HRMS** (ESI) m/z: anal. calculated for $[C_{36}H_{60}O_8 + Na]^+$: 643.4180, found: 643.4160.

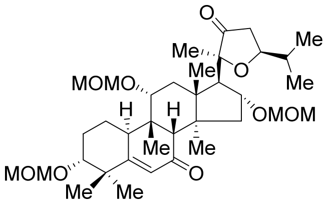

33

33, yield: 0.4 mmol scale, 58%. **^{1}H NMR** (500 MHz, $CDCl_3$) δ 6.19 (d, *J* = 2.1 Hz, 1H), 4.73 (d, *J* = 6.8 Hz, 1H), 4.67 – 4.53 (m, 4H), 4.47 (dd, *J* = 8.0, 6.5 Hz, 1H), 3.88 (dd, *J* = 6.3, 4.5 Hz, 1H), 3.52 – 3.42 (m, 1H), 3.41 – 3.31 (m, 8H), 3.24 (dd, *J* = 11.2, 4.4 Hz, 1H), 3.03 – 2.91 (m, 1H), 2.68 (dd, *J* = 10.8, 4.4 Hz, 2H), 2.62 (ddd, *J* = 12.9, 4.7, 2.1 Hz, 1H), 2.46 (s, 1H), 2.24 (dt, *J* = 13.3, 6.7 Hz, 1H), 2.16 (dd, *J* = 14.2, 8.3 Hz, 1H), 1.99 (dt, *J* = 13.1, 4.0 Hz, 1H), 1.88 (dq, *J* = 13.9, 3.9 Hz, 1H), 1.83 – 1.65 (m, 4H), 1.52 (d, *J* = 14.0 Hz, 2H), 1.28 (s, 3H), 1.27 – 1.13 (m, 7H), 1.08 (s, 3H), 1.06 (s, 3H), 0.96 (d, *J* = 6.6 Hz, 3H), 0.90 (s, 3H), 0.85 (d, *J* = 6.7 Hz, 3H). **^{13}C NMR** (126 MHz, $CDCl_3$) δ 211.9, 200.8, 167.8, 124.1, 96.4, 96.4, 95.9, 85.3, 85.3, 81.2, 80.4, 78.3, 58.5, 56.4, 56.1, 55.8, 55.7, 49.2, 48.7, 48.6, 47.0, 43.3, 42.6, 37.6, 36.2, 33.7, 27.0, 26.6, 24.0, 24.0, 22.2, 21.1, 20.8, 19.8, 19.0. **IR**: $\bar{\upsilon}$ 3442, 2887, 1696, 1618, 1468, 1383, 1294, 1268, 1146, 1100, 880, 735, 702 cm^{-1}. **HRMS** (ESI) m/z: anal. Calculated for $[C_{36}H_{58}O_9 + H]^+$: 635.4154, found: 635.4146.

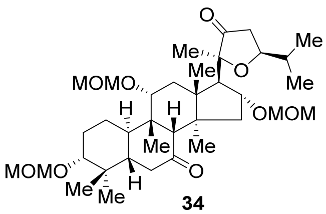

34

A mixture of compound **33** (546 mg, 0.86 mmol,) and Pd/C (100 mg) in MeOH (10 mL) was hydrogenated at room temperature for 4 h under hydrogen atmosphere. The suspension was filtered through a pad of celite and the pad was washed with CH_2Cl_2. The combined filtrates were concentrated to dryness. Flash column chromatography over silica column afforded **34** as yellow oil. Yield: (491 mg, 90%). **^{1}H NMR** (500 MHz, $CDCl_3$) δ 4.71 (d, *J* = 6.8 Hz, 1H), 4.67 – 4.50 (m, 5H), 4.45 (dd, *J* = 8.0, 6.4 Hz, 1H), 3.86 (dd, *J* = 6.3, 4.8 Hz, 1H), 3.47 (q, *J* = 7.6 Hz, 1H), 3.43 – 3.31 (m, 9H), 3.07 (dd, *J* = 11.6, 4.2 Hz, 1H), 2.96 – 2.85 (m, 1H), 2.71 (d, *J* = 6.4 Hz, 1H), 2.68 – 2.53 (m, 2H), 2.42 (s, 1H), 2.34 (dd, *J* = 17.6, 12.3 Hz, 1H), 2.22 (dt, *J* = 13.3, 6.6 Hz, 1H), 2.19 – 2.05 (m, 2H), 1.96 (s, 1H), 1.85 (dt, *J* = 12.5, 3.8 Hz, 1H), 1.82 – 1.62 (m, 2H), 1.58 (dd, *J* = 12.0, 5.8 Hz, 1H), 1.56 – 1.49 (m, 1H), 1.50 – 1.31 (m, 5H), 1.28 – 1.10 (m, 4H), 1.01 – 0.92 (m, 8H), 0.88 (s, 3H), 0.87 – 0.78 (m, 6H). **^{13}C NMR** (126 MHz, $CDCl_3$) δ 213.6, 213.1, 96.6, 96.7, 95.7, 85.678, 85.2, 82.8, 80.5, 78.3, 62.1, 56.0, 55.8, 55.8, 55.6, 51.3, 49.6, 49.3, 46.6, 43.3, 43.3, 41.1, 39.1, 36.1, 34.7, 33.7, 26.8, 26.2, 25.1, 25.1, 22.7, 20.6, 20.0, 19.8, 19.0, 13.7. **HRMS** (ESI) m/z: anal. calcd for $C_{36}H_{60}O_9$ $[M + Na]^+$: 659.4130, found: 659.4116.

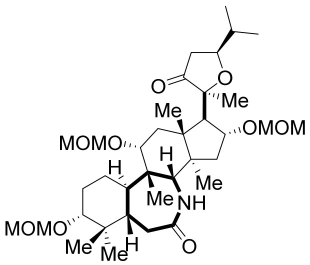

35

35, yield: 40%. **^{1}H NMR** (500 MHz, $CDCl_3$) δ 6.11 (t, *J* = 6.8 Hz, 1H), 4.74 – 4.53 (m, 6H), 4.49 (t, *J* = 7.3 Hz, 1H), 3.84 (t, *J* = 6.2 Hz, 1H), 3.45 (td, *J* = 8.3, 6.5 Hz, 1H), 3.36 (s, 6H), 3.35 (s, 3H), 3.09 (d, *J* = 7.6 Hz, 1H), 3.06 – 2.90 (m, 2H), 2.84 – 2.74 (m, 2H), 2.69 (dd, *J* = 14.8, 1.9 Hz, 1H), 2.55 (d, *J* = 12.7 Hz, 1H), 2.30 (td, *J* = 11.1, 5.4 Hz, 1H), 2.19 (dt, *J* = 12.8, 6.4 Hz, 1H), 1.89 – 1.62 (m, 5H), 1.59 (s, 3H), 1.46 – 1.29 (m, 2H), 1.26 (s, 3H), 1.24 – 1.14 (m, 2H), 1.12 (s, 3H), 1.10 (s, 3H), 0.94 (d, *J* = 6.6 Hz, 3H), 0.84 (d, *J* = 6.7 Hz, 3H), 0.82 (s, 3H), 0.80 (s, 3H). **^{13}C NMR** (126 MHz, $CDCl_3$) δ 212.1, 176.4, 97.1, 96.5, 96.0, 86.5, 84.5, 82.2, 80.5, 78.6, 63.0, 56.3, 55.9, 55.8, 55.6, 55.3, 50.3, 48.8, 46.8, 42.4, 40.2, 39.7, 39.4, 38.7, 36.1, 33.7, 26.8, 26.4, 26.3, 26.0, 21.1, 20.0, 19.8, 18.9, 17.6, 13.8. **IR**: $\bar{\upsilon}$ 1654, 1450, 1366, 1275, 1228, 1099, 1043, 916, 664 cm^{-1}. **HRMS** (ESI) m/z: anal. calculated for $[C_{36}H_{61}NO_9 + H]^+$: 652.4419, found: 652.4415.

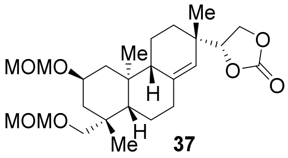

37

To a solution of **36** (1.56 g, 4.4 mol,) in DCM (60 mL) was added MOMCl (1.41 g, 17.6 mmol), DIPEA (2.58 g, 20.0 mmol) and DMAP (12.2 g, 0.10 mmol). The reaction mixture was heated at reflux for 48 h. The mixture was cooled to room temperature, diluted with ethyl acetate (80 mL), and washed with 2N HCl (20 mL × 2), $NaHCO_3$ (20 ml × 2) and brine (20 mL × 2). The organic phase was dried over sodium sulfate and concentrated in vacuum. Flash column chromatography over silica column afforded **37** as a yellow oil. Yield: (1.59 g, 80%). **^{1}H NMR** (500 MHz, $CDCl_3$) δ 5.05 (s, 1H), 4.76 – 4.63 (m, 2H), 4.59 (d, *J* = 13.3 Hz, 3H), 4.39 (t, *J* = 8.5 Hz, 1H), 4.29 (t, *J* = 7.9 Hz, 1H), 3.73 (ddd, *J* = 11.8, 7.8, 4.1 Hz, 1H), 3.55 (d, *J* = 9.4 Hz, 1H), 3.36 (d, *J* = 8.1 Hz, 6H), 3.26 (d, *J* = 9.3 Hz, 1H), 2.26 (t, *J* = 13.0 Hz, 2H), 2.04 (q, *J* = 13.2, 9.2 Hz, 2H), 1.84 (t, *J* = 8.5 Hz, 2H), 1.78 – 1.60 (m, 3H), 1.48 (tdd, *J* = 13.6, 8.9, 3.7 Hz, 1H), 1.25 (dt, *J* = 30.9, 13.0 Hz, 3H), 1.15 – 0.87 (m, 7H), 0.78 (s, 3H). **^{13}C NMR** (126 MHz, $CDCl_3$) δ 155.2, 142.6, 123.5, 96.7, 94.5, 79.7, 71.0, 69.9, 65.6, 55.2, 55.1, 55.0, 50.5, 45.4, 42.3, 39.4, 39.1, 36.5, 36.2, 31.0, 28.2, 22.2, 21.2, 18.1, 16.7. **HRMS** (ESI) m/z: anal. calculated for $C_{25}H_{40}O_7$ $[M + Na]^+$: 475.2666, found: 475.2653.

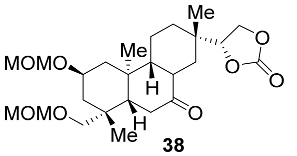

38

To a solution of **37** (1.40 g, 3.0 mol,) in 1,4-dioxane (40 mL) was added SeO_2 (780 mg, 6.0 mmol) under Ar. The reaction mixture was stirred at RT for 24 h. The suspension was filtered through a pad of celite/silica gel and the pad was washed with mixture of CH_2Cl_2/EtOAc. The combined filtrates were concentrated to dryness to afford crude product which was directly used in the next step without further purification.

DMSO (816 mg, 12.0 mmol) was dissolved in DCM (40 mL) and cooled to −78 °C. $(COCl)_2$ (3.0 ml, 2.0 M, 6.0 mmol) was then added dropwise, and the solution was stirred for 15 min, and the above product dissolved in DCM (10 mL) was added. The reaction was kept for 50 min at this temperature. Et_3N (2.02 g, 20 mmol) was added. The reaction was allowed to warm to rt. The mixture was diluted with ethyl acetate (100 mL), and washed with $NaHCO_3$ (30 ml × 2) and brine (50 mL × 2). The organic phase was dried over sodium sulfate and concentrated in vacuum. Flash column chromatography over silica column afford of **38** as white solid. Yield: (839 mg, 60% for two steps). A mixture of compound **38** (178 mg, 0.38 mmol) and Pd/C (36 mg, 10%) in EtOAc (20 mL) was hydrogenated at rt for 24 h under hydrogen atmosphere. The suspension was filtered through a pad of celite and the pad was washed with CH_2Cl_2. The combined filtrates were concentrated to dryness to afford of crude product. Flash column chromatography over silica column afforded **S38** as white solid. Yield: (126 mg. 70%). **^{1}H NMR** (400 MHz, $CDCl_3$) δ 4.86 – 4.76 (m, 1H), 4.76 – 4.64 (m, 2H), 4.63 – 4.53 (m, 2H), 4.39 (t, *J* = 8.6 Hz, 1H), 4.25 (dd, *J* = 8.9, 7.4 Hz, 1H), 3.84 (tt, *J* = 11.6, 4.2 Hz, 1H), 3.50 (d, *J* = 9.5 Hz, 1H), 3.42 (d, *J* = 9.5 Hz, 1H), 3.37 (s, 3H), 3.35 (s, 3H), 2.54 (dd, *J* = 14.4, 3.3 Hz, 1H), 2.43 (t, *J* = 14.3 Hz, 1H), 2.25 – 2.11 (m, 2H), 2.08 – 1.99 (m, 1H), 1.86 – 1.68 (m, 2H), 1.42 (ddd, *J* = 26.4, 13.2, 3.5 Hz, 2H), 1.34 – 1.21 (m, 2H), 1.15 (d, *J* = 3.2 Hz, 4H), 1.13 – 1.00 (m, 5H), 0.96 (d, *J* = 12.0 Hz, 1H), 0.93 (s, 3H). **^{13}C NMR** (101 MHz, $CDCl_3$) δ 210.5, 154.8, 96.7, 94.6, 78.0, 71.2, 69.5, 65.2, 55.3, 55.2, 55.2, 54.3, 44.9, 44.1, 42.9, 39.3, 38.9, 37.9, 34.9, 34.2, 33.44 27.7, 22.5, 20.6, 15.0. **HRMS** (ESI) m/z: anal. calculated for $C_{25}H_{40}O_8$ $[M + Na]^+$: 491.2615, found: 375. 491.2627.

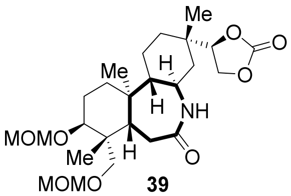

39

39, yield: (44% from **S38**). **^{1}H NMR** (500 MHz, $CDCl_3$) δ 5.30 (s, 1H), 4.78 – 4.69 (m, 1H), 4.66 (q, *J* = 6.8 Hz, 2H), 4.58 (s, 2H), 4.48 (t, *J* = 8.6 Hz, 1H), 4.27 (t, *J* = 8.1 Hz, 1H), 3.71 (tt, *J* = 12.0, 3.9 Hz, 1H), 3.56 – 3.41 (m, 2H), 3.42 – 3.23 (m, 7H), 2.52 – 2.33 (m, 2H), 2.23 (td, *J* = 10.9, 3.6 Hz, 2H), 2.06 – 1.90 (m, 1H), 1.84 (dt, *J* = 14.8, 4.4 Hz, 1H), 1.76 (s, 1H), 1.66 (ddd, *J* = 13.9, 5.0, 2.6 Hz, 1H), 1.53 (dd, *J* = 6.8, 3.5 Hz, 1H), 1.50 – 1.30 (m, 1H), 1.31 – 1.11 (m, 6H), 1.07 (s, 3H), 1.03 (t, *J* = 12.2 Hz, 1H), 0.95 (s, 3H). **^{13}C NMR** (126 MHz, $CDCl_3$) δ 177.8, 154.5, 96.6, 94.6, 78.2, 70.7, 69.3, 65.2, 55.4, 55.3, 55.2, 51.7, 46.8, 46.7, 42.3, 41.9, 40.8, 35.7, 32.7, 32.4, 28.0, 22.4, 19.8, 16.8. **HRMS** (ESI) m/z: anal. calculated for $[C_{25}H_{41}NO_8+H]^+$: 484.2905, found: 484.2898.

Derivatization of medium ring scaffolds.

General procedure for syntheses of carbamates.

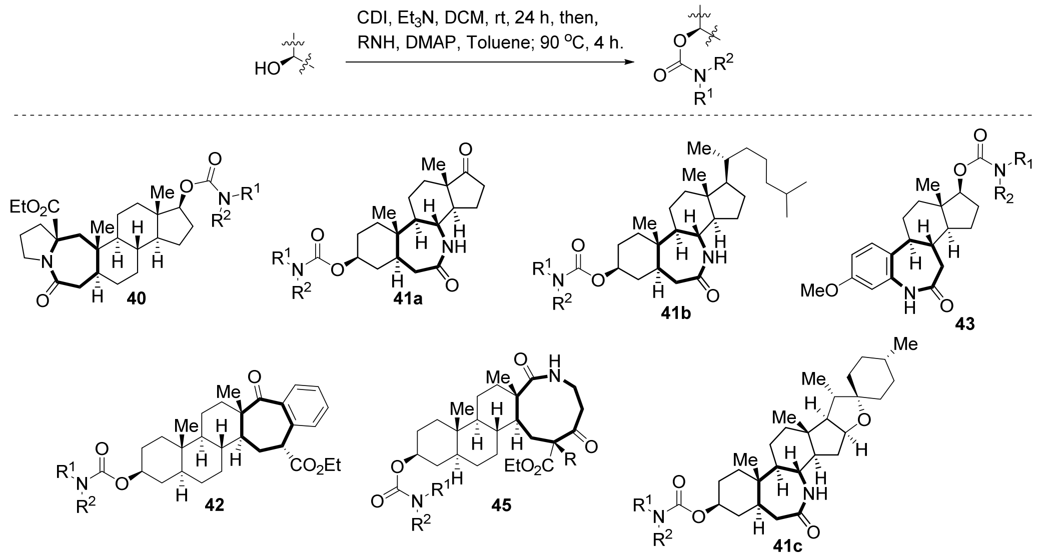

Supplementary Figure 13. Formation of carbamates. General procedure F was used for formation of carbamates **40, 41, 42** and **45**. General procedure G was used for formation of carbamates **43**.

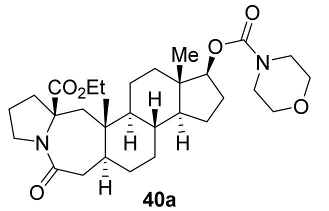

40a

40a, white solid, yield: (15.4 mg, 29%). **^{1}H NMR** ($CDCl_3$, 500 MHz) δ 4.56 (dd, *J* = 9.1, 7.9 Hz,

1H), 4.29-4.23 (m, 1H), 4.18-4.11 (m, 1H), 3.78-3.73 (m, 1H), 3.66 (br, 4H), 3.55-3.50 (m, 1H), 3.46 (br, 4H), 2.77-2.72 (m, 2H), 2.47-2.43 (m, 1H), 2.21-2.13 (m, 1H), 2.07 (d, *J* = 15.2 Hz, 1H), 2.03-1.99 (m, 1H), 1.81-1.76 (m, 2H), 1.69-1.58 (m, 4H), 1.53-1.43 (m, 3H), 1.38-1.15 (m, 9H), 1.06-1.00 (m, 1H), 0.91 (td, *J* = 12.7, 4.4 Hz, 1H), 0.80 (s, 3H), 0.77 (s, 3H), 0.70 (td, *J* = 11.2, 3.7 Hz, 1H). **^{13}C NMR** ($CDCl_3$, 125 MHz) δ 174.9, 173.9, 155.6, 83.8, 68.1, 61.8, 55.0, 50.7, 50.5, 49.0, 44.9, 42.9, 42.5, 40.7, 39.7, 37.1, 34.2, 31.4, 30.3, 28.0, 23.5, 21.0, 20.9, 14.2, 12.4, 12.4. **HRMS** (ESI): *m/z*: calculated for $C_{30}H_{47}N_2O_6$: $[M + H]^+$ 531.3429, found: 531.3430.

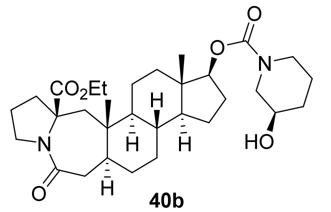

40b

40b, white solid, yield: (25.6 mg, 47%). **^{1}H NMR** ($CDCl_3$, 500 MHz) δ 4.53 (dd, *J* = 8.9, 8.0 Hz, 1H), 4.29-4.22 (m, 1H), 4.17-4.11 (m, 1H), 3.78-3.74 (m, 3H), 3.58-3.48 (m, 2H), 3.18-3.13 (m, 2H), 2.77-2.72 (m, 2H), 2.47-2.43 (m, 1H), 2.19-2.12 (m, 1H), 2.06 (d, *J* = 14.9 Hz, 1H), 2.03-1.98 (m, 1H), 1.90 (m, 1H), 1.80-1.76 (m, 3H), 1.68-1.58 (m, 4H), 1.54-1.43 (m, 5H), 1.37-1.22 (m, 8H), 1.17 (td, *J* = 12.7, 3.6 Hz, 1H), 1.05-0.99 (m, 1H), 0.90 (td, *J* = 12.6, 4.1 Hz, 1H), 0.80 (s, 3H), 0.78 (s, 3H), 0.69 (td, *J* = 11.2, 3.8 Hz, 1H). **^{13}C NMR** ($CDCl_3$, 125 MHz) δ 174.9, 173.9, 155.6, 83.7, 68.0, 61.8, 55.0, 50.8, 50.7, 50.6, 49.0, 44.9, 42.9, 42.5, 40.8, 39.7, 37.1, 34.2, 31.4, 30.3, 28.0, 23.5, 21.0, 20.9, 14.2, 12.4, 12.4. **HRMS** (ESI): *m/z*: calculated for $C_{31}H_{49}N_2O_6$: $[M + H]^+$ 545.3585, found: 545.3584.

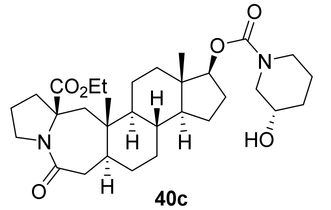

40c

40c, white solid, yield: (30.4 mg, 56%). **^{1}H NMR** ($CDCl_3$, 500 MHz) δ 4.53 (dd, *J* = 9.0, 7.9 Hz, 1H), 4.29-4.22 (m, 1H), 4.17-4.11 (m, 1H), 3.78-3.73 (m, 3H), 3.58-3.48 (m, 2H), 3.20-3.13 (m, 2H), 2.77-2.71 (m, 2H), 2.47-2.42 (m, 1H), 2.19-2.12 (m, 1H), 2.05 (d, *J* = 15.5 Hz, 1H), 2.03-1.98 (m, 1H), 1.90 (m, 1H), 1.80-1.76 (m, 3H), 1.69-1.60 (m, 4H), 1.53-1.42 (m, 5H), 1.37-1.22 (m, 8H), 1.17 (td, *J* = 12.7, 3.7 Hz, 1H), 1.05-0.99 (m, 1H), 0.90 (td, *J* = 12.6, 4.2 Hz, 1H), 0.80 (s, 3H), 0.78 (s, 3H), 0.69 (td, *J* = 11.2, 3.7 Hz, 1H). **^{13}C NMR** ($CDCl_3$, 125 MHz) δ 174.6, 174.0, 156.0, 83.7, 68.0, 61.7, 55.0, 50.8, 50.7, 50.6, 48.9, 44.9, 42.9, 42.5, 40.9, 39.7, 37.1, 34.2, 31.4, 30.4, 28.0, 23.5, 21.0, 20.9, 14.2, 12.4. **HRMS** (ESI): *m/z*: calculated for $C_{31}H_{49}N_2O_6$: $[M + H]^+$ 545.3585, found: 545.3585.

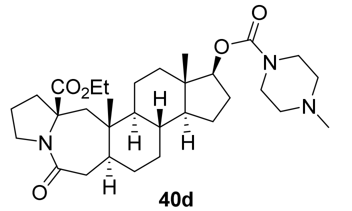

40d

40d, white solid, yield: (19.0 mg, 35%). **^{1}H NMR** ($CDCl_3$, 500 MHz) δ 4.54 (dd, *J* = 8.9, 7.7 Hz, 1H), 4.29-4.22 (m, 1H), 4.17-4.11 (m, 1H), 3.75 (ddd, *J* = 11.3, 8.6, 2.7 Hz, 1H), 3.54-3.51 (m, 4H), 2.77-2.71 (m, 2H), 2.45 (br, 5H), 2.35 (s, 3H), 2.20-2.12 (m, 1H), 2.06-1.98 (m, 2H), 1.80-1.76 (m, 2H), 1.69-1.57 (m, 4H), 1.53-1.43 (m, 3H), 1.38-1.14 (m, 10H), 1.05-0.99 (m, 1H), 0.90 (td, *J* = 12.6, 4.3 Hz, 1H), 0.80 (s, 3H), 0.77 (s, 3H), 0.69 (td, *J* = 11.2, 3.8 Hz, 1H). **^{13}C NMR** ($CDCl_3$, 125 MHz) δ 174.5, 174.0, 155.5, 83.7, 67.9, 61.7, 55.0, 54.5, 50.7, 48.9, 45.8, 45.0, 42.9, 42.5, 40.9, 39.7, 37.1, 34.2, 31.4, 30.4, 28.0, 23.5, 21.0, 20.9, 14.2, 12.4, 12.4. **HRMS** (ESI): *m/z*: calculated for $C_{31}H_{50}N_3O_5$: $[M + H]^+$ 544.3745, found: 544.3739.

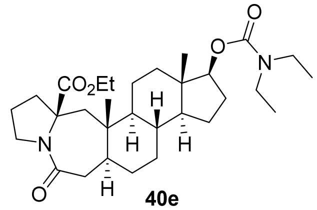

40e

40e, white solid, yield: (10.3 mg, 20%). **^{1}H NMR** ($CDCl_3$, 500 MHz) δ 4.51 (dd, *J* = 9.3, 7.8 Hz, 1H), 4.28-4.22 (m, 1H), 4.17-4.11 (m, 1H), 3.75 (ddd, *J* = 11.6, 8.5, 3.1 Hz, 1H), 3.54-3.49 (m, 1H), 3.27 (br, 4H), 2.78-2.71 (m, 2H), 2.46-2.42 (m, 1H), 2.22-2.14 (m, 1H), 2.05 (d, *J* = 15.3 Hz, 1H), 2.03-1.98 (m, 1H), 1.80-1.76 (m, 2H), 1.69-1.57 (m, 4H), 1.53-1.43 (m, 3H), 1.38-1.24 (m, 8H), 1.18 (td, *J* = 12.5, 4.0 Hz, 1H), 1.12 (t, *J* = 7.2 Hz, 6H), 1.05-0.99 (m, 1H), 0.90 (td, *J* = 13.0, 3.9 Hz, 1H), 0.80 (s, 3H), 0.78 (s, 3H), 0.69 (td, *J* = 11.2, 3.8 Hz, 1H). **^{13}C NMR** ($CDCl_3$, 100 MHz) δ 174.5, 174.0, 156.0, 83.2, 67.9, 61.6, 55.0, 50.6, 48.8, 44.9, 42.9, 42.4, 40.9, 39.7, 37.1, 34.2, 31.4, 30.3, 28.0, 23.5, 20.9, 20.8, 14.1, 12.4, 12.3. **HRMS** (ESI): *m/z*: calculated for $C_{30}H_{49}N_2O_5$: $[M + H]^+$ 517.3636, found: 517.3631.

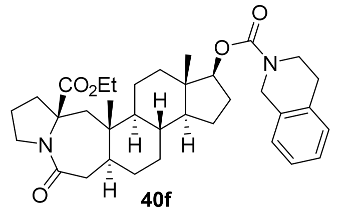

40f

40f, white solid, yield: (25.3 mg, 44%). **^{1}H NMR** ($CDCl_3$, 500 MHz) δ 7.21-7.13 (m, 4H), 4.61 (s, 2H), 4.58 (d, *J* = 8.1 Hz, 1H), 4.29-4.22 (m, 1H), 4.17-4.11 (m, 1H), 3.75 (ddd, *J* = 11.2, 8.1, 3.0 Hz, 1H), 3.69 (br, 2H), 3.55-3.49 (m, 1H), 2.85 (br, 2H), 2.78-2.72 (m, 2H), 2.47-2.42 (m, 1H), 2.23-2.15 (m, 1H), 2.07 (d, *J* = 15.2 Hz, 1H), 2.03-1.98 (m, 1H), 1.80-1.76 (m, 2H), 1.69-1.57 (m, 4H), 1.55-1.43 (m, 3H), 1.38-1.25 (m, 8H), 1.18 (td, *J* = 13.0, 3.5 Hz, 1H), 1.07-1.01 (m, 1H), 0.90 (td, *J* = 12.7, 3.8 Hz, 1H), 0.82 (s, 3H), 0.80 (s, 3H), 0.70 (td, *J* = 11.2, 3.7 Hz, 1H). **^{13}C NMR** ($CDCl_3$, 125 MHz) δ 174.8, 173.9, 155.8, 126.5, 126.3, 68.0, 61.8, 55.0, 50.7, 50.6, 49.0, 45.7, 44.9,

42.9, 42.5, 40.8, 39.7, 37.1, 34.2, 31.4, 30.4, 28.1, 23.5, 21.0, 20.9, 14.2, 12.4, 12.4. **HRMS** (ESI): *m/z*: calculated for $C_{35}H_{49}N_2O_5$: $[M + H]^+$ 577.3636, found: 577.3646.

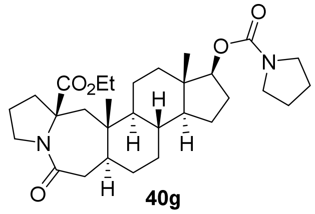

40g

40g, white solid, yield: (37.5 mg, 37%). **^{1}H NMR** ($CDCl_3$, 500 MHz) δ 4.53 (td, *J* = 9.3, 7.9 Hz, 1H), 4.29-4.22 (m, 1H), 4.17-4.11 (m, 1H), 3.75 (ddd, *J* = 11.4, 8.6, 3.1 Hz, 1H), 3.55-3.49 (m, 1H), 3.39-3.43 (m, 4H), 2.78-2.72 (m, 2H), 2.47-2.42 (m, 1H), 2.21-2.13 (m, 1H), 2.06 (d, *J* = 14.7 Hz, 1H), 2.03-1.98 (m, 1H), 1.88-1.83 (m, 4H), 1.79-1.77 (m, 2H), 1.69-1.57 (m, 4H), 1.54-1.43 (m, 3H), 1.37-1.24 (m, 8H), 1.17 (td, *J* = 12.9, 3.6 Hz, 1H), 1.05-0.99 (m, 1H), 0.90 (td, *J* = 12.8, 4.3 Hz, 1H), 0.80 (s, 3H), 0.78 (s, 3H), 0.69 (td, *J* = 11.2, 3.8 Hz, 1H). **^{13}C NMR** ($CDCl_3$, 125 MHz) δ 174.8, 173.9, 155.3, 83.1, 68.0, 61.7, 55.0, 50.7, 50.6, 49.0, 46.1, 45.8, 44.9, 42.9, 42.4, 40.8, 39.7, 37.1, 34.2, 31.4, 30.4, 28.2, 25.8, 25.1, 23.5, 21.0, 20.9, 14.2, 12.4, 12.2. **HRMS** (ESI): *m/z*: calculated for $C_{30}H_{47}N_2O_5$: $[M + H]^+$ 515.3479, found: 515.3485.

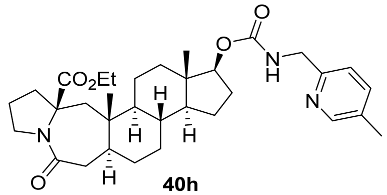

40h

40h, white solid, yield: (30.5 mg, 54%). **^{1}H NMR** ($CDCl_3$, 500 MHz) δ 8.39 (d, *J* = 5.0 Hz, 1H), 7.09 (s, 1H), 7.02 (d, *J* = 5.3 Hz, 1H), 5.73 (t, *J* = 4.7 Hz, 1H), 4.56 (t, *J* = 8.5 Hz, 1H), 4.43 (t, *J* = 4.8 Hz, 2H), 4.29-4.22 (m, 1H), 4.17-4.10 (m, 1H), 3.75 (ddd, *J* = 11.4, 8.5, 2.9 Hz, 1H), 3.54-3.50 (m, 1H), 2.77-2.71 (m, 2H), 2.46-2.42 (m, 1H), 2.35 (s, 3H), 2.19-2.11 (m, 1H), 2.05 (d, *J* = 15.4 Hz, 1H), 2.03-1.98 (m, 1H), 1.86-1.74 (m, 2H), 1.68-1.57 (m, 4H), 1.54-1.42 (m, 3H), 1.37-1.25 (m, 8H), 1.17 (td, *J* = 12.7, 3.2 Hz, 1H), 1.04-0.98 (m, 1H), 0.90 (td, *J* = 12.6, 4.1 Hz, 1H), 0.79 (s, 3H), 0.77 (s, 3H), 0.68 (td, *J* = 11.0, 3.5 Hz, 1H). **^{13}C NMR** ($CDCl_3$, 125 MHz) δ 174.5, 174.0, 156.9, 156.8, 148.9, 148.0, 123.4, 122.7, 83.3, 67.9, 61.7, 55.0, 50.7, 50.7, 48.9, 46.0, 45.0, 42.9, 42.4, 41.0, 39.7, 37.1, 34.2, 31.4, 30.4, 27.8, 23.5, 21.1, 21.0, 20.9, 14.2, 12.4, 12.2. **HRMS** (ESI): *m/z*: calculated for $C_{33}H_{48}N_3O_5$: $[M + H]^+$ 566.3588, found: 566.3584.

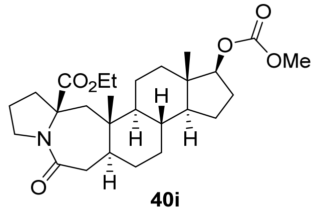

40i

40i, white solid, yield: (34.2 mg, 72%). **^{1}H NMR** ($CDCl_3$, 500 MHz) δ 4.49 (dd, *J* = 8.9, 8.0 Hz, 1H), 4.29-4.22 (m, 1H), 4.17-4.11 (m, 1H), 3.77 (s, 3H), 3.76-3.73 (m, 1H), 3.54-3.48 (m, 1H),

2.78-2.72 (m, 2H), 2.47-2.42 (m, 1H), 2.23-2.16 (m, 1H), 2.05 (d, J = 15.1 Hz, 1H), 2.02-1.97 (m, 1H), 1.87-1.75 (m, 2H), 1.71-1.58 (m, 5H), 1.55-1.43 (m, 2H), 1.39-1.25 (m, 8H), 1.17 (td, J = 12.9, 4.0 Hz, 1H), 1.05-0.99 (m, 1H), 0.90 (td, J = 12.5, 4.2 Hz, 1H), 0.80 (s, 3H), 0.80 (s, 3H), 0.69 (td, J = 11.2, 3.9 Hz, 1H). **^{13}C NMR** ($CDCl_3$, 125 MHz) δ 174.6, 173.9, 155.9, 86.5, 67.9, 61.7, 55.0, 54.6, 50.6, 48.9, 44.9, 42.9, 42.4, 40.9, 39.7, 37.0, 34.1, 31.4, 30.3, 27.5, 23.4, 20.9, 20.9, 14.2, 12.4, 12.1. **HRMS** (ESI): *m/z*: calculated for $C_{27}H_{42}NO_6$: $[M + H]^+$ 476.3007, found: 476.3013.

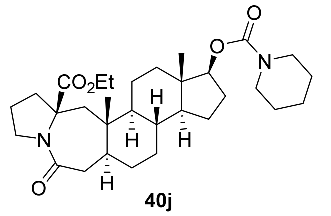

40j

40j, white solid, yield: (20.5 mg, 39%). **^{1}H NMR** ($CDCl_3$, 500 MHz) δ 4.53 (dd, J = 9.3, 8.1 Hz, 1H), 4.28-4.22 (m, 1H), 4.17-4.11 (m, 1H), 3.76 (ddd, J = 11.7, 8.1, 2.7 Hz, 1H), 3.54-3.49 (m, 1H), 3.40 (t, J = 5.3 Hz, 4H), 2.77-2.71 (m, 2H), 2.46-2.42 (m, 1H), 2.20-2.12 (m, 1H), 2.05 (d, J = 15.1 Hz, 1H), 2.03-1.98 (m, 1H), 1.80-1.76 (m, 3H), 1.69-1.58 (m, 5H), 1.52-1.42 (m, 6H), 1.37-1.25 (m, 9H), 1.17 (td, J = 12.9, 3.6 Hz, 1H), 1.05-0.99 (m, 1H), 0.90 (td, J = 12.1, 4.0 Hz, 1H), 0.80 (s, 3H), 0.77 (s, 3H), 0.69 (td, J = 11.3, 4.0 Hz, 1H). **^{13}C NMR** ($CDCl_3$, 125 MHz) δ 174.4, 174.0, 155.6, 83.2, 67.9, 61.6, 55.0, 50.6, 48.8, 44.9, 44.8, 42.9, 42.4, 40.9, 39.7, 37.1, 34.2, 31.4, 30.5, 30.3, 28.0, 24.5, 23.5, 20.9, 20.8, 14.1, 12.4, 12.3. **HRMS** (ESI): *m/z*: calculated for $C_{31}H_{49}N_2O_5$: $[M + H]^+$ 529.3636, found: 529.3640.

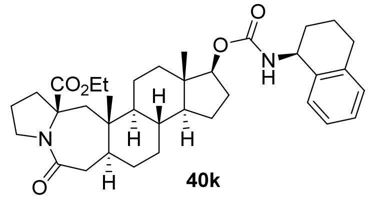

40k

40k, white solid, yield: (15.3 mg, 26%). **^{1}H NMR** ($CDCl_3$, 500 MHz) δ 7.32 (dd, J = 6.0, 4.5 Hz, 1H), 7.17 (dd, J = 5.5, 3.9 Hz, 2H), 7.09 (dd, J = 5.3, 3.5 Hz, 1H), 4.88 (s, 2H), 4.57 (t, J = 8.4 Hz, 1H), 4.28-4.22 (m, 1H), 4.17-4.10 (m, 1H), 3.75 (ddd, J = 11.5, 8.5, 3.0 Hz, 1H), 3.54-3.49 (m, 1H), 2.80-2.71 (m, 4H), 2.47-2.42 (m, 1H), 2.23-2.15 (m, 1H), 2.05 (d, J = 15.0 Hz, 1H), 2.04-1.99 (m, 1H), 1.84-1.77 (m, 5H), 1.69-1.59 (m, 4H), 1.53-1.43 (m, 3H), 1.34-1.20 (m, 10H), 1.06-1.00 (m, 1H), 0.90 (td, J = 12.9, 4.1 Hz, 1H), 0.79 (s, 3H), 0.73 (s, 3H), 0.73-0.67 (m, 1H). **^{13}C NMR** ($CDCl_3$, 125 MHz) δ 174.7, 174.0, 156.3, 137.5, 137.0, 129.2, 128.8, 127.3, 126.3, 83.1, 68.0, 61.7, 55.0, 50.7, 50.6, 49.1, 49.0, 45.0, 42.9, 42.4, 40.9, 39.7, 37.2, 34.2, 31.4, 30.5, 30.4, 29.3, 27.9, 23.5, 21.0, 20.9, 19.9, 14.2, 12.4, 12.2. **HRMS** (ESI): *m/z*: calculated for $C_{36}H_{51}N_2O_5$: $[M + H]^+$ 591.3792, found: 591.3802.

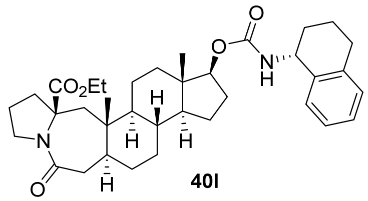

40l

40l, white solid, yield: (15.3 mg, 26%). **^{1}H NMR** ($CDCl_3$, 500 MHz) δ 7.33 (dd, *J* = 5.7, 3.8 Hz, 1H), 7.18 (dd, *J* = 5.7, 3.5 Hz, 2H), 7.09 (dd, *J* = 5.4, 3.6 Hz, 1H), 4.88 (s, 2H), 4.59 (t, *J* = 8.5 Hz, 1H), 4.29-4.22 (m, 1H), 4.17-4.11 (m, 1H), 3.76 (ddd, *J* = 11.3, 8.1, 2.7 Hz, 1H), 3.55-3.49 (m, 1H), 2.82-2.72 (m, 4H), 2.47-2.43 (m, 1H), 2.21-2.13 (m, 1H), 2.05 (d, *J* = 15.2 Hz, 1H), 2.04-1.99 (m, 1H), 1.83-1.76 (m, 5H), 1.71-1.57 (m, 4H), 1.53-1.43 (m, 3H), 1.34-1.22 (m, 10H), 1.06-1.00 (m, 1H), 0.91 (td, *J* = 12.7, 4.0 Hz, 1H), 0.80 (s, 3H), 0.74 (s, 3H), 0.74-0.68 (m, 1H). **^{13}C NMR** ($CDCl_3$, 125 MHz) δ 174.8, 173.9, 156.4, 137.6, 137.1, 129.2, 128.7, 127.3, 126.2, 83.1, 68.0, 61.8, 55.0, 50.7, 50.6, 49.1, 49.0, 44.9, 42.9, 42.5, 40.8, 39.7, 37.2, 34.2, 31.4, 30.5, 30.4, 29.3, 27.8, 23.5, 21.0, 20.9, 19.9, 14.2, 12.4, 12.2. **HRMS** (ESI): *m/z*: calculated for $C_{36}H_{51}N_2O_5$: $[M + H]^+$ 591.3792, found: 591.3778.

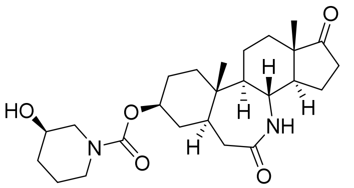

41aa

41aa, white solid, yield: (39.2 mg, 88%). **^{1}H NMR** ($CDCl_3$, 500 MHz) δ 6.60 (s, 1H), 4.59-4.53 (m, 1H), 3.75-3.73 (m, 2H), 3.51 (td, *J* = 10.1, 4.1 Hz, 2H), 3.18-3.12 (m, 2H), 2.72 (dd, *J* = 14.4, 10.3 Hz, 1H), 2.56-2.51 (m, 1H), 2.25-2.16 (m, 2H), 1.95-1.76 (m, 9H), 1.63-1.36 (m, 8H), 1.31-1.23 (m, 2H), 1.16 (td, *J* = 13.6, 2.9 Hz, 1H), 1.05 (s, 3H), 0.88 (s, 3H). **^{13}C NMR** ($CDCl_3$, 125 MHz) δ 218.8, 178.6, 155.4, 73.3, 53.6, 51.7, 50.7, 49.3, 47.5, 41.5, 39.7, 39.0, 37.3, 36.9, 35.7, 30.6, 27.5, 22.4, 21.8, 13.4, 12.8. **HRMS** (ESI): *m/z*: calculated for $C_{25}H_{38}N_2NaO_5$: $[M + Na]^+$ 469.2673, found: 469.2667.

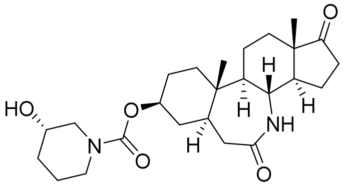

41ab

41ab, white solid, yield: (16.5 mg, 37%). **^{1}H NMR** ($CDCl_3$, 500 MHz) δ 6.20 (s, 1H), 4.59-4.52 (m, 1H), 3.75-3.73 (m, 2H), 3.51 (td, *J* = 10.0, 4.1 Hz, 2H), 3.18-3.12 (m, 2H), 2.71 (dd, *J* = 14.4, 10.3 Hz, 1H), 2.58-2.51 (m, 1H), 2.23-2.15 (m, 2H), 1.95-1.76 (m, 9H), 1.67-1.35 (m, 8H), 1.31-1.22 (m, 2H), 1.16 (td, *J* = 13.8, 3.3 Hz, 1H), 1.04 (s, 3H), 0.88 (s, 3H). **^{13}C NMR** ($CDCl_3$, 125 MHz) δ 218.7, 178.1, 73.3, 53.8, 51.6, 50.7, 49.4, 47.5, 41.5, 39.6, 39.1, 37.3, 37.0, 35.7, 30.7, 27.5, 22.5, 21.8, 13.4, 12.9. **HRMS** (ESI): *m/z*: calculated for $C_{25}H_{38}N_2NaO_5$: $[M + Na]^+$ 469.2673, found: 469.2668.

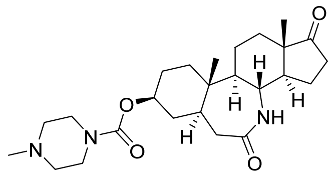

41ac

41ac, white solid, yield: (12.4 mg, 28%). **^{1}H NMR** ($CDCl_3$, 500 MHz) δ 6.50 (s, 1H), 4.61-4.55 (m, 1H), 3.66 (br, 4H), 3.51 (td, *J* = 10.3, 4.1 Hz, 1H), 2.81 (br, 4H), 2.73 (dd, *J* = 14.4, 10.4 Hz, 1H), 2.56 (s, 3H), 2.53-2.52 (m, 1H), 2.25-2.16 (m, 2H), 1.97-1.77 (m, 7H), 1.64-1.48 (m, 4H), 1.43-1.35 (m, 1H), 1.31-1.23 (m, 2H), 1.16 (td, *J* = 13.6, 2.9 Hz, 1H), 1.05 (s, 3H), 0.88 (s, 3H). **^{13}C NMR** ($CDCl_3$, 125 MHz) δ 218.6, 178.4, 154.5, 73.8, 53.6, 53.4, 51.7, 49.3, 47.5, 44.3, 41.4, 39.6, 39.0, 37.2, 36.9, 35.6, 30.6, 27.4, 22.4, 21.8, 13.4, 12.8. **HRMS** (ESI): *m/z*: calculated for $C_{25}H_{40}N_3O_4$: $[M + H]^+$ 446.3013, found: 446.3008.

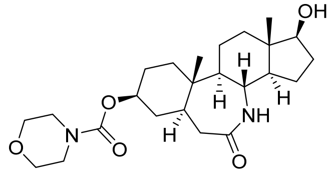

41ad

41ad, white solid, yield: (26.1 mg, 60%). **^{1}H NMR** ($CDCl_3$, 500 MHz) δ 5.65 (s, 1H), 4.60-4.54 (m, 1H), 3.65 (br, 4H), 3.44 (m, 4H), 3.38-3.33 (m, 1H), 2.69 (dd, *J* = 14.4, 10.7 Hz, 1H), 2.14-2.07 (m, 1H), 1.96-1.74 (m, 9H), 1.58-1.34 (m, 6H), 1.24-1.14 (m, 3H), 1.08 (td, *J* = 13.0, 4.1 Hz, 1H), 1.01 (s, 3H), 0.76 (s, 3H). **^{13}C NMR** ($CDCl_3$, 125 MHz) δ 177.7, 155.0, 81.0, 73.5, 53.7, 52.3, 49.1, 42.9, 41.5, 39.5, 39.2, 37.4, 37.0, 35.7, 30.0, 27.5, 24.1, 22.1, 12.9, 10.7. **HRMS** (ESI): *m/z*: calculated for $C_{24}H_{38}N_2NaO_5$: $[M + Na]^+$ 457.2673, found: 457.2668.

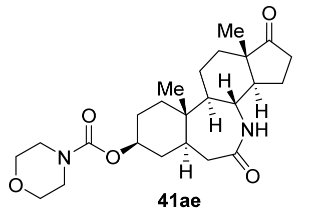

41ae

41ae, Yellow oil. Yield: (27.6 mg, 64%). ^{1}H NMR (500 MHz, $CDCl_3$) δ 5.79 (s, 1H), 4.66 – 4.43 (m, 1H), 3.65 (s, 3H), 3.54 – 3.39 (m, 3H), 2.70 (dd, *J* = 14.4, 10.6 Hz, 1H), 2.54 (dd, *J* = 17.3, 8.7 Hz, 1H), 2.18 (q, *J* = 6.4, 5.7 Hz, 1H), 1.86 (dddd, *J* = 53.6, 31.0, 13.0, 5.5 Hz, 5H), 1.71 – 1.45 (m, 3H), 1.45 – 1.07 (m, 4H), 1.04 (s, 2H), 0.88 (s, 2H). ^{13}C NMR (126 MHz, $CDCl_3$) δ 218.6, 177.3, 154.8, 73.3, 53.9, 51.3, 49.4, 47.4, 41.4, 39.4, 39.2, 37.2, 36.9, 35.6, 30.6, 27.4, 22.4, 21.6, 13.3, 12.8. **HRMS** (ESI) m/z: anal. calculated for $[C_{24}H_{36}N_2O_5 + H]^+$: 433.2697, found: 433.2691.

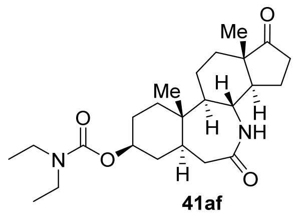

41af

41af, Yellow oil. Yield: (27.1 mg, 65%). **^{1}H NMR** (500 MHz, $CDCl_3$) δ 5.87 (s, 1H), 4.57 (tt, *J* = 10.9, 4.7 Hz, 1H), 3.49 (td, *J* = 10.0, 3.9 Hz, 1H), 3.26 (d, *J* = 14.6 Hz, 2H), 2.70 (dd, *J* = 14.4, 10.5 Hz, 1H), 2.62 – 2.46 (m, 1H), 2.26 – 2.11 (m, 2H), 2.01 – 1.72 (m, 8H), 1.69 – 1.44 (m, 4H), 1.44 – 1.32 (m, 1H), 1.26 (tdd, *J* = 14.1, 11.0, 5.2 Hz, 1H), 1.21 – 1.06 (m, 6H), 1.04 (s, 3H), 0.88 (s, 3H). **^{13}C NMR** (126 MHz, $CDCl_3$) δ 218.7, 177.3, 155.3, 72.5, 53.9, 51.3, 49.3, 47.5, 41.4, 39.5, 39.2, 37.3, 37.0, 35.6, 30.6, 27.5, 22.4, 21.6, 13.3, 12.8. **HRMS** (ESI) m/z: anal. calculated for $[C_{24}H_{38}N_2O_4 + H]^+$: 419.2904, found: 419.2898.

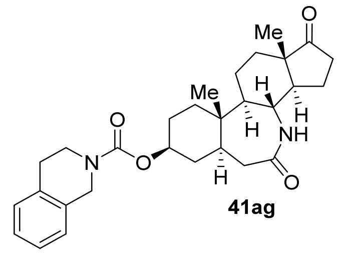

41ag

41ag, Yellow oil. Yield: (36.8 mg, 77%). **^{1}H NMR** (500 MHz, $CDCl_3$) δ 7.24 – 7.02 (m, 4H), 6.42 (s, 1H), 4.60 (t, *J* = 7.4 Hz, 3H), 3.78 – 3.62 (m, 2H), 3.49 (td, *J* = 10.2, 9.8, 3.9 Hz, 1H), 2.84 (s, 2H), 2.71 (dd, *J* = 14.4, 10.6 Hz, 1H), 2.52 (dd, *J* = 19.1, 6.9 Hz, 1H), 2.36 – 2.03 (m, 3H), 2.01 – 1.75 (m, 6H), 1.74 – 1.38 (m, 5H), 1.28 (ddt, *J* = 17.9, 12.2, 5.9 Hz, 2H), 1.23 – 1.11 (m, 1H), 1.05 (s, 3H), 0.88 (s, 3H). **^{13}C NMR** (126 MHz, $CDCl_3$) δ 218.8, 177.5, 154.9, 126.6, 126.5, 126.3, 126.2, 73.1, 53.6, 51.3, 49.0, 47.3, 45.5, 41.3, 39.4, 39.1, 37.1, 36.9, 35.5, 30.4, 27.4, 22.3, 21.6, 13.2, 12.7. **HRMS** (ESI) m/z: anal. calculated for $[C_{29}H_{38}N_2O_4 + H]^+$: 479.2904, found: 479.2896.

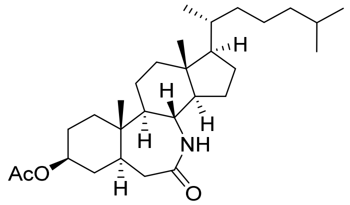

41bb

41bb, yellow oil, yield: (40.4 mg, 88%), **^{1}H NMR** (400 MHz, $CDCl_3$) δ 5.31 (d, *J* = 3.1 Hz, 1H), 4.65 (tt, *J* = 11.5, 4.8 Hz, 1H), 3.31 (td, *J* = 10.0, 3.9 Hz, 1H), 2.68 (dd, *J* = 14.4, 10.3 Hz, 1H), 2.02 (s, 3H), 2.00 – 1.65 (m, 9H), 1.60 – 1.06 (m, 17H), 1.01 (s, 4H), 0.91 (d, *J* = 6.5 Hz, 3H), 0.88 (d, *J* = 2.1 Hz, 3H), 0.86 (d, *J* = 2.1 Hz, 3H), 0.69 (s, 3H). **^{13}C NMR** (101 MHz, $CDCl_3$) δ 177.2, 170.4, 72.4, 55.9, 54.4, 53.5, 52.1, 42.4, 41.3, 39.4, 39.2, 39.2, 38.7, 37.2, 36.4, 35.9, 35.5, 28.0, 27.7, 26.9, 25.1, 23.7, 22.8, 22.5, 22.2, 21.3, 18.5, 12.7, 11.6. **HRMS** (ESI) m/z: anal. calculated for $[C_{29}H_{49}NO_3 + H]^+$: 460.3785, found: 460.3775. LC-MS (t_R= 3.60 min, λ = 210 nm, purity >99%).

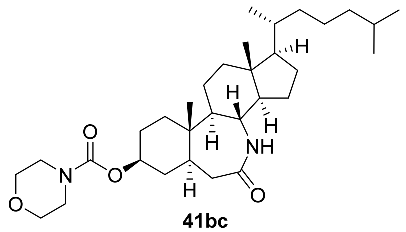

41bc

41bc, white solid, yield: (21.7 mg, 41%). **^{1}H NMR** ($CDCl_3$, 500 MHz) δ 6.56 (s, 1H), 4.61-4.55 (m, 1H), 3.65 (s, 4H), 3.45 (s, 4H), 3.32 (td, *J* = 10.1, 4.0 Hz, 1H), 2.70 (dd, *J* = 14.3, 10.7 Hz, 1H), 1.96-1.89 (m, 4H), 1.86-1.81 (m, 3H), 1.79-1.74 (m, 2H), 1.54-1.42 (m, 3H), 1.39-1.28 (m, 7H), 1.21-1.10 (m, 8H), 1.02 (s, 3H), 0.91 (d, *J* = 6.8 Hz, 3H), 0.87 (d, *J* = 2.8 Hz, 3H), 0.86 (d, *J* = 2.7 Hz, 3H), 0.68 (s, 3H). **^{13}C NMR** ($CDCl_3$, 125 MHz) δ 178.9, 155.0, 73.6, 55.8, 54.1, 52.9, 52.7, 42.5, 41.4, 39.5, 39.4, 38.9, 38.6, 37.2, 36.9, 36.0, 35.6, 28.1, 27.8, 27.5, 25.0, 23.8, 22.9, 22.6, 22.3, 18.7, 12.8, 11.6. **HRMS** (ESI): *m/z*: calculated for $C_{32}H_{55}N_2O_4$: $[M + H]^+$ 531.4156, found: 531.4163.

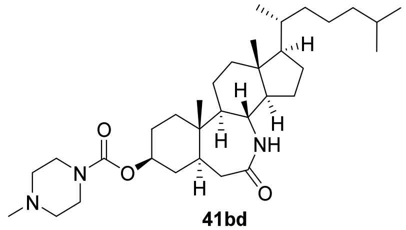

41bd

41bd, white solid, yield: (20.8 mg, 38%). **^{1}H NMR** ($CDCl_3$, 500 MHz) δ 6.18 (s, 1H), 4.60-4.54 (m, 1H), 3.67 (br, 4H), 3.32 (td, *J* = 10.2, 3.8 Hz, 1H), 2.85 (br, 4H), 2.70 (dd, *J* = 14.3, 10.6 Hz, 1H), 2.58 (s, 3H), 1.97-1.87 (m, 4H), 1.82-1.73 (m, 5H), 1.54-1.45 (m, 3H), 1.42-1.25 (m, 7H), 1.21-1.10 (m, 8H), 1.02 (s, 3H), 0.91 (d, *J* = 6.6 Hz, 3H), 0.87 (d, *J* = 2.7 Hz, 3H), 0.86 (d, *J* = 2.6 Hz, 3H), 0.68 (s, 3H). **^{13}C NMR** ($CDCl_3$, 125 MHz) δ 178.4, 154.5, 74.1, 55.9, 54.2, 53.3, 53.1, 52.5, 44.1, 42.5, 41.4, 39.5, 39.4, 39.0, 38.7, 37.2, 36.8, 36.0, 35.6, 28.1, 27.8, 27.4, 25.1, 23.8, 22.9, 22.6, 22.3, 18.7, 12.8, 11.6. **HRMS** (ESI): *m/z*: calculated for $C_{33}H_{58}N_3O_3$: $[M + H]^+$ 544.4473, found: 544.4467.

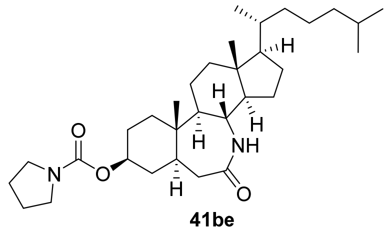

41be

41be, white solid, yield: (46.2 mg, 90%). **^{1}H NMR** ($CDCl_3$, 500 MHz) δ 6.61 (s, 1H), 4.60-4.53 (m, 1H), 3.37-3.30 (m, 5H), 2.70 (dd, *J* = 14.2, 10.8 Hz, 1H), 1.96-1.81 (m, 11H), 1.78-1.74 (m, 2H), 1.55-1.44 (m, 3H), 1.42-1.26 (m, 7H), 1.21-1.10 (m, 8H), 1.02 (s, 3H), 0.91 (d, *J* = 6.4 Hz, 3H), 0.87 (d, *J* = 2.7 Hz, 3H), 0.86 (d, *J* = 2.7 Hz, 3H), 0.68 (s, 3H). **^{13}C NMR** ($CDCl_3$, 125 MHz) δ 179.1, 154.8, 72.8, 55.8, 54.1, 52.9, 52.7, 46.1, 45.8, 42.5, 41.4, 39.5, 39.5, 38.9, 38.6, 37.3, 37.1, 36.0, 35.6, 28.1, 27.8, 27.7, 25.0, 23.8, 22.9, 22.6, 22.3, 18.7, 12.8, 11.6. **HRMS** (ESI): *m/z*: calculated for $C_{32}H_{55}N_2O_3$: $[M + H]^+$ 515.4207, found: 515.4213.

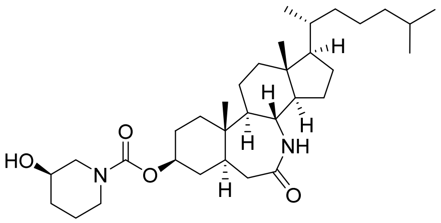

41bf

41bf, white solid, yield: (51.1 mg, 94%). **^{1}H NMR** ($CDCl_3$, 500 MHz) δ 6.40 (s, 1H), 4.58-4.53 (m, 1H), 3.76 (d, *J* = 11.8 Hz, 2H), 3.60-3.53 (m, 1H), 3.32 (td, *J* = 9.9, 3.9 Hz, 1H), 3.15 (br, 2H), 2.70 (dd, *J* = 14.0, 10.5 Hz, 1H), 1.96-1.88 (m, 5H), 1.82-1.73 (m, 6H), 1.54-1.44 (m, 5H), 1.39-1.29 (m, 7H), 1.21-1.10 (m, 8H), 1.02 (s, 3H), 0.91 (d, *J* = 6.7 Hz, 3H), 0.87 (d, *J* = 2.7 Hz, 3H), 0.86 (d, *J* = 2.8 Hz, 3H), 0.68 (s, 3H). **^{13}C NMR** ($CDCl_3$, 125 MHz) δ 178.7, 155.4, 73.5, 55.8, 54.1, 53.0, 52.6, 50.7, 42.5, 41.4, 39.5, 39.4, 38.9, 38.6, 37.2, 36.9, 36.0, 35.6, 29.8, 28.1, 27.8, 27.5, 25.0, 23.8, 22.9, 22.6, 22.3, 18.7, 12.8, 11.6. **HRMS** (ESI): *m/z*: calculated for $C_{33}H_{57}N_2O_4$: $[M + H]^+$ 545.4313, found: 545.4309.

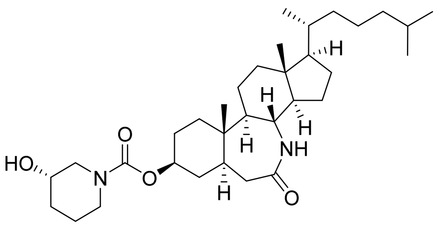

41bg

41bg, white solid, yield: (46.2 mg, 85%). **^{1}H NMR** ($CDCl_3$, 500 MHz) δ 6.30 (s, 1H), 4.57-4.53 (m, 1H), 3.75 (d, *J* = 11.0 Hz, 2H), 3.57 (br, 1H), 3.32 (td, *J* = 10.1, 3.6 Hz, 1H), 3.16-3.10 (m, 2H), 2.70 (dd, *J* = 14.2, 10.7 Hz, 1H), 1.96-1.88 (m, 5H), 1.83-1.73 (m, 6H), 1.54-1.45 (m, 5H), 1.38-1.27 (m, 7H), 1.21-1.10 (m, 9H), 1.01 (s, 3H), 0.91 (d, *J* = 6.5 Hz, 3H), 0.87 (d, *J* = 2.5 Hz, 3H), 0.86 (d, *J* = 2.3 Hz, 3H), 0.68 (s, 3H). **^{13}C NMR** ($CDCl_3$, 125 MHz) δ 178.6, 155.4, 73.5, 55.9, 54.2, 53.0, 52.5, 50.7, 42.5, 41.4, 39.5, 39.4, 39.0, 38.7, 37.3, 36.9, 36.0, 35.6, 29.8, 28.1, 27.8, 27.5, 25.1, 23.8, 22.9, 22.6, 22.3, 18.7, 12.8, 11.6. **HRMS** (ESI): *m/z*: calculated for $C_{33}H_{57}N_2O_4$: $[M + H]^+$ 545.4313, found: 545.4324.

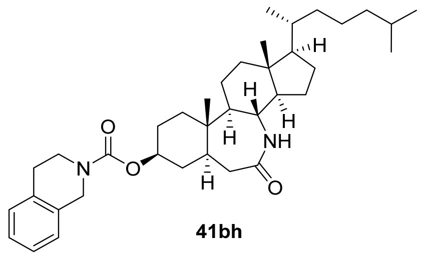

41bh

41bh, white solid, yield: (46.1 mg, 80%). **^{1}H NMR** ($CDCl_3$, 500 MHz) δ 7.20-7.14 (m, 4H), 5.66 (s, 1H), 4.64-4.59 (m, 3H), 3.68-3.65 (m, 2H), 3.32 (td, *J* = 9.8, 3.7 Hz, 1H), 2.84 (br, 2H), 2.69 (dd, *J* = 14.2, 10.6 Hz, 1H), 1.97-1.89 (m, 4H), 1.87-1.74 (m, 5H), 1.58-1.47 (m, 3H), 1.42-1.24 (m, 7H), 1.21-1.10 (m, 8H), 1.02 (s, 3H), 0.91 (d, *J* = 6.3 Hz, 3H), 0.87 (d, *J* = 2.4 Hz, 3H), 0.86 (d, *J* = 2.5 Hz, 3H), 0.69 (s, 3H). **^{13}C NMR** ($CDCl_3$, 125 MHz) δ 177.7, 155.1, 126.5, 126.3, 73.4, 55.9, 54.4,

53.4, 52.3, 45.7, 42.5, 41.4, 39.5, 39.4, 39.2, 38.8, 37.4, 37.0, 36.0, 35.6, 28.1, 27.8, 27.6, 25.1, 23.8, 22.9, 22.6, 22.3, 18.7, 12.9, 11.7. **HRMS** (ESI): *m/z*: calculated for $C_{37}H_{57}N_2O_3$: $[M + H]^+$ 577.4364, found: 577.4364.

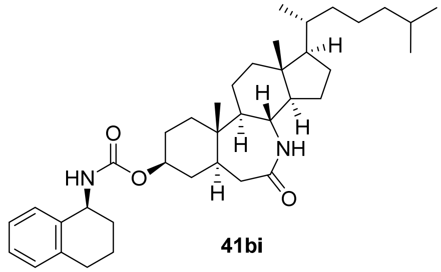

41bi

41bi, white solid, yield: (38.3 mg, 65%). **^{1}H NMR** ($CDCl_3$, 500 MHz) δ 7.32-7.30 (m, 1H), 7.17-7.15 (m, 2H), 7.09-7.07 (m, 1H), 6.62 (s, 1H), 4.87-4.84 (m, 2H), 4.62-4.57 (m, 1H), 3.34-3.30 (m, 1H), 2.82-2.67 (m, 3H), 2.05-2.02 (m, 1H), 1.96-1.92 (m, 4H), 1.83-1.74 (m, 8H), 1.54-1.45 (m, 3H), 1.40-1.27 (m, 7H), 1.22-1.10 (m, 8H), 1.00 (s, 3H), 0.91 (d, J = 6.7 Hz, 3H), 0.87 (d, J = 2.8 Hz, 3H), 0.86 (d, J = 2.7 Hz, 3H), 0.68 (s, 3H). **^{13}C NMR** ($CDCl_3$, 125 MHz) δ 179.0, 155.6, 137.5, 137.0, 129.2, 128.7, 127.3, 126.2, 72.9, 55.8, 54.1, 52.9, 52.7, 49.2, 42.5, 41.4, 39.5, 39.5, 38.9, 38.6, 37.2, 36.8, 36.0, 35.6, 30.5, 29.3, 28.1, 27.8, 27.5, 25.0, 23.8, 22.9, 22.6, 22.3, 20.0, 18.7, 12.8, 11.6. **HRMS** (ESI): *m/z*: calculated for $C_{38}H_{59}N_2O_3$: $[M + H]^+$ 591.4520, found: 591.4512.

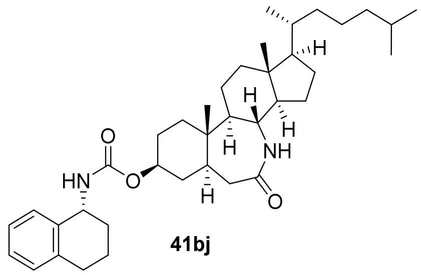

41bj

41bj, white solid, yield: (47.2 mg, 80%). **^{1}H NMR** ($CDCl_3$, 500 MHz) δ 7.34-7.32 (m, 1H), 7.18-7.16 (m, 2H), 7.09-7.07 (m, 1H), 6.25 (s, 1H), 4.88-4.84 (m, 2H), 4.62-4.57 (m, 1H), 3.32 (td, J = 10.0, 3.8 Hz, 1H), 2.82-2.66 (m, 3H), 2.06-2.01 (m, 1H), 1.97-1.92 (m, 4H), 1.84-1.75 (m, 8H), 1.54-1.44 (m, 3H), 1.40-1.26 (m, 7H), 1.21-1.10 (m, 8H), 1.00 (s, 3H), 0.91 (d, J = 6.6 Hz, 3H), 0.87 (d, J = 2.7 Hz, 3H), 0.86 (d, J = 2.8 Hz, 3H), 0.68 (s, 3H). **^{13}C NMR** ($CDCl_3$, 125 MHz) δ 178.5, 155.6, 137.4, 137.0, 129.1, 128.7, 127.3, 126.3, 72.9, 55.9, 54.2, 53.1, 52.5, 49.1, 42.5, 41.4, 39.5, 39.4, 39.0, 38.7, 37.3, 36.9, 36.0, 35.6, 30.6, 29.3, 28.1, 27.8, 27.5, 25.1, 23.8, 22.9, 22.6, 22.3, 20.0, 18.7, 12.8, 11.6. **HRMS** (ESI): *m/z*: calculated for $C_{38}H_{59}N_2O_3$: $[M + H]^+$ 591.4520, found: 591.4528.

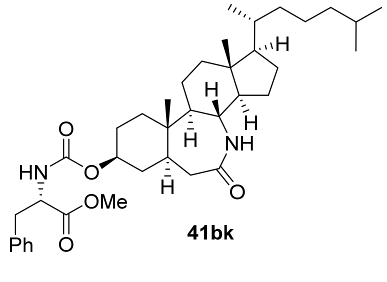

41bk

41bk, white solid, yield: (36.1 mg, 58%). **^{1}H NMR** ($CDCl_3$, 500 MHz) δ 7.31-7.23 (m, 3H), 7.11 (d, *J* = 7.2 Hz, 2H), 5.91 (s, 1H), 5.06 (d, *J* = 8.3 Hz, 1H), 4.63 (q, *J* = 6.6 Hz, 1H), 4.54-4.49 (m, 1H), 3.72 (s, 3H), 3.30 (td, *J* = 10.0, 3.9 Hz, 1H), 3.10 (qd, *J* = 13.9, 5.9 Hz, 2H), 2.68 (dd, *J* = 14.2, 10.5 Hz, 1H), 1.96-1.88 (m, 3H), 1.82-1.70 (m, 6H), 1.54-1.44 (m, 3H), 1.41-1.24 (m, 7H), 1.20-1.06 (m, 8H), 0.98 (s, 3H), 0.91 (d, *J* = 6.3 Hz, 3H), 0.87 (d, *J* = 2.6 Hz, 3H), 0.86 (d, *J* = 2.5 Hz, 3H), 0.68 (s, 3H). **^{13}C NMR** ($CDCl_3$, 125 MHz) δ 178.0, 172.2, 155.3, 135.8, 129.3, 128.7, 127.2, 73.3, 55.9, 54.7, 54.3, 53.2, 52.4, 52.4, 42.5, 41.4, 39.5, 39.3, 39.1, 38.7, 38.3, 37.2, 36.7, 36.0, 35.6, 28.1, 27.8, 27.5, 25.1, 23.8, 22.9, 22.6, 22.3, 18.7, 12.8, 11.6. **HRMS** (ESI): *m/z*: calculated for $C_{38}H_{59}N_2O_5$: $[M + H]^+$ 623.4418, found: 623.4428.

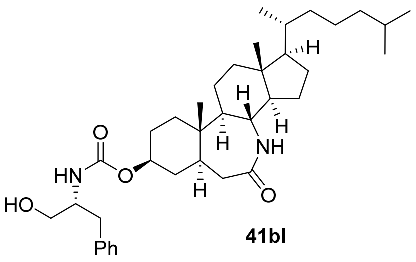

41bl

41bl, white solid, yield: (39.7 mg, 67%). **^{1}H NMR** ($CDCl_3$, 500 MHz) δ 7.31-7.28 (m, 2H), 7.24-7.17 (m, 3H), 6.17 (s, 1H), 4.98 (d, *J* = 6.6 Hz, 1H), 4.52-4.48 (m, 1H), 3.90 (s, 1H), 3.67 (dd, *J* = 11.2, 2.7 Hz, 1H), 3.57 (dd, *J* = 11.1, 4.9 Hz, 1H), 3.31 (td, *J* = 10.2, 3.9 Hz, 1H), 2.86 (d, *J* = 6.1 Hz, 2H), 2.68 (dd, *J* = 14.2, 10.8 Hz, 1H), 1.96-1.88 (m, 3H), 1.83-1.70 (m, 6H), 1.54-1.41 (m, 3H), 1.38-1.25 (m, 7H), 1.22-1.07 (m, 9H), 0.99 (s, 3H), 0.91 (d, *J* = 6.4 Hz, 3H), 0.87 (d, *J* = 2.6 Hz, 3H), 0.86 (d, *J* = 2.5 Hz, 3H), 0.68 (s, 3H). **^{13}C NMR** ($CDCl_3$, 125 MHz) δ 178.5, 156.2, 137.8, 129.4, 128.6, 126.6, 73.1, 64.0, 55.9, 54.2, 53.1, 52.5, 42.5, 41.4, 39.5, 39.4, 39.0, 38.7, 37.2, 36.0, 35.6, 28.1, 27.8, 27.4, 25.1, 23.8, 22.9, 22.6, 22.3, 18.7, 12.8, 11.6. **HRMS** (ESI): *m/z*: calculated for $C_{37}H_{59}N_2O_4$: $[M + H]^+$ 595.4469, found: 595.4475.

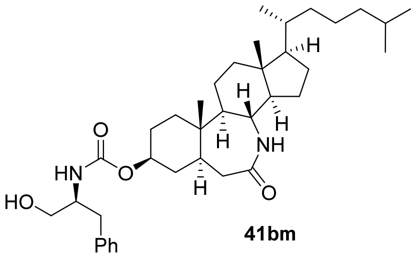

41bm

41bm, white solid, yield: (27.3 mg, 46%). **^{1}H NMR** ($CDCl_3$, 500 MHz) δ 7.32-7.29 (m, 2H), 7.24-7.17 (m, 3H), 6.15 (s, 1H), 4.89 (d, *J* = 7.1 Hz, 1H), 4.53-4.47 (m, 1H), 3.91 (s, 1H), 3.67 (dd, *J* = 11.2, 2.2 Hz, 1H), 3.57 (dd, *J* = 11.1, 5.0 Hz, 1H), 3.31 (td, J = 10.2, 4.1 Hz, 1H), 2.86 (d, *J* = 7.0 Hz, 2H), 2.68 (dd, *J* = 14.3, 10.6 Hz, 1H), 1.96-1.89 (m, 4H), 1.84-1.69 (m, 6H), 1.55-1.41 (m, 3H), 1.38-1.26 (m, 7H), 1.22-1.10 (m, 8H), 0.99 (s, 3H), 0.91 (d, *J* = 6.4 Hz, 3H), 0.87 (d, *J* = 2.8 Hz, 3H), 0.86 (d, *J* = 2.8 Hz, 3H), 0.68 (s, 3H). **^{13}C NMR** ($CDCl_3$, 125 MHz) δ 178.4, 156.2, 137.7, 129.3, 128.7, 126.7, 73.1, 64.2, 55.9, 54.2, 53.1, 52.5, 42.5, 41.4, 39.5, 39.4, 39.0, 38.6, 37.2, 36.8, 36.0, 35.6, 28.1, 27.8, 25.1, 23.8, 22.9, 22.6, 22.3, 18.7, 12.8, 11.6. **HRMS** (ESI): *m/z*: calculated for $C_{37}H_{59}N_2O_4$: $[M + H]^+$ 595.4469, found: 595.4484.

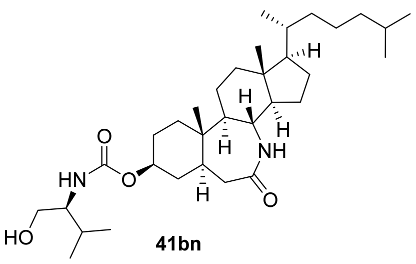

41bn

41bn, white solid, yield: (30.6 mg, 56%). **^{1}H NMR** ($CDCl_3$, 500 MHz) δ 5.95 (s, 1H), 4.80 (d, *J* = 8.4 Hz, 1H), 4.54-4.51 (m, 1H), 3.71 (dd, *J* = 11.0, 2.8 Hz, 1H), 3.63 (dd, *J* = 10.9, 6.5 Hz, 1H), 3.48-3.47 (m, 1H), 3.31 (td, *J* = 10.1, 4.0 Hz, 1H), 2.68 (dd, *J* = 14.4, 10.6 Hz, 1H), 1.97-1.72 (m, 10H), 1.54-1.45 (m, 3H), 1.40-1.26 (m, 7H), 1.20-1.10 (m, 9H), 1.00 (s, 3H), 0.95 (d, *J* = 6.7 Hz, 3H), 0.93 (d, *J* = 6.8 Hz, 3H), 0.91 (d, *J* = 6.4 Hz, 3H), 0.87 (d, *J* = 2.7 Hz, 3H), 0.86 (d, *J* = 2.6 Hz, 3H), 0.68 (s, 3H). **^{13}C NMR** ($CDCl_3$, 125 MHz) δ 178.1, 156.9, 73.1, 64.1, 58.5, 55.9, 54.3, 53.2, 52.4, 42.5, 41.4, 39.5, 39.4, 39.1, 38.7, 37.3, 36.8, 36.0, 35.6, 28.1, 27.8, 27.4, 25.1, 23.8, 22.9, 22.6, 22.3, 19.6, 18.7, 12.8, 11.6. **HRMS** (ESI): *m/z*: calculated for $C_{33}H_{59}N_2O_4$: $[M + H]^+$ 547.4469, found: 547.4474.

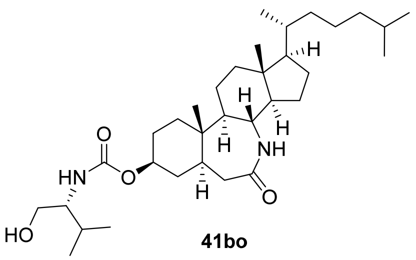

41bo

41bo, white solid, yield: (43.6 mg, 80%). **^{1}H NMR** ($CDCl_3$, 500 MHz) δ 6.58 (s, 1H), 4.83 (d, *J* = 8.5 Hz, 1H), 4.56-4.51 (m, 1H), 3.71 (dd, *J* = 11.0, 3.0 Hz, 1H), 3.64 (dd, *J* = 10.6, 5.9 Hz, 1H), 3.46 (m, 1H), 3.32 (td, *J* = 10.2, 4.2 Hz, 1H), 2.70 (dd, *J* = 14.1, 10.5 Hz, 1H), 1.97-1.71 (m, 10H), 1.54-1.44 (m, 3H), 1.41-1.26 (m, 7H), 1.21-1.10 (m, 9H), 1.01 (s, 3H), 0.96 (d, *J* = 7.0 Hz, 3H), 0.93 (d, *J* = 6.8 Hz, 3H), 0.91 (d, *J* = 6.5 Hz, 3H), 0.87 (d, *J* = 2.8 Hz, 3H), 0.86 (d, *J* = 2.7 Hz, 3H), 0.68 (s, 3H). **^{13}C NMR** ($CDCl_3$, 125 MHz) δ 179.0, 156.9, 73.1, 64.0, 58.5, 55.8, 54.1, 52.9, 52.7, 42.5, 41.4, 39.5, 39.4, 38.8, 38.6, 37.2, 36.7, 36.0, 35.6, 28.1, 27.8, 27.4, 25.0, 23.8, 22.9, 22.6, 22.3, 19.6, 18.7, 12.8, 11.6. **HRMS** (ESI): *m/z*: calculated for $C_{33}H_{59}N_2O_4$: $[M + H]^+$ 547.4469, found: 547.4478.

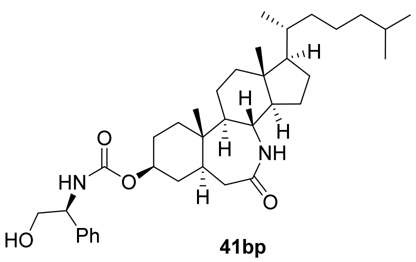

41bp

41bp, white solid, yield: (25.5 mg, 44%). **^{1}H NMR** ($CDCl_3$, 500 MHz) δ 7.37-7.34 (m, 2H), 7.30-7.28 (m, 3H), 5.98 (s, 1H), 5.45 (s, 3H), 4.81 (s, 1H), 4.52 (m, 1H), 3.85 (m, 2H), 3.32-3.28 (m, 1H), 2.67 (dd, *J* = 13.9, 10.8 Hz, 1H), 1.96-1.68 (m, 4H), 1.80-1.68 (m, 5H), 1.54-1.43 (m, 3H), 1.40-

1.23 (m, 7H), 1.18-1.10 (m, 8H), 0.98 (s, 3H), 0.91 (d, *J* = 6.3 Hz, 3H), 0.87 (d, *J* = 2.6 Hz, 3H), 0.86 (d, *J* = 2.4 Hz, 3H), 0.68 (s, 3H). **^{13}C NMR** ($CDCl_3$, 125 MHz) δ 178.2, 156.2, 128.9, 127.9, 126.6, 73.4, 55.9, 54.3, 53.1, 52.4, 42.5, 41.4, 39.5, 39.3, 39.0, 38.7, 37.2, 36.8, 36.0, 35.6, 28.1, 27.8, 25.1, 23.8, 22.9, 22.6, 22.3, 18.7, 12.8, 11.6. **HRMS** (ESI): *m/z*: calculated for $C_{36}H_{57}N_2O_4$: $[M + H]^+$ 581.4313, found: 581.4304.

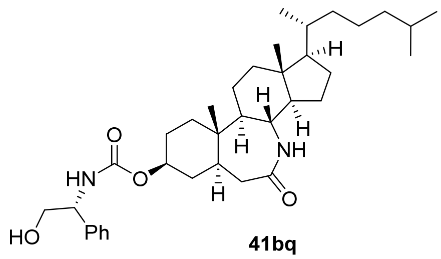

41bq

41bq, white solid, yield: (15.1 mg, 26%). **^{1}H NMR** ($CDCl_3$, 500 MHz) δ 7.37-7.34 (m, 2H), 7.30-7.29 (m, 3H), 6.00 (s, 1H), 5.38 (s, 3H), 4.83 (s, 1H), 4.54 (m, 1H), 3.87 (m, 2H), 3.32-3.29 (m, 1H), 2.70-2.66 (m, 1H), 1.96-1.72 (m, 9H), 1.55-1.43 (m, 3H), 1.40-1.27 (m, 6H), 1.20-1.10 (m, 8H), 0.99 (s, 3H), 0.91 (d, *J* = 6.3 Hz, 3H), 0.87 (d, *J* = 2.6 Hz, 3H), 0.86 (d, *J* = 2.6 Hz, 3H), 0.68 (s, 3H). **^{13}C NMR** ($CDCl_3$, 125 MHz) δ 178.2, 156.1, 128.9, 127.9, 126.6, 73.3, 55.9, 54.3, 53.2, 52.4, 42.5, 41.4, 39.5, 39.3, 39.0, 38.7, 37.2, 36.8, 36.0, 35.6, 28.1, 27.8, 27.4, 25.1, 23.8, 22.9, 22.6, 22.3, 18.7, 12.8, 11.6. **HRMS** (ESI): *m/z*: calculated for $C_{36}H_{57}N_2O_4$: $[M + H]^+$ 581.4313, found: 581.4303.

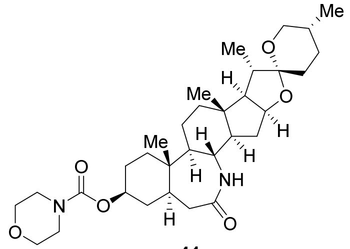

41ca

41ca, yield: (45.2 mg, 81%). **^{1}H NMR** (500 MHz, $CDCl_3$) δ 5.96 – 5.67 (m, 1H), 4.53 (dt, *J* = 11.5, 6.4 Hz, 1H), 4.36 (d, *J* = 6.8 Hz, 1H), 3.76 – 3.53 (m, 4H), 3.53 – 3.12 (m, 6H), 2.67 (dd, *J* = 14.3, 10.5 Hz, 1H), 2.13 (t, *J* = 7.4 Hz, 1H), 1.97 – 1.69 (m, 8H), 1.69 – 1.52 (m, 5H), 1.52 – 1.25 (m, 6H), 1.23 – 1.04 (m, 3H), 1.00 (s, 3H), 0.93 (d, *J* = 5.9 Hz, 3H), 0.76 (d, *J* = 6.4 Hz, 3H), 0.74 (s, 3H). **^{13}C NMR** (126 MHz, $CDCl_3$) δ 177.0, 154.7, 109.1, 79.5, 73.4, 66.8, 66.5, 61.7, 53.8, 53.2, 51.0, 43.9, 41.6, 41.3, 38.0, 39.3, 39.2, 38.6, 37.0, 36.8, 32.3, 31.2, 30.1, 28.6, 27.3, 22.0, 17.0, 15.8, 14.3, 12.6. **HRMS** (ESI) m/z: anal. calculated for $[C_{32}H_{50}N_2O_6 + Na]^+$: 581.3561, found: 581.3553. LC-MS (t_R= 3.21 min, λ = 210 nm, purity 91%).

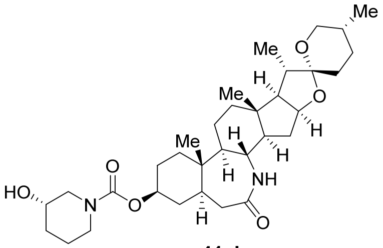

41cb

41cb, yield: (44.0 mg, 77%). **^{1}H NMR** (400 MHz, $CDCl_3$) δ 5.61 – 5.37 (m, 1H), 4.53 (ddd, *J* = 11.4, 8.8, 4.6 Hz, 1H), 4.38 (q, *J* = 7.0 Hz, 1H), 3.82 – 3.63 (m, 2H), 3.60 (d, *J* = 29.3 Hz, 1H), 3.45 (dt, *J* = 13.8, 6.9 Hz, 2H), 3.35 (t, *J* = 10.9 Hz, 1H), 3.09 (dt, *J* = 26.8, 9.6 Hz, 2H), 2.68 (dd, *J* = 14.3, 10.5 Hz, 1H), 2.14 (ddd, *J* = 11.3, 7.4, 4.3 Hz, 1H), 1.95 – 1.05 (m, 30H), 1.02 (s, 3H), 0.78 (d, *J* = 6.2 Hz, 3H), 0.76 (s, 3H). **^{13}C NMR** (101 MHz, $CDCl_3$) δ 177.1, 155.2, 109.2, 79.5, 73.2, 66.8, 66.0, 61.8, 43.9, 41.7, 41.4, 40.0, 39.3, 39.2, 38.7, 37.1, 36.9, 32.4, 31.2, 30.2, 28.7, 27.4, 22.3, 22.1, 17.0, 15.9, 14.4, 12.6. **HRMS** (ESI) m/z: anal. calculated for $[C_{33}H_{52}N_2O_6 + H]^+$: 573.3898, found: 573.3889. LC-MS (t_R= 3.09 min, λ = 210 nm, purity 91%).

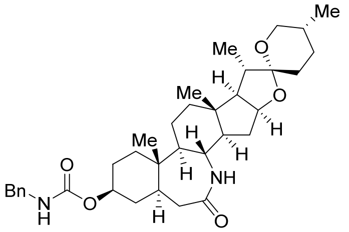

41cc

41cc, yield: (48.5 mg, 84%). **^{1}H NMR** (400 MHz, $CDCl_3$) δ 7.47 – 7.16 (m, 5H), 5.75 – 5.45 (m, 1H), 5.01 (s, 1H), 4.57 (tt, *J* = 11.4, 4.7 Hz, 1H), 4.37 (dd, *J* = 17.5, 6.2 Hz, 3H), 3.57 – 3.19 (m, 3H), 2.68 (dd, *J* = 14.3, 10.5 Hz, 1H), 2.21 – 2.11 (m, 1H), 1.99 – 1.55 (m, 13H), 1.55 – 1.25 (m, 6H), 1.25 – 1.05 (m, 3H), 1.01 (s, 3H), 0.97 (d, *J* = 6.5 Hz, 3H), 0.79 (d, *J* = 6.4 Hz, 3H), 0.77 (s, 3H). **^{13}C NMR** (101 MHz, $CDCl_3$) δ 177.0, 156.0, 138.6, 128.6, 127.5, 127.4, 109.2, 79.5, 72.9, 66.8, 61.8, 54.0, 53.3, 51.0, 44.9, 41.7, 41.4, 40.0, 39.3, 39.2, 38.7, 37.1, 36.8, 32.4, 31.2, 30.2, 28.7, 27.3, 22.0, 17.0, 15.9, 14.4, 12.6. **HRMS** (ESI) m/z: anal. calculated for $[C_{35}H_{50}N_2O_5 + H]^+$: 579.3792, found: 579.3780. LC-MS (t_R= 3.15 min, λ = 210 nm, purity >99%).

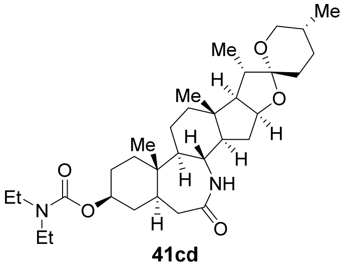

41cd

41cd, yield: (22.8 mg, 42%). **^{1}H NMR** (400 MHz, $CDCl_3$) δ 5.30 – 5.15 (m, 1H), 4.56 (tt, *J* = 11.5, 4.5 Hz, 1H), 4.40 (q, *J* = 7.7 Hz, 1H), 3.48 (dd, *J* = 10.5, 4.4 Hz, 2H), 3.37 (t, *J* = 10.9 Hz, 1H), 3.26 (s, 3H), 2.70 (dd, *J* = 14.4, 10.5 Hz, 1H), 2.15 (ddt, *J* = 12.2, 7.5, 3.6 Hz, 1H), 1.98 – 1.06 (m, 29H), 1.04 (s, 3H), 0.98 (d, *J* = 6.6 Hz, 3H), 0.80 (d, *J* = 6.4 Hz, 3H), 0.78 (s, 3H). **^{13}C NMR** (101 MHz, $CDCl_3$) δ 177.0, 155.4, 109.2, 79.6, 72.7, 66.9, 61.9, 54.2, 53.5, 51.1, 41.7, 41.5, 40.1, 39.4, 39.3,

38.8, 37.2, 37.1, 32.5, 31.3, 30.2, 28.7, 27.6, 22.1, 17.1, 16.0, 14.4, 12.7. **HRMS** (ESI) m/z: anal. calculated for $[C_{32}H_{52}N_2O_5 + H]^+$: 545.3949, found: 545.3946.

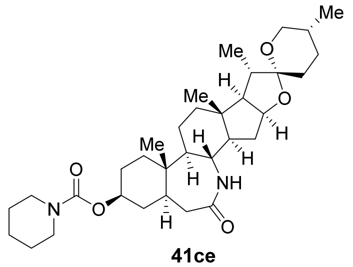

41ce

41ce, yield: (37.8 mg, 68%). **^{1}H NMR** (400 MHz, $CDCl_3$) δ 5.50 (d, J = 2.1 Hz, 1H), 4.53 (tt, J = 11.5, 4.6 Hz, 1H), 4.38 (q, J = 7.6 Hz, 1H), 3.64 – 3.20 (m, 7H), 2.68 (dd, J = 14.4, 10.5 Hz, 1H), 2.19 – 2.09 (m, 1H), 1.95 – 1.26 (m, 25H), 1.15 (dtd, J = 13.4, 8.2, 5.0 Hz, 3H), 1.02 (s, 3H), 0.96 (d, J = 6.6 Hz, 3H), 0.78 (d, J = 6.2 Hz, 3H), 0.76 (s, 3H). **^{13}C NMR** (101 MHz, $CDCl_3$) δ 177.1, 155.0, 109.2, 79.6, 72.9, 66.9, 61.9, 54.1, 53.4, 51.1, 44.7, 41.7, 41.5, 40.1, 39.4, 39.3, 38.8, 37.2, 37.0, 32.5, 31.3, 30.2, 28.7, 27.5, 25.7, 24.4, 22.1, 17.1, 16.0, 14.4, 12.7. **HRMS** (ESI) m/z: anal. calculated for $[C_{33}H_{52}N_2O_5 + Na]^+$: 579.3768, found: 579.3774. LC-MS (t_R= 3.42 min, λ = 210 nm, purity 92%).

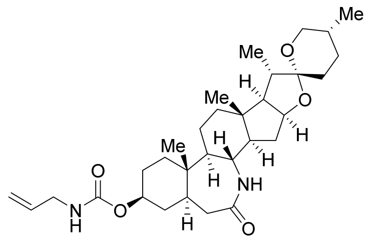

41cf

41cf, yield: (45.9 mg, 87%). **^{1}H NMR** (400 MHz, $CDCl_3$) δ 5.83 (ddt, J = 16.0, 10.8, 5.6 Hz, 1H), 5.56 (d, J = 12.3 Hz, 1H), 5.26 – 5.02 (m, 2H), 4.72 (s, 1H), 4.54 (dq, J = 11.5, 6.7, 5.8 Hz, 1H), 4.38 (q, J = 7.5 Hz, 1H), 3.78 (t, J = 6.2 Hz, 2H), 3.45 (tt, J = 8.6, 4.1 Hz, 2H), 3.35 (t, J = 10.9 Hz, 1H), 2.68 (dd, J = 14.4, 10.5 Hz, 1H), 2.14 (ddd, J = 11.2, 7.4, 4.3 Hz, 1H), 1.96 – 1.54 (m, 13H), 1.54 – 1.27 (m, 5H), 1.27 – 1.04 (m, 4H), 1.01 (s, 3H), 0.96 (d, J = 6.6 Hz, 3H), 0.78 (d, J = 6.2 Hz, 3H), 0.76 (s, 3H). **^{13}C NMR** (101 MHz, $CDCl_3$) δ 177.1, 155.8, 134.6, 115.9, 109.2, 79.5, 72.8, 66.8, 61.8, 54.0, 53.3, 51.0, 43.3, 41.7, 41.4, 40.0, 39.3, 39.2, 38.7, 37.1, 36.8, 32.4, 31.2, 30.2, 28.7, 27.3, 22.0, 17.0, 15.9, 14.4, 12.6. **HRMS** (ESI) m/z: anal. calculated for $[C_{31}H_{48}N_2O_5 + Na]^+$: 551.3455, found: 551.3450. LC-MS (t_R= 3.42 min, λ = 210 nm, purity 94%).

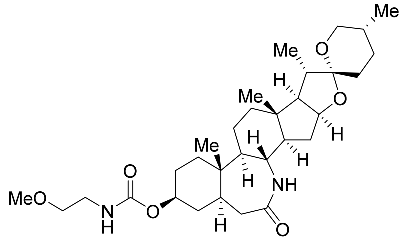

41cg

41cg, yield: (44.7 mg, 82%). **^{1}H NMR** (400 MHz, $CDCl_3$) δ 5.64 (s, 1H), 4.99 (t, J = 5.7 Hz, 1H),

4.51 (tt, *J* = 11.4, 5.1 Hz, 1H), 4.37 (q, *J* = 7.6 Hz, 1H), 3.43 (q, *J* = 6.5, 5.1 Hz, 4H), 3.34 (d, *J* = 5.4 Hz, 6H), 2.67 (dd, *J* = 14.3, 10.5 Hz, 1H), 2.27 – 2.06 (m, 1H), 1.95 – 1.51 (m, 13H), 1.53 – 1.24 (m, 6H), 1.25 – 1.02 (m, 3H), 1.00 (s, 3H), 0.95 (d, *J* = 6.6 Hz, 3H), 0.77 (d, *J* = 6.4 Hz, 3H), 0.75 (s, 3H). **^{13}C NMR** (101 MHz, CDCl$_3$) δ 177.1, 155.9, 109.2, 79.5, 72.6, 71.4, 66.8, 61.8, 58.7, 54.0, 53.3, 51.0, 41.6, 41.4, 40.56, 40.0, 39.3, 39.2, 38.6, 37.1, 36.8, 32.4, 31.2, 30.2, 28.7, 27.3, 22.0, 17.0, 15.9, 14.4, 12.6. **HRMS** (ESI) m/z: anal. calculated for [$C_{31}H_{50}N_2O_6$ + Na]$^+$: 569.3561, found: 569.3545.

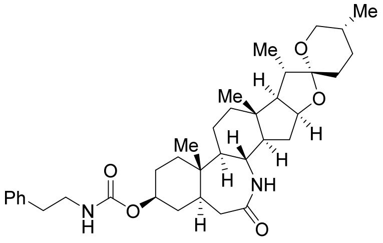

41ch

41ch, yield: (52.6 mg, 89%). **^{1}H NMR** (400 MHz, CDCl$_3$) δ 7.62 – 6.97 (m, 5H), 5.68 (t, *J* = 5.4 Hz, 1H), 4.67 (t, *J* = 6.1 Hz, 1H), 4.52 (tt, *J* = 10.9, 4.6 Hz, 1H), 4.39 (q, *J* = 7.0 Hz, 1H), 3.41 (ddd, *J* = 30.6, 16.6, 8.5 Hz, 5H), 2.80 (t, *J* = 7.1 Hz, 2H), 2.68 (dd, *J* = 14.3, 10.3 Hz, 1H), 2.16 (ddd, *J* = 10.8, 7.1, 3.7 Hz, 1H), 2.06 – 1.54 (m, 14H), 1.48 – 1.25 (m, 7H), 1.24 – 1.05 (m, 3H), 1.00 (s, 3H), 0.97 (d, *J* = 6.1 Hz, 3H), 0.79 (d, *J* = 6.2 Hz, 3H), 0.77 (s, 3H). **^{13}C NMR** (101 MHz, CDCl$_3$) δ 177.1, 155.8, 138.8, 128.7, 128.5, 126.4, 109.2, 79.5, 72.6, 66.8, 61.8, 53.9, 53.3, 51.0, 41.9, 41.6, 41.4, 40.0, 39.3, 39.2, 38.6, 37.1, 36.8, 36.0, 32.4, 31.2, 30.2, 28.7, 27.3, 22.0, 17.0, 15.9, 14.4, 12.6. **HRMS** (ESI) m/z: anal. calculated for [$C_{36}H_{52}N_2O_5$ + H]$^+$: 593.3949, found: 593.3936. LC-MS (t_R= 3.35 min, λ = 210 nm, purity >99%).

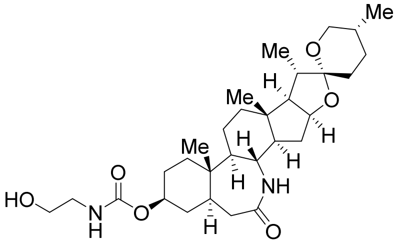

41ci

41ci, yield: (42.6 mg, 79%). **^{1}H NMR** (500 MHz, CDCl$_3$) δ 5.50 (t, *J* = 6.6 Hz, 1H), 5.29 (d, *J* = 7.6 Hz, 1H), 4.52 (hept, *J* = 5.2 Hz, 1H), 4.39 (q, *J* = 7.6 Hz, 1H), 3.69 (t, *J* = 5.2 Hz, 2H), 3.53 – 3.40 (m, 2H), 3.40 – 3.17 (m, 3H), 2.81 (s, 1H), 2.69 (dd, *J* = 14.3, 10.6 Hz, 1H), 2.14 (ddd, *J* = 12.0, 7.3, 4.6 Hz, 1H), 1.97 – 1.24 (m, 16H), 1.23 – 1.04 (m, 3H), 1.01 (s, 3H), 0.96 (d, *J* = 6.5 Hz, 3H), 0.79 (d, *J* = 6.3 Hz, 3H), 0.77 (s, 3H). **^{13}C NMR** (126 MHz, CDCl$_3$) δ 177.2, 156.7, 109.2, 79.5, 72.9, 66.9, 62.2, 61.8, 54.0, 53.3, 51.1, 43.4, 41.7, 41.4, 40.1, 39.3, 39.1, 38.7, 37.1, 36.9, 32.4, 31.2, 30.2, 28.7, 27.3, 22.1, 17.1, 15.9, 14.4, 12.6. **HRMS** (ESI) m/z: anal. calculated for [$C_{30}H_{48}N_2O_6$ + H]$^+$: 533.3585, found: 533.3590. LC-MS (t_R= 2.98 min, λ = 210 nm, purity >99%).

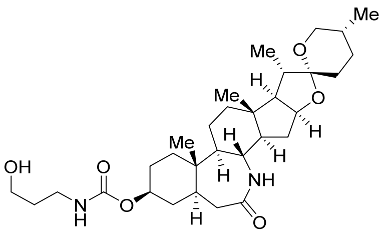

41cj

41cj, yield: (43.7 mg, 80%). **^{1}H NMR** (500 MHz, $CDCl_3$) δ 5.50 (s, 1H), 5.08 (q, *J* = 8.4, 6.3 Hz, 1H), 4.52 (hept, *J* = 4.7 Hz, 1H), 4.39 (q, *J* = 7.3 Hz, 1H), 3.66 (t, *J* = 5.9 Hz, 2H), 3.55 – 3.12 (m, 4H), 2.92 (s, 1H), 2.69 (dd, *J* = 14.5, 10.4 Hz, 1H), 2.14 (ddd, *J* = 11.8, 7.4, 4.7 Hz, 1H), 2.03 – 1.54 (m, 10H), 1.54 – 1.26 (m, 4H), 1.26 – 1.05 (m, 3H), 1.01 (s, 3H), 0.96 (d, *J* = 6.5 Hz, 3H), 0.78 (d, *J* = 6.3 Hz, 3H), 0.76 (s, 3H). **^{13}C NMR** (126 MHz, $CDCl_3$) δ 177.1, 156.9, 109.2, 79.5, 72.9, 66.9, 61.8, 59.5, 54.0, 53.3, 51.1, 41.7, 41.4, 40.1, 39.3, 39.2, 38.7, 37.5, 37.1, 36.8, 32.6, 32.4, 31.2, 30.2, 28.7, 27.3, 22.0, 17.1, 15.9, 14.4, 12.6. **HRMS** (ESI) m/z: anal. calculated for $[C_{31}H_{50}N_2O_6 + H]^+$: 547.3742, found: 547.3740. LC-MS (t_R= 3.01 min, λ = 210 nm, purity >99%).

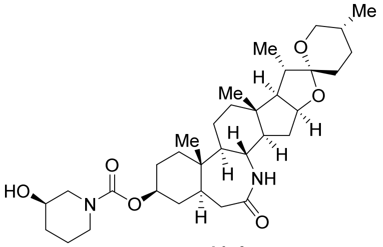

41ck

41ck, yield: (48.0 mg, 84%). **^{1}H NMR** (400 MHz, $CDCl_3$) δ 5.44 (s, 1H), 4.54 (tt, *J* = 11.5, 4.7 Hz, 1H), 4.39 (q, *J* = 7.7 Hz, 1H), 3.91 – 3.41 (m, 5H), 3.36 (t, *J* = 10.9 Hz, 1H), 3.25 – 2.94 (m, 2H), 2.69 (dd, *J* = 14.4, 10.5 Hz, 1H), 2.14 (td, *J* = 7.3, 3.9 Hz, 2H), 1.99 – 1.07 (m, 28H), 1.02 (s, 3H), 0.96 (d, *J* = 6.6 Hz, 3H), 0.79 (d, *J* = 6.4 Hz, 3H), 0.77 (s, 3H). **^{13}C NMR** (101 MHz, $CDCl_3$) δ 177.1, 155.3, 109.2, 79.5, 73.3, 66.9, 66.0, 61.8, 54.1, 53.3, 51.1, 50.6, 43.9, 41.7, 41.4, 40.1, 39.4, 39.2, 38.7, 37.1, 36.9, 32.4, 31.2, 30.2, 28.7, 27.4, 22.3, 22.1, 17.1, 15.9, 14.4, 12.6. **HRMS** (ESI) m/z: anal. calculated for $[C_{33}H_{52}N_2O_6 + Na]^+$: 595.3718, found: 595.3718. LC-MS (t_R= 3.03 min, λ = 210 nm, purity 87%).

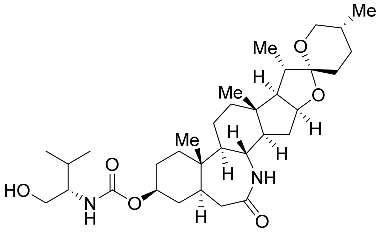

41cl

41cl, yield: (47.7 mg, 82%). **^{1}H NMR** (500 MHz, $CDCl_3$) δ 5.44 (dt, *J* = 6.3, 3.2 Hz, 1H), 4.87 – 4.65 (m, 1H), 4.52 (p, *J* = 6.2 Hz, 1H), 4.39 (q, *J* = 7.8 Hz, 1H), 3.83 – 3.54 (m, 2H), 3.57 – 3.40 (m, 2H), 3.36 (t, *J* = 11.0 Hz, 1H), 2.69 (dd, *J* = 14.3, 10.6 Hz, 1H), 2.40 (s, 1H), 2.15 (ddd, *J* = 12.1, 7.6, 4.9 Hz, 1H), 1.97 – 1.54 (m, 10H), 1.54 – 1.28 (m, 5H), 1.23 – 1.05 (m, 3H), 1.02 (s, 3H), 0.97 – 0.92 (m, 9H), 0.79 (d, *J* = 6.5 Hz, 3H), 0.77 (s, 3H). **^{13}C NMR** (126 MHz, $CDCl_3$) δ 177.1, 156.7,

109.2, 79.6, 72.9, 66.9, 64.0, 61.9, 58.4, 54.1, 53.4, 51.1, 41.7, 41.4, 40.1, 39.3, 39.2, 38.7, 37.1, 36.8, 32.4, 31.3, 30.2, 29.3, 28.7, 27.3, 22.1, 19.5, 18.5, 17.1, 15.9, 14.4, 12.6. **HRMS** (ESI) m/z: anal. calculated for $[C_{33}H_{54}N_2O_6 + Na]^+$: 597.3874, found: 597.3865. LC-MS (t_R= 3.04 min, λ = 210 nm, purity 87%).

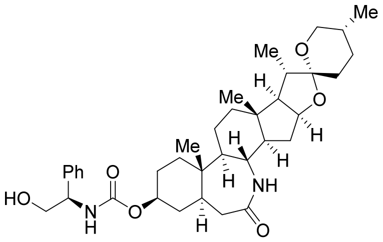

41cm

41cm, yield: (38.9 mg, 64%). **^{1}H NMR** (400 MHz, $CDCl_3$) δ 7.65 – 7.07 (m, 5H), 6.61 (s, 1H), 5.53 (s, 1H), 4.81 (s, 1H), 4.52 (s, 1H), 4.38 (q, *J* = 7.1 Hz, 1H), 4.01 – 3.64 (m, 2H), 3.54 – 3.20 (m, 3H), 2.71 (dd, *J* = 30.8, 17.2 Hz, 2H), 2.14 (ddd, *J* = 11.6, 7.5, 4.6 Hz, 1H), 2.01 – 1.53 (m, 14H), 1.54 – 1.22 (m, 7H), 1.20 – 1.06 (m, 4H), 1.00 (s, 3H), 0.96 (d, *J* = 6.1 Hz, 3H), 0.79 (d, *J* = 6.2 Hz, 3H), 0.76 (s, 3H). **^{13}C NMR** (101 MHz, $CDCl_3$) δ 177.2, 156.0, 139.5, 128.6, 127.6, 126.6, 109.2, 79.5, 73.0, 66.9, 66.4, 61.8, 57.0, 54.0, 53.2, 51.1, 41.7, 41.4, 40.0, 39.3, 39.1, 38.7, 37.0, 32.4, 31.2, 30.2, 28.7, 27.3, 22.0, 17.1, 15.9, 14.4, 12.6. **HRMS** (ESI) m/z: anal. calculated for $[C_{36}H_{52}N_2O_6 + Na]^+$: 631.3718, found: 631.3717. LC-MS (t_R= 3.37 min, λ = 254 nm, purity >99%).

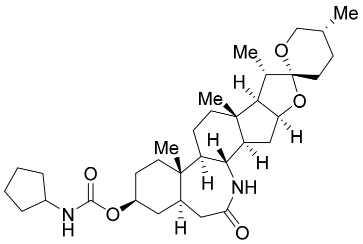

41cn

41cn, yield: (35.5 mg, 64%). **^{1}H NMR** (400 MHz, $CDCl_3$) δ 5.50 (d, *J* = 14.5 Hz, 1H), 4.76 – 4.45 (m, 2H), 4.39 (q, *J* = 7.6 Hz, 1H), 3.95 (dt, *J* = 7.9, 4.8 Hz, 1H), 3.56 – 3.41 (m, 2H), 3.37 (q, *J* = 11.4, 10.9 Hz, 1H), 2.68 (dd, *J* = 14.4, 10.5 Hz, 1H), 2.14 (ddd, *J* = 11.5, 7.5, 4.3 Hz, 1H), 2.01 – 1.27 (m, 29H), 1.27 – 1.05 (m, 3H), 1.01 (s, 3H), 0.96 (d, *J* = 6.5 Hz, 3H), 0.79 (d, *J* = 6.2 Hz, 3H), 0.76 (s, 3H). **^{13}C NMR** (101 MHz, $CDCl_3$) δ 177.0, 155.4, 109.2, 79.5, 72.4, 66.9, 61.8, 54.1, 53.4, 52.6, 51.0, 41.7, 41.4, 40.1, 39.3, 39.2, 38.7, 37.1, 36.9, 33.2, 32.4, 31.2, 30.2, 28.7, 27.4, 23.5, 22.1, 17.1, 15.9, 14.4, 12.6. **HRMS** (ESI) m/z: anal. calculated for $[C_{33}H_{52}N_2O_5 + Na]^+$: 579.3768, found: 579.3771. LC-MS (t_R= 3.62 min, λ = 210 nm, purity 88%).

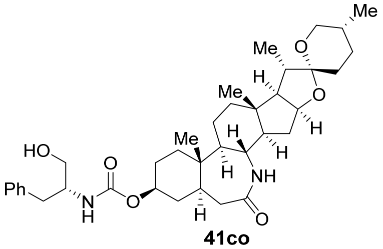

41co

41co, yield: (46.0 mg, 74%). **^{1}H NMR** (400 MHz, $CDCl_3$) δ 7.48 – 7.01 (m, 5H), 5.76 – 5.39 (m, 1H), 5.26 – 4.96 (m, 1H), 4.44 (dq, *J* = 39.0, 7.6, 6.3 Hz, 2H), 4.08 – 3.72 (m, 1H), 3.65 (dd, *J* = 11.2, 3.9 Hz, 1H), 3.55 (dd, *J* = 11.2, 5.0 Hz, 1H), 3.45 (hept, *J* = 4.6 Hz, 2H), 3.36 (t, *J* = 10.9 Hz, 1H), 2.86 (d, *J* = 7.3 Hz, 3H), 2.68 (dd, *J* = 14.4, 10.5 Hz, 1H), 2.15 (td, *J* = 7.2, 3.7 Hz, 1H), 1.97 – 1.52 (m, 13H), 1.53 – 1.24 (m, 7H), 1.24 – 1.01 (m, 4H), 1.00 (s, 3H), 0.97 (d, *J* = 6.5 Hz, 3H), 0.79 (d, *J* = 6.2 Hz, 3H), 0.77 (s, 3H). **^{13}C NMR** (101 MHz, $CDCl_3$) δ 177.3, 156.0, 137.9, 129.3, 128.5, 126.4, 109.2, 79.5, 77.3, 72.8, 66.9, 63.7, 61.8, 54.0, 53.2, 51.1, 41.7, 41.4, 40.1, 39.3, 39.1, 38.7, 37.3, 37.1, 36.8, 32.4, 31.2, 30.2, 28.7, 27.3, 22.0, 17.1, 15.9, 14.4, 12.6. **HRMS** (ESI) m/z: anal. calculated for $[C_{37}H_{54}N_2O_6 + H]^+$: 623.4055, found: 623.4059. LC-MS (t_R= 3.37 min, λ = 210 nm, purity >99%).

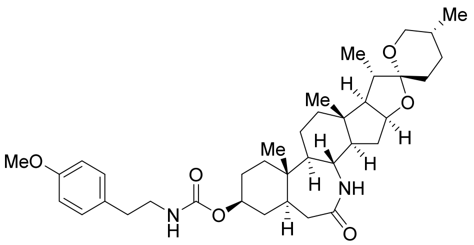

41cp

41cp, yield: (47.9 mg, 77%). **^{1}H NMR** (400 MHz, $CDCl_3$) δ 7.22 (t, *J* = 7.8 Hz, 1H), 6.83 – 6.61 (m, 3H), 5.75 – 5.52 (m, 1H), 4.65 (t, *J* = 5.8 Hz, 1H), 4.52 (dq, *J* = 11.2, 5.6, 4.5 Hz, 1H), 4.46 – 4.31 (m, 1H), 3.80 (s, 3H), 3.59 – 3.20 (m, 5H), 2.78 (t, *J* = 7.0 Hz, 2H), 2.69 (dd, *J* = 14.4, 10.3 Hz, 1H), 2.16 (ddd, *J* = 11.1, 7.5, 4.2 Hz, 1H), 1.97 – 1.53 (m, 13H), 1.53 – 1.24 (m, 6H), 1.24 – 1.04 (m, 3H), 1.01 (s, 3H), 0.97 (d, *J* = 6.6 Hz, 3H), 0.79 (d, *J* = 6.4 Hz, 3H), 0.77 (s, 3H). **^{13}C NMR** (101 MHz, $CDCl_3$) δ 177.2, 159.7, 155.8, 140.4, 129.5, 121.1, 114.4, 111.8, 109.2, 79.6, 72.6, 66.8, 61.8, 55.1, 54.0, 53.3, 51.1, 41.9, 41.7, 41.4, 40.0, 39.3, 39.2, 38.7, 37.1, 36.8, 36.1, 32.4, 31.2, 30.2, 28.7, 27.3, 22.1, 17.1, 15.9, 14.4, 12.6. **HRMS** (ESI) m/z: anal. calculated for $[C_{37}H_{54}N_2O_6 + Na]^+$: 645.3874, found: 645.3881. LC-MS (t_R= 3.07 min, λ = 210 nm, purity >99%).

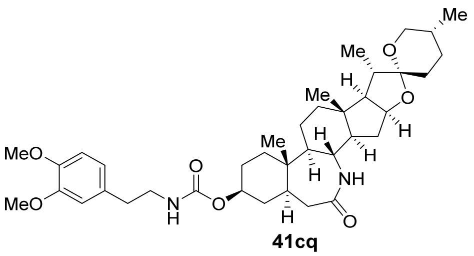

41cq

41cq, yield: (56.7 mg, 87%). **^{1}H NMR** (400 MHz, $CDCl_3$) δ 6.80 (d, *J* = 8.1 Hz, 1H), 6.76 – 6.62 (m, 2H), 5.58 (d, *J* = 11.6 Hz, 1H), 4.64 (t, *J* = 6.2 Hz, 1H), 4.58 – 4.44 (m, 1H), 4.39 (q, *J* = 7.6 Hz, 1H), 3.86 (d, *J* = 3.9 Hz, 7H), 3.56 – 3.18 (m, 5H), 2.88 – 2.56 (m, 3H), 2.15 (ddd, *J* = 11.1, 6.8, 4.2 Hz, 1H), 1.97 – 1.52 (m, 14H), 1.52 – 1.23 (m, 7H), 1.23 – 1.04 (m, 3H), 1.00 (s, 3H), 0.96 (d, *J* = 6.6 Hz, 3H), 0.78 (d, *J* = 6.4 Hz, 3H), 0.76 (s, 3H). **^{13}C NMR** (101 MHz, $CDCl_3$) δ 177.1, 155.8, 148.9, 147.6, 131.3, 120.6, 111.9, 111.3, 109.2, 79.5, 72.6, 66.8, 61.8, 55.9, 55.8, 54.0, 53.3, 51.0, 42.1, 41.7, 41.4, 40.0, 39.3, 39.2, 38.7, 37.1, 36.8, 35.6, 32.4, 31.2, 30.2, 28.7, 27.3, 22.0, 17.0, 15.9, 14.4, 12.6. **HRMS** (ESI) m/z: anal. calculated for $[C_{38}H_{56}N_2O_7 + Na]^+$: 675.3980, found: 675.3978. LC-MS (t_R= 3.01 min, λ = 254 nm, purity 98%).

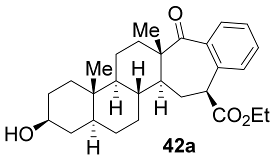

42a

To a solution of **14** (199 mg, 0.36 mol,) in THF (10 mL) was added 2 N HCl (2.0 ml). The reaction mixture was heated at reflux for 2 h. The mixture was cooled to room temperature, diluted with ethyl acetate (50 mL), and washed with $NaHCO_3$ (20 mL × 2) and brine (20 mL × 2). The organic phase was dried over sodium sulfate and concentrated in vacuum. Flash column chromatography over silica column afforded **40a**; Yield: (142 mg, 90%). **^{1}H NMR** (500 MHz, $CDCl_3$) δ 7.40 (td, *J* = 7.4, 1.7 Hz, 1H), 7.37 – 7.28 (m, 2H), 7.13 (dd, *J* = 7.6, 1.2 Hz, 1H), 4.06 (qq, *J* = 10.8, 7.2 Hz, 2H), 3.86 (dd, *J* = 9.3, 4.5 Hz, 1H), 3.54 (tt, *J* = 11.1, 4.8 Hz, 1H), 2.36 (ddd, *J* = 14.7, 9.4, 3.4 Hz, 1H), 2.17 – 1.95 (m, 2H), 1.78 (ddd, *J* = 11.2, 5.2, 2.6 Hz, 1H), 1.73 – 1.49 (m, 5H), 1.48 – 1.21 (m, 7H), 1.19 (s, 3H), 1.15 (t, *J* = 7.1 Hz, 3H), 1.00 (tt, *J* = 12.5, 3.4 Hz, 1H), 0.92 – 0.83 (m, 2H), 0.78 (s, 3H), 0.68 – 0.55 (m, 1H). **^{13}C NMR** (126 MHz, $CDCl_3$) δ 213.7, 173.5, 140.2, 134.3, 130.5, 129.1, 128.0, 127.6, 71.1, 61.3, 53.3, 50.6, 49.5, 45.9, 44.1, 37.8, 37.7, 36.7, 35.6, 35.5, 31.3, 30.8, 28.5, 28.0, 20.4, 14.9, 13.9, 12.2. **HRMS** (ESI) m/z: anal. calculated for $[C_{28}H_{38}O_4 + NH_4]^+$: 456.3108, found: 456.3116. LC-MS (t_R= 3.23 min, λ = 254 nm, purity 95%).

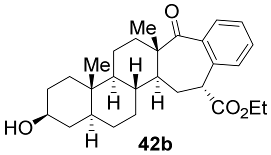

42b

42b, Yield: (147 mg, 84%). **^{1}H NMR** (500 MHz, $CDCl_3$) δ 7.40 (td, *J* = 7.6, 1.5 Hz, 1H), 7.31 (t, *J* = 7.4 Hz, 1H), 7.28 – 7.20 (m, 1H), 7.10 (d, *J* = 7.7 Hz, 1H), 4.32 – 4.14 (m, 2H), 3.71 (dd, *J* = 12.6, 5.9 Hz, 1H), 3.54 (tt, *J* = 10.6, 4.8 Hz, 1H), 2.33 (td, *J* = 13.2, 5.4 Hz, 1H), 1.97 (dq, *J* = 13.0, 3.7 Hz, 1H), 1.85 – 1.67 (m, 3H), 1.67 – 1.45 (m, 4H), 1.43 – 1.14 (m, 14H), 0.99 (tt, *J* = 12.6, 3.3 Hz, 1H), 0.93 – 0.83 (m, 2H), 0.77 (s, 3H), 0.59 (td, *J* = 11.1, 3.6 Hz, 1H). **^{13}C NMR** (126 MHz, $CDCl_3$) δ 215.6, 172.8, 139.8, 134.3, 131.0, 127.4, 126.0, 124.3, 71.1, 61.0, 52.9, 49.0, 45.7, 43.9, 43.9, 37.9, 37.8, 36.8, 36.6, 35.5, 31.3, 31.2, 30.7, 28.4, 20.6, 14.1, 14.0, 12.2. **HRMS** (ESI) m/z: anal. calculated for $[C_{28}H_{38}O_4 + NH_4]^+$: 456.3108, found: 456.3103. LC-MS (t_R= 2.36 min, λ = 254 nm, purity 98%).

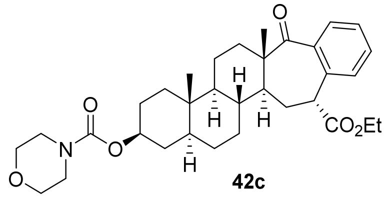

42c

42c, white solid, yield: (42.9 mg, 77%). **^{1}H NMR** ($CDCl_3$, 500 MHz) δ 7.41 (td, *J* = 7.5, 1.0 Hz, 1H), 7.32 (t, *J* = 7.3 Hz, 1H), 7.25 (d, *J* = 7.4 Hz, 1H), 7.10 (d, *J* = 7.8 Hz, 1H), 4.58-4.52 (m, 1H), 4.26-4.17 (m, 2H), 3.70 (dd, *J* = 12.9, 5.8 Hz, 1H), 3.63 (br, 4H), 3.44-3.43 (m, 4H), 2.34 (td, *J* = 13.2, 5.2 Hz, 1H), 1.98-1.95 (m, 1H), 1.84-1.82 (m, 1H), 1.76-1.71 (m, 2H), 1.64-1.58 (m, 3H),

1.49-1.15 (m, 14H), 1.09-1.05 (m, 1H), 0.94 (td, *J* = 13.4, 3.6 Hz, 1H), 0.79 (s, 3H), 0.77-0.72 (m, 1H), 0.64-0.60 (m, 1H). **^{13}C NMR** ($CDCl_3$, 125 MHz) δ 215.7, 172.8, 155.2, 139.9, 134.4, 131.1, 127.5, 126.1, 124.4, 74.7, 61.1, 52.8, 49.0, 45.7, 43.9, 43.8, 37.9, 36.7, 36.6, 35.6, 34.3, 31.3, 30.7, 28.4, 27.8, 20.6, 14.2, 14.1, 12.2. **HRMS** (ESI): *m/z*: calculated for $C_{33}H_{46}NO_6$: $[M + H]^+$ 552.3320, found: 552.3322.

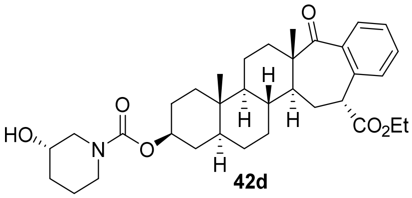

42d

42d, white solid, yield: (54.0 mg, 99%). **^{1}H NMR** ($CDCl_3$, 500 MHz) δ 7.41 (td, *J* = 7.6, 1.0 Hz, 1H), 7.32 (t, *J* = 7.3 Hz, 1H), 7.25 (d, *J* = 7.3 Hz, 1H), 7.09 (d, *J* = 7.8 Hz, 1H), 4.56-4.49 (m, 1H), 4.26-4.17 (m, 2H), 3.75-3.68 (m, 3H), 3.53 (br, 1H), 3.16-3.11 (m, 2H), 2.33 (td, *J* = 13.1, 5.2 Hz, 1H), 1.98-1.95 (m, 1H), 1.87-1.81 (m, 2H), 1.75-1.70 (m, 3H), 1.64-1.57 (m, 3H), 1.53-1.43 (m, 3H), 1.36-1.16 (m, 13H), 1.08-1.03 (m, 1H), 0.93 (td, *J* = 13.5, 3.8 Hz, 1H), 0.79 (s, 3H), 0.76-0.71 (m, 1H), 0.64-0.59 (m, 1H). **^{13}C NMR** ($CDCl_3$, 125 MHz) δ 215.7, 172.8, 155.7, 139.9, 134.4, 131.1, 127.5, 126.1, 124.4, 74.6, 61.1, 52.9, 50.7, 49.0, 45.7, 43.9, 43.8, 37.9, 36.7, 36.6, 35.6, 34.3, 31.3, 30.7, 28.4, 27.8, 20.6, 14.2, 14.1, 12.3. **HRMS** (ESI): *m/z*: calculated for $C_{34}H_{48}NO_6$: $[M + Na]^+$ 566.3476, found: 566.3480.

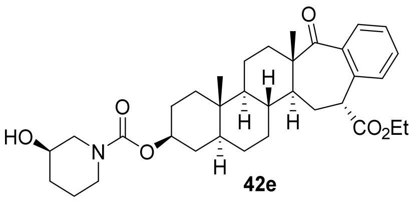

42e

42e, white solid, yield: (15.8 mg, 28%). **^{1}H NMR** ($CDCl_3$, 500 MHz) δ 7.41 (td, *J* = 7.6, 1.0 Hz, 1H), 7.32 (t, *J* = 7.3 Hz, 1H), 7.25 (d, *J* = 7.4 Hz, 1H), 7.09 (d, *J* = 7.6 Hz, 1H), 4.55-4.49 (m, 1H), 4.26-4.17 (m, 2H), 3.76-3.68 (m, 3H), 3.54 (br, 1H), 3.14-3.08 (m, 2H), 2.33 (td, *J* = 13.1, 5.1 Hz, 1H), 1.98-1.95 (m, 1H), 1.87-1.81 (m, 2H), 1.75-1.70 (m, 3H), 1.62-1.57 (m, 3H), 1.52-1.43 (m, 3H), 1.36-1.15 (m, 13H), 1.08-1.03 (m, 1H), 0.93 (td, *J* = 13.6, 3.6 Hz, 1H), 0.79 (s, 3H), 0.76-0.71 (m, 1H), 0.64-0.59 (m, 1H). **^{13}C NMR** ($CDCl_3$, 125 MHz) δ 215.7, 172.8, 155.6, 139.9, 134.4, 131.1, 127.5, 126.1, 124.4, 74.5, 61.1, 52.8, 50.7, 49.0, 45.7, 43.9, 43.8, 37.9, 36.7, 36.6, 35.6, 34.3, 31.3, 30.7, 28.4, 27.8, 20.6, 14.2, 14.1, 12.2. **HRMS** (ESI): *m/z*: calculated for $C_{34}H_{48}NO_6$: $[M + H]^+$ 566.3476, found: 566.3476.

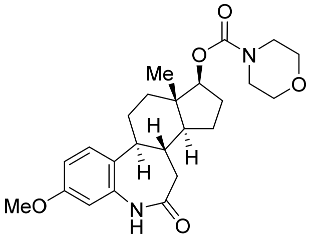

43a

43a, white solid, yield: (11.1 mg, 26%). **^{1}H NMR** ($CDCl_3$, 500 MHz) δ 7.93 (s, 1H), 7.19 (d, *J* = 8.8 Hz, 1H), 6.76 (dd, *J* = 8.5, 2.6 Hz, 1H), 6.54 (d, *J* = 2.5 Hz, 1H), 4.71 (dd, *J* = 9.1, 7.8 Hz, 1H), 3.80 (s, 3H), 3.67 (m, 4H), 3.48-3.46 (m, 4H), 2.49 (td, *J* = 12.1, 3.0 Hz, 1H), 2.44 (dd, *J* = 12.7, 8.6 Hz, 1H), 2.30-2.22 (m, 1H), 2.17 (dd, *J* = 12.8, 1.4 Hz, 1H), 2.04-1.98 (m, 2H), 1.96-1.89 (m, 2H), 1.78-1.68 (m, 2H), 1.62-1.54 (m, 1H), 1.45-1.32 (m, 2H), 0.88 (s, 3H). **^{13}C NMR** ($CDCl_3$, 125 MHz) δ 174.6, 158.7, 155.5, 138.2, 129.7, 126.2, 111.3, 107.7, 83.4, 55.5, 49.1, 45.9, 44.2, 43.7, 36.9, 35.6, 27.8, 25.2, 23.7, 12.5. **HRMS** (ESI): *m/z*: calculated for $C_{24}H_{33}N_2O_5$: $[M + H]^+$ 429.2384, found: 429.2396. LC-MS (t_R= 2.79 min, λ = 254 nm, purity >99%).

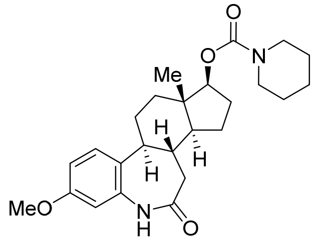

43b

43b, white solid, yield: (11.1 mg, 13%). **^{1}H NMR** ($CDCl_3$, 500 MHz) δ 7.54 (s, 1H), 7.19 (d, *J* = 8.6 Hz, 1H), 6.75 (dd, *J* = 8.6, 2.6 Hz, 1H), 6.53 (d, *J* = 2.5 Hz, 1H), 4.69 (dd, *J* = 9.2, 7.8 Hz, 1H), 3.80 (s, 3H), 3.42 (t, *J* = 4.8 Hz, 4H), 2.49 (td, *J* = 12.2, 2.3 Hz, 1H), 2.43 (dd, *J* = 12.8, 8.5 Hz, 1H), 2.29-2.21 (m, 1H), 2.17 (d, *J* = 12.7, 1.8 Hz, 1H), 2.05-1.97 (m, 2H), 1.96-1.89 (m, 2H), 1.77-1.68 (m, 2H), 1.60-1.53 (m, 7H), 1.44-1.34 (m, 2H), 0.88 (s, 3H). **^{13}C NMR** ($CDCl_3$, 125 MHz) δ 174.2, 158.6, 155.5, 138.2, 129.8, 126.3, 111.2, 107.7, 82.9, 55.5, 49.1, 45.9, 44.9, 44.3, 43.7, 36.9, 35.6, 27.9, 25.2, 24.5, 23.8, 12.5. **HRMS** (ESI): *m/z*: calculated for $C_{25}H_{35}N_2O_4$: $[M + H]^+$ 427.2591, found: 427.2597. LC-MS (t_R= 3.01 min, λ = 254 nm, purity 98%).

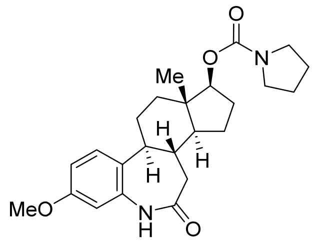

43c

43c, white solid, yield: (10.0 mg, 12%). **^{1}H NMR** ($CDCl_3$, 500 MHz) δ 7.75 (s, 1H), 7.19 (d, *J* = 8.6 Hz, 1H), 6.75 (dd, *J* = 8.6, 2.6 Hz, 1H), 6.53 (d, *J* = 2.5 Hz, 1H), 4.68 (dd, *J* = 9.0, 7.9 Hz, 1H), 3.80 (s, 3H), 3.40-3.33 (m, 4H), 2.49 (td, *J* = 12.1, 2.9 Hz, 1H), 2.43 (dd, *J* = 12.8, 8.5 Hz, 1H), 2.30-2.22 (m, 1H), 2.17 (d, *J* = 12.6, 1.4 Hz, 1H), 2.05-2.00 (m, 2H), 1.94-1.84 (m, 6H), 1.77-1.68 (m, 2H), 1.62-1.54 (m, 1H), 1.44-1.34 (m, 2H), 0.89 (s, 3H). **^{13}C NMR** ($CDCl_3$, 125 MHz) δ 174.5, 158.6, 155.2, 138.2, 129.8, 126.3, 111.3, 107.7, 82.7, 55.5, 49.1, 46.0, 44.3, 43.7, 36.8, 35.6, 28.0, 25.2, 23.8, 12.4. **HRMS** (ESI): *m/z*: calculated for $C_{24}H_{33}N_2O_4$: $[M + H]^+$ 413.2435, found: 413.2442. LC-MS (t_R= 2.97 min, λ = 254 nm, purity 97%).

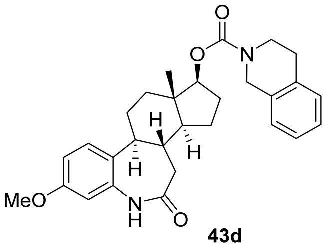

43d

43d, white solid, yield: (17.0 mg, 18%). **^{1}H NMR** ($CDCl_3$, 500 MHz) δ 7.87 (s, 1H), 7.20-7.11 (m, 5H), 6.75 (dd, *J* = 8.7, 2.5 Hz, 1H), 6.54 (d, *J* = 2.4 Hz, 1H), 4.75 (dd, *J* = 8.9, 8.0 Hz, 1H), 4.63 (m, 2H), 3.80 (s, 3H), 3.71 (t, *J* = 5.7 Hz, 2H), 2.86 (t, *J* = 5.7 Hz, 2H), 2.49 (td, *J* = 12.1, 2.9 Hz, 1H), 2.43 (dd, *J* = 12.8, 8.6 Hz, 1H), 2.32-2.24 (m, 1H), 2.18 (d, *J* = 12.9, 1.7 Hz, 1H), 2.05-1.99 (m, 2H), 1.97-1.90 (m, 2H), 1.78-1.69 (m, 2H), 1.65-1.58 (m, 1H), 1.46-1.36 (m, 2H), 0.92 (s, 3H). **^{13}C NMR** ($CDCl_3$, 125 MHz) δ 174.6, 158.6, 138.1, 129.7, 126.6, 126.3, 111.3, 107.7, 83.2, 55.5, 49.1, 46.0, 45.8, 44.2, 43.8, 36.9, 35.6, 27.9, 25.2, 23.7, 12.6. **HRMS** (ESI): *m/z*: calculated for $C_{29}H_{35}N_2O_4$: $[M + H]^+$ 475.2591, found: 475.2596. LC-MS (t_R= 3.15 min, λ = 254 nm, purity 98%).

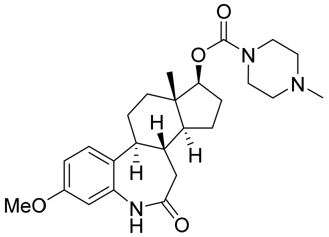

43e

43e, white solid, yield: (15.1 mg, 36%). **^{1}H NMR** ($CDCl_3$, 500 MHz) δ 8.03 (s, 1H), 7.18 (d, *J* = 8.6 Hz, 1H), 6.76 (dd, *J* = 8.5, 2.4 Hz, 1H), 6.55 (d, *J* = 2.8 Hz, 1H), 4.69 (dd, *J* = 8.9, 7.8 Hz, 1H), 3.80 (s, 3H), 3.67 (br, 4H), 2.79 (br, 4H), 2.50-2.41 (m, 2H), 2.29-2.21 (m, 1H), 2.16 (d, *J* = 13.5 Hz, 1H), 2.04-1.97 (m, 2H), 1.96-1.87 (m, 2H), 1.77-1.67 (m, 2H), 1.60-1.53 (m, 1H), 1.45-1.32 (m, 2H), 0.87 (s, 3H). **^{13}C NMR** ($CDCl_3$, 125 MHz) δ 174.5, 158.7, 155.0, 138.2, 129.6, 126.2, 111.2, 107.8, 83.7, 55.5, 53.5, 49.0, 45.9, 44.4, 44.2, 43.7, 36.8, 35.6, 27.8, 25.2, 23.7, 12.5. **HRMS** (ESI): *m/z*: calculated for $C_{25}H_{36}N_3O_4$: $[M + H]^+$ 442.2700, found: 442.2706. LC-MS (t_R= 2.07 min, λ = 254 nm, purity >99%).

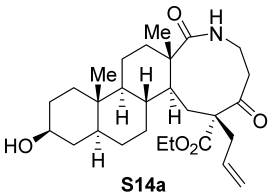

S14a

S14a, white solid, yield: (125 mg, 88%). **^{1}H NMR** ($CDCl_3$, 400 MHz) δ 6.18-6.17 (m, 1H), 5.61-5.50 (m, 1H), 5.06-4.99 (m, 2H), 4.31-4.23 (m, 1H), 4.17-4.07 (m, 2H), 3.62-3.57 (m, 1H), 3.20-3.16 (m, 1H), 3.08-3.02 (m, 1H), 2.68-2.63 (m, 1H), 2.45-2.40 (m, 1H), 1.86-1.81 (m, 2H), 1.73-1.57 (m, 8H), 1.43-1.10 (m, 13H), 1.07 (s, 3H), 1.02-0.93 (m, 1H), 0.86-0.82 (m, 1H), 0.79 (s, 3H). **^{13}C NMR** ($CDCl_3$, 100 MHz) δ 210.5, 180.3, 171.8, 132.9, 119.1, 71.1, 65.9, 61.1, 53.5, 48.0, 46.7, 44.8, 42.6, 38.8, 37.9, 37.6, 37.2, 37.1, 36.7, 36.1, 35.5, 31.4, 30.8, 28.9, 20.0, 14.7, 14.0, 12.3.

HRMS (ESI): *m/z*: calculated for $C_{28}H_{43}NNaO_5$: $[M + Na]^+$ 496.3033, found: 496.3019.

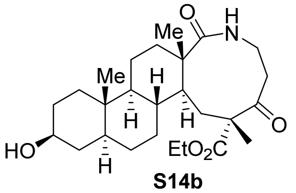

S14b

S14b, white solid, yield: (118 mg, 88%). **^{1}H NMR** ($CDCl_3$, 400 MHz) δ 6.17-6.15 (m, 1H), 4.23-4.15 (m, 2H), 4.08-4.03 (m, 1H), 3.66-3.60 (m, 1H), 3.30-3.20 (m, 2H), 2.24-2.21 (m, 1H), 1.83-1.59 (m, 10H), 1.47-1.24 (m, 13H), 1.08 (s, 3H), 1.03-0.86 (m, 4H), 0.80 (s, 3H). **^{13}C NMR** ($CDCl_3$, 100 MHz) δ 211.6, 180.8, 172.3, 71.1, 62.2, 61.0, 53.3, 48.1, 46.7, 44.8, 39.5, 38.0, 37.9, 37.4, 36.8, 36.1, 35.8, 35.1, 31.4, 30.6, 28.9, 23.6, 20.1, 14.5, 14.0, 12.4. **HRMS** (ESI): *m/z*: calculated for $C_{26}H_{42}NO_5$: $[M + H]^+$ 448.3058, found: 448.3058.

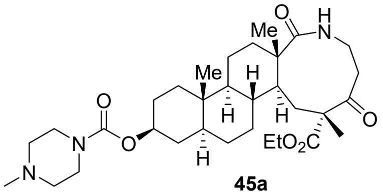

45a

45a, white solid, yield: (516 mg, 90%). **^{1}H NMR** ($CDCl_3$, 400 MHz) δ 6.07-6.05 (m, 1H), 4.61-4.56 (m, 1H), 4.20-4.17 (m, 2H), 4.10-4.14 (m, 1H), 3.59 (br, 4H), 3.22-3.20 (m, 2H), 2.63 (br, 4H), 2.44 (s, 3H), 2.31-2.28 (m, 1H), 1.86-1.59 (m, 10H), 1.49-1.40 (m, 2H), 1.34-1.26 (m, 10H), 1.19-1.17 (m, 2H), 1.08 (s, 3H), 0.90-0.85 (m, 2H), 0.81 (s, 3H). **^{13}C NMR** ($CDCl_3$, 100 MHz) δ 211.6, 180.7, 172.5, 154.9, 74.8, 62.1, 61.1, 53.8, 53.5, 48.1, 46.8, 42.4, 39.5, 38.0, 37.4, 36.5, 36.1, 36.0, 35.2, 34.3, 30.6, 28.8, 27.7, 23.7, 20.0, 14.5, 14.0, 12.3. **HRMS** (ESI): *m/z*: calculated for $C_{32}H_{52}N_3O_6$: $[M + H]^+$ 574.3851, found: 574.3849.

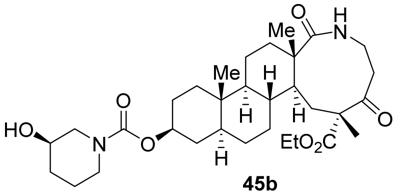

45b

45b, white solid, yield: (51.0 mg, 89%). **^{1}H NMR** ($CDCl_3$, 400 MHz) δ 6.09-6.07 (m, 1H), 4.58-4.55 (m, 1H), 4.19-4.17 (m, 2H), 4.09-4.03 (m, 1H), 3.74 (d, *J* = 10.5 Hz, 2H), 3.55-3.52 (m, 1H), 3.21-3.11 (m, 4H), 2.30-2.27 (m, 1H), 1.86-1.63 (m, 13H), 1.50-1.46 (m, 4H), 1.36-1.29 (m, 10H), 1.19-1.16 (m, 2H), 1.07 (s, 3H), 0.89-0.87 (m, 2H), 0.81 (s, 3H). **^{13}C NMR** ($CDCl_3$, 100 MHz) δ 211.6, 180.7, 172.5, 155.6, 74.4, 66.1, 62.1, 61.1, 53.3, 50.6, 48.1, 46.7, 44.6, 39.5, 38.0, 37.4, 36.6, 36.1, 36.0, 35.2, 34.3, 30.6, 28.8, 27.8, 23.7, 20.3, 14.5, 14.0, 12.3. **HRMS** (ESI): *m/z*: calculated for $C_{32}H_{51}N_2O_7$: $[M + H]^+$ 575.3691, found: 575.3697.

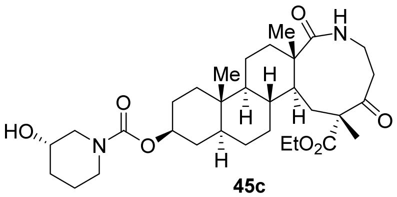

45c

45c, white solid, yield: (52.8 mg, 92%). **^{1}H NMR** ($CDCl_3$, 400 MHz) δ 6.10-6.09 (m, 1H), 4.60-4.55 (m, 1H), 4.21-4.16 (m, 2H), 4.09-4.03 (m, 1H), 3.74 (d, *J* = 10.8 Hz, 2H), 3.55 (br, 1H), 3.22-3.13 (m, 4H), 2.31-2.27 (m, 1H), 1.87-1.63 (M, 13H), 1.52-1.45 (m, 4H), 1.34-1.26 (m, 10H), 1.19-1.17 (m, 2H), 1.07 (s, 3H), 0.91-0.84 (m, 2H), 0.81 (s, 3H). **^{13}C NMR** ($CDCl_3$, 100 MHz) δ 211.6, 180.7, 172.5, 155.6, 74.4, 66.2, 62.1, 61.1, 53.3, 50.6, 48.1, 46.7, 44.6, 39.5, 38.0, 37.4, 36.6, 36.1, 36.0, 35.2, 34.3, 30.6, 28.8, 27.8, 23.7, 22.3, 20.0, 14.5, 14.0, 12.3. **HRMS** (ESI): *m/z*: calculated for $C_{32}H_{51}N_2O_7$: $[M + H]^+$ 575.3691, found: 575.3692.

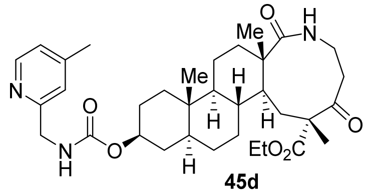

45d

45d, white solid, yield: (10.1 mg, 17%). **^{1}H NMR** ($CDCl_3$, 400 MHz) δ 8.39 (d, *J* = 5.0 Hz, 1H), 7.21 (s, 1H), 7.10 (d, *J* = 4.4 Hz, 1H), 6.09 (d, *J* = 7.2 Hz, 1H), 5.91 (m, 1H), 4.60-4.55 (m, 1H), 4.45 (d, *J* = 5.5 Hz, 2H), 4.22-4.16 (m, 2H), 4.09-4.03 (m, 1H), 3.22-3.20 (m, 4H), 2.38 (s, 3H), 2.31-2.28 (m, 1H), 1.83-1.79 (m, 2H), 1.72-1.63 (m, 6H), 1.48-1.42 (m, 2H), 1.35-1.29 (m, 9H), 1.17-1.15 (m, 2H), 1.07 (s, 3H), 1.03-0.97 (m, 1H), 0.88-0.82 (m, 2H), 0.79 (s, 3H). **^{13}C NMR** ($CDCl_3$, 100 MHz) δ 211.6, 180.8, 172.5, 156.6, 156.5, 149.5, 147.7, 123.8, 123.6, 74.1, 62.1, 61.1, 53.3, 48.1, 46.8, 45.3, 44.7, 39.5, 38.0, 37.4, 36.6, 36.1, 36.0, 35.2, 34.2, 30.6, 28.7, 27.7, 23.7, 21.2, 20.0, 14.5, 14.0, 12.3. **HRMS** (ESI): *m/z*: calculated for $C_{34}H_{50}N_3O_6$: $[M + H]^+$ 596.3694, found: 596.3692.

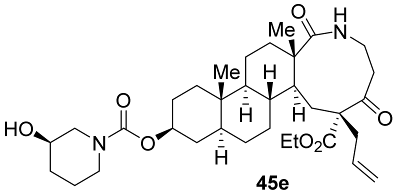

45e

48e, white solid, yield: (34.2 mg, 57%). **^{1}H NMR** ($CDCl_3$, 400 MHz) δ 6.19-6.17 (m, 1H), 5.61-5.50 (m, 1H), 5.01-4.97 (m, 2H), 4.61-4.54 (m, 1H), 4.31-4.23 (m, 1H), 4.16-4.10 (m, 2H), 3.75 (d, *J* = 10.6 Hz, 2H), 3.56-3.53 (m, 1H), 3.21-3.10 (m, 3H), 3.02-2.95 (m, 1H), 2.66-2.61 (m, 1H), 2.51-2.40 (m, 2H), 1.88-1.40 (m, 16H), 1.34-1.19 (m, 9H), 1.06 (s, 3H), 1.01-0.98 (m, 1H), 0.88-0.82 (m, 2H), 0.80 (s, 3H). **^{13}C NMR** ($CDCl_3$, 100 MHz) δ 210.5, 180.1, 172.0, 155.6, 132.9, 119.1, 74.4, 66.1, 65.9, 61.2, 53.4, 50.6, 47.9, 46.7, 44.6, 42.7, 38.7, 37.8, 37.2, 37.1, 36.6, 36.1, 35.6, 34.3, 30.8, 28.7, 27.8, 19.9, 14.7, 14.0, 12.3. **HRMS** (ESI): *m/z*: calculated for $C_{34}H_{52}N_2NaO_7$: $[M + Na]^+$ 623.3667, found: 623.3647.

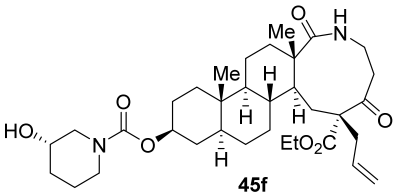

45f

45f, white solid, yield: (40.8 mg, 68%). **^{1}H NMR** ($CDCl_3$, 500 MHz) δ 6.17-6.15 (m, 1H), 5.60-5.51 (m, 1H), 5.01-4.98 (m, 2H), 4.60-4.55 (m, 1H), 4.30-4.24 (m, 1H), 4.18-4.10 (m, 2H), 3.75 (d, *J* = 10.4 Hz, 2H), 3.55 (br, 1H), 3.19-3.13 (m, 3H), 3.00-2.97 (m, 1H), 4.65-4.61 (m, 1H), 2.50-2.41 (m, 2H), 1.86-1.41 (m, 16H), 1.33-1.19 (m, 9H), 1.07 (s, 3H), 1.01-0.99 (m, 1H), 0.89-0.84 (m, 2H), 0.80 (s, 3H). **^{13}C NMR** ($CDCl_3$, 125 MHz) δ 210.6, 180.2, 172.1, 155.5, 133.0, 119.2, 74.5, 66.3, 66.0, 61.3, 53.5, 50.7, 48.0, 46.8, 44.7, 42.8, 38.8, 37.9, 37.3, 37.2, 36.6, 36.1, 35.7, 34.4, 30.9, 28.8, 27.8, 20.0, 14.8, 14.1, 12.4. **HRMS** (ESI): *m/z*: calculated for $C_{34}H_{52}N_2NaO_7$: $[M + Na]^+$ 623.3667, found: 623.3639.

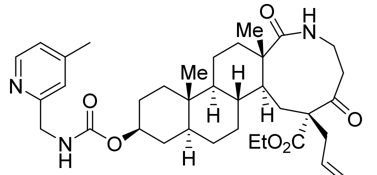

45g

45g, white solid, yield: (17.3 mg, 28%). **^{1}H NMR** ($CDCl_3$, 400 MHz) δ 8.38 (d, *J* = 4.0 Hz, 1H), 7.14 (s, 1H), 7.04 (d, *J* = 4.0 Hz 1H), 6.16 (d, *J* = 8.0 Hz, 1H), 5.77 (s, 1H), 5.01-5.60 (m, 1H), 4.98-5.01 (m, 2H), 4.56-4.61 (m, 1H), 4.43 (d, *J* = 4.0 Hz, 2H), 4.25-4.31 (m, 1H), 4.10-4.18 (m, 2H), 3.15-3.21 (m, 1H), 2.95-3.01 (m, 1H), 2.40-2.66 (m, 6H), 2.36 (s, 1H), 1.83 (dd, *J* = 16.0, 6.0 Hz, 1H), 1.54-1.71 (m, 6H), 1.18-1.48 (m, 3H), 1.06 (s, 2H), 0.72-0.86 (m, 4H). **HRMS** (ESI): *m/z*: calculated for $C_{32}H_{55}NO_2$: $[M + H]^+$ 622.3851, found: 622.3849.

General procedure for syntheses of azide

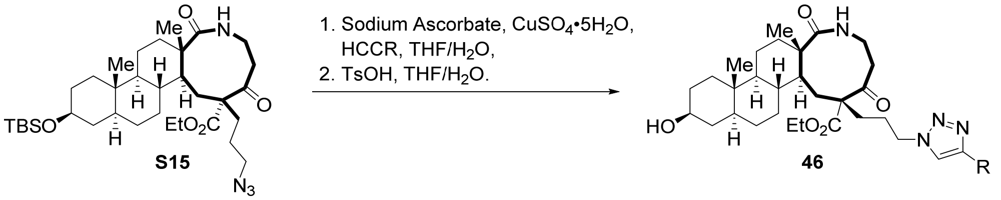

Supplementary Figure 14. Synthesis of azide. General procedure B was used for alkylation of **17**.

To a vial containing azide **S5** (0.25 mmol) was added a solution of copper sulfate pentahydrate (187 mg, 0.125 mmol) and sodium ascorbate (75 mg, 0.375 mmol) in 2:1 water/*t*-butanol (6 ml) followed by alkyne (0.75 mmol). Dichloromethane (3.0 ml) was then added to vials help dissolve the azide. The reaction was stirred at room temperature for 24 hours then diluted with diethyl ether (50 mL), and washed with brine (10 mL × 2). The organic phase was dried over sodium sulfate and concentrated in vacuum to afford crude product which was directly used in the next step without further purification. To a solution of the above product in THF/H_2O (5:1, 10 mL) was added TsOH

(19.0 mg, 0.1 mmol). The reaction mixture was stirred overnight. The mixture was diluted with EtOAc (50 mL), and washed with brine (10 mL × 2). The organic phase was dried over sodium sulfate and concentrated in vacuum. Flash column chromatography over silica column afforded **46**.

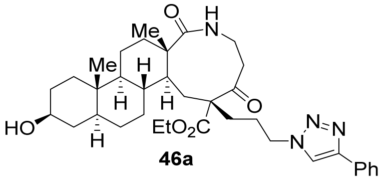

46a

46a, yield: (51.9 mg, 84%). **^{1}H NMR** (500 MHz, $CDCl_3$) δ 7.82 (d, J = 7.6 Hz, 2H), 7.71 (s, 1H), 7.43 (t, J = 7.6 Hz, 2H), 7.34 (t, J = 7.4 Hz, 1H), 6.23 (d, J = 8.9 Hz, 1H), 4.44 – 4.01 (m, 5H), 3.58 (tt, J = 10.7, 4.9 Hz, 1H), 3.17 (ddt, J = 12.9, 5.8, 3.0 Hz, 1H), 2.93 (dt, J = 12.6, 6.4 Hz, 1H), 2.60 (dt, J = 11.6, 5.6 Hz, 1H), 1.99 – 1.46 (m, 12H), 1.48 – 1.12 (m, 9H), 1.12 – 0.90 (m, 5H), 0.86 – 0.73 (m, 4H), 0.68 (qd, J = 12.2, 3.9 Hz, 1H). **^{13}C NMR** (126 MHz, $CDCl_3$) δ 210.7, 179.9, 171.7, 147.8, 130.4, 128.8, 128.1, 125.6, 119.4, 70.9, 65.4, 61.3, 53.4, 50.1, 47.8, 46.6, 44.7, 38.6, 38.1, 37.8, 37.6, 37.1, 36.6, 36.0, 35.5, 35.0, 31.3, 30.8, 28.7, 26.6, 19.8, 14.6, 13.9, 12.2. **HRMS** (ESI) m/z: anal. calculated for $[C_{36}H_{50}N_4O_5 + H]^+$: 619.3854, found: 619.3839. LC-MS (t_R= 1.81 min, λ = 254 nm, purity 98%).

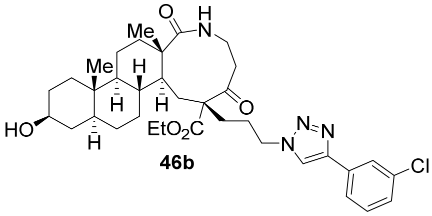

46b

46b, yield: (46.9 mg, 72%). **^{1}H NMR** (500 MHz, $CDCl_3$) δ 7.82 (s, 1H), 7.78 – 7.61 (m, 2H), 7.49 – 7.20 (m, 2H), 6.24 (dd, J = 9.5, 3.5 Hz, 1H), 4.48 – 3.93 (m, 5H), 3.58 (dt, J = 11.1, 5.8 Hz, 1H), 3.30 – 3.12 (m, 1H), 2.93 (dt, J = 12.5, 6.3 Hz, 1H), 2.61 (dd, J = 11.8, 5.9 Hz, 1H), 2.00 – 1.47 (m, 12H), 1.49 – 1.12 (m, 9H), 1.12 – 0.85 (m, 6H), 0.86 – 0.74 (m, 4H), 0.68 (qd, J = 12.4, 4.0 Hz, 1H). **^{13}C NMR** (126 MHz, $CDCl_3$) δ 210.7, 179.9, 171.7, 146.5, 134.7, 132.2, 130.11, 128.1, 125.7, 123.7, 119.9, 70.9, 65.4, 61.3, 53.4, 50.2, 47.8, 46.6, 44.7, 38.6, 38.1, 37.8, 37.56, 37.1, 36.6, 36.0, 35.5, 35.0, 31.3, 30.8, 28.7, 26.6, 19.8, 14.6, 13.9, 12.2. **HRMS** (ESI) m/z: anal. calculated for $[C_{36}H_{49}Cl\,N_4O_5 + Na]^+$: 675.3284, found: 675.3273. LC-MS (t_R= 2.65 min, λ = 254 nm, purity 99%).

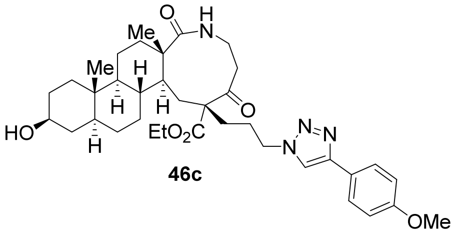

46c

46c, yield: (49.2 mg, 76%). **^{1}H NMR** (500 MHz, Chloroform-*d*) δ 7.74 (d, J = 8.7 Hz, 2H), 7.63 (s, 1H), 7.03 – 6.85 (m, 2H), 6.29 (dd, J = 9.7, 3.4 Hz, 1H), 4.48 – 3.99 (m, 5H), 3.84 (s, 3H), 3.58 (tt, J = 10.7, 4.8 Hz, 1H), 3.24 – 3.10 (m, 1H), 2.94 (dt, J = 12.6, 6.3 Hz, 1H), 2.58 (dt, J = 11.9, 5.8 Hz, 1H), 2.04 (s, 2H), 1.97 – 1.47 (m, 13H), 1.49 – 1.12 (m, 10H), 1.12 – 1.00 (m, 4H), 0.94 (td, J

= 13.5, 3.9 Hz, 1H), 0.85 – 0.73 (m, 4H), 0.68 (qd, J = 12.4, 4.2 Hz, 1H). **^{13}C NMR** (126 MHz, $CDCl_3$) δ 210.7, 179.9, 171.6, 159.5, 147.6, 126.9, 123.1, 118.6, 114.2, 70.9, 65.4, 61.2, 55.2, 53.4, 50.0, 47.7, 46.6, 44.7, 38.6, 38.0, 37.7, 37.4, 37.0, 36.6, 36.0, 35.4, 34.9, 31.2, 30.7, 28.7, 26.6, 19.8, 14.6, 13.8, 12.2. **HRMS** (ESI) m/z: anal. calculated for $[C_{37}H_{52}N_4O_6 + H]^+$: 649.3960, found: 649.3955. LC-MS (t_R= 1.96 min, λ = 254 nm, purity 98%).

General procedure for syntheses of imide.

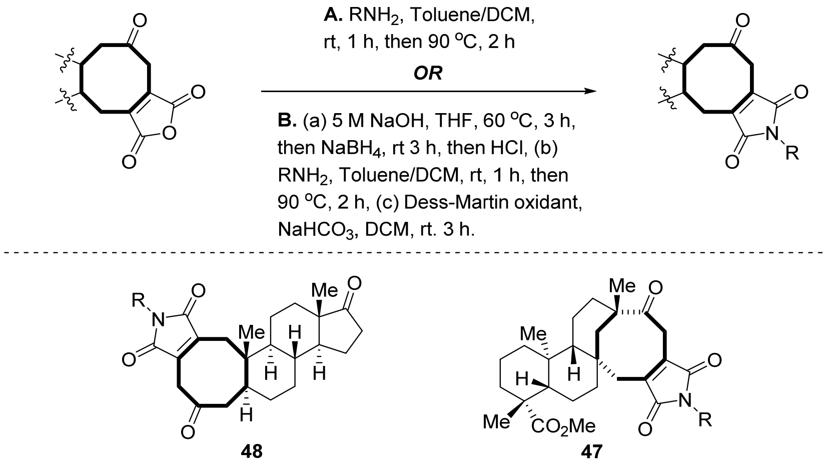

Supplementary Figure 15. Synthesis of imides. General procedure H was used for preparation of **47**, DMF was used as solvent for amino acid. General procedure I was used for preparation of **48**.

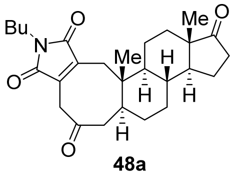

48a

48a, yield: 23.7 mg, 54%. **^{1}H NMR** (500 MHz, $CDCl_3$) δ 3.62 (d, J = 17.8 Hz, 1H), 3.50 (t, J = 7.2 Hz, 2H), 3.36 (d, J = 18.1 Hz, 1H), 2.85 – 2.63 (m, 2H), 2.44 (dd, J = 19.3, 8.4 Hz, 1H), 2.33 (dq, J = 13.3, 3.5 Hz, 1H), 2.21 (dd, J = 15.7, 2.2 Hz, 1H), 2.17 – 1.99 (m, 2H), 1.99 – 1.75 (m, 4H), 1.64 – 1.43 (m, 5H), 1.37 – 1.12 (m, 6H), 1.10 – 0.97 (m, 1H), 0.96 (s, 3H), 0.92 (t, J = 7.3 Hz, 3H), 0.87 (s, 3H), 0.63 (td, J = 9.4, 8.5, 5.1 Hz, 1H). **^{13}C NMR** (126 MHz, $CDCl_3$) δ 220.4, 208.7, 171.2, 170.3, 137.7, 137.2, 51.6, 50.4, 49.2, 47.6, 42.2, 40.1, 38.1, 35.8, 35.1, 31.5, 30.5, 30.5, 30.4, 29.7, 29.5, 21.6, 20.9, 19.9, 15.0, 13.9, 13.6. **HRMS** (ESI) m/z: anal. calculated for $[C_{27}H_{37}NO_4 + Na]^+$: 462.2615, found: 462.2604.

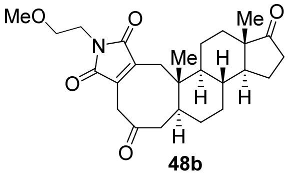

48b

48b, yield: 79.2 mg, 60%. **^{1}H NMR** (500 MHz, $CDCl_3$) δ 3.66 (td, J = 5.6, 1.6 Hz, 2H), 3.61 (d, J = 17.9 Hz, 1H), 3.53 – 3.42 (m, 2H), 3.32 (d, J = 18.1 Hz, 1H), 3.27 (d, J = 1.8 Hz, 3H), 2.79 – 2.62

(m, 2H), 2.40 (dd, J = 19.2, 9.1 Hz, 1H), 2.29 (dd, J = 13.5, 3.8 Hz, 1H), 2.19 (dt, J = 15.7, 2.0 Hz, 1H), 2.14 – 1.71 (m, 6H), 1.56 – 1.39 (m, 3H), 1.39 – 1.09 (m, 3H), 0.99 (td, J = 12.3, 11.7, 6.4 Hz, 1H), 0.92 (s, 3H), 0.83 (s, 3H), 0.68 – 0.51 (m, 1H). **^{13}C NMR** (126 MHz, $CDCl_3$) δ 220.3, 208.5, 170.9, 170.0, 137.7, 137.3, 69.1, 58.4, 51.4, 50.1, 49.1, 47.5, 42.5, 42.1, 40.0, 37.5, 35.7, 35.7, 35.0, 31.4, 30.3, 30.3, 29.3, 21.5, 20.8, 14.9, 13.8. **HRMS** (ESI) m/z: anal. calculated for $[C_{26}H_{35}NO_5 + Na]^+$: 464.2407, found: 464.2393. LC-MS (t_R= 2.48 min, λ = 254 nm, purity 97%).

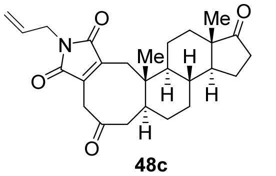

48c

48c, yield: 71.0 mg, 56%. **^{1}H NMR** (500 MHz, $CDCl_3$) δ 5.78 (ddt, J = 17.3, 9.9, 5.7 Hz, 1H), 5.22 – 5.08 (m, 2H), 4.10 (dt, J = 5.7, 1.5 Hz, 2H), 3.64 (dd, J = 18.1, 1.3 Hz, 1H), 3.36 (d, J = 18.1 Hz, 1H), 2.73 (dd, J = 15.6, 12.3 Hz, 2H), 2.44 (ddd, J = 19.2, 8.9, 1.0 Hz, 1H), 2.32 (dq, J = 13.3, 3.6 Hz, 1H), 2.22 (dd, J = 15.7, 2.3 Hz, 1H), 2.18 – 1.99 (m, 2H), 1.99 – 1.77 (m, 3H), 1.59 – 1.45 (m, 4H), 1.40 – 1.14 (m, 4H), 1.02 (ddd, J = 18.3, 7.0, 5.3 Hz, 1H), 0.96 (s, 3H), 0.87 (s, 3H), 0.63 (ddd, J = 12.1, 10.3, 3.7 Hz, 1H). **^{13}C NMR** (126 MHz, $CDCl_3$) δ 220.3, 208.4, 170.5, 169.7, 137.8, 137.4, 131.5, 117.6, 51.4, 50.3, 49.1, 47.5, 42.7, 42.2, 40.3, 40.1, 35.7, 35.0, 31.4, 30.4, 30.3, 29.4, 21.6, 20.8, 15.0, 13.9. **HRMS** (ESI) m/z: anal. calculated for $[C_{26}H_{33}NO_4 + Na]^+$: 446.2302, found: 446.2286. LC-MS (t_R= 2.27 min, λ = 254 nm, purity 98%).

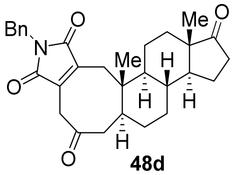

48d

48d, yield: 73.4 mg, 52%. **^{1}H NMR** (500 MHz, $CDCl_3$) δ 7.35 – 7.17 (m, 5H), 4.73 – 4.51 (m, 2H), 3.62 (d, J = 18.2 Hz, 1H), 3.33 (d, J = 18.2 Hz, 1H), 2.83 – 2.63 (m, 2H), 2.44 (dd, J = 19.2, 8.9 Hz, 1H), 2.31 (dq, J = 13.3, 3.6 Hz, 1H), 2.20 (dd, J = 15.7, 2.3 Hz, 1H), 2.15 – 1.98 (m, 2H), 1.96 – 1.74 (m, 4H), 1.48 (dtd, J = 15.0, 8.7, 8.1, 4.1 Hz, 4H), 1.39 – 1.24 (m, 1H), 1.23 – 1.12 (m, 2H), 1.03 – 0.95 (m, 1H), 0.94 (s, 3H), 0.86 (s, 3H), 0.58 (ddd, J = 12.0, 10.3, 3.7 Hz, 1H). **^{13}C NMR** (126 MHz, $CDCl_3$) δ 220.3, 208.4, 170.7, 169.8, 137.9, 137.5, 136.1, 128.6, 128.3, 127.8, 51.4, 50.2, 49.1, 47.5, 42.5, 42.1, 41.9, 40.0, 35.7, 35.0, 31.4, 30.4, 30.3, 29.3, 21.5, 20.8, 14.9, 13.9. **HRMS** (ESI) m/z: anal. calculated for $[C_{30}H_{35}NO_4 + K]^+$: 512.2200, found: 512.2188. LC-MS (t_R= 2.97 min, λ = 254 nm, purity 97%).

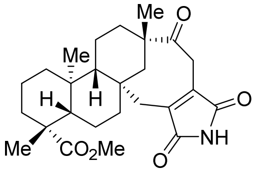

47aa

47aa, White solid, 26.0 mg, yield 59%. **^{1}H NMR** (500 MHz, $CDCl_3$) δ 7.62 (s, 1H), 3.86 (d, J =

18.5 Hz, 1H), 3.67 (s, 3H), 3.16 (d, J = 18.5 Hz, 1H), 2.83 (d, J = 14.0 Hz, 1H), 2.49 (dd, J = 13.5, 3.5 Hz, 1H), 2,17-2.07 (m, 3H), 1.93 (dd, J = 15.0, 3.0 Hz, 1H), 1.89-1.73 (m, 3H), 1.60 (d, J = 14.0 Hz, 1H), 1.48-1.39 (m, 2H), 1.35 (d, J = 13.5 Hz, 1H), 1.16 (s, 3H), 1.11 (s, 3H), 1.10-1.04 (m, 2H), 1.03-0.96 (m, 3H), 0.92-0.81 (m, 2H), 0.66 (s, 3H). **^{13}C NMR** (125 MHz, $CDCl_3$) δ 212.0, 177.9, 171.1, 170.3, 140.7, 138.7, 59.0, 57.5, 51.4, 50.0, 48.5, 44.0, 40.4, 40.1, 39.9, 38.4, 37.6, 37.1, 34.7, 29.5, 28.7, 22.7, 19.7, 19.2, 18.4, 14.2. **HRMS** (ESI): *m/z*: calculated for $C_{26}H_{35}NO_5Na$: $[M + Na]^+$ 464.2407, found: 464.2395. LC-MS (t_R= 0.56 min, λ = 254 nm, purity >99%).

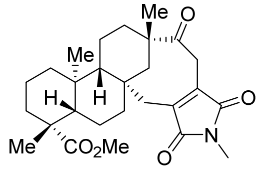

47ab

47ab, White solid, 20.9 mg, 46%. **^{1}H NMR** (500 MHz, $CDCl_3$) δ 3.87 (dd, J = 18.5, 2.0 Hz, 1H), 3.68 (s, 3H), 3.18 (dd, J = 18.5, 1.5 Hz, 1H), 3.01 (s, 3H), 2.86 (d, J = 14.0 Hz, 1H), 2.51 (dq, J = 13.5, 3.0 Hz, 1H), 2.18-2.08 (m, 3H), 1.93 (dd, J = 14.5, 3.0 Hz, 1H), 1.87 (dt, J = 14.0, 4.0 Hz, 1H), 1.81 (dq, J = 15.0, 3.0 Hz, 1H), 1.75 (d, J = 12.5 Hz, 1H), 1.62-1.59 (m, 1H), 1.50-1.41 (m, 2H), 1.35 (dt, J = 13.0, 3.0 Hz, 1H), 1.17 (s, 3H), 1.12 (s, 3H), 1.09-1.06 (m, 2H), 1.03-0.96 (m, 3H), 0.92-0.82 (m, 2H), 0.67 (s, 3H). **^{13}C NMR** (125 MHz, $CDCl_3$) δ 211.9, 177.7, 171.5, 170.5, 139.4, 137.6, 58.7, 57.2, 51.1, 49.8, 48.2, 43.8, 40.2, 39.8, 39.6, 38.2, 37.4, 37.0, 34.4, 29.3, 28.5, 24.1, 22.5, 19.5, 19.0, 18.2, 14.0. **HRMS** (ESI): *m/z*: calculated for $C_{27}H_{41}N_2O_5$: $[M + NH_4]^+$ 473.3010, found: 473.2998. LC-MS (t_R= 0.72 min, λ = 254 nm, purity >99%).

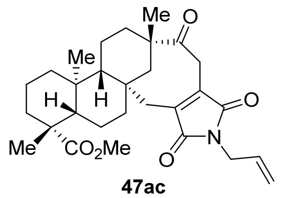

47ac

47ac, White solid, 41.8 mg, yield 87%. **^{1}H NMR** (500 MHz, $CDCl_3$) δ 5.80 (ddt, J = 17.5, 10,0, 5.5 Hz, 1H), 5.19-5.15 (m, 2H), 4.12-4.10 (m, 2H), 3.88 (dd, J = 18.5, 1.5 Hz, 1H), 3.67 (s, 3H), 3.19 (dd, J = 18.5, 1.5 Hz, 1H), 2.87 (d, J = 14.0 Hz, 1H), 2.51 (dq, J = 13.5, 2.5 Hz, 1H), 2.18-2.09 (m, 3H), 1.93 (dd, J = 14.5, 2.5 Hz, 1H), 1.87 (dt, J = 14.0, 4.0 Hz, 1H), 1.81 (dq, J = 14.5, 3.0 Hz, 1H), 1.75 (d, J = 12.5 Hz, 1H), 1.63-1.59 (m, 1H), 1.50-1.41 (m, 2H), 1.34 (dt, J = 13.0, 3.5 Hz, 1H), 1.17 (s, 3H), 1.12 (s, 3H), 1.10-1.06 (m, 2H), 1.04-0.96 (m, 3H), 0.92-0.82 (m, 2H), .067 (s, 3H). **^{13}C NMR** (125 MHz, $CDCl_3$) δ 211.9, 177.7, 171.0, 169.9, 139.4, 137.6, 131.6, 117.5, 58.7, 57.2, 51.1, 49.8, 48.2, 43.8, 40.3, 40.2, 39.8, 39.7, 38.2, 37.4, 37.0, 34.4, 29.2, 28.5, 22.5, 19.5, 19.0, 18.2, 13.9. **HRMS** (ESI): *m/z*: calculated for $C_{29}H_{39}NO_5Na$: $[M + Na]^+$ 504.2720, found: 504.2712.

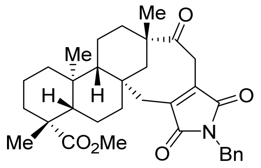

47ad

47ad, White solid, 53.1 mg, yield 99%. **^{1}H NMR** (500 MHz, $CDCl_3$) δ 7.32-7.25 (m, 5H), 4.69 (*A*B, *J* = 15.0 Hz, 1H), 4.62 (A*B*, *J* = 14.5 Hz, 1H), 3.86 (dd, *J* = 18.5, 2.0 Hz, 1H), 3.67 (s, 3H), 3.18 (dd, *J* = 18.5, 2.0 Hz, 1H), 2.86 (d, *J* = 14.0 Hz, 1H), 2.49 (dq, *J* = 14.0, 3.0 Hz, 1H), 2.18-2.08 (m, 3H), 1.92 (dd, *J* = 15.0, 3.0 Hz, 1H), 1.88-1.79 (m, 2H), 1.75 (d, *J* = 12.5 Hz, 1H), 1.62-1.58 (m, 1H), 1.48-1.40 (m, 2H), 1.34 (dt, *J* = 13.5, 3.5 Hz, 1H), 1.17 (s, 3H), 1.11 (s, 3H), 1.08-1.05 (m, 2H), 1.03-0.97 (m, 3H), .091-0.82 (m, 2H), 0.66 (s, 3H). **^{13}C NMR** (125 MHz, $CDCl_3$) δ 212.1, 177.9, 171.3, 170.3, 139.7, 137.9, 136.5, 128.9, 128.5, 138.0, 59.0, 57.5, 51.4, 50.0, 48.5, 44.1, 42.1, 40.4, 40.1, 39.9, 38.5, 37.6, 37.3, 34.7, 29.5, 28.7, 22.9, 19.8, 19.2, 18.4, 14.2. **HRMS** (ESI): *m/z*: calculated for $C_{33}H_{45}N_2O_5$: $[M + NH_4]^+$ 549.3323, found: 549.3313.

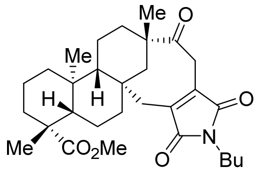

47ae

47ae, White solid, 42.2 mg, yield 85%. **^{1}H NMR** (500 MHz, $CDCl_3$) δ 3.87 (dd, *J* = 18.5, 2.0 Hz, 1H), 3..68 (s, 3H), 3.49 (t, *J* = 7.5 Hz, 2H), 3.18 (dd, *J* = 18.5, 2.0 Hz, 1H), 2.85 (d, *J* = 14.0 Hz, 1), 2.51 (dq, *J* = 13.0, 3.0 Hz, 1H), 2.18-2.08 (m, 3H), 1.93 (dd, *J* = 15.0, 3.0 Hz, 1H), 1.87 (dt, *J* = 14.0, 3.5 Hz, 1H), 1.81 (dq, *J* = 4.5, 2.5 Hz, 1H), 1.76 (d, J = 12.5 Hz, 1H), 1.59-1.53 (m, 3H), 1.50-1.41 (m, 2H), 1.36-1.25 (m, 3H), 1.17 (s, 3H), 1.13 (s, 3H), 1.09-1.06 (m, 2H), 1.03-0.96 (m, 3H), 0.92 (t, *J* = 7.5 Hz, 3H), 0.89-0.82 (m, 2H), 0.68 (s, 3H). **^{13}C NMR** (125 MHz, $CDCl_3$) δ 212.1, 177.8, 171.7, 170.6, 139.2, 137.5, 58.8, 57.3, 51.3, 49.9, 48.3, 43.9, 40.3, 39.9, 39.8, 38.3, 38.2, 37.5, 37.1, 34.5, 30.7, 29.3, 28.6, 22.6, 20.1, 19.6, 19.1, 18.3, 14.0, 13.7. **HRMS** (ESI): *m/z*: calculated for $C_{30}H_{47}N_2O_5$: $[M + NH_4]^+$ 515.3479, found: 515.3475.

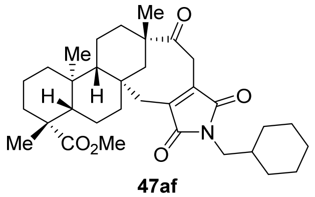

47af

47af, White solid, 50.7 mg, yield 89%. **^{1}H NMR** (500 MHz, $CDCl_3$) δ 3.87 (dd, *J* = 18.5, 2.0 Hz, 1H), 3.67 (s, 3H), 3.32 (d, *J* = 7.0 Hz, 2H), 3.18 (dd, *J* = 18.5, 2.0 Hz, 1H), 2.85 (d, *J* = 14.0 Hz, 1H), 2.50 (dq, *J* = 13.0, 3.0 Hz, 1H), 2.18-2.09 (m, 3H), 1.93 (dd, *J* = 14.5, 2.5 Hz, 1H), 1.86 (dt, *J* = 13.5, 3.5 Hz, 1H), 1.81 (dq, *J* = 14.5, 3.0 Hz, 1H), 1.76-1.59 (m, 9H), 1.50-1.41 (m, 2H), 1.33 (dt, *J* = 13.5, 3.5 Hz, 1H), 1.22-1.15 (m, 5H), 1.12 (s, 3H), 1.09-1.05 (m, 2H), 1.03-0.82 (m, 7H), 0.67 (s, 3H). **^{13}C NMR** (125 MHz, $CDCl_3$) δ 212.3, 177.9, 172.0, 171.0, 139.2, 137.5, 59.0, 57.5, 51.4,

50.1, 48.5, 44.7, 44.1, 40.5, 40.0, 39.9, 38.5, 37.7, 37.3, 37.1, 34.7, 30.9 (2 carbons), 29.5, 28.7, 26.4, 25.9, 25.8, 22.8, 19.8, 19.2, 18.5, 14.2. **HRMS** (ESI): *m/z*: calculated for $C_{33}H_{51}N_2O_5$: [M + $NH_4]^+$ 555.3792, found: 555.3776.

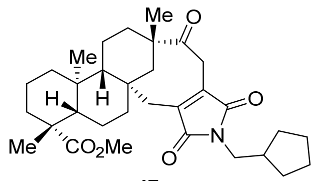

47ag

47ag, White solid, 42.7 mg, yield 84%. **^{1}H NMR** (500 MHz, $CDCl_3$) δ 4.41-4.35 (m, 1H), 3.84 (dd, J = 18.5, 2.0 Hz, 1H), 3.67 (s, 3H), 3.15 (dd, J = 18.5, 2.0 Hz, 1H), 2.83 (d, J = 14.0 Hz, 1H), 2.49 (dq, J = 13.5, 3.0 Hz, 1H), 2.19-2.08 (m, 3H), 1.97-1.79 (m, 9H), 1.75 (d, J = 12.5 Hz, 1H), 1.61-1.56 (m, 3H), 1.49-1.40 (m, 2H), 1.34 (dt, J = 13.0, 3.5 Hz, 1H), 1.17 (s, 3H), 1.11 (s, 3H), 1.08-1.05 (m, 2H), 1.02-0.96 (m, 3H), 0.91-0.82 (m, 2H), 0.67 (s, 3H). **^{13}C NMR** (125 MHz, $CDCl_3$) δ 212.4, 177.9, 171.7, 170.7, 139.2, 137.5, 59.0, 57.5, 51.5, 51.4, 50.1, 48.4, 44.1, 40.4, 40.4, 39.9, 38.5, 37.6, 37.2, 34.7, 29.8, 29.7, 29.5, 28.7, 24.9 (2 carbons), 22.7, 19.8, 19.2, 18.5, 14.2. **HRMS** (ESI): *m/z*: calculated for $C_{31}H_{47}N_2O_5$: [M + $NH_4]^+$ 527.2379, found: 527.3470.

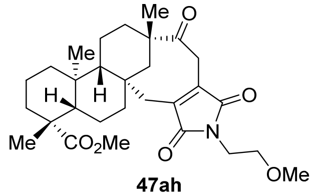

47ah

47ah, White solid, 49.9 mg, yield 99%. **^{1}H NMR** (500 MHz, $CDCl_3$) δ 3.87 (dd, J = 18.0, 1.5 Hz, 1H), 3.71-3.69 (m, 2H), 3.67 (s, 3H), 3.52 (td, J = 5.5, 1.5 Hz, 1H), 3.33 (s, 3H), 3.18 (dd, J = 18.0, 1.5 Hz, 1H), 2.86 (d, J = 14.0 Hz, 1H), 2.50 (dq, J = 13.0, 2.5 Hz, 1H), 2.18-2.08 (m, 3H), 1.93 (dd, J = 14.5, 2.5 Hz, 1H), 1.86 (dt, J = 14.0, 4.0 Hz, 1H), 1.80 (dq, J = 15.0, 3.0 Hz, 1H), 1.75 (d, J = 13.0 Hz, 1H), 1.63-1.58 (m, 1H), 1.49-1.40 (m, 2H), 1.34 (dt, J = 13.5, 3.0 Hz, 1H), 1.16 (s, 3H), 1.11 (s, 3H), 1.08-1.05 (m, 2H), 1.03-0.96 (m, 3H), 0.91-0.82 (m, 2H), 0.67 (s, 3H). **^{13}C NMR** (125 MHz, $CDCl_3$) δ 212.2, 177.9, 171.6, 170.5, 139.6, 137.8, 69.6, 59.0, 58.9, 57.5, 51.4, 50.0, 48.5, 44.1, 40.4, 40.0, 39.9, 38.5, 37.9, 37.6, 37.3, 34.7, 29.5, 28.7, 22.8, 19.7, 19.2, 18.4, 14.2. **HRMS** (ESI): *m/z*: calculated for $C_{29}H_{42}NO_6$: [M + H]$^+$ 500.3007, found: 500.3000. LC-MS (t_R= 0.71 min, λ = 254 nm, purity >99%).

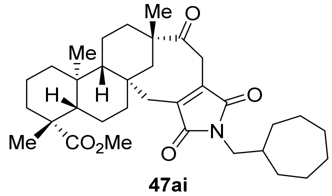

47ai

47ai, Pale yellow oil, 23.1 mg, yield 42%. **^{1}H NMR** (500 MHz, $CDCl_3$) δ 4.14 (tt, J = 10.5, 3.5 Hz, 1H), 3.83 (dd, J = 18.0, 1.5 Hz, 1H), 3.67 (s, 3H), 3.15 (dd, J = 18.5, 1.0 Hz, 1H), 2.82 (d, J = 14.0

Hz, 1H), 2.49 (dq, J = 13.0, 3.0 Hz, 1H), 2.20-2.12 (m, 4H), 2.07 (d, J = 14.5 Hz, 1H), 1.93 (dd, J = 14.5, 2.5 Hz, 1H), 1.88-1.43 (m, 18H), 1.33 (dt, J = 13.5, 3.0 Hz, 1H), 1.16 (s, 3H), 1.11 (s, 3H), 1.08-1.05 (m, 2H), 1.02-0.96 (m, 3H), 0.91-0.82 (m, 2H), 0.66 (s, 3H). **^{13}C NMR** (125 MHz, $CDCl_3$) δ 212.4, 177.9, 171.5, 170.5, 139.0, 137.4, 59.0, 57.5, 52.0, 51.4, 50.1, 48.4, 44.1, 40.5, 40.0, 39.9, 38.5, 37.6, 37.3, 34.7, 32.0, 29.5, 28.7, 26.5 (2 carbons), 26.1, 25.3, 25.2, 22.7, 19.8, 19.2, 18.5, 14.2. **HRMS** (ESI): *m/z*: calculated for $C_{34}H_{50}NO_5$: $[M + H]^+$ 552.3684, found: 552.3673.

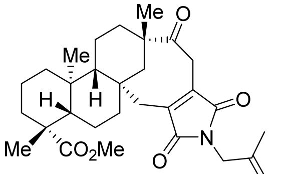

47aj

47aj, White solid, 39.6 mg, yield 81% with 2-methyl allylamine HCl salt and Et_3N. **^{1}H NMR** (500 MHz, $CDCl_3$) δ 4.87-4.86 (m, 1H), 4.73 (s, 1H), 4.03 (s, 2H), 3.89 (dd, J = 18.0, 1.5 Hz, 1H), 3.66 (s, 3H), 3.20 (dd, J = 18.0, 1.5 Hz, 1H), 2.87 (d, J = 14.5 Hz, 1H), 2.51 (dq, J = 13.0, 2.5 Hz, 1H), 2.18-2.08 (m, 3H), 1.92 (dd, J = 14.5, 2.5 Hz, 1H), 1.86 (dt, J = 14.0, 4.0 Hz, 1H), 1.82-1.74 (m, 2H), 1.72 (s, 3H), 1.62-1.58 (m, 1H), 1.50-1.41 (m, 2H), 1.33 (dt, J = 13.0, 3.0 Hz, 1H), 1.16 (s, 3H), 1.12 (s, 3H), 1.10-1.05 (m, 2H), 1.04-0.96 (m, 3H), 0.92-0.82 (m,2H), 0.67 (s, 3H); **^{13}C NMR** (125 MHz, $CDCl_3$) δ 212.2, 177.9, 171.4,170.3, 139.7, 139.6, 137.7,112.0, 59.0, 57.5, 51.4, 50.1, 48.5, 44.1,43.8, 40.4, 40.1, 39.9, 38.5, 37.6, 37.3, 34.7, 29.5, 28.7, 22.8, 20.4, 19.8, 19.2, 18.4, 14.2; **HRMS** (ESI): *m/z*: calculated for $C_{30}H_{41}NO_5Na$: $[M + Na]^+$ 518.2877, found 518.2876.

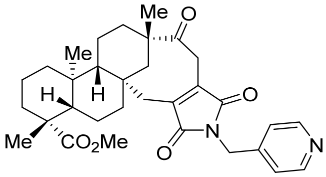

47ak

47ak, White solid, 53.2 mg, yield 99%. **^{1}H NMR** (500 MHz, $CDCl_3$) δ 8.56 (dd, J = 4.5, 1.5 Hz, 2H), 7.19 (dd, J = 4.5, 2.0 Hz, 2H), 4.68 (*A*B, J = 15.5 Hz, 1H), 4.62 (*A*B, J = 15.0 Hz, 1H), 3.88 (dd, J = 18.5, 1.5 Hz, 1H), 3.67 (s, 3H), 3.20 (dd, J = 18.5, 1.5 Hz, 1H), 2.87 (d, J = 14.0 Hz, 1H), 2.50 (dq, J = 13.0, 2.5 Hz, 1H), 2.18-2.08 (m, 3H), 1.92 (dd, J = 14.5, 2.5 Hz, 1H), 1.88-1.79 (m, 2H), 1.75 (d, J = 13.0 Hz, 1H), 1.63-1.59 (m, 1H), 1.49-1.40 (m, 2H), 1.32 (dt, J = 13.5, 3.0 Hz, 1H), 1.17 (s, 3H), 1.12 (s, 3H), 1.11-1.05 (s, 2H), 1.04-0.96 (m, 3H), 0.94-0.82 (m, 2H), 0.66 (s, 3H). **^{13}C NMR** (125 MHz, $CDCl_3$) δ 211.8, 177.8, 171.0, 170.0, 150.5, 145.0, 140.0, 138.2, 122.9, 58.9, 57.5, 51.4, 50.0, 48.5, 44.1, 40.9, 40.4, 40.1, 40.0, 38.4, 37.6, 37.3, 34.6, 29.4, 28.7, 22.9, 19.7, 19.2, 18.4, 14.2. **HRMS** (ESI): *m/z*: calculated for $C_{32}H_{41}N_2O_5$: $[M + H]^+$ 533.3010, found: 533.3010. LC-MS (t_R= 0.46 min, λ = 254 nm, purity 99%).

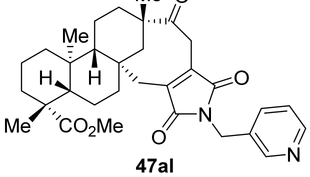

47al

47al, Light yellow solid, 51.0 mg, yield 96%. **^{1}H NMR** (500 MHz, $CDCl_3$) δ 8.62 (s, 1H), 8.54 (d, J = 4.0 Hz, 1H), 7.66 (dt, J = 8.0, 2.0 Hz, 1H), 7.26 (dd, J = 8.0, 4.5 Hz, 1H), 4.69 (d, J = 15.0 Hz, 1H), 4.63 (d, J = 15.0 Hz, 1H), 3.86 (dd, J = 18.5, 1.5 Hz, 1H), 3.67 (s, 3H), 3.17 (dd, J = 18.5, 1.5 Hz, 1H), 2.85 (d, J = 14.5 Hz, 1H), 2.49 (dq, J = 13.5, 3.0 Hz, 1H), 2.18-2.09 (m, 3H), 1.90 (dd, J = 14.5, 3.0 Hz, 1H), 1.87-1.69 (m, 4H), 1.61-1.58 (m, 1H), 1.48-1.39 (m, 2H), 1.31 (dt, J = 13.5, 3.0 Hz, 1H), 1.17 (s, 3H), 1.11 (s, 3H), 1.09-1.05 (m, 2H), 1.03-0.96 (m, 3H), 0.91-0.81 (m, 2H), 0.66 (s, 3H); **^{13}C NMR** (125 MHz, $CDCl_3$) δ 211.9, 177.9, 171.1, 170.0, 150.0, 19.6, 139.9, 18.1, 136.3, 132.1, 123.8, 58.9, 57.4, 51.4, 50.0, 8.5, 44.1, 40.4, 40.1, 39.9, 39.6, 38.4, 37.6, 37.2, 34.6, 29.4, 28.7, 22.9, 19.7, 19.2, 18.4, 14.2; **HRMS** (ESI): *m/z*: calculated for $C_{32}H_{40}N_2O_5Na$: $[M + Na]^+$ 555.2829, found 555.2827. LC-MS (t_R= 0.56 min, λ = 254 nm, purity 98%).

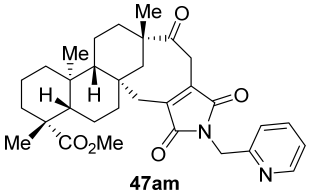

47am

47am, Light yellow solid, 53.2 mg, yield 99%. **^{1}H NMR** (500 MHz, $CDCl_3$) δ 8.52 (ddd, J = 5.0, 2.0, 1.0 Hz, 1H), 7.63 (ddd, J = 8.0, 8.0, 2.0 Hz, 1H), 7.19 (d, J = 8.0 Hz, 1H), 7.16 (ddd, J = 7.5, 5.0, 0.5 Hz, 1H), 4.82 (s, 2H), 3.91 (dd, J = 18.0, 1.5 Hz, 1H), 3.64 (s, 3H), 3.22 (dd, J = 18.0, 1.5 Hz, 1H), 2.89 (d, J = 14.0 Hz, 1H), 2.51 (dq, J = 13.0, 3.0 Hz, 1H), 2.17-2.06 (m, 3H), 1.98 (dd, J = 15.0, 3.0 Hz, 1H), 1.86 (dt, J = 14.0, 3.5 Hz, 1H), 1.81-1.74 (m, 2H), 1.62-1.60 (m, 1H), 1.50-1.37 (m, 3H), 1.15 (s, 3H), 1.13 (s, 3H), 1.11-1.03 (m, 2H), 1.01-0.95 (m, 3H), 0.92-0.82 (m, 2H), 0.66 (s, 3H); **^{13}C NMR** (125 MHz, $CDCl_3$) δ 212.2, 177.9, 171.6, 170.5, 155.5, 149.9, 139.9, 138.1, 136.9, 122.7, 121.5, 59.0, 57.5, 51.4, 50.0, 48.5, 44.0, 43.4, 40.4, 40.1, 39.8, 38.5, 37.6, 37.4, 34.7, 29.5, 28.7, 22.9, 19.8, 19.2, 18.5, 14.1; **HRMS** (ESI): *m/z*: calculated for $C_{32}H_{40}N_2O_5Na$: $[M + Na]^+$ 555.2829, found 555.2825. LC-MS (t_R= 0.64 min, λ = 254 nm, purity 99%).

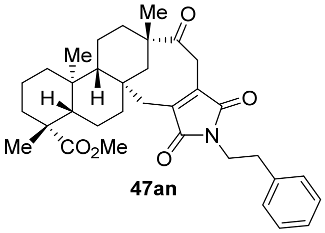

47an

47an, White solid, 51.8 mg, yield 95%. **^{1}H NMR** (500 MHz, $CDCl_3$) δ 7.31-7.27 (m, 2H), 7.23-7.19 (m, 3H), 3.82 (dd, J = 18.5, 1.5 Hz, 1H), 3.74 (ddd, J = 8.5, 7.0, 4.0 Hz, 1H), 3.68 (s, 3H), 3.14 (dd, J = 18.5, 1.5 Hz, 1H), 2.89 (t, J = 8.0 Hz, 2H), 2.82 (d, J = 14.0 Hz, 1H), 2.50 (dq, J = 13.5, 3.0 Hz, 1H), 2.19-2.06 (m, 3H), 1.90-1.74 (m, 4H), 1.61-1.58 (m, 1H), 1.48-1.40 (m, 2H), 1.77 (dt, J = 13.5, 3.0 Hz, 1H), 1.17 (s, 3H), 1.11 (s, 3H), 1.08-0.96 (m, 5H), 0.89-0.82 (m, 2H), 0.67 (s, 3H); **^{13}C NMR** (125 MHz, $CDCl_3$) δ 212.2, 177.9, 171.5, 170.4, 139.4, 138.1, 137.6, 129.1, 128.8, 126.9, 59.0, 57.5, 51.4, 50.0, 48.5, 44.1, 40.4, 40.0, 39.9, 39.7, 38.5, 37.6, 37.2, 34.8, 34.7, 29.5, 28.7, 22.7, 19.7, 19.2, 18.4, 14.2; **HRMS** (ESI): *m/z*: calculated for $C_{34}H_{43}NO_5Na$: $[M + Na]^+$ 568.3033, found 568.3045.

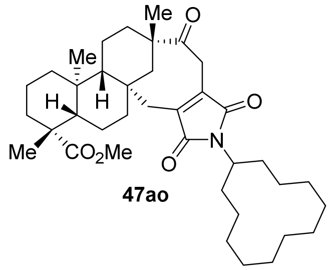

47ao

47ao, Light brown oil, 22.2 mg, yield 40%. **^{1}H NMR** (500 MHz, $CDCl_3$) δ 4.24-4.18 (m, 1H), 3.84 (dd, *J* = 18.0, 1.5 Hz, 1H), 3.68 (s, 3H), 3.16 (dd, *J* = 18.5, 1.0 Hz, 1H), 2.83 (d, *J* = 14.0 Hz, 1H), 2.50 (dq, *J* = 13.0, 2.5 Hz, 1H), 2.18-2.06 (m, 3H), 2.02-1.97 (m, 2H), 1.93 (dd, *J* = 14.5, 2.5 Hz, 1H), 1.88-1.74 (m, 3H), 1.62-1.59 (m, 3H), 1.50-1.25 (m, 21H), 1.17 (s, 3H), 1.12 (s, 3H), 1.06 (d, *J* = 14.5 Hz, 2H), 1.02-0.97 (m, 3H), 0.90-0.82 (m, 2H), 0.67 (s, 3H);); **^{13}C NMR** (125 MHz, $CDCl_3$) δ 212.5, 177.9, 171.9, 171.0, 139.0, 137.3, 59.1, 57.5, 51.5, 50.2, 48.5, 47.4, 44.1, 40.5, 40.0, 38.5, 37.6, 37.3, 34.7, 29.5, 28.7, 28.4, 24.4, 24.3, 24.2 (2 carbons), 22.9, 22.8 (2 carbons), 22.7, 19.8, 19.3, 18.5, 14.2; **HRMS** (ESI): *m/z*: calculated for $C_{38}H_{57}NO_5Na$: $[M + Na]^+$ 580.3972, found 580.3970.

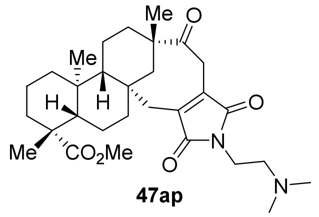

47ap

47ap, Pale yellow solid, 49.2 mg, yield 96%. **^{1}H NMR** (500 MHz, $CDCl_3$) δ 3.86 (dd, *J* = 18.5, 1.5 Hz, 1H), 3.67 (s, 3H), 3.63-3.56 (m, 2H), 3.17 (dd, *J* = 18.5, 1.5 Hz, 1H), 2.85 (d, *J* = 14.0 Hz, 1H), 2.51-2.41 (m, 3H), 2.24 (s, 6H), 2.18-2.07 (m, 3H), 1.93 (dd, *J* = 14.5, 2.5 Hz, 1H), 1.88-1.79 (m, 2H), 1.75 (d, *J* = 13.0 Hz, 1H), 1.61-1.58 (m, 1H), 1.49-1.40 (m, 2H), 1.34 (dt, *J* = 13.5, 3.0 Hz, 1H), 1.16 (s, 3H), 1.11 (s, 3H), 1.08-1.05 (m, 2H), 1.02-0.97 (m, 3H), 0.90-0.82 (m, 2H), 0.67 (s, 3H); **^{13}C NMR** (125 MHz, $CDCl_3$) δ 212.2, 177.9, 171.7, 170.6, 139.5, 137.7, 59.0, 57.5, 57.4, 51.4, 50.0, 48.5, 46.7, 44.1, 40.4, 40.0, 39.9, 38.5, 37.6, 37.3, 36.5, 34.7, 29.5, 28.7, 22.8, 19.7, 19.2, 18.4, 14.2; **HRMS** (ESI): *m/z*: calculated for $C_{30}H_{45}N_2O_5$: $[M + H]^+$ 513.3323, found 513.3340.

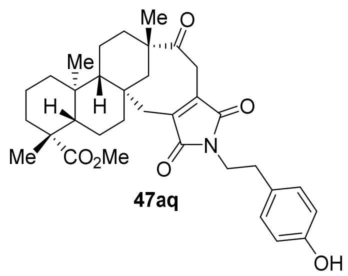

47aq

47aq, White solid, 56.1 mg, yield 99%. **^{1}H NMR** (500 MHz, $CDCl_3$) δ 7.05 (d, *J* = 8.5 Hz, 2H), 6.75 (d, *J* = 8.5 Hz, 2H), 5.09 (s, 1H), 3.82 (dd, *J* = 18.0, 1.5 Hz, 1H), 3.71-3.68 (m, 5H), 3.14 (dd, *J* = 18.0, 1.5 Hz, 1H), 2.83-2.80 (m, 3H), 2.49 (dq, *J* = 13.5, 2.5 Hz, 1H), 2.18-2.06 (m, 3H), 1.89-1.74 (m, 4H), 1.62-1.58 (m, 1H), 1.46-1.43 (m, 2H), 1.27 (dt, *J* = 13.5, 3.0 Hz, 1H), 1.17 (s, 3H), 1.11 (s, 3H), 1.08-0.97 (m, 5H), 0.90-0.82 (m, 2H), 0.66 (s, 3H); **^{13}C NMR** (125 MHz, $CDCl_3$) δ

212.1, 177.9, 171.4, 170.3, 154.5, 139.3, 137.5, 130.1, 130.0, 115.5, 58.8, 57.3, 51.3, 49.9, 48.3, 43.9, 40.3, 39.9, 39.8, 39.7, 38.3, 37.5, 37.1, 34.6, 33.7, 29.4, 28.6, 22.6, 19.6, 19.1, 18.3, 14.1; **HRMS** (ESI): *m/z*: calculated for $C_{34}H_{43}NO_6Na$: $[M + Na]^+$ 584.2983, found 584.2980. LC-MS (t_R= 0.73 min, λ = 254 nm, purity 99%).

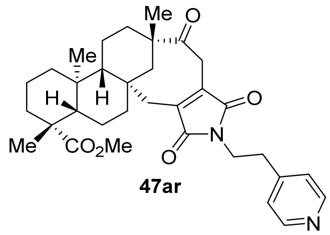

47ar

47ar, White solid, 51.8 mg, yield 95%. **^{1}H NMR** (500 MHz, $CDCl_3$) δ 8.52 (d, *J* = 5.0 Hz, 2H), 7.13 (dd, *J* = 4.5, 2.0 Hz, 2H), 3.82 (dd, *J* = 18.5, 1.5 Hz, 1H), 3.77 (td, *J* = 7.5, 2.0 Hz, 2H), 3.68 (s, 3H), 3.14 (dd, *J* = 18.0, 1.0 Hz, 1H), 2.92 (t, *J* = 7.5 Hz, 2H), 2.80 (d, *J* = 14.0 Hz, 1H), 2.49 (dq, *J* = 13.5, 2.5 Hz, 1H), 2.16 (d, *J* = 13.5 Hz, 1H), 2.11-2.06 (m, 2H), 1.89-1.75 (m, 4H), 1.61-1.58 (m, 1H), 1.46-1.38 (m, 2H), 1.21 (dt, *J* = 13.5, 3.0 Hz, 1H), 1.17 (s, 3H), 1.11 (s, 3H), 1.07-0.96 (m, 5H), 0.86 (qd, *J* = 13.0, 3.5 Hz, 2H), 0.66 (s, 3H); **^{13}C NMR** (125 MHz, $CDCl_3$) δ 212.0, 177.9, 171.3, 170.2, 150.2, 147.0, 139.6, 137.7, 124.4, 58.9, 57.4, 51.4, 50.0, 48.5, 44.1, 40.4, 40.0, 39.9, 38.5, 38.4, 37.6, 37.2, 34.6, 33.9, 29.5, 28.7, 22.8, 19.7, 19.2, 18.4, 14.2; **HRMS** (ESI): *m/z*: calculated for $C_{33}H_{43}N_2O_5$: $[M + H]^+$ 547.3166, found 547.3172. LC-MS (t_R= 0.37 min, λ = 254 nm, purity >99%).

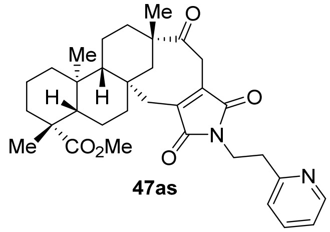

47as

47as, White solid, 40.9 mg, yield 95%. **^{1}H NMR** (500 MHz, $CDCl_3$) δ 8.51 (ddd, *J* = 5.0, 1.0, 1.0 Hz, 1H), 5.89 (ddd, *J* = 7.5, 7.5, 2.0 Hz, 1H), 7.14-7.11 (m, 2H), 3.95-3.86 (m, 1H), 3.83 (dd, *J* = 18.0, 1.5 Hz, 1H), 3.67 (s, 3H), 3.13 (dd, *J* = 18.5, 1.5 Hz, 1H), 3.07 (t, *J* = 7.0 Hz, 2H), 2.81 (d, *J* = 14.5 Hz, 1H), 2.49 (dq, *J* = 13.5, 3.0 Hz, 1H), 2.16 (d, *J* = 13.5 Hz, 1H), 2.11-2.05 (m, 2H), 1.92-1.73 (m, 4H), 1.61-1.58 (m, 1H), 1.48-1.39 (m, 2H), 1.29 (dt, *J* = 13.5, 3.0 Hz, 1H), 1.16 (s, 3H), 1.10 (s, 3H), 1.07 (s, 1H), 1.04 (s, 1H), 0.99 (td, *J* = 13.5, 4.5 Hz, 2H), 0.88-0.81 (m, 2H), 0.66 (s, 3H); **^{13}C NMR** (125 MHz, $CDCl_3$) δ 212.7, 177.9, 171.4, 170.3, 158.5, 149.7, 139.4, 137.6, 136.6, 123.4, 121.8, 59.0, 57.5, 51.4, 50.0, 48.5, 44.1, 40.4, 40.0, 39.8, 38.5, 38.3, 37.6, 37.2, 36.8, 34.7, 29.5, 28.7, 22.7, 19.7, 19.2, 18.4, 14.2; **HRMS** (ESI): *m/z*: calculated for $C_{33}H_{43}N_2O_5$: $[M + H]^+$ 547.3166, found 547.3184. LC-MS (t_R= 0.50 min, λ = 210 nm, purity >99%).

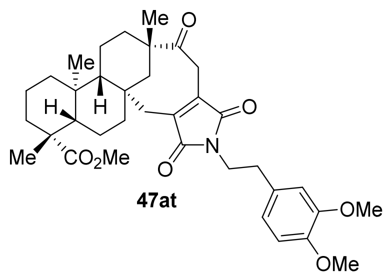

47at

47at, White solid, 59.8 mg, yield 99%. **^{1}H NMR** (500 MHz, $CDCl_3$) δ 6.78 (d, *J* = 8.0 Hz, 1H), 6.74-6.71 (m, 2H), 3.87 (s, 3H), 3.85-3.81 (m, 4H), 3.77-3.70 (m, 2H), 3.68 (s, 3H), 3.14 (dd, *J* = 18.5, 1.5 Hz, 1H), 2.89-2.79 (m, 3H), 2.49 (dq, *J* = 13.0, 3.0 Hz, 1H), 2.17 (d, *J* = 14.0 Hz, 1H), 2.12-2.05 (m, 2H), 1.90-1.73 (m, 4H), 1.60-1.58 (m, 1H), 1.45-1.39 (m, 2H), 1.23 (dt, *J* = 13.5, 3.0 Hz, 1H), 1.17 (s, 3H), 1.11 (s, 3H), 1.07-0.96 (m, 5H), 0.87-0.81 (m, 2H), 0.66 (s, 3H); **^{13}C NMR** (125 MHz, $CDCl_3$) δ 212.1, 177.9, 171.5, 170.5, 149.1, 148.0, 139.4, 137.6, 130.6, 121.1, 112.1, 111.4, 58.9, 57.5, 56.1 (2 carbons), 51.4, 50.0, 48.4, 44.1, 40.4, 40.0, 39.8, 38.4, 37.6, 37.2, 34.7, 34.3, 29.5, 28.7, 22.7, 19.7, 19.2, 18.5, 14.2; **HRMS** (ESI): *m/z*: calculated for $C_{36}H_{47}NO_7Na$: $[M + Na]^+$ 628.3245, found 628.3252.

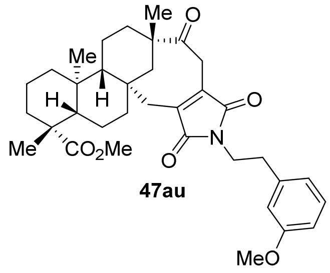

47au

47au, White solid, 56.9 mg, yield 99%. **^{1}H NMR** (500 MHz, $CDCl_3$) δ 7.20 (t, *J* = 7.5 Hz, 1H), 6.79 (d, *J* = 5.0 Hz, 1H), 6.77-6.74 (m, 2H), 3.83 (dd, *J* = 18.0, 1.0 Hz, 1H), 3.80 (s, 3H), 3.77-3.70 (m, 2H), 3.68 (s, 3H), 3.14 (dd, *J* = 18.0, 1.0 Hz, 1H), 2.87 (t, *J* = 8.0 Hz, 1H), 2.82 (d, *J* = 14.0 Hz, 1H), 2.50 (dt, *J* = 13.5, 3.0 Hz, 1H), 2.18-2.06 (m, 3H), 1.90-1.84 (m, 2H), 1.82-1.74 (m, 2H), 1.61-1.59 (m, 1H), 1.26 (dt, *J* = 13.5, 3.0 Hz, 1H), 1.17 (s, 3H), 1.11 (s, 3H), 1.08-1.01 (m, 3H), 0.99-0.96 (m, 2H), 0.88-0.82 (m, 2H), 0.67 (s, 3H); **^{13}C NMR** (125 MHz, $CDCl_3$) δ 212.2, 177.9, 171.5, 170.4, 159.9, 139.7, 139.4, 137.7, 129.8, 121.4, 114.6, 112.4, 59.0, 57.5, 55.4, 51.4, 50.0, 48.5, 44.1, 40.4, 40.0, 39.9, 39.6, 38.5, 37.6, 37.2, 34.8, 34.7, 39.5, 28.7, 22.7, 19.7, 19.2, 18.4, 14.2; **HRMS** (ESI): *m/z*: calculated for $C_{35}H_{45}NO_6Na$: $[M + Na]^+$ 598.3139, found 598.3133.

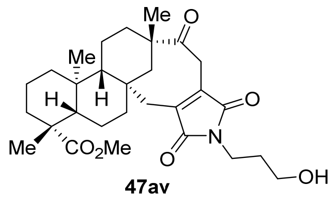

47av

47av, White solid, 49.4 mg, yield 99%. **^{1}H NMR** (500 MHz, $CDCl_3$) δ 3.87 (dd, *J* = 18.5, 1.5 Hz, 1H), 3.67-3.65 (m, 5H), 3.57 (dt, *J* = 5.5, 5.5 Hz, 2H), 3.18 (dd, *J* = 18.5, 1.5 Hz, 1H), 2.85 (d, *J* = 14.0 Hz, 1H), 2.50 (dq, *J* = 13.0, 3.0 Hz, 1H), 2.30 (t, *J* = 6.0 Hz, 1H), 2.18-2.08 (m, 3H), 1.92 (dd, *J* = 15.0, 3.0 Hz, 1H), 1.88-1.74 (m, 5H), 1.62-1.58 (m, 1H), 1.49-1.40 (m, 2H), 1.32 (dt, *J* = 13.5,

3.0 Hz, 1H), 1.16 (s, 3H), 1.12 (s, 3H), 1.08 (d, *J* = 14.5 Hz, 1H), 1.06 (dd, *J* = 12.5, 2.5 Hz, 1H), 1.03-0.96 (m, 3H), 0.92-0.82 (m, 2H), 0.67 (s, 3H); **^{13}C NMR** (125 MHz, $CDCl_3$) δ 212.0, 177.9, 172.3, 171.0, 139.7, 137.9, 59.3, 58.9, 57.5, 51.4, 50.0, 48.5, 44.1, 40.4, 40.1, 39.9, 38.4, 37.6, 37.2, 34.7, 34.6, 31.5, 29.5, 28.7, 22.8, 19.7, 19.2, 18.4, 14.2; **HRMS** (ESI): *m/z*: calculated for $C_{29}H_{41}NO_6Na$: $[M + Na]^+$ 522.2826, found 522.2829. LC-MS (t_R= 0.57 min, λ = 254 nm, purity >99%).

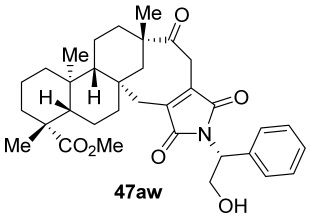

47aw

47aw, White solid, 55.6 mg, yield 99%. **^{1}H NMR** (500 MHz, $CDCl_3$) δ 7.38-7.29 (m, 5H), 5.24 (dd, *J* = 9.0, 5.0 Hz, 1H), 4.50 (ddd, *J* = 11.2, 9.0, 8.0 Hz, 1H), 4.11 (dt, *J* = 11.5, 5.0 Hz, 1H), 3.86 (dd, *J* = 18.5, 1.5 Hz, 1H), 3.67 (s, 3H), 3.16 (dd, *J* = 18.5, 1.5 Hz, 1H), 2.87 (d, *J* = 14.5 Hz, 1H), 2.49 (dq, *J* = 13.5, 3.0 Hz, 1H), 2.31 (dd, *J* = 8.0, 5.0 Hz, 1H), 2.18-2.09 (m, 3H), 1.93 (dd, *J* = 14.5, 2.5 Hz, 1H), 1.90-1.74 (m, 3H), 1.61-1.59 (m, 1H), 1.49-1.40 (m, 2H), 1.32 (dt, *J* = 13.5, 3.0 Hz, 1H), 1.17 (s, 3H), 1.11 (s, 3H), 1.08 (d, *J* = 14.5 Hz, 1H), 1.06 (dd, *J* = 13.0, 2.5 Hz, 1H), 1.03-0.97 (m, 3H), 0.91-0.82 (m, 2H), 0.66 (s, 3H); **^{13}C NMR** (125 MHz, $CDCl_3$) δ 212.1, 177.9, 172.0, 170.9, 139.7, 137.8, 136.9, 129.0, 128.4, 127.9, 62.4, 59.0, 57.8, 57.5, 51.5, 50.0, 48.5, 44.1, 40.4, 40.1, 39.9, 38.4, 37.6, 37.3, 34.7, 29.4, 28.7, 22.9, 19.8, 19.2, 18.4, 14.2; **HRMS** (ESI): *m/z*: calculated for $C_{34}H_{43}NO_6Na$: $[M + Na]^+$ 584.2983, found 584.2989. LC-MS (t_R= 0.91 min, λ = 254 nm, purity >99%).

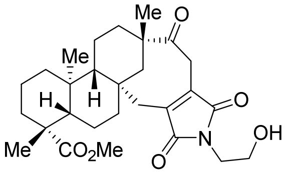

47ax

47ax, White solid, 47.9 mg, yield 99%. **^{1}H NMR** (500 MHz, $CDCl_3$) δ 3.87 (dd, *J* = 18.5, 1.5 Hz, 1H), 3.80-3.77 (m, 2H), 3.72-3.70 (m, 2H), 3.67 (s, 3H), 3.18 (dd, *J* = 18.5, 1.5 Hz, 1H), 2.87 (d, *J* = 14.0 Hz, 1H), 2.50 (dq, *J* = 13.5, 3.5 Hz, 1H), 2.18-2.08 (m, 4H), 1.94 (dd, *J* = 14.5, 2.5 Hz, 1H), 1.86 (qt, *J* = 14.0, 4.0 Hz, 1H), 1.80 (dq, *J* = 15.0, 3.5 Hz, 1H), 1.75 (d, *J* = 13.5 Hz, 1H), 1.62-1.159 (m, 1H), 1.49-1.40 (m, 2H), 1.34 (dt, *J* = 13.5, 3.5 Hz, 1H), 1.17 (s, 3H), 1.12 (s, 3H), 1.09 (dd, *J* = 14.5, 1.5 Hz, 1H), 1.07 (dd, *J* = 13.0, 2.5 Hz, 1H), 1.04-0.96 (m, 3H), 0.93-0.82 (m, 3H), 0.67 (m, 3H); **^{13}C NMR** (125 MHz, $CDCl_3$) δ 212.0, 177.9, 172.1, 171.0, 139.8, 138.0, 61.3, 59.0, 57.5, 51.4, 50.0, 48.5, 44.1, 41.4, 40.4, 40.1, 39.9, 38.4, 37.6, 37.3, 34.6, 29.5, 28.7, 22.9, 19.7, 19.2, 18.4, 14.2; **HRMS** (ESI): *m/z*: calculated for $C_{28}H_{40}NO_6$: $[M + H]^+$ 486.2850, found 486.2853. LC-MS (t_R= 0.51 min, λ = 254 nm, purity 97%).

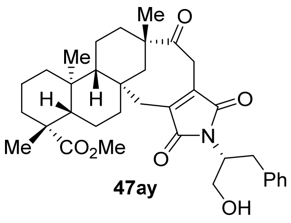

47ay

47ay, White solid, 51.7 mg, yield 90%. **^{1}H NMR** (500 MHz, $CDCl_3$) δ 7.27-7.24 (m, 2H), 7.20-7.16 (m, 3H), 4.42 (dtd, *J* = 9.0, 7.0, 3.5 Hz, 1H), 4.01-3.95 (m, 1H), 3.85 (dt, *J* = 12.0, 3.5 Hz, 1H), 3.89 (dd, *J* = 18.5, 1.5 Hz, 1H), 3.66 (s, 3H), 3.14-3.05 (m, 3H), 2.73 (d, *J* = 14.0 Hz, 1H), 2.59 (dd, *J* = 8.5, 1.5 Hz, 1H), 2.47 (dq, *J* = 13.5, 3.0 Hz, 1H), 2.16 (d, *J* = 13.5 Hz, 1H), 2.07-2.00 (m, 2H), 1.84 (qt, *J* = 13.5, 3.5 Hz, 1H), 1.74-1.70 (m, 3H), 1.59-1.56 (m, 1H), 1.45-1.36 (m, 2H), 1.17 (s, 3H), 1.10 (s, 3H), 1.05-0.92 (m, 6H), 0.83 (td, *J* = 13.5, 4.0 Hz, 1H), 0.74 (t, *J* = 13.5 Hz, 1H), 0.63 (s, 3H); **^{13}C NMR** (125 MHz, $CDCl_3$) δ 212.0, 177.9, 171.8, 171.2, 139.3, 137.6 (2 carbons), 129.3, 128.8, 127.0, 63.2, 58.9, 57.4, 55.5, 51.4, 49.9, 48.4, 44.1, 40.4, 39.9, 39.7, 38.5, 37.6, 37.1, 34.9, 34.7, 29.5, 28.8, 22.8, 19.7, 19.2, 18.4, 14.2; **HRMS** (ESI): *m/z*: calculated for $C_{35}H_{46}NO_6$: $[M + H]^+$ 576.3320, found 576.3330. LC-MS (t_R= 0.94 min, λ = 254 nm, purity >99%).

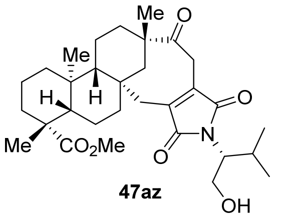

47az

47az, White solid, 37.9 mg, yield 72%. **^{1}H NMR** (500 MHz, $CDCl_3$) δ 4.03 (ddd, *J* = 12.0, 9.0, 7.0 Hz, 1H), 3.87 (dd, *J* = 18.5, 1.5 Hz, 1H), 3.82-3.74 (m, 2H), 3.67 (s, 3H), 3.18 (dd, *J* = 18.5, 1.5 Hz, 1H), 2.85 (d, *J* = 14.0 Hz, 1H), 2.72 (dd, *J* = 9.5, 3.0 Hz, 1H), 2.50 (dq, *J* = 13.5, 3.0 Hz, 1H), 2.37-2.30 (m, 1H), 2.19-2.08 (m, 3H), 1.92 (dd, *J* = 14.5, 3.0 Hz, 1H), 1.88-1.7 (m, 3H), 1.62-1.59 (m, 1H), 1.49-1.41 (m, 2H), 1.31 (dt, *J* = 13.0, 3.5 Hz, 1H), 1.17 (s, 3H), 1.12 (s, 3H), 1.10-0.96 (m, 8H), 0.92-0.82 (m, 2H), 0.80 (d, *J* = 7.0 Hz, 3H), 0.67 (s, 3H); **^{13}C NMR** (125 MHz, $CDCl_3$) δ 212.1, 177.9, 172.6, 171.5, 139.4, 137.5, 62.4, 60.3, 58.9, 57.5, 51.4, 50.0, 48.5, 44.1, 40.4, 40.1, 39.9, 38.4, 37.6, 37.3, 34.7, 29.5, 28.7, 27.3, 22.8, 20.2, 20.0, 19.8, 19.2, 18.4, 14.2; **HRMS** (ESI): *m/z*: calculated for $C_{31}H_{45}NO_6Na$: $[M + Na]^+$ 550.3139, found 550.3147. LC-MS (t_R= 0.90 min, λ = 254 nm, purity >99%).

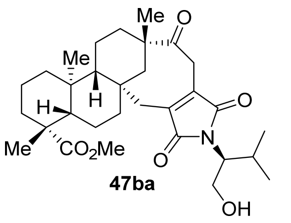

47ba

47ba, White solid, 44.7 mg, yield 85%. **^{1}H NMR** (500 MHz, $CDCl_3$) δ 4.02 (ddd, *J* = 12.0, 9.0, 7.0 Hz, 1H), 3.87 (dd, *J* = 18.5, 1.5 Hz, 1H), 3.81 (dt, *J* = 12.0, 3.0 Hz, 1H), 3.75 (ddd, *J* = 10.5, 7.5, 3.0 Hz, 1H), 3.67 (s, 3H), 3.17 (dd, *J* = 18.5, 1.0 Hz, 1H), 2.86 (d, *J* = 14.0 Hz, 1H), 2.65 (dd, *J* = 9.0, 3.0 Hz, 1H), 2.50 (dq, *J* = 13.0, 3.0 Hz, 1H), 2.36 (ddt, *J* = 13.0, 10.5, 6.5 Hz, 1H), 2.18-2.07

(m, 3H), 1.92 (dd, J = 15.0, 3.0 Hz, 1H), 1.8801.74 (m, 3H), 1.63-1.58 (m, 1H), 1.50-1.1 (m, 2H), 1.31 (dt, J = 13.5, 3.0 Hz, 1H), 1.16 (s, 3H), 1.13 (s, 3H), 1.10-0.97 (m, 8H), 0.92-0.83 (m, 3H), 0.80 (d, J = 7.0 Hz, 3H), 0.67 (s, 3H); **^{13}C NMR** (125 MHz, $CDCl_3$) δ 212.1, 177.9, 172.5, 171.5, 139.4, 137.5, 62.5, 60.4, 58.9, 57.5, 51.4, 50.1, 48.5, 44.1, 40.4, 40.1, 39.9, 38.4, 37.6, 37.3, 34.6, 29.6, 28.7, 27.1, 22.8, 20.2, 20.0, 19.7, 19.2, 18.4, 14.2; **HRMS** (ESI): *m/z*: calculated for $C_{31}H_{45}NO_6Na$: $[M + Na]^+$ 550.3139, found 550.3150. LC-MS (t_R= 0.86 min, λ = 254 nm, purity 97%).

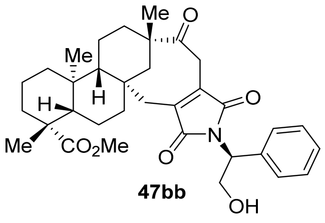

47bb

47bb White solid, 53.8 mg, yield 96%. **^{1}H NMR** (500 MHz, $CDCl_3$) δ 7.36-7.28 (m, 5H), 5.21 (dd, J = 9.0, 5.0 Hz, 1H), 4.49 (dt, J = 12.0, 8.0 Hz, 1H), 4.10 (dt, J = 12.0, 5.0 Hz, 1H), 3.86 (dd, J = 18.5, 1.5 Hz, 1H), 3.67 (s, 3H), 3.18 (dd, J = 18.5, 1.5 Hz, 1H), 2.86 (d, J = 14.0 Hz, 1H), 2.50 (dq, J = 13.5, 3.0 Hz, 1H), 2.42 (dd, J = 8.0, 5.0 Hz, 1H), 2.18-2.09 (m, 3H), 1.93 (dd, J = 14.5, 2.5 Hz, 1H), 1.90-1.81 (m, 2H), 1.75 (d, J = 13.0 Hz, 1H), 1.62-1.59 (m, 1H), 1.49-1.40 (m, 2H), 1.35 (dt, J = 13.5, 3.0 Hz, 1H), 1.17 (s, 3H), 1.11 (s, 3H), 1.08 (d, J = 14.5 Hz, 1H), 1.07 (dd, J = 12.5, 2.5 Hz, 1H), 1.03-0.97 (m, 3H), 0.93-0.82 (m, 2H), 0.66 (s, 3H); **^{13}C NMR** (125 MHz, $CDCl_3$) δ 212.0, 177.9, 172.1, 171.0, 139.7, 138.0, 137.2, 129.0, 128.4, 127.7, 62.8, 59.0, 58.4, 57.5, 51.5, 50.0, 48.5, 44.1, 40.4, 40.1, 39.9, 38.4, 37.6, 37.3, 34.6, 29.4, 28.8, 22.9, 19.8, 19.2, 18.4, 14.2; **HRMS** (ESI): *m/z*: calculated for $C_{34}H_{43}NO_6Na$: $[M + Na]^+$ 584.2983, found 584.2989. LC-MS (t_R= 0.88 min, λ = 254 nm, purity >99%).

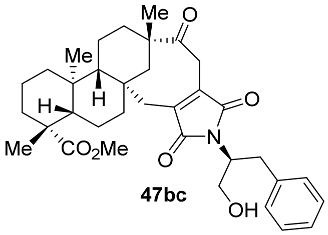

47bc

47bc, White solid, 56.9 mg, yield 99%. **^{1}H NMR** (500 MHz, $CDCl_3$) δ 7.28-7.25 (m, 2H), 7.22-7.19 (m, 1H), 7.17-7.16 (m, 2H), 4.43-4.38 (m, 1H), 3.99-3.93 (m 1H), 3.85 (d, J = 12.0 Hz, 1H), 3.73-3.69 (m, 4H), 3.14-3.06 (m, 3H), 2.79 (d, J = 14.5 Hz, 1H), 2.58 (d, J = 5.5 Hz, 1H), 2.48 (dq, J = 13.5, 3.0 Hz, 1H), 2.19-2.09 (m, 2H), 2.04 (d, J = 14.0 Hz, 1H), 1.90-1.73 (m, 4H), 1.60-1.57 (m, 1H), 1.46-1.37 (m, 2H), 1.34 (dt, J = 13.5, 3.0 Hz, 1H), 1.19 (s, 3H), 1.08 (s, 3H), 1.06-0.95 (m, 5H), 0.92-0.82 (m, 2H), 0.66 (s, 3H); **^{13}C NMR** (125 MHz, $CDCl_3$) δ 212.0, 177.9, 172.3, 170.7, 139.4, 137.6, 137.5, 129.4, 128.7, 126.9, 63.2, 59.0, 57.5, 55.6, 51.5, 49.9, 48.4, 44.1, 40.4, 40.0, 39.9, 38.4, 37.6, 37.2, 34.9, 34.7, 29.4, 28.8, 22.7, 19.8, 19.2, 18.4, 14.2; **HRMS** (ESI): *m/z*: calculated for $C_{35}H_{46}NO_6$: $[M + H]^+$ 576.3320, found 576.3329. LC-MS (t_R= 0.94 min, λ = 254 nm, purity 98%).

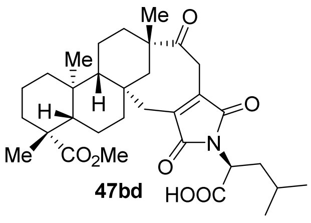

47bd

47bd, Light brown solid, 45.1 mg, yield 86%. **^{1}H NMR** (500 MHz, $CDCl_3$) δ 11.02 (brs, 1H), 4.77 (dd, *J* = 11.5, 4.5 Hz, 1H), 3.89 (dd, *J* = 13.5, 1.5 Hz, 1H), 3.68 (s, 3H), 3.18 (dd, *J* = 13.5, 1.5 Hz, 1H), 2.86 (d, *J* = 14.5 Hz, 1H), 2.50 (dq, *J* = 13.5,3.0 Hz, 1H), 2.25 (ddd, *J* = 14.5, 11.5, 4.5 Hz, 1H), 2.18-2.07 (m, 3H), 1.94 (dd, *J* = 14.5, 2.5 Hz, 1H), 1.90-1.74 (m, 4H), 1.62-1.60 (m, 1H), 1.49-1.41 (m, 3H), 1.32 (dt, *J* = 13.5, 3.0 Hz, 1H), 1.16 (s, 3H), 1.13 (s, 3H), 1.11-0.82 (m, 13H), 0.67 (s, 3H); **^{13}C NMR** (125 MHz, $CDCl_3$) δ 212.1, 178.0, 175.6, 170.9, 169.9, 139.9, 138.1, 59.0, 57.5, 51.5, 50.9, 50.0, 48.5, 44.1, 40.4, 40.1, 39.9, 38.4, 37.6, 37.3, 37.1, 34.6, 29.4, 28.7, 25.3, 23.2, 22.9, 21.2, 19.7, 19.2, 18.4, 14.2; **HRMS** (ESI): *m/z*: calculated for $C_{32}H_{45}NO_7Na$: $[M + Na]^+$ 578.3088, found 578.3098.

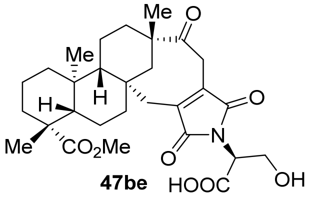

47be

47be, White solid, 41.2 mg, yield 78%. **^{1}H NMR** (500 MHz, $CDCl_3$) δ 6.62 (brs, 2H), 4.86 (dd, *J* = 5.5, 3.5 Hz, 1H), 4.25 (dd, *J* = 12.5, 5.5 Hz, 1H), 4.11 (dd, *J* = 12.5, 3.5 Hz, 1H), 3.90 (d, *J* = 18.5 Hz, 1H), 3.66 (s, 3H), 3.20 (d, *J* = 18.0 Hz, 1H), 2.87 (d, *J* = 14.0 Hz, 1H), 2.50 (dq, *J* = 13.5, 3.5 Hz, 1H), 2.17-2.04 (m, 3H), 1.96 (dd, *J* = 14.5, 2.5 Hz, 1H), 1.89-1.73 (m, 3H), 1.61 (d, *J* = 9.5 Hz, 1H), 1.48-1.40 (m, 2H), 1.33 (dt, *J* = 13.0, 3.0 Hz, 1H), 1.16 (s, 3H), 1.13 (s, 3H), 1.11-0.96 (m, 5H), 0.93-0.82 (m, 2H), 0.66 (s, 3H); **^{13}C NMR** (125 MHz, $CDCl_3$) δ 211.9, 178.1, 171.5, 171.1, 170.4, 140.4, 138.6, 61.1, 58.9, 57.5, 55.3, 51.6, 49.9, 48.5, 44.1, 40.4, 40.2, 39.9, 38.3, 37.6, 37.2, 34.6, 29.4, 28.8, 23.0, 19.7, 19.2, 18.4, 14.2; **HRMS** (ESI): *m/z*: calculated for $C_{29}H_{39}NO_8Na$: $[M + Na]^+$ 552.2568, found 552.2576. LC-MS (t_R= 3.1 min, λ = 254 nm, purity 99%).

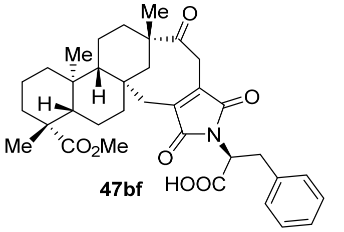

47bf

47bf, White solid, 47.1 mg, yield 80%. **^{1}H NMR** (500 MHz, $CDCl_3$) δ 7.28-7.25 (m, 2H), 7.23-7.20 (m, 1H), 7.14-7.13 (m, 2H), 5.00 (t, *J* = 8.5 Hz, 1H), 3.72 (dd, *J* = 18.5, 1.5 Hz, 1H), 3.68 (s, 3H), 3.50 (d, *J* = 8.5 Hz, 2H), 3.08 (dd, *J* = 18.5, 1.5 Hz, 1H), 2.77 (d, *J* = 14.5 Hz, 1H), 2.47 (dq, *J* = 13.0, 3.0 Hz, 1H), 2.18-2.01 (m, 3H), 1.89-1.81 (m, 2H), 1.74 (dd, *J* = 14.5, 2.5 Hz, 1H), 1.58 (d, *J* = 14.5 Hz, 1H), 1.47-1.36 (m, 2H), 1.31 (dt, *J* = 13.5, 3.0 Hz, 1H), 1.18 (s, 3H), 1.07 (s, 3H), 1.10-0.94 (m, 6H), 0.91-0.81 (m, 2H), 0.64 (s, 3H); **^{13}C NMR** (125 MHz, $CDCl_3$) δ 212.0, 178.0, 174.3,

170.7, 169.5, 139.7, 137.8, 136.7, 129.1, 128.9, 127.3, 59.0, 57.5, 53.5, 51.5, 49.8, 48.4, 44.1, 40.4, 40.0, 39.8, 38.4, 37.6, 37.1, 34.7, 34.5, 29.4, 28.8, 22.8, 19.7, 19.2, 18.4, 14.2; **HRMS** (ESI): *m/z*: calculated for $C_{35}H_{43}NO_7Na$: $[M + Na]^+$ 612.2932, found 612.2939. LC-MS (t_R= 3.04 min, λ = 254 nm, purity 98%).

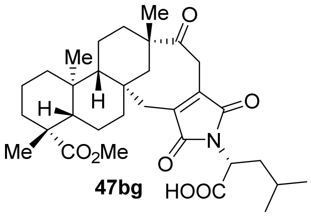

47bg

47bg, White solid, 47.0 mg, yield 85%. **^{1}H NMR** (500 MHz, $CDCl_3$) δ 9.13 (brs, 1H), 4.76 (dd, *J* = 11.5, 4.5 Hz, 1H), 3.89 (dd, *J* = 18.5, 1.5 Hz, 1H), 3.67 (s, 3H), 3.19 (dd, *J* = 18.5, 1.5 Hz, 1H), 2.85 (d, *J* = 14.0 Hz, 1H), 2.50 (dq, *J* = 13.5, 3.0 Hz, 1H), 2.28 (ddd, *J* = 14.5, 12.0, 4.0 Hz, 1H), 2.19-2.07 (m, 3H), 1.94 (dd, *J* = 14.5, 2.5 Hz, 1H), 1.89-1.80 (m, 2H), 1.75 (d, *J* = 12.5 Hz, 1H), 1.62-1.59 (m, 1H), 1.49-1.41 (m, 3H), 1.34 (dt, *J* = 13.5, 3.0 Hz, 1H), 1.17 (s, 3H), 1.13 (s, 3H), 1.09 (d, *J* = 14.5 Hz, 1H), 1.07 (dd, *J* = 12.5, 2.5 Hz, 1H), 1.04-0.96 (m, 3H), 0.93 (d, *J* = 6.5 Hz, 3H), 0.91 (d, *J* = 6.5 Hz, 3H), 0.91-0.82 (m, 2H), .067 (s, 3H); **^{13}C NMR** (125 MHz, $CDCl_3$) δ 212.2, 178.0, 175.6, 171.0, 169.9, 140.0, 137.9, 59.0, 57.5, 51.5, 50.9, 50.0, 48.5, 44.1, 40.5, 40.1, 39.8, 38.5, 37.6, 37.3, 37.1, 34.7, 29.5, 28.7, 25.3, 23.3, 22.9, 21.1, 19.8, 19.2, 18.4, 14.2; **HRMS** (ESI): *m/z*: calculated for $C_{32}H_{45}NO_7Na$: $[M + Na]^+$ 578.3088, found 578.3098. LC-MS (t_R= 3.12 min, λ = 254 nm, purity >99%).

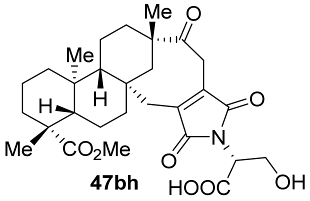

47bh

47bh, White solid, 37.0 mg, yield 70%. **^{1}H NMR** (500 MHz, $CDCl_3$) δ 6.48 (brs, 2H), 4.87 (dd, *J* = 6.0, 3.5 Hz, 1H), 4.24 (dd, *J* = 12.5, 5.5 Hz, 1H), 4.12 (dd, *J* = 12.5, 3.5 Hz, 1H), 3.90 (d, *J* = 18.5 Hz, 1H), 3.67 (s, 3H), 3.20 (d, *J* = 18.5 Hz, 1H), 2.87 (d, *J* = 14.0 Hz, 1H), 2.50 (d, *J* = 11.5 Hz, 1H), 2.18-2.04 (m, 3H), 1.96 (d, *J* = 14.5 Hz, 1H), 1.87-1.74 (m, 3H), 1.61 (d, *J* = 14.0 Hz, 1H), 1.48-1.41 (m, 2H), 1.36 (dt, *J* = 13.5, 3.5 Hz, 1H), 1.17 (s, 3H), 1.13 (s, 3H), 1.11-0.97 (m, 5H), 0.93-0.83 (m, 2H), 0.66 (s, 3H); **^{13}C NMR** (125 MHz, $CDCl_3$) δ 212.0, 178.1, 171.5, 171.2, 170.3, 140.4, 138.4, 61.2, 58.9, 57.5, 55.1, 51.6, 49.9, 48.5, 44.1, 40.4, 40.2, 39.8, 38.3, 37.6, 37.2, 34.6, 29.4, 28.8, 23.0, 19.7, 19.2, 18.4, 14.2; **HRMS** (ESI): *m/z*: calculated for $C_{29}H_{39}NO_8Na$: $[M + Na]^+$ 552.2568, found 552.2578. LC-MS (t_R= 3.26 min, λ = 254 nm, purity 99%).

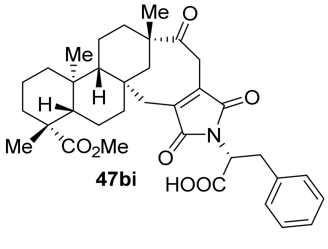

47bi

47bi, White solid, 51.8 mg, yield 88%. **^{1}H NMR** (500 MHz, $CDCl_3$) δ 10.49 (brs, 1H), 7.27-7.24 (m, 2H), 7.21-7.18 (m, 1H), 7.15-7.13 (m, 2H), 5.02 (dd, *J* = 11.0, 6.0 Hz, 1H), 3.80 (d, *J* = 18.5 Hz, 1H), 3.67 (s, 3H), 3.53-3.45 (m, 2H), 3.08 (d, *J* = 18.5 Hz, 1H), 2.72 (d, *J* = 14.5 Hz, 1H), 2.47 (dq, *J* = 13.5, 2.5 Hz, 1H), 2.16 (d, *J* = 13.0 Hz, 1H), 2.05-1.97 (m, 2H), 1.84 (qt, *J* = 13.5, 3.5 Hz, 1H), 1.72-1.69 (m, 3H), 1.57 (d, *J* = 15.0 Hz, 1H), 1.45-1.35 (m, 2H), 1.17 (s, 3H), 1.09 (s, 3H), 1.04-0.92 (m, 6H), 0.83 (td, *J* = 13.5, 4.0 Hz, 1H), 0.72 (t, *J* = 13.0 Hz, 1H), 0.63 (s, 3H);); **^{13}C NMR** (125 MHz, $CDCl_3$) δ 212.0, 178.0, 174.1, 170.5, 169.6, 139.7, 137.7, 136.6, 129.0, 128.9, 127.3, 58.9, 57.5, 53.4, 51.5, 49.8, 48.5, 44.1, 40.4, 39.9, 39.5, 38.4, 37.6, 37.1, 34.7, 3.6, 29.5, 28.7, 22.8, 19.7, 19.2, 18.4, 14.2; **HRMS** (ESI): *m/z*: calculated for $C_{35}H_{44}NO_7$: $[M + H]^+$ 590.3112, found 590.3124. LC-MS (t_R= 0.89 min, λ = 254 nm, purity 96%).

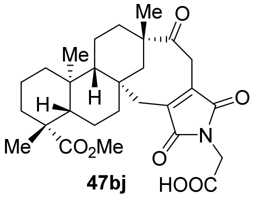

47bj

47bj, White solid, 41.9 mg, yield 84%. **^{1}H NMR** (500 MHz, $CDCl_3$) δ 10.40 (brs, 1H), 4.31 (s, 2H), 3.90 (dd, *J* = 18.5, 1.5 Hz, 1H), 3.67 (s, 3H), 3.22 (dd, *J* = 18.5, 1.5 Hz, 1H), 2.88 (d, *J* = 14.0 Hz, 1H), 2.51 (dq, *J* = 13.5, 3.0 Hz, 1H), 2.18-2.06 (m, 3H), 1.94 (dd, *J* = 14.5, 2.5 Hz, 1H), 1.90-1.74 (m, 3H), 1.62-1.59 (m, 1H), 1.49-1.41 (m, 2H), 1.34 (dt, *J* = 13.5, 3.5 Hz, 1H), 1.16 (s, 3H), 1.13 (s, 3H), 1.11-1.05 (m, 2H), 1.04-0.96 (m, 3H), 0.93-0.82 (m, 2H), 0.67 (s, 3H); **^{13}C NMR** (125 MHz, $CDCl_3$) δ 212.0, 178.1, 172.5, 170.7, 169.6, 140.3, 138.4, 58.9, 57.5, 51.5, 49.9, 48.5, 44.1, 40.4, 40.1, 39.8, 39.0, 38.4, 37.6, 37.2, 34.6, 29.5, 28.7, 22.9, 19.7, 19.2, 18.4, 14.2; **HRMS** (ESI): *m/z*: calculated for $C_{28}H_{37}NO_7Na$: $[M + Na]^+$ 522.2462, found 522.2474. LC-MS (t_R= 0.48 min, λ = 254 nm, purity >99%).

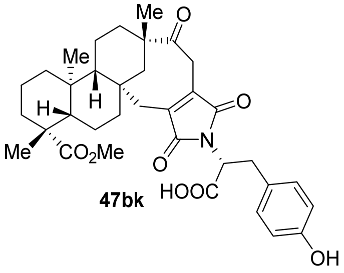

47bk

47bk, Pale yellow solid, 38.5 mg, yield 64%. **^{1}H NMR** (500 MHz, $CDCl_3$) δ 6.69 (d, *J* = 8.5 Hz, 2H), 6.71 (d, J = 9.0 Hz, 2H), 4.96 (dd, *J* = 11.5, 5.5 Hz, 1H), 3.80 (d, *J* = 18.5 Hz, 1H), 3.67 (s, 3H), 3.43 (dd, *J* = 14.5, 5.5 Hz, 1H), 3.38 (dd, *J* = 14.5, 11.5 Hz, 1H), 3.08 (d, *J* = 18.5 Hz, 1H), 2.72 (d, *J* = 14.5 Hz, 1H), 2.47 (d, *J* = 13.5 Hz, 1H), 2.15 (d, *J* = 13.5 Hz, 1H), 2.06-1.98 (m, 2H), 1.83 (q, *J* = 13.5 Hz, 1H), 1.74-1.71 (m, 3H), 1.57 (d, *J* = 13.5 Hz, 1H), 1.45-1.35 (m, 2H), 1.16 (s, 3H), 1.09 (s, 3H), 1.05-0.93 (m, 7H), 0.85-0.74 (m, 2H), 0.63 (s, 3H); **^{13}C NMR** (125 MHz, $CDCl_3$) δ 212.3, 178.3, 173.8, 170.6, 169.8, 154.9, 139.7, 137.7, 130.3, 128.6, 115.8, 58.9, 57.5, 53.7, 51.6, 49.7, 48.5, 44.1, 40.4, 39.9, 39.3, 38.4, 37.6, 37.1, 34.7, 33.8, 29.5, 28.8, 22.8, 19.7, 19.2, 18.4, 14.2; **HRMS** (ESI): *m/z*: calculated for $C_{35}H_{43}NO_8Na$: $[M + Na]^+$ 628.2881, found 628.2893. LC-MS (t_R= 2.42 min, λ = 210 nm, purity 95%).

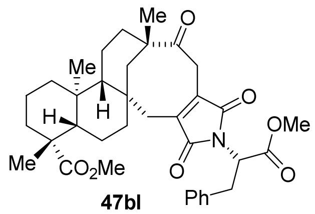

47bl, Yellow oil, yield: (53.6 mg, 89%) **^{1}H NMR** (500 MHz, $CDCl_3$) δ 7.27 – 7.09 (m, 3H), 7.10 – 7.00 (m, 2H), 4.85 (dd, *J* = 10.5, 6.3 Hz, 1H), 3.71 (s, 3H), 3.68 – 3.57 (m, 4H), 3.45 – 3.35 (m, 2H), 3.00 (d, *J* = 18.4 Hz, 1H), 2.71 (d, *J* = 14.2 Hz, 1H), 2.40 (dq, *J* = 13.4, 3.1 Hz, 1H), 2.18 – 1.90 (m, 3H), 1.90 – 1.59 (m, 4H), 1.60 – 1.46 (m, 1H), 1.44 – 1.30 (m, 2H), 1.25 (dt, *J* = 13.4, 3.3 Hz, 1H), 1.11 (s, 3H), 1.06 – 0.86 (m, 8H), 0.79 (tdd, *J* = 17.8, 12.9, 3.7 Hz, 2H), 0.57 (s, 3H). **^{13}C NMR** (126 MHz, $CDCl_3$) δ 211.7, 177.6, 170.6, 169.4, 169.3, 139.4, 137.5, 136.7, 128.9, 128.6, 126.9, 58.8, 57.3, 53.6, 52.9, 51.2, 49.6, 48.2, 43.8, 40.2, 39.7, 39.6, 38.2, 37.4, 36.9, 34.4, 34.4, 29.2, 28.5, 22.5, 19.5, 19.0, 18.2, 14.0. **HRMS** (ESI) m/z: anal. calculated for $[C_{36}H_{45}NO_7 + NH_4]^+$: 621.3534, found: 621.3529. LC-MS (t_R= 3.80 min, λ = 254 nm, >99%).

Ring-cleavage of 5b and 5c.

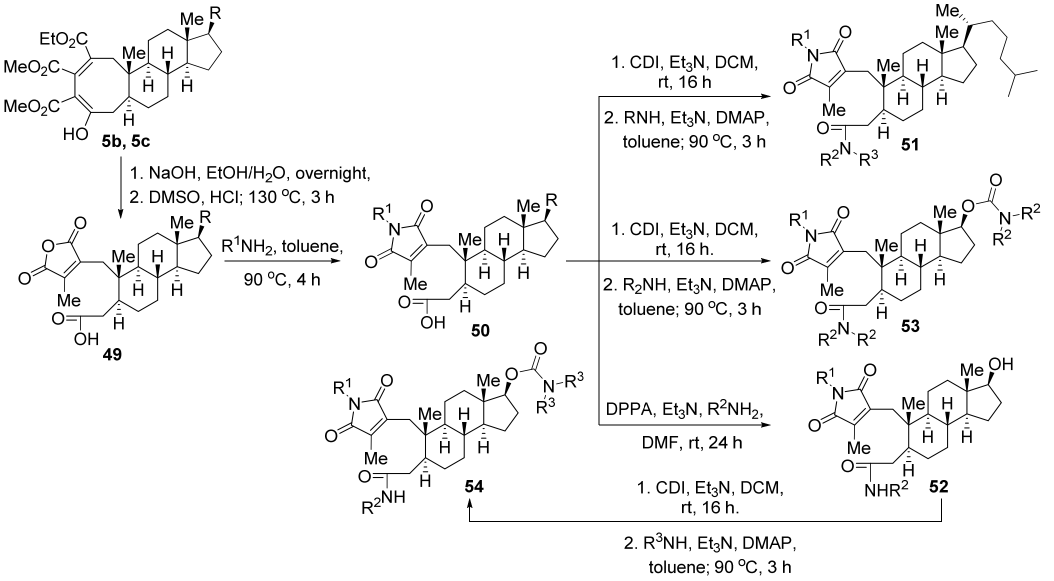

Supplementary Figure 16. Ring-cleavage of 5b and 5c.

General procedure H was used for preparation of **50**. General procedure F was used for formation of carbamates **51**, **52** and **54**.

To a solution of **5b** or **5c** (1.44 g, 2.33 mol) in EtOH (20 mL) and H_2O (5 mL) was added NaOH (928 mg, 23.3 mmol). The reaction mixture was heated at 100 ºC overnight. The mixture was cooled to room temperature, acidification of the mixture with 2N HCl, diluted with EtOAc (150 mL), and washed with brine (50 mL × 2). The organic phase was dried over sodium sulfate and concentrated in vacuum to afford crude product which was directly used in the next step without further purification. To a solution of the above product in DMSO (10 mL) was added HCl (3 mL,

2.0 N). The reaction mixture was heated at 130 ºC for 2 h. The mixture was cooled to room temperature then diluted with ethyl acetate (150 mL), and washed with brine (50 mL × 3). The organic phase was dried over sodium sulfate and concentrated in vacuum. Flash column chromatography over silica column afforded **49**.

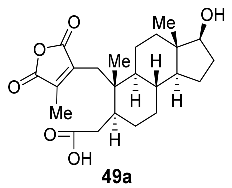

49a

49a, yield: (189 mg, 47%). **^{1}H NMR** (500 MHz, DMSO-d_6) δ 12.03 (s, 1H), 4.59 – 4.34 (m, 1H), 3.60 – 3.46 (m, 1H), 2.79 (dd, *J* = 16.7, 1.5 Hz, 1H), 2.61 (d, *J* = 14.4 Hz, 1H), 2.53 (d, *J* = 14.3 Hz, 1H), 2.08 (s, 3H), 2.00 – 1.82 (m, 3H), 1.79 (dt, *J* = 12.5, 3.4 Hz, 1H), 1.66 – 1.43 (m, 4H), 1.43 – 1.30 (m, 2H), 1.29 – 0.97 (m, 4H), 0.98 – 0.88 (m, 2H), 0.88 (s, 3H), 0.85 – 0.72 (m, 1H), 0.70 (s, 3H). **^{13}C NMR** (126 MHz, DMSO) δ 174.8, 167.0, 166.8, 144.5, 141.8, 80.5, 51.3, 48.8, 42.9, 42.6, 37.2, 36.7, 36.6, 31.7, 30.4, 30.1, 28.6, 23.50, 22.6, 16.4, 12.1, 10.8. **HRMS** (ESI) m/z: anal. calculated for $[C_{23}H_{32}O_6 + H]^+$: 405.2272, found: 405.2260.

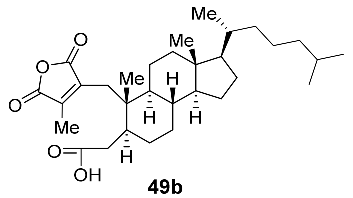

49b

49b, yield: (398 mg, 44%). **^{1}H NMR** (500 MHz, $CDCl_3$) δ 2.88 (dd, *J* = 16.5, 1.7 Hz, 1H), 2.63 (d, *J* = 14.1 Hz, 1H), 2.47 (d, *J* = 14.1 Hz, 1H), 2.13 (s, 3H), 2.09 – 1.97 (m, 2H), 1.89 – 1.75 (m, 2H), 1.71 (dq, *J* = 13.4, 3.6 Hz, 1H), 1.65 – 1.42 (m, 5H), 1.42 – 0.93 (m, 16H), 0.91 (t, *J* = 3.3 Hz, 5H), 0.88 (d, *J* = 2.4 Hz, 3H), 0.86 (d, *J* = 2.4 Hz, 3H), 0.85 – 0.76 (m, 2H), 0.69 (s, 3H). **^{13}C NMR** (126 MHz, $CDCl_3$) δ 179.1, 166.4, 166.1, 143.4, 142.3, 56.8, 56.1, 49.2, 42.76, 42.4, 40.0, 40.0, 39.5, 36.5, 36.4, 36.1, 35.8, 32.0, 30.4, 28.5, 28.2, 28.0, 24.0, 23.7, 23.0, 22.8, 22.5, 18.6, 16.1, 12.3, 10.8. **HRMS** (ESI) m/z: anal. calculated for $[C_{31}H_{48}O_5 + Na]^+$: 523.3394, found: 523.3384.

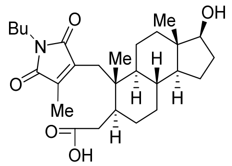

50a

50a, yield: (40.9 mg, 89%). **^{1}H NMR** (500 MHz, $CDCl_3$) δ 3.65 (t, *J* = 8.5 Hz, 1H), 3.46 (t, *J* = 7.3 Hz, 2H), 3.03 (dd, *J* = 16.2, 1.8 Hz, 1H), 2.51 (d, *J* = 14.3 Hz, 1H), 2.44 (d, *J* = 14.3 Hz, 1H), 2.11 – 1.93 (m, 4H), 1.88 (ddt, *J* = 15.6, 12.3, 3.3 Hz, 2H), 1.67 (dq, *J* = 13.4, 3.5 Hz, 1H), 1.61 – 1.37 (m, 6H), 1.37 – 1.18 (m, 4H), 1.18 – 1.02 (m, 2H), 0.98 – 0.87 (m, 7H), 0.87 – 0.80 (m, 1H), 0.78 (s, 3H), 0.75 (dd, *J* = 12.8, 4.1 Hz, 1H). **^{13}C NMR** (126 MHz, $CDCl_3$) δ 178.5, 172.6, 172.0, 139.8, 138.4, 81.8, 51.4, 49.2, 42.9, 42.4, 40.1, 37.9, 36.9, 36.8, 36.4, 31.2, 30.6, 30.0, 28.4, 23.3, 22.6, 19.9, 16.1, 13.6, 11.4, 10.0. **HRMS** (ESI) m/z: anal. calculated for $[C_{27}H_{41}NO_5 + Na]^+$: 482.2877,

found: 482.2882. LC-MS (t_R= 2.27 min, λ = 254 nm, purity 98%).

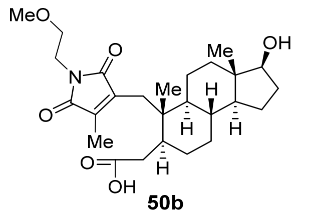

50b

50b, yield: (34.1 mg, 74%). **^{1}H NMR** (400 MHz, $CDCl_3$) δ 3.70 – 3.59 (m, 2H), 3.50 (t, *J* = 5.7 Hz, 2H), 3.32 (s, 3H), 3.03 (dd, *J* = 16.3, 1.3 Hz, 1H), 2.52 (d, *J* = 14.3 Hz, 1H), 2.45 (d, *J* = 14.2 Hz, 1H), 2.13 – 1.95 (m, 5H), 1.95 – 1.80 (m, 2H), 1.76 – 1.62 (m, 1H), 1.63 – 1.33 (m, 5H), 1.34 – 1.00 (m, 4H), 1.01 – 0.82 (m, 5H), 0.77 (s, 3H). **^{13}C NMR** (101 MHz, $CDCl_3$) δ 178.5, 172.5, 171.8, 140.1, 138.6, 81.8, 69.4, 58.5, 51.4, 49.1, 42.8, 42.4, 40.0, 37.4, 36.9, 36.7, 36.4, 31.2, 30.5, 30.0, 28.5, 23.3, 22.5, 16.1, 11.4, 10.1. **HRMS** (ESI) m/z: anal. calculated for $[C_{26}H_{39}NO_6 + Na]^+$: 484.2670, found: 484.2668. LC-MS (t_R= 1.66 min, λ = 254 nm, purity 98%).

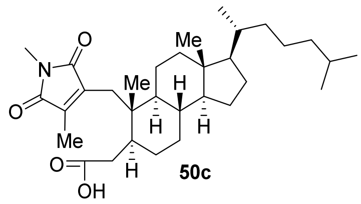

50c

50c, White solid, 24.8 mg, yield 48%. **^{1}H NMR** (500 MHz, $CDCl_3$) δ 10.85 (brs, 1H), 3.03 (dd, *J* = 16.0, 1.0 Hz, 1H), 2.97 (s, 3H), 2.50 (d, *J* = 14.5, 1H), 2.43 (d, *J* = 14.5, 1H), 2.04-1.96 (m, 2H), 2.02 (s, 3H), 1.85-1.76 (m, 2H), 1.65 (dq, *J* = 13.5, 3.5 Hz, 1H), 1.58-1.41 (m, 5H), 1.38-0.94 (m, 16H), 0.90 (d, *J* = 6.5 Hz, 1H), 0.86 (s, 3H), 0.86 (d, *J* = 6.5 Hz, 3H), 0.85 (d, *J* = 7.5 Hz, 3H), 0.83-0.73 (m, 2H), 0.68 (s, 3H); **^{13}C NMR** (125 MHz, $CDCl_3$) δ 179.2, 172.9, 172.4, 140.3, 139.0, 57.1, 56.4, 49.1, 42.6 (2 carbons), 40.4, 40.1, 39.7, 37.0, 36.6, 36.3, 36.0, 31.4, 30.7, 28.9, 28.5, 28.2, 24.3, 24.2, 24.0, 23.1, 23.0, 22.8, 18.8, 16.4, 12.5, 10.3; **HRMS** (ESI): *m/z*: calculated for $C_{32}H_{51}NO_4Na$: $[M + Na]^+$ 536.3710, found 536.3705. LC-MS (t_R= 3.86 min, λ = 254 nm, purity 99%).

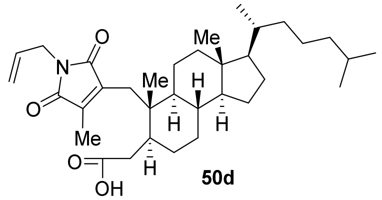

50d

50d, Pale yellow solid, 36.1 mg, yield 67%. **^{1}H NMR** (500 MHz, $CDCl_3$) δ 11.18 (brs, 1H), 5.77 (ddt, *J* = 17.0, 10.5, 4.5 Hz, 1H), 5.17-5.11 (m, 2H), 4.07 (dt, *J* = 5.5, 1.5 Hz, 2H), 3.04 (d, *J* = 15.5 Hz, 1H), 2.51 (d, *J* = 14.0 Hz, 1H), 2.44 (d, *J* = 14.0 Hz, 1H), 2.03 (s, 3H), 2.02-1.96 (m, 2H), 1.85-1.77 (m, 2H), 1.65 (dq, *J* = 13.0, 3.0 Hz, 1H), 1.56-0.94 (m, 19H), 0.90 (d, *J* = 6.5 Hz, 3H), 0.87 (s, 3H), 0.86 (d, *J* = 6.5 Hz, 3H), 0.85 (d, *J* = 7.0 Hz, 3H), 0.83-0.72 (m, 2H), 0.68 (s, 3H); **^{13}C NMR** (125 MHz, $CDCl_3$) δ 179.2, 172.4, 171.7, 140.2, 139.0, 132.0, 117.5, 57.1, 56.4, 49.2, 42.7, 42.6, 40.4, 40.2, 39.7, 36.9, 36.6, 36.3, 36.0, 31.3, 30.7, 28.9, 28.5, 28.2, 24.3, 24.0, 23.2, 23.0, 22.8, 18.8,

16.4, 12.6, 10.4; **HRMS** (ESI): *m/z*: calculated for $C_{34}H_{53}NO_4Na$: $[M + Na]^+$ 562.3867, found 562.3855. LC-MS (t_R= 3.89 min, λ = 254 nm, purity 99%).

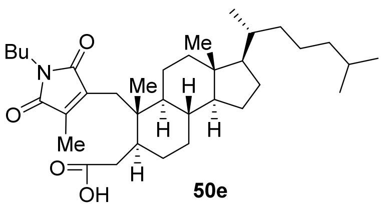

50e

50e, Pale yellow solid, 39.4 mg, yield 71%. **^{1}H NMR** (500 MHz, $CDCl_3$) δ 3.46 (t, *J* = 7.5 Hz, 2H), 3.06 (dd, *J* = 16.0, 1.5 Hz, 1H), 2.49 (d, *J* = 14.0 Hz, 1H), 2.42 (d, *J* = 14.5 Hz, 1H), 2.03-1.97 (m, 2H), 2.02 (s, 3H), 1.85-1.77 (m, 2H), 1.65 (dq, *J* = 13.5, 3.0 Hz, 1H), 1.55-0.96 (m, 24H), 0.90 (d, *J* = 6.5 Hz, 3H), 0.90 (t, *J* = 7.0 Hz, 3H), 0.86 (s, 3H), 0.86 (d, *J* = 6.5 Hz, 3H), 0.85 (d, *J* = 6.0 Hz, 3H), 0.82-0.73 (m, 2H), 0.68 (s, 3H); **^{13}C NMR** (125 MHz, $CDCl_3$) δ 179.0, 173.0, 172.2, 140.0, 138.8, 57.1, 56.5, 49.1, 42.6 (2 carbons), 40.4, 40.2, 39.7, 38.2, 37.0, 36.7, 36.3, 36.0, 31.3, 30.8, 30.7, 28.8, 28.5, 28.2, 24.3, 24.0, 23.2, 23.0, 22.8, 20.2, 18.8, 16.3, 13.9, 12.6, 10.3; **HRMS** (ESI): *m/z*: calculated for $C_{35}H_{57}NO_4Na$: $[M + Na]^+$ 578.4180, found 578.4167.

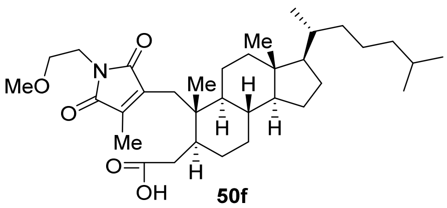

50f

50f, Pale yellow solid, 42.9 mg, yield 77%. **^{1}H NMR** (500 MHz, $CDCl_3$) δ 3.72-3.64 (m, 2H), 3.50 (t, *J* = 6.0 Hz, 2H), 3.31 (s, 3H), 3.05 (d, *J* = 11.0 Hz, 1H), 2.50 (d, *J* = 14.0 Hz, 1H), 2.43 (d, *J* = 14.0 Hz, 1H), 2.03 (s, 3H), 2.01-1.96 (m, 2H), 1.85-1.76 (m, 2H), 1.64 (dq, *J* = 13.5, 3.5 Hz, 1H), 1.55-0.94 (m, 19H), 0.90 (d, *J* = 6.5 Hz, 3H), 0.86 (s, 3H), 0.86 (d, *J* = 6.5 Hz, 3H), 0.85 (d, *J* = 6.5 Hz, 3H), 0.84-0.71 (m, 2H), 0.68 (s, 3H); **^{13}C NMR** (125 MHz, $CDCl_3$) δ 179.0, 172.8, 172.0, 140.3, 138.9, 69.7, 58.8, 57.1, 56.5, 49.1, 42.6 (2 carbons), 0.4, 40.2, 39.7, 37.6, 37.0, 36.6, 36.3, 36.0, 31.4, 30.7, 28.7, 28.5, 28.2, 24.3, 24.0, 23.2, 23.0, 22.8, 18.8, 16.3, 12.6, 10.4; **HRMS** (ESI): *m/z*: calculated for $C_{34}H_{55}NO_5Na$: $[M + Na]^+$ 580.3972, found 580.3970. LC-MS (t_R= 3.83 min, λ = 254 nm, purity >99%).

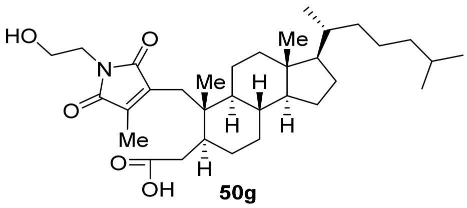

50g

50g, Pale yellow solid, 35.8 mg, yield 66%. **^{1}H NMR** (500 MHz, $CDCl_3$) δ 6.65 (brs, 1H), 3.76-3.69 (m, 3H), 3.62-3.58 (m, 2H), 3.03 (d, *J* = 17.0 Hz, 1H), 2.51 (d, *J* = 14.5 Hz, 1H), 2.46 (d, *J* = 14.0 Hz, 1H), 2.06-2.03 (m, 1H), 2.04 (s, 3H), 1.93 (dd, *J* = 17.0, 10.5 Hz, 1H), 1.86-1.76 (m, 2H), 1.60-1.44 (m, 6H), 1.39-0.96 (m, 15H), 0.90 (d, *J* = 6.5 Hz, 3H), 0.87-0.85 (m, 9H), 0.83-0.75 (m,

2H), 0.69 (s, 3H); **^{13}C NMR** (125 MHz, $CDCl_3$) δ 178.9, 173.4, 172.3, 139.9, 139.3, 60.7, 57.2, 56.5, 48.8, 43.0, 42.6, 41.1, 40.5, 39.7, 36.8, 36.5, 36.3, 36.0, 30.8, 30.6, 29.4, 28.5, 28.2, 24.3, 24.0, 23.0 (2 carbons), 22.8, 18.8, 16.6, 12.5, 10.6; **HRMS** (ESI): *m/z*: calculated for $C_{33}H_{53}NO_5Na$: [M + Na]$^+$ 566.3816, found 566.3827. LC-MS (t_R= 3.73 min, λ = 254 nm, purity 99%).

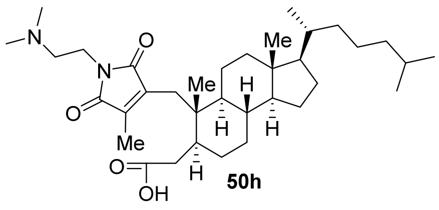

50h

50h, Pale yellow solid, 18.2 mg, yield 32%. **^{1}H NMR** (500 MHz, $CDCl_3$) δ 11.35 (brs, 1H), 3.76-3.71 (m, 2H), 3.15 (dt, *J* = 13.0, 6.5 Hz, 1H), 2.91 (dt, *J* = 13.5, 5.5 Hz, 1H), 2.78 (d, *J* = 16.5 Hz, 1H), 2.68 (s, 6H), 2.51 (s, 2H), 2.06-2.03 (m, 1H), 2.04 (s, 3H), 1.85-1.69 (m, 3H), 1.59-1.44 (m, 5H), 1.37-0.76 (m, 17H), 0.90 (d, *J* = 6.5 Hz, 3H), 0.88 (s, 3H), 0.86 (d, *J* = 6.5 Hz, 3H), 0.85 (d, *J* = 6.5 Hz, 3H), 0.68 (s, 3H); **^{13}C NMR** (125 MHz, $CDCl_3$) δ 177.9, 172.3, 171.9, 140.3, 139.4, 57.2, 56.5, 55.8, 49.9, 43.4, 43.3, 42.6, 40.4, 40.3, 39.7, 38.4, 36.3 (2 carbons), 36.0, 33.5, 31.3, 31.0, 30.1, 28.5, 28.2, 24.4, 24.0, 23.0, 22.8 (2 carbons), 18.8, 18.6, 12.4, 10.8; **HRMS** (ESI): *m/z*: calculated for $C_{35}H_{58}N_2O_4Na$: [M + Na]$^+$ 593.4289, found 593.4298. LC-MS (t_R= 2.99 min, λ = 254 nm, purity 97%).

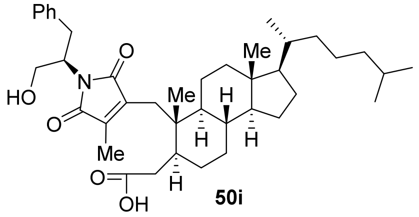

50i

50i, Pale yellow solid, 43.6 mg, yield 70%. **^{1}H NMR** (500 MHz, $CDCl_3$) δ 7.24-7.21 (m, 2H), 7.18-7.12 (m, 3H), 4.42 (qd, *J* = 8.5, 3.5 Hz, 1H), 4.06 (dd, *J* = 12.0, 8.5 Hz, 1H), 3.74 (dd, *J* = 12.0, 3.5 Hz, 1H), 3.05-2.94 (m, 3H), 2.45 (s, 2H), 2.02 (dt, *J* = 12.5, 3.5 Hz, 1H), 1.95-1.88 (m, 1H), 1.92 (s, 3H), 1.86-1.73 (m, 2H), 1.55-0.92 (m, 21H), 0.90 (d, *J* = 6.5 Hz, 3H), 0.86 (d, *J* = 7.0 Hz, 3H), 0.86 (s, 3H), 0.85 (d, *J* = 6.5 Hz, 3H), 0.82-0.63 (m, 2H), 0.68 (s, 3H); **^{13}C NMR** (125 MHz, $CDCl_3$) δ 178.4, 173.5, 172.2, 139.3, 138.8, 137.6, 129.2, 128.7, 126.8, 61.7, 57.1, 56.5, 55.2, 48.7, 43.0, 42.6, 40.4, 40.0, 39.7, 36.5, 36.3, 36.0, 35.2, 30.8, 30.3, 29.2, 28.5, 28.2, 24.3, 24.0, 23.0, 22.9, 22.8, 18.8, 16.6, 12.5, 10.7; **HRMS** (ESI): *m/z*: calculated for $C_{40}H_{59}NO_5Na$: [M + Na]$^+$ 656.4285, found 656.4274. LC-MS (t_R= 3.84 min, λ = 254 nm, purity >99%).

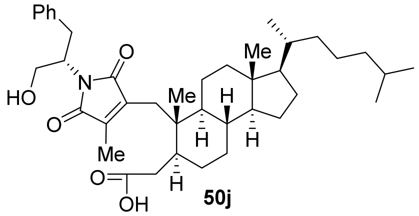

50j

50j, Pale yellow solid, 44.3 mg, yield 70%. **^{1}H NMR** (500 MHz, $CDCl_3$) δ 7.23-7.14 (m, 5H), 4.38

(dtd, J = 9.5, 7.5, 4.0 Hz, 1H), 3.93 (dd, J = 12.0, 7.0 Hz, 1H), 3.79 (dd, J = 12.0, 4.0 Hz, 1H), 3.11-3.01 (m, 2H), 2.95 (d, J = 16.5 Hz, 1H), 2.42 (d, J = 14.5 Hz, 1H), 2.35 (d, J = 14.0 Hz, 1H), 2.00 (dt, J = 13.0, 3.5 Hz, 1H), 1.95 (s, 3H), 1.92 (dd, J = 17.0, 11.0 Hz, 1H), 1.87-1.80 (m, 1H), 1.72 (dq, J = 14.0, 3.5 Hz, 1H), 1.62 (dq, J = 13.0, 3.5 Hz, 1H), 1.57-1.48 (m, 4H), 1.43 (dd, J = 13.0, 3.5 Hz, 1H), 1.39-0.94 (m, 14H), 0.90 (d, J = 6.5 Hz, 3H), 0.87 (d, J = 6.5 Hz, 3H), 0.86 (d, J = 6.5 Hz, 3H), 0.82 (s, 3H), 0.78-0.64 (m, 2H), 0.68 (s, 3H); **^{13}C NMR** (125 MHz, $CDCl_3$) δ 178.6, 173.2, 172.5, 139.9, 138.8, 138.0, 129.3, 128.6, 126.7, 63.1, 57.0, 56.4, 55.6, 49.1, 42.7, 42.6, 40.4, 40.0, 39.7, 36.9, 36.6, 36.3, 36.0, 34.9, 31.2, 30.8, 29.1, 28.5, 28.2, 24.3, 24.0, 23.0, 22.8, 18.8, 16.4, 12.5, 10.4; **HRMS** (ESI): *m/z*: calculated for $C_{40}H_{59}NO_5Na$: $[M + Na]^+$ 656.4285, found 656.4304. LC-MS (t_R= 3.79 min, λ = 210 nm, purity >99%).

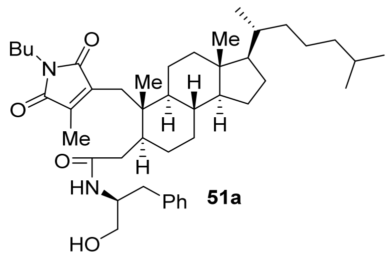

51a

51a, Pale yellow solid, 39.2 mg, yield 57%. **^{1}H NMR** (500 MHz, $CDCl_3$) δ 7.30-7.27 (m, 2H), 7.24-7.19 (m, 3H), 6.45 (d, J = 7.0 Hz, 1H), 4.15-4.09 (m, 1H), 3.76 (dt, J = 11.5, 4.0 Hz, 1H), 3.53 (dt, J = 11.5, 5.5 Hz, 1H), 3.49-3.38 (m, 2H), 3.23 (t, J = 5.5 Hz, 1H), 2.89-2.83 (m, 3H), 2.46 (d, J = 14.5 Hz, 1H), 2.41 (d, J = 14.0 Hz, 1H), 2.03 (s, 3H), 2.00 (dt, J = 13.0, 3.0 Hz, 1H), 1.85-1.76 (m, 2H), 1.72 (dq, J = 14.0, 3.5 Hz, 1H), 1.66 (dq, J = 13.5, 3.5 Hz, 1H), 1.56-0.95 (m, 22H), 0.93 (t, J = 7.5 Hz, 3H), 0.89 (d, J = 6.5 Hz, 3H), 0.86 (d, J = 7.0 Hz, 3H), 0.86 (d, J = 7.0 Hz, 3H) , 0.83 (s, 3H), 0.73-0.64 (m, 2H), 0.68 (s, 3H); **^{13}C NMR** (125 MHz, $CDCl_3$) δ 174.5, 174.0, 172.0, 140.7, 138.9, 138.3, 129.4, 128.7, 126.7, 65.0, 57.2, 56.4, 53.7, 49.2, 42.7 (2 carbons), 41.6, 40.5, 39.7, 39.2, 38.3, 37.4, 36.7, 36.3, 36.0, 31.6, 30.8, 30.7, 28.5, 28.4, 28.2, 24.2, 24.0, 23.4, 23.0, 22.8, 20.3, 18.8, 16.5, 13.9, 12.6, 10.4; **HRMS** (ESI): *m/z*: calculated for $C_{44}H_{68}N_2O_4Na$: $[M + Na]^+$ 711.5071, found 711.5097.

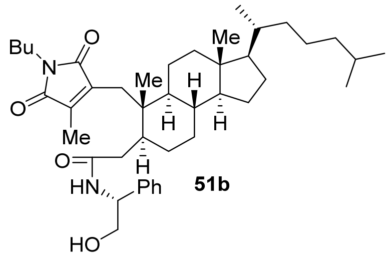

51b

51b, Pale yellow solid, 14.8 mg, yield 22%. **^{1}H NMR** (500 MHz, $CDCl_3$) δ 7.36-7.28 (m, 6H), 5.04 (td, J = 6.5, 4.0 Hz, 1H), 3.92 (dd, J = 11.5, 7.0 Hz, 1H), 3.87 (dd, J = 11.5, 3.5 Hz, 1H), 3.34 (dt, J = 14.0, 7.0 Hz, 1H), 3.22 (dt, J = 14.0, 7.0 Hz, 1H), 3.04 (dd, J = 12.5, 2.5 Hz, 1H), 2.50 (d, J = 14.0 Hz, 1H), 2.41 (d, J = 14.5 Hz, 1H), 2.02 (s, 3H), 2.02-1.92 (m, 2H), 1.83-1.76 (m, 2H), 1.63-0.83 (m, 38H), 0.68 (s, 3H), 0.68-0.60 (m, 2H); **^{13}C NMR** (125 MHz, $CDCl_3$) δ 174.9, 174.2, 172.0, 141.0, 139.5, 138.9, 128.9, 128.0, 127.0, 68.0, 57.1 (2 carbons), 56.4, 49.4, 42.7, 42.5, 42.3, 40.4, 39.7 (2 carbons), 38.2, 36.7, 36.3, 36.0, 32.0, 30.7 (2 carbons), 28.4, 28.2, 27.8, 24.2, 24.0, 23.4,

23.0, 22.8, 20.2, 18.8, 16.4, 13.9, 12.6, 10.3; **HRMS** (ESI): *m/z*: calculated for $C_{43}H_{66}N_2O_4Na$: [M + Na]$^+$ 697.4915, found 697.4935.

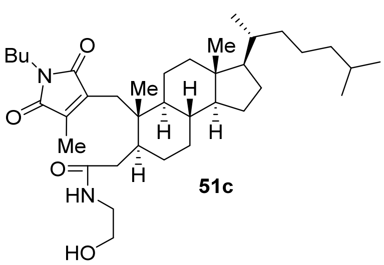

51c

51c, Pale yellow solid, 25.7 mg, yield 43%. **^{1}H NMR** (500 MHz, $CDCl_3$) δ 6.77 (t, *J* = 5.5 Hz, 1H), 3.71 (dqd, *J* = 11.0, 6.5, 3.5 Hz, 2H), 3.48 (t, *J* = 7.0 Hz, 2H), 3.44-3.34 (m, 2H), 3.21 (brs, 1H), 2.91 (dd, *J* = 13.0, 2.0 Hz, 1H), 2.49 (d, *J* = 14.0 Hz, 1H), 2.42 (d, *J* = 14.5 Hz, 1H), 2.03 (s, 3H), 1.99 (dt, *J* = 12.5, 3.5 Hz, 1H), 1.86 (dd, *J* = 13.0, 1.5 Hz, 1H), 1.83-1.76 (m, 1H), 1.73 (dq, *J* = 13.5, 3.5 Hz, 1H), 1.66 (dq, *J* = 13.5, 3.5 Hz, 1H), 1.57-0.94 (m, 23H), 0.92 (t, *J* = 7.5 Hz, 3H), 0.89 (d, *J* = 6.5 Hz, 3H), 0.86-0.85 (m, 9H), 0.74-0.64 (m, 2H), 0.68 (s, 3H); **^{13}C NMR** (125 MHz, $CDCl_3$) δ 175.3, 174.1, 172.0, 140.8, 139.0, 63.1, 57.1, 56.4, 49.4, 43.1, 42.6, 41.9, 40.4, 39.7, 39.3, 38.3, 36.7, 36.3, 36.0, 31.8, 30.8, 30.7, 28.4, 28.2, 24.2, 24.0, 23.4, 23.0,22.8, 20.2,18.8, 16.4, 13.9, 12.6, 10.4; **HRMS** (ESI): *m/z*: calculated for $C_{37}H_{62}N_2O_4Na$: [M + Na]$^+$ 621.4602, found 621.4618. LC-MS (t_R= 3.85 min, λ = 254 nm, purity >99%).

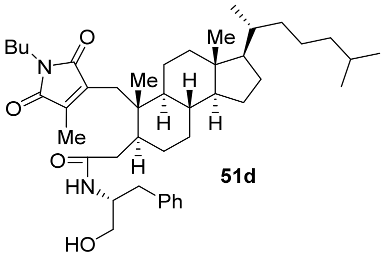

51d

51d, Pale yellow solid, 22.0 mg, yield 32%. **^{1}H NMR** (500 MHz, $CDCl_3$) δ 7.27-7.24 (m, 2H), 7.20-7.17 (m, 3H), 6.65 (d, *J* = 7.5 Hz, 1H), 4.23-4.16 (m, 1H), 3.74 (dd, *J* = 11.0, 3.5 Hz, 1H), 3.62 (dd, *J* = 11.0, 6.0 Hz, 1H), 3.48 (dt, J = 7.0, 7.0 Hz, 1H), 3.43 (dt, J = 7.0, 7.0 Hz, 1H), 2.92-2.79 (m, 3H), 2.47 (d, *J* = 14.0 Hz, 1H), 2.38 (d, *J* = 14.5 Hz, 1H), 2.02 (s, 3H), 1.99 (dt, *J* = 13.0, 3.5 Hz, 1H), 1.82 (t , *J* = 12.5 Hz, 2H), 1.63-0.95 (m, 25H), 0.93 (t, *J* = 7.5 Hz, 3H), 0.89 (d, *J* = 6.5 Hz, 3H), 0.87 (d, *J* = 7.0 Hz, 3H), 0.86 (d, *J* = 6.5 Hz, 3H), 0.82 (s, 3H), 0.68-0.62 (m, 1H), 0.67 (s, 3H), 0.55 (qd, *J* = 13.0, 3.0 Hz, 1H); **^{13}C NMR** (125 MHz, $CDCl_3$) δ 174.4, 174.1, 172.0, 140.9, 139.0, 138.2, 129.3, 128.7, 126.7, 65.5, 57.2, 56.4, 53.3, 49.6, 42.7, 42.4, 42.2, 40.4, 39.8, 39.7, 38.3, 37.3, 36.7, 36.3, 36.0, 32.1, 30.8, 29.9, 28.5, 28.3, 27.8, 24.3, 24.0, 23.4, 23.0, 22.8, 20.3, 18.9, 16.3, 13.9, 12.6, 10.4; **HRMS** (ESI): *m/z*: calculated for $C_{44}H_{68}N_2O_4Na$: [M + Na]$^+$ 711.5071, found 711.5090.

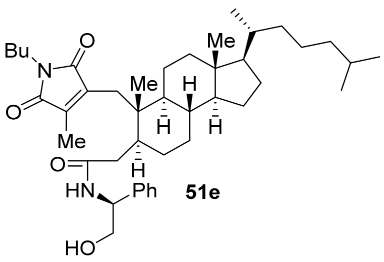

51e

51e, Pale yellow solid, 40.4 mg, yield 60%. **^{1}H NMR** (500 MHz, $CDCl_3$) δ 7.39-7.28 (m, 5H), 7.04 (d, J = 7.5 Hz, 1H), 5.09 (td, J = 6.5, 4.0 Hz, 1H), 3.88-3.81 (m, 2H), 3.44 (dt, J = 14.0, 7.0 Hz, 1H), 3.36 (dt, J = 14.0, 7.0 Hz, 1H), 3.23 (brs, 1H), 2.98 (dd, J = 13.5, 1.5 Hz, 1H), 2.48 (d, J = 14.0 Hz, 1H), 2.42 (d, J = 14.0 Hz, 1H), 2.03 (s, 3H), 2.00 (dt, J = 13.0, 3.5 Hz, 1H), 1.90 (dd, J = 13.0, 12.0 Hz, 1H), 1.83-1.78 (m, 2H), 1.67-0.81 (m, 39H), 0.78-0.64 (m, 2H), 0.69 (s, 3H); **^{13}C NMR** (125 MHz, $CDCl_3$) δ 174.6, 174.1, 172.0, 140.8, 139.5, 138.9, 129.0, 128.0, 127.2, 67.5, 57.1, 56.5, 56.4, 49.3, 42.7, 42.6, 41.9, 40.5, 39.7, 39.5, 38.3, 36.7, 36.3, 36.0, 31.8, 30.8 (2 carbons), 28.5, 28.2, 24.2, 24.0, 23.4, 23.0, 22.8, 20.2, 18.8, 16.5, 13.9, 12.6, 10.3; **HRMS** (ESI): *m/z*: calculated for $C_{43}H_{66}N_2O_4Na$: $[M + Na]^+$ 697.4915, found 697.4933.

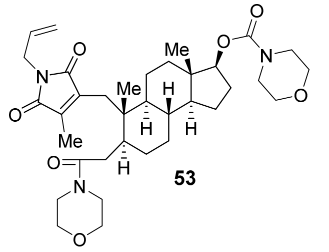

53

53, yield: (56.2 mg, 90%). **^{1}H NMR** (500 MHz, $CDCl_3$) δ 5.77 (ddt, J = 16.1, 10.7, 5.6 Hz, 1H), 5.20 – 5.04 (m, 2H), 4.59 (dd, J = 9.2, 7.7 Hz, 1H), 4.17 – 3.88 (m, 2H), 3.90 – 3.37 (m, 16H), 2.83 (d, J = 15.8 Hz, 1H), 2.58 – 2.36 (m, 2H), 2.16 (dtd, J = 13.9, 9.4, 6.3 Hz, 1H), 1.99 (s, 3H), 1.79 (dddd, J = 55.8, 29.6, 13.1, 3.2 Hz, 5H), 1.62 (tdt, J = 9.4, 6.7, 3.0 Hz, 1H), 1.58 – 1.48 (m, 2H), 1.43 (qd, J = 13.3, 3.4 Hz, 1H), 1.27 (dqd, J = 24.0, 13.0, 12.0, 3.5 Hz, 3H), 1.14 – 0.97 (m, 2H), 0.97 – 0.85 (m, 4H), 0.81 (s, 3H). **^{13}C NMR** (126 MHz, $CDCl_3$) δ 171.9, 171.4, 170.8, 155.5, 139.6, 138.8, 132.0, 117.2, 83.6, 66.9, 66.6, 51.1, 49.5, 42.8, 42.5, 40.2, 39.7, 37.2, 36.2, 34.7, 31.0, 30.1, 29.3, 27.9, 23.4, 22.3, 16.5, 12.5, 10.2. **HRMS** (ESI) m/z: anal. calculated for $[C_{35}H_{51}N_3O_7 + Na]^+$: 648.3619, found: 648.3622. LC-MS (t_R= 3.06 min, λ = 254 nm, purity 99%).

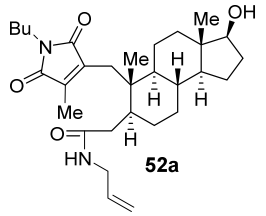

52a

52a, yield: (41.8 mg, 84%). **^{1}H NMR** (500 MHz, $CDCl_3$) δ 6.58 (t, J = 5.7 Hz, 1H), 5.85 (ddt, J = 17.3, 10.7, 5.5 Hz, 1H), 5.19 (dq, J = 17.1, 1.8 Hz, 1H), 5.10 (dq, J = 10.4, 1.6 Hz, 1H), 3.87 (tt, J = 5.6, 1.7 Hz, 2H), 3.61 (t, J = 8.5 Hz, 1H), 3.46 (td, J = 7.0, 1.5 Hz, 2H), 3.03 – 2.84 (m, 1H), 2.51 (d, J = 14.4 Hz, 1H), 2.43 (d, J = 14.2 Hz, 1H), 2.02 (s, 3H), 1.92 – 1.80 (m, 2H), 1.76 (dq, J = 13.9, 3.4 Hz, 1H), 1.70 (dq, J = 13.8, 3.5 Hz, 1H), 1.62 – 1.35 (m, 6H), 1.35 – 1.10 (m, 4H), 1.02 (td, J = 12.7, 3.5 Hz, 1H), 0.91 (t, J = 7.4 Hz, 3H), 0.87 (s, 3H), 0.77 (s, 3H), 0.75 – 0.59 (m, 2H). **^{13}C NMR** (126 MHz, $CDCl_3$) δ 173.7, 173.2, 171.8, 140.5, 138.6, 134.7, 115.5, 81.7, 51.4, 49.5, 42.8, 42.4, 42.1, 41.7, 39.3, 38.0, 37.0, 36.5, 31.7, 30.6, 30.5, 30.1, 27.5, 23.2, 22.7, 20.0, 16.1, 13.6, 11.5, 10.0. **HRMS** (ESI) m/z: anal. calculated for $[C_{30}H_{46}N_2O_4 + Na]^+$: 521.3350, found: 521.3356. LC-MS (t_R= 3.23 min, λ = 254 nm, purity 97%).

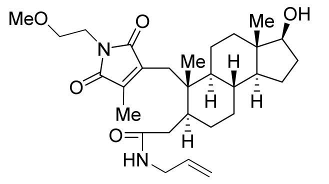

52b

52b, yield: (36.0 mg, 72%). **^{1}H NMR** (500 MHz, $CDCl_3$) δ 6.63 (t, *J* = 5.7 Hz, 1H), 5.86 (ddt, *J* = 17.2, 10.6, 5.5 Hz, 1H), 5.24 – 5.05 (m, 2H), 3.87 (ddt, *J* = 5.6, 3.9, 1.6 Hz, 2H), 3.62 (t, *J* = 8.5 Hz, 1H), 3.47 (td, *J* = 7.1, 1.3 Hz, 2H), 2.93 (dd, *J* = 12.9, 2.4 Hz, 1H), 2.52 (d, *J* = 14.2 Hz, 1H), 2.44 (d, *J* = 14.2 Hz, 1H), 2.03 (s, 4H), 1.94 – 1.81 (m, 3H), 1.77 (dq, *J* = 13.7, 3.2 Hz, 1H), 1.70 (dq, *J* = 13.8, 3.5 Hz, 1H), 1.64 – 1.38 (m, 7H), 1.36 – 1.11 (m, 5H), 1.10 – 1.00 (m, 1H), 0.92 (t, *J* = 7.4 Hz, 4H), 0.88 (s, 3H), 0.77 (s, 3H), 0.76 – 0.68 (m, 1H). **^{13}C NMR** (126 MHz, $CDCl_3$) δ 173.6, 173.1, 171.7, 140.4, 138.5, 134.6, 115.4, 81.5, 51.4, 49.4, 42.8, 42.4, 42.0, 41.6, 39.2, 37.9, 36.9, 36.5, 31.6, 30.5, 30.4, 30.0, 27.4, 23.2, 22.6, 19.9, 16.1, 13.5, 11.4, 10.0. **HRMS** (ESI) m/z: anal. calculated for $[C_{29}H_{44}N_2O_5 + Na]^+$: 523.3142, found: 523.3152. LC-MS (t_R= 2.35 min, λ = 254 nm, purity 96%).

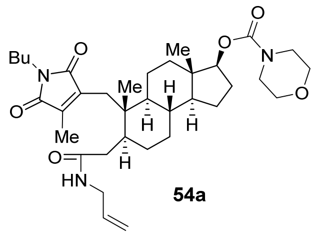

54a

54a, yield: (46.4 mg, 77%). **^{1}H NMR** (400 MHz, $CDCl_3$) δ 6.51 (t, *J* = 5.7 Hz, 1H), 5.86 (ddt, *J* = 17.2, 10.6, 5.5 Hz, 1H), 5.19 (dq, *J* = 17.2, 1.7 Hz, 1H), 5.10 (dq, *J* = 10.3, 1.5 Hz, 1H), 4.65 – 4.45 (m, 1H), 4.01 – 3.80 (m, 2H), 3.77 – 3.58 (m, 6H), 3.58 – 3.37 (m, 6H), 3.31 (s, 3H), 2.95 (dd, *J* = 13.0, 2.3 Hz, 1H), 2.53 (d, *J* = 14.4 Hz, 1H), 2.43 (d, *J* = 14.2 Hz, 1H), 2.27 – 2.07 (m, 1H), 2.02 (s, 3H), 1.96 – 1.67 (m, 4H), 1.67 – 1.38 (m, 5H), 1.38 – 1.11 (m, 4H), 1.11 – 0.98 (m, 1H), 0.88 (s, 3H), 0.81 (s, 3H), 0.81 – 0.58 (m, 2H). **^{13}C NMR** (101 MHz, $CDCl_3$) δ 173.5, 173.1, 171.5, 155.4, 140.7, 138.7, 134.8, 115.5, 83.5, 69.2, 66.5, 58.6, 51.0, 49.3, 44.1, 42.6, 42.5, 41.9, 41.7, 39.3, 37.4, 37.2, 36.3, 31.7, 30.0, 27.9, 27.6, 23.3, 22.5, 16.1, 12.6, 10.1. **HRMS** (ESI) m/z: anal. calculated for $[C_{35}H_{53}N_3O_6 + Na]^+$: 634.3827, found: 634.3827. LC-MS (t_R= 3.38 min, λ = 254 nm, purity 99%).

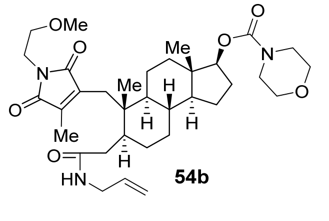

54b

54b, yield: (43.5 mg, 71%). **^{1}H NMR** (500 MHz, $CDCl_3$) δ 6.50 (t, *J* = 5.7 Hz, 1H), 5.97 – 5.76 (m, 1H), 5.18 (dt, *J* = 17.2, 1.6 Hz, 1H), 5.09 (dt, *J* = 10.4, 1.6 Hz, 1H), 4.02 – 3.77 (m, 2H), 3.77 – 3.55 (m, 3H), 3.56 – 3.44 (m, 2H), 3.29 (s, 3H), 2.91 (dd, *J* = 12.9, 2.3 Hz, 1H), 2.52 (d, *J* = 14.0 Hz, 1H), 2.43 (d, *J* = 14.2 Hz, 1H), 2.02 (s, 3H), 1.89 – 1.80 (m, 2H), 1.76 (dq, *J* = 13.8, 3.6 Hz, 1H), 1.73 – 1.59 (m, 2H), 1.59 – 1.34 (m, 5H), 1.33 – 1.09 (m, 3H), 1.02 (td, *J* = 12.8, 3.7 Hz, 1H), 0.92

(dd, J = 11.2, 7.2 Hz, 1H), 0.87 (s, 3H), 0.76 (s, 3H), 0.74 – 0.58 (m, 2H). **^{13}C NMR** (126 MHz, $CDCl_3$) δ 173.5, 173.1, 171.6, 140.6, 138.9, 134.7, 115.5, 81.6, 69.2, 58.5, 51.4, 49.5, 42.8, 42.5, 42.0, 41.7, 39.3, 37.4, 37.0, 36.5, 31.8, 30.5, 30.0, 27.6, 23.2, 22.7, 16.1, 11.5, 10.0. **HRMS** (ESI) m/z: anal. calculated for $[C_{34}H_{51}N_3O_7 + Na]^+$: 636.3619, found: 636.3634. LC-MS (t_R= 3.17 min, λ = 210 nm, purity 97%).

Cheminformatic analysis of medium ring library.

Principal component analysis (PCA)

To generate the plots shown in **Fig. 6a–c** of the manuscript and **Supplementary Fig. 4**, a total of 145 compounds were compared by principal component analysis (PCA):

- 25 top steroid drugs by prescriptions and retail sales in 2016 (**Supp. Fig. 17**)
- 40 top selling small molecule drugs in 2016 without including steroid drugs (**Supp. Fig. 18**)
- 25 steroid and terpenoid natural products with diverse oxidative stage and skeleton (**Supp. Fig. 19**). Notes: The first seven compounds are starting materials we used for the preparation of our library. The next six compounds are steroids with different oxidative stage. These oxidative steroids were included because the increase of oxidative stage is the key diversity element in the first phase of our library synthesis. The remaining 12 terpenoid natural products represent skeleton diversity of steroid analogues because increase of skeleton diversity is the key element in the second phase of our library synthesis. In summary, the set of 25 steroid and terpenoid natural products was selected to represent "oxidative stage diversity" and "skeleton diversity".
- 25 medium-sized ring natural products (**Supp. Fig. 20**). Notes: The 25 diverse natural products with a medium-sized ring represent medium-sized ring natural products with 7-11 membered *N*-heterocyclic, *O*-heterocyclic and carbocyclic rings with skeleton and functional group diversity.
- 30 medium-sized ring library members (**Supp. Fig. 3**). Notes: The structures presented in **Supp. Fig. 3** and **Supp. Fig. 17-20** are those used for computing the descriptors.

A set of 20 physicochemical properties (**Supplementary Table 1**) for all 145 compounds, were calculated using the Molecular Operating Environment (v2016.08, Chemical Computing Group, Montreal). The mean average value for each parameter was calculated for each compound series (**Supplementary Table 2**). This hypothetical average structure for each series was also included in the PCA analysis.

The MOE descriptor names are given in the table. (**Supplementary Table 1**) Descriptors that are not explicitly part of the MOE Descriptor Calculation mode are all easily calculated from MOE SVL functions (aInRing, aInHRing, R and S from aRSChirality) or from combinations of other descriptor values (deltaRS, fChiralMW and fArRing).

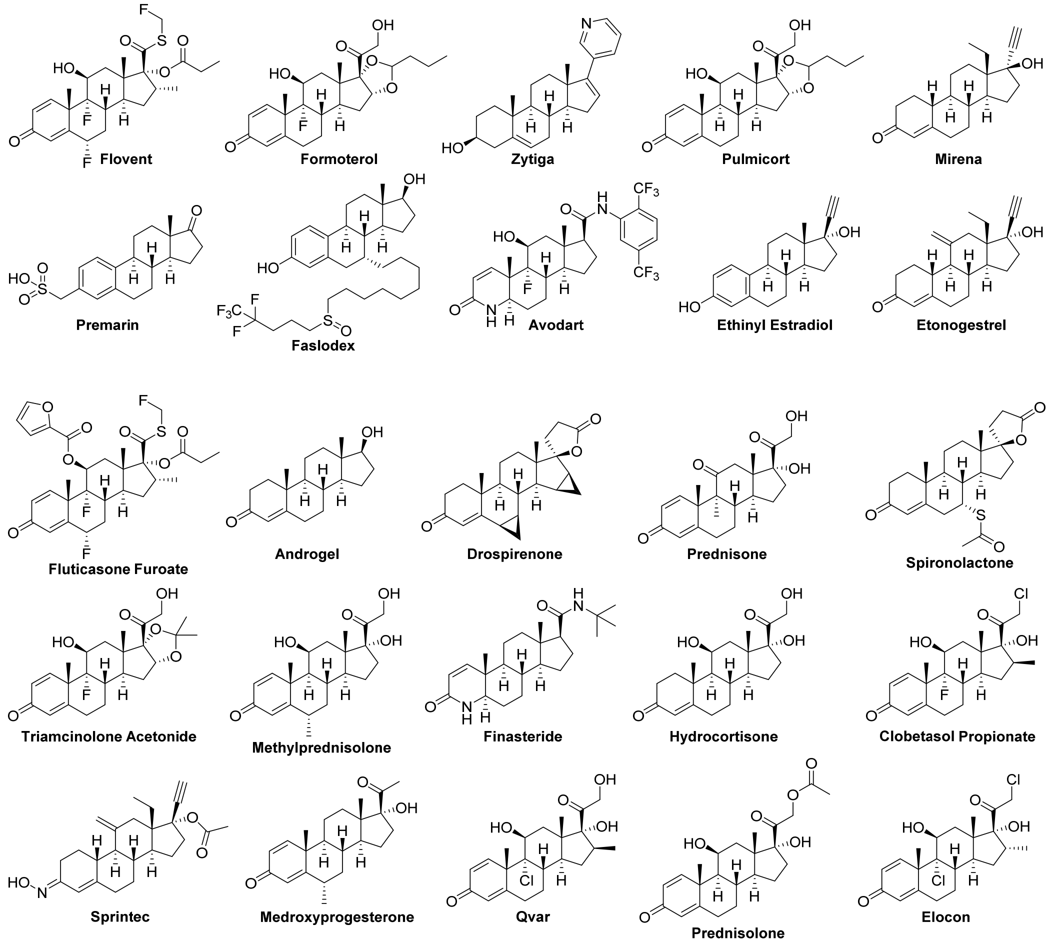

Supplementary Figure 17. Steroid drug reference set for PCA and PMI analyses (25 structures, top steroid drugs by prescriptions and retail sales in 2016).

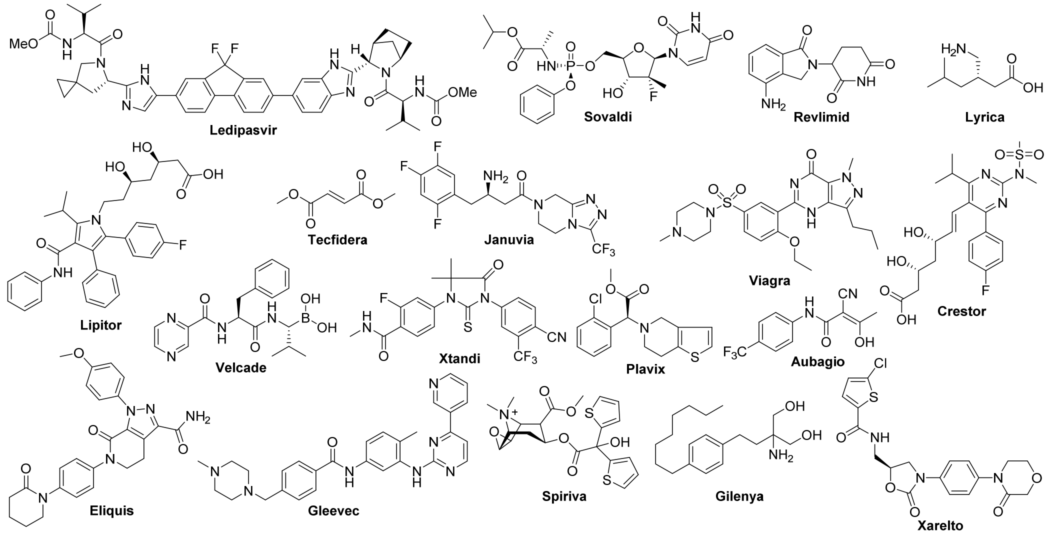

Supplementary Figure 18. Top pharmaceutical products reference set for PCA and PMI analyses by retail sales in 2016 (steroid drugs were not included here) structures are shown in

no particular order. (40 structures). (continued on next page)

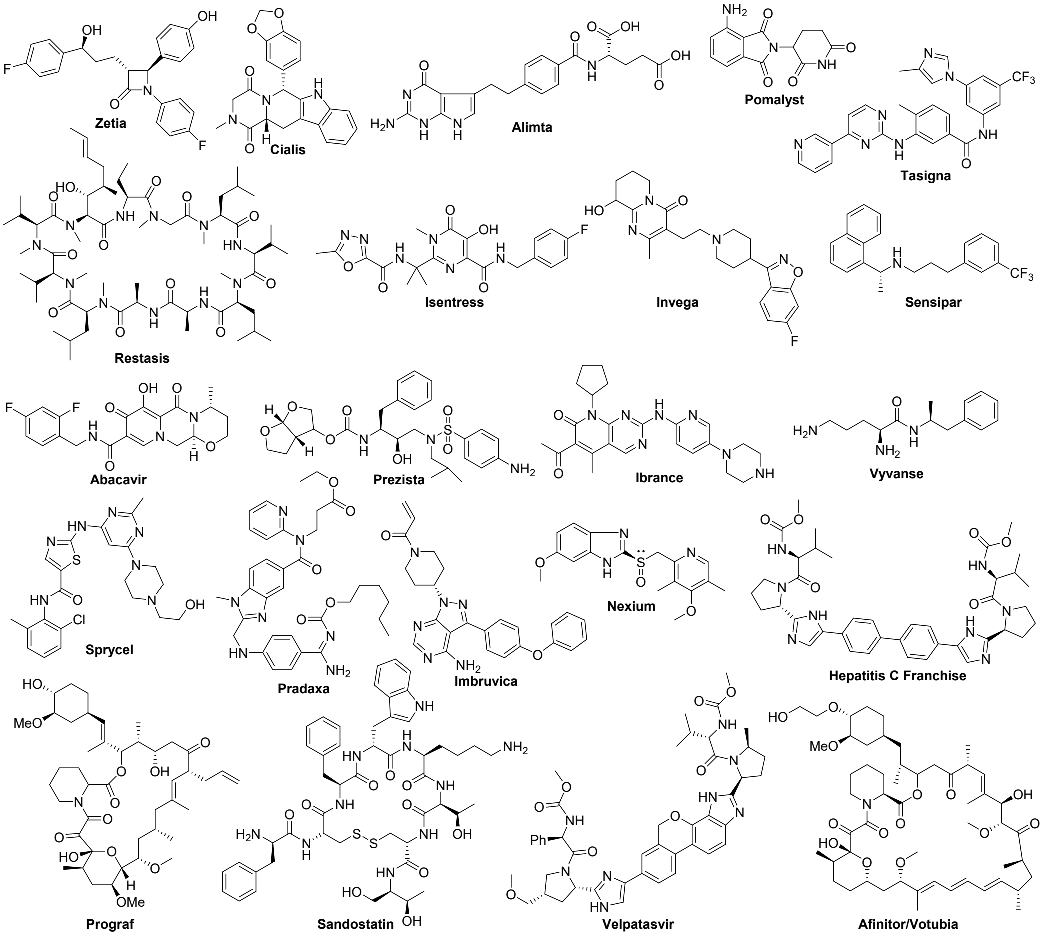

Supplementary Figure 18 (continued). Top pharmaceutical products reference set for PCA and PMI analyses by retail sales in 2016 (steroid drugs were not included here) structures are shown in no particular order. (40 structures).

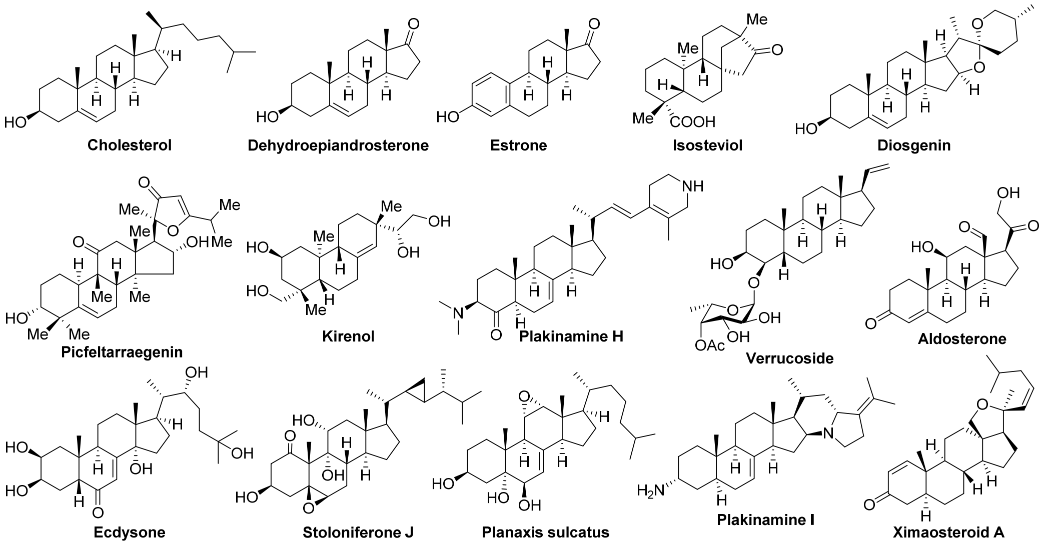

Supplementary Figure 19. Steroid and terpenoid natural products reference set with oxidative stage diversity and skeleton diversity for PCA and PMI (25 structures). (continued on next

page)

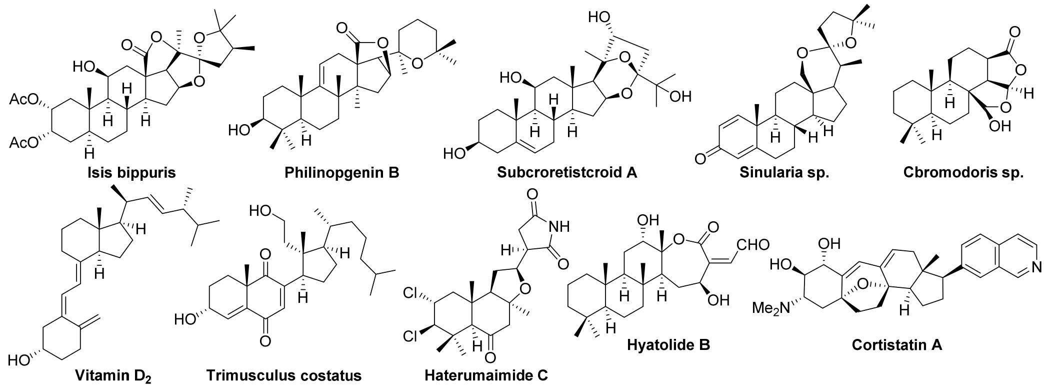

Supplementary Figure 19 (continued). Steroid and terpenoid natural products reference set with oxidative stage diversity and skeleton diversity for PCA and PMI (25 structures).

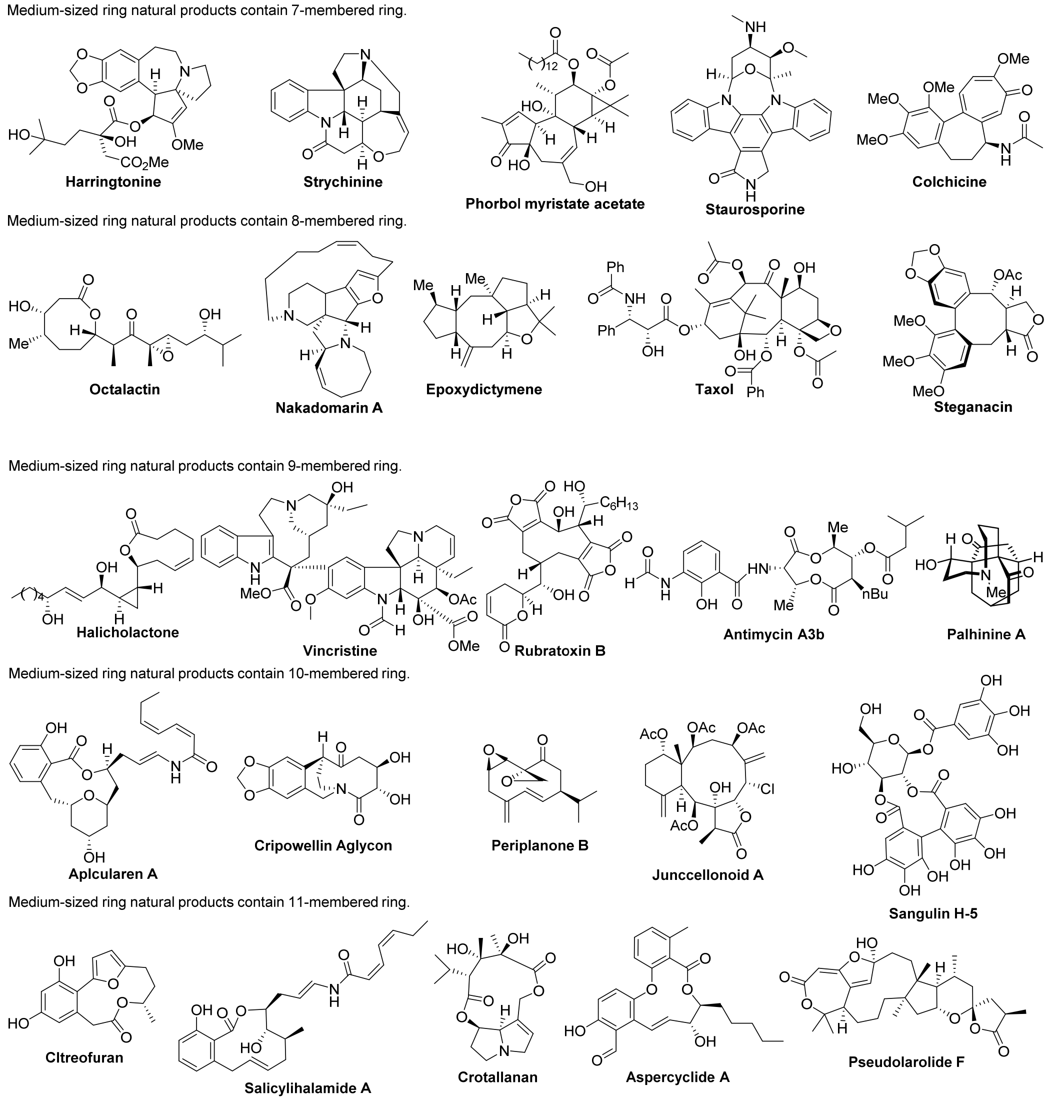

Supplementary Figure 20. Medium ring natural products used in PCA and PMI analyses. (25

structures).

Supplementary Table 1. Parameters employed in PCA.

Property	Description
a_acc	H-Bond Acceptors
a_don	H-Bond Donors
a_nN	Number of N atoms
a_nO	Number of O atoms
b_rotN	Number of rotatable bonds
b_rotR	Fraction of rotatable bonds
chiral	Number of chiral centers
logS	Solubility
opr_nring	OpreaRingCount
rings	Number of Rings
SlogP	LogP
TPSA	Polar Surface Area
Weight	MW
aRing	Number of Atoms in rings
aArRing	Number of Atoms in Aromatic rings
fArRing	aInHRing/aInRing
fChiralWT	chiral/Weight
R	Number of R centers
S	Number of S centers
deltaRS	R-S

Supplementary Table 2. Average parameters by compound series.

AVG	Ster Drugs	Drugs	Med NPs	Ster NPs	Med Lib
**a_acc**	3.64	5.725	5.52	3.64	4.066667
**a_don**	1.76	2.85	2.12	1.8	1.2
**a_nN**	0.32	4.175	0.88	0.28	1.433333
**a_nO**	3.84	4.575	6.76	3.68	4.966667
**b_rotN**	3.36	8.3	5.32	2.52	5.033333
**b_rotR**	0.099261	0.23118	0.134602	0.073338	0.115841
**chiral**	6.92	2.7	5.24	8.76	7.433333
**logS**	-5.03341	-5.12016	-4.73232	-6.05492	-6.59984
**opr_nring**	4.28	3.6	3.64	4.8	4.933333
**rings**	4.28	3.7	4.28	4.8	5.133333
**SlogP**	3.801621	3.208221	2.96813	4.609051	4.935359
**TPSA**	70.8444	119.0815	106.9536	63.5132	84.84667
**Weight**	401.2981	496.0485	456.3872	416.4329	495.5132
**aRing**	18.12	21.05	20.36	20.16	24.16667
**aArRing**	1.88	12.45	6.12	0.64	3.366667

**fArRing**	0.112313	0.620504	0.265271	0.027911	0.128273
**fChiralWT**	0.017493	0.004341	0.011749	0.020835	0.015097
**R**	2.24	1.325	2.72	4.4	2.966667
**S**	4.72	1.475	2.84	4.4	4.466667
**deltaRS**	-2.48	-0.15	-0.12	0	-1.5

Principal component analysis was then carried out using the procedure outlined by Tan.[11] This resulted in the construction of 3 plots of PC1/PC2, PC1/PC3 and PC2/PC3 (**Supplementary Fig. 4a-c**). Summary information from R (the open source statistical computing package used for the PCA analysis) is shown below in **Supplementary Table 3**.

Supplementary Table 3. Standard deviation and proportion of variance for each component in PCA plot (R Summary).

	PC1	PC2	PC3	PC4	PC5	PC6	PC7	PC8	PC9	PC10
Standard Deviation	2.4343	2.3637	1.6974	1.35545	1.2431	0.77439	0.74009	0.63951	0.48702	0.3564
Proportion of Variance	0.2963	0.2794	0.1441	0.09186	0.07727	0.02998	0.02739	0.02045	0.01186	0.00635
Cumulative Proportion	0.2963	0.5756	0.7197	0.81157	0.88883	0.91882	0.9462	0.96665	0.97851	0.98486

This data shows that of the 20-dimensional dataset, >90% of the variance is accounted for within the first 6 principal components (PC1 – PC6). In order to simplify the interpretation of this data, the first 3 principal components (accounting for ~72% of the variation) were used to generate the PCA plots shown in **Fig. 6a-c**.

Supplementary Table 4. Component loadings for PCA of each of the 20 structural and physiochemical descriptors for the first 6 principal components (PC1 – PC6).

	PC1	PC2	PC3	PC4	PC5	PC6
**a_acc**	0.329798	-0.0671072	0.25438635	0.12750934	0.15369497	0.18305368
**a_don**	0.27912506	0.01921988	0.22174865	0.0467632	0.07960758	-0.6951455
**a_nN**	0.29685025	0.10371903	-0.1720181	0.11478516	0.01020065	-0.2543469
**a_nO**	0.24166884	-0.1485845	0.34151278	0.08036167	0.06197324	0.45357125
**b_rotN**	0.33685851	0.02880172	0.03279327	-0.2374038	-0.3028305	-0.0331336
**b_rotR**	0.20130238	0.19836261	0.1018756	-0.3089116	-0.3477849	-0.0393965
**chiral**	-0.0268736	-0.3910315	0.16855769	-0.0947218	-0.0520732	-0.1381713
**logS**	-0.1541052	0.2253012	0.22977875	0.28438894	0.28198913	-0.0457574
**opr_nring**	0.06139484	-0.2678652	-0.351451	0.15069489	0.18582394	-0.0670989
**rings**	0.08563166	-0.2907151	-0.318923	0.17219697	0.20403389	-0.0043174
**SlogP**	0.02182947	-0.1969173	-0.3718897	-0.2970161	-0.288737	0.07523319
**TPSA**	0.35559281	-0.0396474	0.2549833	0.11194292	0.04651661	0.02015902
**Weight**	0.33488006	-0.216455	0.02423513	-0.0466049	-0.098654	0.12459598
**aRing**	0.23714719	-0.2628607	-0.209957	0.14602618	0.15405494	0.12268112
**aArRing**	0.29173449	0.15838578	-0.2862231	0.09450528	0.10563285	-0.0904635
**fArRing**	0.22072837	0.27646334	-0.199584	0.0132127	0.03751628	-0.0747786
**fChiralWT**	-0.20883	-0.3226519	0.1355699	-0.0394227	0.00354747	-0.3336323
**R**	0.00753151	-0.2896045	0.13412887	-0.4190554	0.28920759	-0.0931565

**S**	-0.0471995	-0.3361565	0.12603017	0.24248684	-0.3317587	-0.1159127
**deltaRS**	0.04689182	0.05822877	-0.0013082	-0.5464534	0.51938778	0.02545369

Top contributing parameters to each principal component are marked in grey. The values were normalized automatically by the MOE software.

Principal Moment of Inertia Analysis

Principal moment of inertia analysis was carried out by calculation of the lowest energy conformation of each representative scaffold and library compound, and each compound from the above reference set. The conformation calculation was performed using the MOE molecular modelling software package7172 in a similar manner to that reported by Tan.[11]

The parameters used for conformer generation are listed below:

□ ● maxConfs: 1000
□ ● RMSD: ≤0.15
□ ● Failure limit: 100
□ ● Energy cutoff: 7 kcal/mol
□ ● Iteration limit: 1000
□ ● MM iteration limit: 500

Once the lowest energy conformer was calculated, the three principal moments of inertia (Ixx, Iyy, Izz) and normalized principal moments of inertia, npr1 (Ixx/Izz) and npr2 (Iyy/Izz) were determined using MOE. These PMI ratios were calculated for our representative scaffolds and library members, in addition to the reference sets drugs, steroid drugs steroid natural products and medium-sized ring natural products. The ratios were plotted on a triangular graph where the vertices (0,1), (0.5,0.5) and (1,1) represent a perfect rod, disc and sphere, respectively (**Fig. 6d**).

X-Ray report.

MOLECULAR STRUCTURE LABORATORY

MICHAEL M. ARISTOV, ILIA A. GUZEI, PH.D.

University of Wisconsin-Madison ☎ 608-263-4694
2124 Chemistry Department Fax 608-262-0381
1101 University Ave
Madison, WI 53706 E-mail: iguzei@chem.wisc.edu

Structural report on **4a**

(CCDC 1888660)

MARCH 14, 2018

Crystallographic Experimental Section

Data Collection

A colorless crystal with approximate dimensions 0.365 x 0.254 x 0.219 mm^3 was selected under oil under ambient conditions and attached to the tip of a MiTeGen MicroMount©. The crystal was mounted in a stream of cold nitrogen at 200(1) K and centered in the X-ray beam by using a video camera.

The crystal evaluation and data collection were performed on a Bruker SMART APEXII diffractometer with Cu K_α (λ = 1.54178 Å) radiation and the diffractometer to crystal distance of 4.03 cm [12].

The initial cell constants were obtained from three series of ω scans at different starting angles. Each series consisted of 41 frames collected at intervals of 0.6º in a 25º range about ω with the exposure time of 10 seconds per frame. The reflections were successfully indexed by an automated indexing routine built in the APEXII program. The final cell constants were calculated

from a set of 9982 strong reflections from the actual data collection.

The data were collected by using the full sphere data collection routine to survey the reciprocal space to the extent of a full sphere to a resolution of 0.82 Å. A total of 45302 data were harvested by collecting 14 sets of frames with 0.6º scans in ω and φ with an exposure time 8-16 sec per frame. These highly redundant datasets were corrected for Lorentz and polarization effects. The absorption correction was based on fitting a function to the empirical transmission surface as sampled by multiple equivalent measurements [13].

Structure Solution and Refinement

The systematic absences in the diffraction data were consistent for the space groups $P3_1$, $P3_2$, $P3_121$, and $P3_221$. Space group $P3_221$ was chosen because it had the lowest figure of merit, yielded a chemically reasonable structure with the correct absolute configuration, and produced computationally stable results of refinement [14-19].

A successful solution by the direct methods provided most non-hydrogen atoms from the *E*-map. The remaining non-hydrogen atoms were located in an alternating series of least-squares cycles and difference Fourier maps. All non-hydrogen atoms were refined with anisotropic displacement coefficients. All hydrogen atoms were included in the structure factor calculation at idealized positions and were allowed to ride on the neighboring atoms with relative isotropic displacement coefficients.

The absolute structure was unequivocally established by anomalous scattering. A total of eight stereocenter were found with C6 and C19 in the *R* conformation with C3, C7, C10, C11, C15, and C16 in the *S* conformation.

The final least-squares refinement of 275 parameters against 4633 data resulted in residuals *R* (based on F^2 for $I \geq 2\sigma$) and *wR* (based on F^2 for all data) of 0.0401 and 0.1076, respectively. The final difference Fourier map was featureless.

Summary

Crystal Data for $C_{25}H_{39}NO_4$ (M =417.57 g/mol): trigonal, space group $P3_221$ (no. 154), a = 11.3911(9) Å, c = 31.049(6) Å, V = 3489.0(9) Å^3, Z = 6, T = 200.0 K, μ(CuKα) = 0.630 mm^{-1}, $Dcalc$ = 1.192 g/cm^3, 45302 reflections measured (8.544° ≤ 2Θ ≤ 146.81°), 4633 unique (R_{int} = 0.0286, R_{sigma} = 0.0125) which were used in all calculations. The final R_1 was 0.0401 (I > 2σ(I)) and wR_2 was 0.1076 (all data).

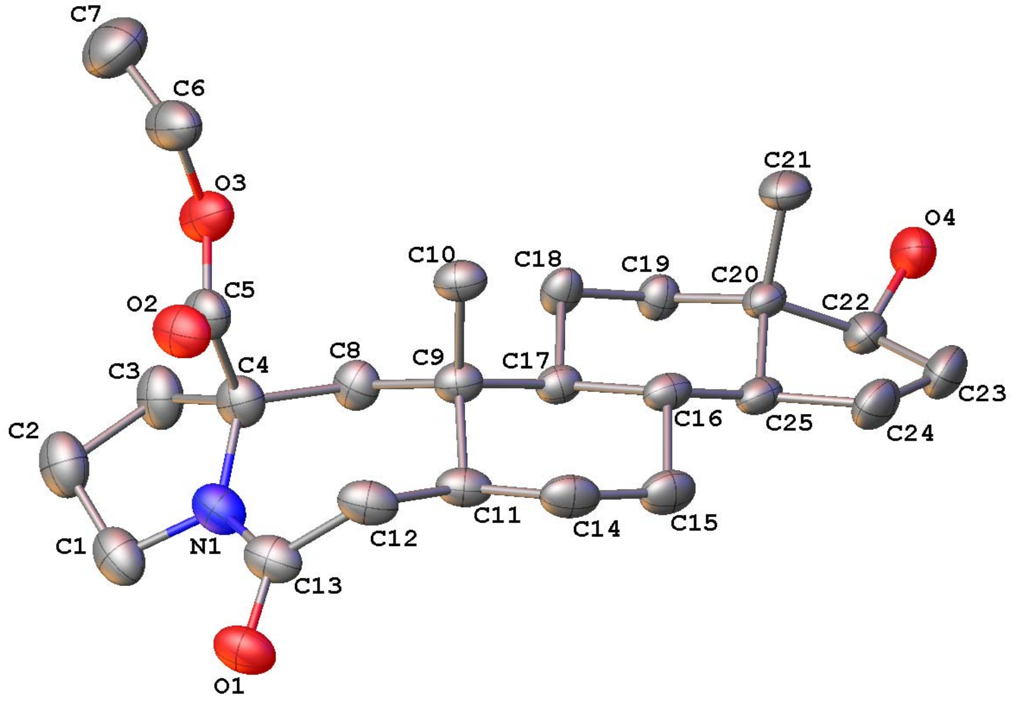

Supplementary Figure 21. A molecular drawing of **4a** shown with 30% probability ellipsoids. All H atoms are omitted.

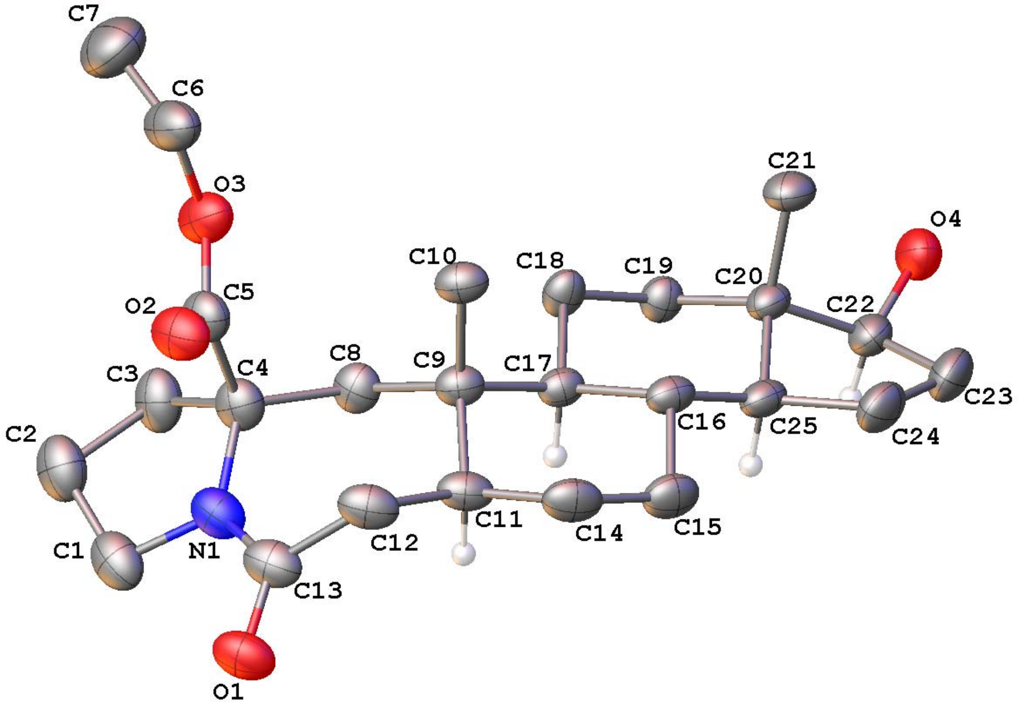

Supplementary Figure 22. A molecular drawing of **4a** shown with 30% probability ellipsoids. All H atoms other than those attached to stereocenter are omitted.

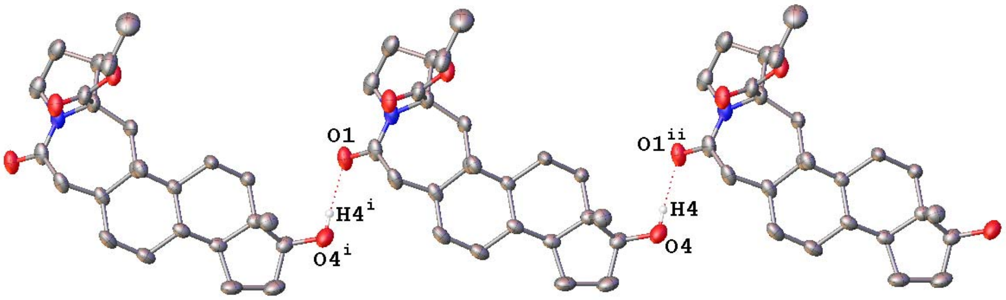

Supplementary Figure 23. Hydrogen bonding interactions in **4a**. The molecules are shown with 30% probability ellipsoids. H atoms not involved in the hydrogen bonding network are omitted. [Symmetry codes: (i) 1+x, 1+y, +z; (ii) -1+x, -1+y, +z.]

Supplementary Table 5. Crystal data and structure refinement for **4a**.

Identification code	**4a**
Empirical formula	$C_{25}H_{39}NO_4$
Formula weight	417.57
Temperature/K	200.0
Crystal system	trigonal
Space group	$P3_221$
a/Å	11.3911(9)
b/Å	11.3911(9)
c/Å	31.049(6)
α/°	90
β/°	90
γ/°	120
Volume/Å^3	3489.0(9)
Z	6
ρ_{calc}g/cm^3	1.192
μ/mm^{-1}	0.630
F(000)	1368.0
Crystal size/mm^3	0.46 × 0.24 × 0.21
Radiation	CuKα (λ = 1.54178)
2Θ range for data collection/°	8.544 to 146.81
Index ranges	$-14 \leq h \leq 14$, $-13 \leq k \leq 14$, $-32 \leq l \leq 36$
Reflections collected	45302
Independent reflections	4633 [R_{int} = 0.0286, R_{sigma} = 0.0125]
Data/restraints/parameters	4633/0/275
Goodness-of-fit on F^2	1.030

Final R indexes [I>=2σ (I)]	R_1 = 0.0401, wR_2 = 0.1048
Final R indexes [all data]	R_1 = 0.0430, wR_2 = 0.1076
Largest diff. peak/hole / e Å^{-3}	0.16/-0.16
Flack parameter	-0.02(5)

Supplementary Table 6. Fractional Atomic Coordinates (×10^4) and Equivalent Isotropic Displacement Parameters (Å^2×10^3) for **4a**. U_{eq} is defined as 1/3 of of the trace of the orthogonalised U_{IJ} tensor.

**Atom**	***x***	***y***	***z***	**U(eq)**
O1	11572.8(19)	5756.8(18)	5717.4(7)	78.6(5)
O2	10210(2)	4551.7(19)	6925.0(6)	78.5(5)
O3	8874(2)	2371(2)	7108.1(6)	80.6(6)
O4	967.0(19)	-2362(2)	5345.1(6)	80.1(6)
N1	10663(2)	3864(2)	6112.4(7)	65.6(5)
C1	10615(3)	4922(2)	5938.7(8)	66.1(7)
C2	9375(3)	5032(2)	6010.4(8)	65.0(6)
C3	8084(3)	3892(2)	5799.8(7)	56.9(6)
C4	7186(3)	4451(2)	5647.2(8)	67.1(7)
C5	5961(3)	3410(2)	5400.2(8)	67.2(6)
C6	5138(2)	2084(2)	5654.1(7)	51.7(5)
C7	4007(2)	1014(2)	5383.3(6)	54.0(5)
C8	2941(3)	1306(3)	5189.7(9)	77.9(8)
C9	1778(3)	-98(4)	5060.1(9)	84.2(9)
C10	2143(3)	-1135(3)	5237.9(7)	62.1(6)
C11	3139(2)	-362(2)	5607.0(6)	50.5(5)
C12	2345(3)	-313(3)	5998.0(7)	66.1(6)
C13	4082(2)	-889(2)	5736.4(7)	53.1(5)
C14	5242(2)	144(2)	6020.9(7)	54.0(5)
C15	6073(2)	1548(2)	5807.0(6)	47.5(4)
C16	7296(2)	2604(2)	6082.1(7)	50.7(5)
C17	6786(3)	2912(2)	6502.3(7)	57.5(5)
C18	8205(2)	1986(2)	6177.7(8)	57.5(5)
C19	9629(2)	2791(2)	6384.8(9)	63.4(6)
C20	9614(2)	3369(3)	6831.9(9)	66.6(6)
C21	8791(3)	2760(4)	7552.1(9)	92.4(10)
C22	8727(5)	1667(5)	7838.2(12)	123.2(15)
C23	10217(3)	1834(3)	6435.2(11)	77.0(8)

C24	11750(3)	2786(3)	6428.8(11)	84.7(9)
C25	11910(3)	3789(3)	6086.9(10)	76.7(8)

Supplementary Table 7. Anisotropic Displacement Parameters (Å^2×10^3) for **4a**. The Anisotropic displacement factor exponent takes the form: $-2\pi^2[h^2a^{*2}U_{11}+2hka^*b^*U_{12}+\ldots]$.

Atom	U_{11}	U_{22}	U_{33}	U_{23}	U_{13}	U_{12}
O1	68.3(11)	54.1(9)	87.1(12)	-4.0(9)	18.8(9)	10.9(8)
O2	71.0(11)	59.9(10)	77.3(11)	-18.8(8)	-1.3(9)	12.4(9)
O3	69.4(11)	68.4(11)	68.1(11)	-2.7(9)	-9.4(9)	7.6(9)
O4	59.4(10)	92.4(14)	70.8(11)	5.3(10)	-10.4(8)	24.7(10)
N1	58.6(11)	48(1)	78.0(14)	-15.5(9)	4.4(10)	17.4(9)
C1	65.8(14)	43.2(11)	70.4(15)	-17.7(10)	8.8(12)	13(1)
C2	76.4(15)	36.4(10)	71.2(14)	-2.5(9)	18.9(12)	20.0(11)
C3	70.4(14)	40.9(10)	55.5(12)	2.6(9)	19(1)	24.9(10)
C4	87.6(18)	46.1(11)	69.5(14)	17.1(10)	22.4(13)	34.9(12)
C5	90.0(18)	59.6(13)	61.9(13)	18.4(11)	12.0(12)	44.8(13)
C6	69.4(13)	52.9(11)	42.9(10)	8.4(8)	10.4(9)	38.2(10)
C7	68.0(13)	65.2(13)	38.4(10)	9.4(9)	7.1(9)	40.6(11)
C8	91(2)	97(2)	63.2(14)	19.0(14)	-4.7(14)	60.2(18)
C9	85.7(19)	117(2)	61.5(15)	12.1(15)	-14.2(14)	59.3(19)
C10	59.4(13)	80.9(16)	43.9(11)	3.5(10)	-1.8(9)	33.4(12)
C11	58.4(12)	60.8(12)	36.6(10)	3.4(8)	3.0(8)	33.1(10)
C12	72.9(15)	80.0(16)	46.5(12)	5.0(11)	12.1(11)	39.1(13)
C13	55.2(12)	45.3(10)	56.4(12)	2.3(9)	-5.9(9)	23.5(9)
C14	58.1(12)	39.6(10)	59.0(12)	5.9(8)	-9.9(10)	20.4(9)
C15	59.1(11)	42.3(10)	42.7(10)	2.8(8)	4.1(8)	26.5(9)
C16	60.5(12)	37.6(9)	49.4(11)	-0.5(8)	5.1(9)	21.1(9)
C17	68.6(14)	43.9(10)	49.9(11)	-0.6(8)	7.6(10)	20.6(10)
C18	58.8(12)	38.5(10)	65.5(13)	-8.5(9)	-8.6(10)	17.1(9)
C19	57.9(13)	43.5(11)	74.6(15)	-9.5(10)	-7.1(11)	14.7(10)
C20	52.1(12)	56.9(13)	70.7(15)	-9.5(11)	-7.7(11)	12.2(11)
C21	68.1(16)	98(2)	69.8(17)	-6.7(16)	-3.5(13)	10.3(16)
C22	132(4)	142(4)	79(2)	4(2)	-14(2)	55(3)
C23	65.8(15)	57.3(13)	101(2)	-20.2(13)	-24.7(14)	25.4(12)
C24	67.4(16)	70.7(17)	108(2)	-29.6(16)	-23.2(15)	28.3(14)
C25	59.0(14)	68.8(16)	90.2(18)	-31.4(15)	-7.4(13)	22.9(12)

Supplementary Table 8. Bond Lengths for **4a**.

Atom	Atom	Length/Å	Atom	Atom	Length/Å
O1	C1	1.237(3)	C7	C11	1.539(3)
O2	C20	1.202(3)	C8	C9	1.535(5)
O3	C20	1.334(3)	C9	C10	1.537(4)
O3	C21	1.466(4)	C10	C11	1.542(3)
O4	C10	1.409(3)	C11	C12	1.531(3)
N1	C1	1.346(4)	C11	C13	1.524(3)
N1	C19	1.468(3)	C13	C14	1.535(3)
N1	C25	1.468(4)	C14	C15	1.543(3)
C1	C2	1.496(4)	C15	C16	1.563(3)
C2	C3	1.538(4)	C16	C17	1.539(3)
C3	C4	1.526(4)	C16	C18	1.544(3)
C3	C16	1.552(3)	C18	C19	1.548(3)
C4	C5	1.512(4)	C19	C20	1.540(4)
C5	C6	1.538(3)	C19	C23	1.546(4)
C6	C7	1.510(3)	C21	C22	1.501(6)
C6	C15	1.544(3)	C23	C24	1.528(4)
C7	C8	1.532(3)	C24	C25	1.502(5)

Supplementary Table 9. Bond Angles for **4a**.

Atom	Atom	Atom	Angle/°	Atom	Atom	Atom	Angle/°
C20	O3	C21	117.1(2)	C13	C11	C10	115.65(19)
C1	N1	C19	127.0(2)	C13	C11	C12	110.53(18)
C1	N1	C25	120.3(2)	C11	C13	C14	110.82(18)
C25	N1	C19	112.3(2)	C13	C14	C15	113.20(17)
O1	C1	N1	119.7(3)	C6	C15	C16	112.97(17)
O1	C1	C2	121.7(3)	C14	C15	C6	110.77(18)
N1	C1	C2	118.6(2)	C14	C15	C16	114.29(16)
C1	C2	C3	114.22(19)	C3	C16	C15	106.88(17)
C2	C3	C16	114.5(2)	C17	C16	C3	111.65(16)
C4	C3	C2	109.71(19)	C17	C16	C15	110.23(18)
C4	C3	C16	112.25(19)	C17	C16	C18	110.90(19)
C5	C4	C3	112.24(19)	C18	C16	C3	109.70(18)

C4	C5	C6	112.38(19)	C18	C16	C15	107.31(16)
C5	C6	C15	110.07(19)	C16	C18	C19	123.56(18)
C7	C6	C5	111.23(17)	N1	C19	C18	114.8(2)
C7	C6	C15	109.38(17)	N1	C19	C20	109.82(18)
C6	C7	C8	119.5(2)	N1	C19	C23	101.3(2)
C6	C7	C11	115.13(16)	C20	C19	C18	113.3(2)
C8	C7	C11	102.9(2)	C20	C19	C23	108.1(2)
C7	C8	C9	104.0(2)	C23	C19	C18	108.62(18)
C8	C9	C10	106.4(2)	O2	C20	O3	124.3(2)
O4	C10	C9	111.0(2)	O2	C20	C19	125.1(2)
O4	C10	C11	117.01(19)	O3	C20	C19	110.6(2)
C9	C10	C11	103.4(2)	O3	C21	C22	106.8(3)
C7	C11	C10	99.26(16)	C24	C23	C19	104.1(2)
C12	C11	C7	114.30(19)	C25	C24	C23	102.1(2)
C12	C11	C10	109.3(2)	N1	C25	C24	104.7(2)
C13	C11	C7	107.47(18)				

Supplementary Table 10. Torsion Angles for **4a**.

A	B	C	D	Angle/°	A	B	C	D	Angle/°
O1	C1	C2	C3	112.8(2)	C8	C7	C11	C13	169.05(19)
O4	C10	C11	C7	-165.6(2)	C8	C9	C10	O4	149.0(2)
O4	C10	C11	C12	-45.7(3)	C8	C9	C10	C11	22.7(3)
O4	C10	C11	C13	79.8(3)	C9	C10	C11	C7	-43.2(2)
N1	C1	C2	C3	-65.5(3)	C9	C10	C11	C12	76.7(2)
N1	C19	C20	O2	10.1(4)	C9	C10	C11	C13	-157.8(2)
N1	C19	C20	O3	-168.2(2)	C10	C11	C13	C14	165.95(19)
N1	C19	C23	C24	32.5(3)	C11	C7	C8	C9	-34.8(3)
C1	N1	C19	C18	57.6(3)	C11	C13	C14	C15	-56.5(3)
C1	N1	C19	C20	-71.4(3)	C12	C11	C13	C14	-69.2(2)
C1	N1	C19	C23	174.4(2)	C13	C14	C15	C6	53.3(2)
C1	N1	C25	C24	161.5(2)	C13	C14	C15	C16	-177.69(18)
C1	C2	C3	C4	-145.6(2)	C14	C15	C16	C3	174.65(18)
C1	C2	C3	C16	87.1(3)	C14	C15	C16	C17	-63.8(2)
C2	C3	C4	C5	174.68(19)	C14	C15	C16	C18	57.0(2)
C2	C3	C16	C15	-177.23(18)	C15	C6	C7	C8	-179.62(19)
C2	C3	C16	C17	62.2(3)	C15	C6	C7	C11	57.1(2)

C2 C3 C16 C18 -61.2(2)	C15 C16 C18 C19 170.7(2)
C3 C4 C5 C6 53.7(3)	C16 C3 C4 C5 -56.8(3)
C3 C16 C18 C19 54.9(3)	C16 C18 C19 N1 -68.2(3)
C4 C3 C16 C15 56.8(2)	C16 C18 C19 C20 59.1(3)
C4 C3 C16 C17 -63.8(3)	C16 C18 C19 C23 179.3(2)
C4 C3 C16 C18 172.87(19)	C17 C16 C18 C19 -68.8(3)
C4 C5 C6 C7 -174.2(2)	C18 C19 C20 O2 -119.8(3)
C4 C5 C6 C15 -52.8(3)	C18 C19 C20 O3 61.9(3)
C5 C6 C7 C8 -57.8(3)	C18 C19 C23 C24 153.8(2)
C5 C6 C7 C11 178.93(19)	C19 N1 C1 O1 179.4(2)
C5 C6 C15 C14 -174.10(18)	C19 N1 C1 C2 -2.2(3)
C5 C6 C15 C16 56.2(2)	C19 N1 C25 C24 -11.2(3)
C6 C7 C8 C9 -163.8(2)	C19 C23 C24 C25 -39.9(3)
C6 C7 C11 C10 -179.98(19)	C20 O3 C21 C22 -145.6(3)
C6 C7 C11 C12 63.8(3)	C20 C19 C23 C24 -82.9(3)
C6 C7 C11 C13 -59.2(2)	C21 O3 C20 O2 0.2(4)
C6 C15 C16 C3 -57.5(2)	C21 O3 C20 C19 178.5(2)
C6 C15 C16 C17 64.0(2)	C23 C19 C20 O2 119.8(3)
C6 C15 C16 C18 -175.10(17)	C23 C19 C20 O3 -58.5(3)
C7 C6 C15 C14 -51.6(2)	C23 C24 C25 N1 31.2(3)
C7 C6 C15 C16 178.71(16)	C25 N1 C1 O1 7.8(3)
C7 C8 C9 C10 7.3(3)	C25 N1 C1 C2 -173.9(2)
C7 C11 C13 C14 56.2(2)	C25 N1 C19 C18 -130.2(2)
C8 C7 C11 C10 48.3(2)	C25 N1 C19 C20 100.8(2)
C8 C7 C11 C12 -67.9(2)	C25 N1 C19 C23 -13.4(2)

Supplementary Table 11. Hydrogen Atom Coordinates (Å×10^4) and Isotropic Displacement Parameters (Å^2×10^3) for **4a**.

**Atom**	***x***	***y***	***z***	**U(eq)**
H4	1188.8	-2889.22	5460.84	120
H2A	9541.53	5913.04	5895.46	78
H2B	9218.59	5027.52	6324.17	78
H3	8380.93	3608.36	5535.86	68
H4A	6878.96	4755.74	5899.99	80
H4B	7723.85	5248.68	5460.66	80
H5A	6263.8	3209.3	5124.93	81

H5B	5370.72	3791.77	5331.43	81
H6	4733.48	2271.53	5912.41	62
H7	4453.78	838.85	5134.06	65
H8A	3306.62	1907.22	4935.6	93
H8B	2633.02	1735.64	5404.59	93
H9A	1686.51	-172.64	4742.8	101
H9B	911.35	-255.54	5184.58	101
H10	2643.96	-1325.86	5009.39	75
H12A	2978.11	314.47	6212.8	99
H12B	1827.26	-1221.11	6124.07	99
H12C	1721.53	-2.4	5907.5	99
H13A	4462.71	-1069.3	5474.5	64
H13B	3561.64	-1753.04	5895.32	64
H14A	5854.91	-210.68	6090.45	65
H14B	4858.84	248.67	6294.9	65
H15	6474.02	1399.22	5540.34	57
H17A	6128.3	3200.44	6434.33	86
H17B	7553.25	3636.72	6658.88	86
H17C	6351.41	2094.17	6681.34	86
H18A	8327.67	1630.83	5900.62	69
H18B	7668.56	1189.72	6364.83	69
H21A	7970.06	2839.13	7590.48	111
H21B	9596.73	3643.62	7622.68	111
H22A	7900.9	807.99	7774.83	185
H22B	8716.12	1910.64	8140.28	185
H22C	9521.71	1568.1	7786.19	185
H23A	9923.4	1329.51	6710.4	92
H23B	9926.63	1177.75	6194.43	92
H24A	12085.69	3234.8	6711.04	102
H24B	12233.47	2296.49	6350.25	102
H25A	12716.23	4685.99	6144.07	92
H25B	12005.95	3473.15	5798.85	92

MOLECULAR STRUCTURE LABORATORY

MICHAEL M. ARISTOV, ILIA A. GUZEI, PH.D.

University of Wisconsin-Madison ☎ 608-263-4694
2124 Chemistry Department Fax 608-262-0381
1101 University Ave
Madison, WI 53706 E-mail: iguzei@chem.wisc.edu

Structural report on S10

(CCDC 1888661)

MARCH 14, 2018

Crystallographic Experimental Section

Data Collection

A colorless needle crystal with approximate dimensions 0.20 x 0.25 x 0.58 mm^3 was selected under oil under ambient conditions and attached to the tip of a MiTeGen MicroMount©. The crystal was mounted in a stream of cold nitrogen at 100(1) K and centered in the X-ray beam by using a video camera.

The crystal evaluation and data collection were performed on a Bruker SMART APEXII diffractometer with Cu K_α (λ = 1.54178 Å) radiation and the diffractometer to crystal distance of 4.03 cm [12].

The initial cell constants were obtained from three series of ω scans at different starting angles. Each series consisted of 35 frames collected at intervals of 0.7º in a 25º range about ω with the exposure time of 3 seconds per frame. The reflections were successfully indexed by an

automated indexing routine built in the APEXII program. The final cell constants were calculated from a set of 9094 strong reflections from the actual data collection.

The data were collected by using the full sphere data collection routine to survey the reciprocal space to the extent of a full sphere to a resolution of 0.80 Å. A total of 31858 data were harvested by collecting 18 sets of frames with 0.6º scans in ω and φ with an exposure time 4-10 sec per frame. These highly redundant datasets were corrected for Lorentz and polarization effects. The absorption correction was based on fitting a function to the empirical transmission surface as sampled by multiple equivalent measurements [13].

Structure Solution and Refinement

The systematic absences in the diffraction data were uniquely consistent for the space group $P2_12_12_1$ that yielded chemically reasonable and computationally stable results of refinement [14-19].

A successful solution by the direct methods provided most non-hydrogen atoms from the *E*-map. The remaining non-hydrogen atoms were located in an alternating series of least-squares cycles and difference Fourier maps. All non-hydrogen atoms were refined with anisotropic displacement coefficients. All hydrogen atoms were included in the structure factor calculation at idealized positions and were allowed to ride on the neighboring atoms with relative isotropic displacement coefficients.

The absolute structure was unequivocally established by anomalous scattering. A total of six stereocenter were found with C2, C6, C8, and C11 in the *R* conformation with C13 and C17 in the *S* conformation.

The final least-squares refinement of 220 parameters against 3497 data resulted in residuals *R* (based on F^2 for $I \geq 2\sigma$) and *wR* (based on F^2 for all data) of 0.0299 and 0.0814, respectively. The final difference Fourier map was featureless.

Summary

Crystal Data for $C_{21}H_{32}O_3$ (M =332.46 g/mol): orthorhombic, space group $P2_12_12_1$ (no. 19), a = 8.2422(3) Å, b = 10.1792(5) Å, c = 20.8464(9) Å, V = 1748.99(13) Å^3, Z = 4, T = 99.97 K, μ(CuKα) = 0.645 mm^{-1}, $Dcalc$ = 1.263 g/cm^3, 31740 reflections measured (8.482° ≤ 2Θ ≤

146.748°), 3497 unique (R_{int} = 0.0198, R_{sigma} = 0.0087) which were used in all calculations. The final R_1 was 0.0299 (I > 2σ(I)) and wR_2 was 0.0814 (all data).

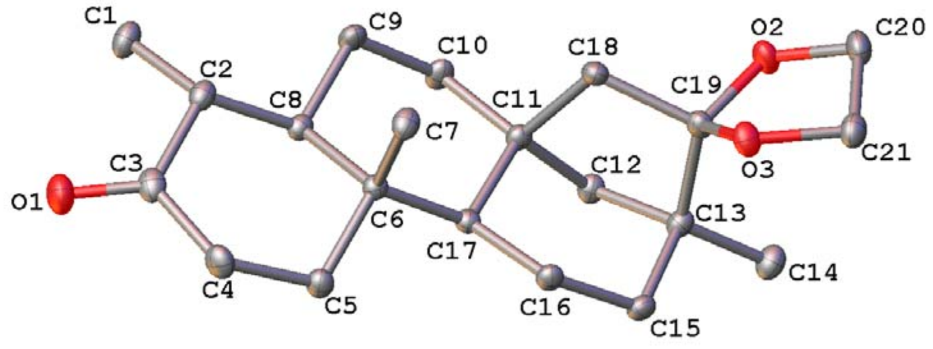

Supplementary Figure 24. A molecular drawing of **S10** shown with 50% probability ellipsoids. All H atoms are omitted for clarity.

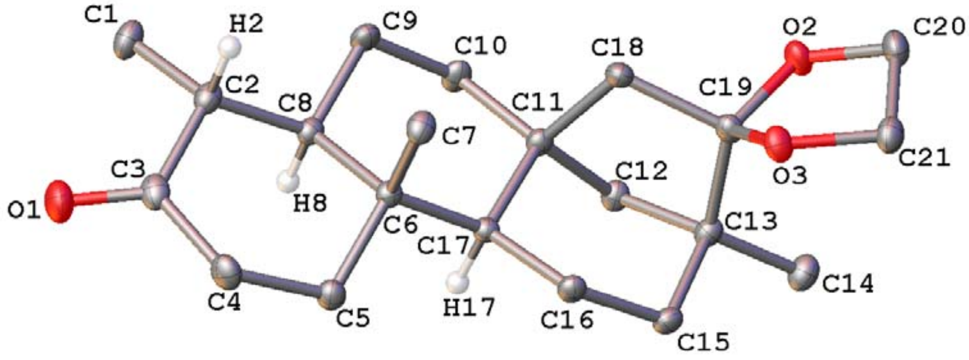

Supplementary Figure 25. A molecular drawing of **S10** shown with 50% probability ellipsoids. All H atoms other than those around stereocenter are omitted for clarity.

Supplementary Table 12. Crystal data and structure refinement for **S10**.

Identification code	tang26
Empirical formula	$C_{21}H_{32}O_3$
Formula weight	332.46
Temperature/K	99.97
Crystal system	orthorhombic
Space group	$P2_12_12_1$
a/Å	8.2422(3)
b/Å	10.1792(5)
c/Å	20.8464(9)
α/°	90

β/°	90
γ/°	90
Volume/Å^3	1748.99(13)
Z	4
ρ_{calc}g/cm^3	1.263
μ/mm^{-1}	0.645
F(000)	728.0
Crystal size/mm^3	0.585 × 0.254 × 0.202
Radiation	CuKα (λ = 1.54178)
2Θ range for data collection/°	8.482 to 146.748
Index ranges	-10 ≤ h ≤ 10, -12 ≤ k ≤ 12, -25 ≤ l ≤ 25
Reflections collected	31740
Independent reflections	3497 [R_{int} = 0.0198, R_{sigma} = 0.0087]
Data/restraints/parameters	3497/0/220
Goodness-of-fit on F^2	1.079
Final R indexes [I>=2σ (I)]	R_1 = 0.0299, wR_2 = 0.0813
Final R indexes [all data]	R_1 = 0.0300, wR_2 = 0.0814
Largest diff. peak/hole / e Å^{-3}	0.26/-0.18
Flack parameter	0.05(2)

Supplementary Table 13. Fractional Atomic Coordinates (×10^4) and Equivalent Isotropic Displacement Parameters (Å^2×10^3) for **S10**. U_{eq} is defined as 1/3 of of the trace of the orthogonalised U_{IJ} tensor.

**Atom**	***x***	***y***	***z***	**U(eq)**
O1	3194.4(16)	11465.4(12)	6650.0(6)	28.1(3)
O2	8159.7(13)	3028.4(10)	5567.2(5)	19.7(2)
O3	9941.9(13)	4742.3(10)	5572.6(5)	18.0(2)
C1	1613.8(19)	9353.4(16)	6110.9(8)	20.6(3)
C2	3471.4(18)	9397.3(14)	6107.3(7)	15.6(3)
C3	4075.0(19)	10560.1(15)	6498.0(7)	17.6(3)
C4	5863(2)	10594.2(14)	6667.6(8)	18.7(3)
C5	6614.0(18)	9254.6(14)	6825.7(7)	15.8(3)
C6	6149.8(17)	8162.4(14)	6348.3(6)	12.1(3)
C7	6835.4(18)	8515.3(15)	5682.3(7)	16.8(3)
C8	4261.3(18)	8091.9(14)	6329.4(7)	13.0(3)
C9	3672.9(18)	6910.5(15)	5940.3(7)	16.7(3)
C10	4291.6(18)	5631.0(15)	6230.6(8)	16.5(3)

C11	6142.1(17)	5566.0(14)	6296.9(7)	13.1(3)
C12	6626.7(18)	4381.2(14)	6715.3(7)	14.7(3)
C13	8449.1(17)	4227.8(14)	6590.0(7)	14.2(3)
C14	9168.3(19)	2968.9(15)	6872.2(7)	19.4(3)
C15	9308.4(17)	5431.9(15)	6879.6(7)	15.6(3)
C16	8674.5(17)	6755.9(14)	6633.0(7)	14.3(3)
C17	6811.1(17)	6828.9(14)	6616.5(7)	11.9(3)
C18	7047.1(18)	5233.1(14)	5664.8(7)	15.3(3)
C19	8461.0(18)	4307.5(14)	5842.2(7)	14.9(3)
C20	9584(2)	2619.9(16)	5231.3(8)	21.4(3)
C21	10873(2)	3586.5(16)	5444.1(8)	19.7(3)

Supplementary Table 14. Anisotropic Displacement Parameters (Å^2×10^3) for S10. The Anisotropic displacement factor exponent takes the form: $-2\pi^2[h^2a^{*2}U_{11}+2hka^*b^*U_{12}+\ldots]$.

**Atom**	**U_{11}**	**U_{22}**	**U_{33}**	**U_{23}**	**U_{13}**	**U_{12}**
O1	27.8(6)	22.5(6)	34.0(7)	-5.4(5)	-0.2(5)	9.4(5)
O2	18.3(5)	15.0(5)	25.7(6)	-7.6(4)	0.7(4)	0.8(4)
O3	17.5(5)	16.5(5)	20.1(5)	-0.9(4)	5.5(4)	0.6(4)
C1	14.6(7)	21.5(7)	25.8(8)	4.6(6)	1.1(6)	4.0(6)
C2	14.1(7)	17.1(7)	15.6(6)	3.2(5)	1.3(5)	2.3(6)
C3	21.1(7)	14.6(7)	17.1(7)	4.0(6)	3.5(6)	2.6(6)
C4	20.8(7)	12.1(7)	23.1(7)	-2.3(6)	1.3(6)	-1.4(6)
C5	14.3(7)	14.0(6)	19.3(7)	-1.3(5)	-0.7(5)	-0.8(6)
C6	11.3(6)	12.5(6)	12.5(6)	0.3(5)	0.7(5)	-0.8(5)
C7	15.6(7)	18.4(7)	16.4(7)	3.6(5)	3.0(6)	2.0(6)
C8	11.6(6)	14.4(7)	12.9(6)	1.2(5)	0.5(5)	0.3(5)
C9	12.6(6)	17.6(7)	19.8(7)	-2.1(6)	-4.6(5)	0.2(6)
C10	11.5(6)	13.9(7)	24.2(7)	-0.7(6)	-2.5(6)	-2.7(6)
C11	11.0(6)	13.2(7)	15.1(6)	-0.5(5)	-0.9(5)	-0.8(5)
C12	13.1(7)	13.2(6)	18.0(7)	0.9(5)	0.6(5)	-1.1(5)
C13	12.3(6)	14.7(6)	15.8(7)	1.9(5)	-0.7(5)	-0.6(5)
C14	17.8(7)	18.2(7)	22.2(7)	4.4(6)	0.4(6)	2.0(6)
C15	13.0(6)	18.2(7)	15.6(6)	-0.4(5)	-3.2(5)	0.2(6)
C16	11.8(6)	14.4(6)	16.8(6)	-1.3(5)	-1.7(5)	-1.7(5)
C17	12.3(6)	12.4(6)	11.1(6)	0.0(5)	0.2(5)	-0.6(5)
C18	16.5(7)	15.8(6)	13.6(7)	-3.1(5)	-2.4(5)	1.3(6)

C19	14.2(7)	13.3(7)	17.3(7)	-3.0(5)	-0.3(5)	-0.5(5)
C20	23.2(8)	19.0(7)	22.1(7)	-4.0(6)	1.3(6)	7.2(6)
C21	17.8(7)	20.5(7)	20.7(7)	1.0(6)	3.1(6)	5.3(6)

Supplementary Table 15. Bond Lengths for **S10**.

Atom	Atom	Length/Å	Atom	Atom	Length/Å
O1	C3	1.215(2)	C8	C9	1.5294(19)
O2	C19	1.4441(17)	C9	C10	1.524(2)
O2	C20	1.4288(19)	C10	C11	1.5329(19)
O3	C19	1.4147(18)	C11	C12	1.5411(19)
O3	C21	1.4302(18)	C11	C17	1.5494(18)
C1	C2	1.532(2)	C11	C18	1.5516(19)
C2	C3	1.521(2)	C12	C13	1.5326(19)
C2	C8	1.5504(19)	C13	C14	1.5296(19)
C3	C4	1.516(2)	C13	C15	1.5388(19)
C4	C5	1.533(2)	C13	C19	1.5610(19)
C5	C6	1.5404(19)	C15	C16	1.534(2)
C6	C7	1.5415(19)	C16	C17	1.5380(18)
C6	C8	1.5587(19)	C18	C19	1.544(2)
C6	C17	1.5660(19)	C20	C21	1.515(2)

Supplementary Table 16. Bond Angles for **S10**.

Atom	Atom	Atom	Angle/°	Atom	Atom	Atom	Angle/°
C20	O2	C19	108.39(11)	C12	C11	C17	108.28(11)
C19	O3	C21	106.27(11)	C12	C11	C18	100.67(11)
C1	C2	C8	113.16(12)	C17	C11	C18	112.05(11)
C3	C2	C1	110.31(13)	C13	C12	C11	103.73(12)
C3	C2	C8	111.70(12)	C12	C13	C15	107.64(11)
O1	C3	C2	122.32(14)	C12	C13	C19	99.85(11)
O1	C3	C4	120.17(14)	C14	C13	C12	113.55(12)
C4	C3	C2	117.44(13)	C14	C13	C15	109.76(12)
C3	C4	C5	114.98(13)	C14	C13	C19	115.16(12)
C4	C5	C6	113.74(12)	C15	C13	C19	110.33(12)
C5	C6	C7	108.83(12)	C16	C15	C13	114.30(11)

C5	C6	C8	107.31(11)	C15	C16	C17	112.95(12)
C5	C6	C17	107.97(11)	C11	C17	C6	116.22(11)
C7	C6	C8	110.73(12)	C16	C17	C6	113.42(11)
C7	C6	C17	113.33(11)	C16	C17	C11	108.96(11)
C8	C6	C17	108.46(11)	C19	C18	C11	107.03(11)
C2	C8	C6	112.79(12)	O2	C19	C13	110.39(12)
C9	C8	C2	112.48(11)	O2	C19	C18	108.99(11)
C9	C8	C6	111.49(12)	O3	C19	O2	105.83(11)
C10	C9	C8	110.81(12)	O3	C19	C13	114.73(12)
C9	C10	C11	113.93(12)	O3	C19	C18	111.42(12)
C10	C11	C12	110.04(12)	C18	C19	C13	105.44(11)
C10	C11	C17	110.92(12)	O2	C20	C21	104.12(12)
C10	C11	C18	114.28(12)	O3	C21	C20	102.28(12)

Supplementary Table 17. Torsion Angles for **S10**.

A	B	C	D	Angle/°	A	B	C	D	Angle/°
O1	C3	C4	C5	145.69(15)	C11	C12	C13	C19	-47.65(13)
O2	C20	C21	O3	-29.58(15)	C11	C18	C19	O2	113.19(13)
C1	C2	C3	O1	-16.0(2)	C11	C18	C19	O3	-130.39(12)
C1	C2	C3	C4	167.10(13)	C11	C18	C19	C13	-5.32(15)
C1	C2	C8	C6	-177.88(12)	C12	C11	C17	C6	-167.64(12)
C1	C2	C8	C9	54.98(16)	C12	C11	C17	C16	62.74(14)
C2	C3	C4	C5	-37.34(19)	C12	C11	C18	C19	-23.28(14)
C2	C8	C9	C10	-172.17(12)	C12	C13	C15	C16	-55.58(15)
C3	C2	C8	C6	-52.67(16)	C12	C13	C19	O2	-85.73(13)
C3	C2	C8	C9	-179.82(12)	C12	C13	C19	O3	154.82(11)
C3	C4	C5	C6	45.78(18)	C12	C13	C19	C18	31.83(14)
C4	C5	C6	C7	63.78(16)	C13	C15	C16	C17	45.29(17)
C4	C5	C6	C8	-56.09(16)	C14	C13	C15	C16	-179.62(12)
C4	C5	C6	C17	-172.82(12)	C14	C13	C19	O2	36.25(17)
C5	C6	C8	C2	60.26(14)	C14	C13	C19	O3	-83.20(15)
C5	C6	C8	C9	-172.07(11)	C14	C13	C19	C18	153.81(12)
C5	C6	C17	C11	166.21(12)	C15	C13	C19	O2	161.16(11)
C5	C6	C17	C16	-66.35(14)	C15	C13	C19	O3	41.71(16)
C6	C8	C9	C10	60.00(15)	C15	C13	C19	C18	-81.27(14)
C7	C6	C8	C2	-58.39(15)	C15	C16	C17	C6	-178.45(11)

C7	C6	C8	C9	69.28(15)	C15	C16	C17	C11	-47.31(15)
C7	C6	C17	C11	-73.17(15)	C17	C6	C8	C2	176.67(11)
C7	C6	C17	C16	54.28(15)	C17	C6	C8	C9	-55.66(14)
C8	C2	C3	O1	-142.78(14)	C17	C11	C12	C13	-73.27(13)
C8	C2	C3	C4	40.32(17)	C17	C11	C18	C19	91.60(13)
C8	C6	C17	C11	50.22(14)	C18	C11	C12	C13	44.40(13)
C8	C6	C17	C16	177.67(11)	C18	C11	C17	C6	82.23(14)
C8	C9	C10	C11	-56.35(16)	C18	C11	C17	C16	-47.38(15)
C9	C10	C11	C12	168.45(12)	C19	O2	C20	C21	12.17(16)
C9	C10	C11	C17	48.65(16)	C19	O3	C21	C20	36.73(14)
C9	C10	C11	C18	-79.18(16)	C19	C13	C15	C16	52.43(16)
C10	C11	C12	C13	165.34(12)	C20	O2	C19	O3	10.15(15)
C10	C11	C17	C6	-46.80(15)	C20	O2	C19	C13	-114.55(13)
C10	C11	C17	C16	-176.41(12)	C20	O2	C19	C18	130.09(13)
C10	C11	C18	C19	-141.15(12)	C21	O3	C19	O2	-29.95(14)
C11	C12	C13	C14	-170.77(12)	C21	O3	C19	C13	92.01(14)
C11	C12	C13	C15	67.52(14)	C21	O3	C19	C18	-148.28(12)

Supplementary Table 18. Hydrogen Atom Coordinates (Å×10^4) and Isotropic Displacement Parameters (Å^2×10^3) for **S10**.

**Atom**	***x***	***y***	***z***	**U(eq)**
H1A	1233.55	9046.89	6530.42	31
H1B	1234.82	8750.27	5776.53	31
H1C	1184.53	10235.07	6028.08	31
H2	3817.43	9547.84	5653.42	19
H4A	6011.33	11181.4	7041.57	22
H4B	6465.17	10982.69	6303.15	22
H5A	6263.85	8988.71	7261.04	19
H5B	7809.9	9344.69	6831.86	19
H7A	8023.32	8485.72	5695.7	25
H7B	6480.95	9401.34	5563.21	25
H7C	6437.65	7883.18	5364.47	25
H8	3895.34	7943.01	6780.95	16
H9A	2472.04	6901.51	5932.12	20
H9B	4064.08	6988.3	5492.93	20
H10A	3925.81	4890.33	5958.91	20

H10B	3798.64	5517.18	6660.1	20
H12A	6411.96	4558.03	7174.52	18
H12B	6027.34	3581.76	6584.96	18
H14A	8847.53	2889.83	7323.25	29
H14B	10354.13	3001.69	6842.08	29
H14C	8763.87	2208.73	6632.14	29
H15A	9181.69	5406.14	7351.65	19
H15B	10482.25	5370.7	6783.48	19
H16A	9099.88	6907.83	6195.16	17
H16B	9092.55	7465.69	6912.52	17
H17	6452.14	6789.95	7074.36	14
H18A	6303.28	4795.6	5359.17	18
H18B	7467.32	6044.81	5462.8	18
H20A	9417.12	2664.78	4761.51	26
H20B	9887.2	1710.62	5349.67	26
H21A	11440.49	3274.59	5834.01	24
H21B	11679.36	3743.04	5100.41	24

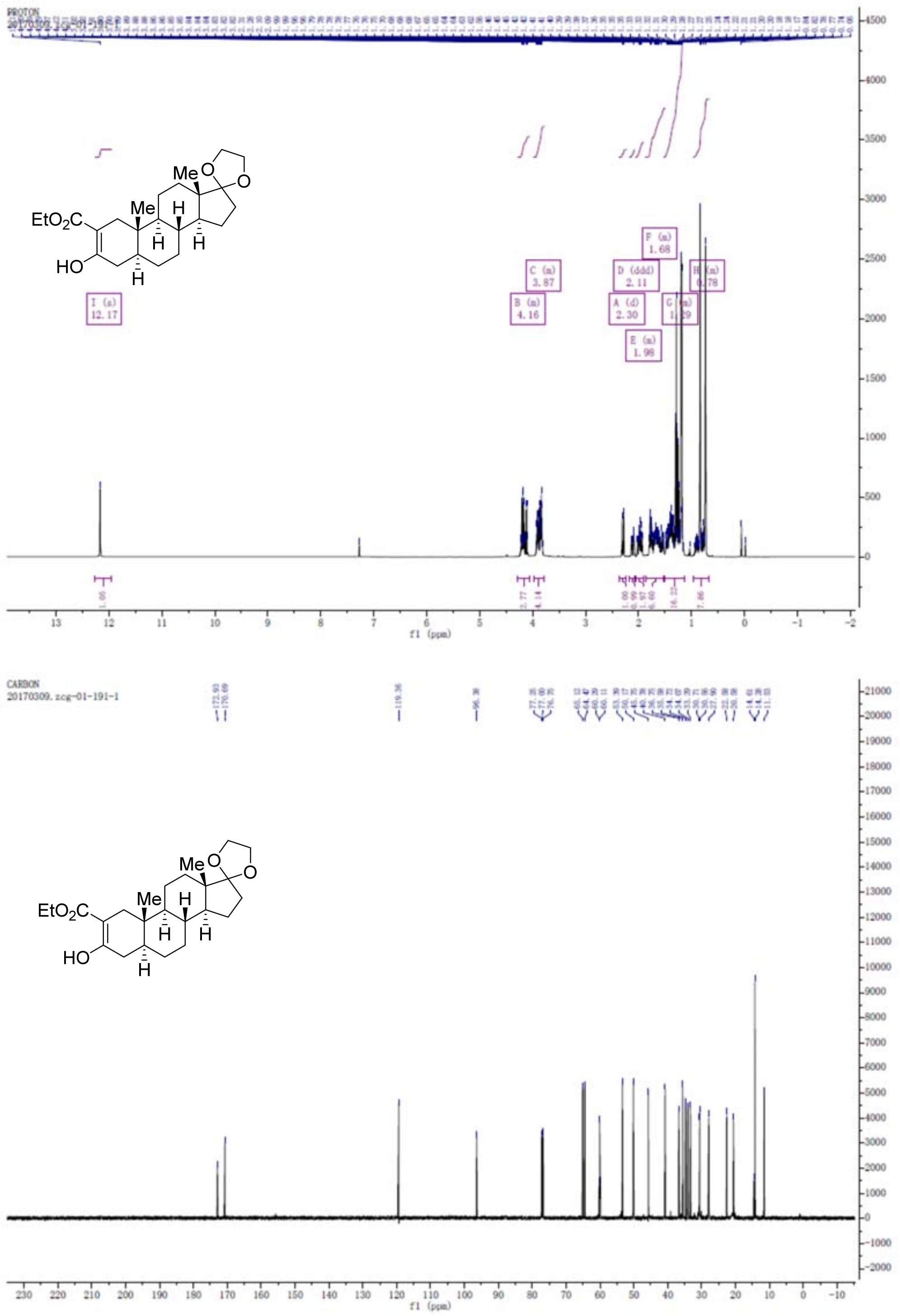

Supplementary Figure 26. ^{1}H and ^{13}C spectra of **2a**.

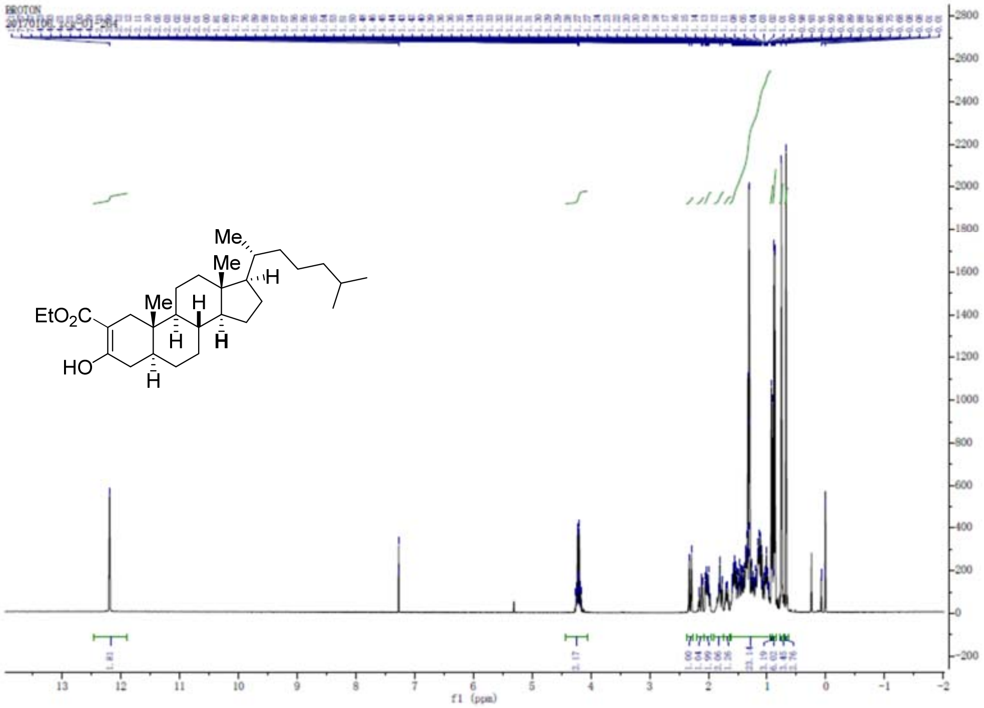

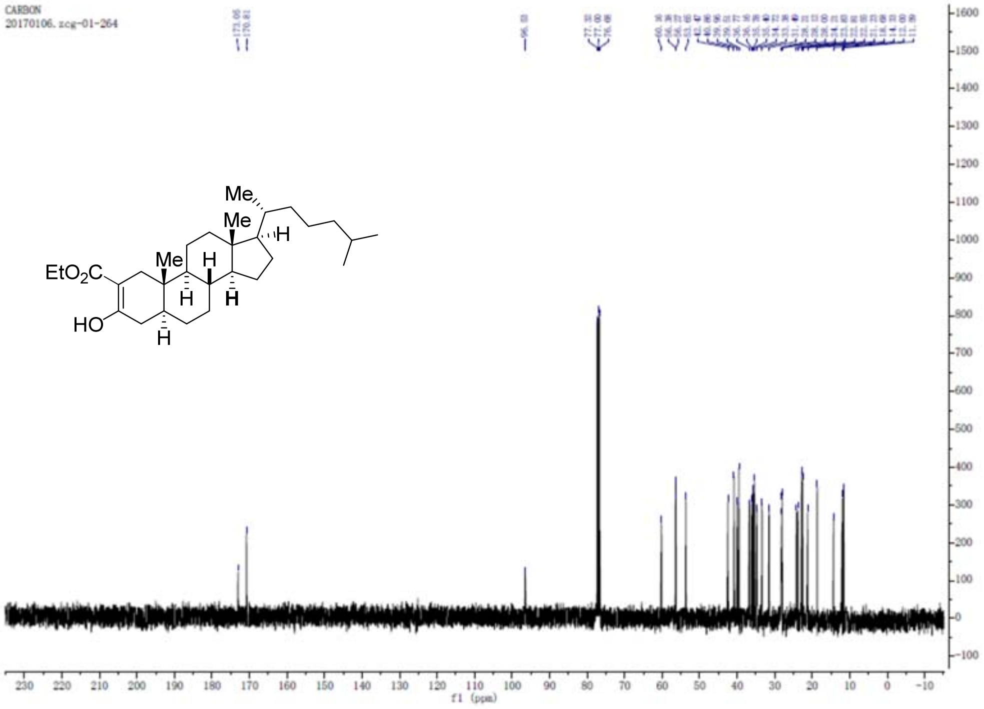

Supplementary Figure 27. ^{1}H and ^{13}C spectra of **2b**.

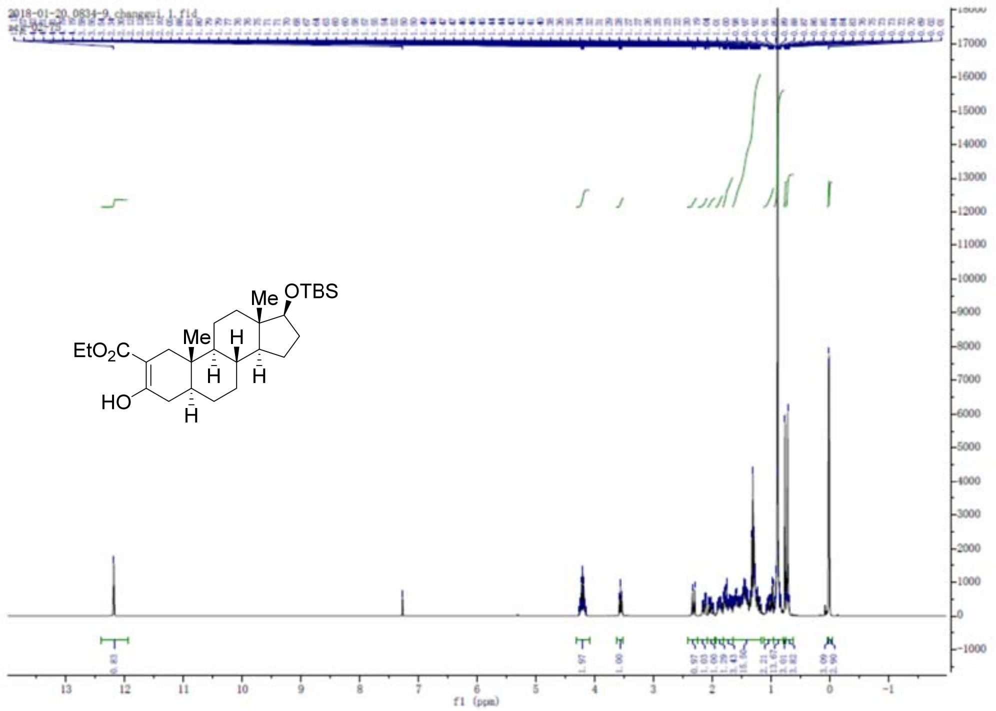

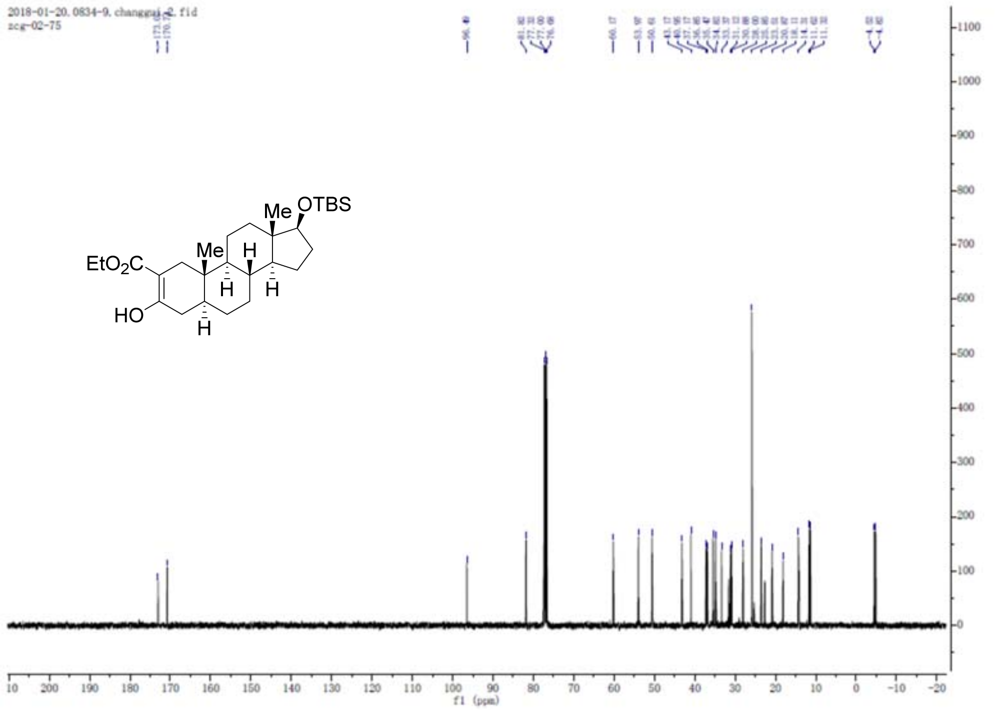

Supplementary Figure 28. ^{1}H and ^{13}C spectra of **2c**.

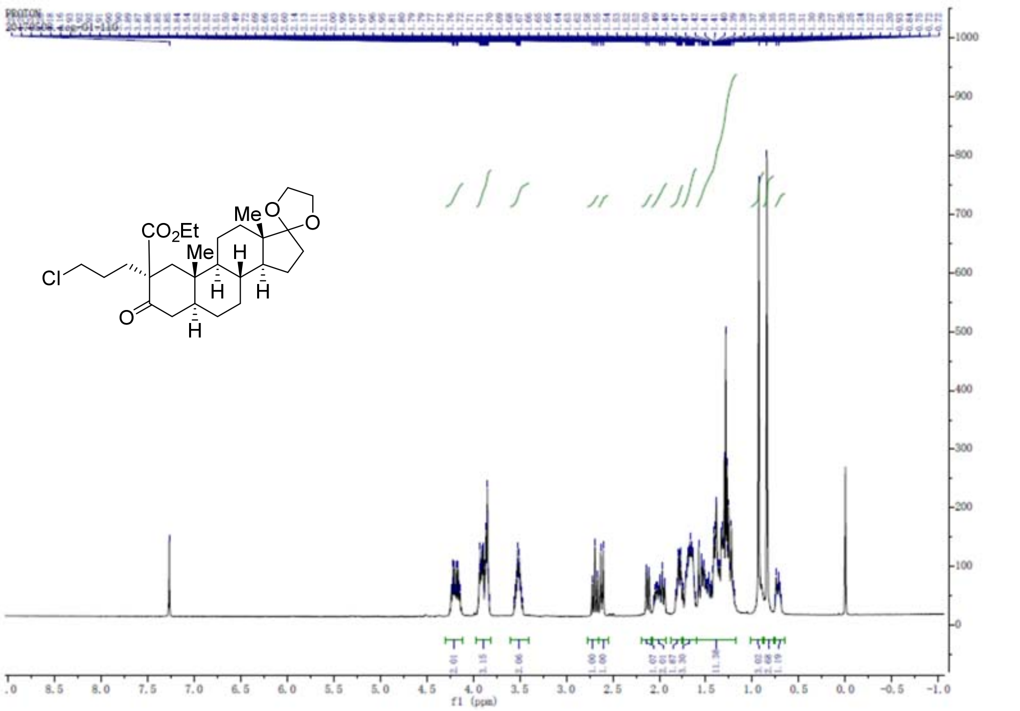

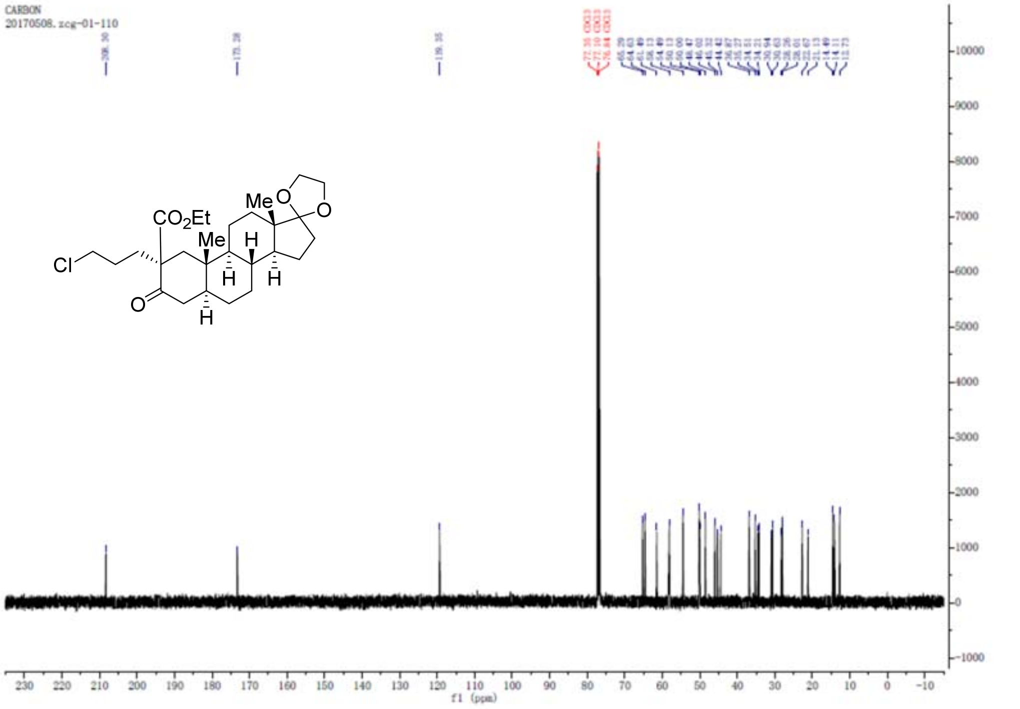

Supplementary Figure 29. ^{1}H and ^{13} C spectra of **S2a**.

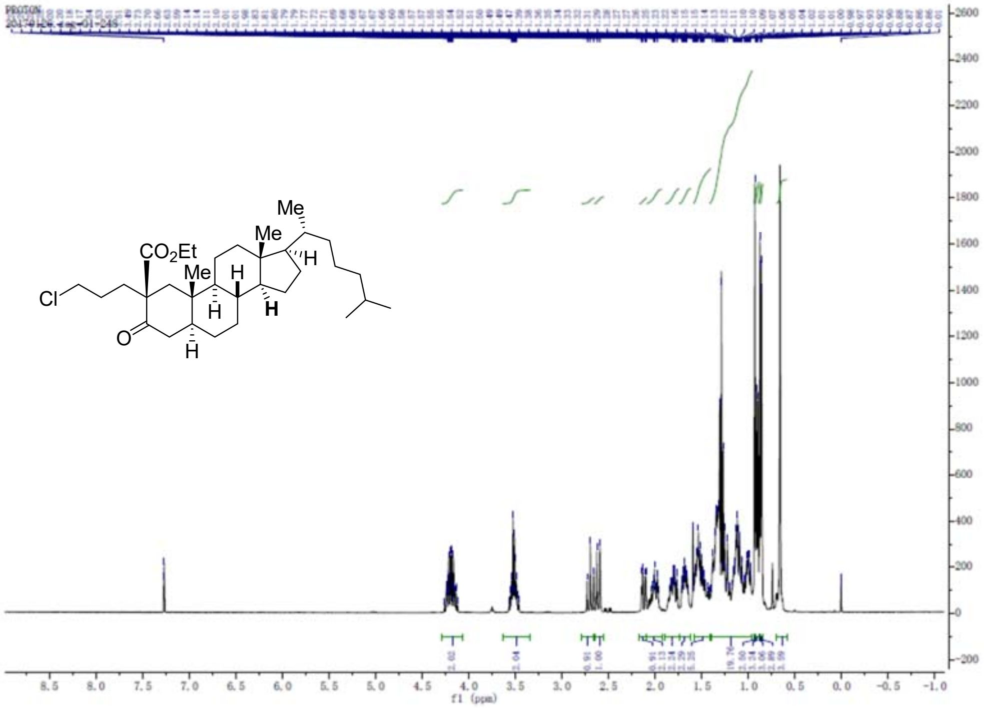

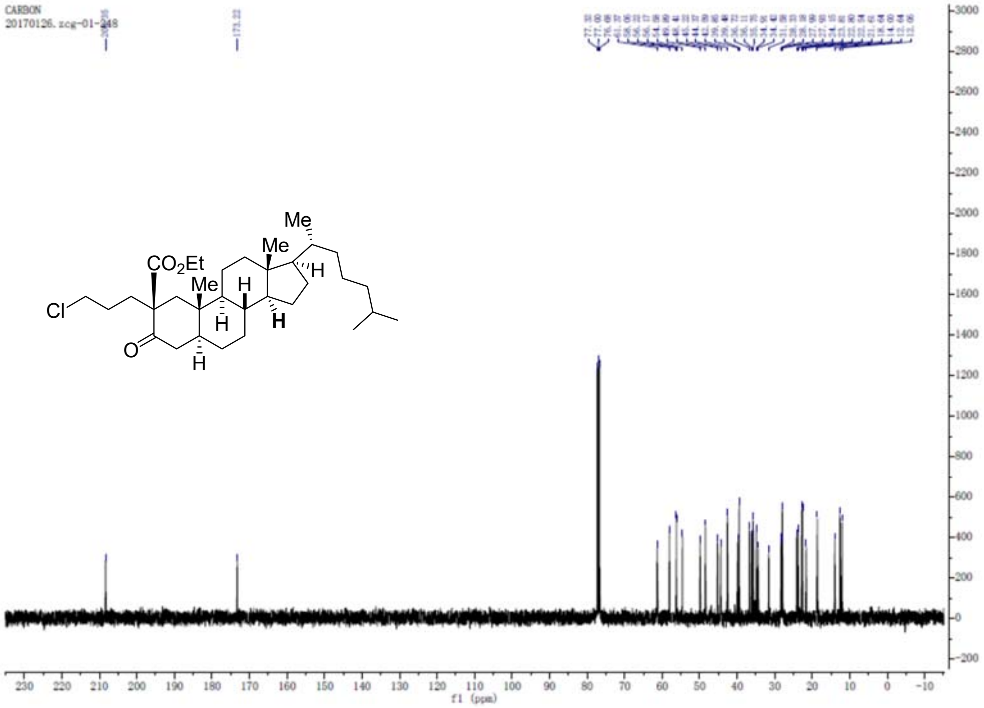

Supplementary Figure 30. ^{1}H and ^{13}C spectra of **S2b**.

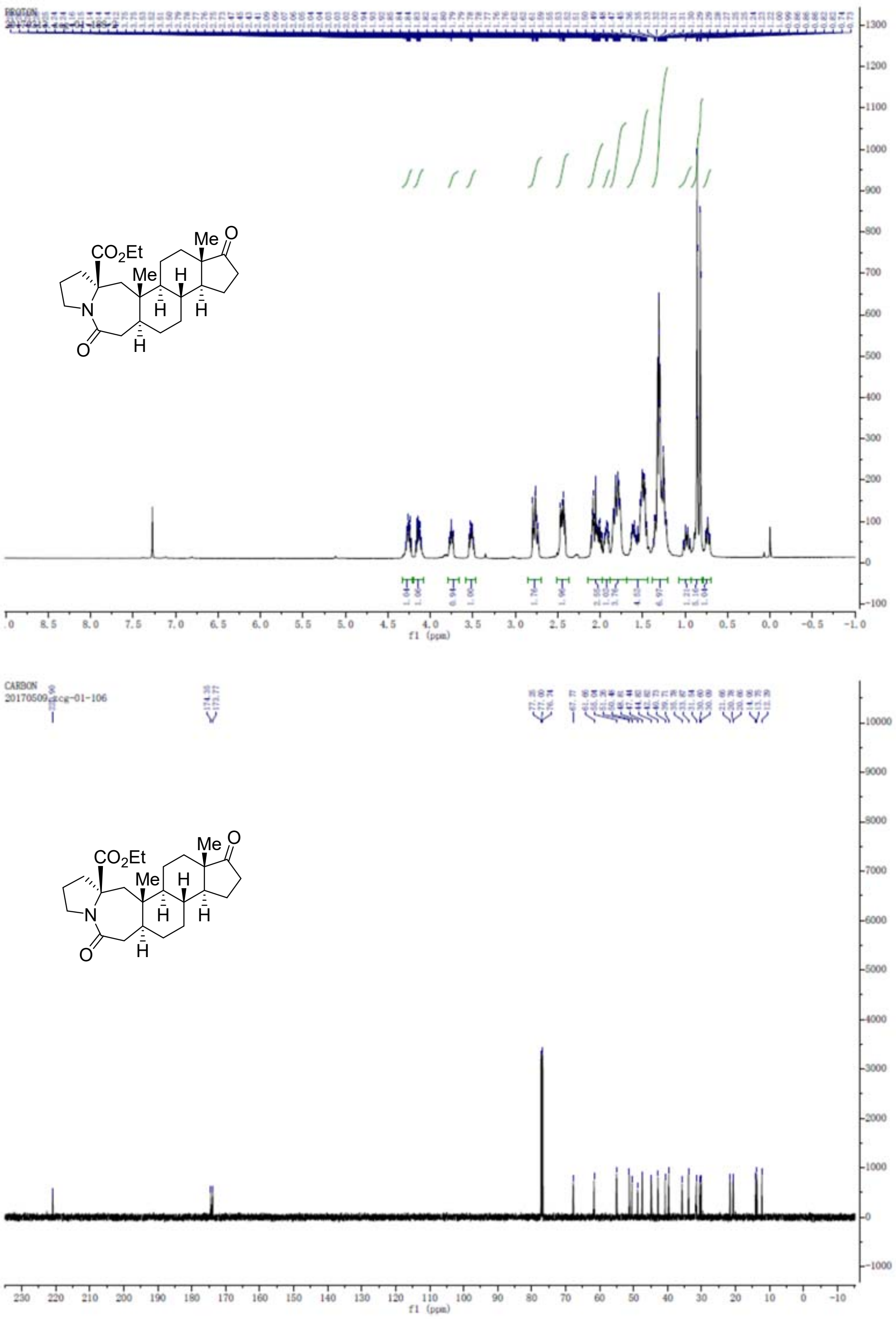

Supplementary Figure 31. ^{1}H and ^{13}C spectra of **3a**.

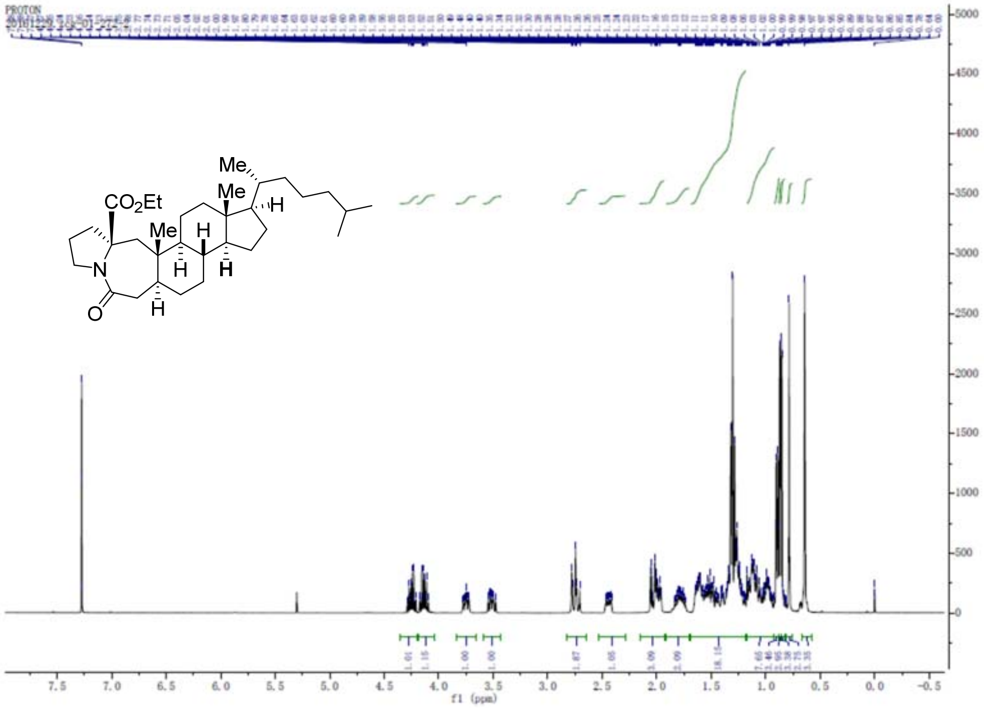

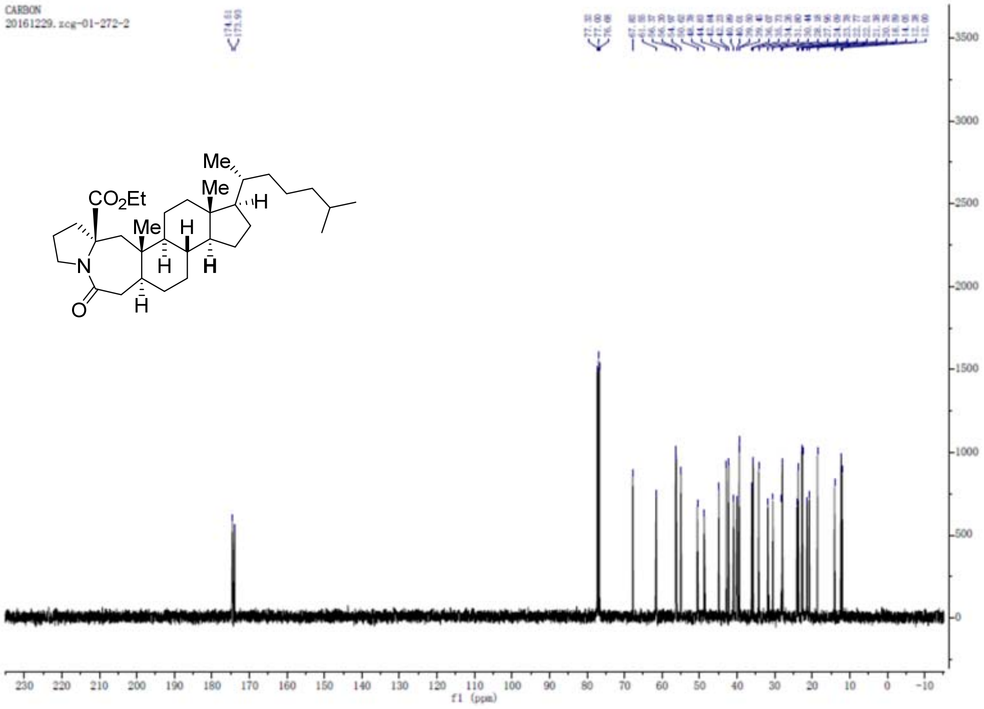

Supplementary Figure 32. ^{1}H and ^{13}C spectra of **3b**.

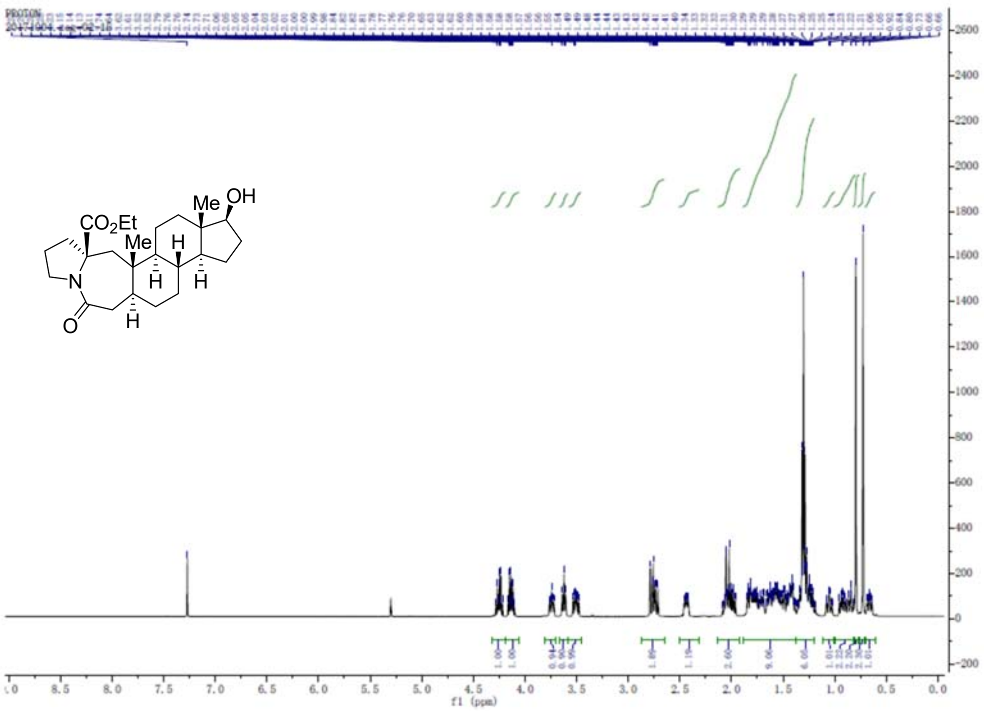

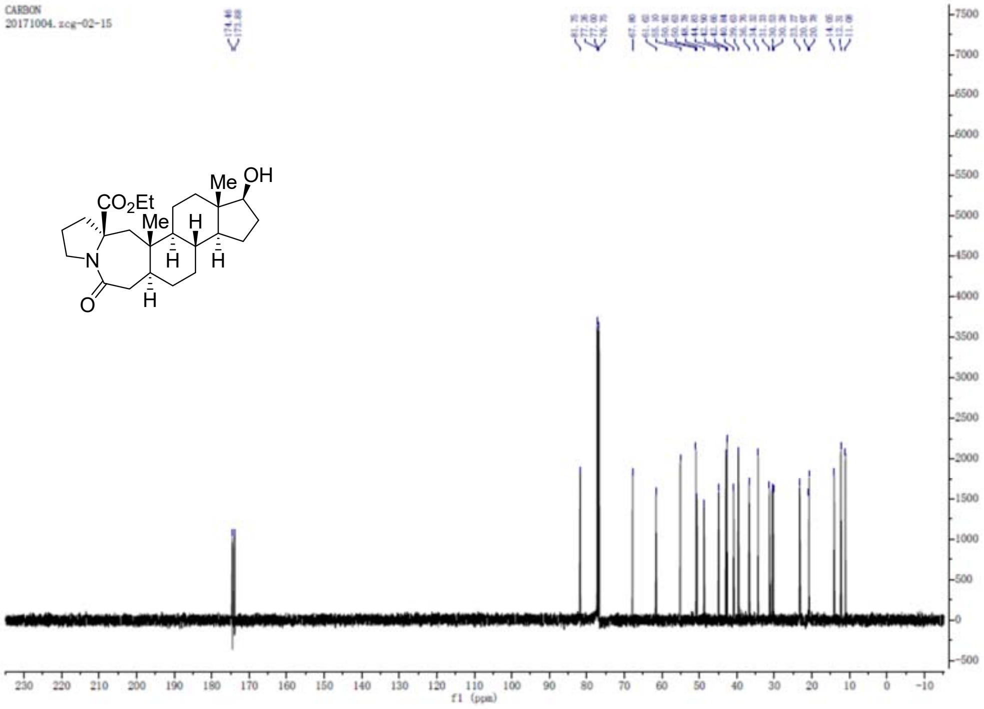

Supplementary Figure 33. ^{1}H and ^{13}C spectra of **4a**.

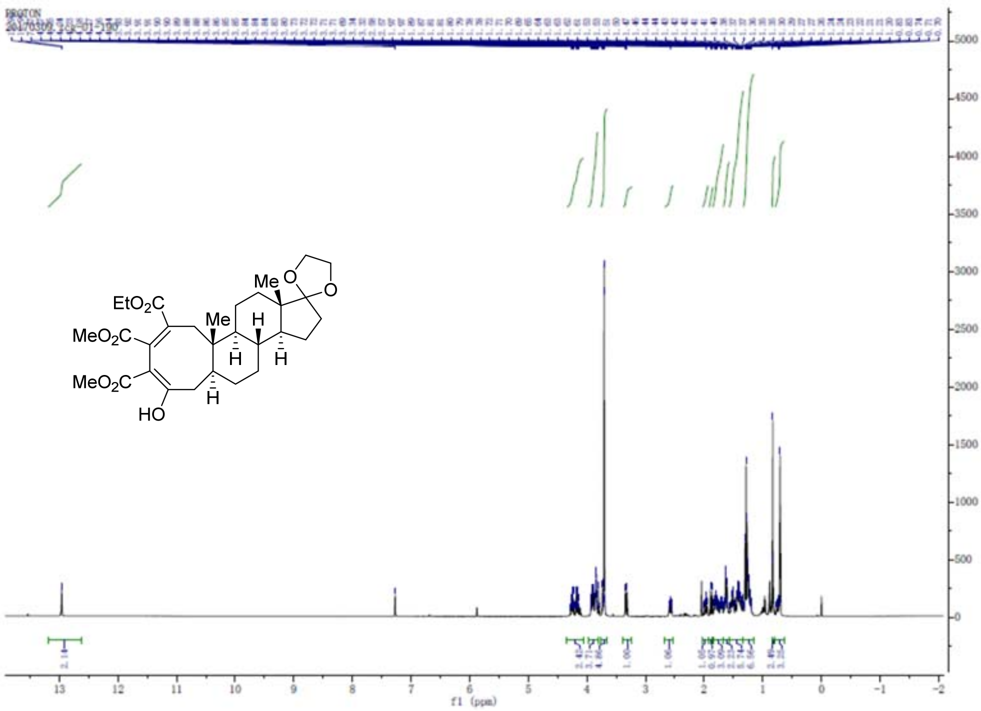

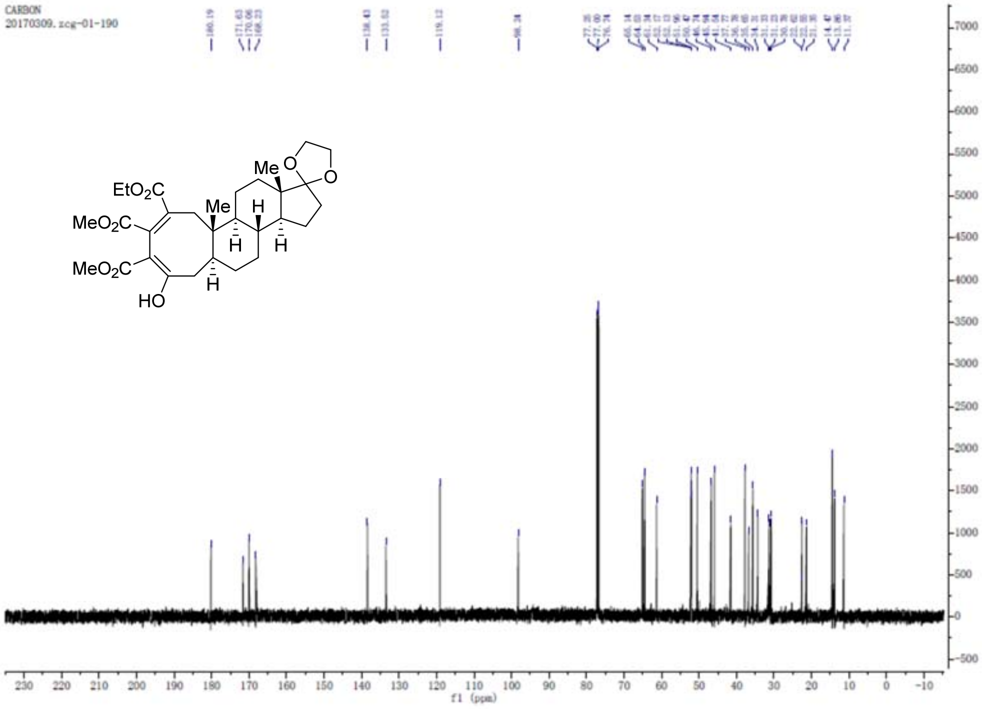

Supplementary Figure 34. ^{1}H and ^{13} C spectra of **5a**.

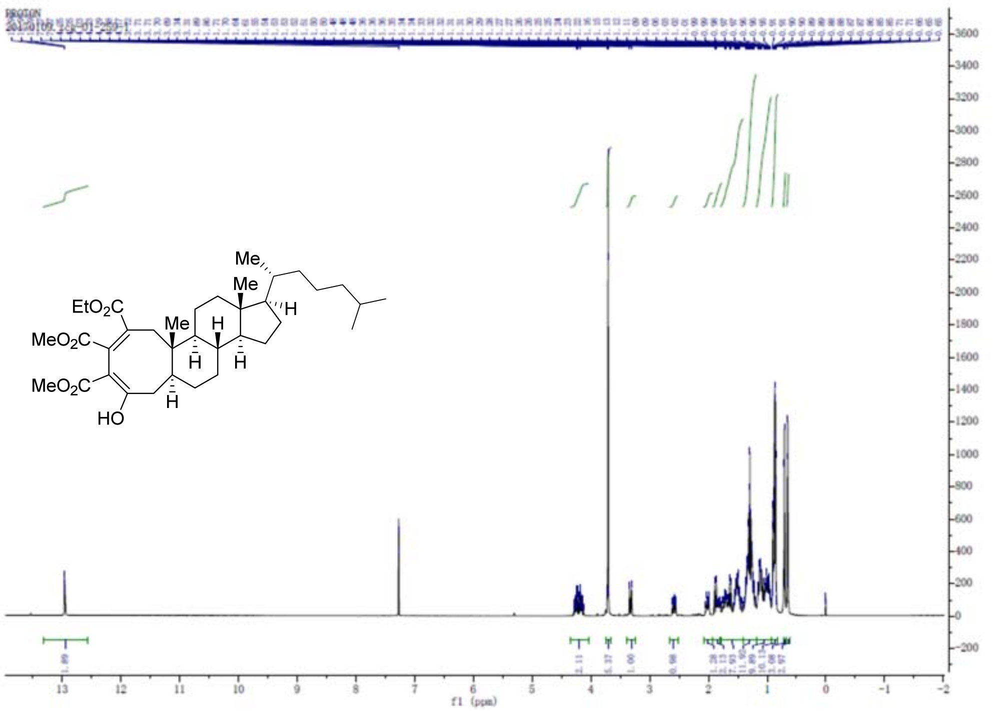

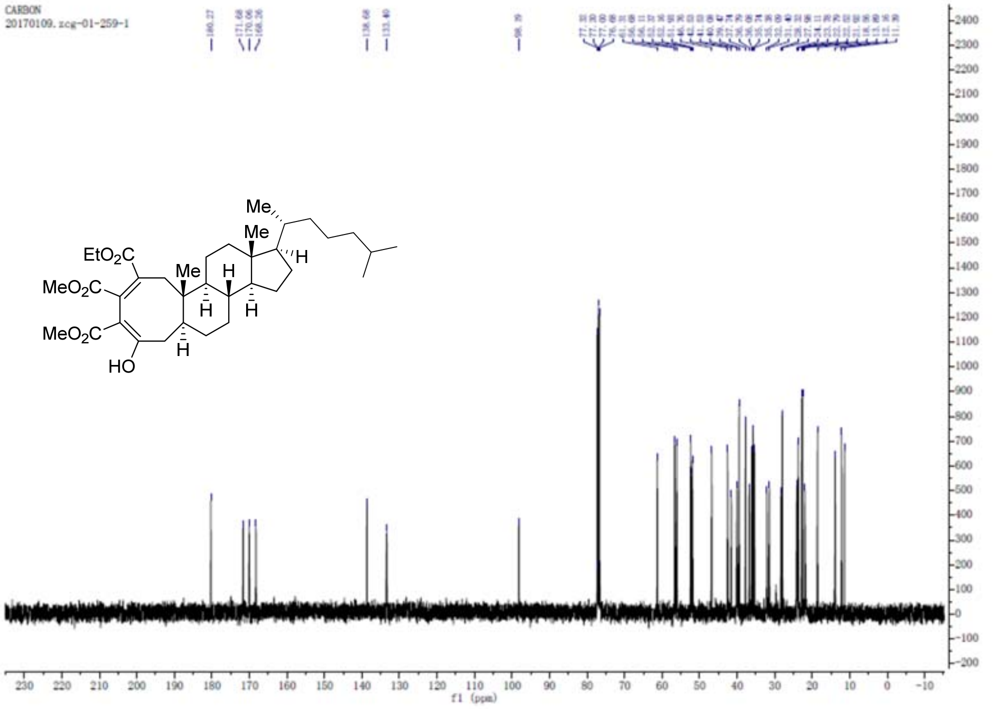

Supplementary Figure 35. ^{1}H and ^{13} C spectra of **5b**.

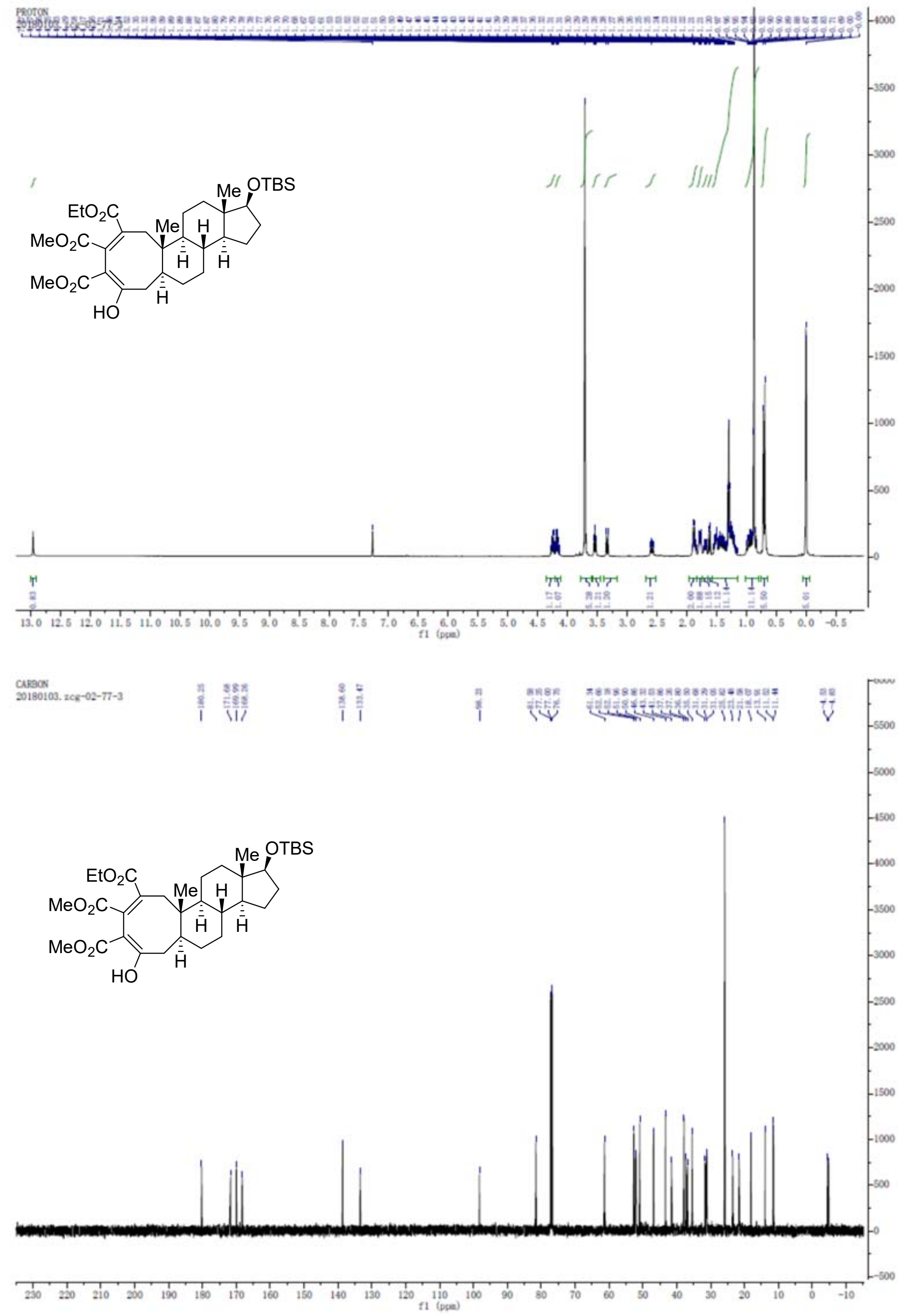

Supplementary Figure 36. ^{1}H and ^{13}C spectra of **5c**.

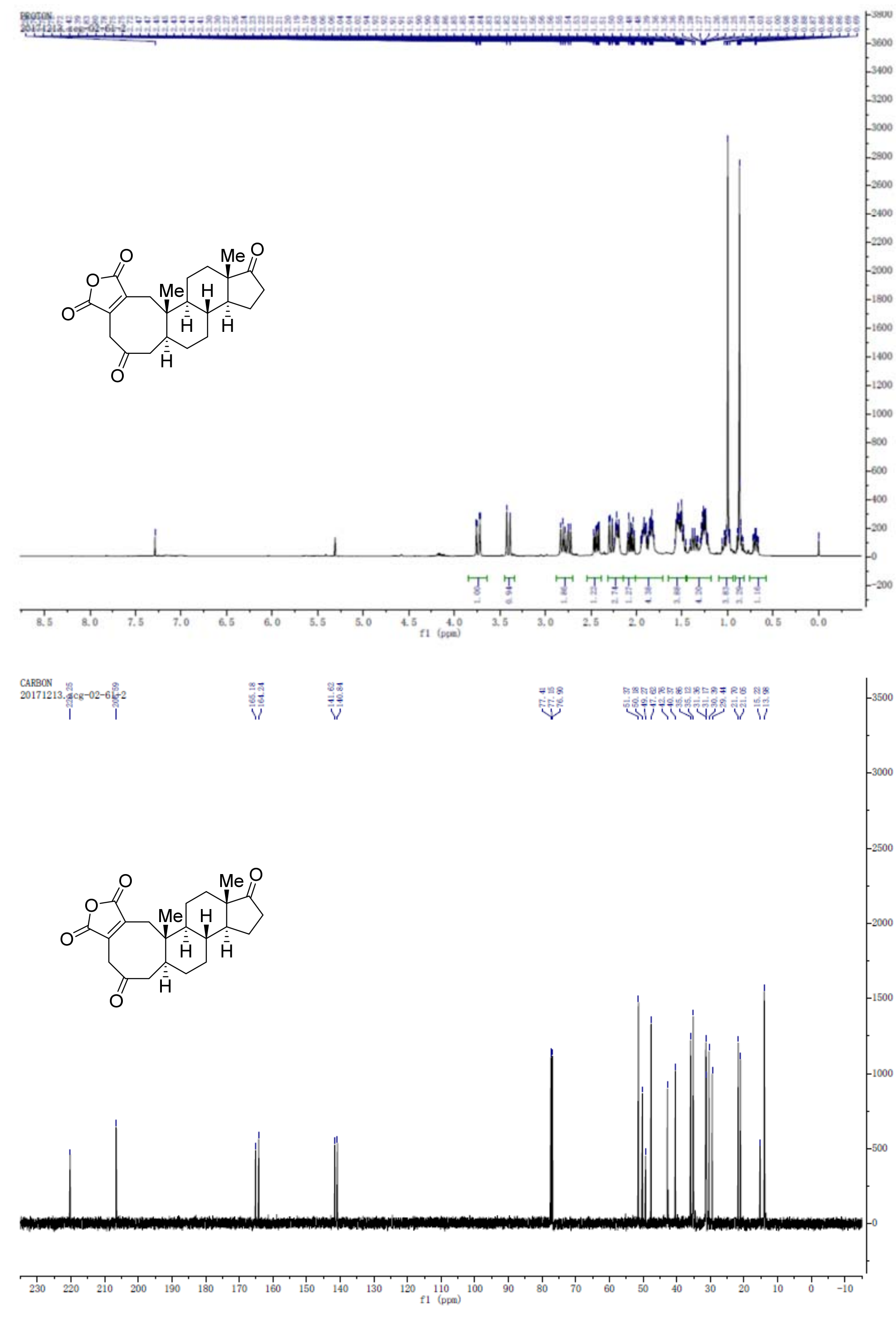

Supplementary Figure 37. ^{1}H and ^{13} C spectra of **6a**.

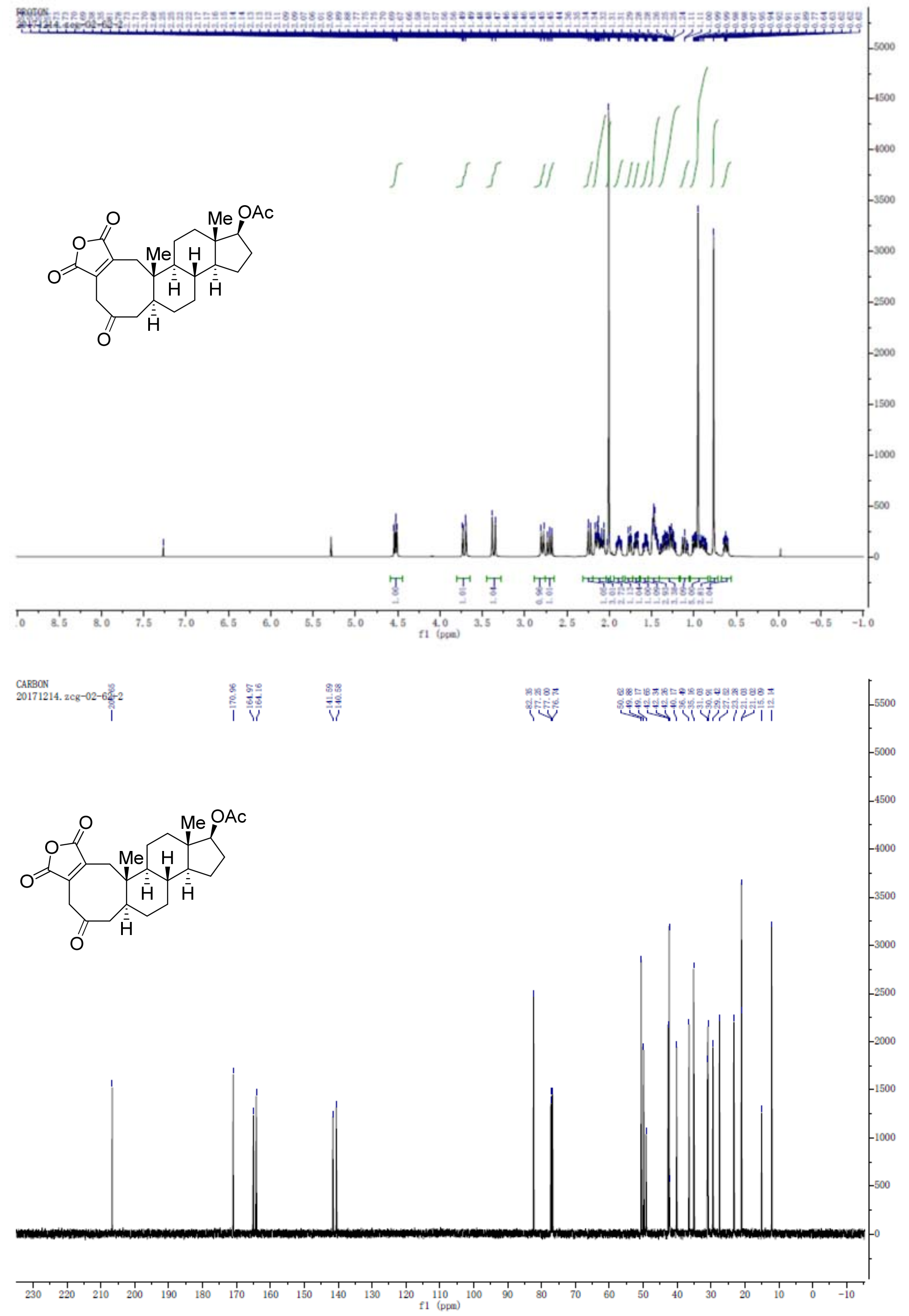

Supplementary Figure 38. ^{1}H and ^{13}C spectra of **6c**.

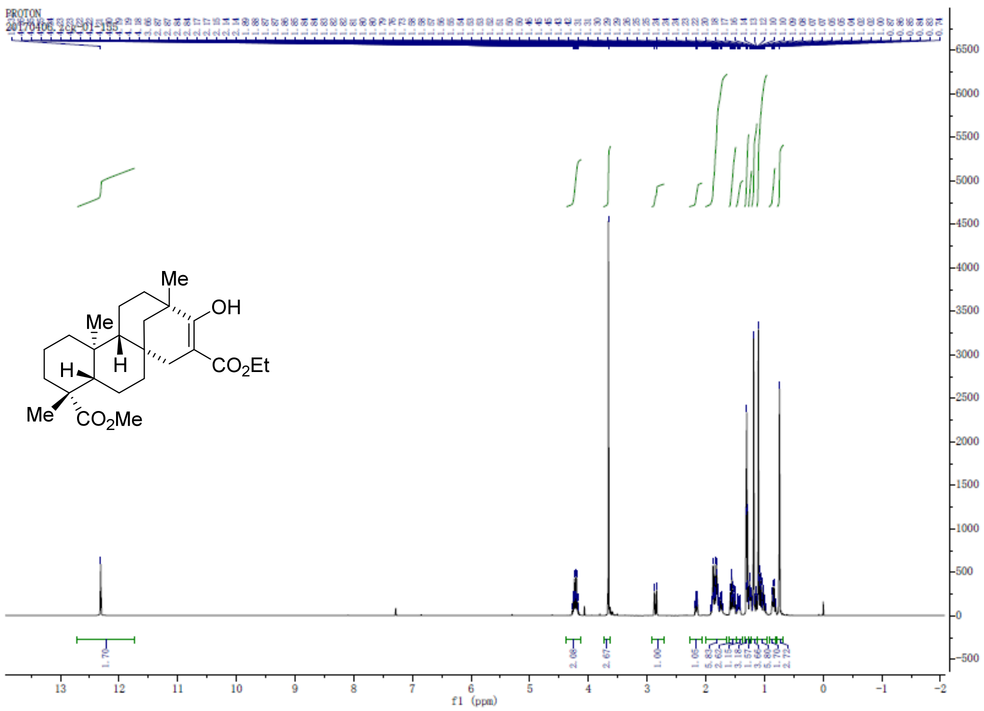

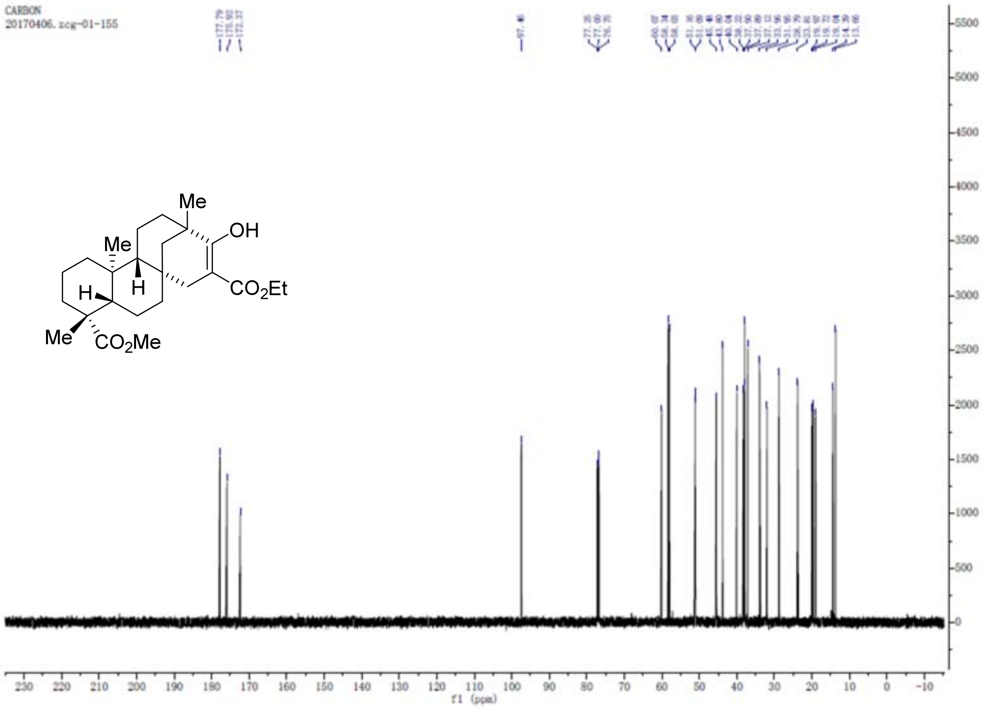

Supplementary Figure 39. ^{1}H and ^{13}C spectra of **7**.

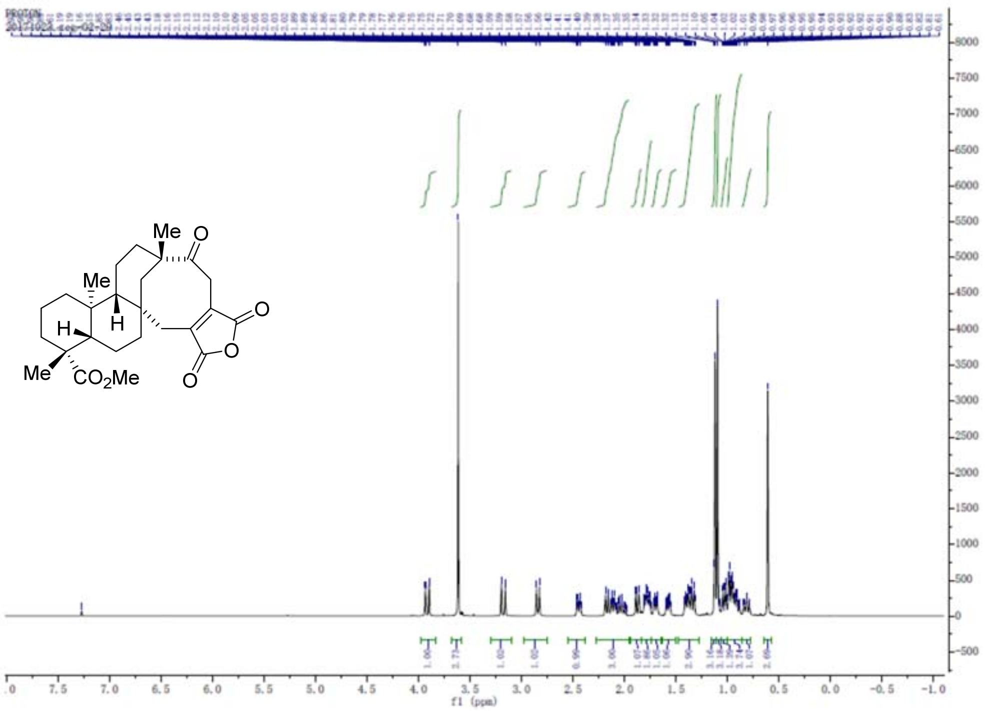

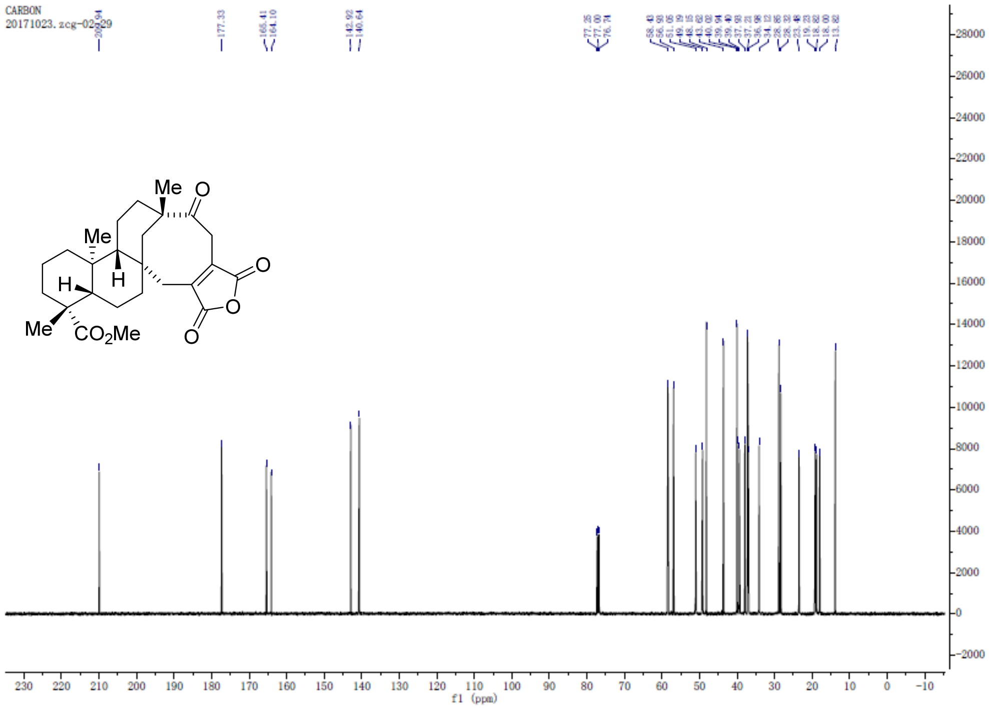

Supplementary Figure 40. ^{1}H and ^{13}C spectra of **8**.

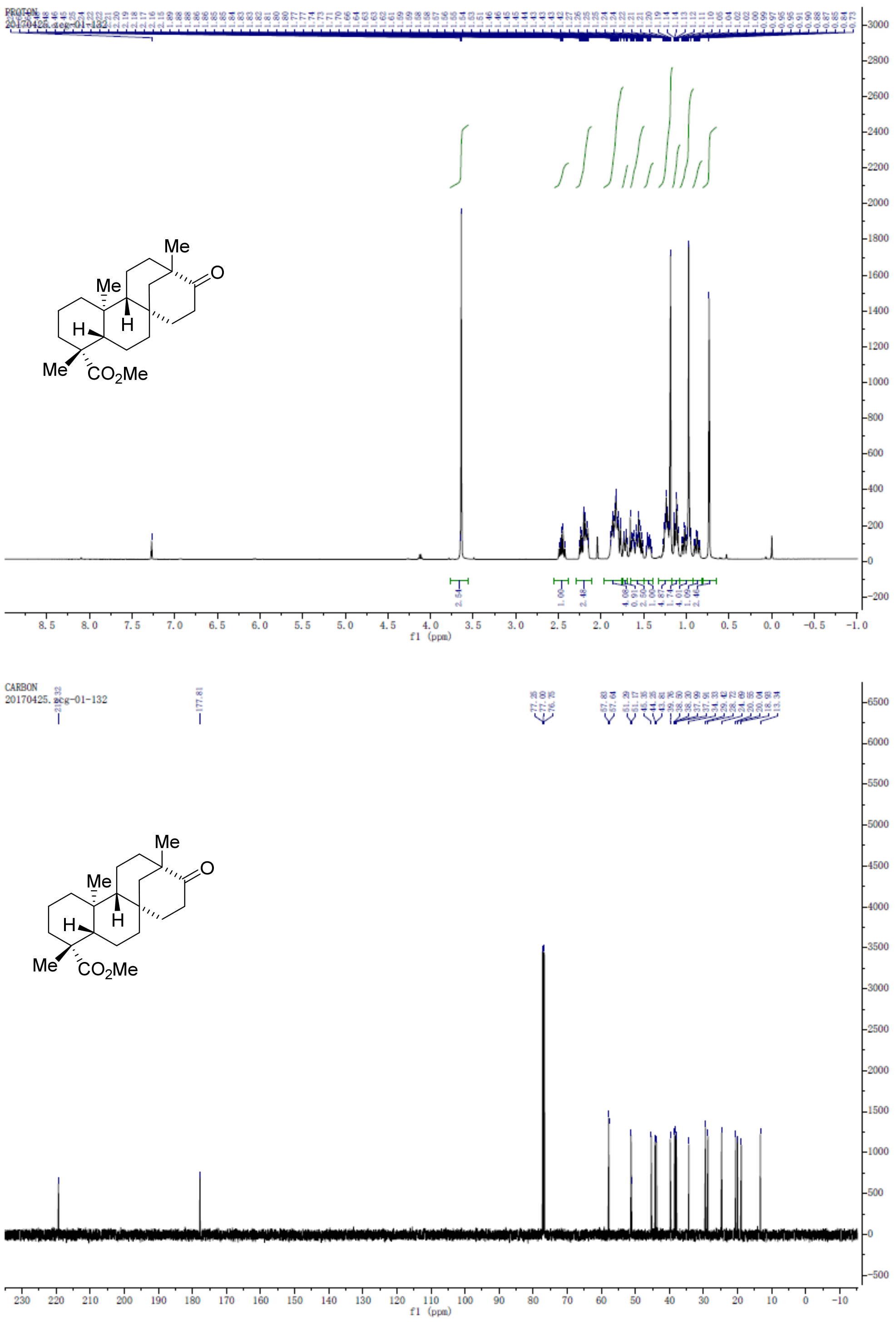

Supplementary Figure 41. ^{1}H and ^{13}C spectra of **S4**.

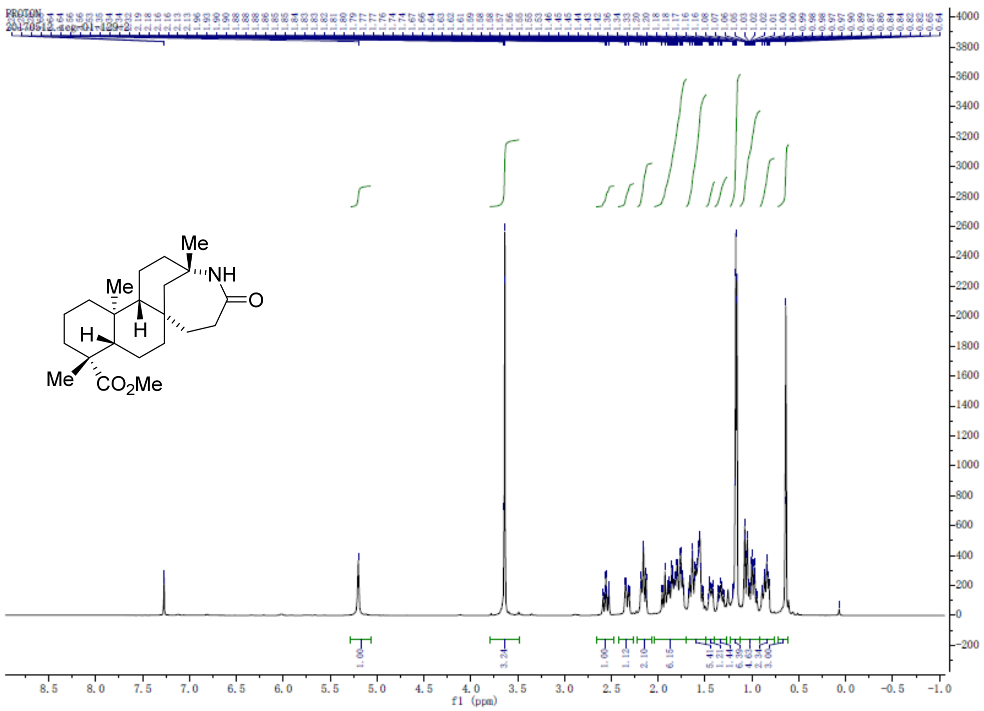

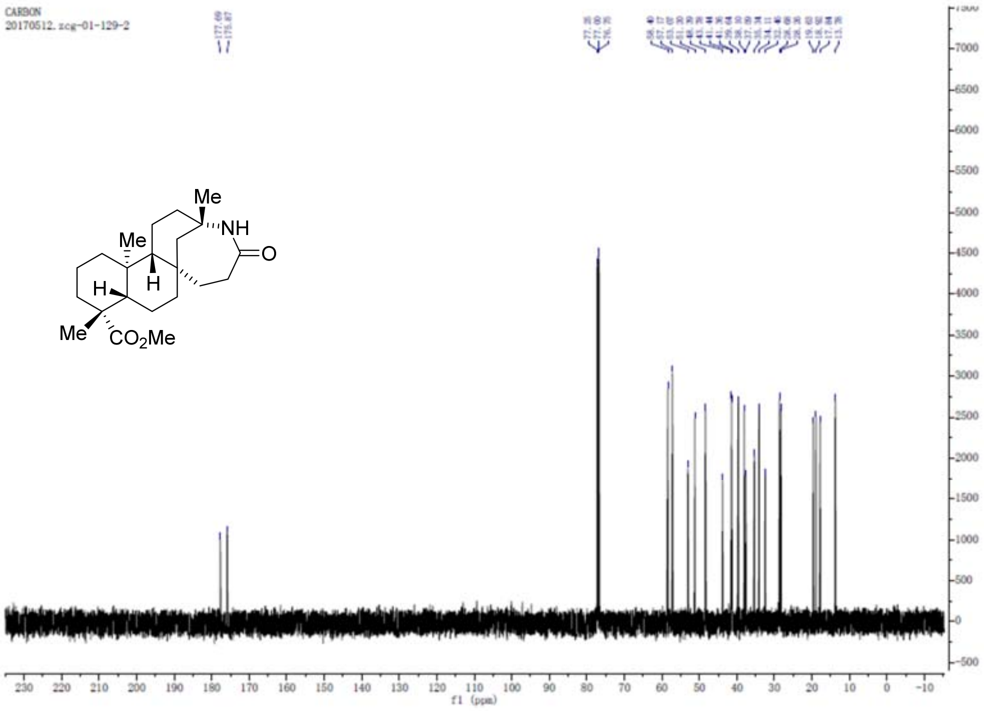

Supplementary Figure 42. ^{1}H and ^{13}C spectra of **9**.

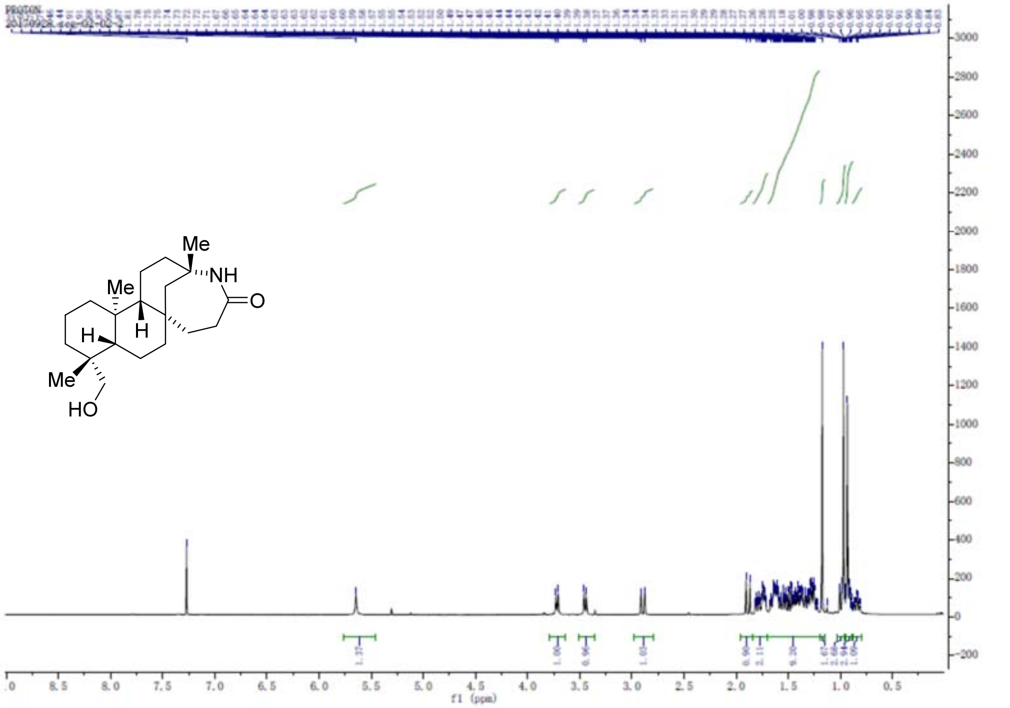

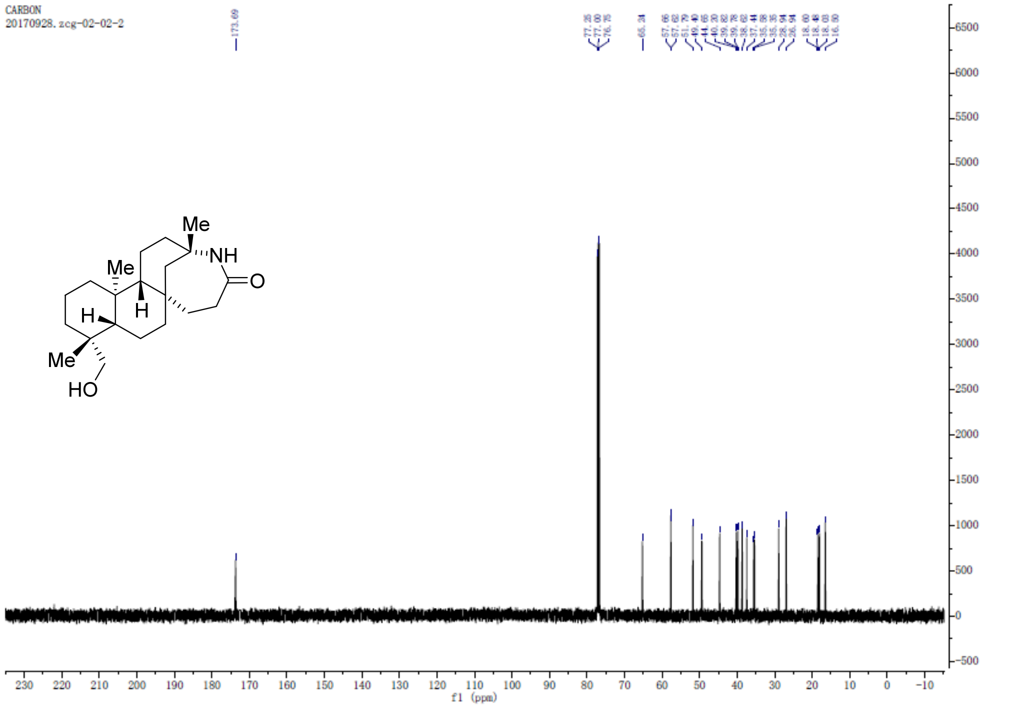

Supplementary Figure 43. ^{1}H and ^{13}C spectra of **10**.

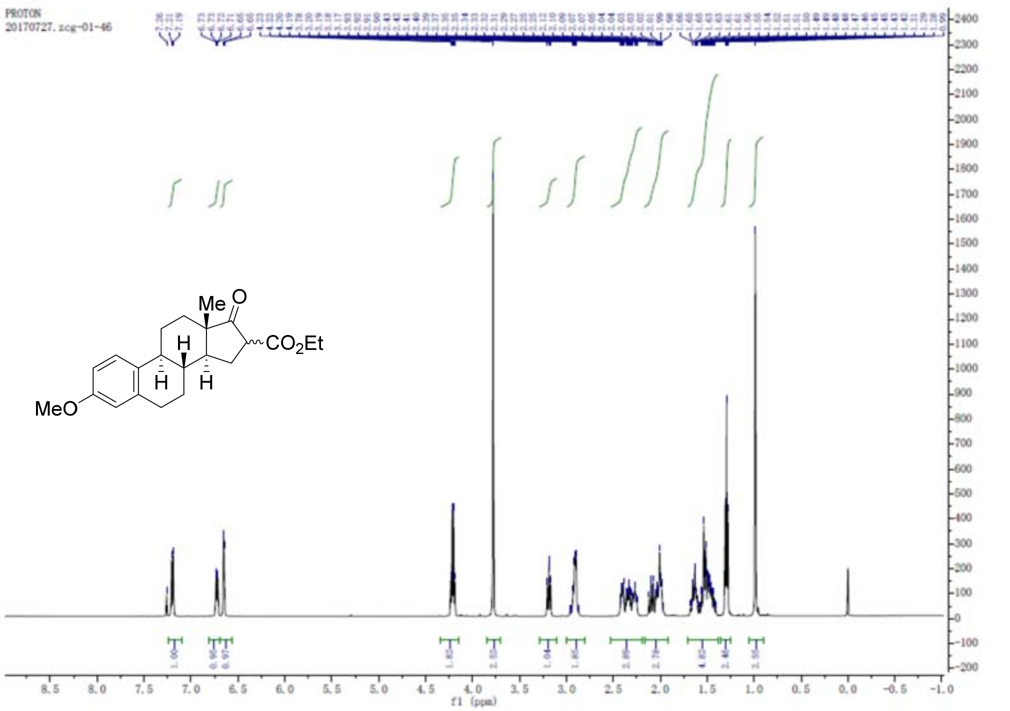

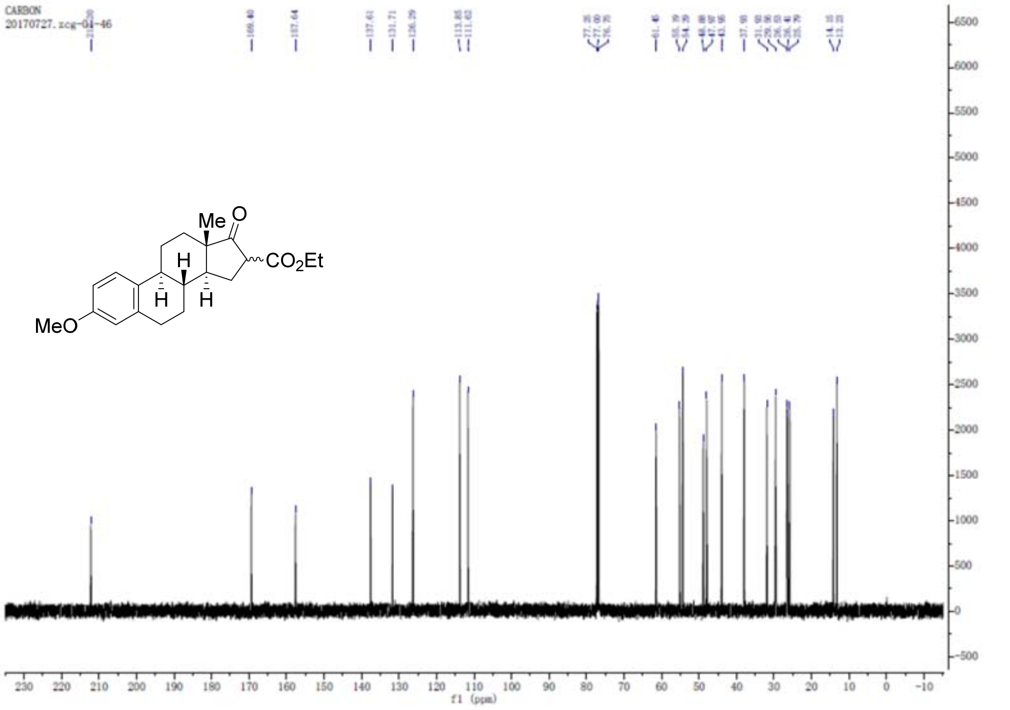

Supplementary Figure 44. ^{1}H and ^{13}C spectra of **11**.

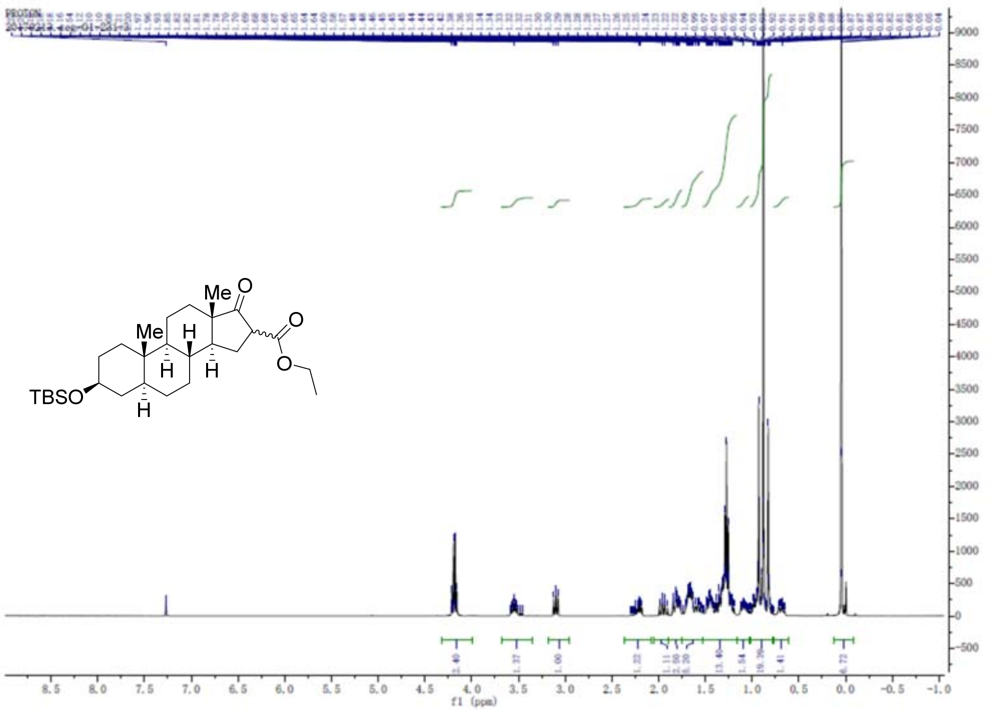

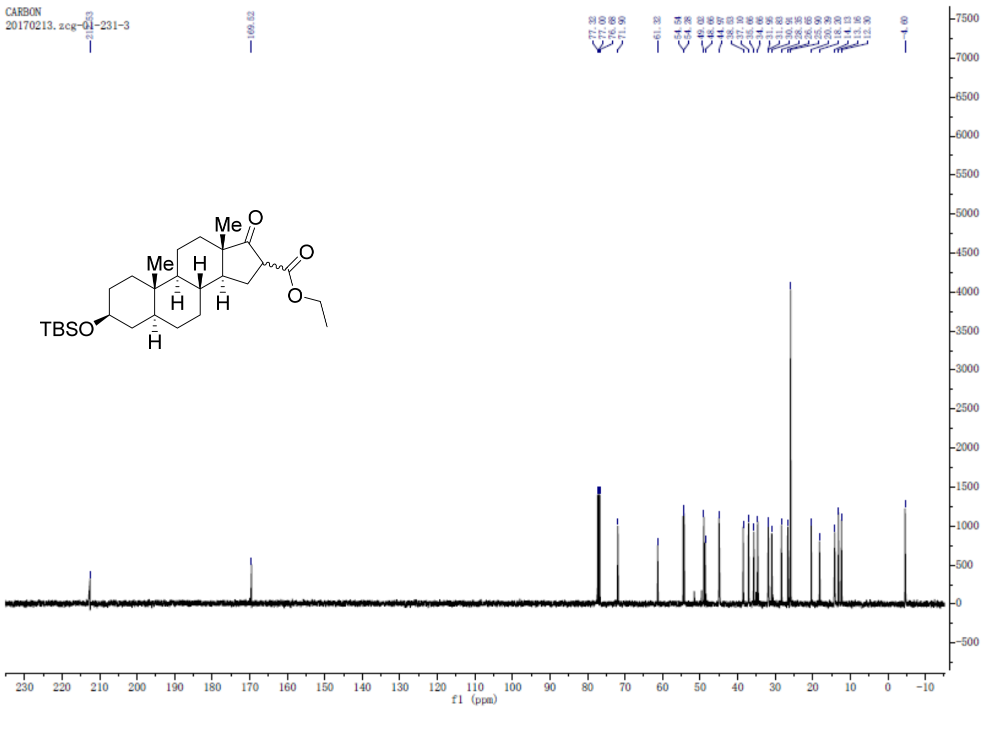

Supplementary Figure 45. ^{1}H and ^{13} C spectra of **13**.

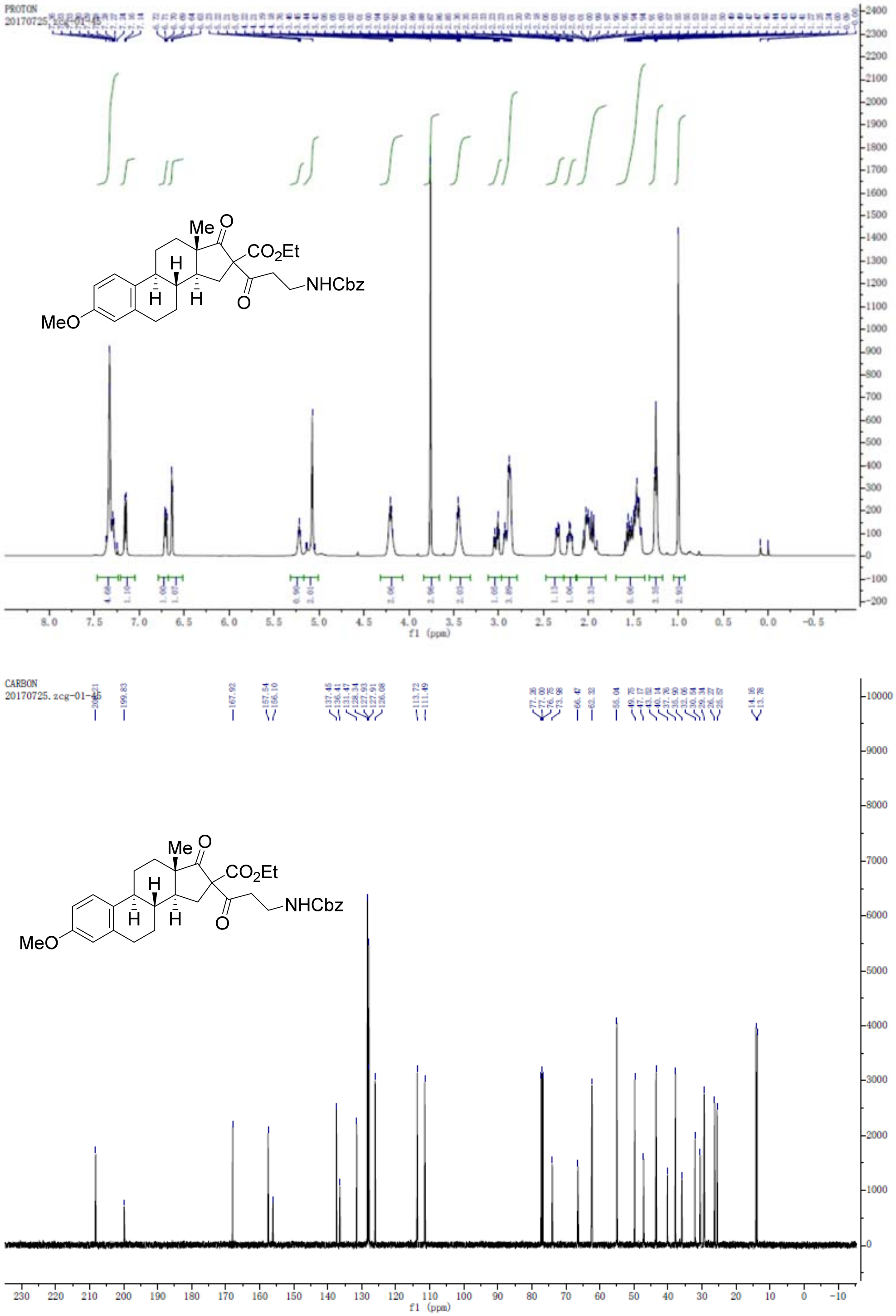

Supplementary Figure 46. ^{1}H and ^{13}C spectra of **S6a**.

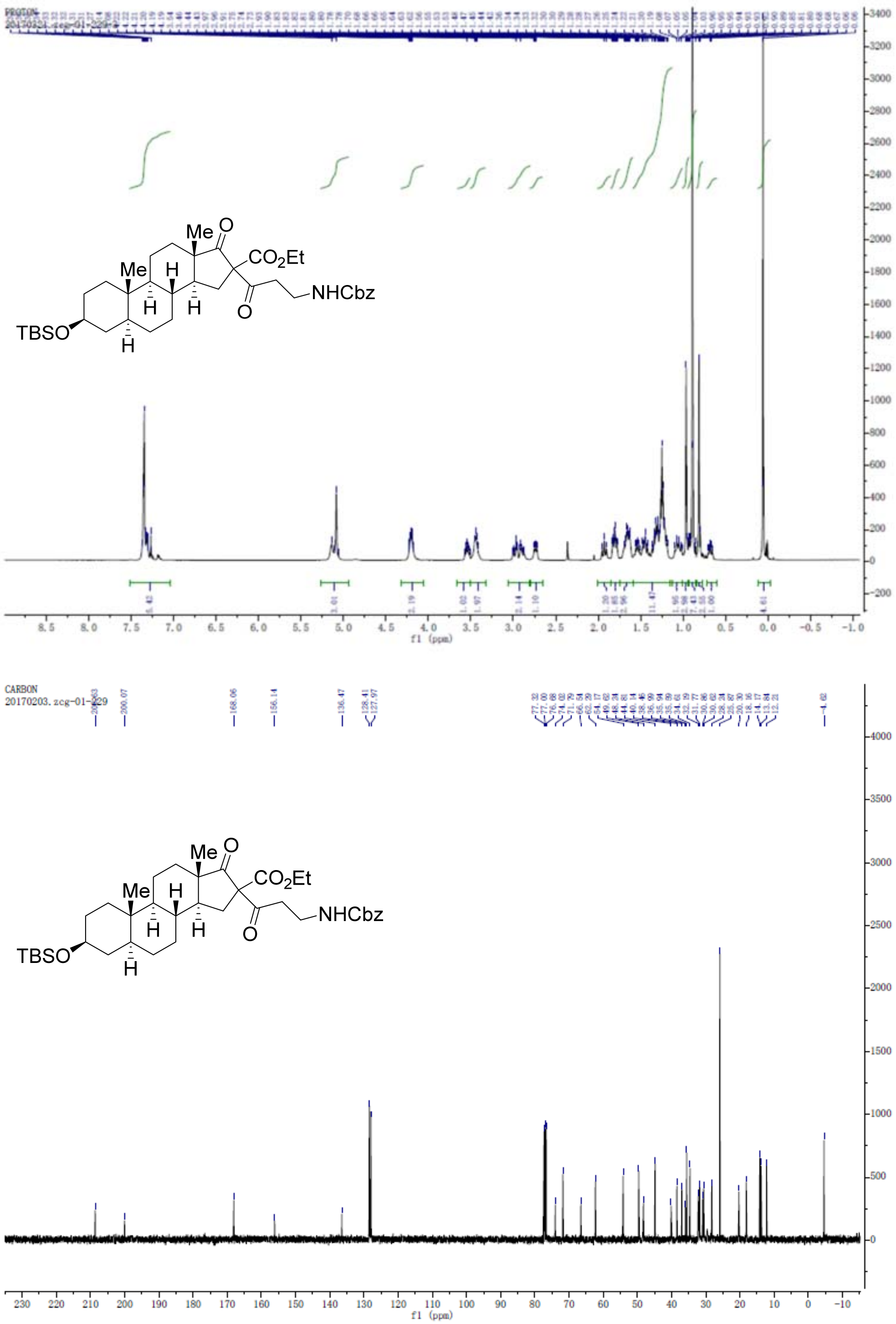

Supplementary Figure 47. ^{1}H and ^{13}C spectra of **S6b**.

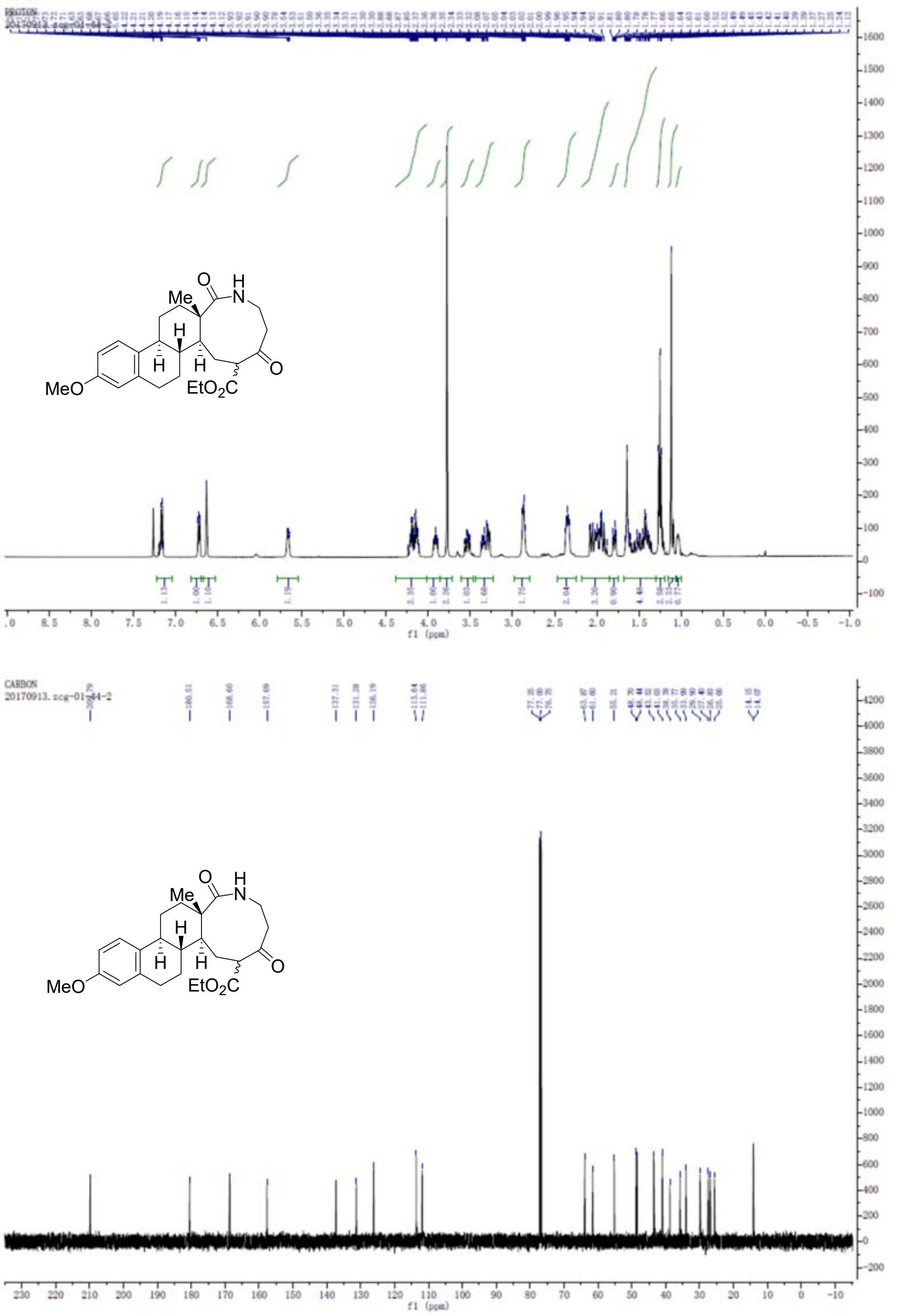

Supplementary Figure 48. ^{1}H and ^{13} C spectra of **12**.

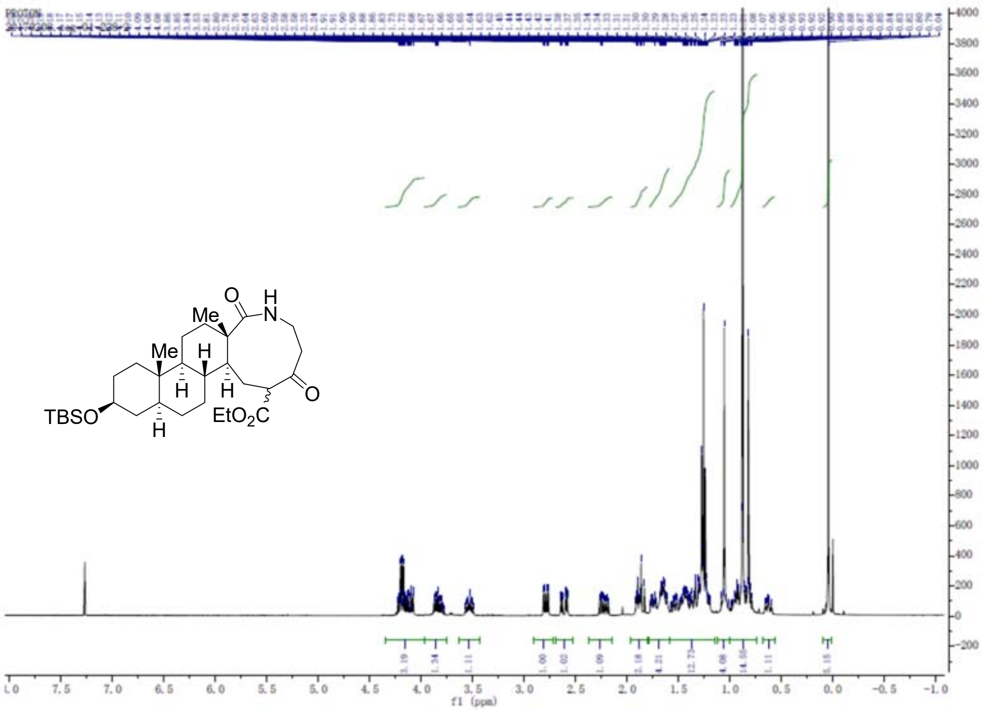

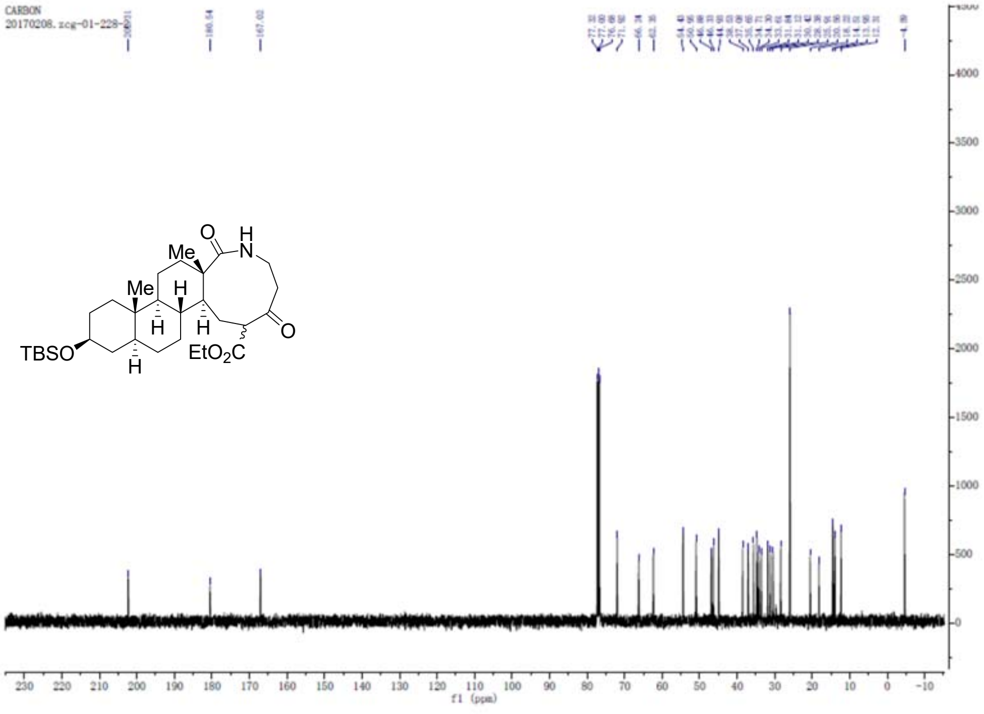

Supplementary Figure 49. ^{1}H and ^{13}C spectra of **17**.

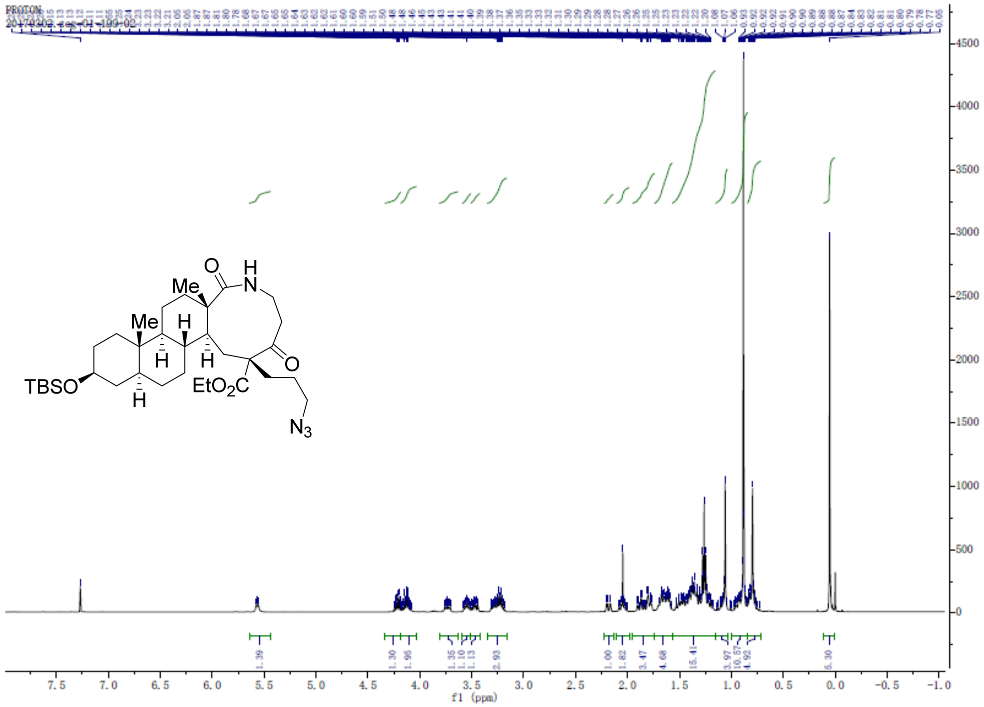

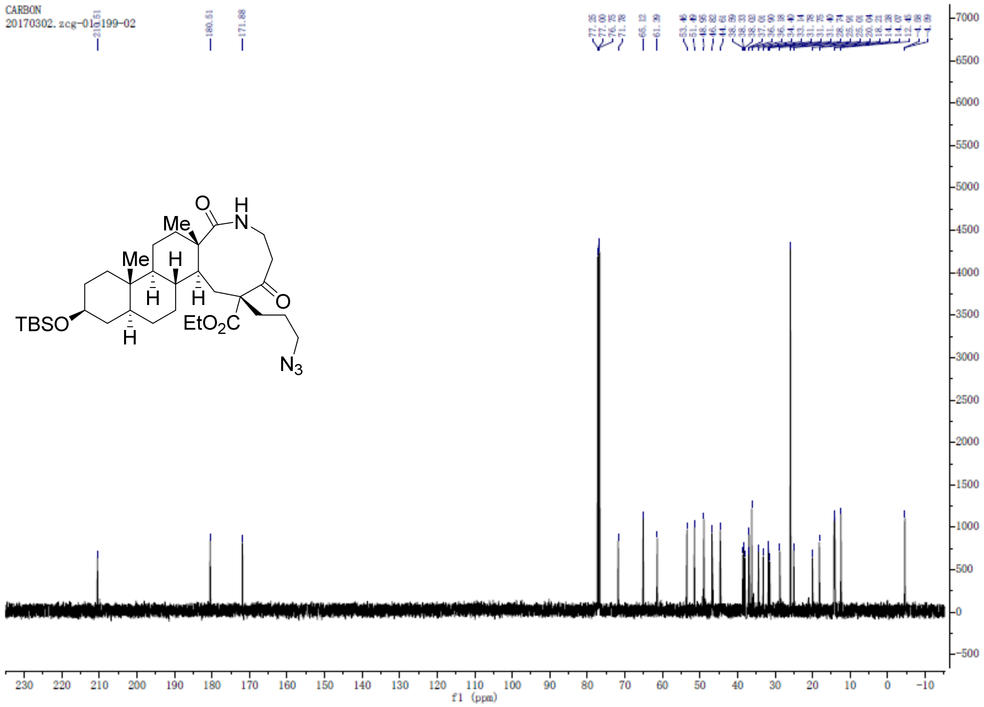

Supplementary Figure 50. ^{1}H and ^{13} C spectra of **S7**.

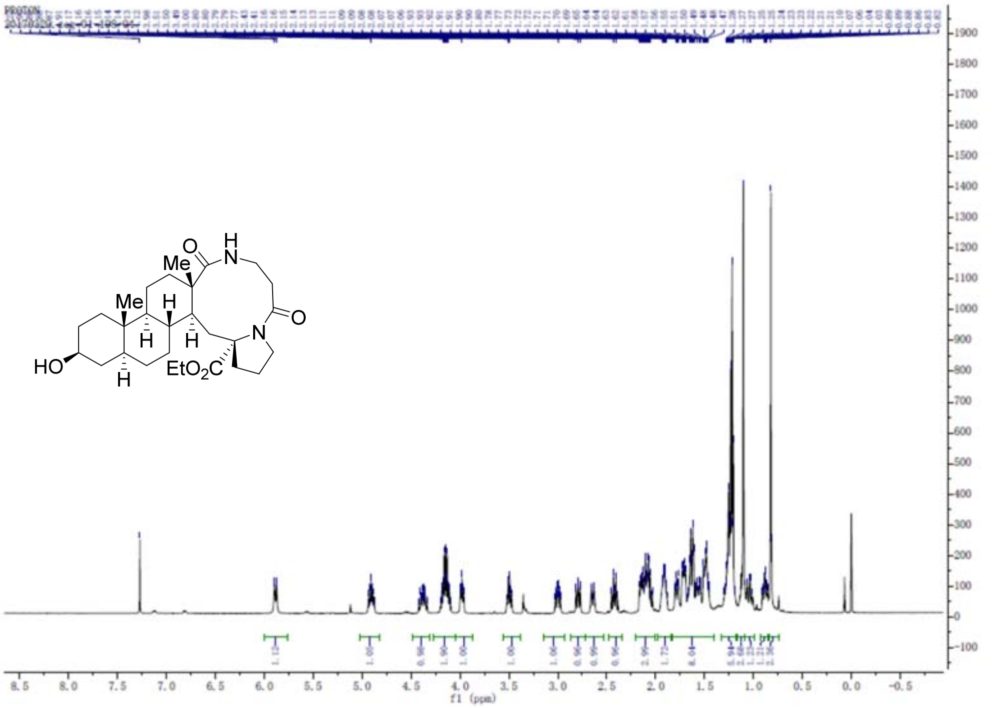

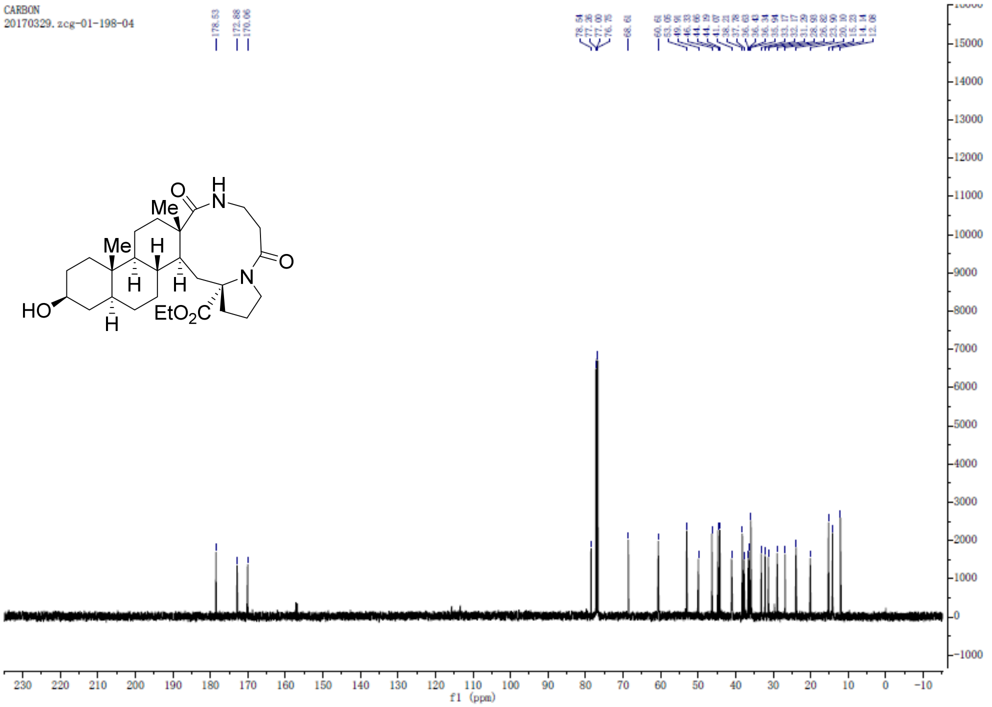

Supplementary Figure 51. ^{1}H and ^{13}C spectra of **18**.

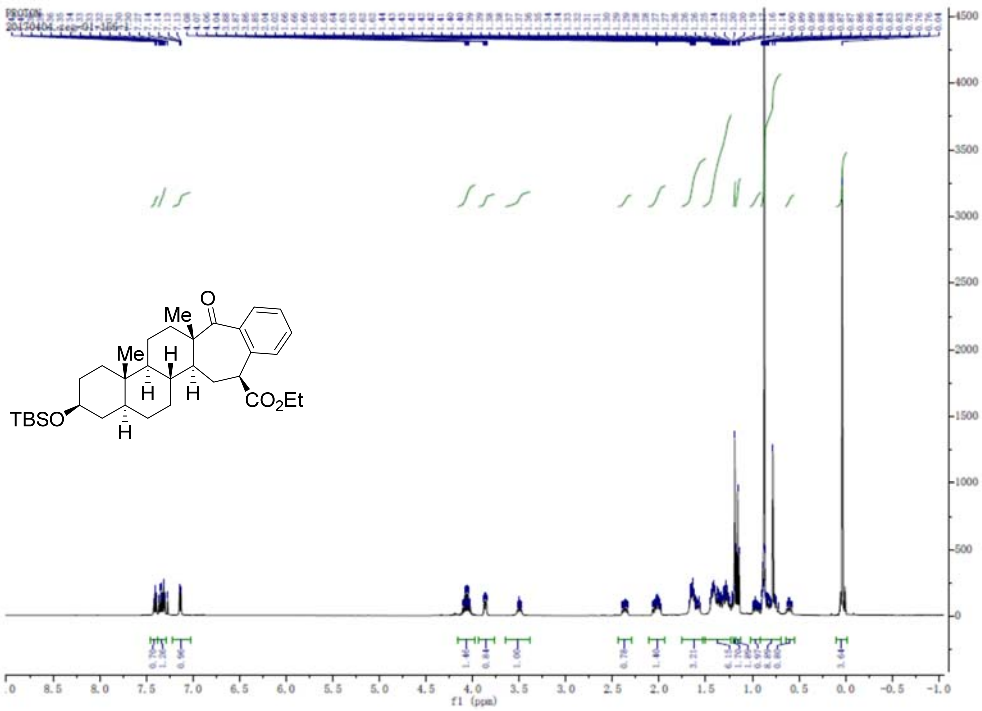

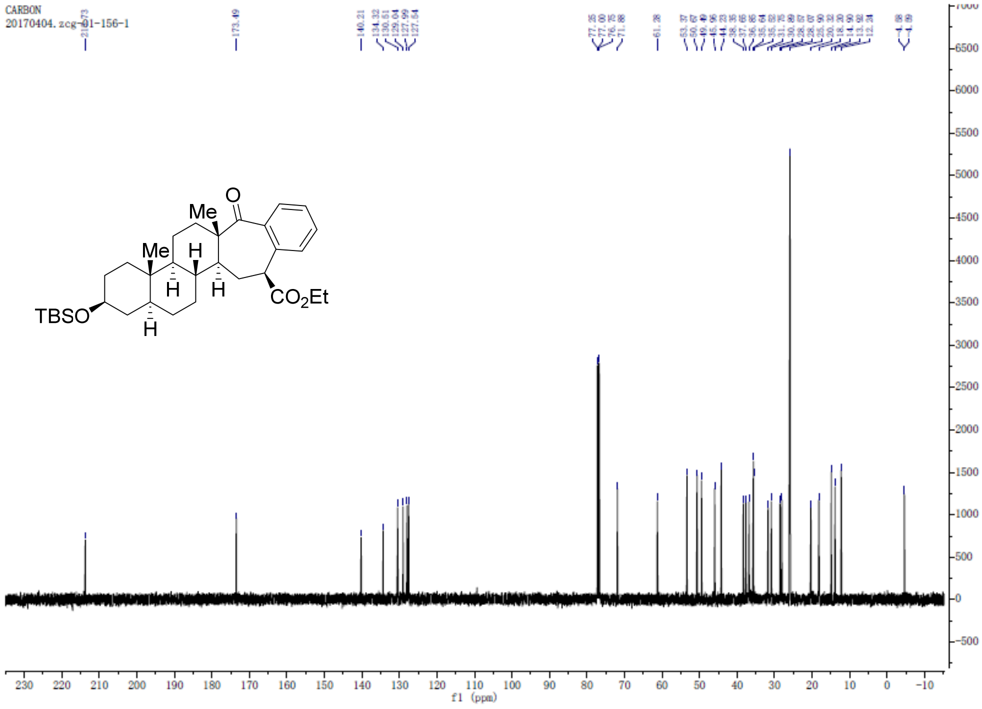

Supplementary Figure 52. ^{1}H, ^{13} C and DEPT 135 spectra of **14**.

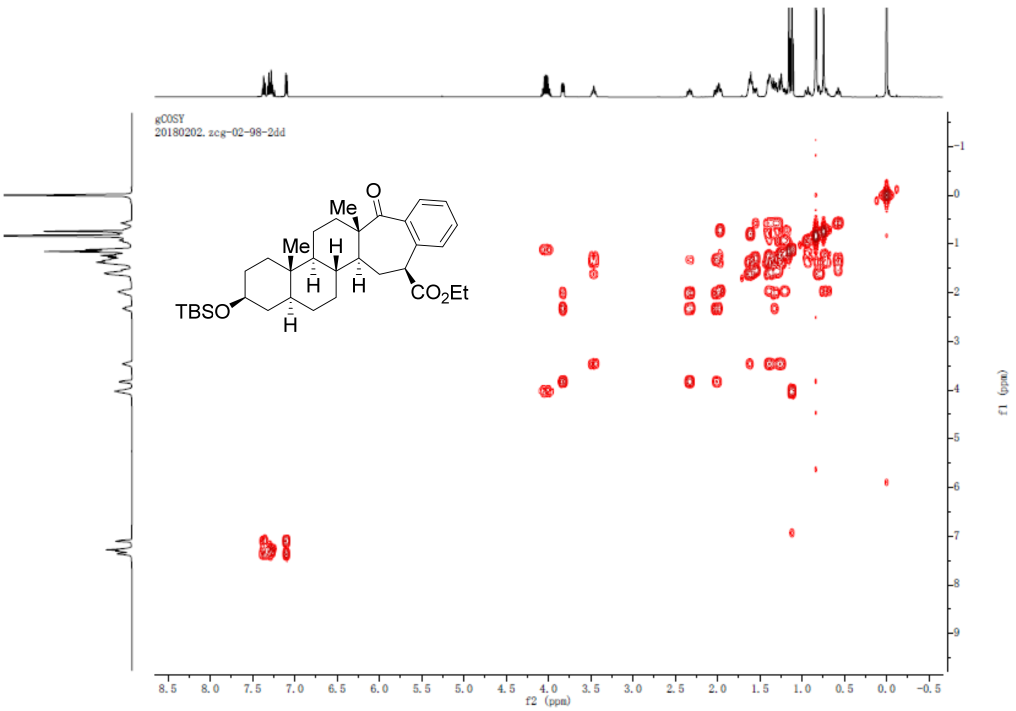

Supplementary Figure 53. H-H Cosy spectra of **14**.

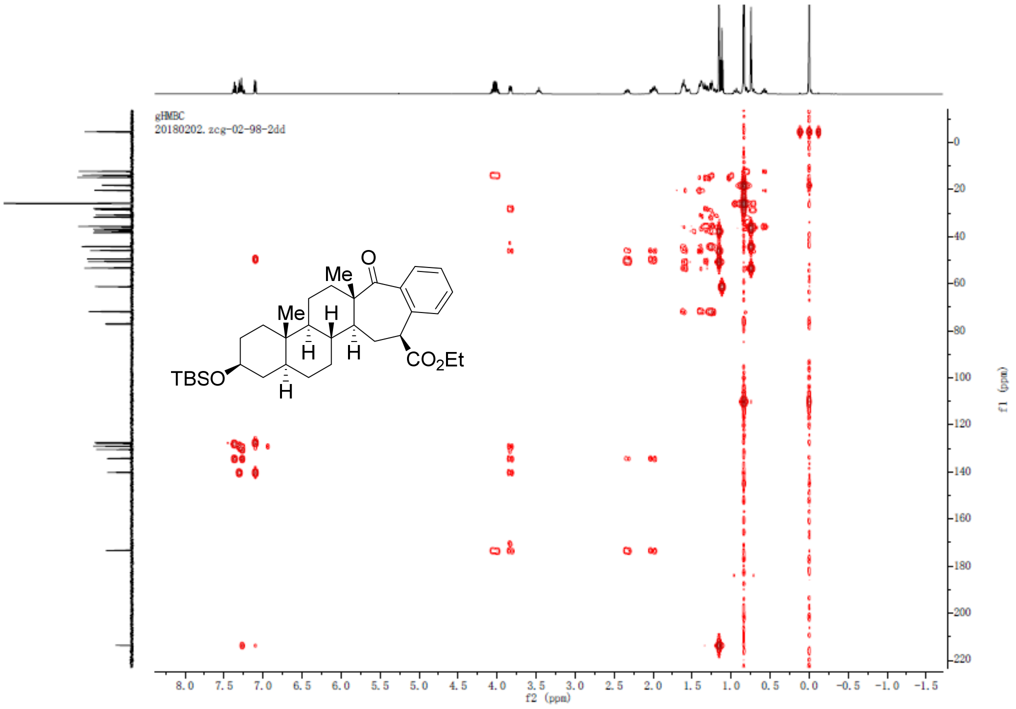

Supplementary Figure 54. HMBC spectra of **14**.

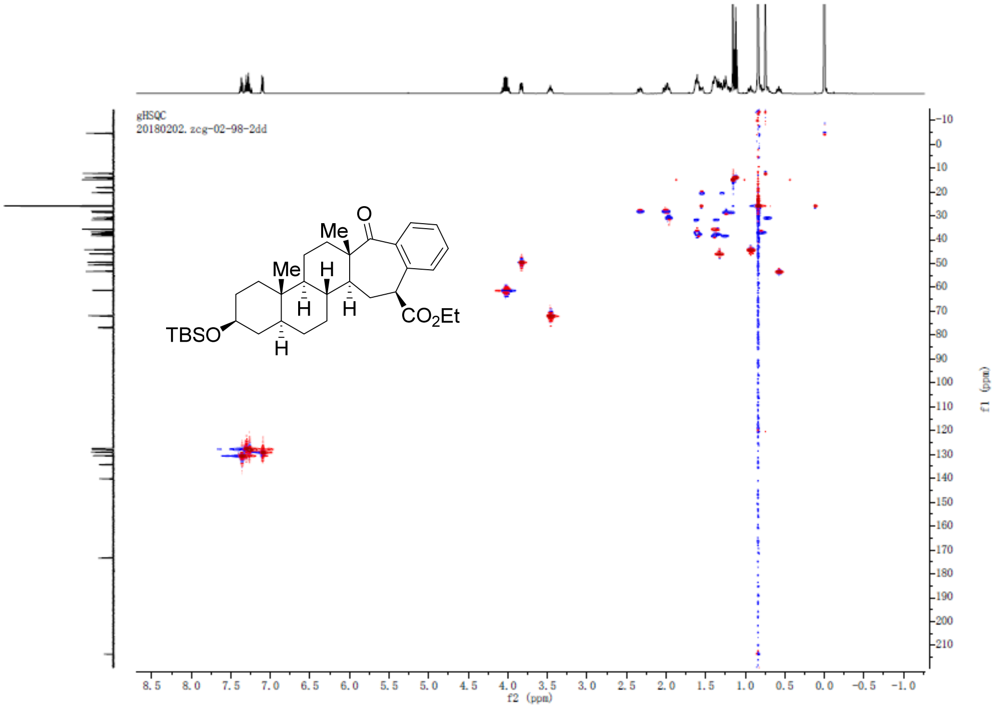

Supplementary Figure 55. HSQC spectra of **14**.

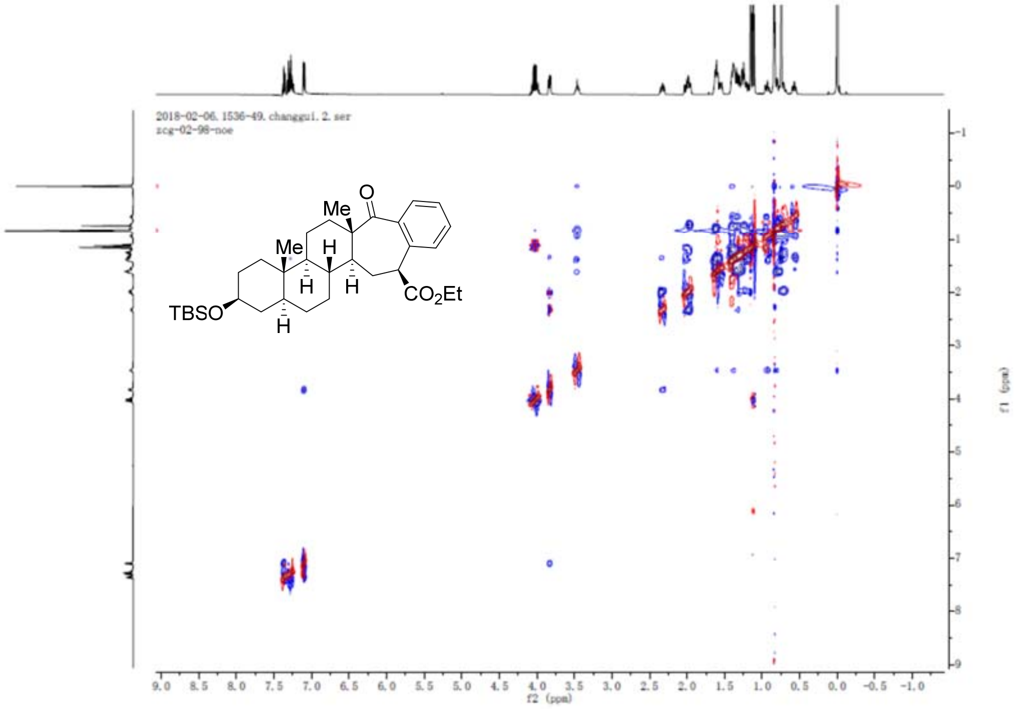

Supplementary Figure 56. 2D Noesy of 14.

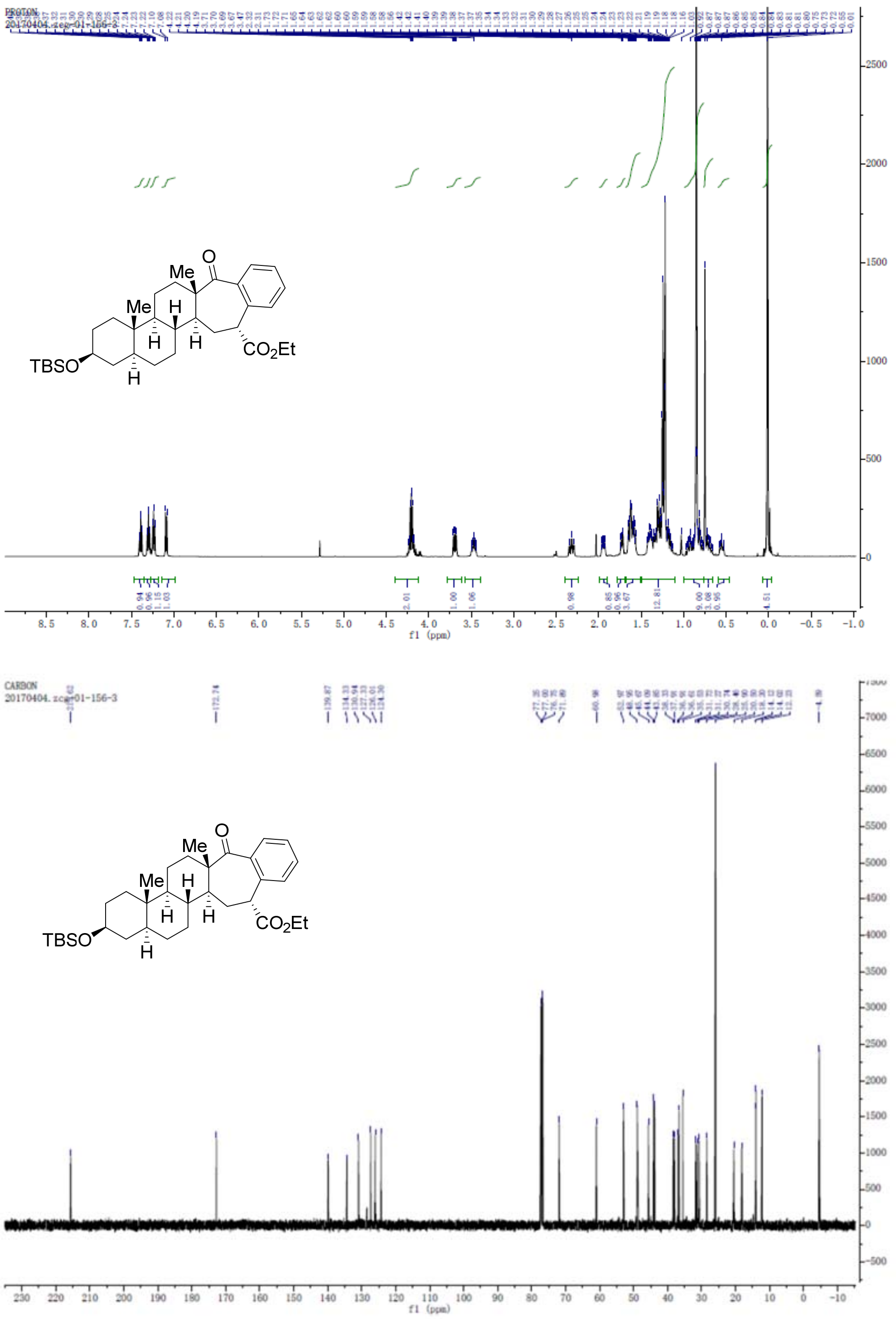

Supplementary Figure 57. ^{1}H and ^{13}C spectra of **15**.

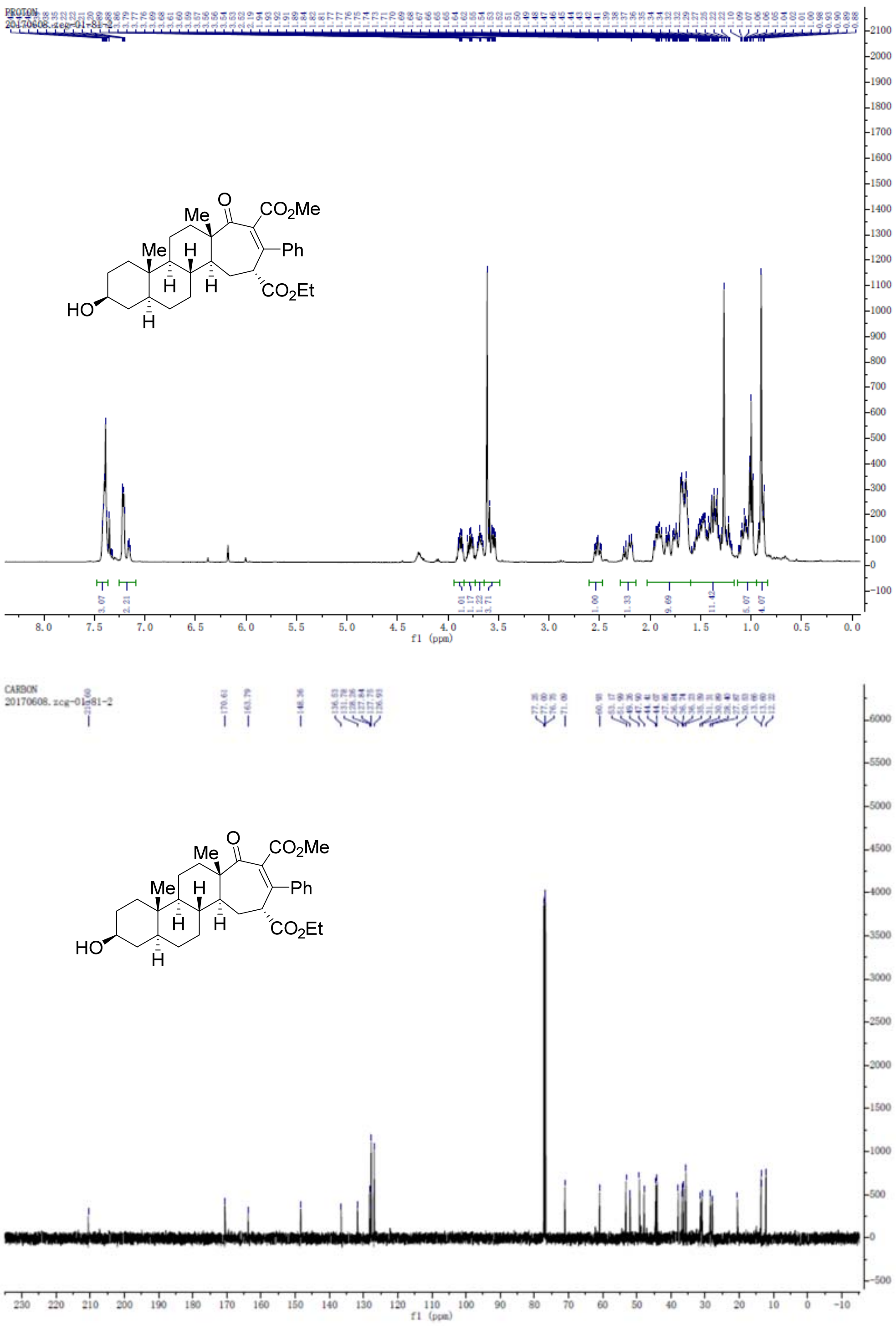

Supplementary Figure 58. ^{1}H and ^{13} C spectra of **16**.

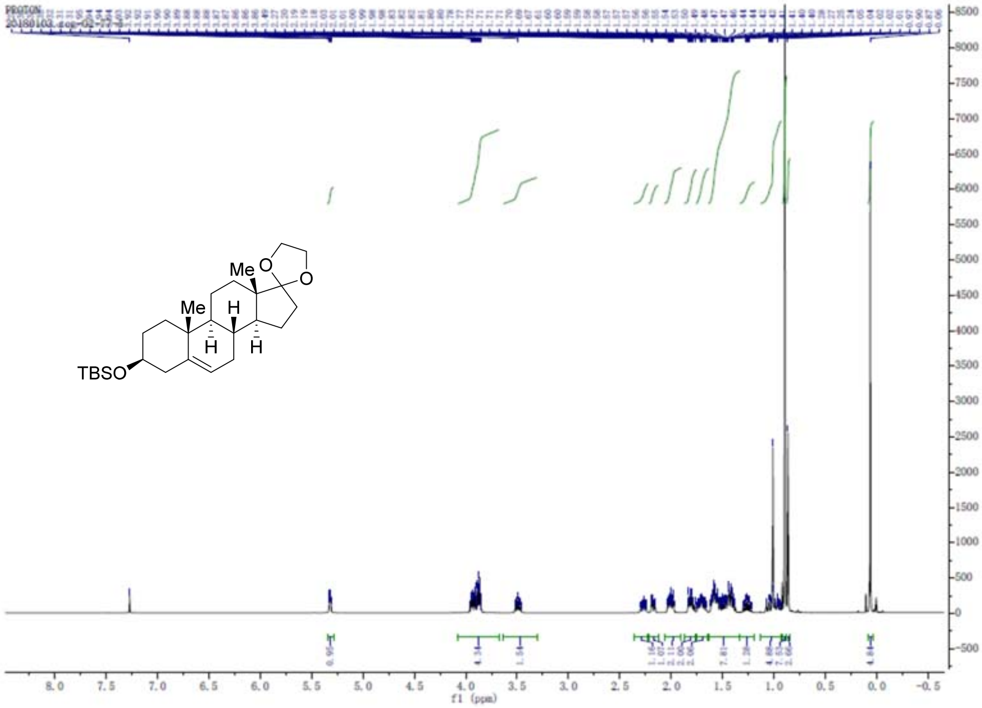

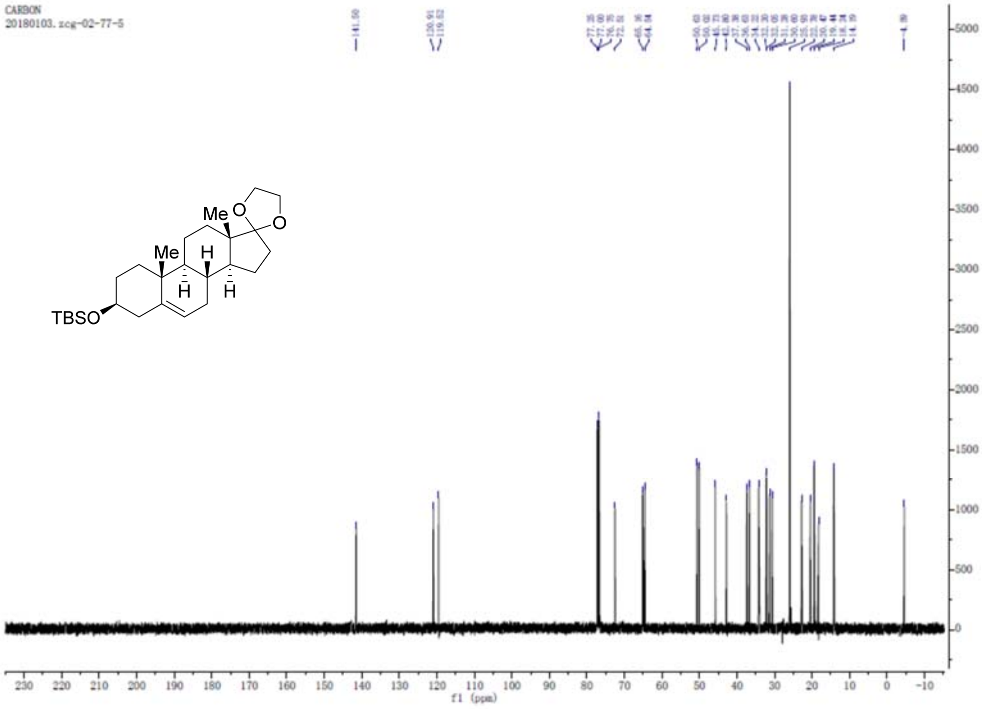

Supplementary Figure 59. ^{1}H and ^{13}C spectra of **19g**.

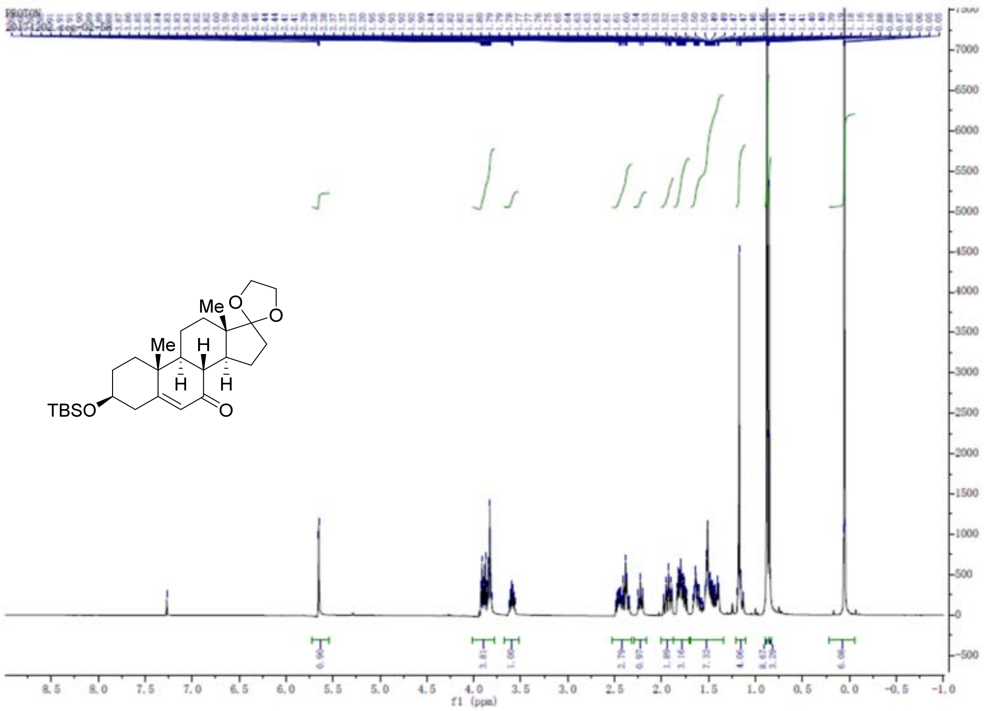

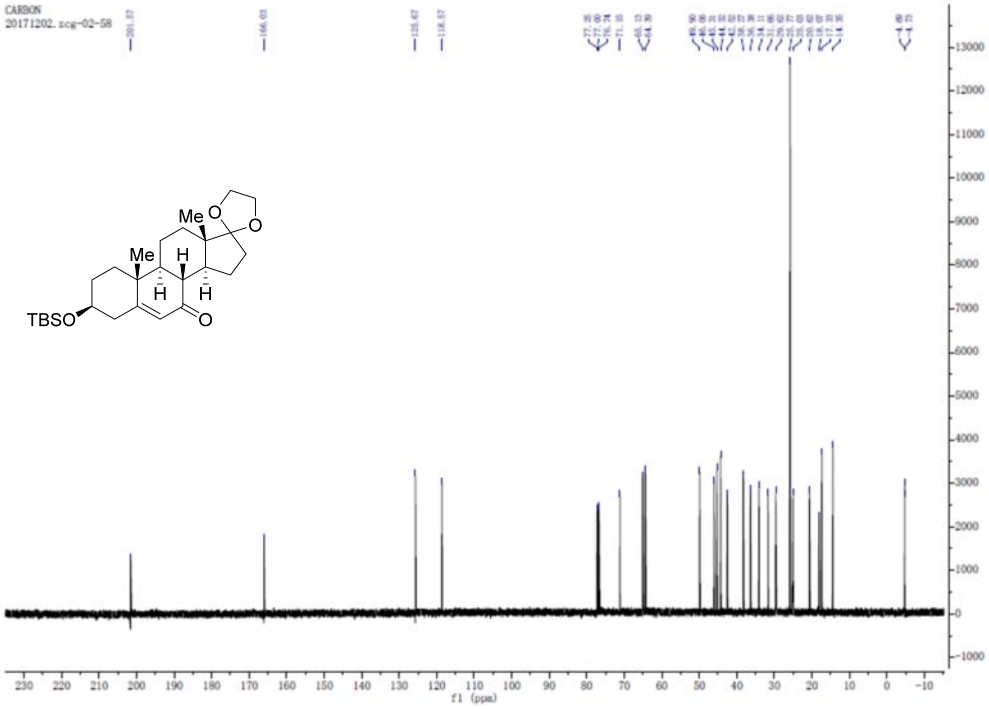

Supplementary Figure 60. ^{1}H and ^{13}C spectra of **20g**.

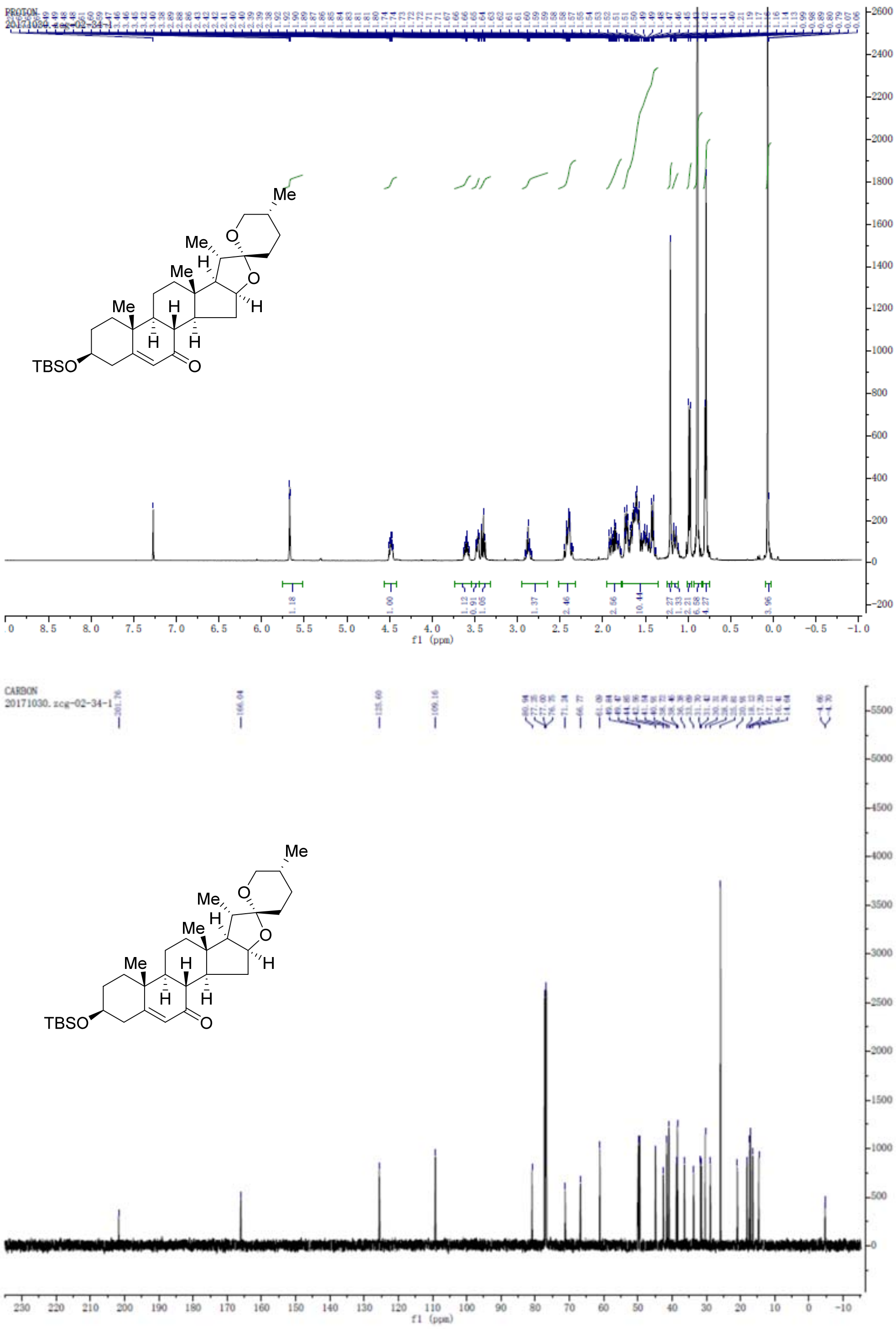

Supplementary Figure 61. ^{1}H and ^{13}C spectra of **20h**.

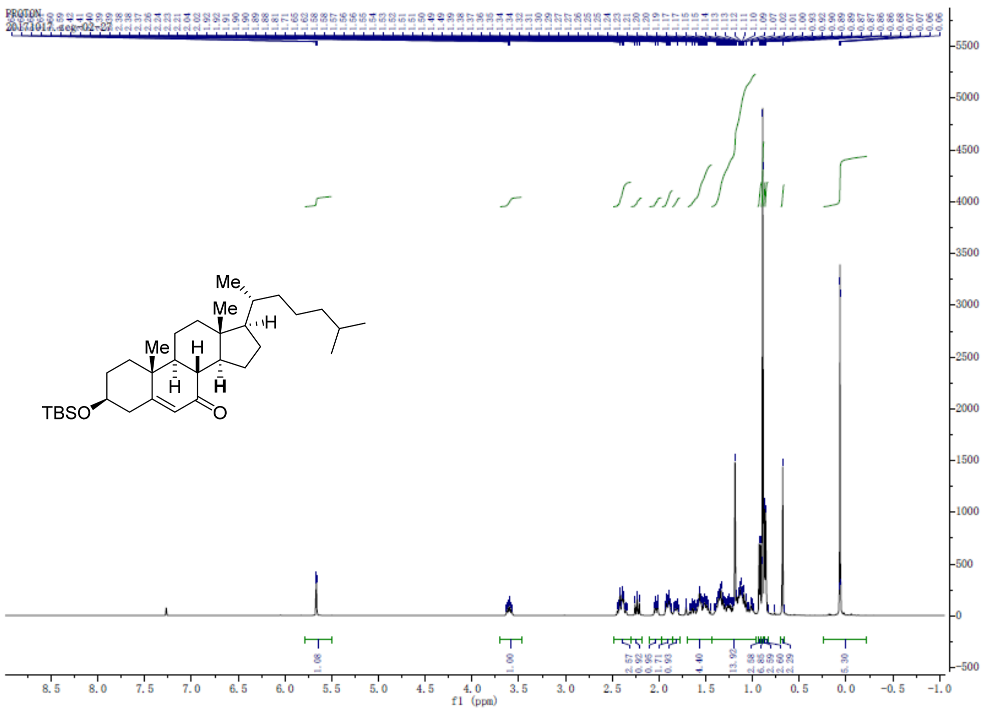

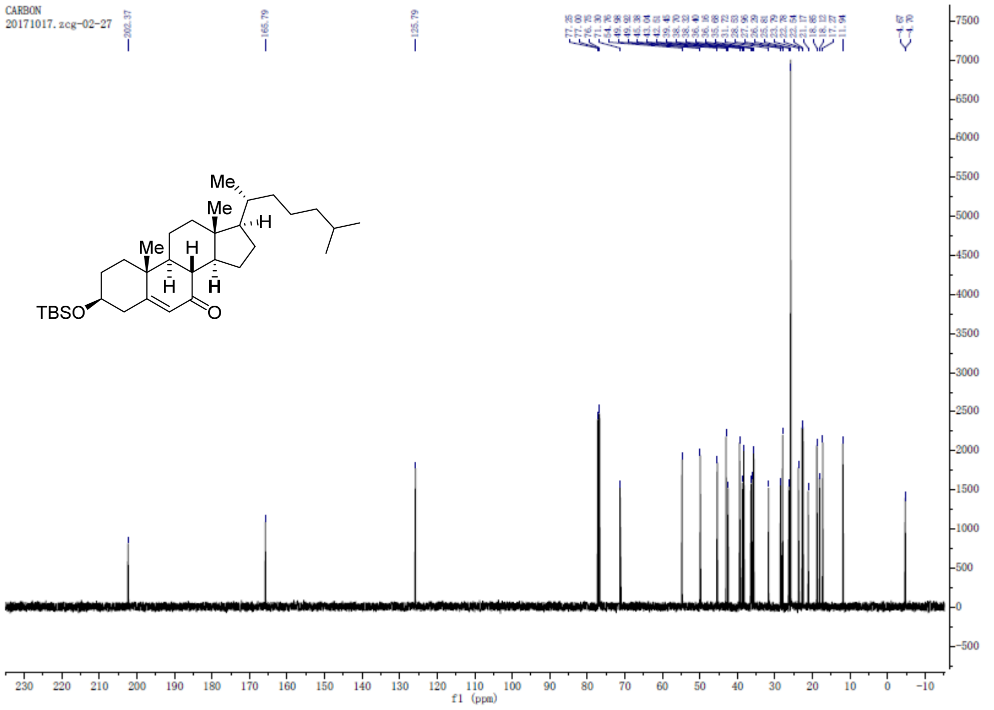

Supplementary Figure 62. ^{1}H and ^{13} C spectra of **20i**.

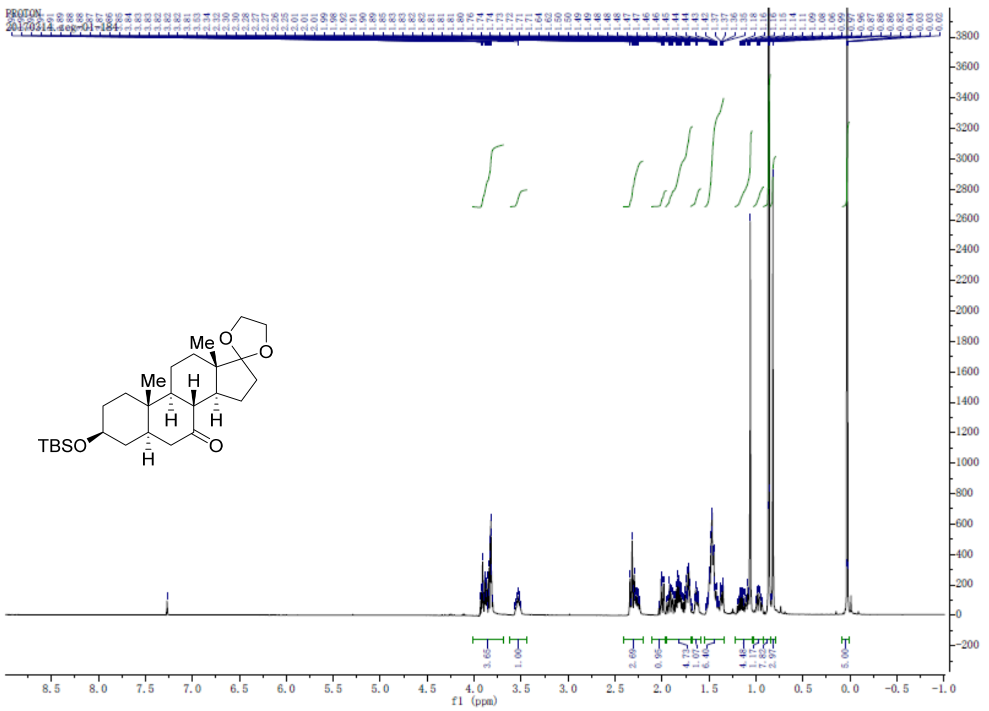

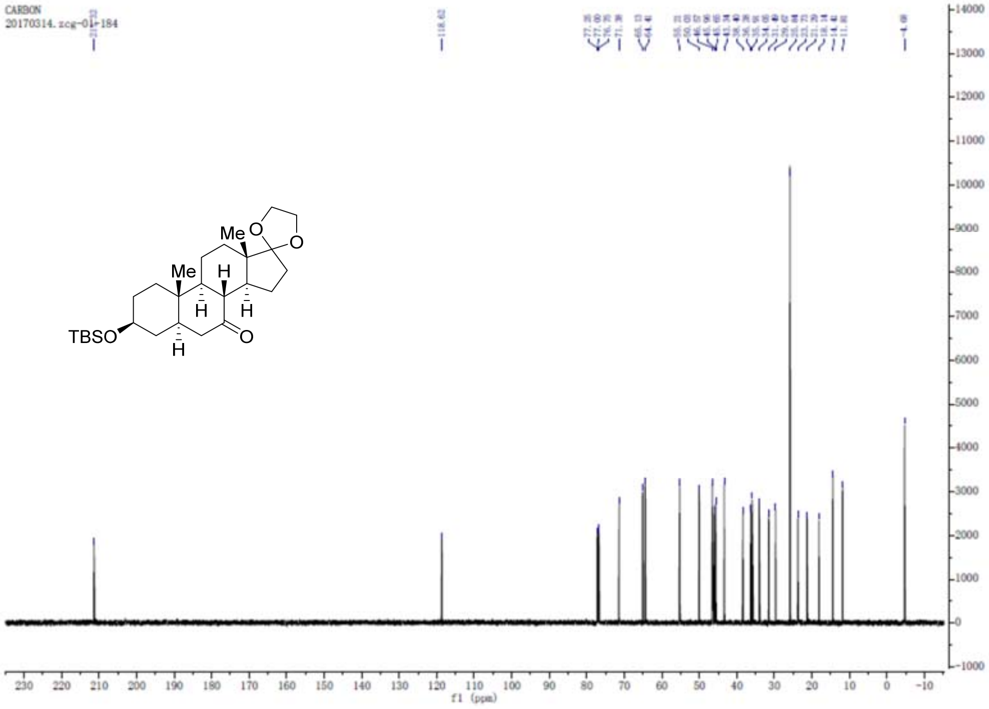

Supplementary Figure 63. ^{1}H and ^{13}C spectra of **S8g**.

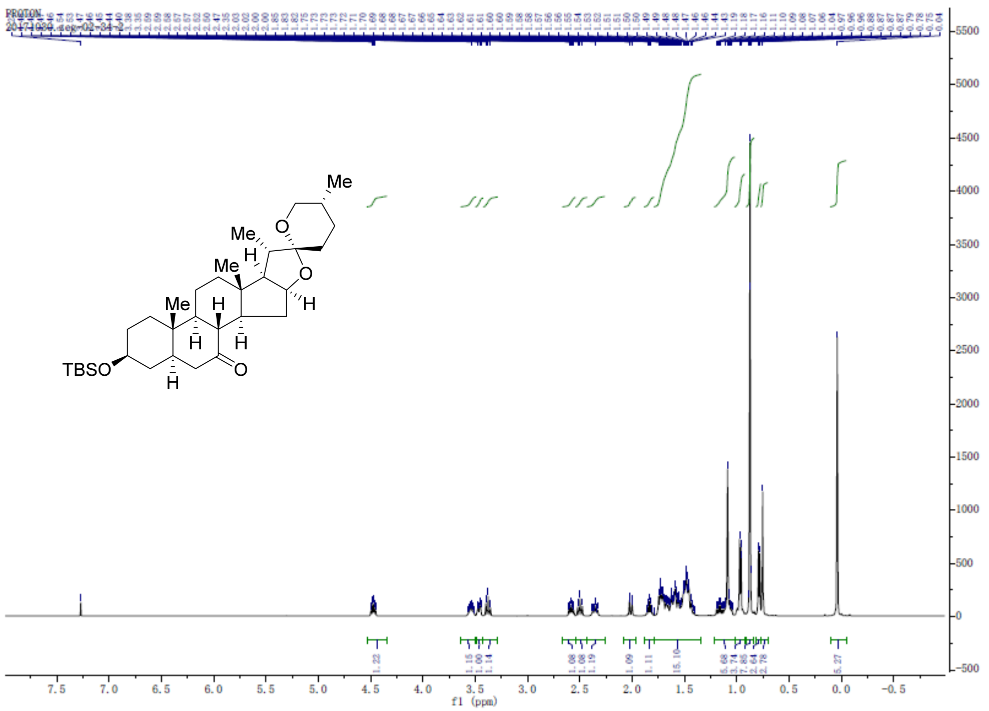

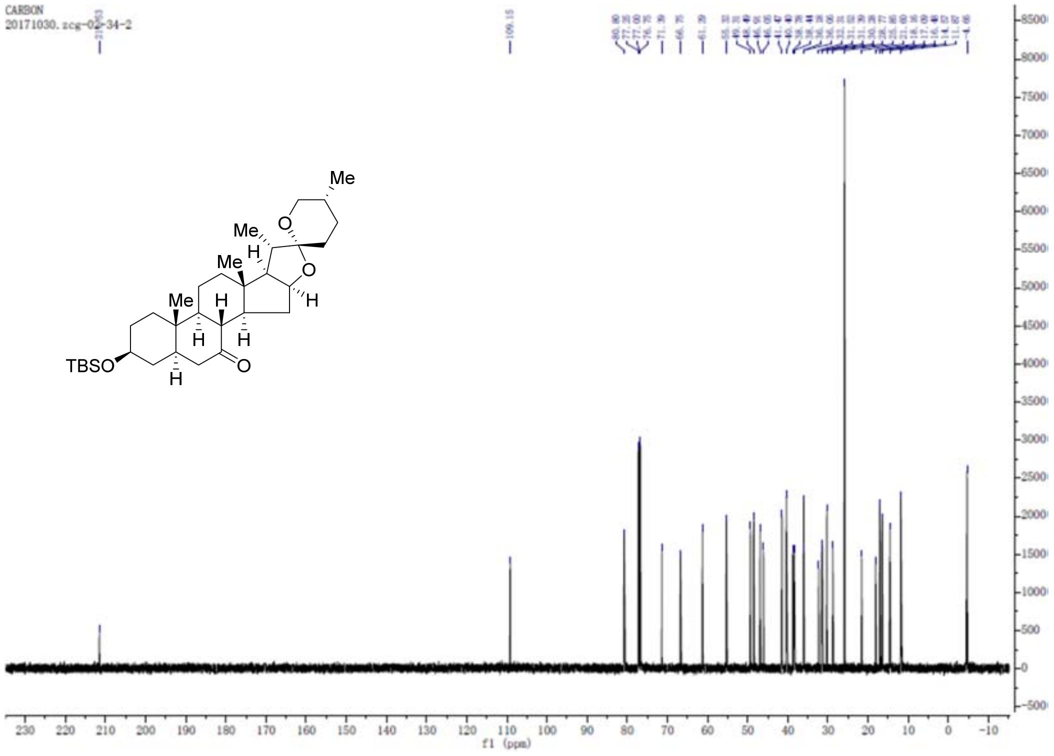

Supplementary Figure 64. ^{1}H and ^{13} C spectra of **S8h**.

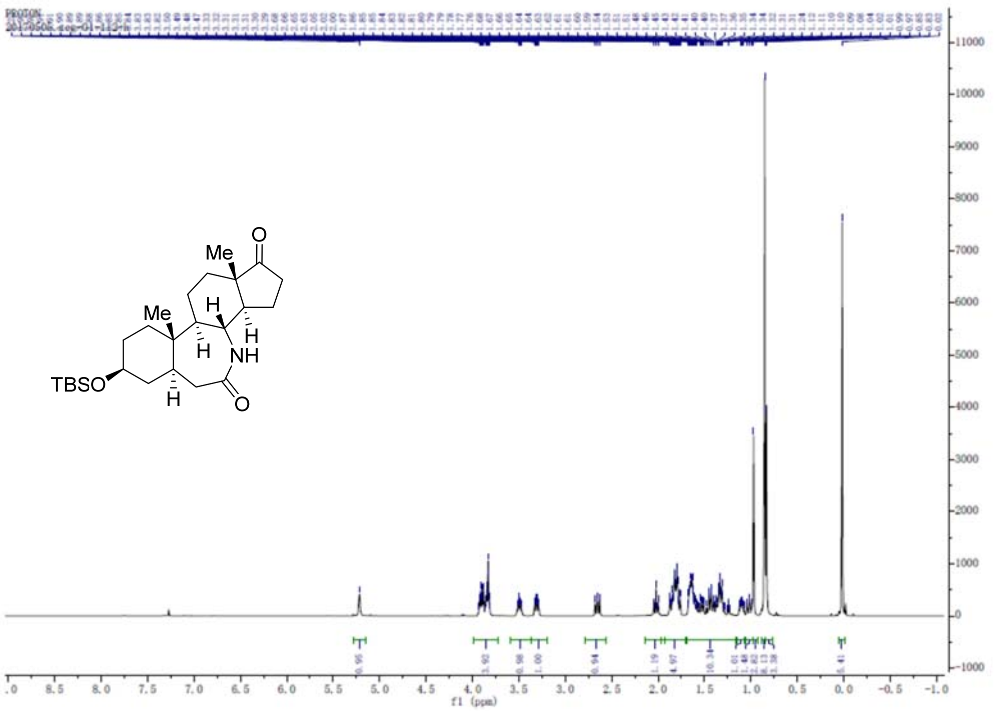

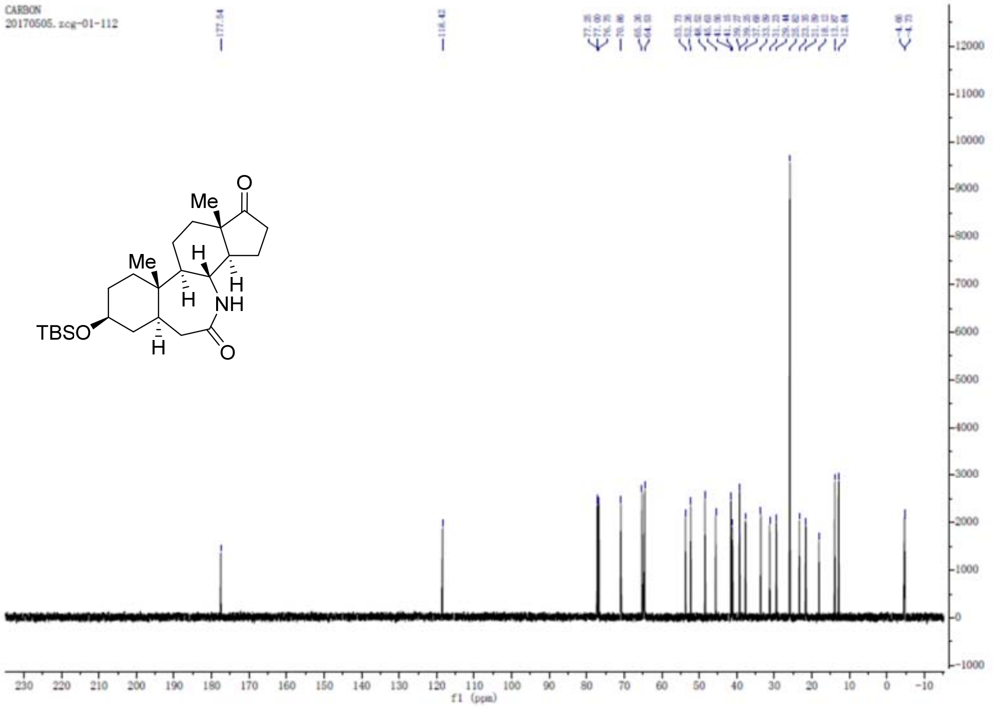

Supplementary Figure 65. ^{1}H and ^{13}C spectra of **S9g**.

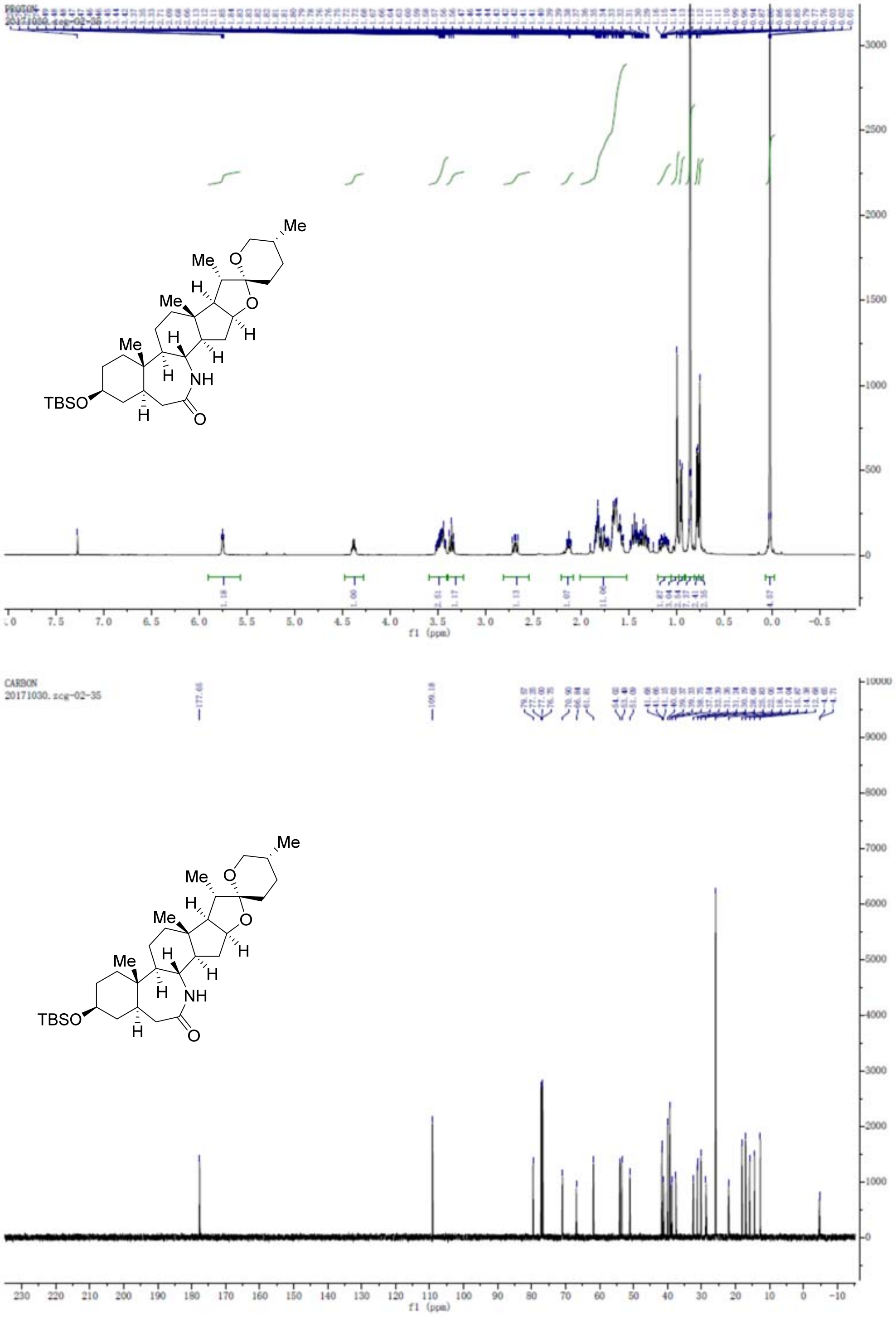

Supplementary Figure 66. ^{1}H and ^{13}C spectra of **S9h**.

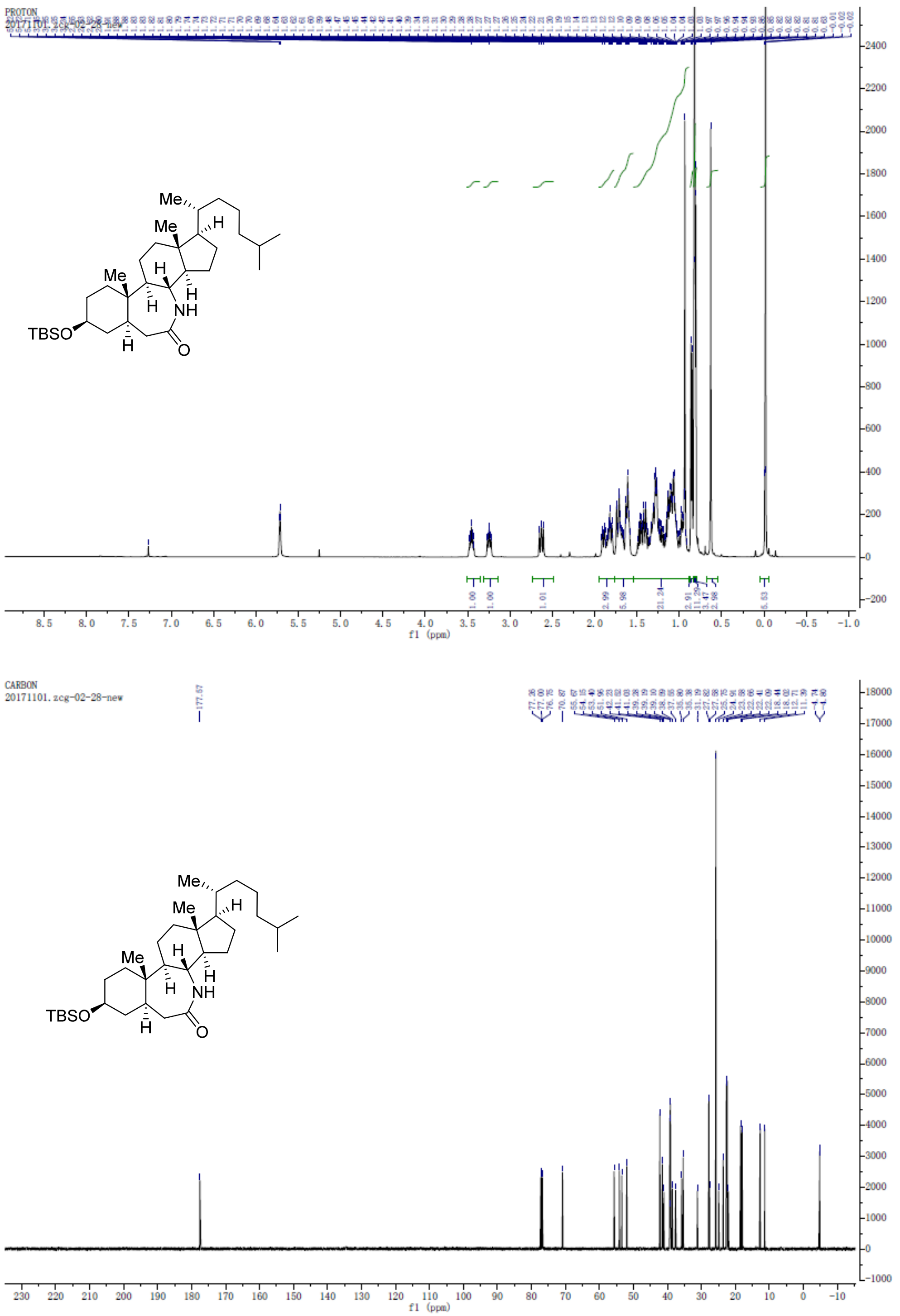

Supplementary Figure 67. ^{1}H and ^{13}C spectra of **S9i**.

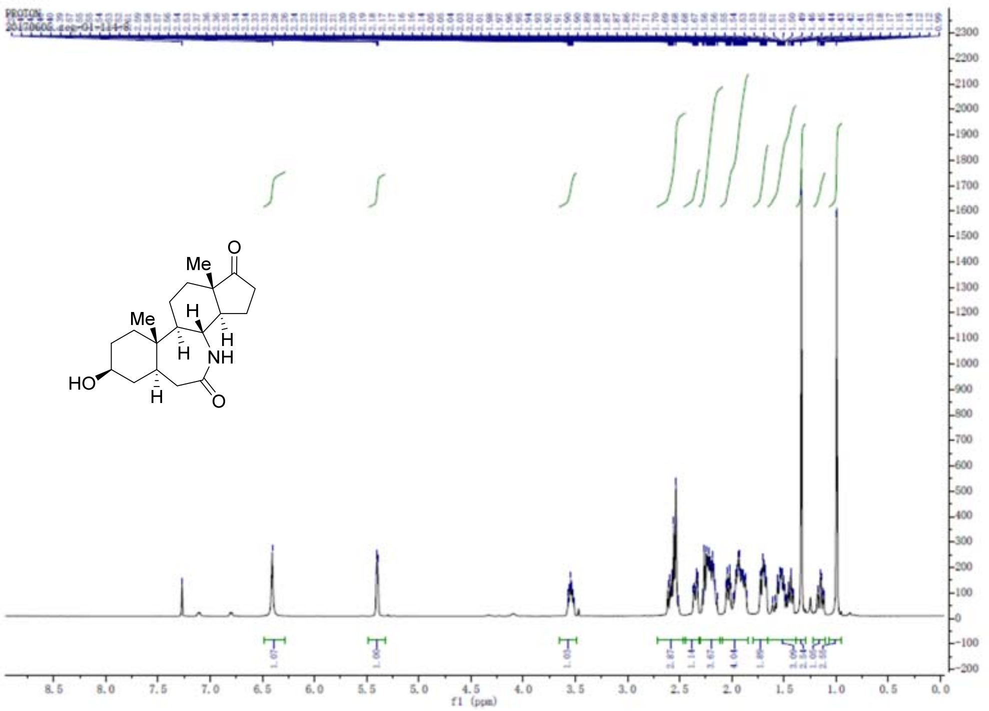

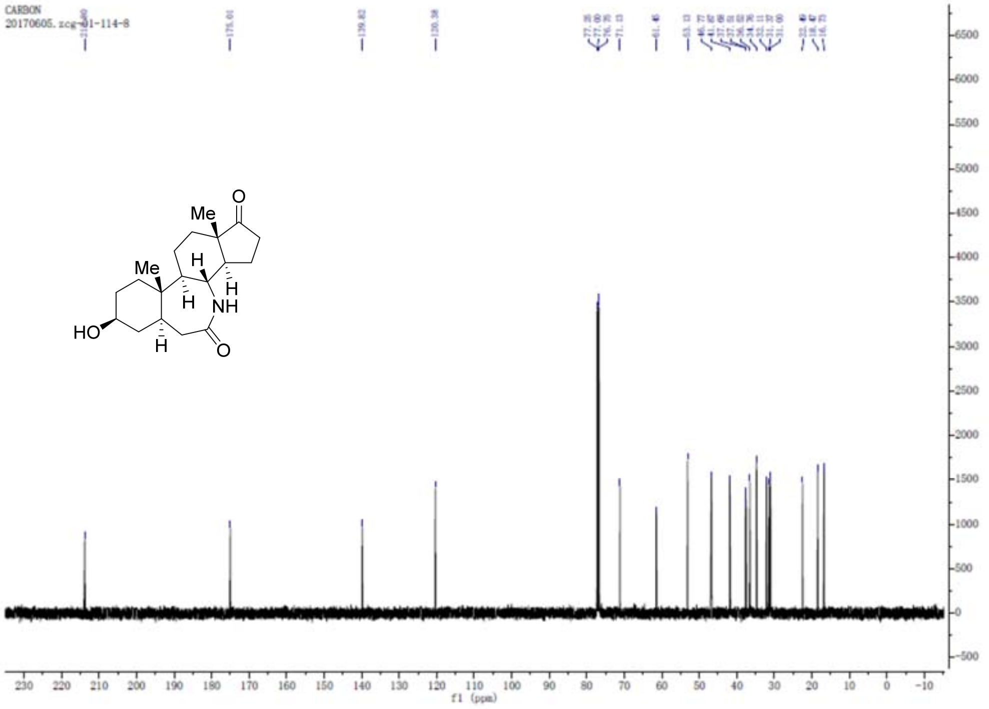

Supplementary Figure 68. ^{1}H and ^{13}C spectra of **21g**.

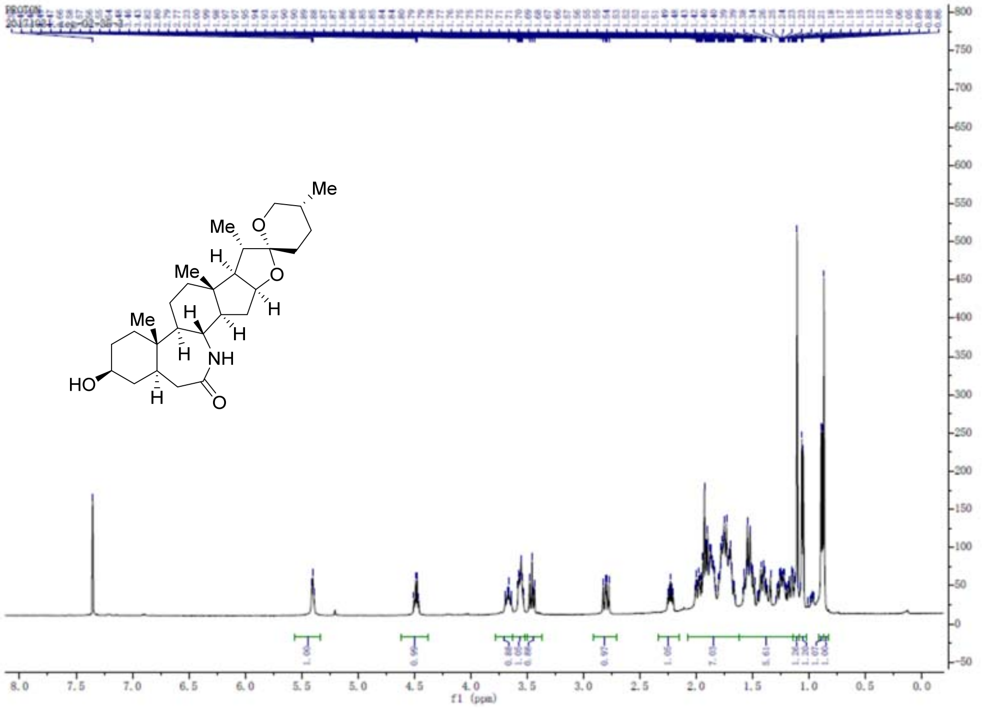

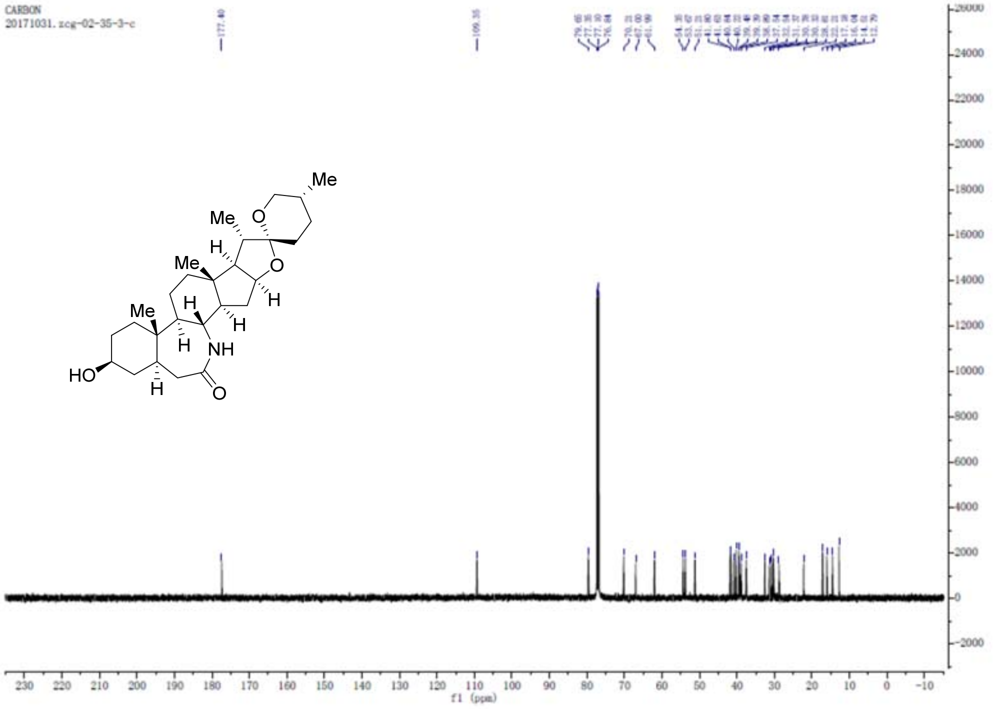

Supplementary Figure 69. ^{1}H and ^{13} C spectra of **21h**.

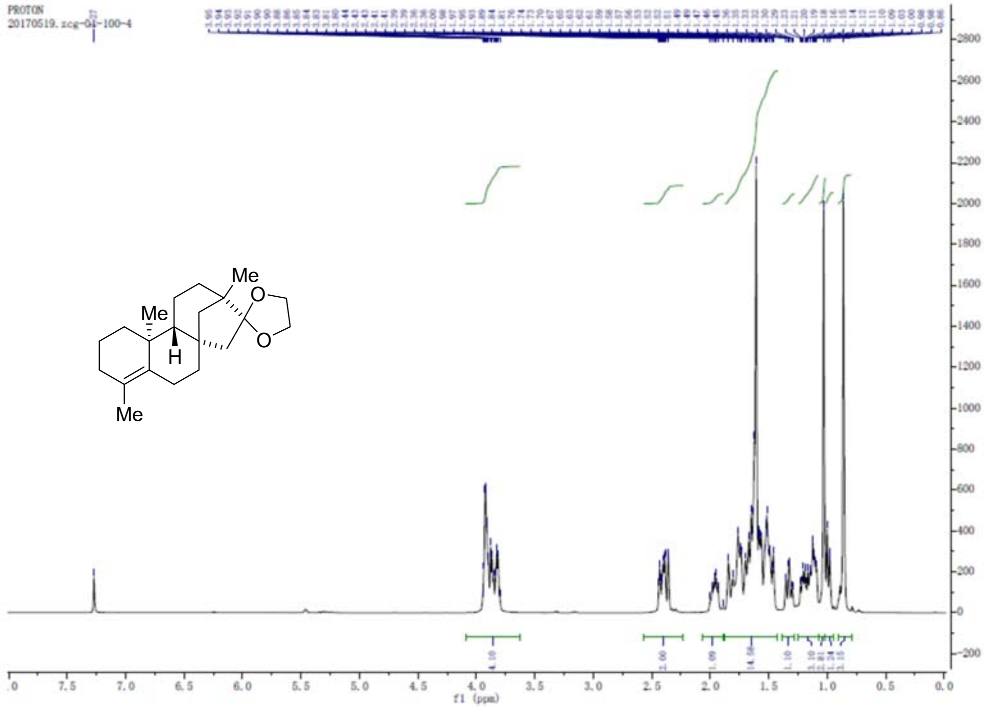

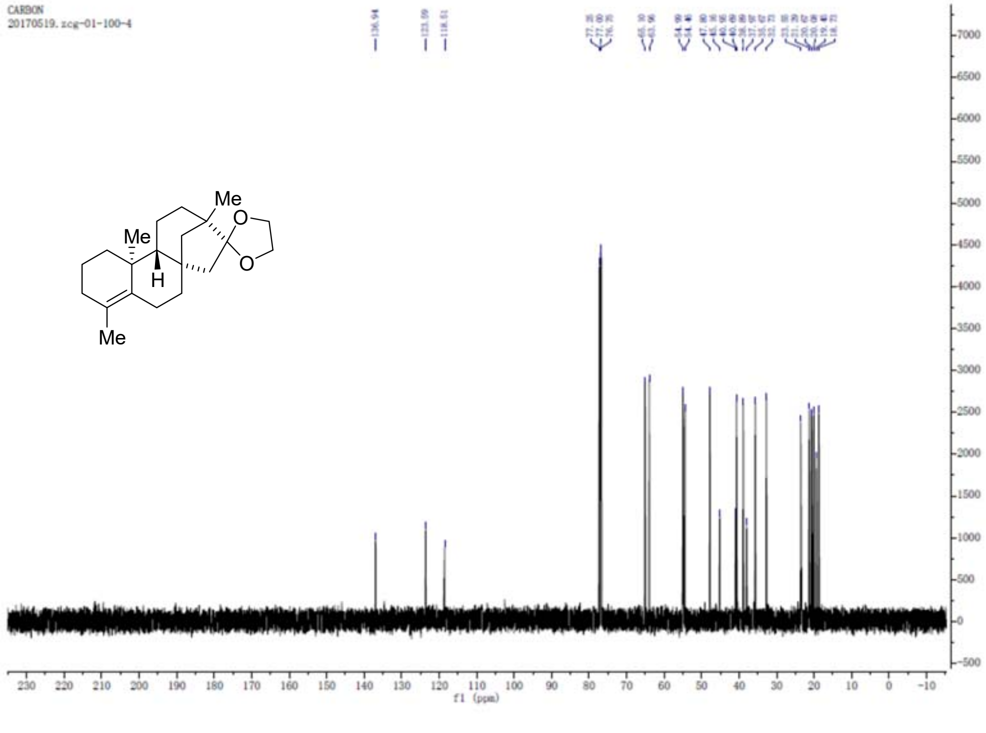

Supplementary Figure 70. ^{1}H and ^{13} C spectra of **22**.

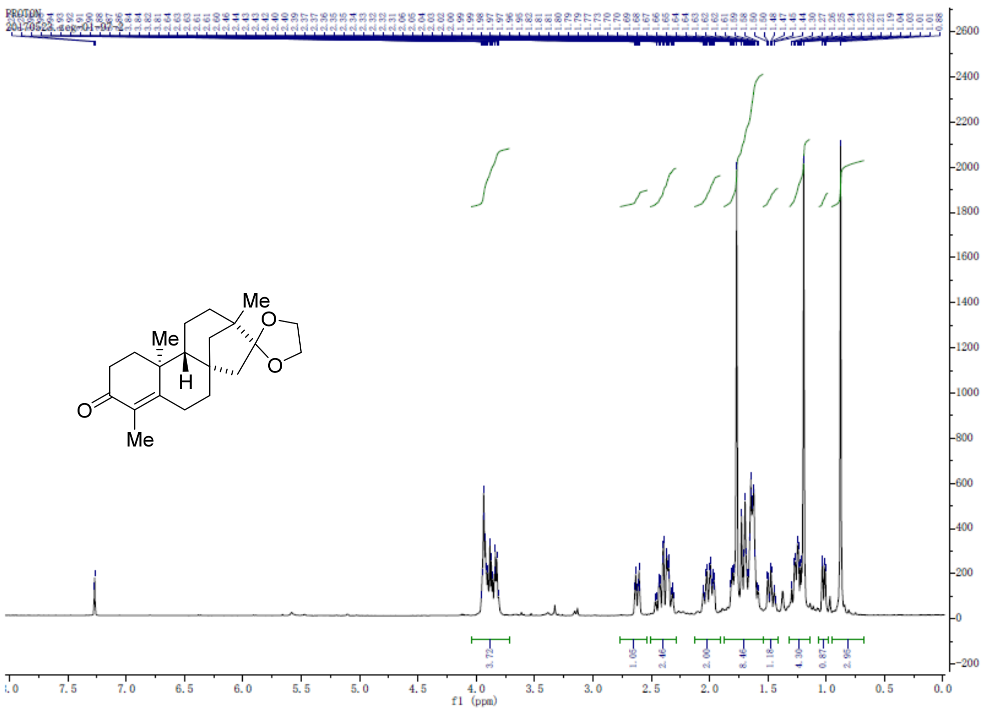

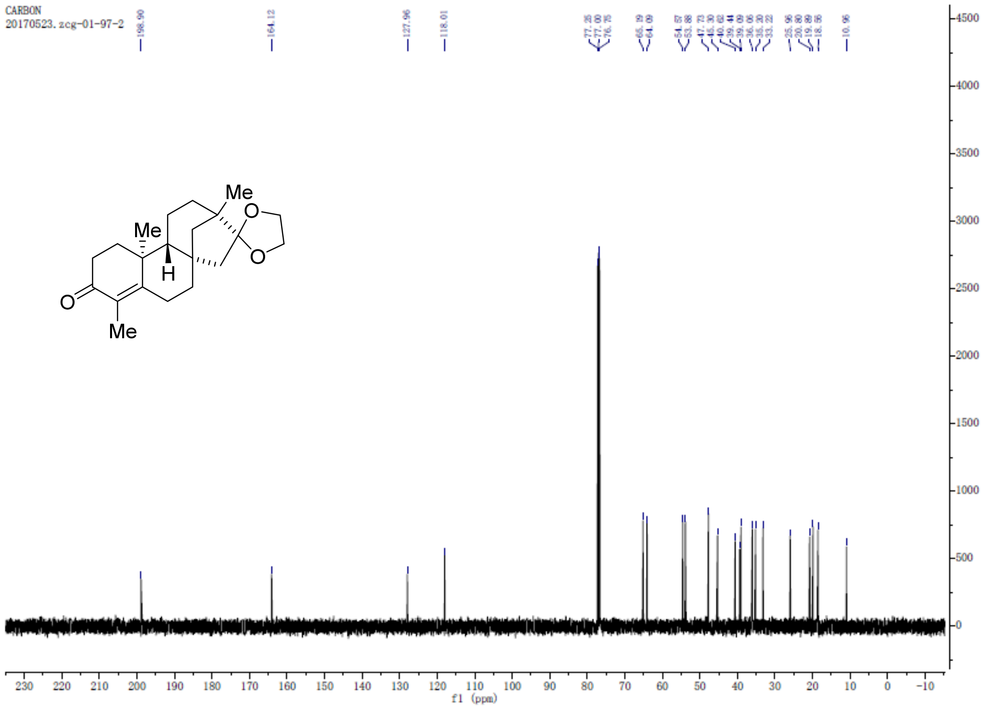

Supplementary Figure 71. ^{1}H and ^{13} C spectra of **23**.

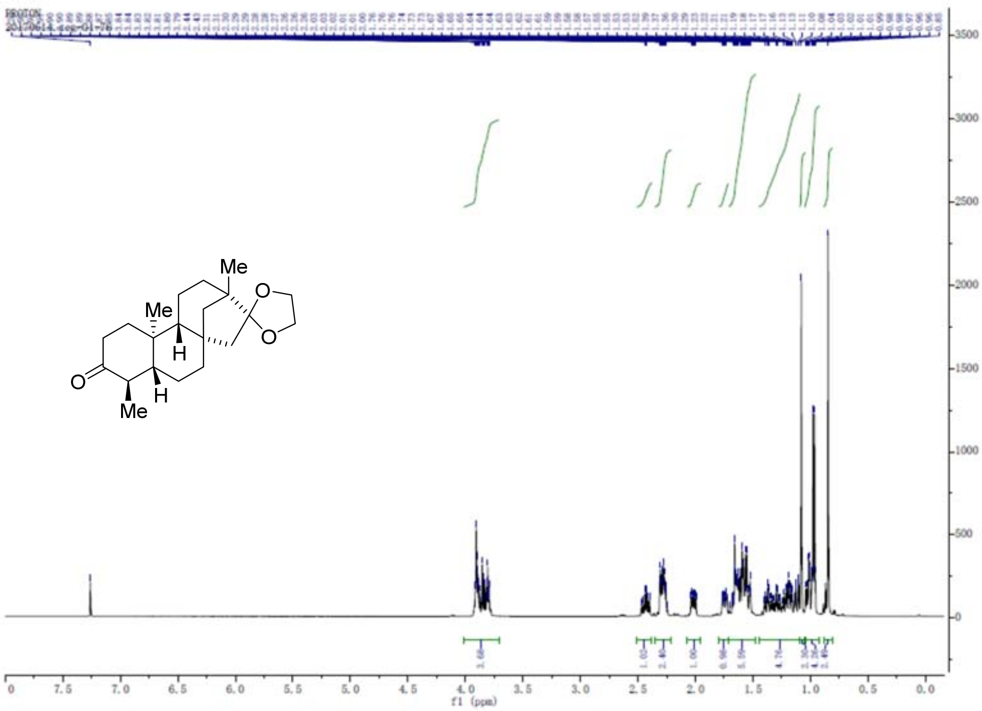

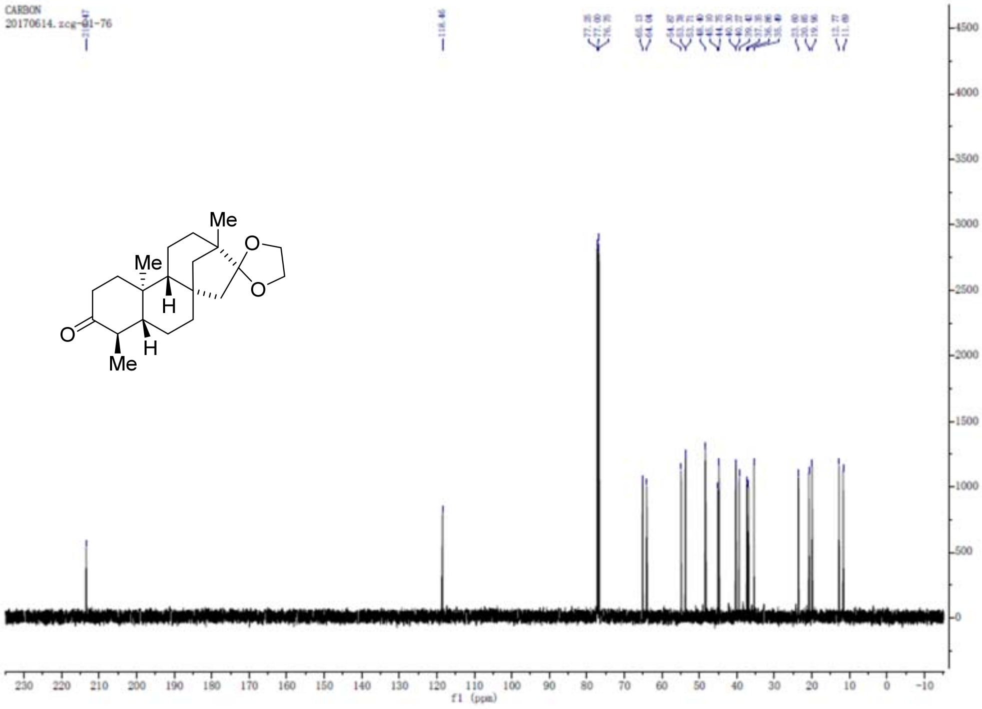

Supplementary Figure 72. ^{1}H and ^{13}C spectra of **S10**.

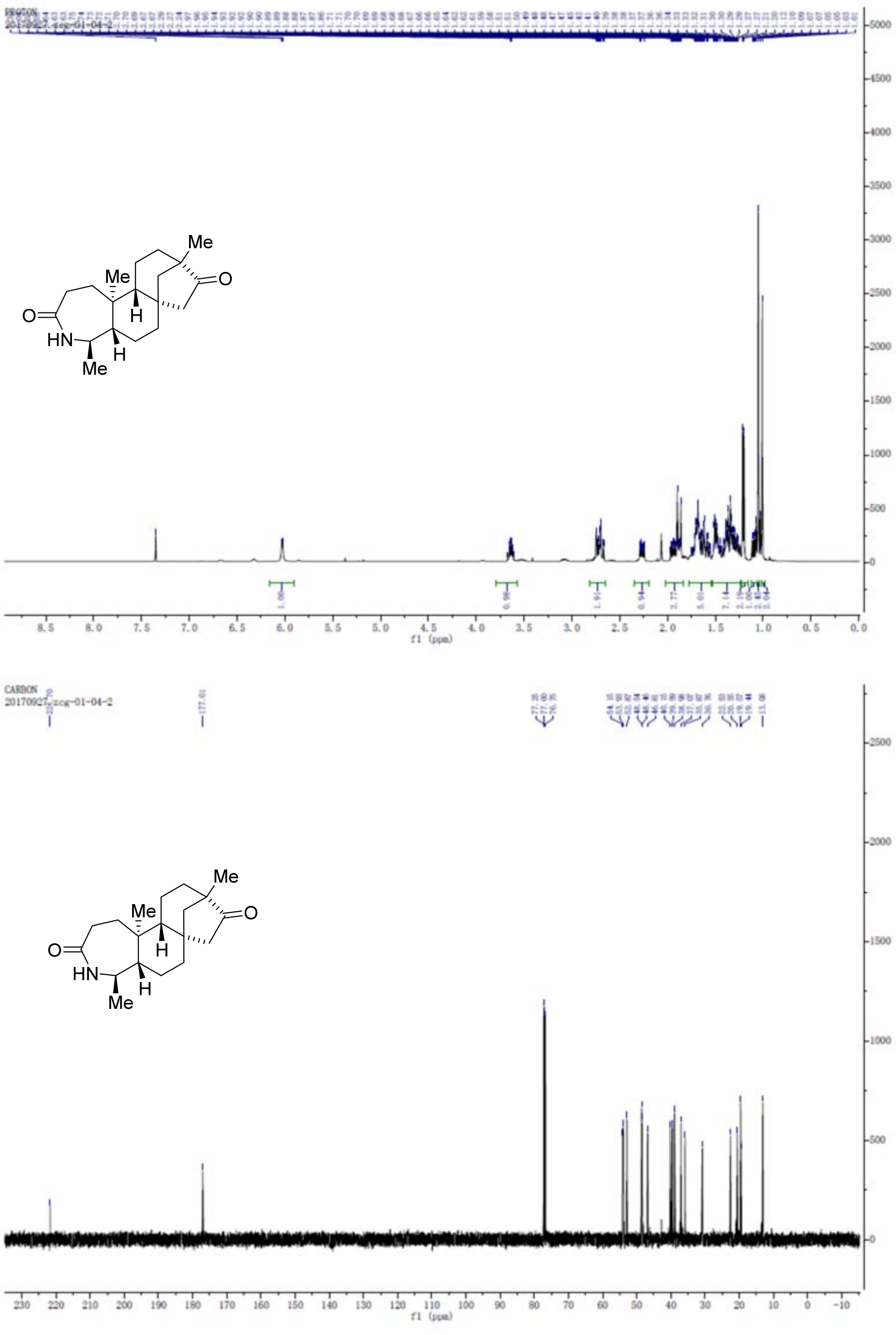

Supplementary Figure 73. ^{1}H and ^{13}C spectra of **24**.

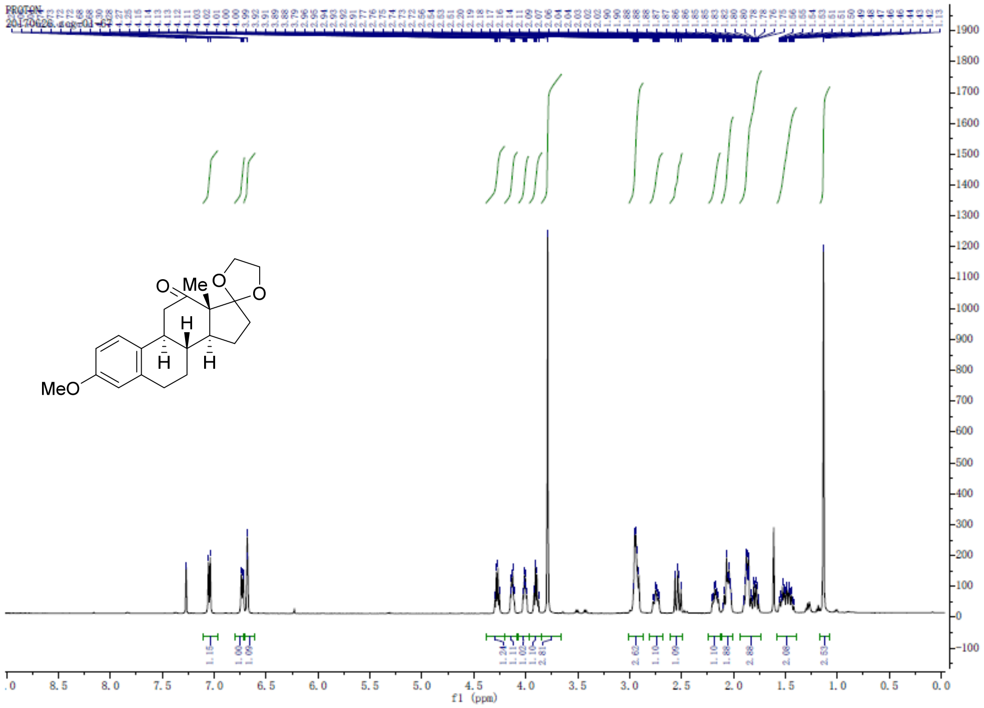

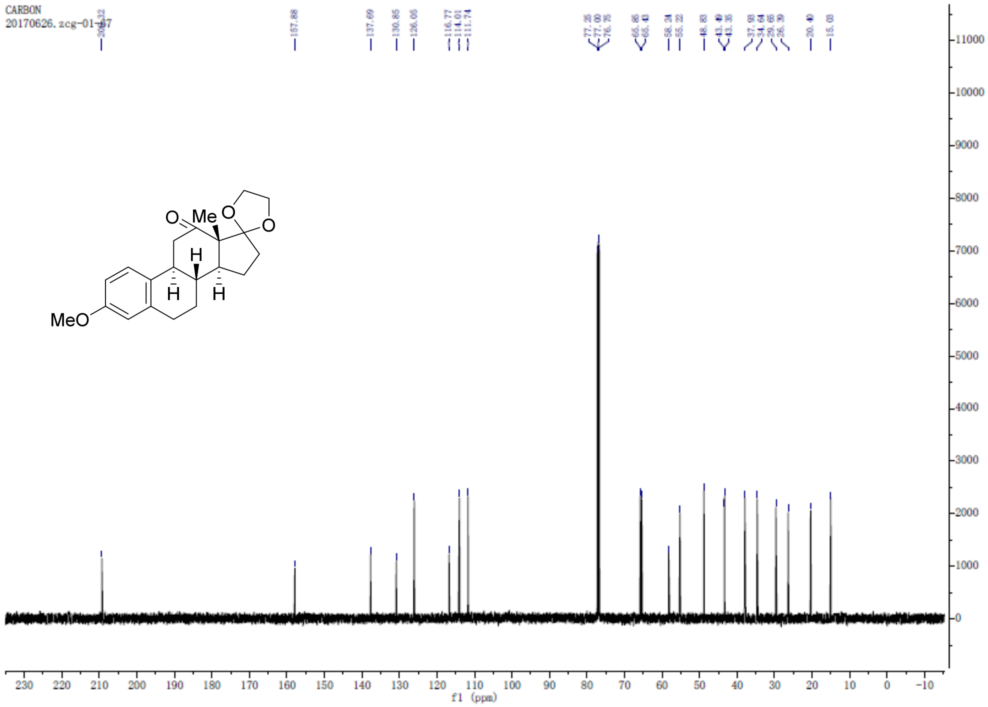

Supplementary Figure 74. ^{1}H and ^{13}C spectra of **S12a**.

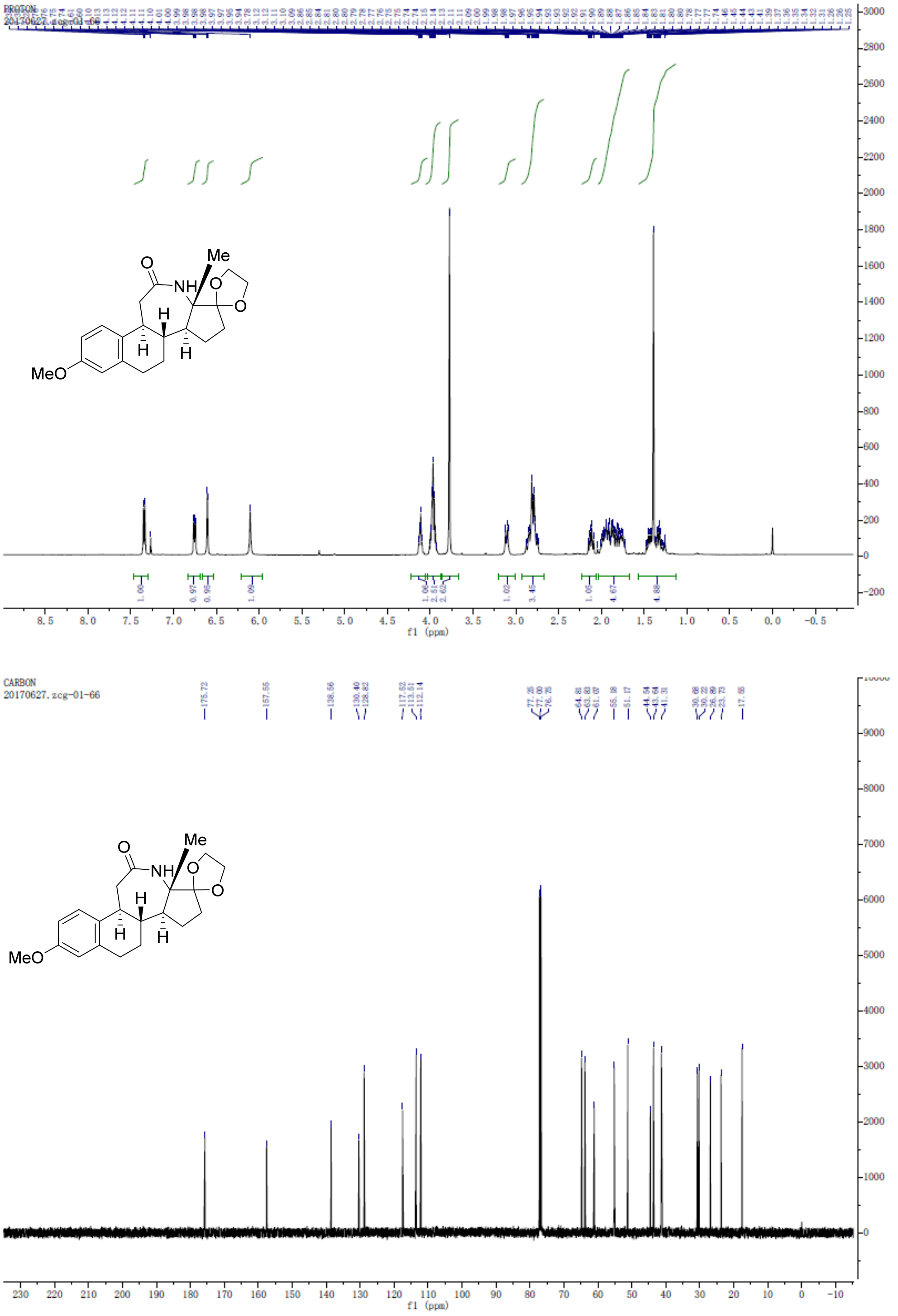

Supplementary Figure 75. ^{1}H and ^{13} C spectra of **27a**.

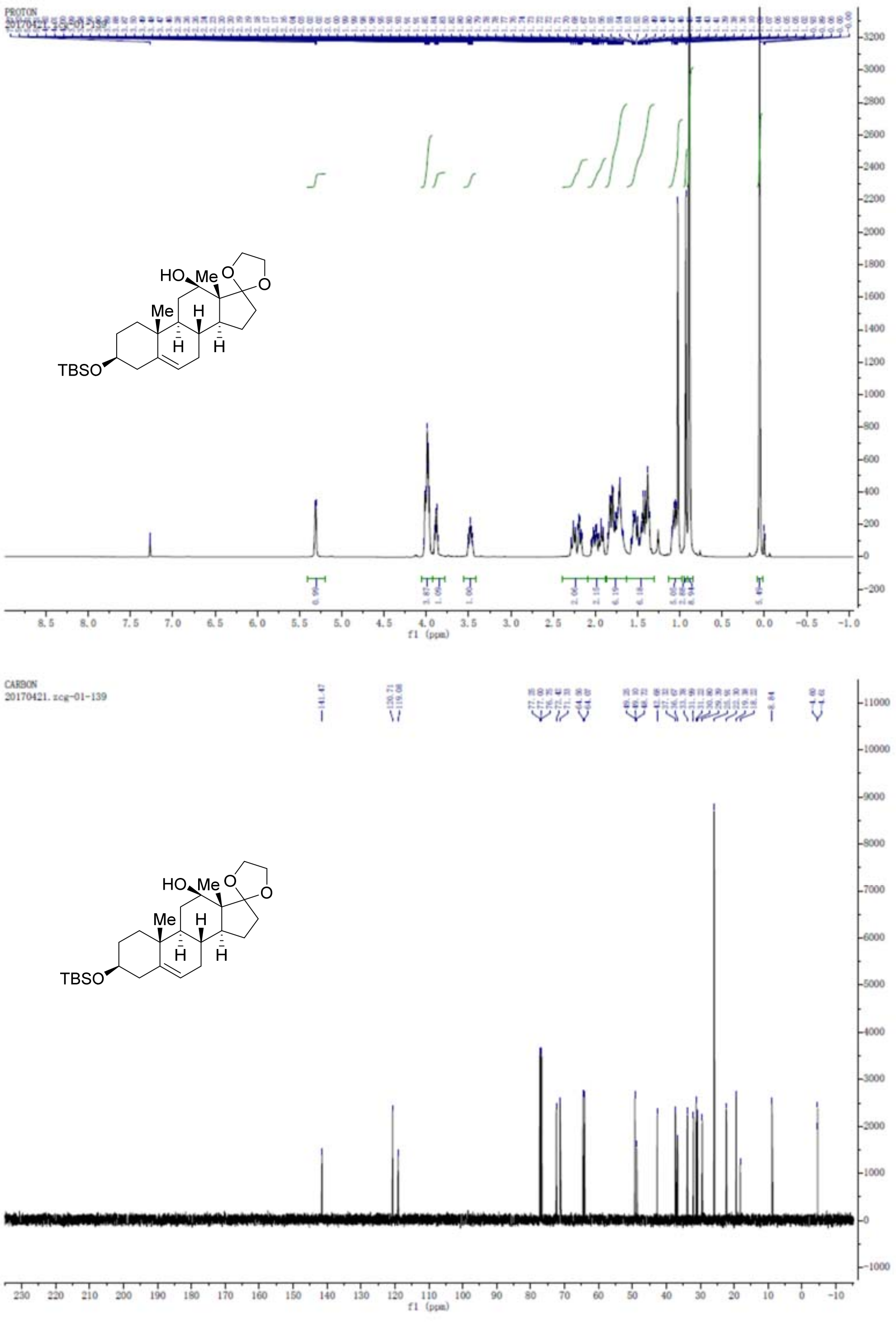

Supplementary Figure 76. ^{1}H and ^{13}C spectra of **S11**.

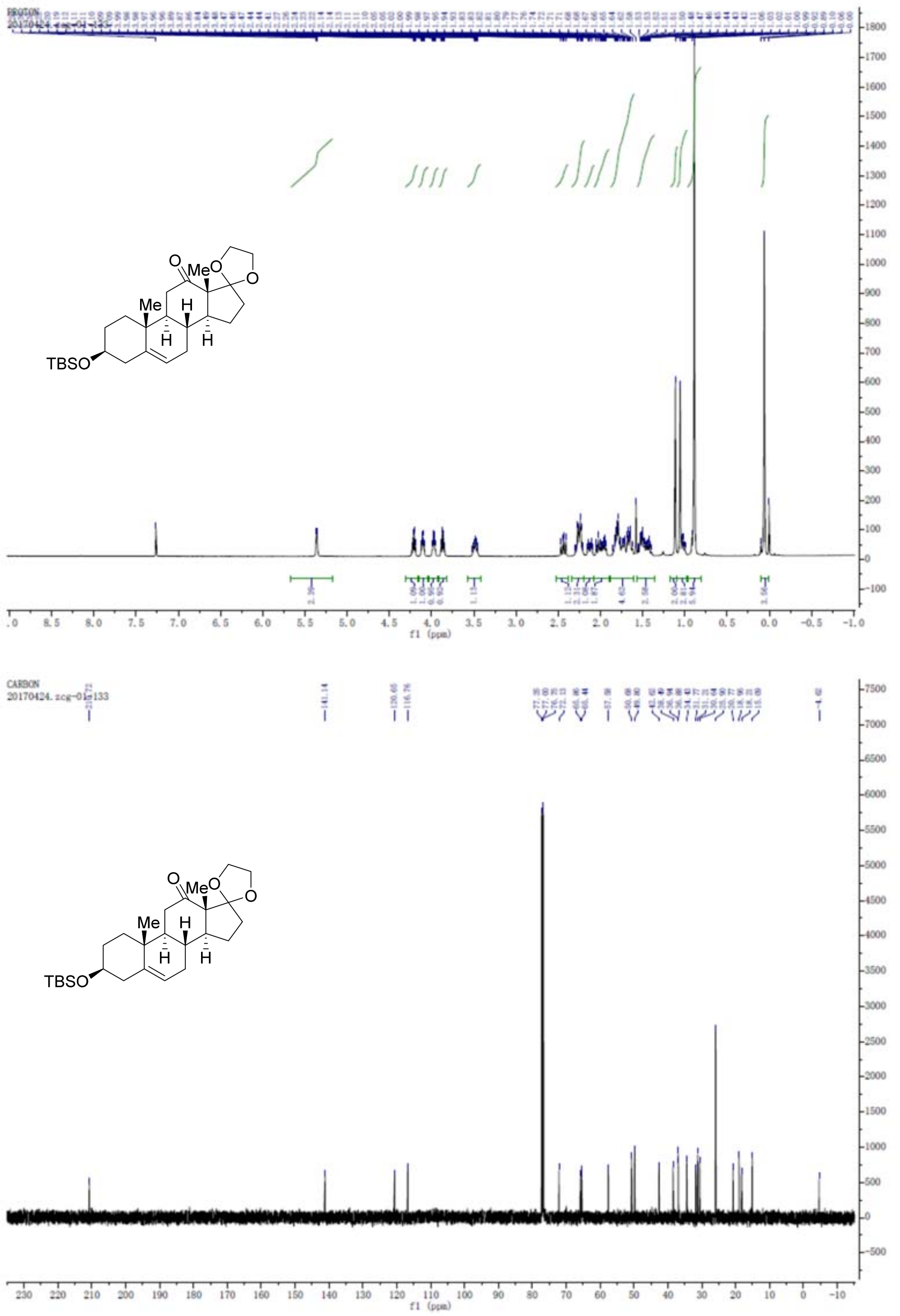

Supplementary Figure 77. ^{1}H and ^{13}C spectra of **S12b**.

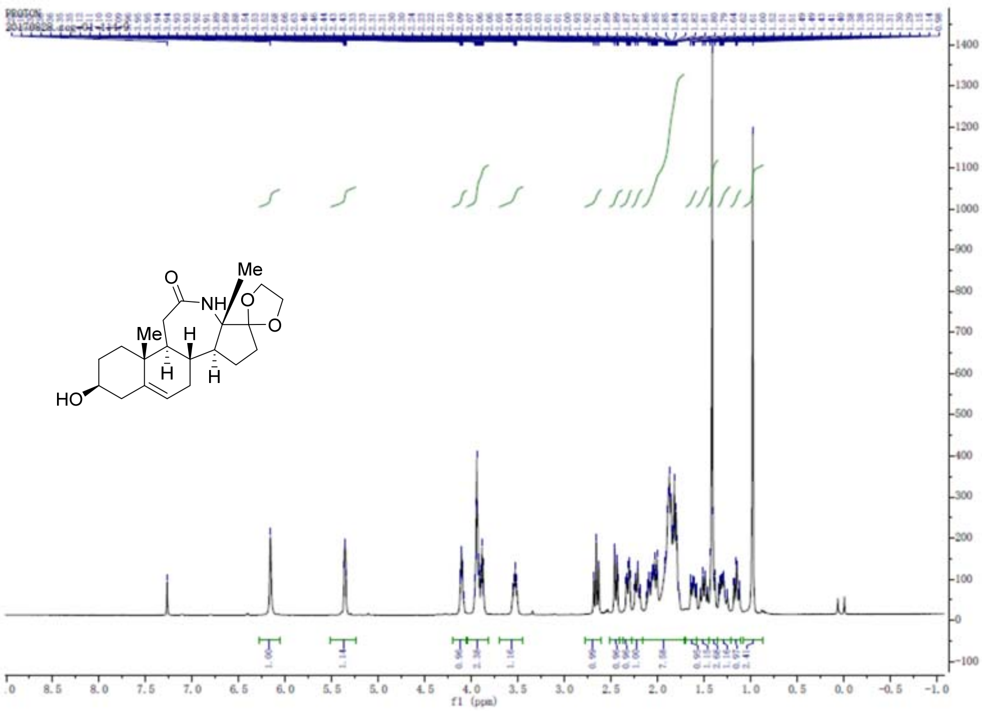

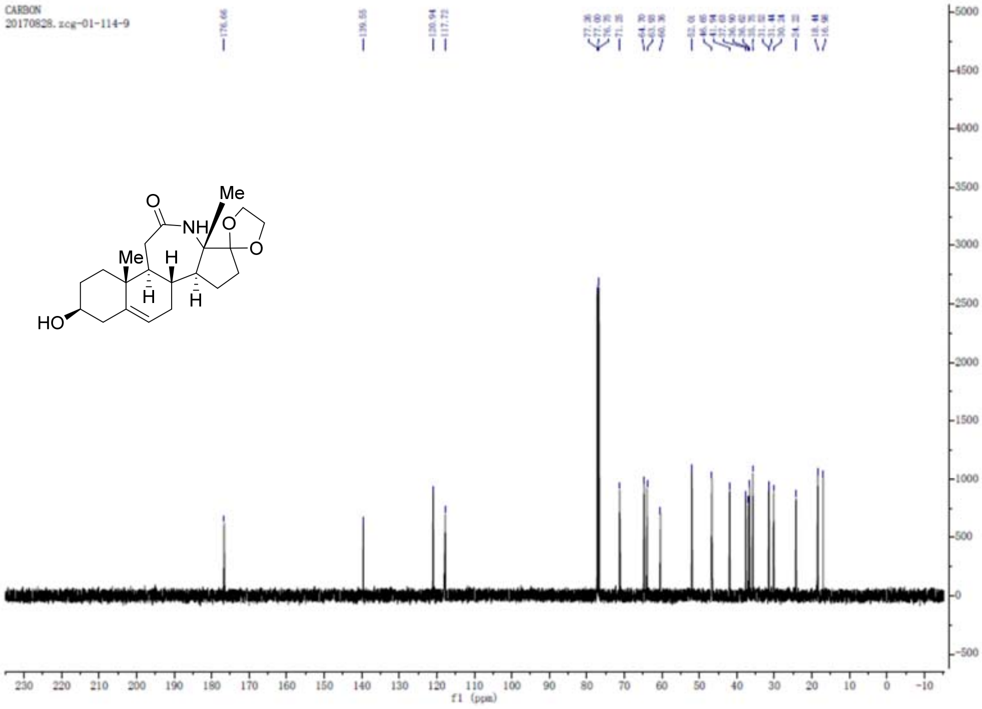

Supplementary Figure 78. ^{1}H and ^{13}C spectra of **27b**.

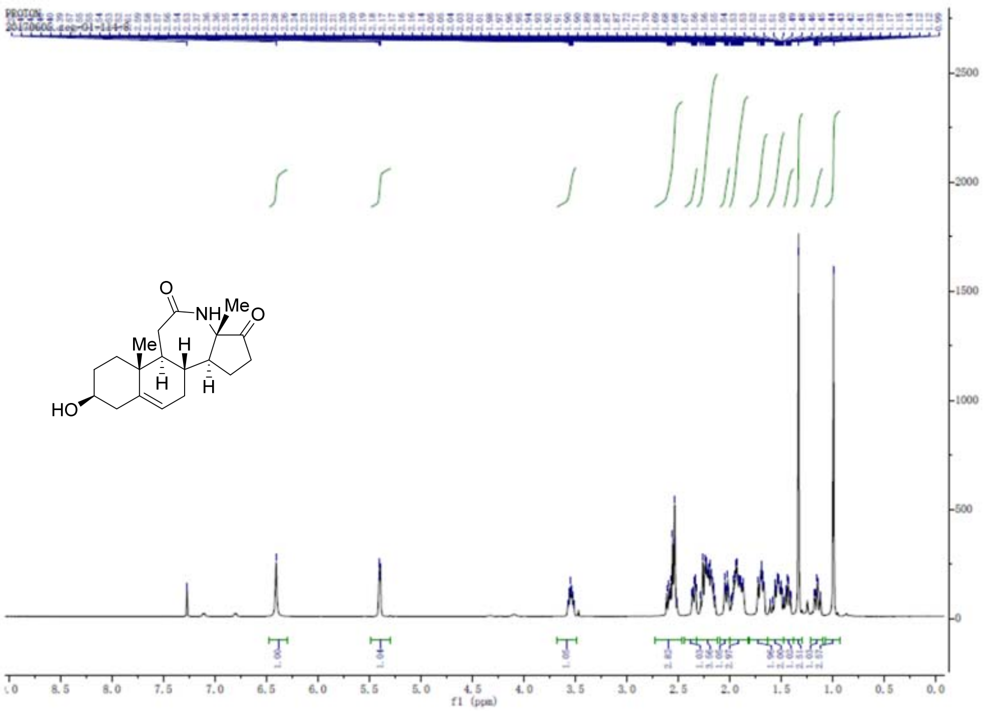

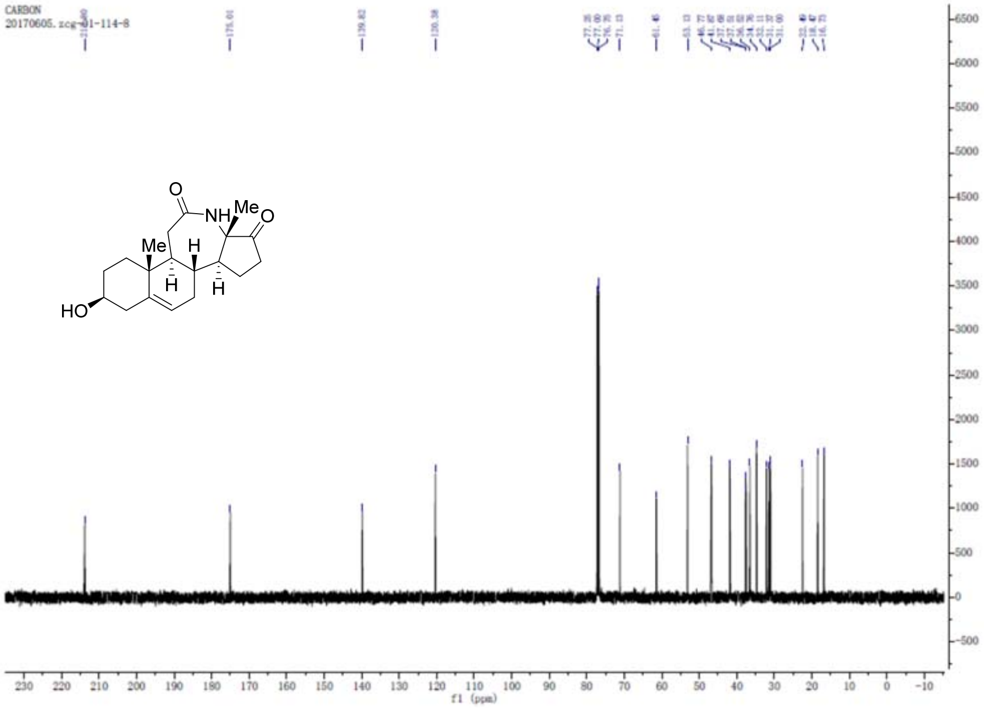

Supplementary Figure 79. ^{1}H and ^{13}C spectra of **27c**.

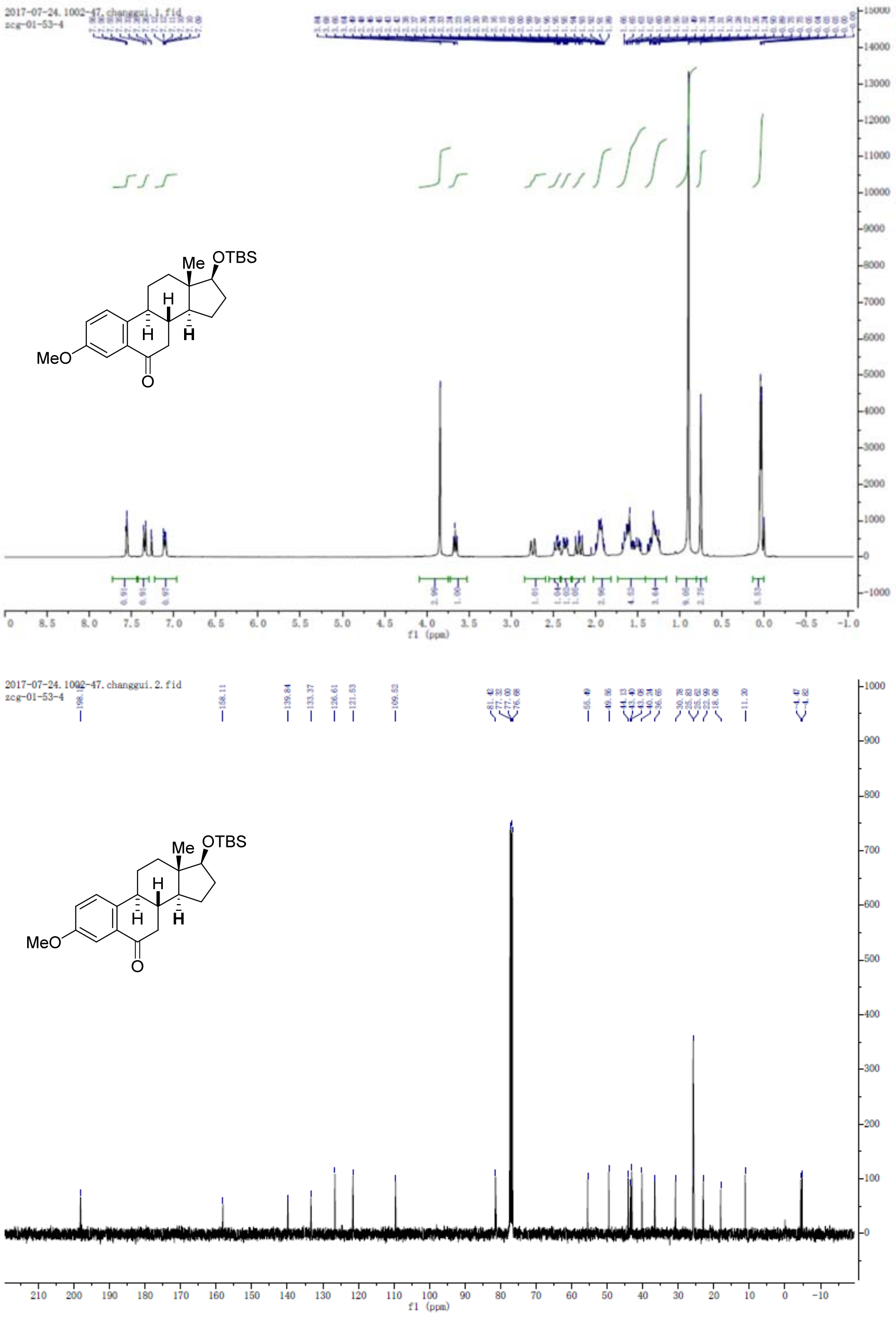

Supplementary Figure 80. ^{1}H and ^{13}C spectra of **28**.

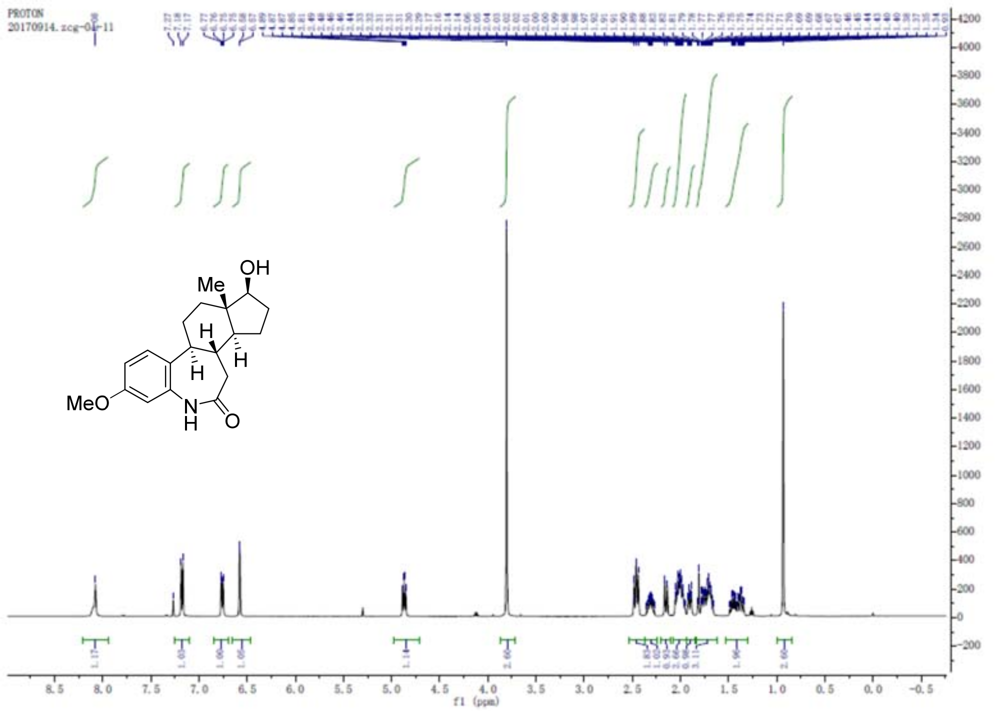

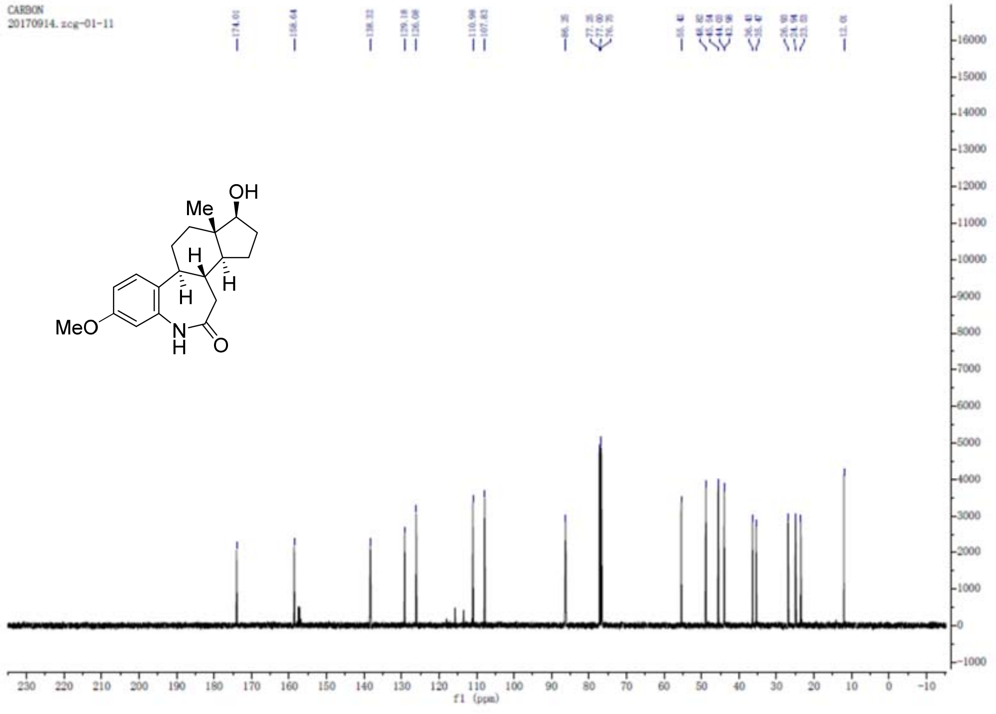

Supplementary Figure 81. ^{1}H and ^{13}C spectra of **29**.

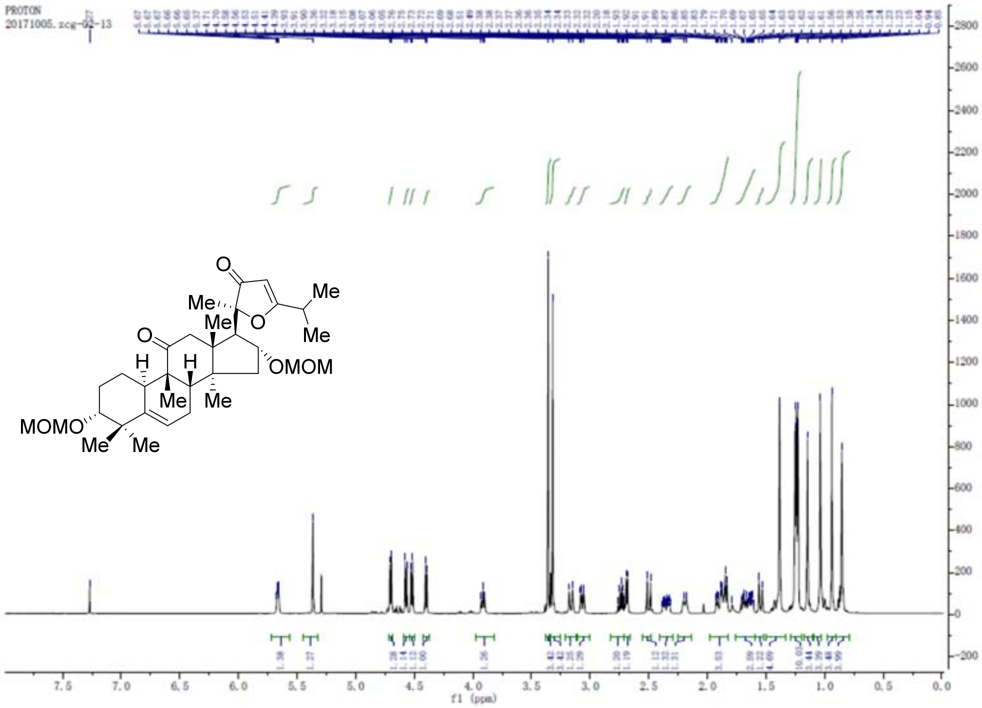

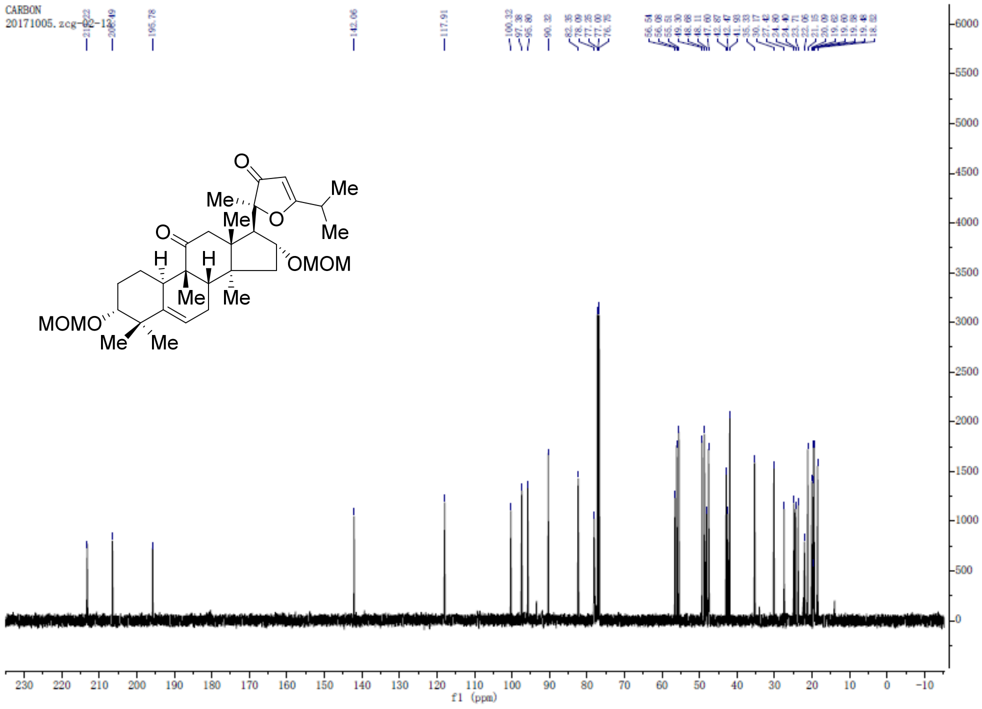

Supplementary Figure 82. ^{1}H and ^{13}C spectra of **31**.

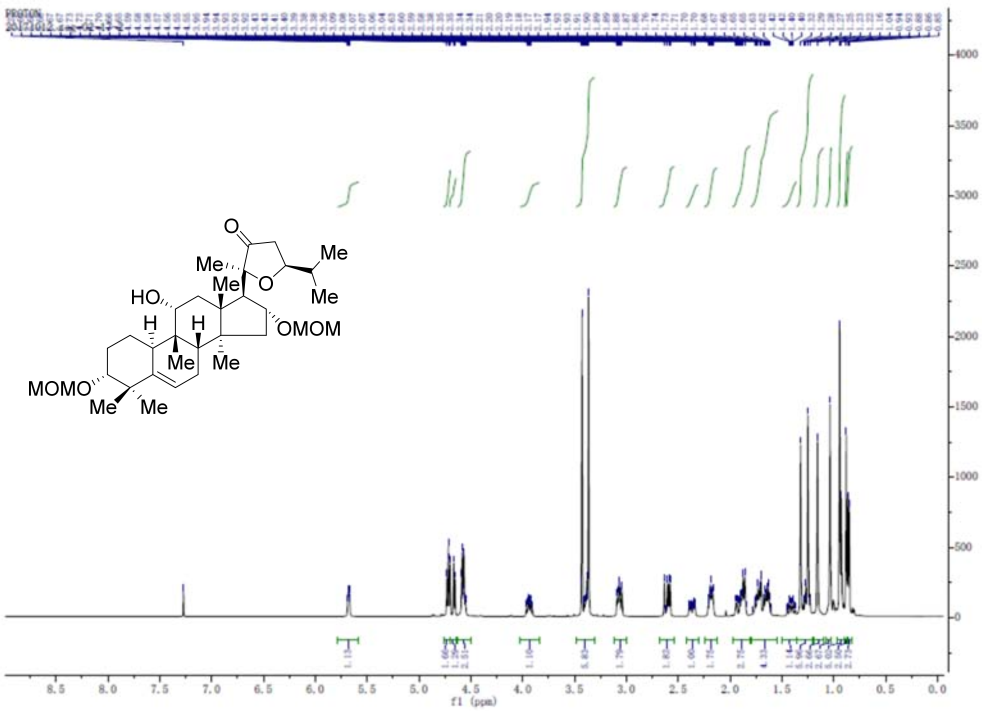

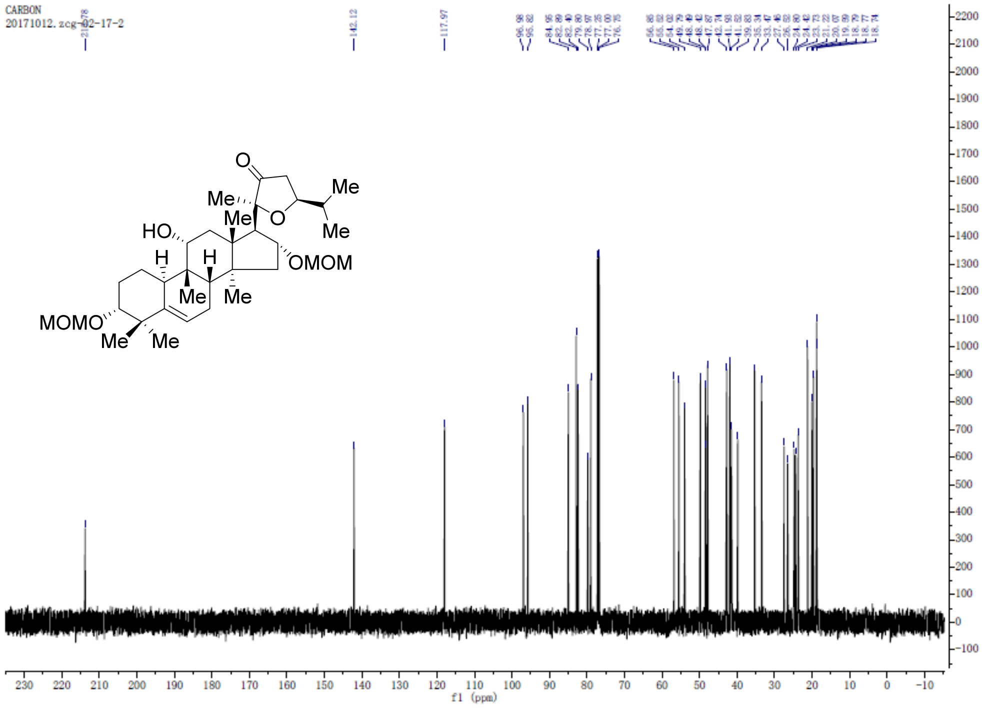

Supplementary Figure 83. ^{1}H and ^{13}C spectra of **32**.

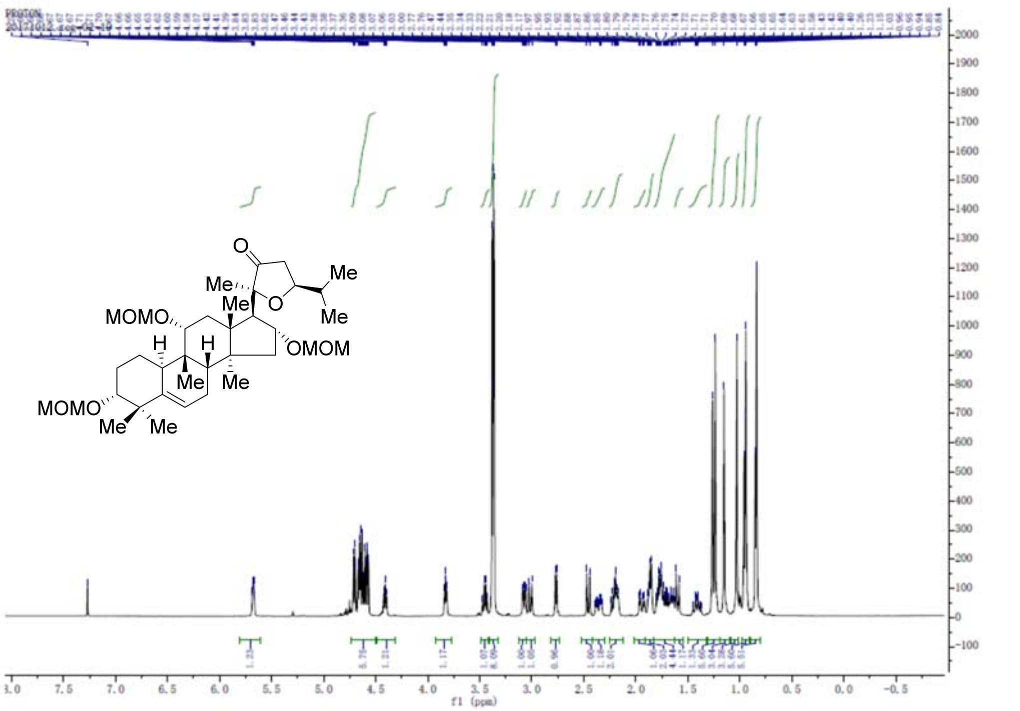

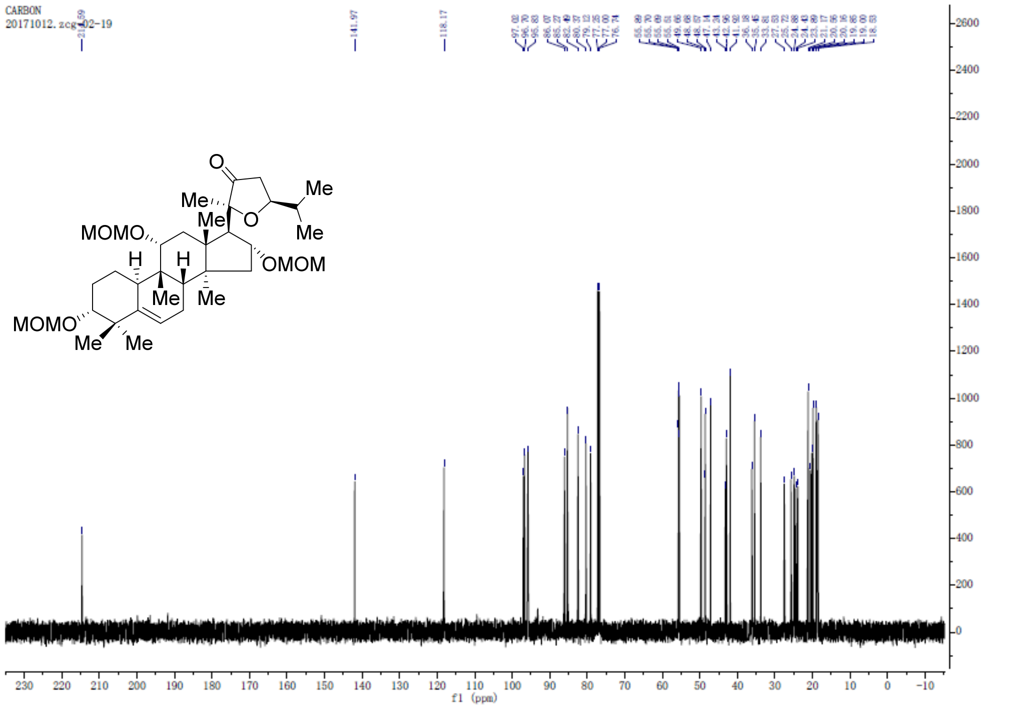

Supplementary Figure 84. ^{1}H and ^{13}C spectra of **S13**.

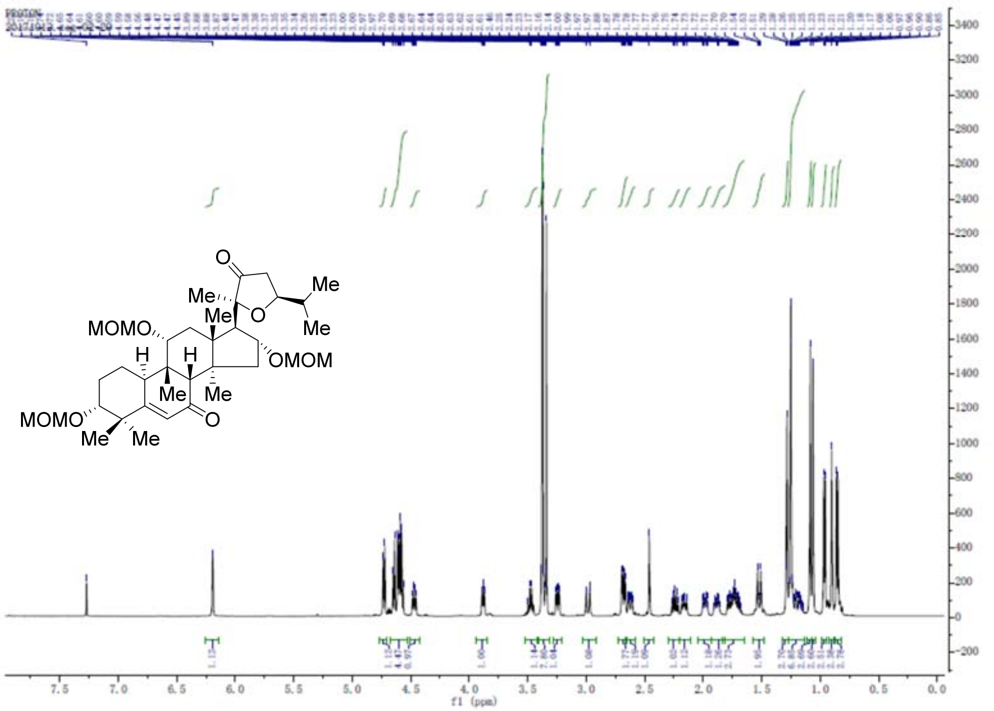

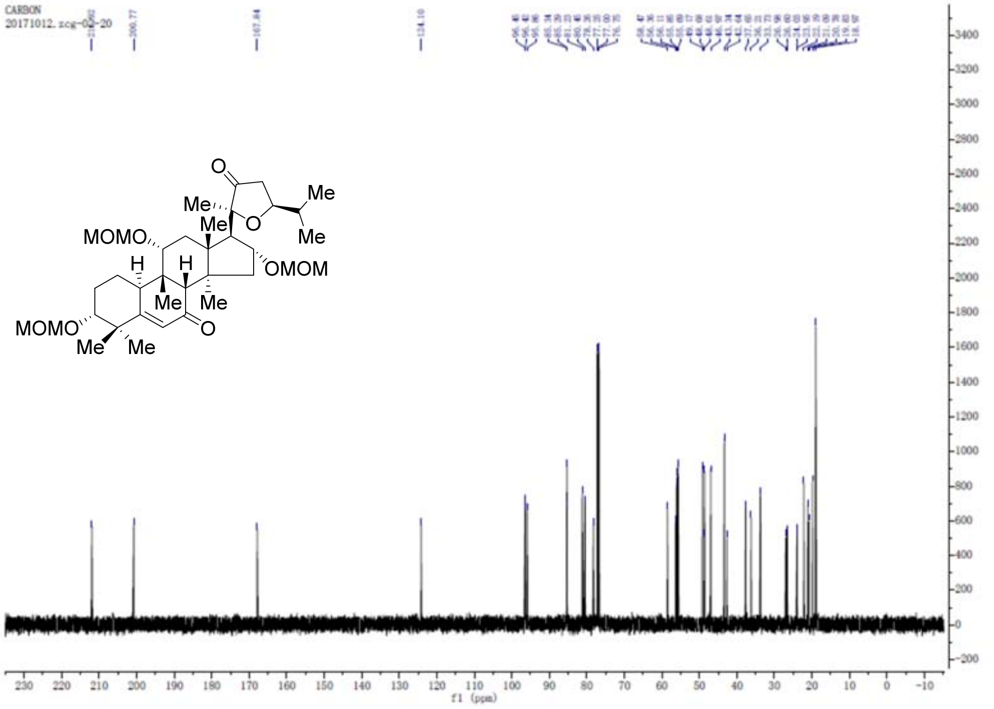

Supplementary Figure 85. ^{1}H and ^{13}C spectra of **33**.

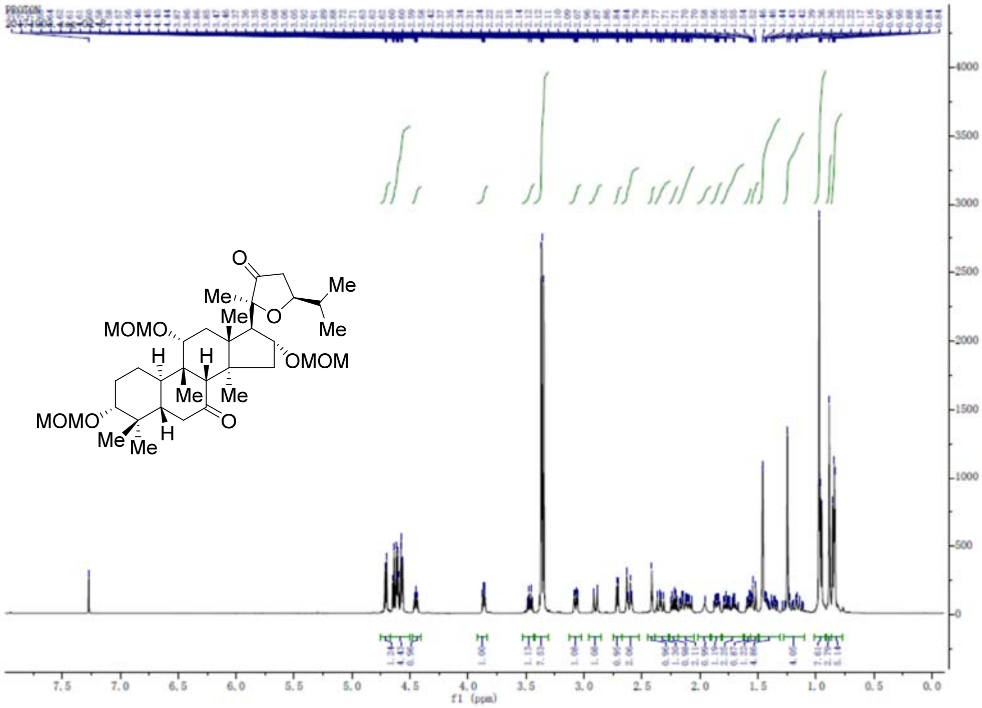

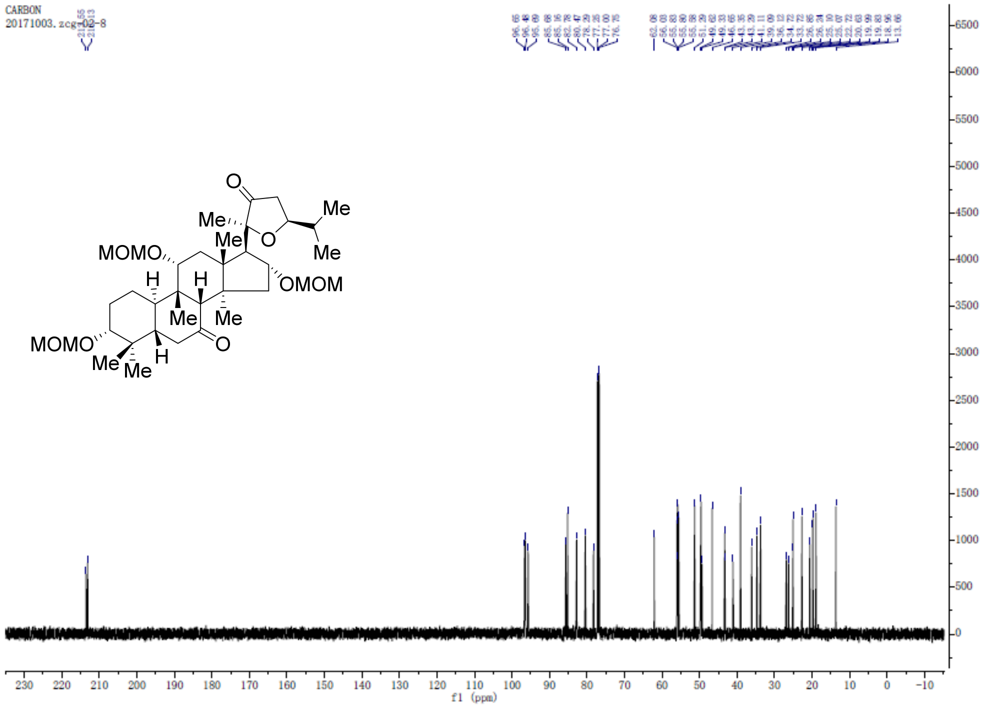

Supplementary Figure 86. ^{1}H and ^{13}C spectra of **34**.

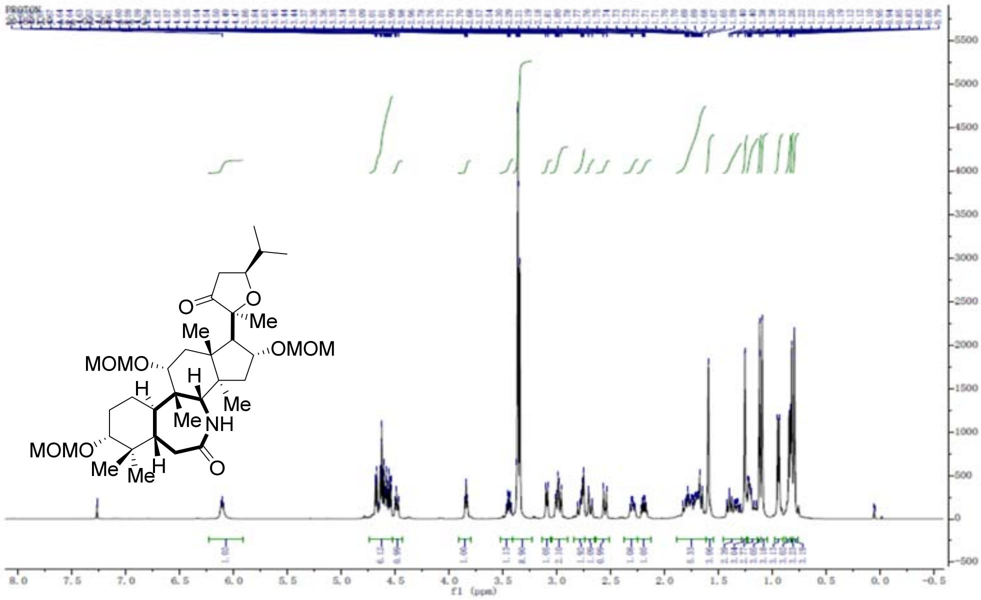

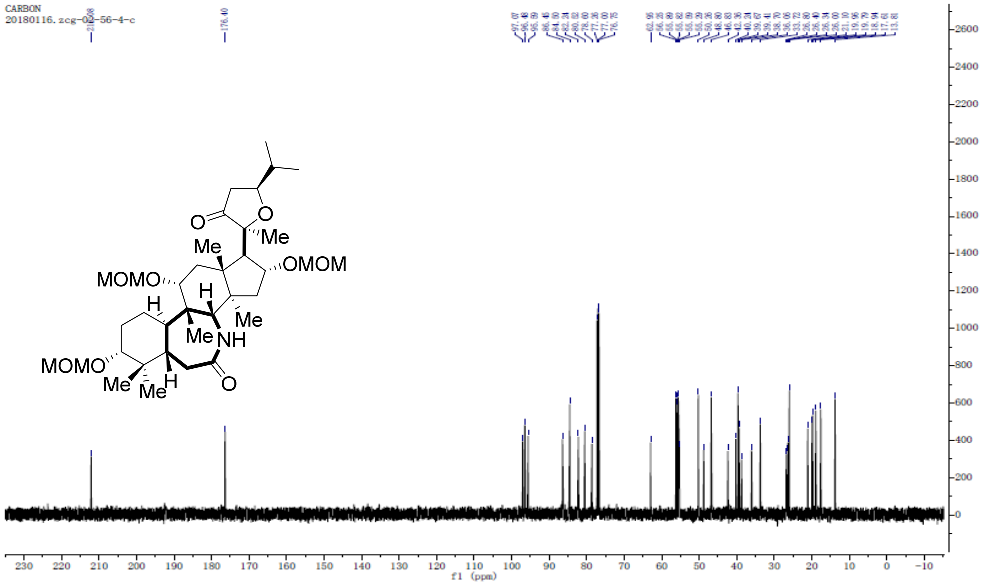

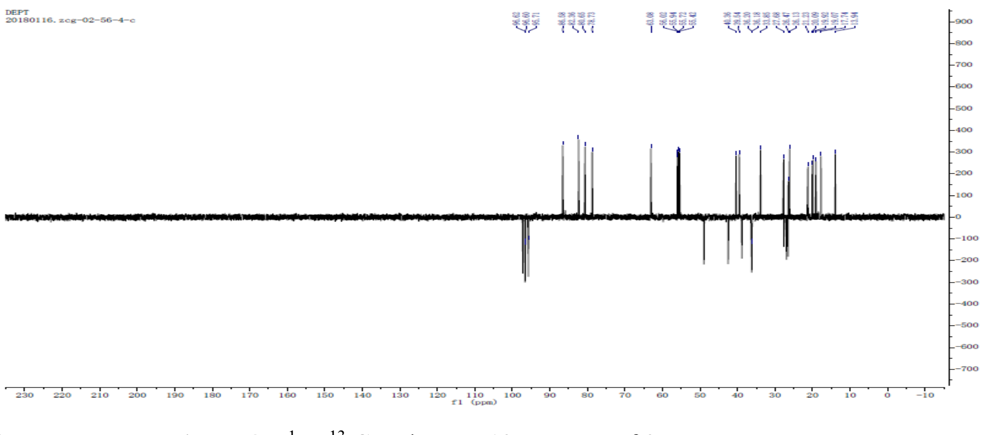

Supplementary Figure 87. ^{1}H, ^{13} C and DPET135 spectra of **35**.

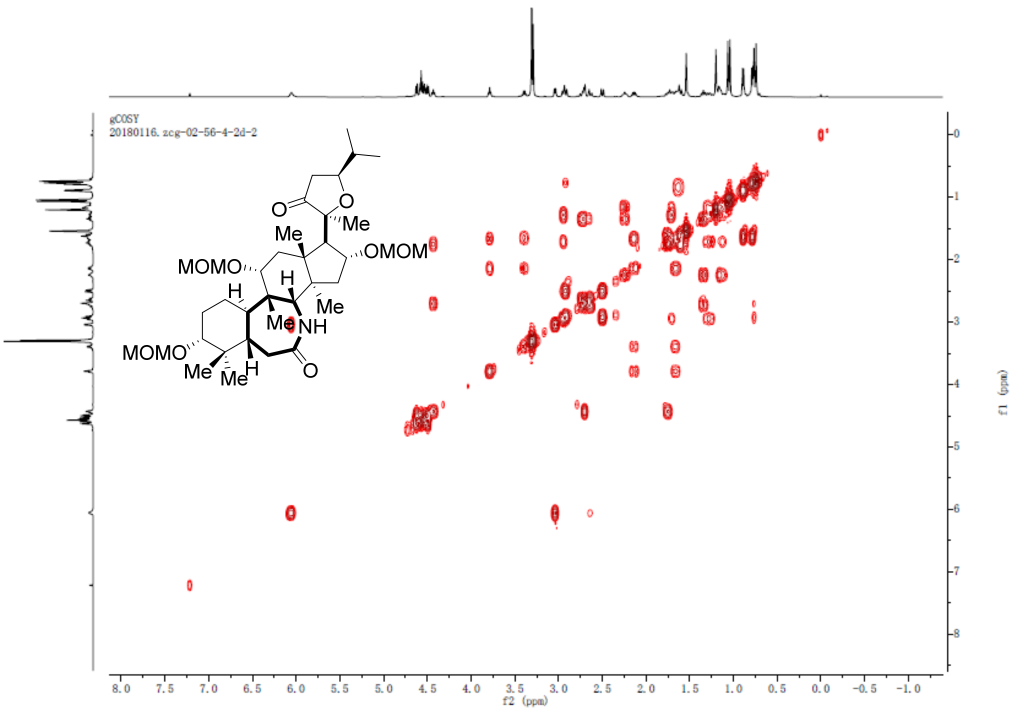

Supplementary Figure 88. ^{1}H-^{1}H COSY spectra of **35**.

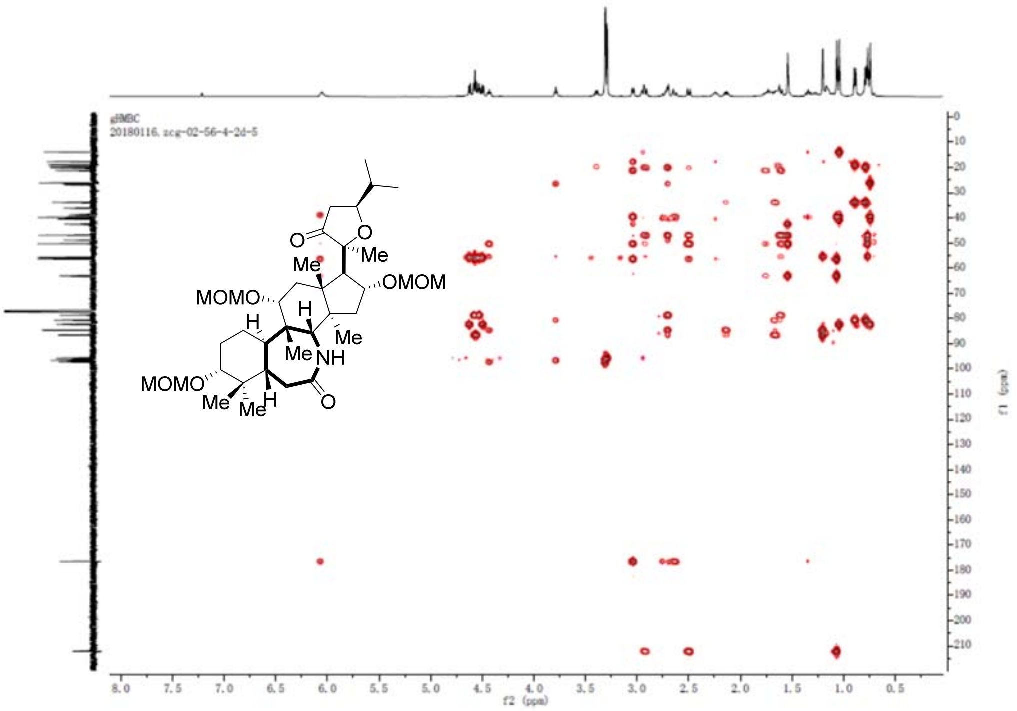

Supplementary Figure 89. HMBC spectra of **35**.

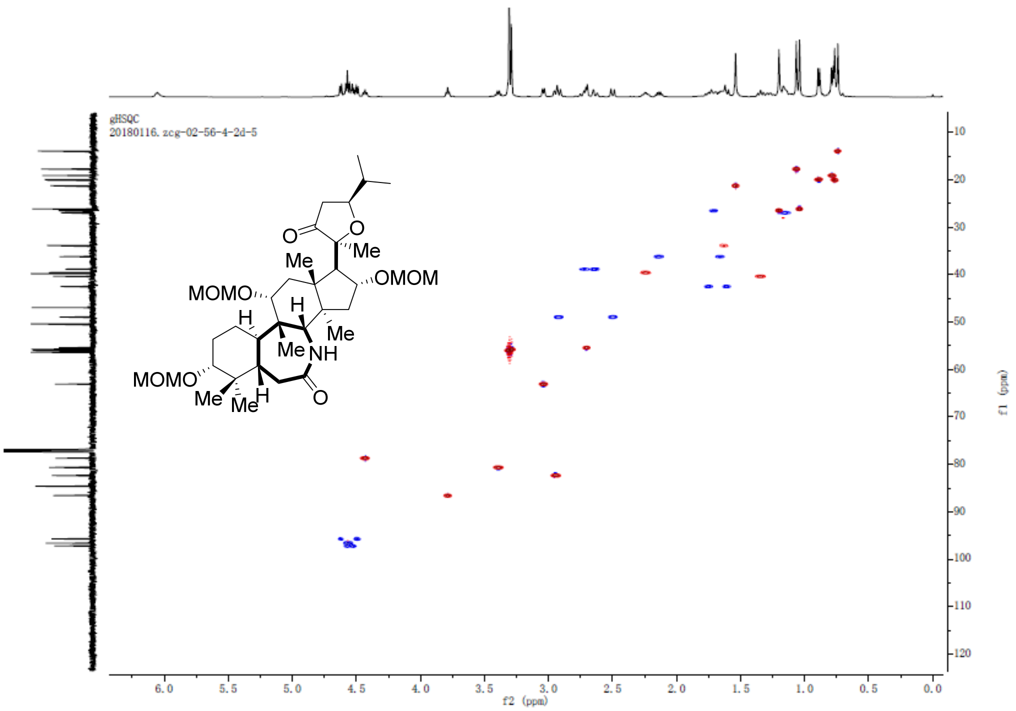

Supplementary Figure 90. HSQC of **35.**

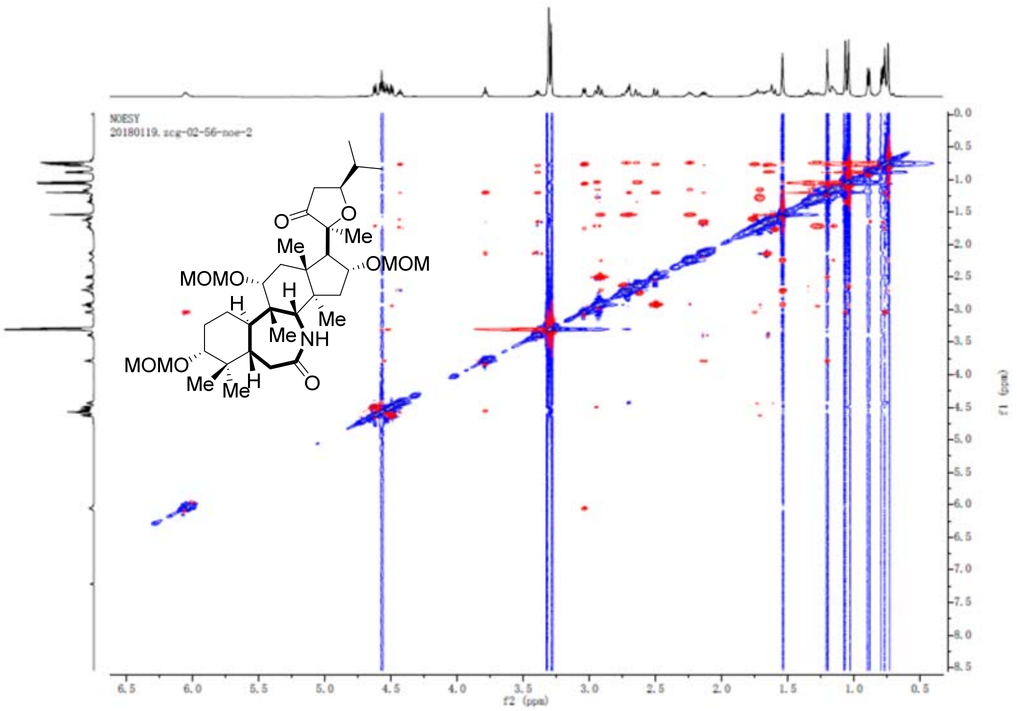

Supplementary Figure 91. NOESY spectra of **35**.

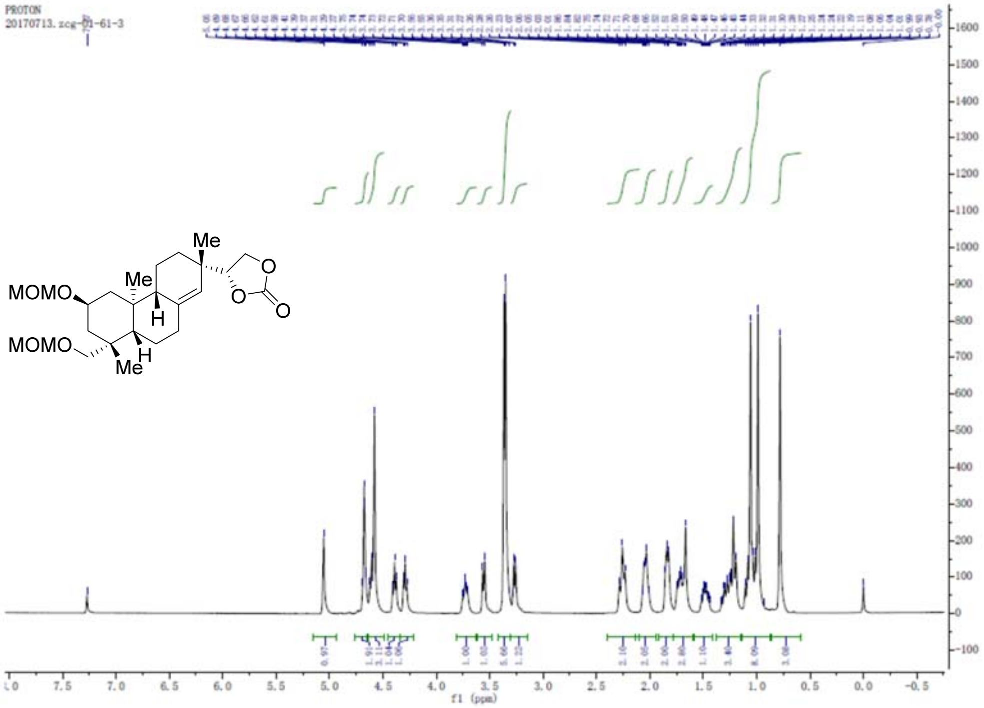

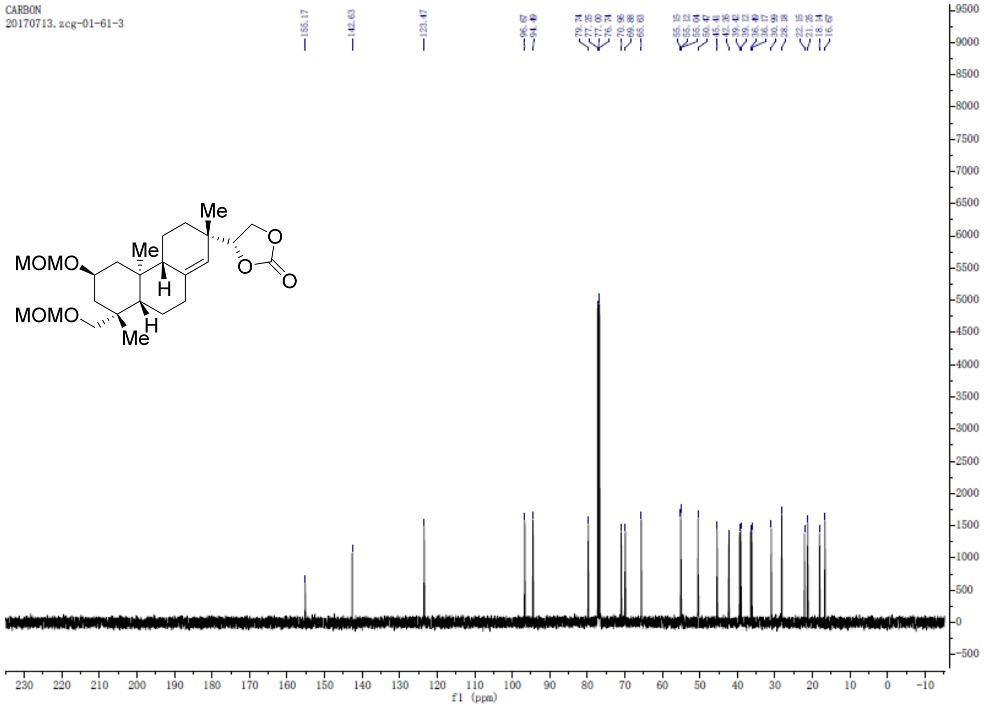

Supplementary Figure 92. ^{1}H and ^{13} C spectra of **37**.

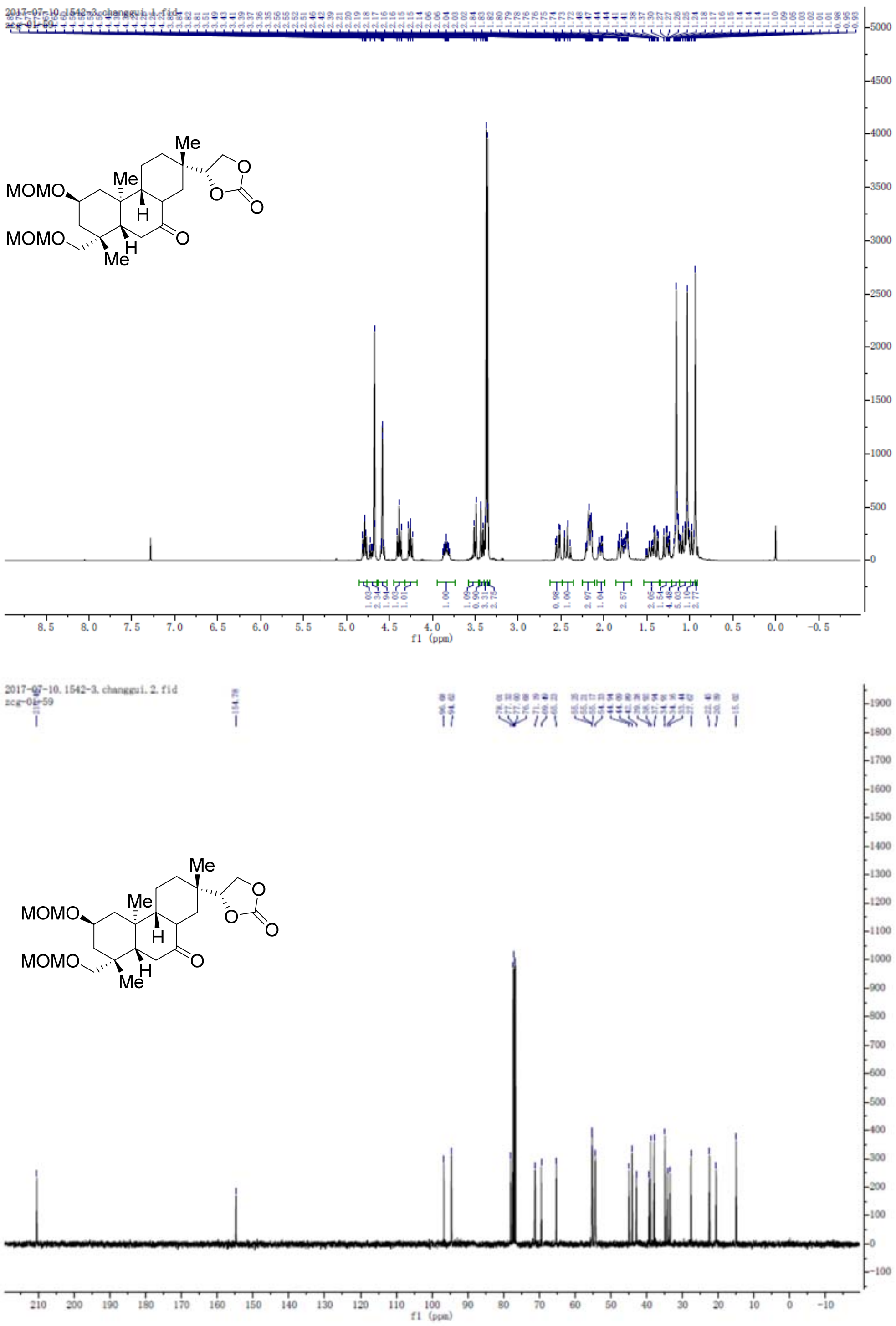

Supplementary Figure 93. ^{1}H and ^{13} C spectra of **38**.

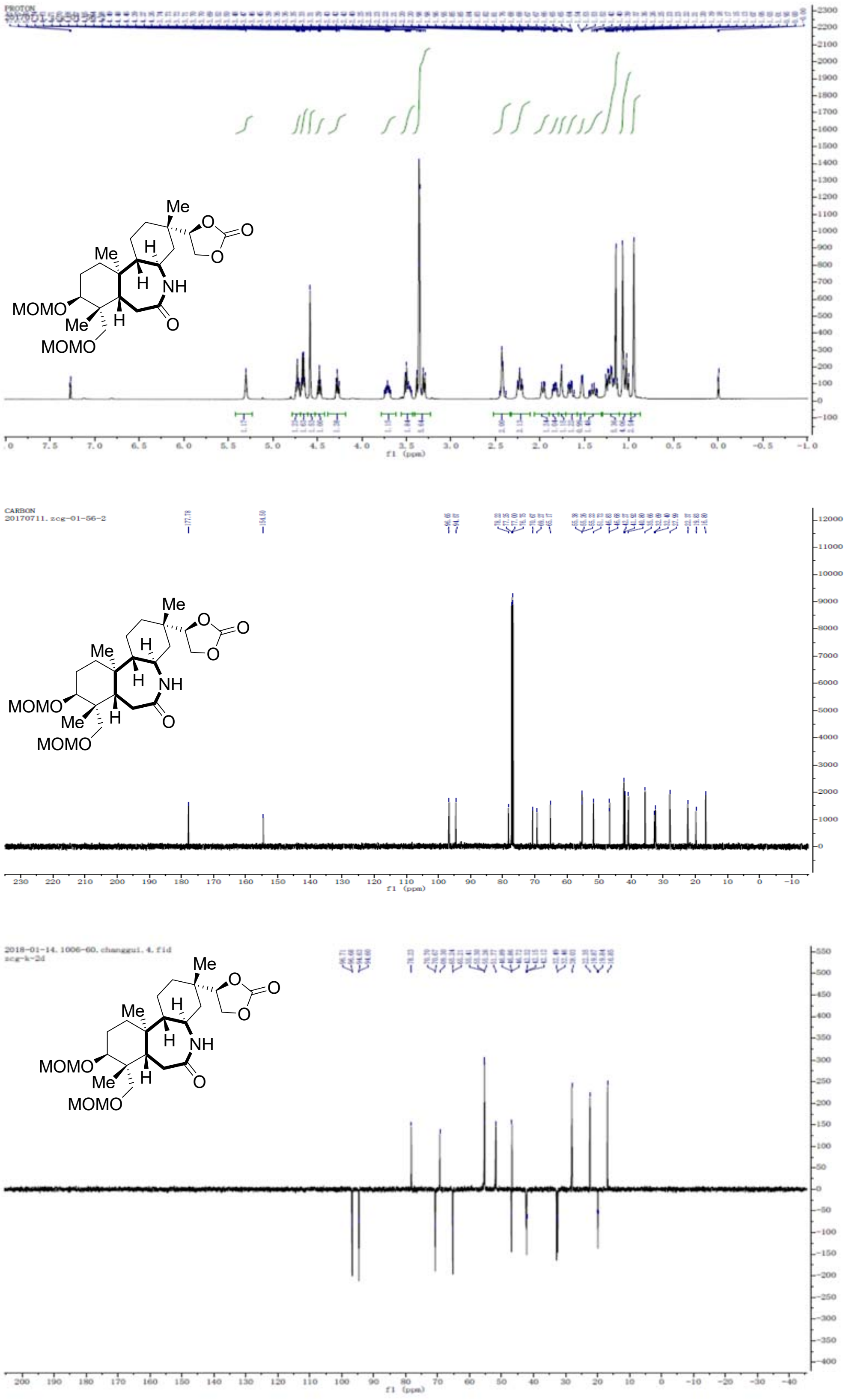

Supplementary Figure 94. ^{1}H, ^{13}C and DEPT135 spectra of **39**.

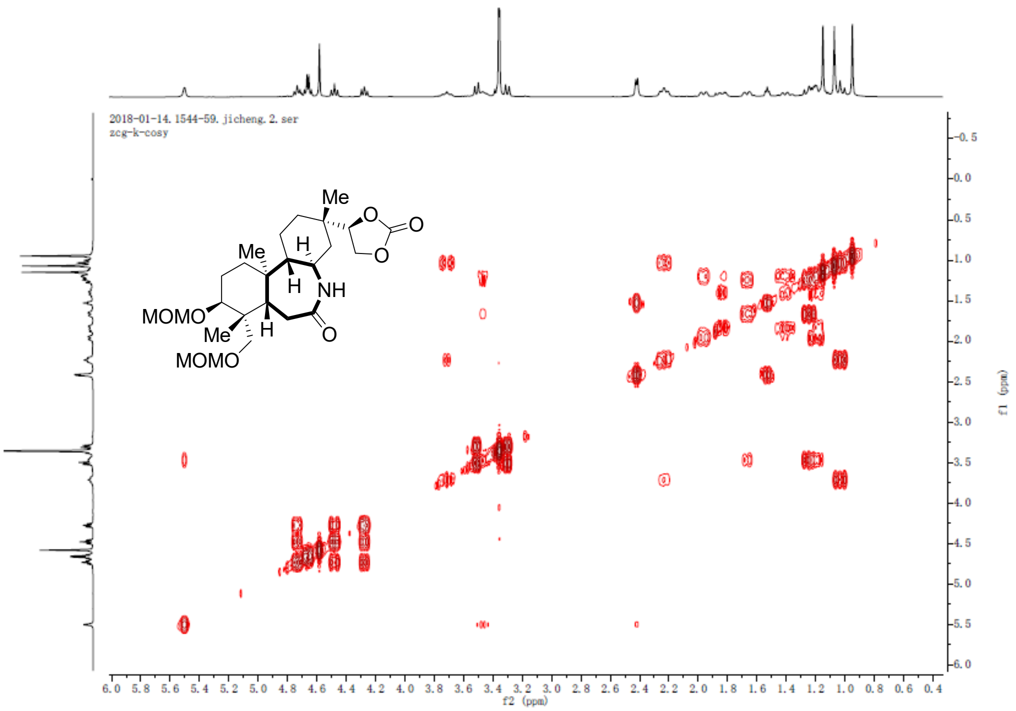

Supplementary Figure 95. H-H Cosy spectra of **39**.

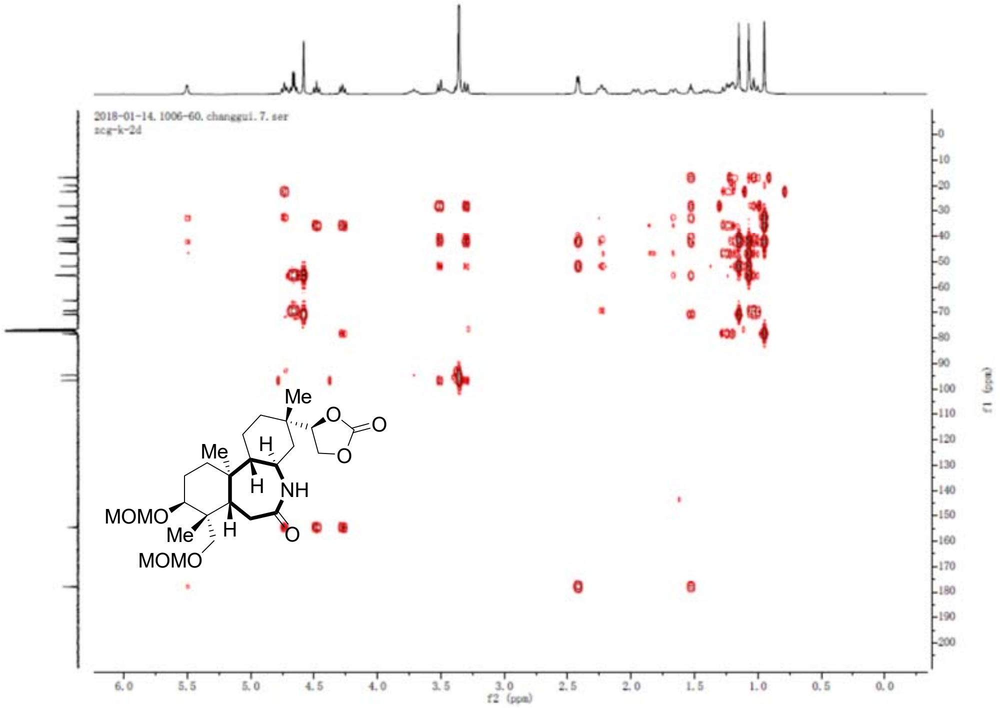

Supplementary Figure 96. HMBC spectra of **39**.

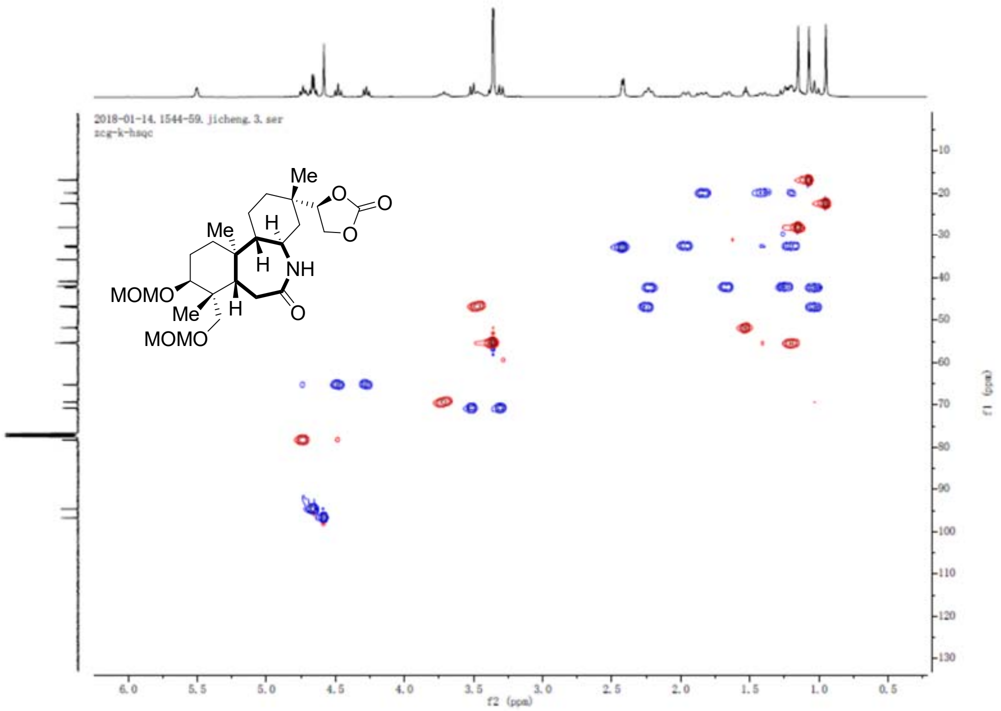

Supplementary Figure 97. HSQC spectra of **39**.

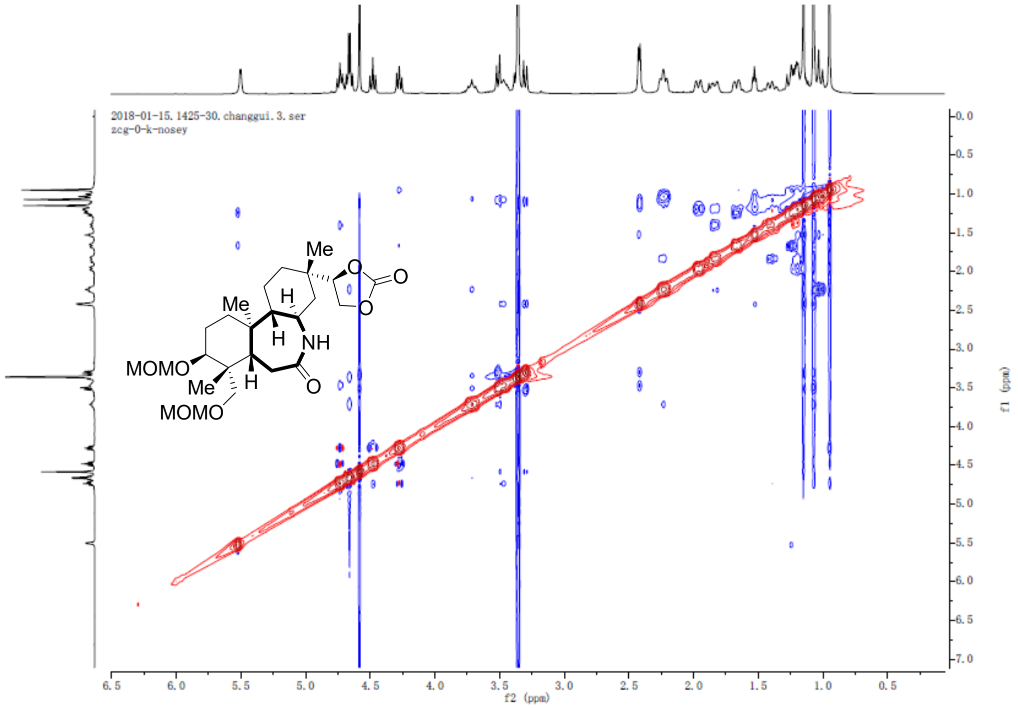

Supplementary Figure 98. NOESY spectra of **39**.

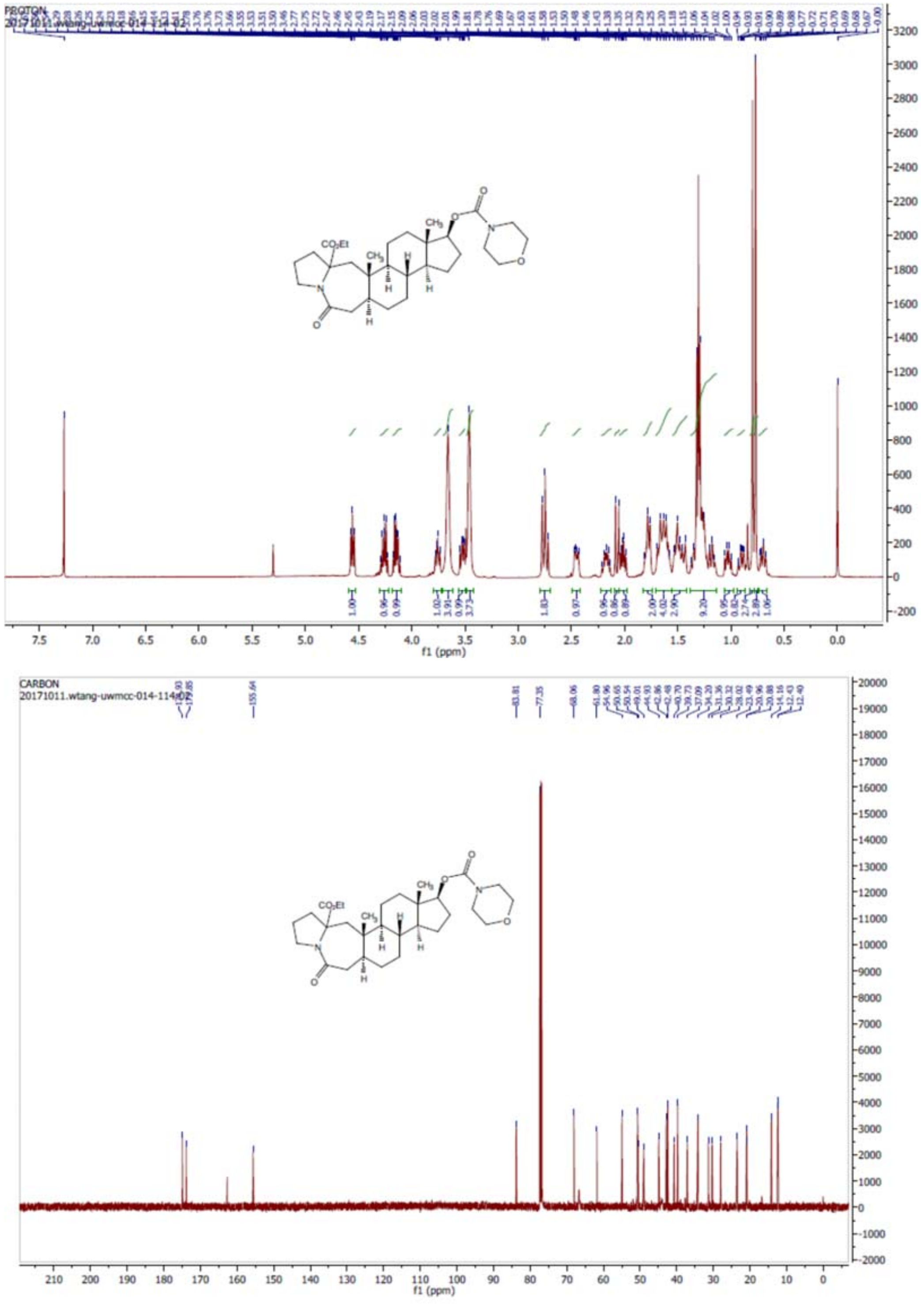

Supplementary Figure 99. ^{1}H and ^{13}C spectra of **40a**.

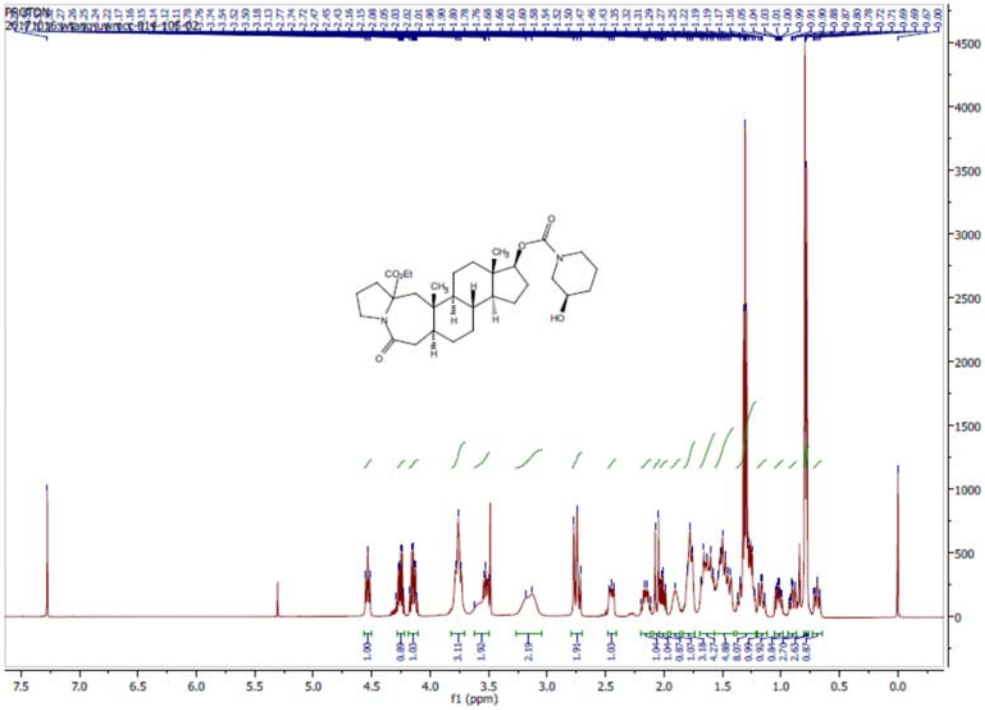

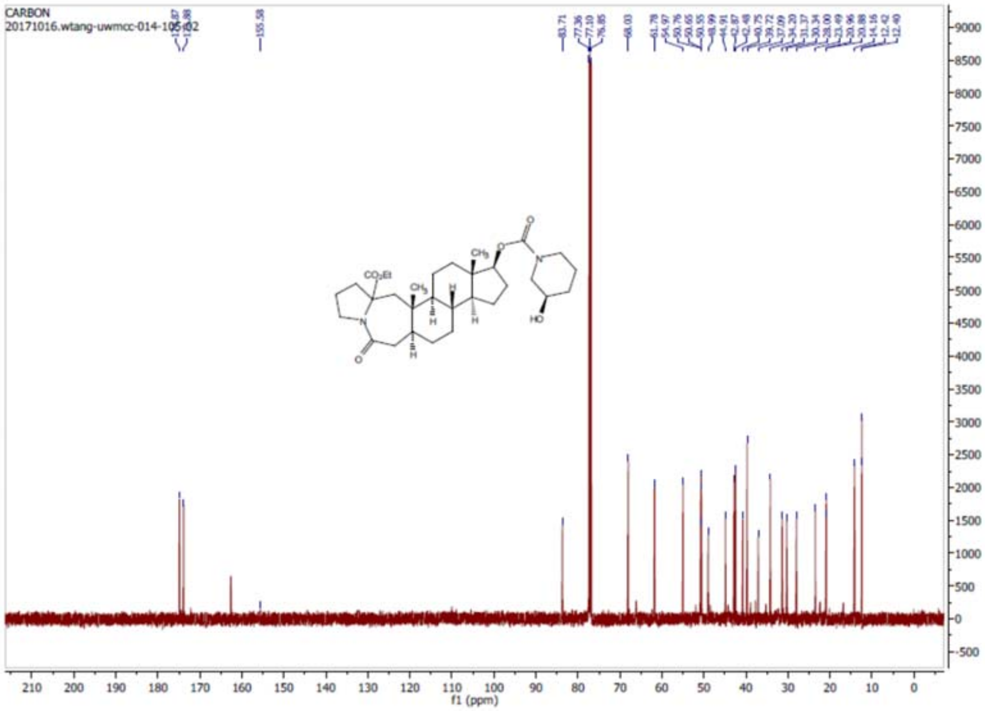

Supplementary Figure 100. ^{1}H and ^{13} C spectra of **40b**.

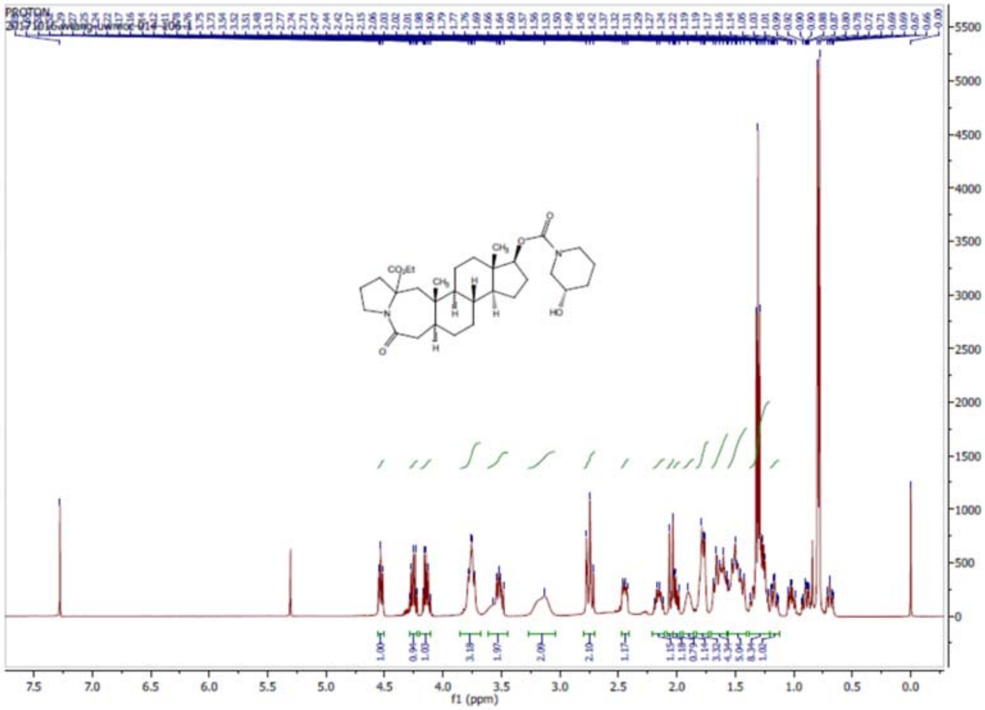

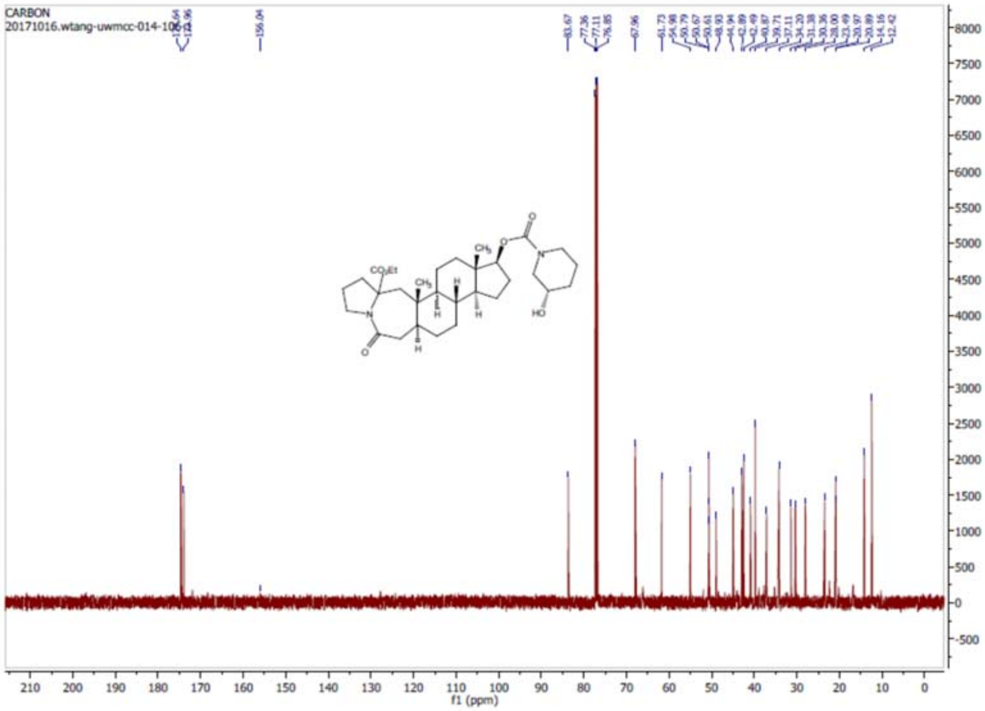

Supplementary Figure 101. ^{1}H and ^{13}C spectra of **40c**.

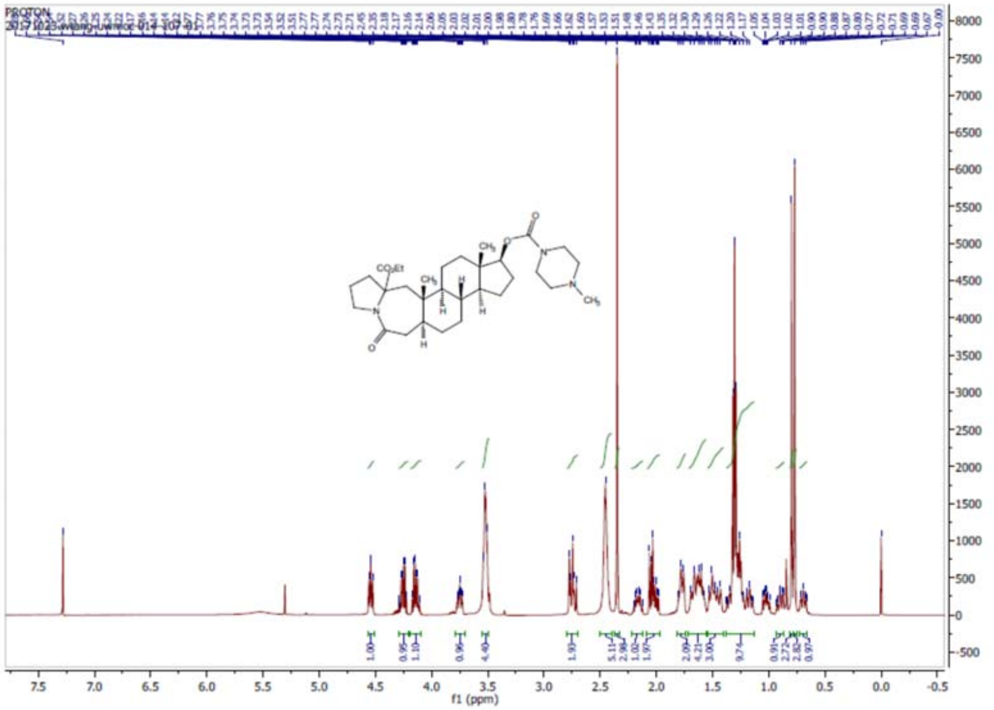

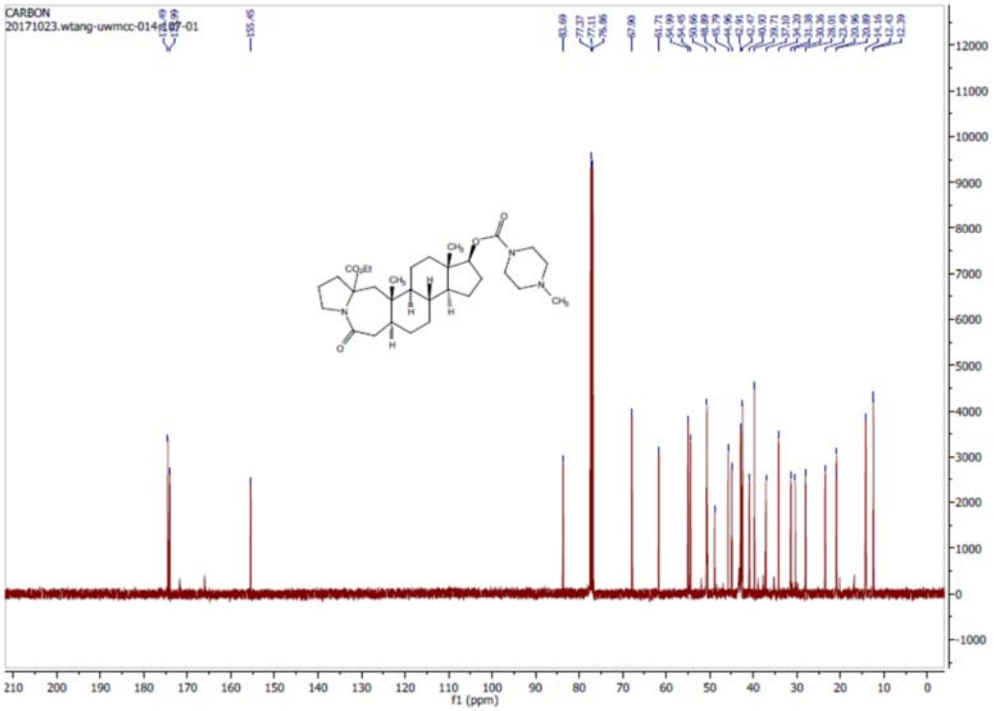

Supplementary Figure 102. ^{1}H and ^{13} C spectra of **40d**.

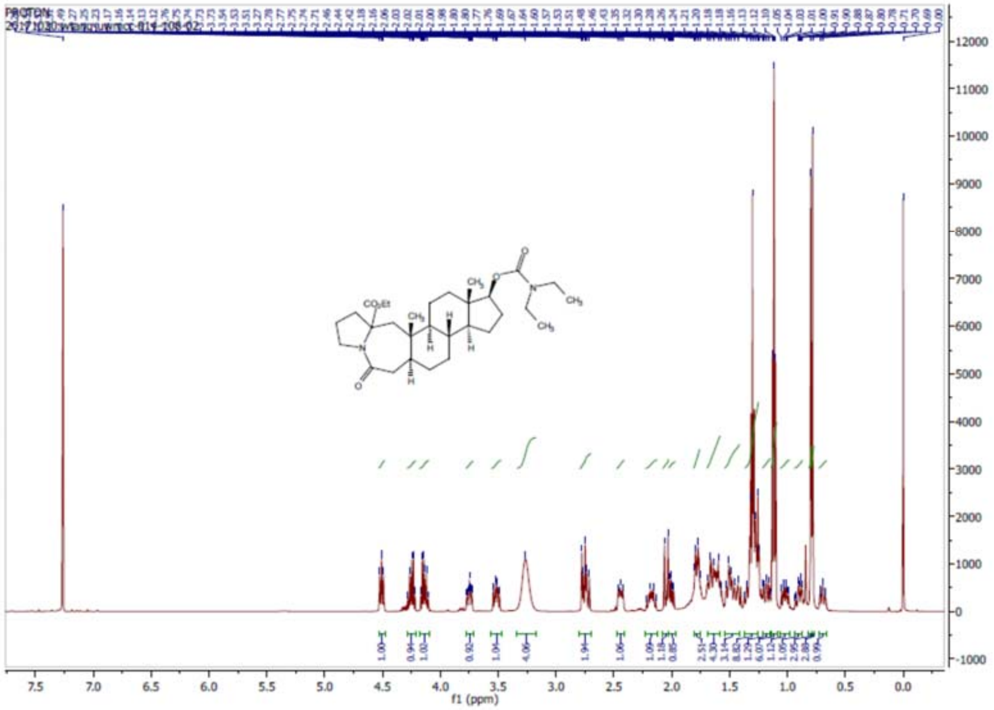

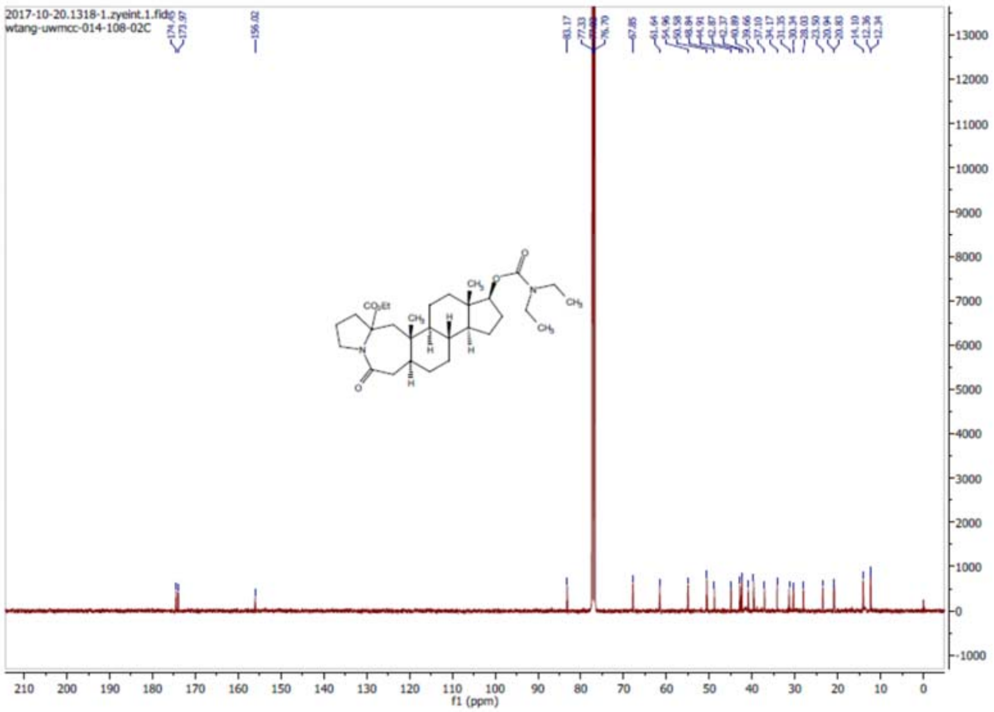

Supplementary Figure 103. ^{1}H and ^{13}C spectra of **40e**.

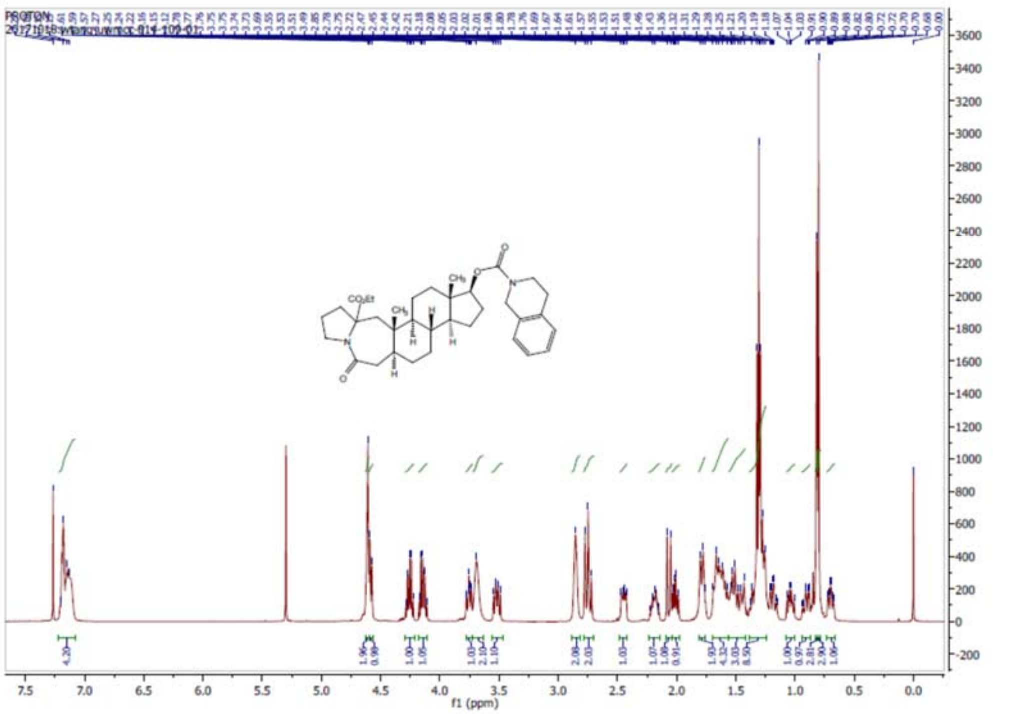

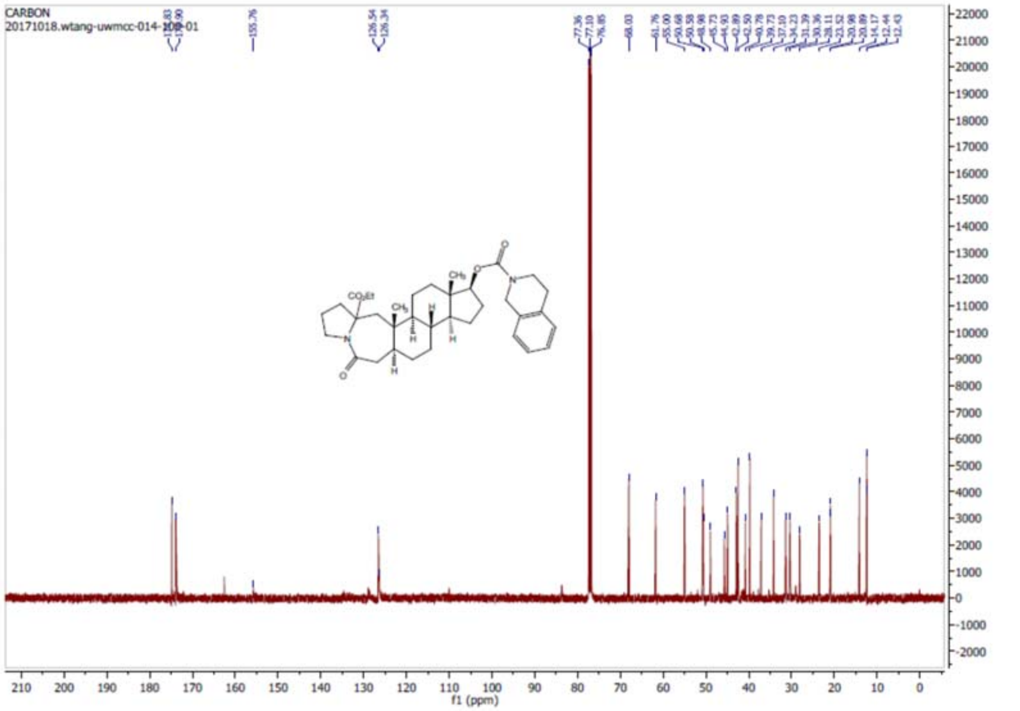

Supplementary Figure 104. ^{1}H and ^{13} C spectra of **40f**.

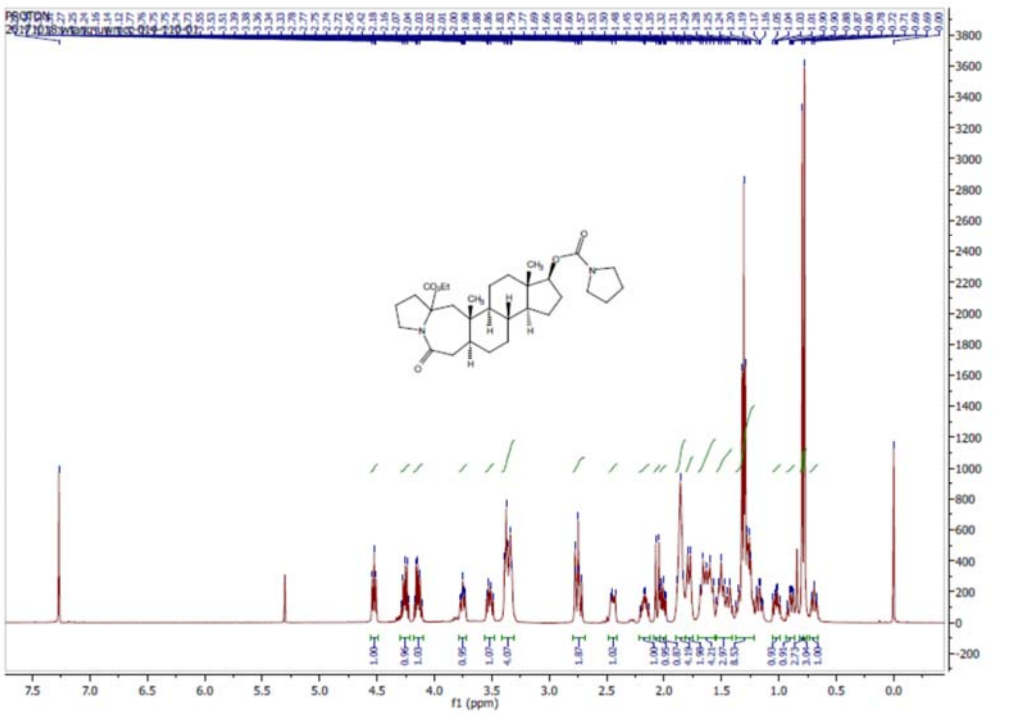

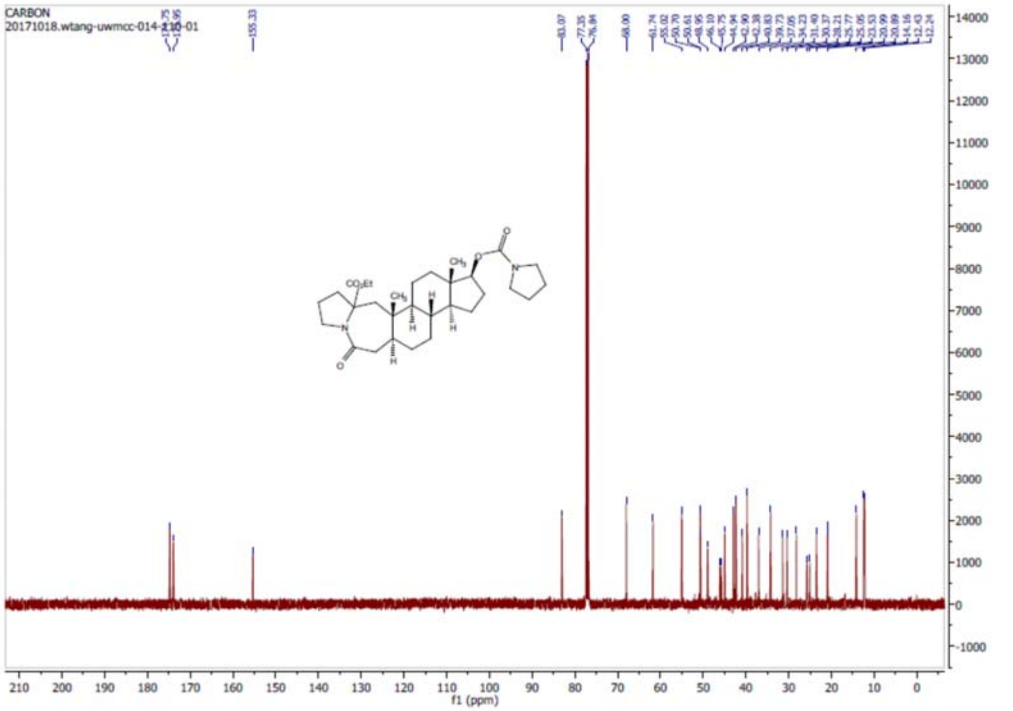

Supplementary Figure 105. ^{1}H and ^{13}C spectra of **40g**.

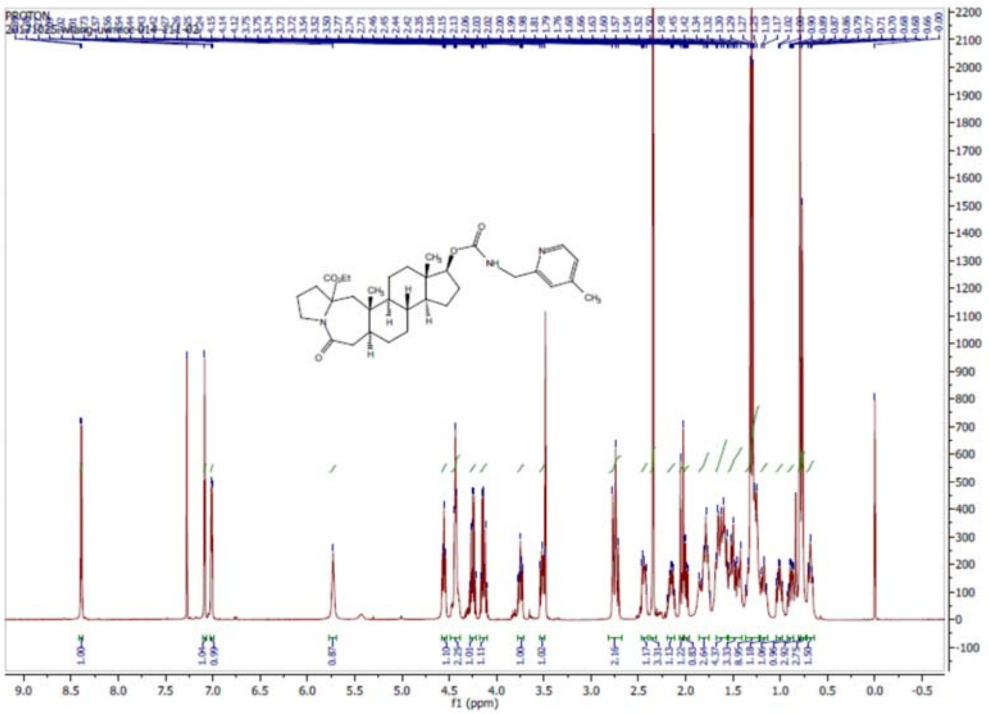

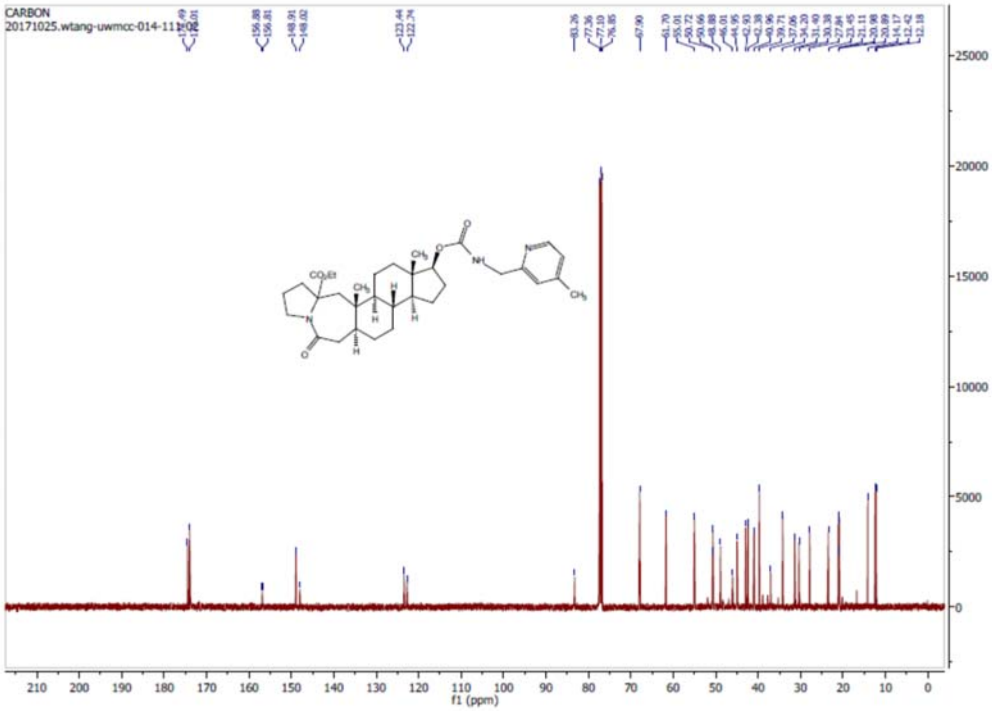

Supplementary Figure 106. ^{1}H and ^{13}C spectra of **40h**.

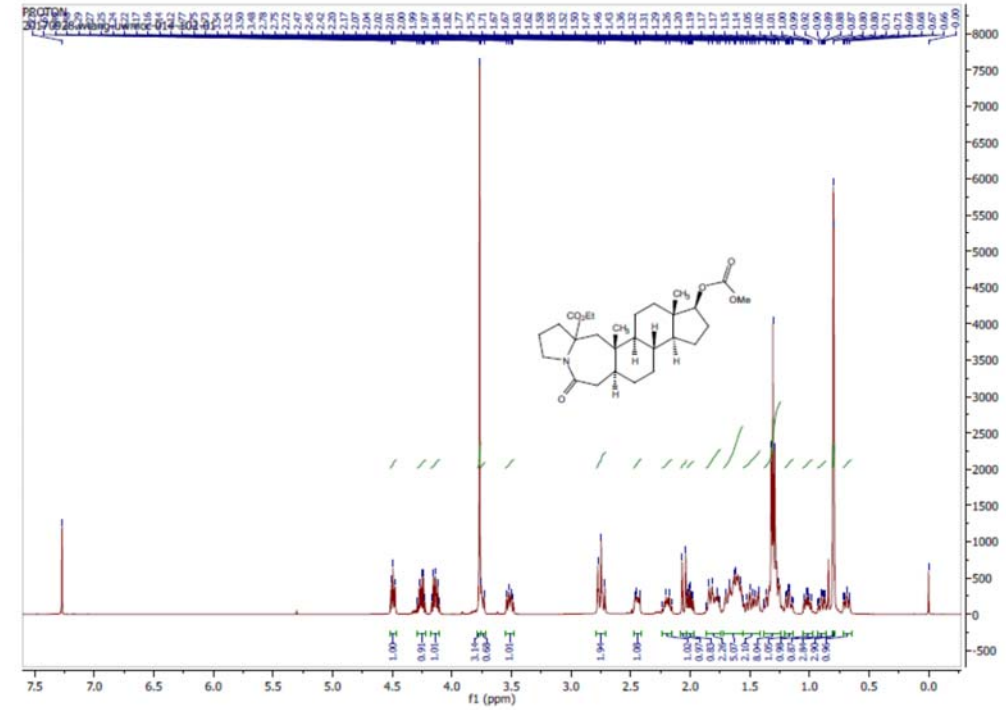

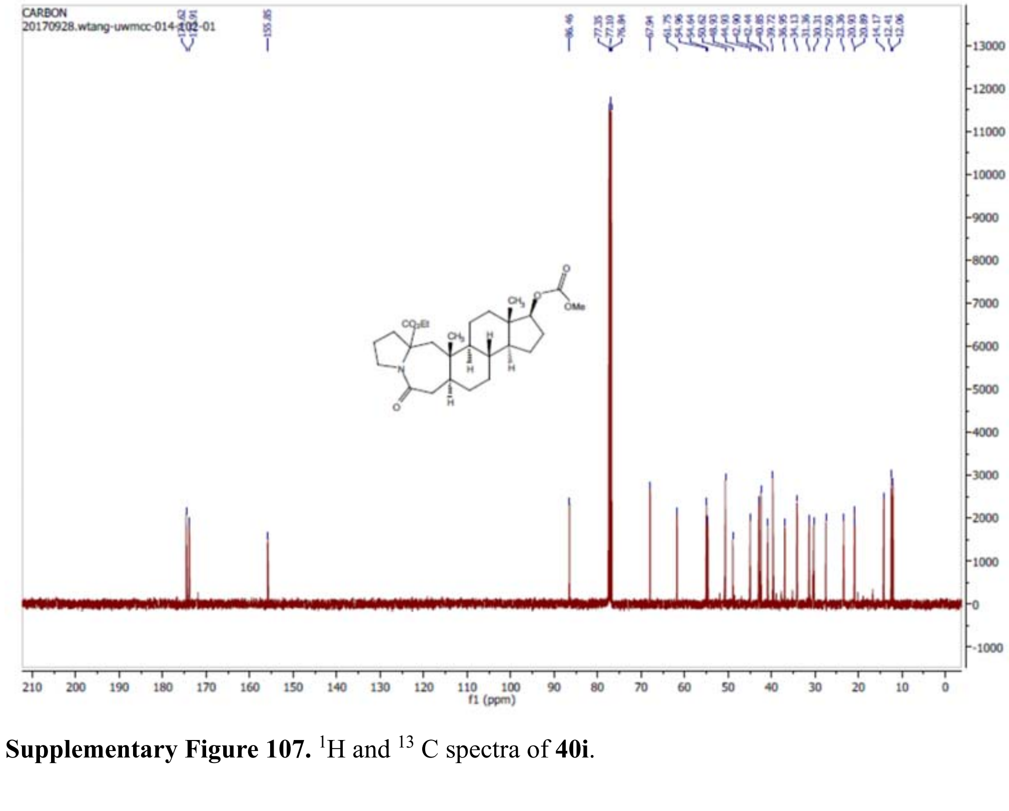

Supplementary Figure 107. ^{1}H and ^{13} C spectra of **40i**.

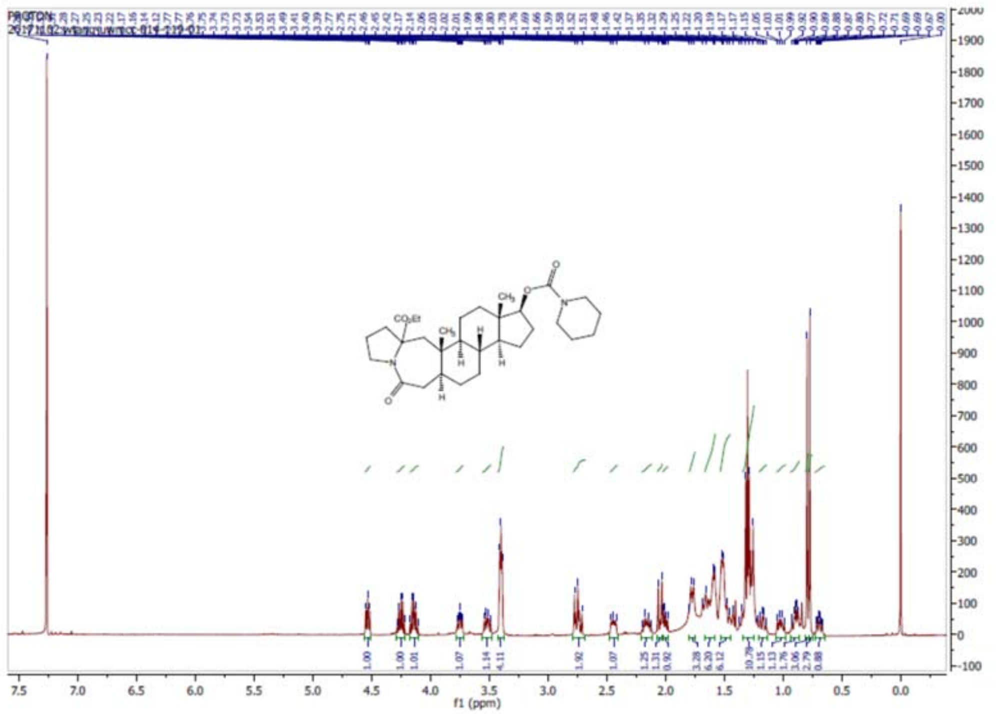

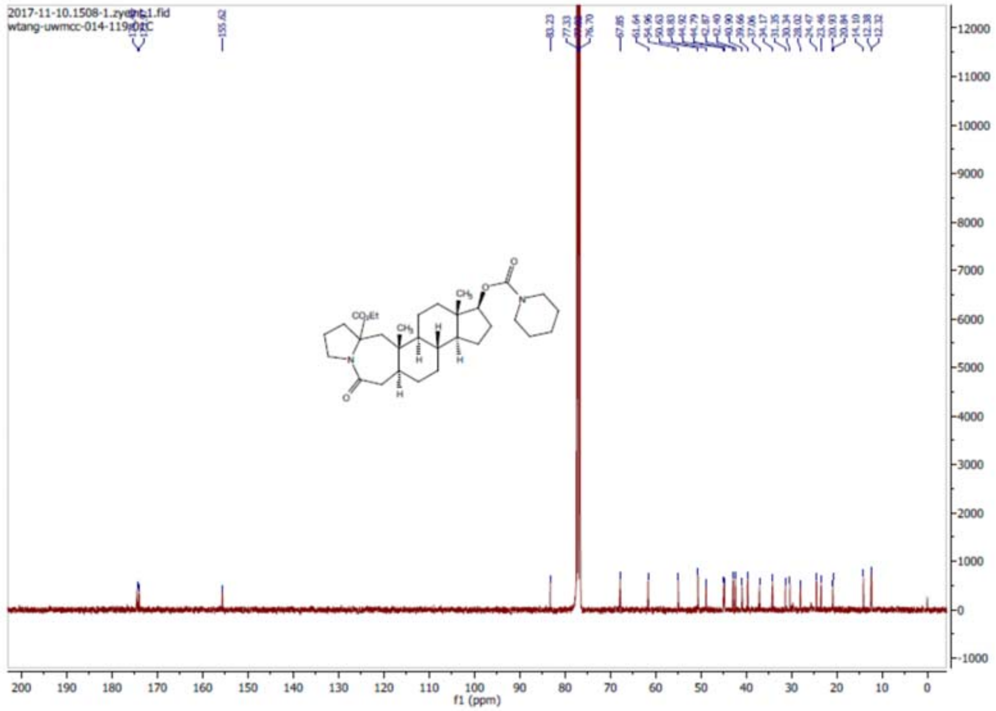

Supplementary Figure 108. ^{1}H and ^{13}C spectra of **40j**.

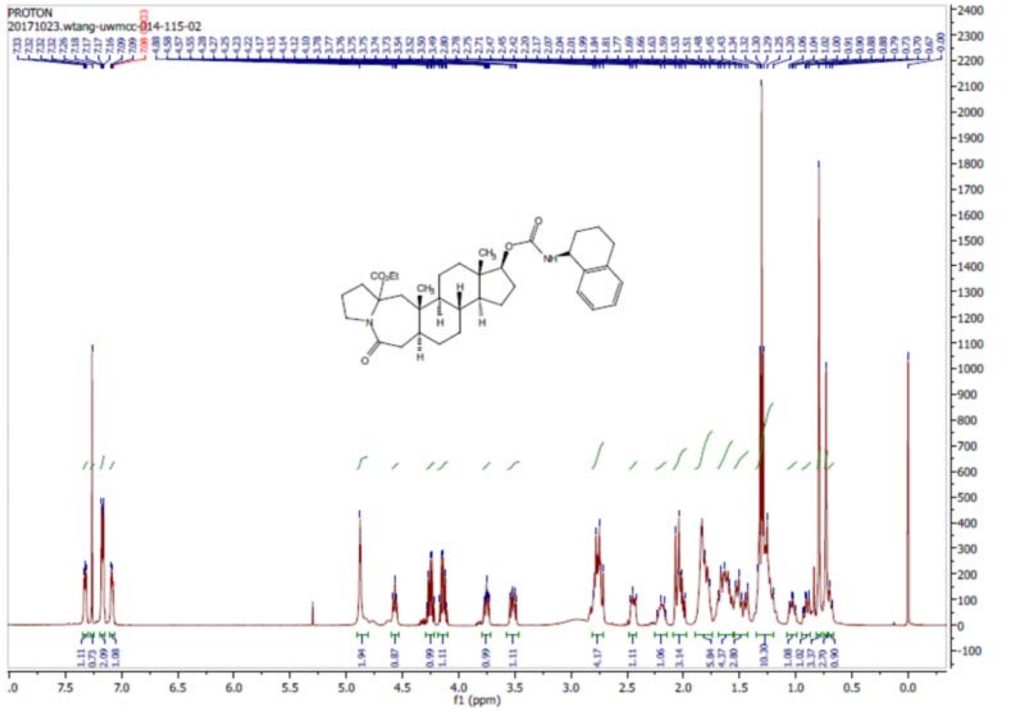

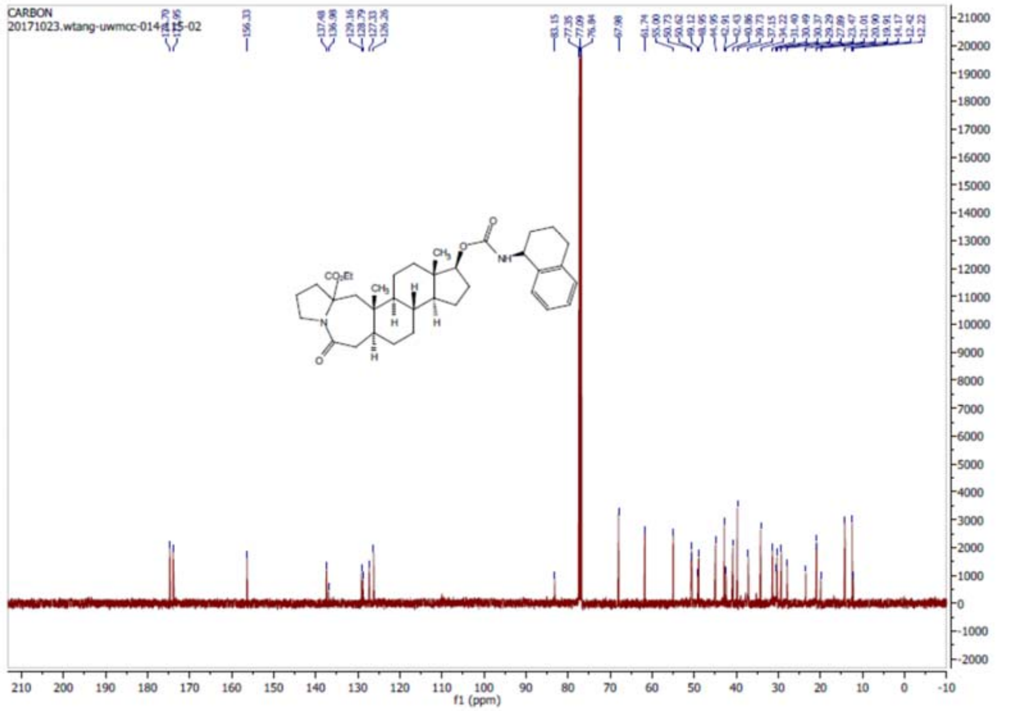

Supplementary Figure 109. ^{1}H and ^{13}C spectra of **40k**.

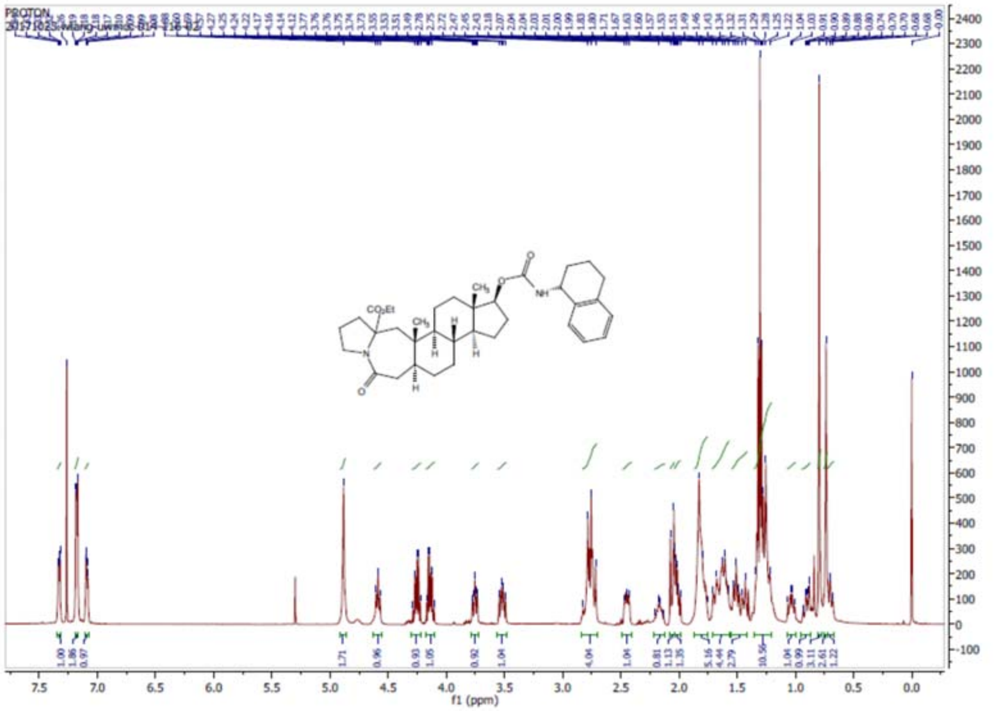

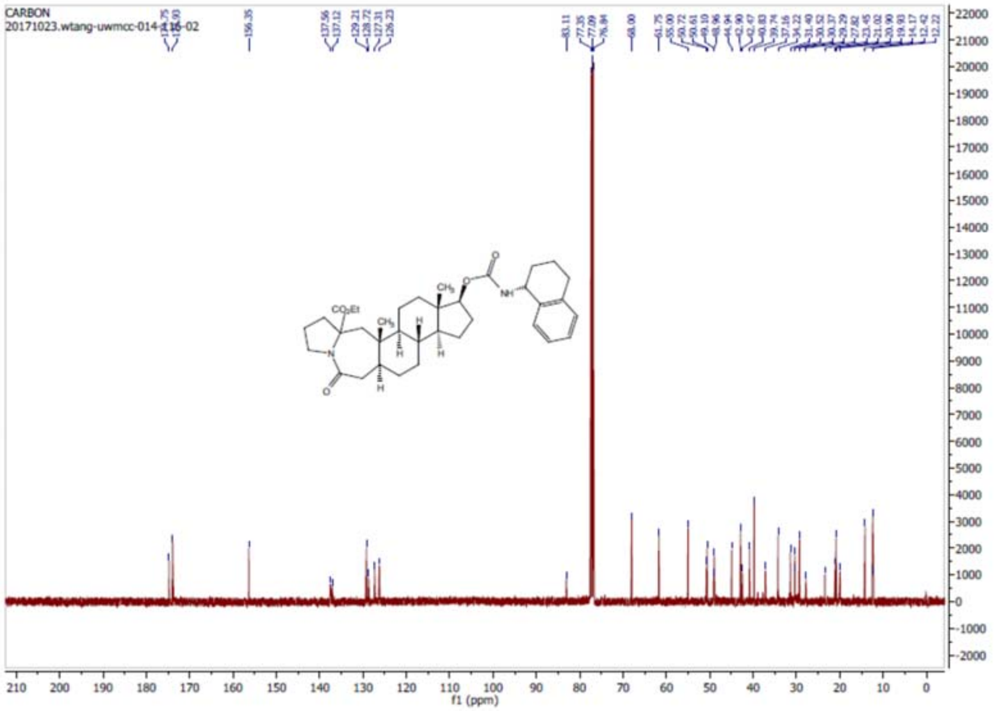

Supplementary Figure 110. ^{1}H and ^{13}C spectra of **40l**.

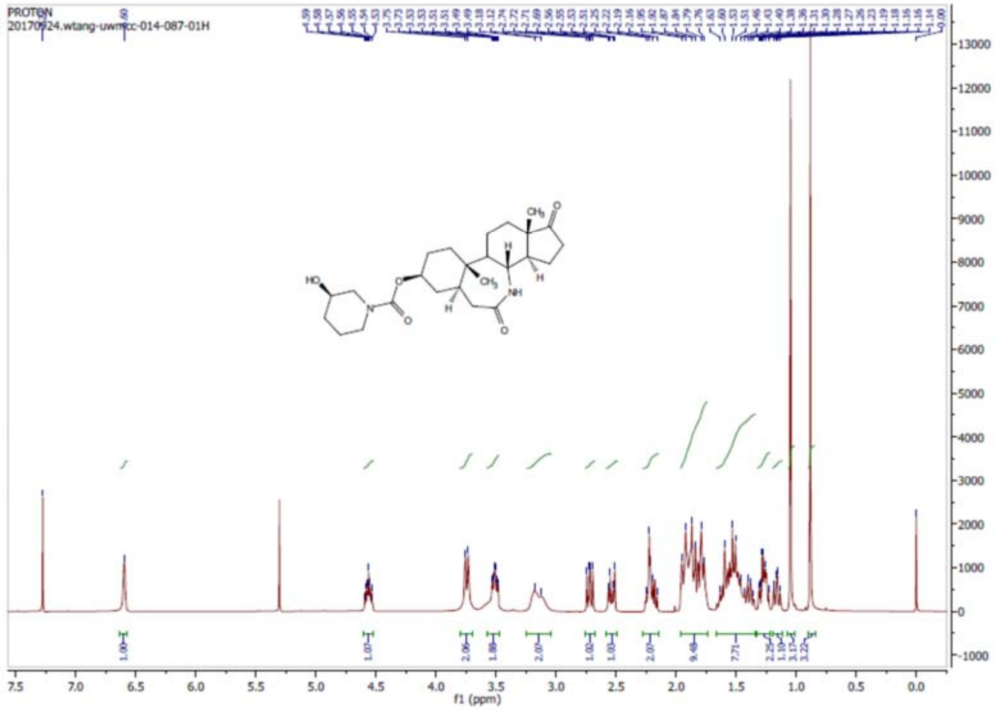

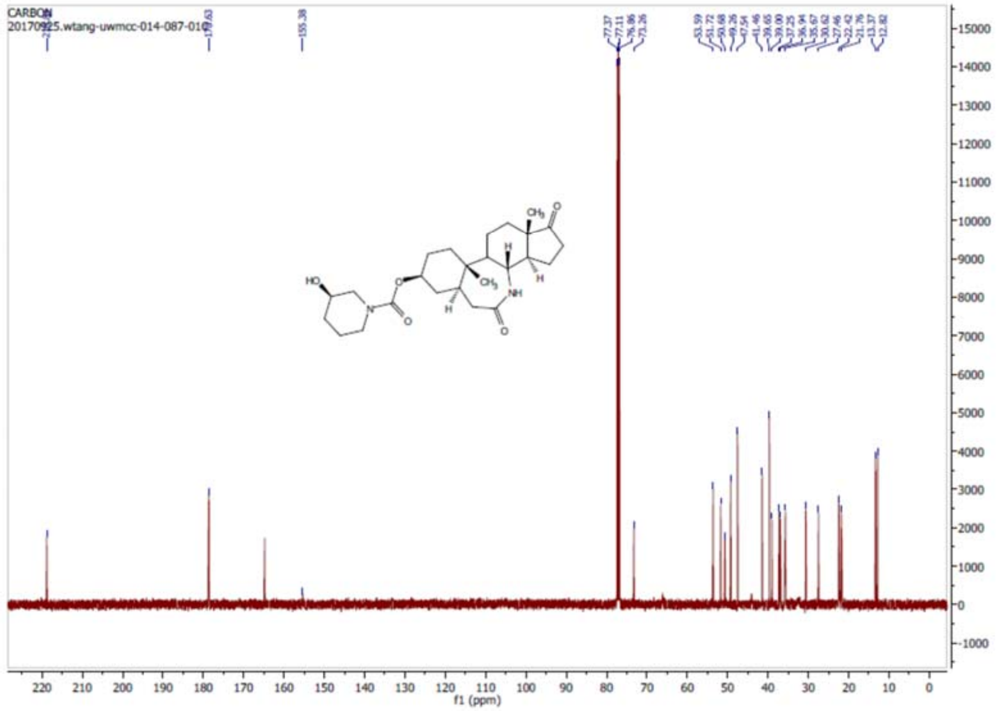

Supplementary Figure 111. ^{1}H and ^{13}C spectra of **41aa**.

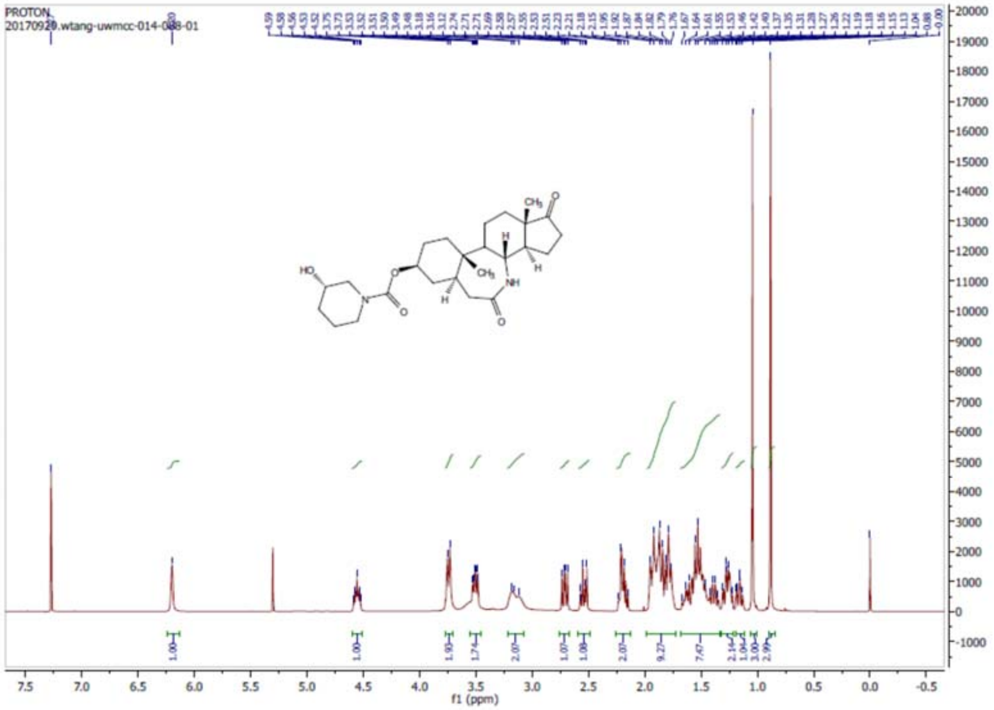

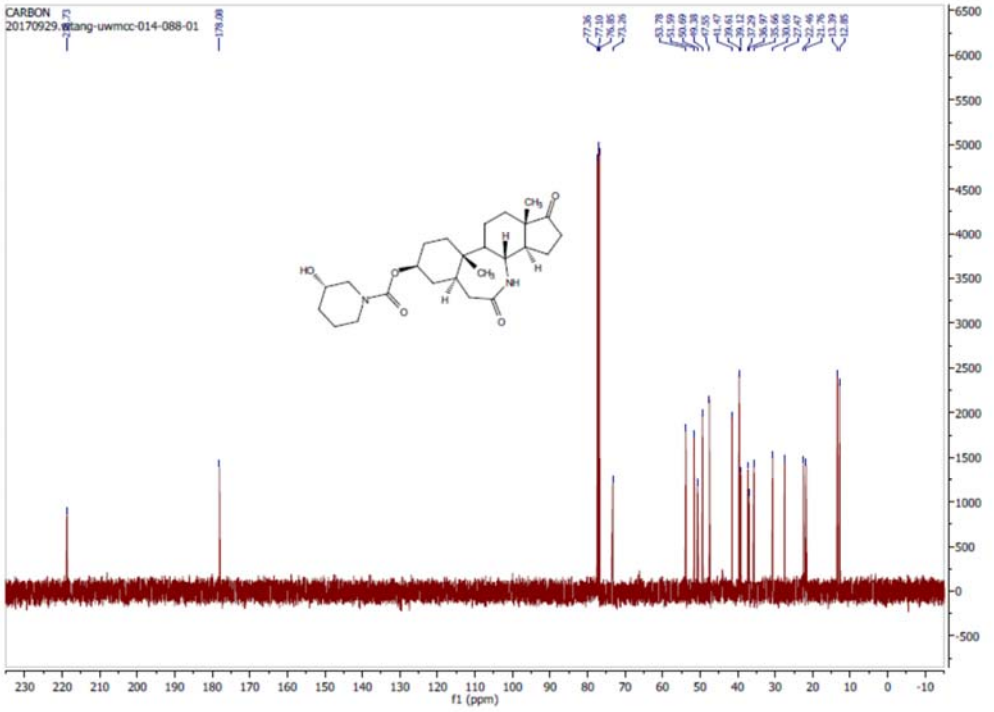

Supplementary Figure 112. ^{1}H and ^{13} C spectra of **41ab**.

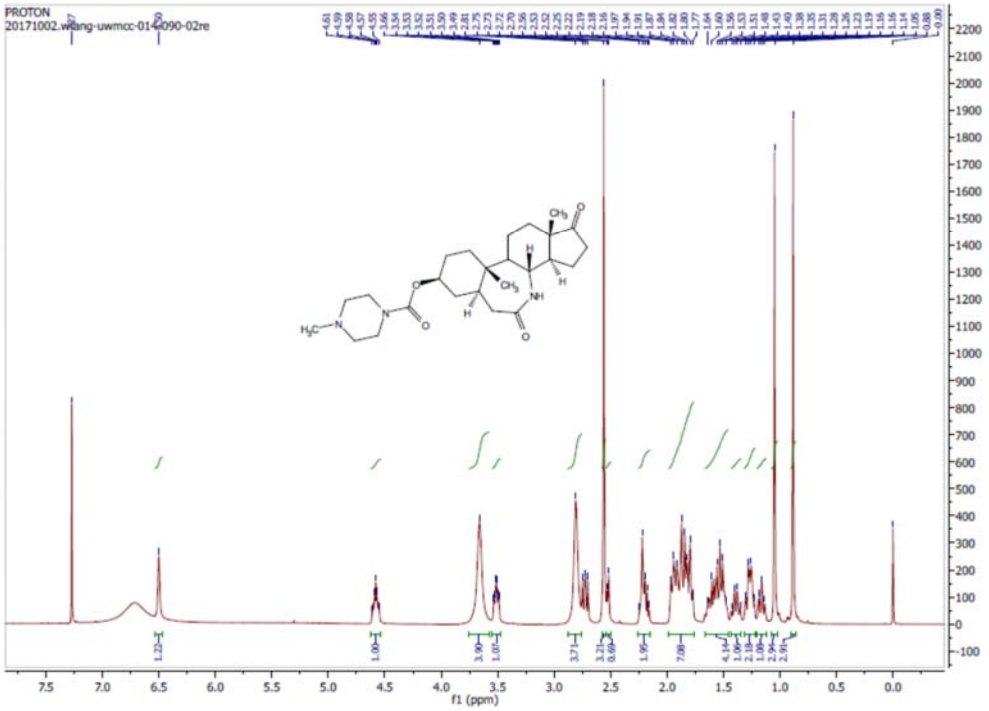

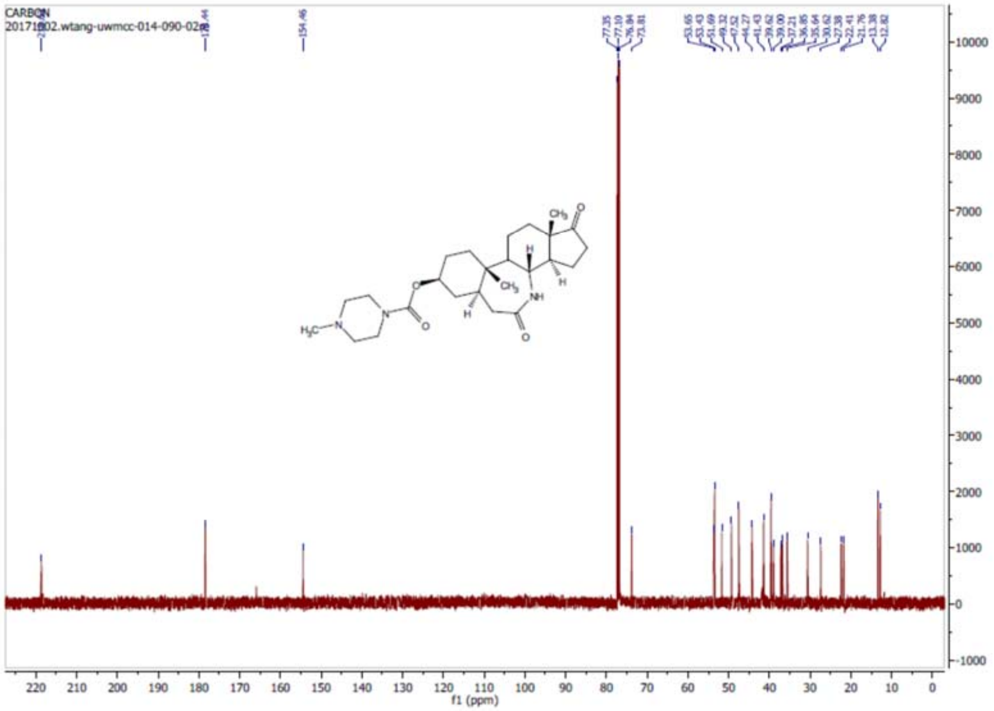

Supplementary Figure 113. ^{1}H and ^{13}C spectra of **41ab**.

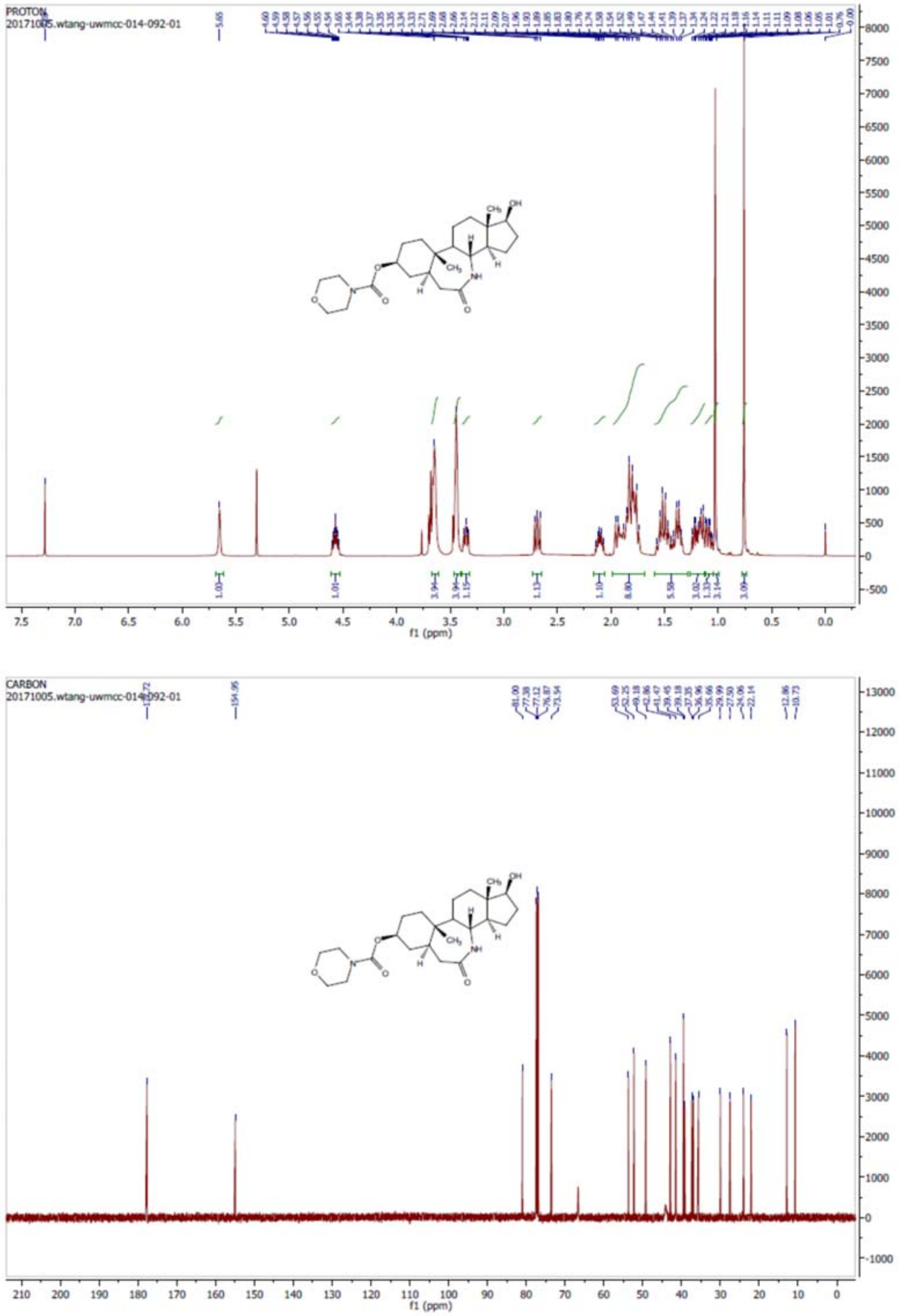

Supplementary Figure 114. ^{1}H and ^{13} C spectra of **41ac**.

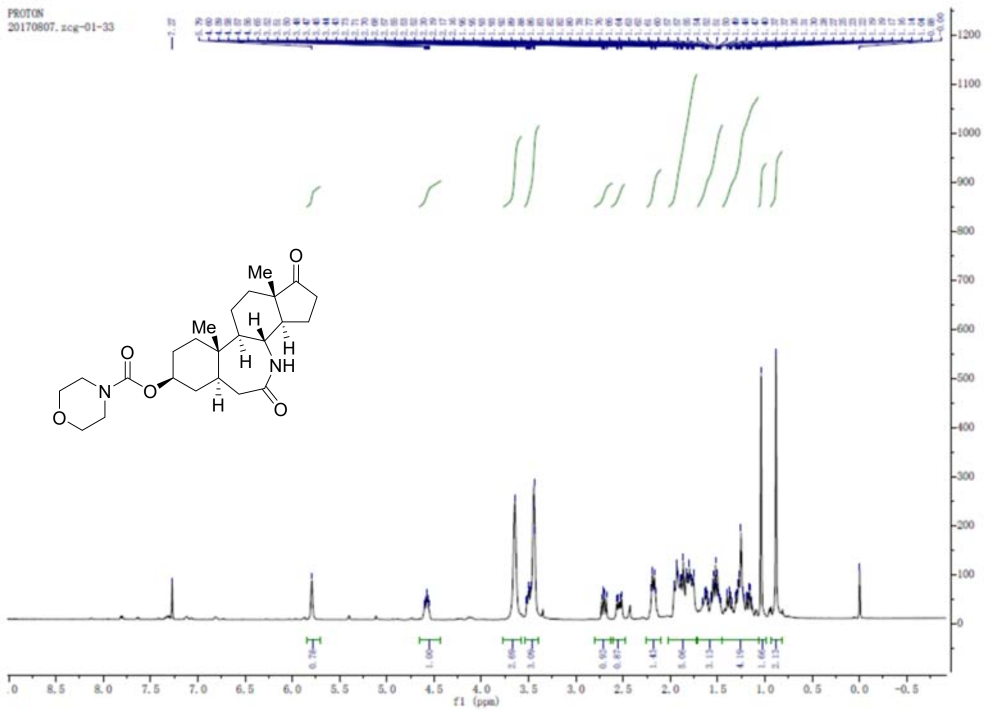

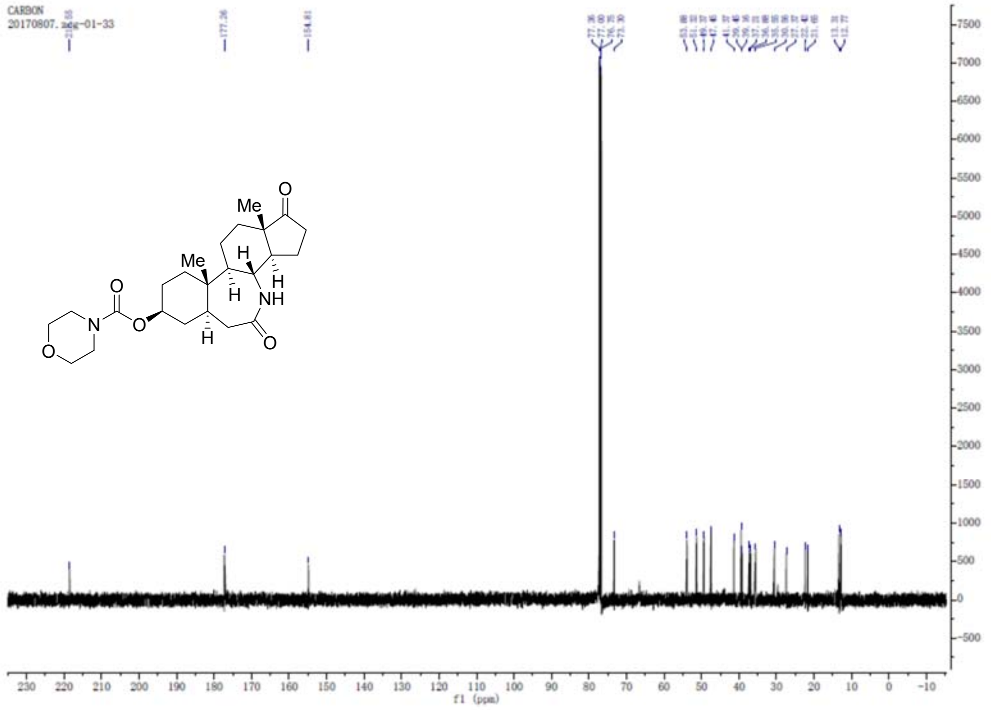

Supplementary Figure 115. ^{1}H and ^{13}C spectra of **41ad**.

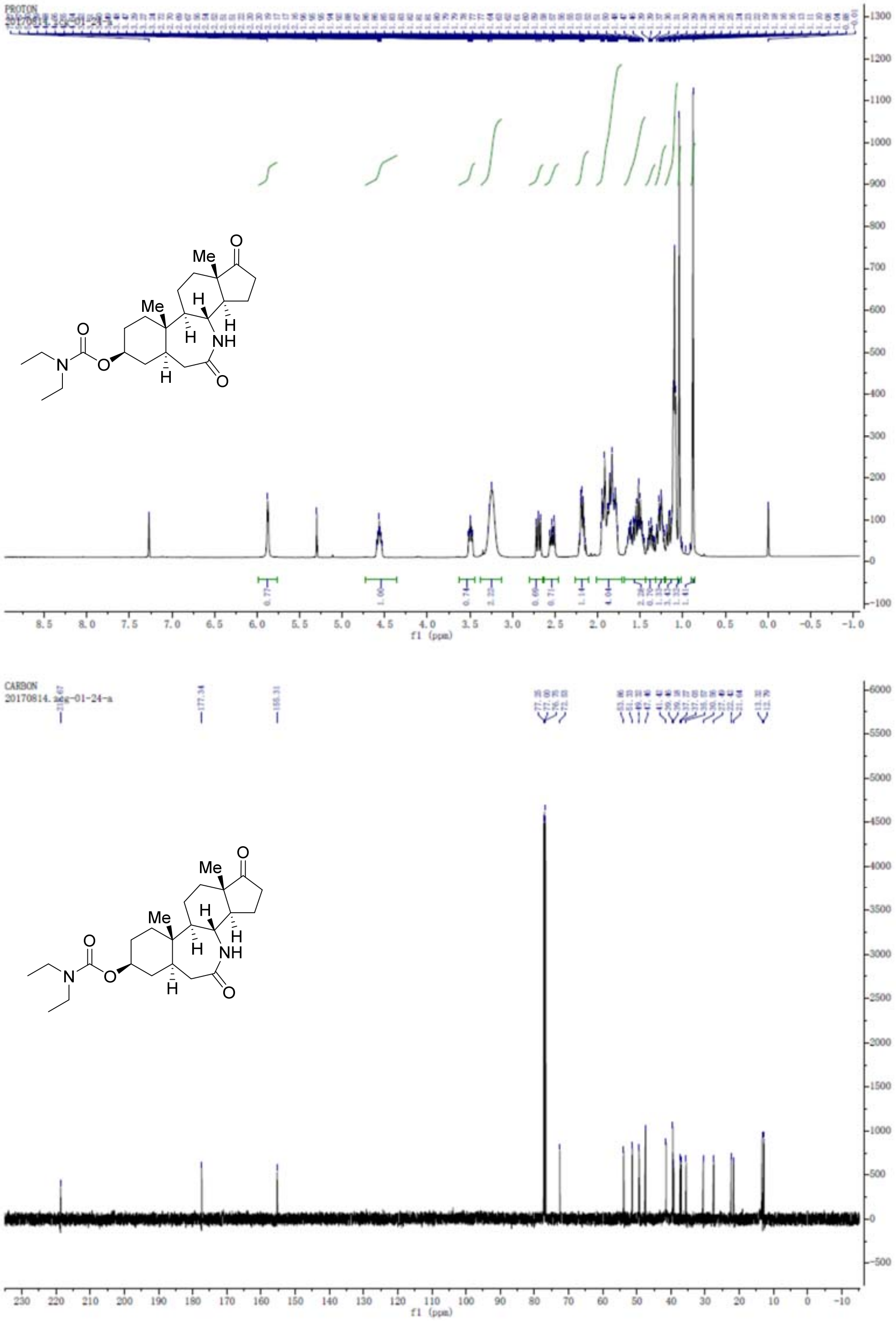

Supplementary Figure 116. ^{1}H and ^{13} C spectra of **41ae**.

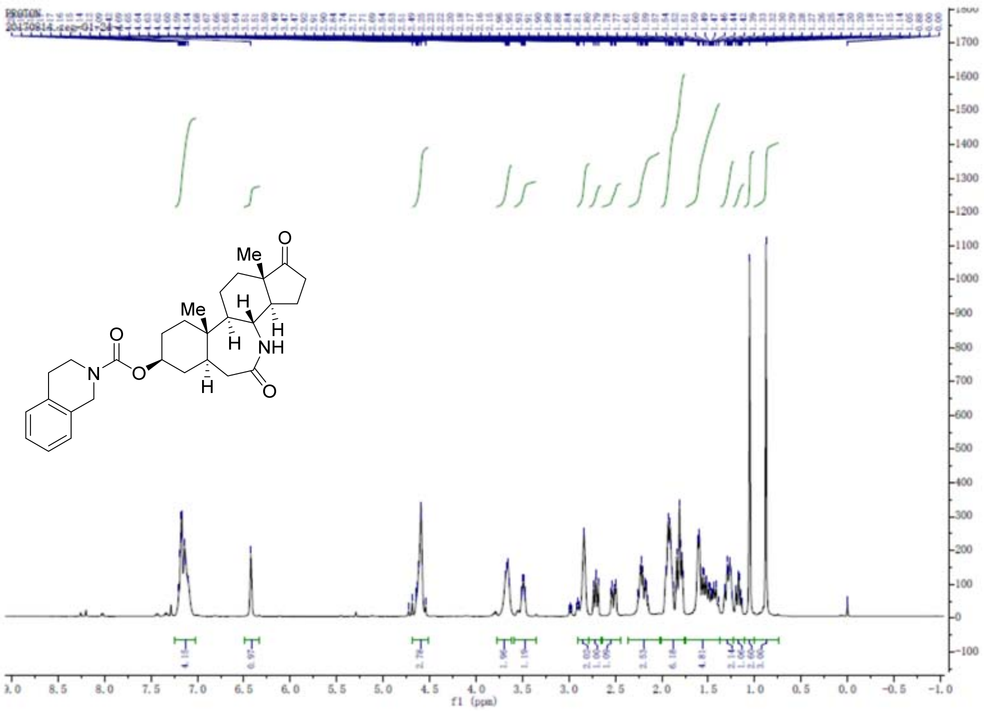

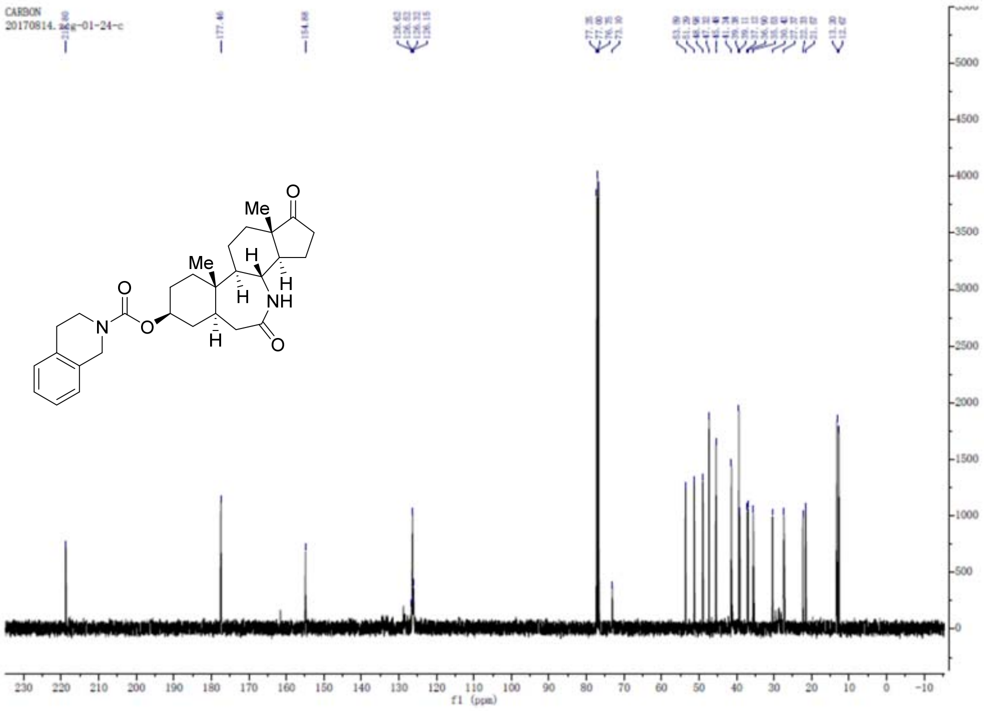

Supplementary Figure 117. ^{1}H and ^{13}C spectra of **41af**.

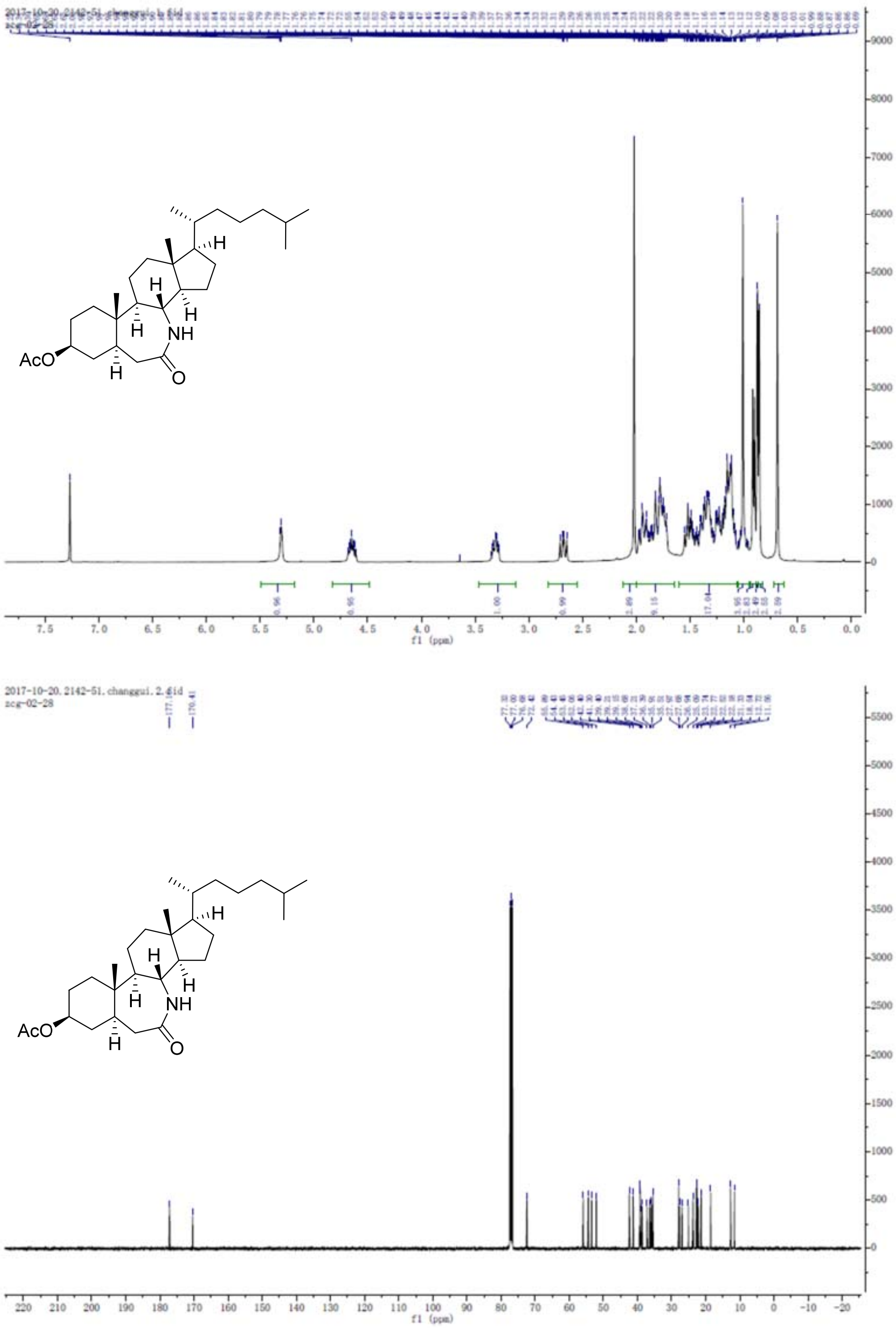

Supplementary Figure 118. ^{1}H and ^{13} C spectra of **41bb**.

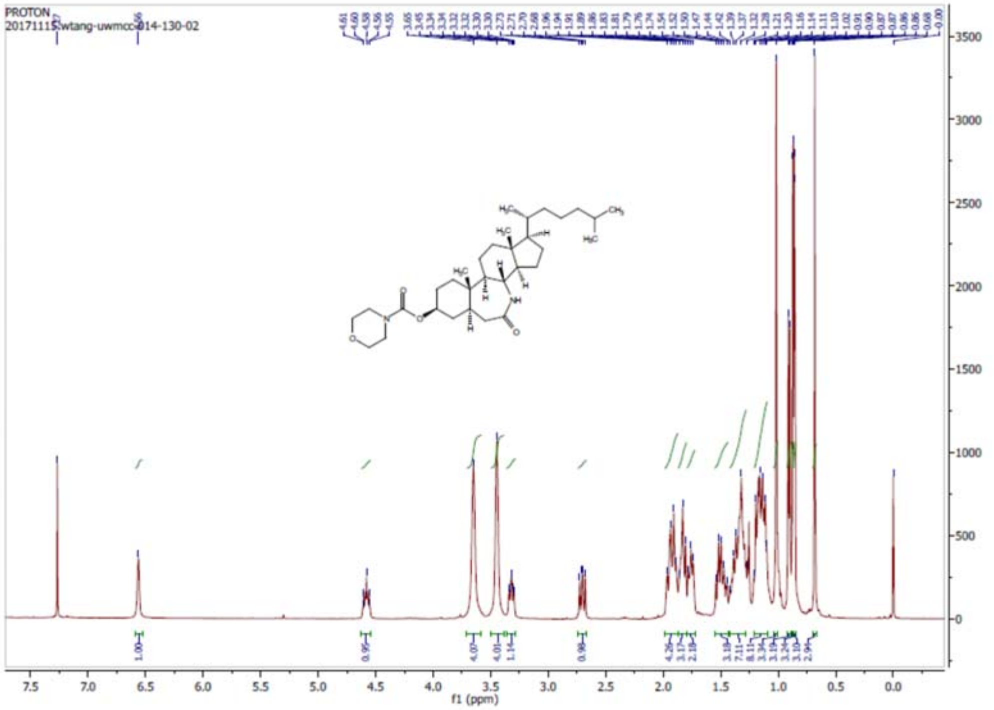

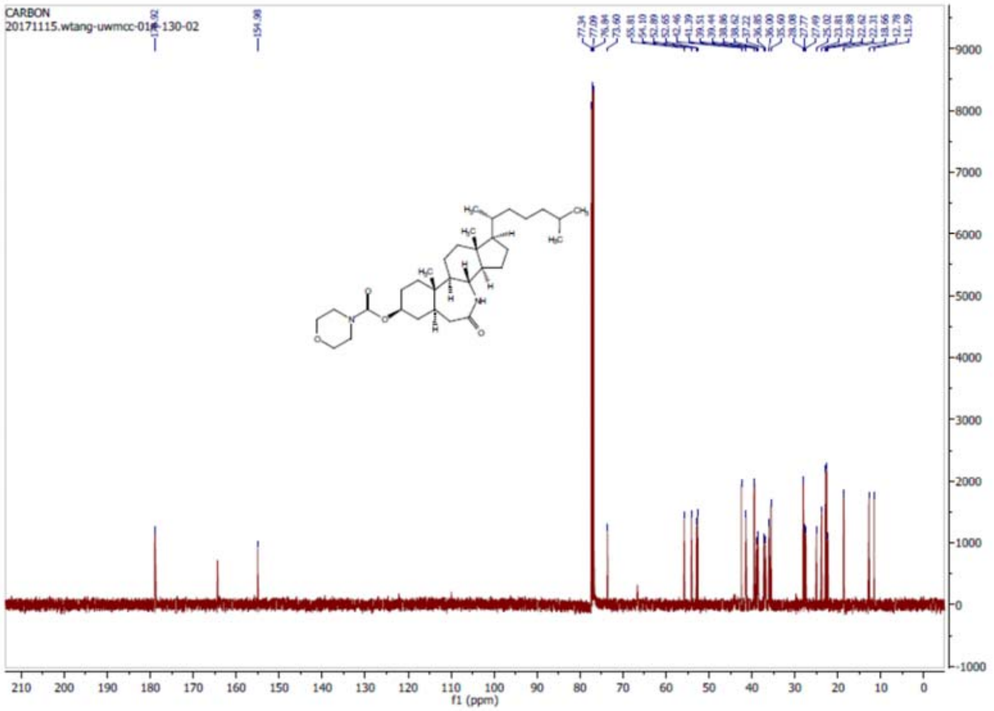

Supplementary Figure 119. ^{1}H and ^{13} C spectra of **41bc**.

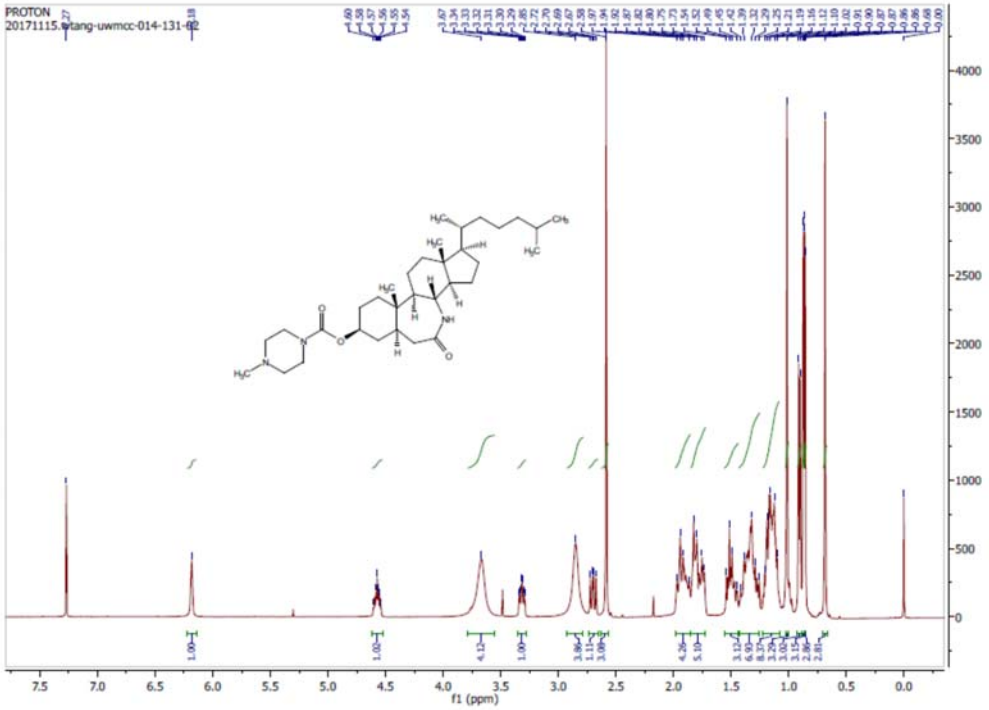

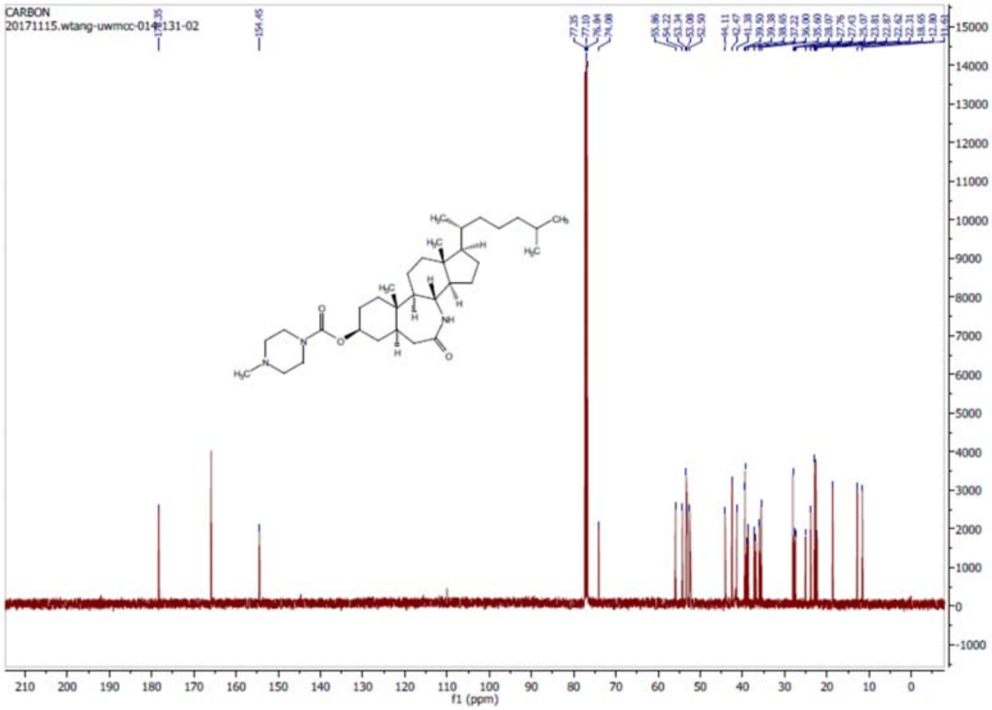

Supplementary Figure 120. ^{1}H and ^{13} C spectra of **41bd**.

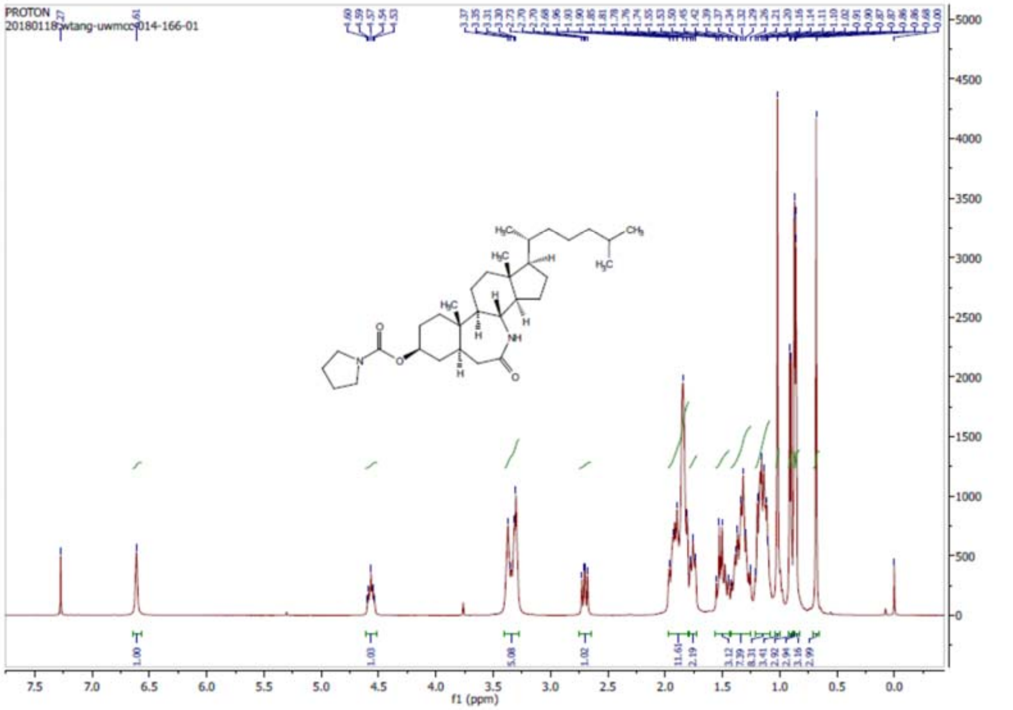

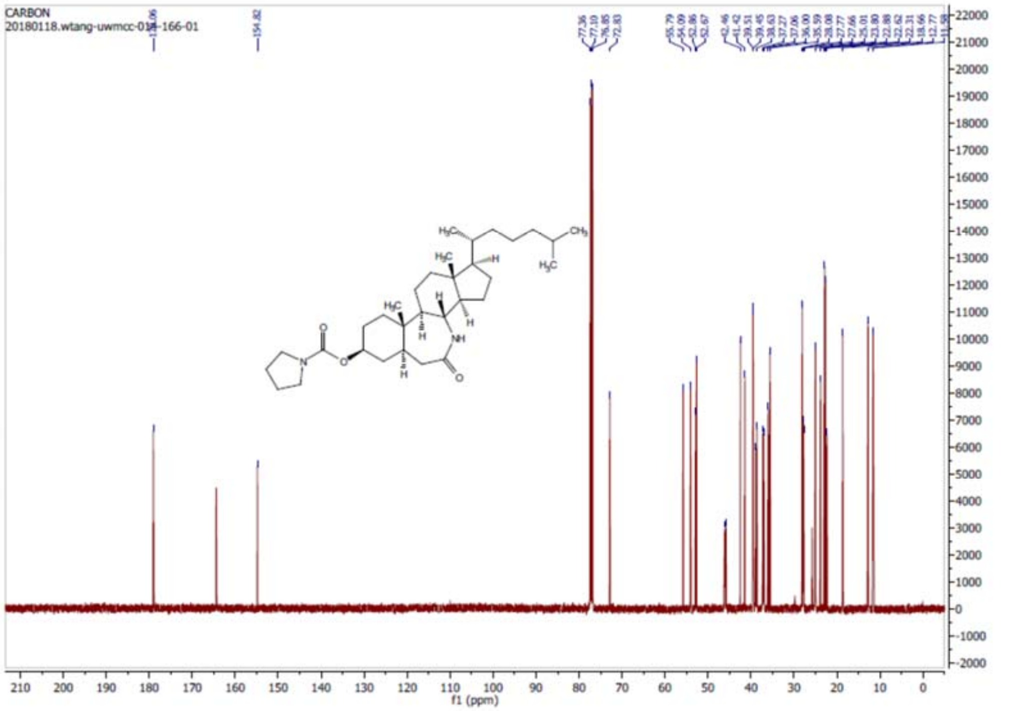

Supplementary Figure 121. ^{1}H and ^{13} C spectra of **41be**.

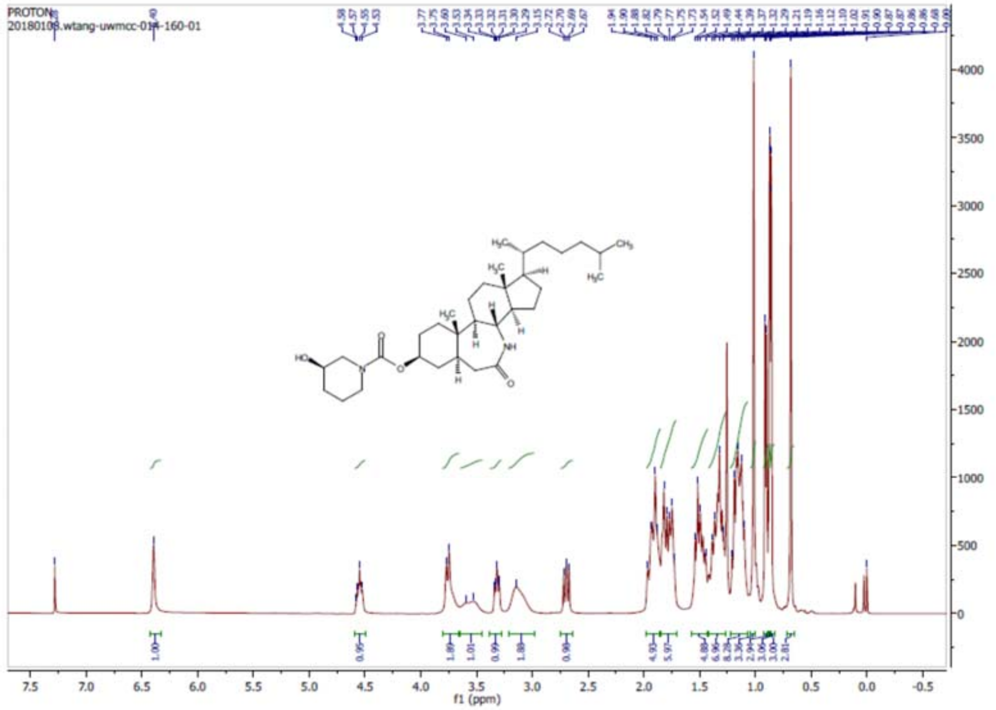

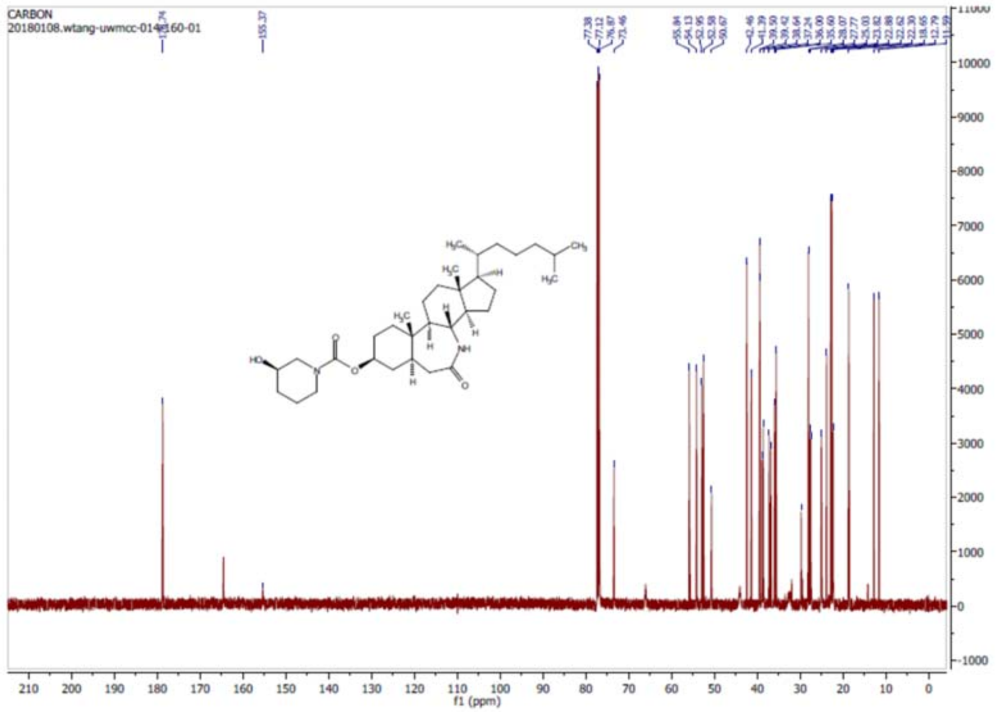

Supplementary Figure 122. ^{1}H and ^{13}C spectra of **41bf**.

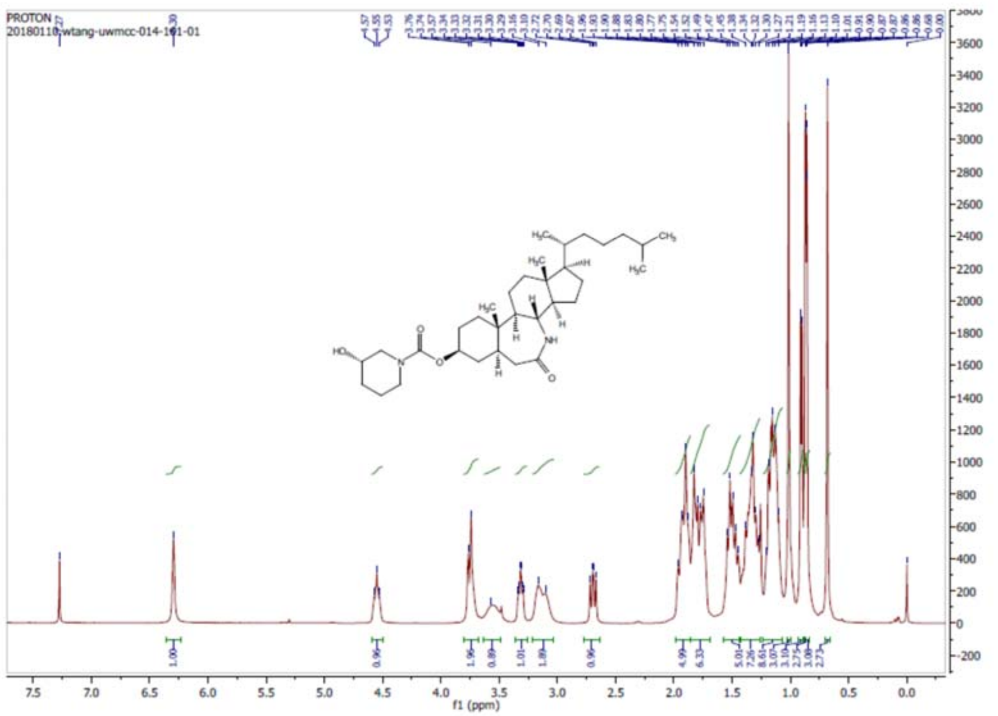

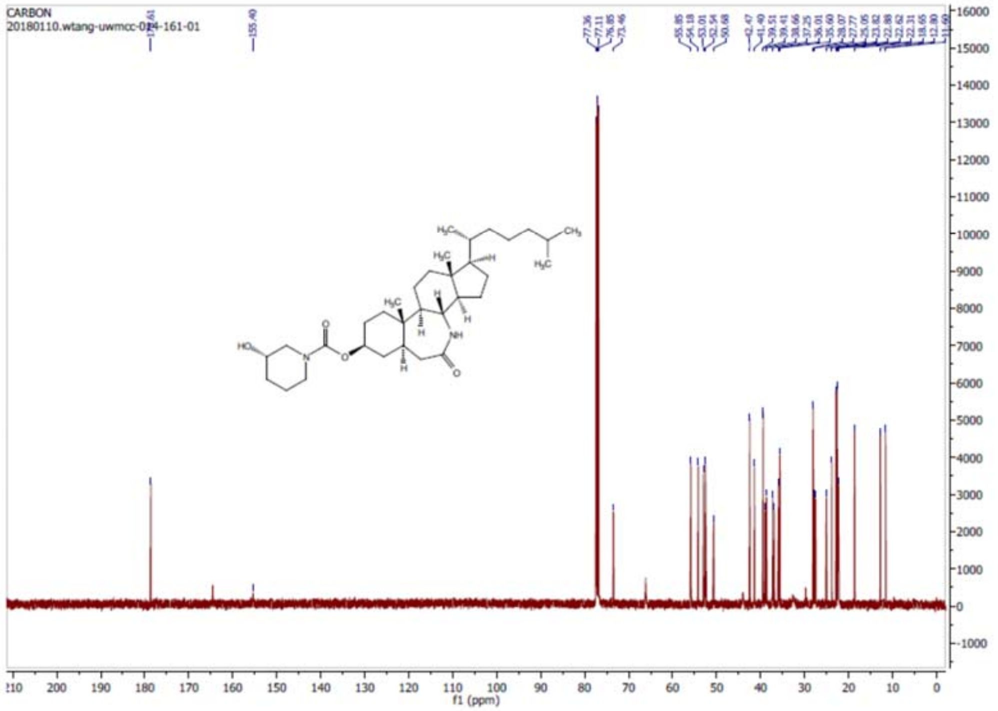

Supplementary Figure 123. ^{1}H and ^{13}C spectra of **41bg**.

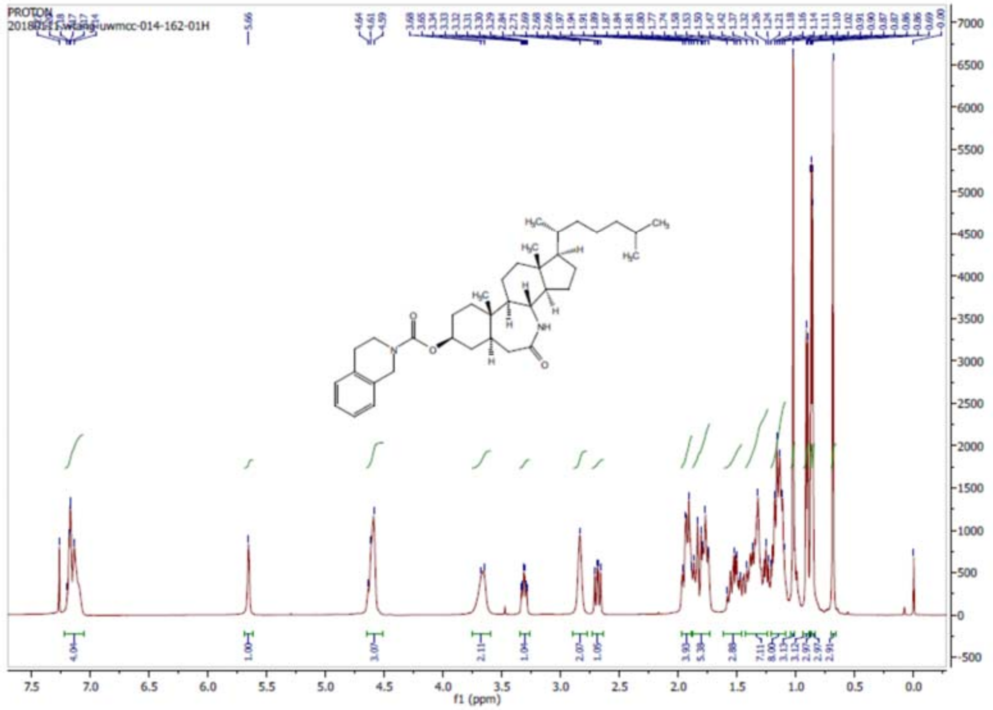

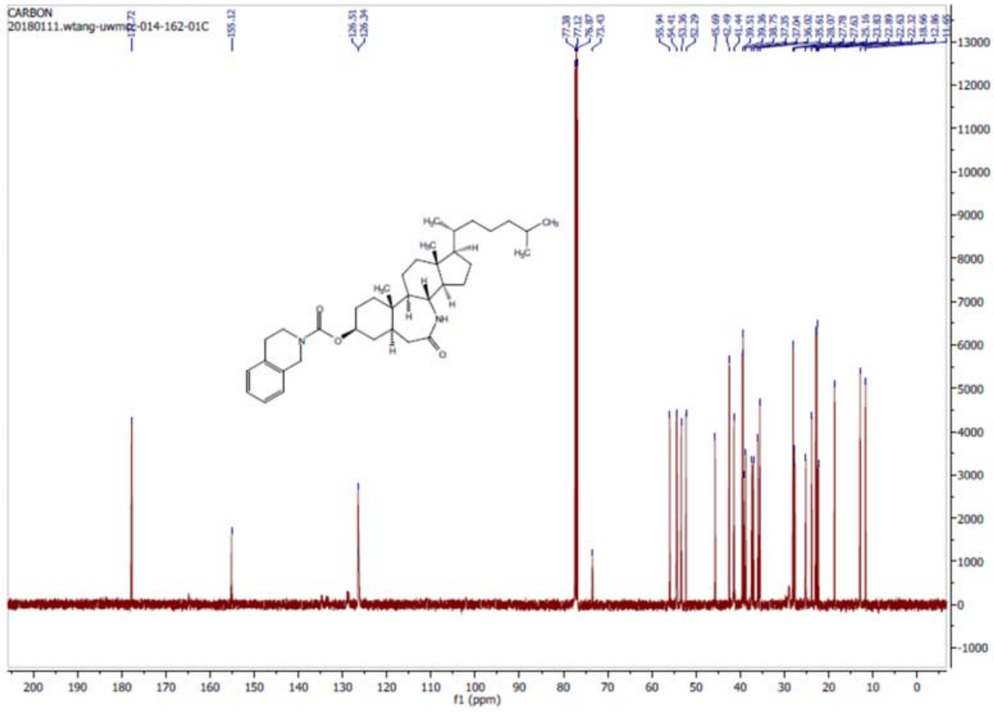

Supplementary Figure 124. ^{1}H and ^{13} C spectra of **41bh**.

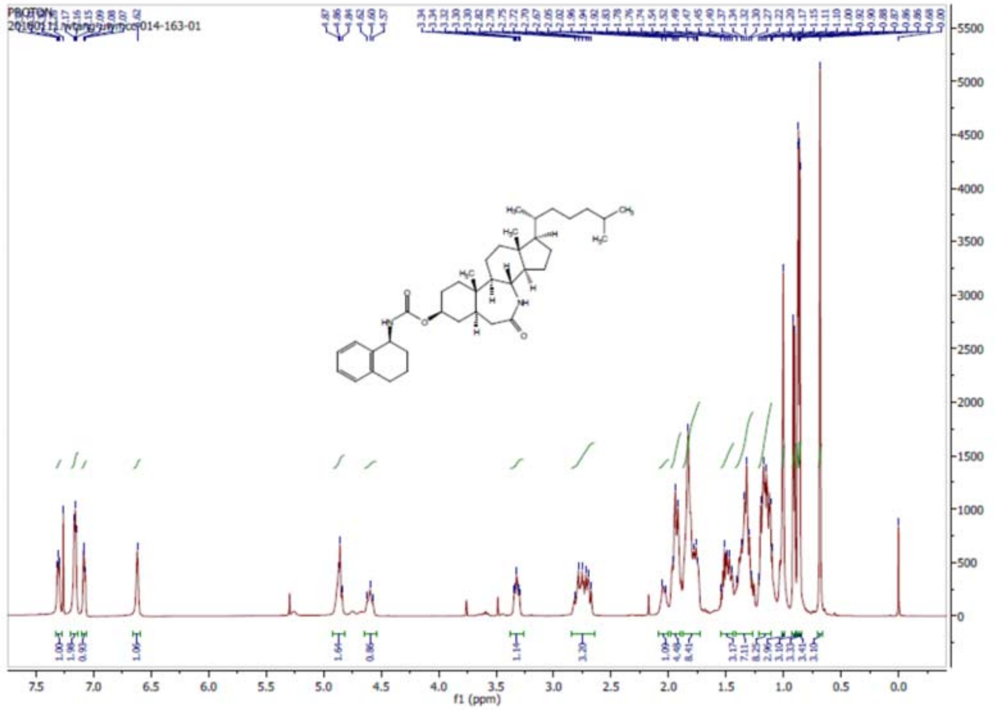

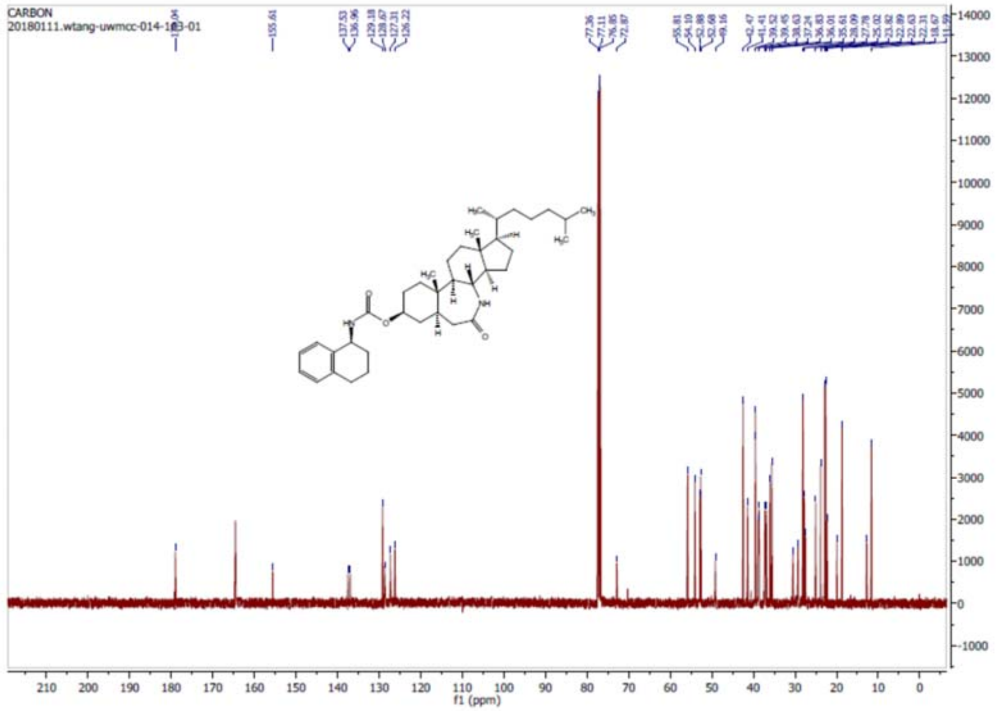

Supplementary Figure 125. ^{1}H and ^{13}C spectra of **41bi**.

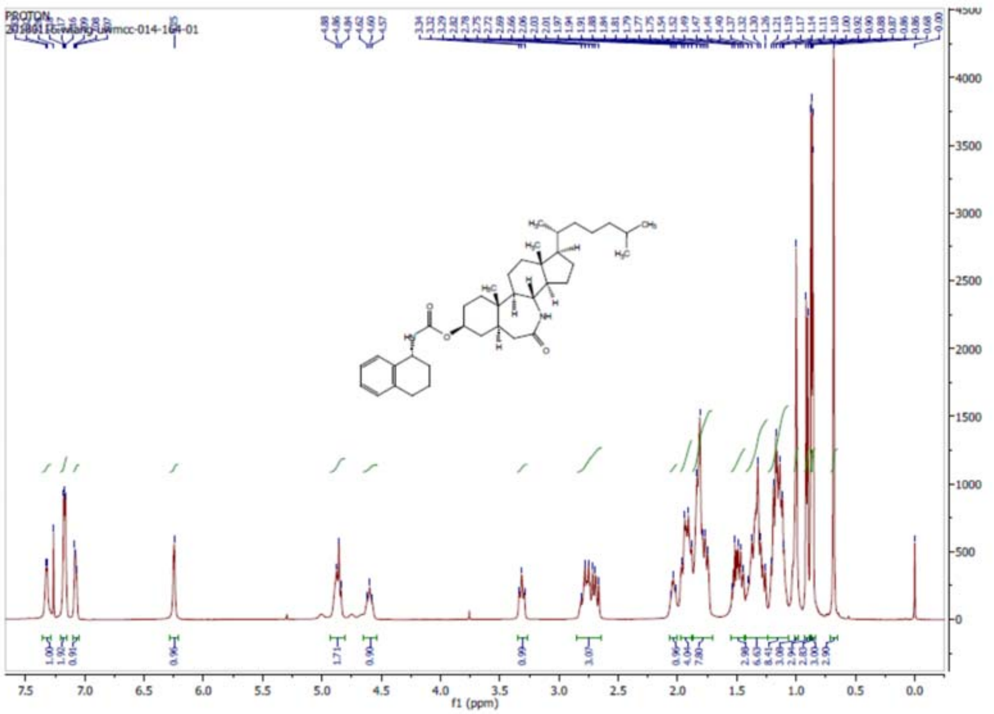

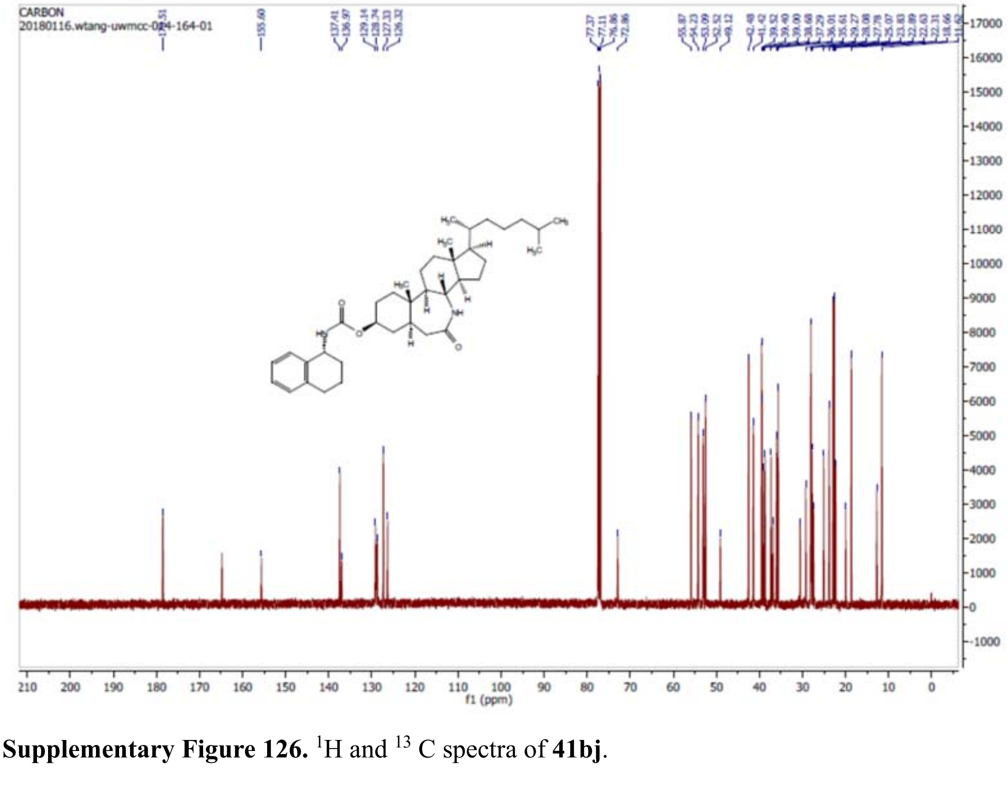

Supplementary Figure 126. ^{1}H and ^{13}C spectra of **41bj**.

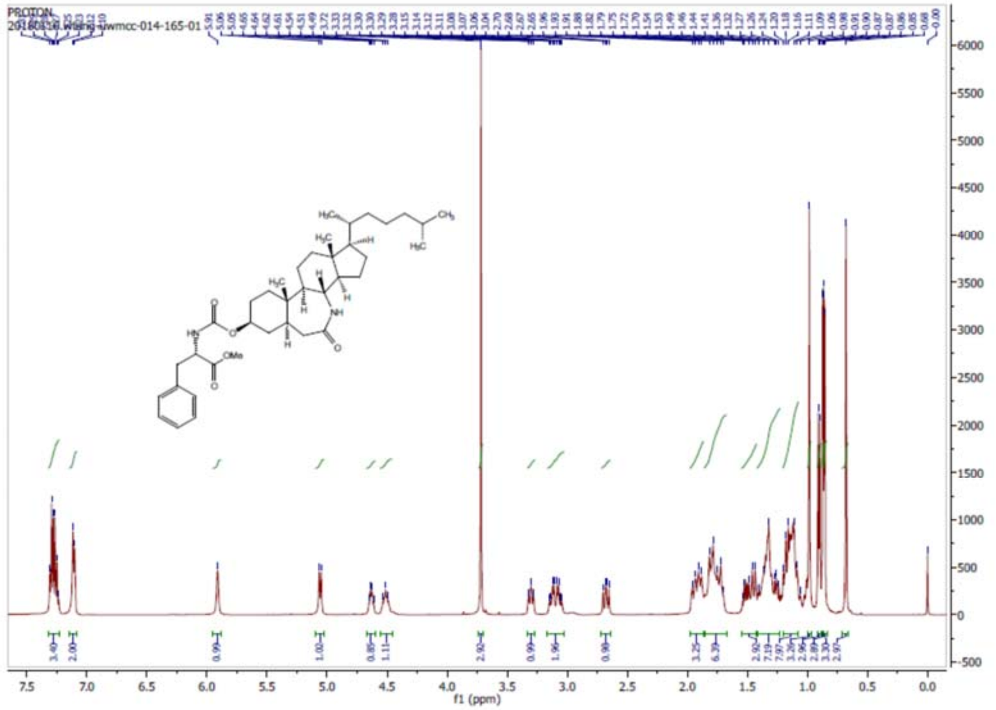

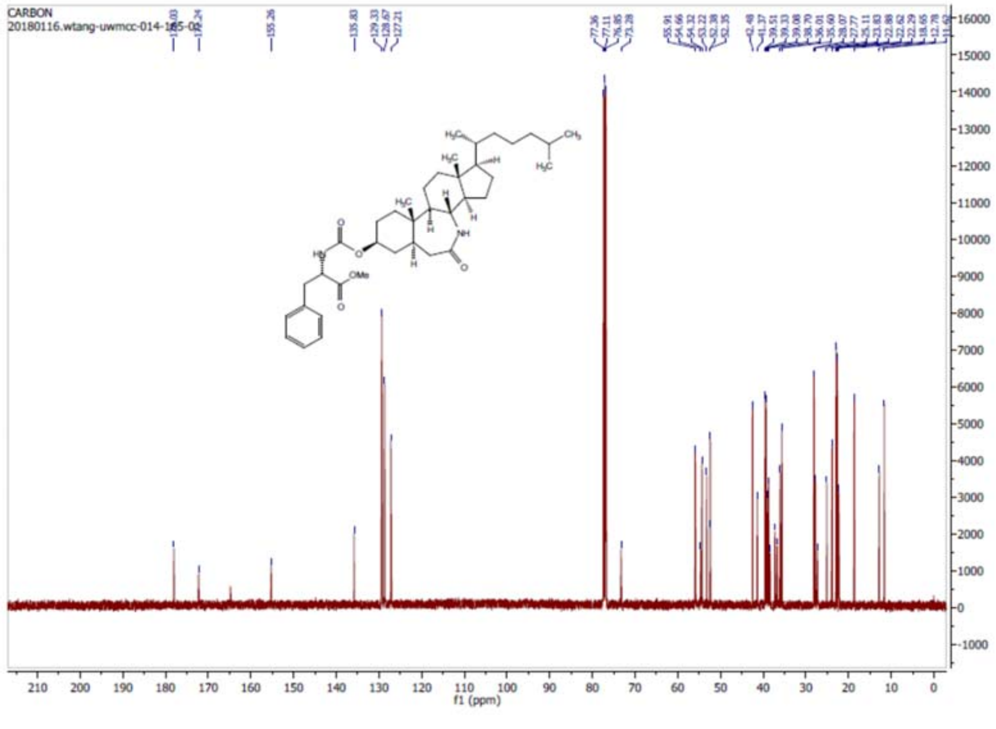

Supplementary Figure 127. ^{1}H and ^{13}C spectra of **41bk**.

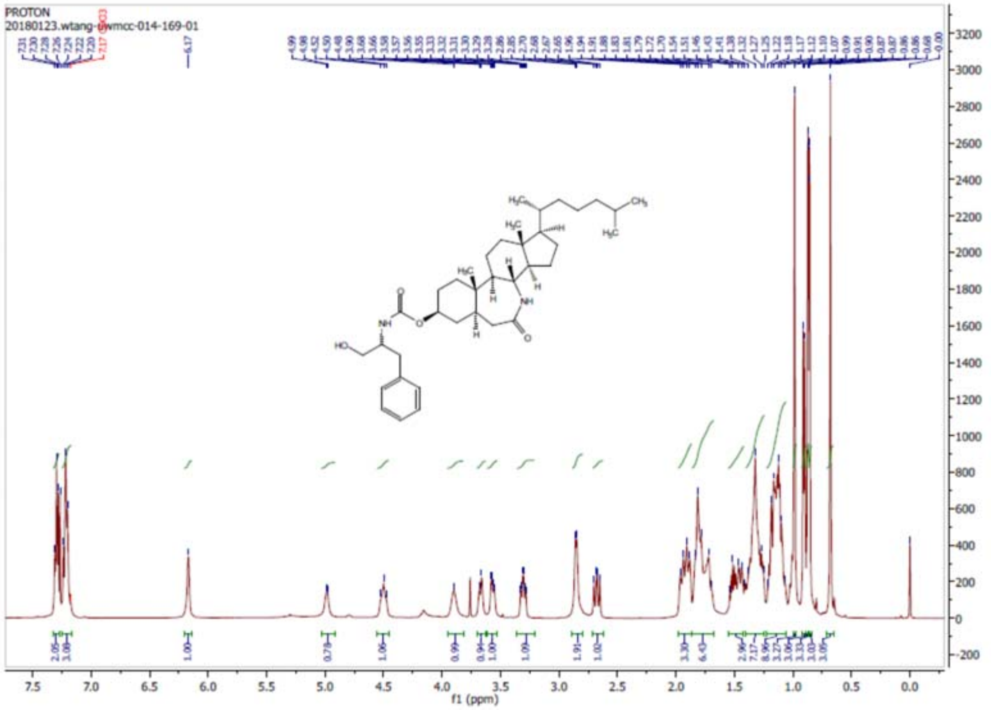

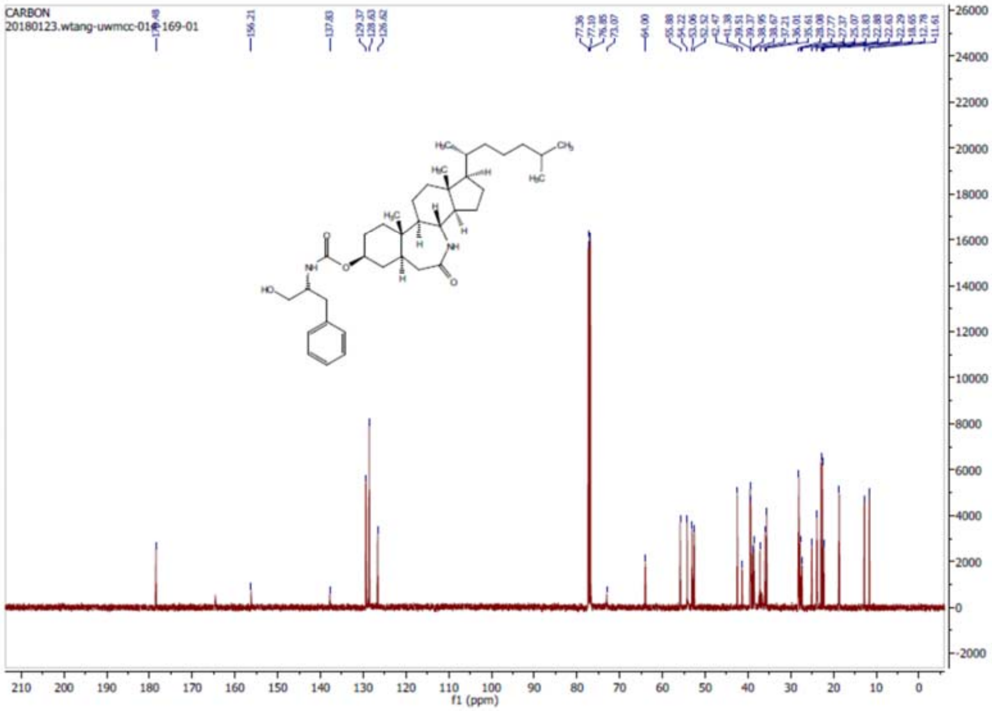

Supplementary Figure 128. ^{1}H and ^{13}C spectra of **41bl**.

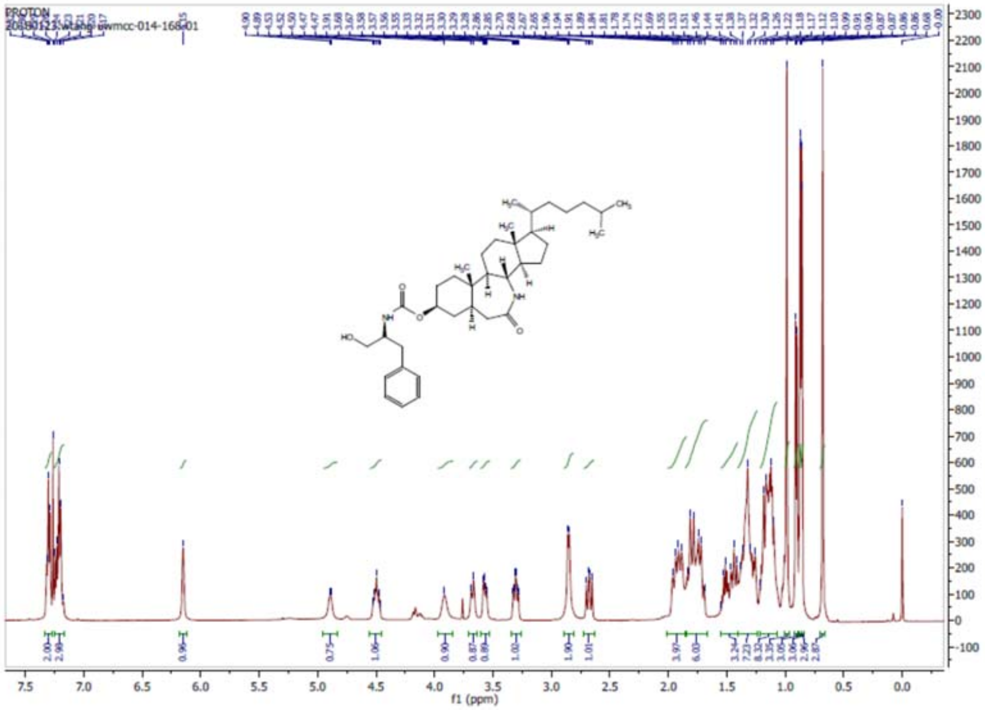

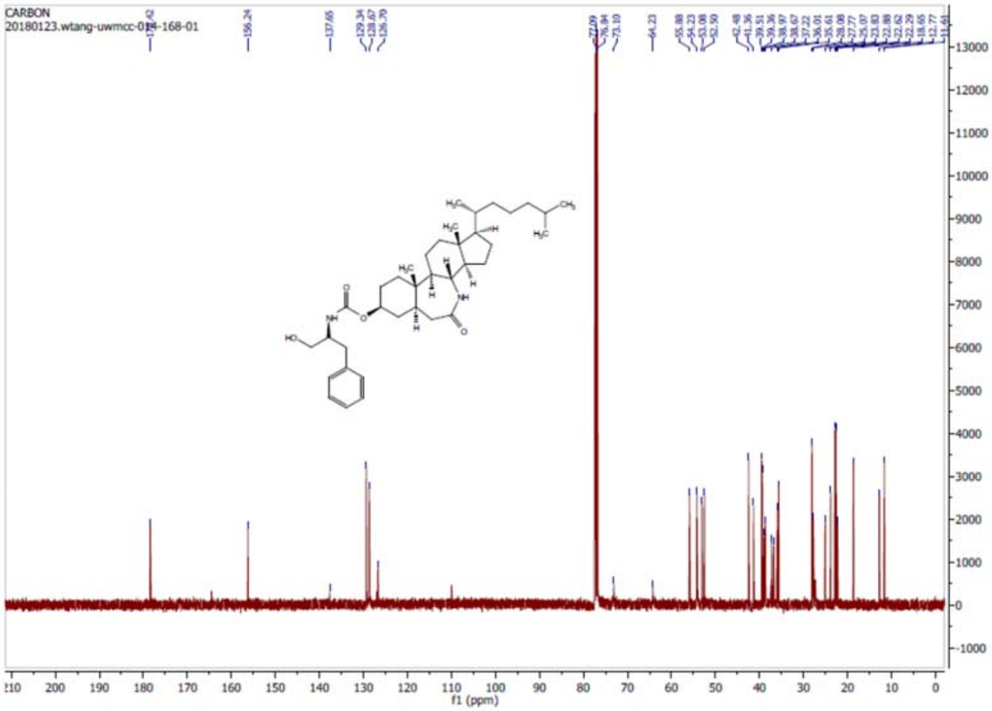

Supplementary Figure 129. ^{1}H and ^{13} C spectra of **41bm**.

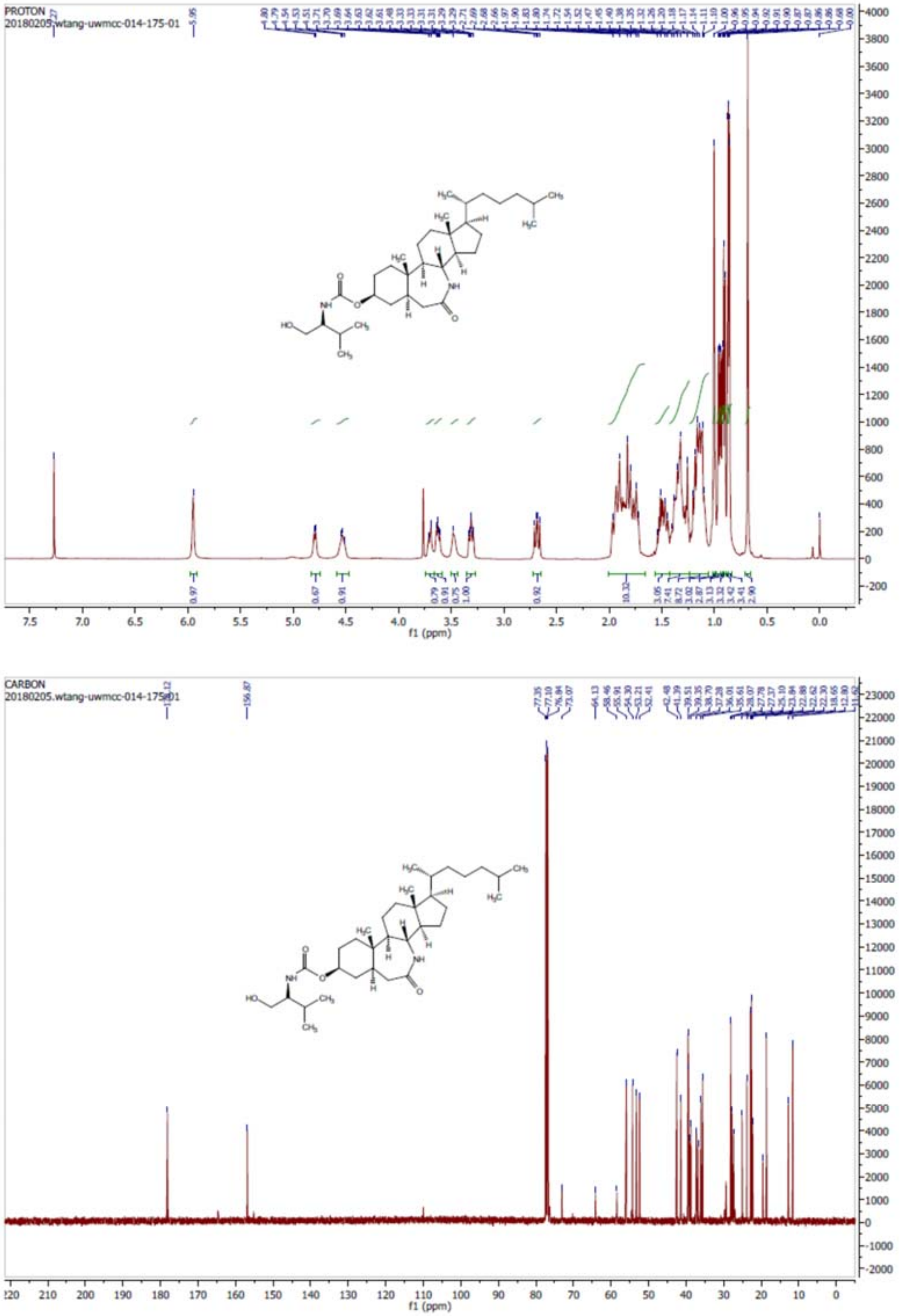

Supplementary Figure 130. ^{1}H and ^{13}C spectra of **41bn**.

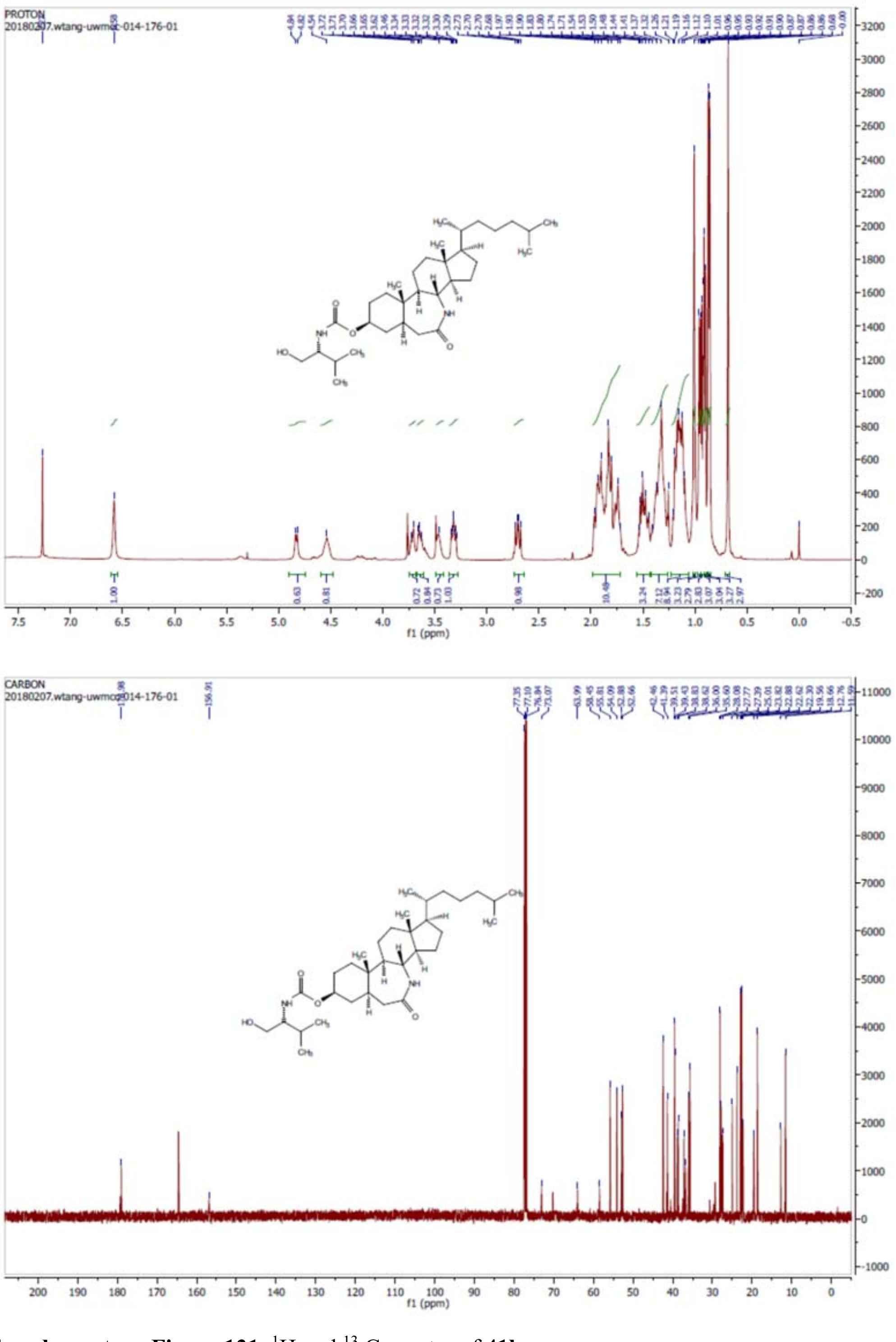

Supplementary Figure 131. ^{1}H and ^{13}C spectra of **41bo**.

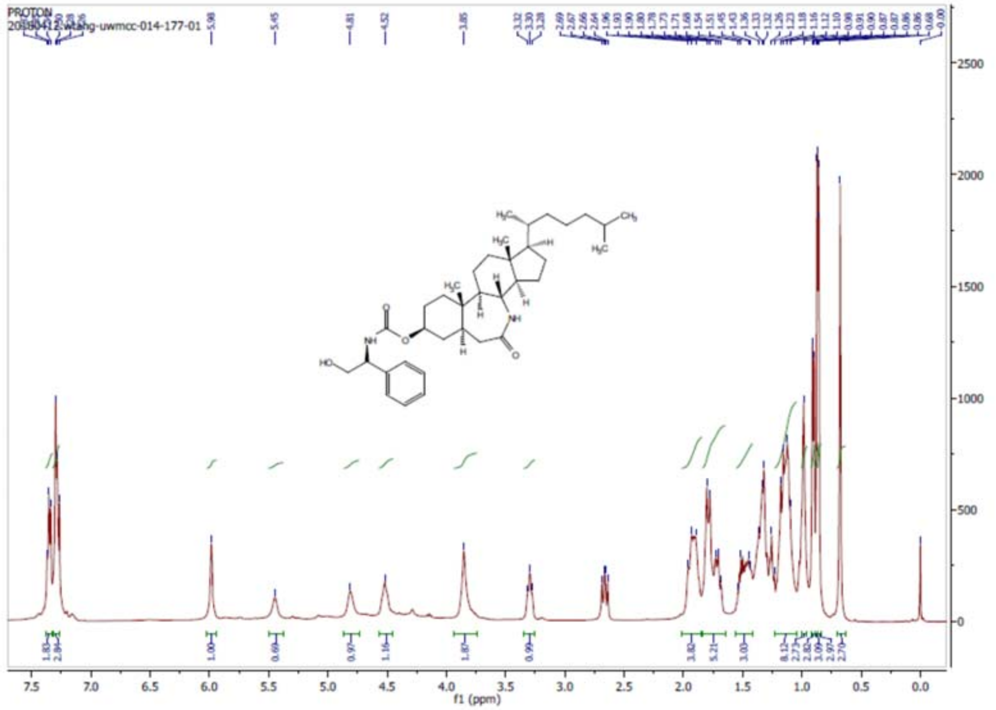

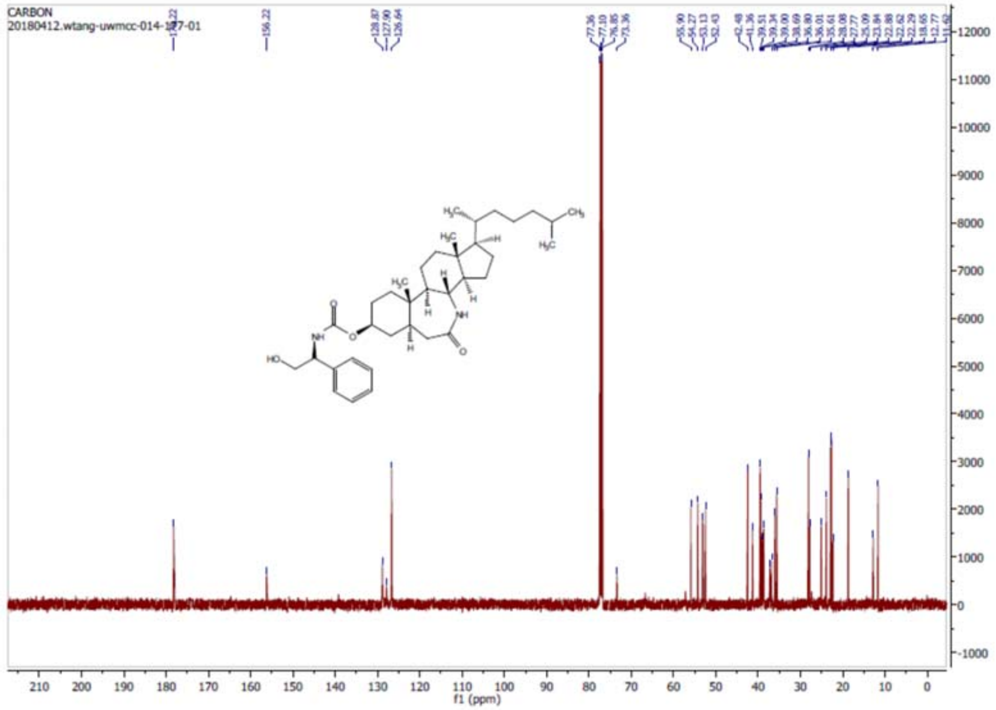

Supplementary Figure 132. ^{1}H and ^{13}C spectra of **41bp**.

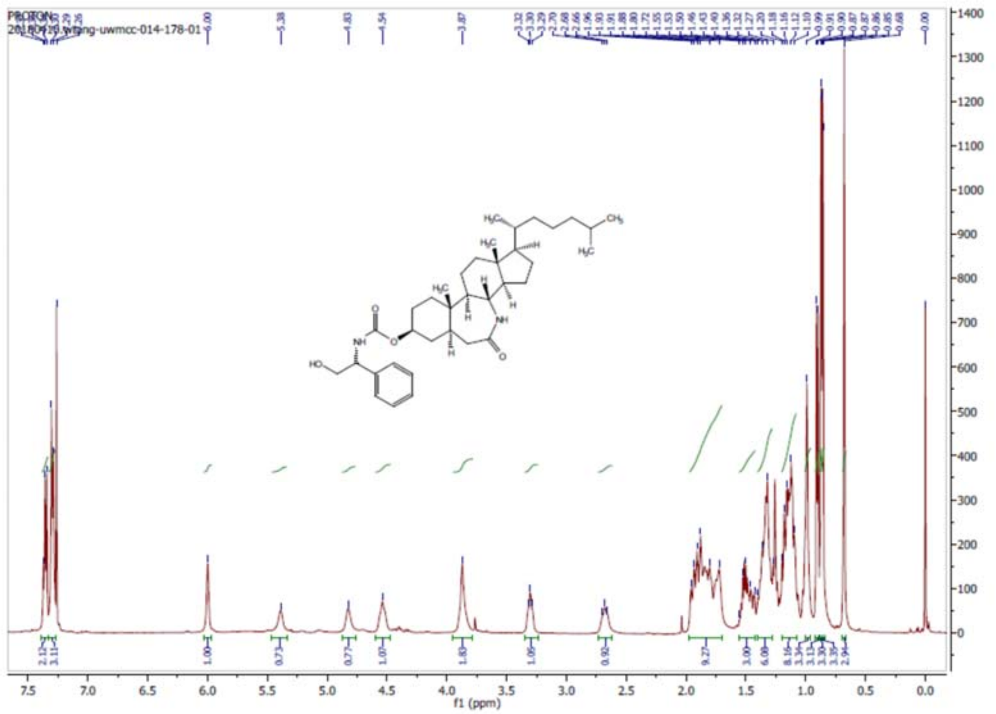

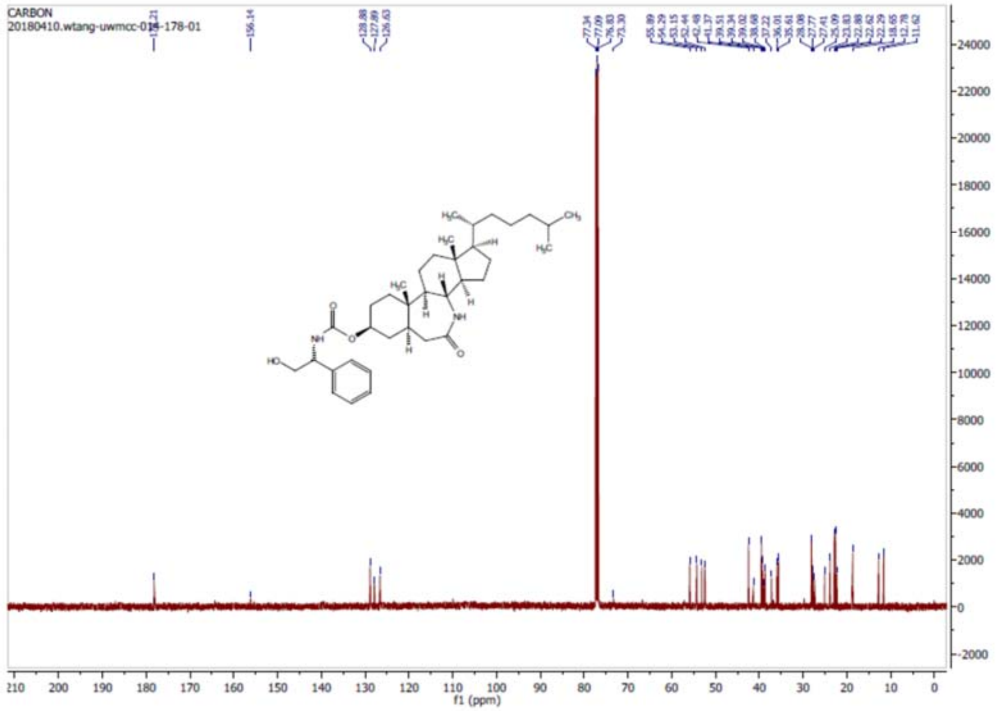

Supplementary Figure 133. ^{1}H and ^{13}C spectra of **41bq**.

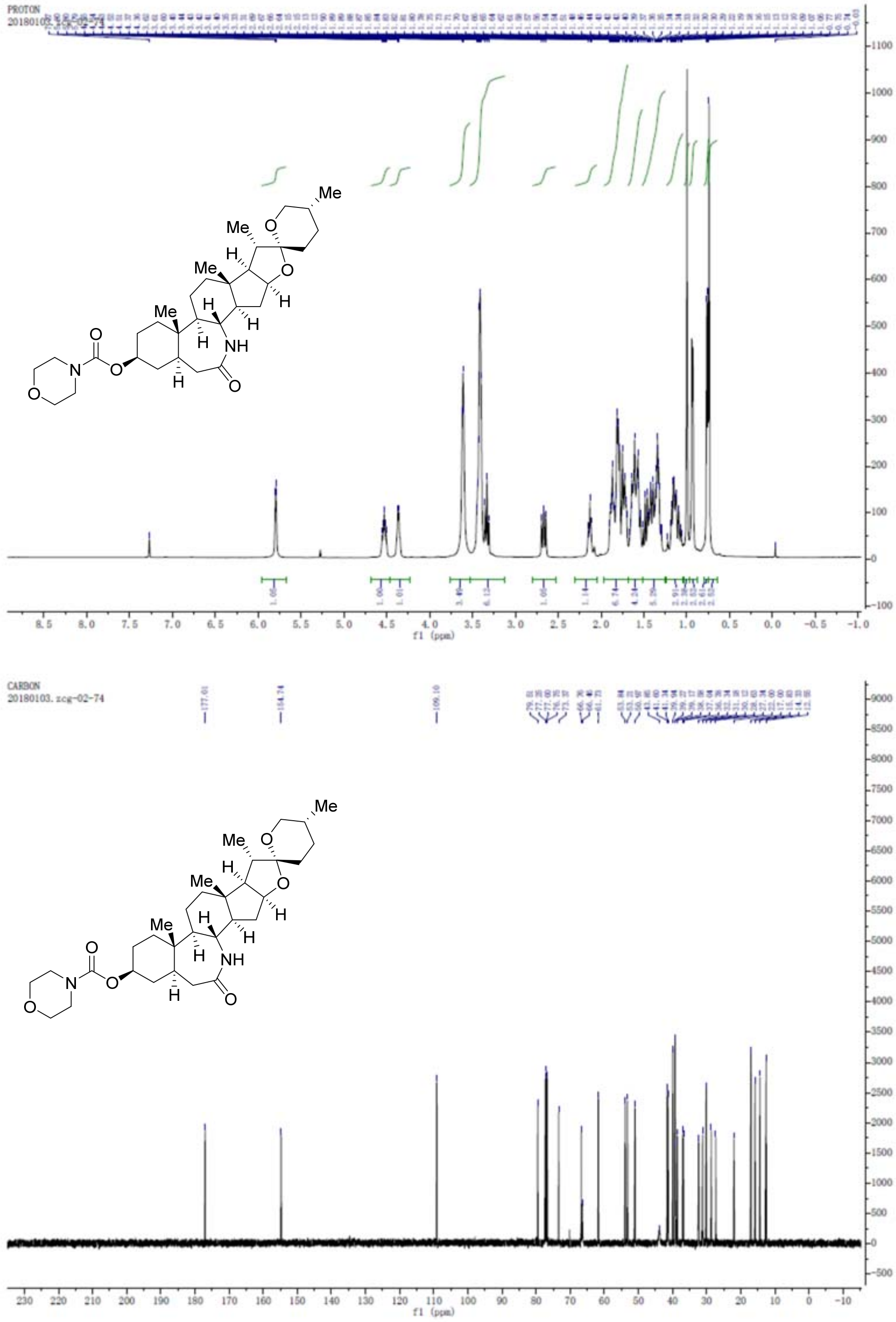

Supplementary Figure 134. ^{1}H and ^{13} C spectra of **41ca**.

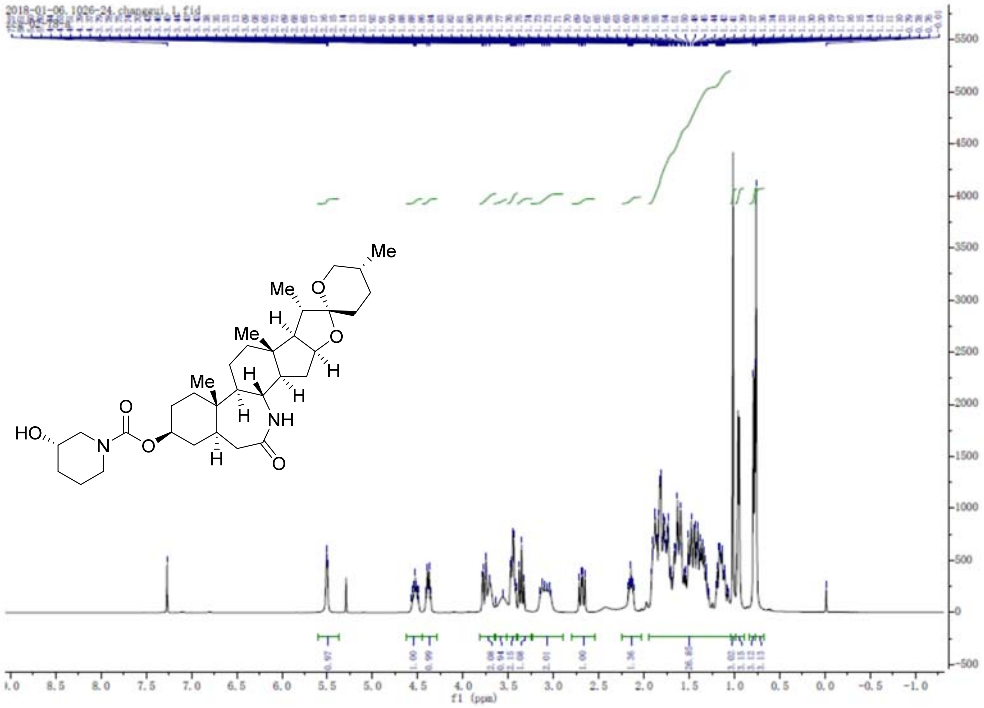

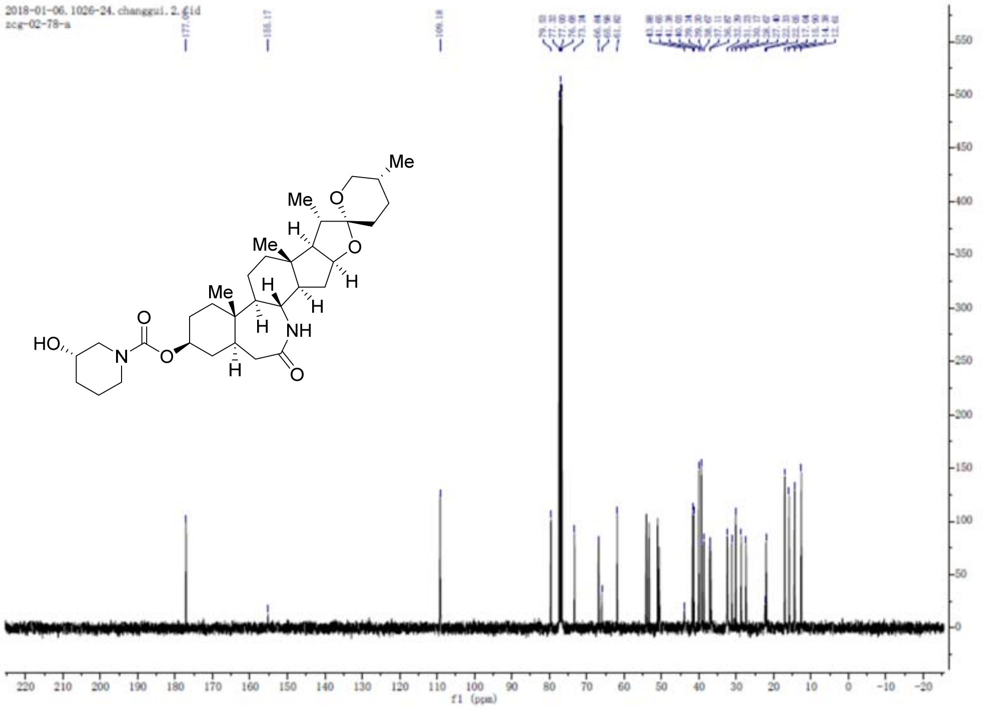

Supplementary Figure 135. ^{1}H and ^{13}C spectra of **41cb**.

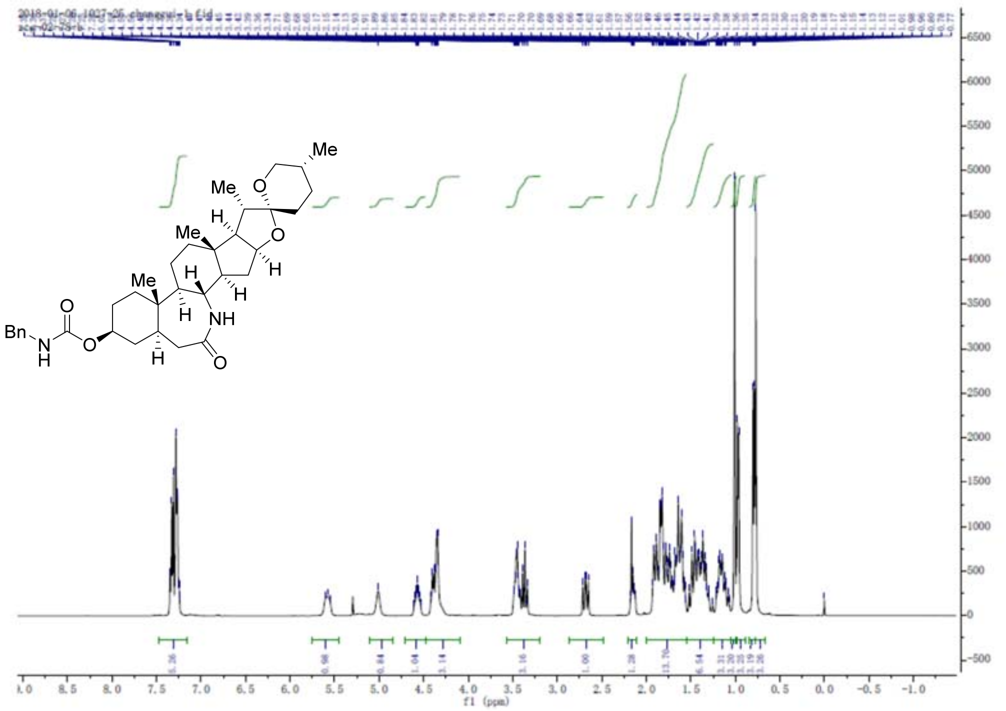

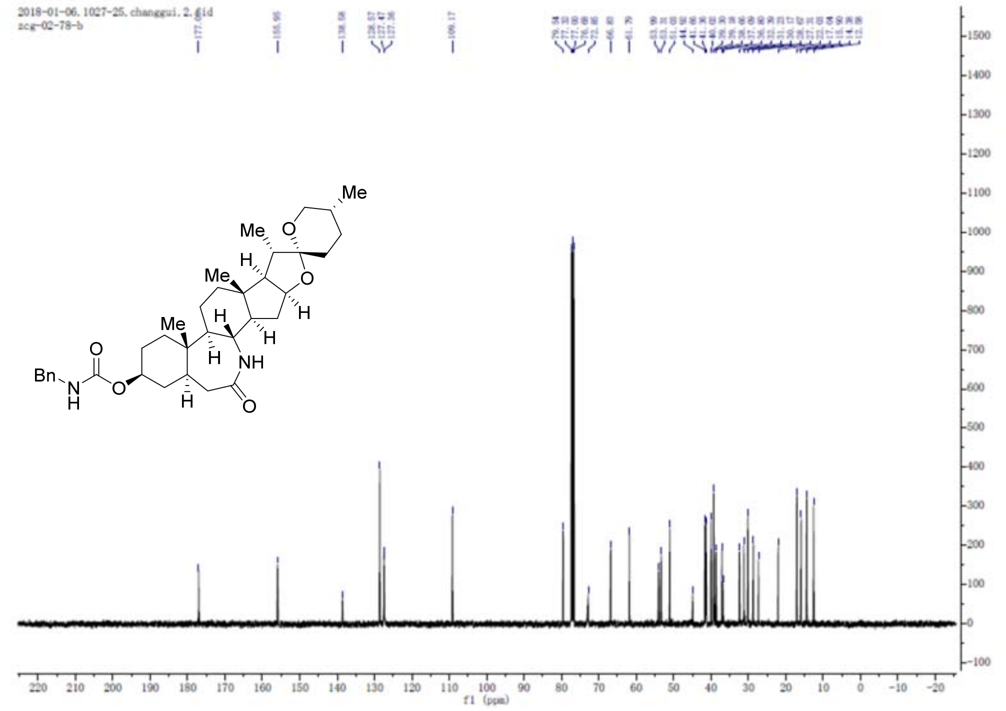

Supplementary Figure 136. ^{1}H and ^{13}C spectra of **41cc**.

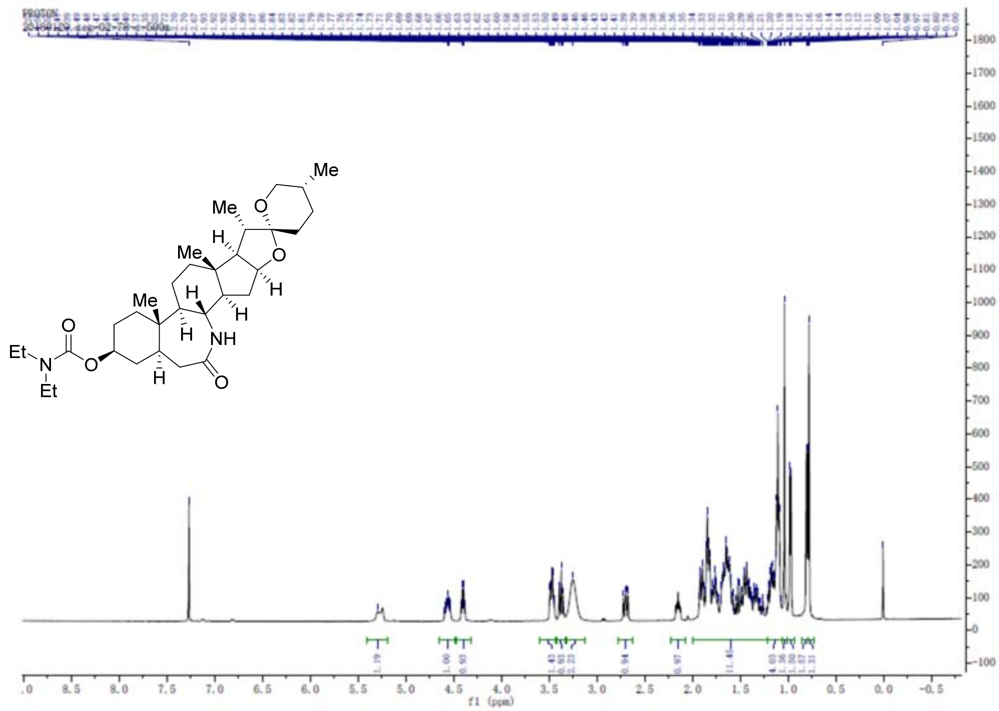

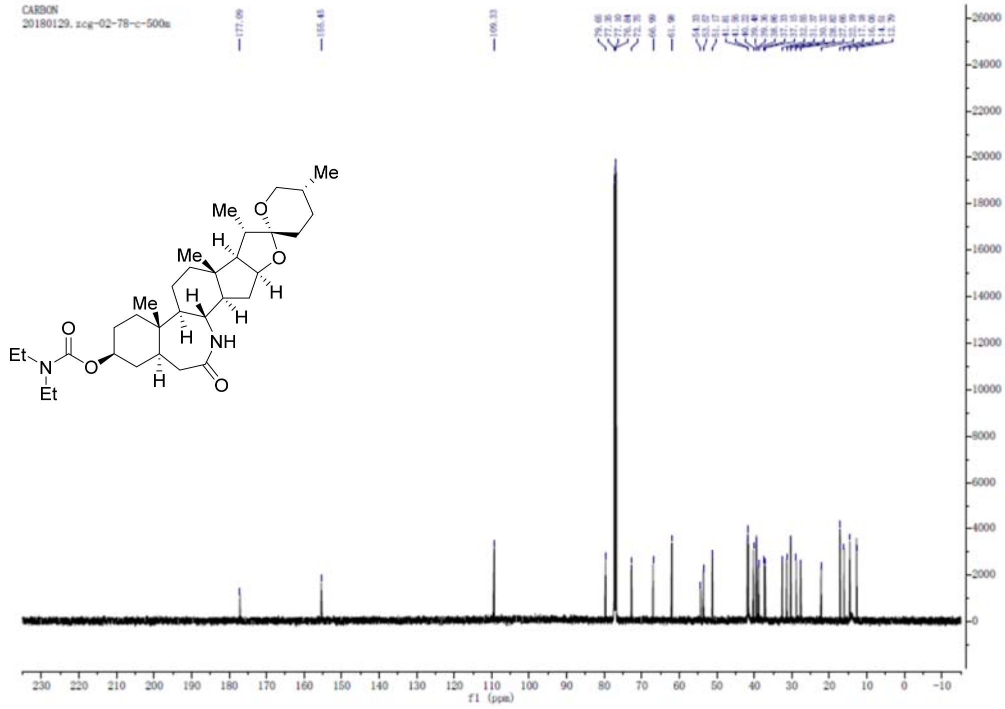

Supplementary Figure 137. ^{1}H and ^{13}C spectra of **41cd**.

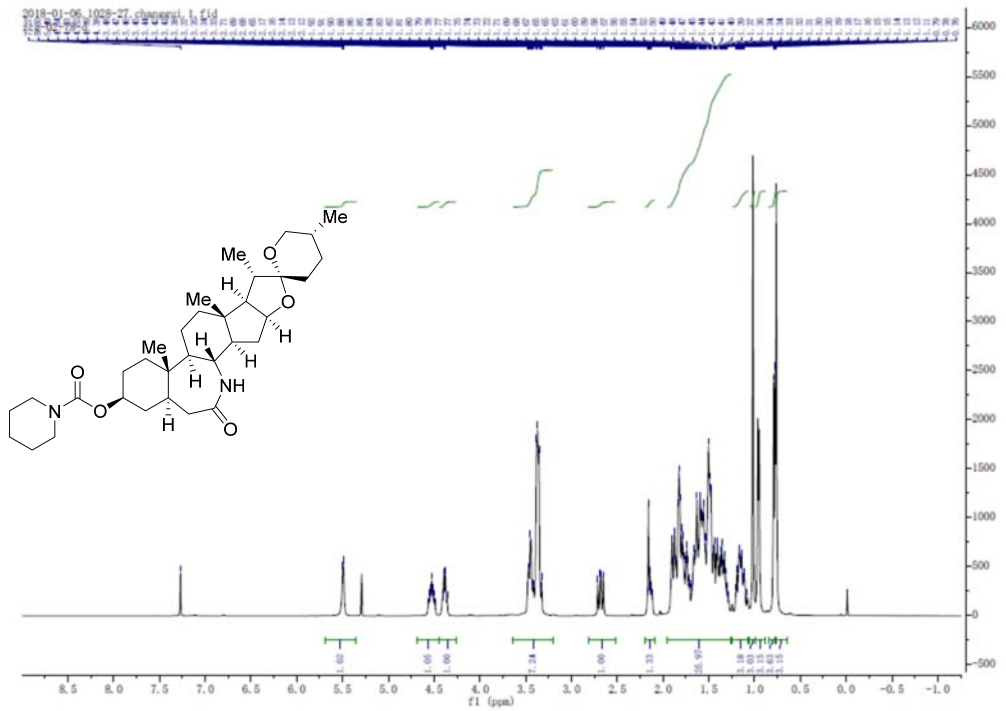

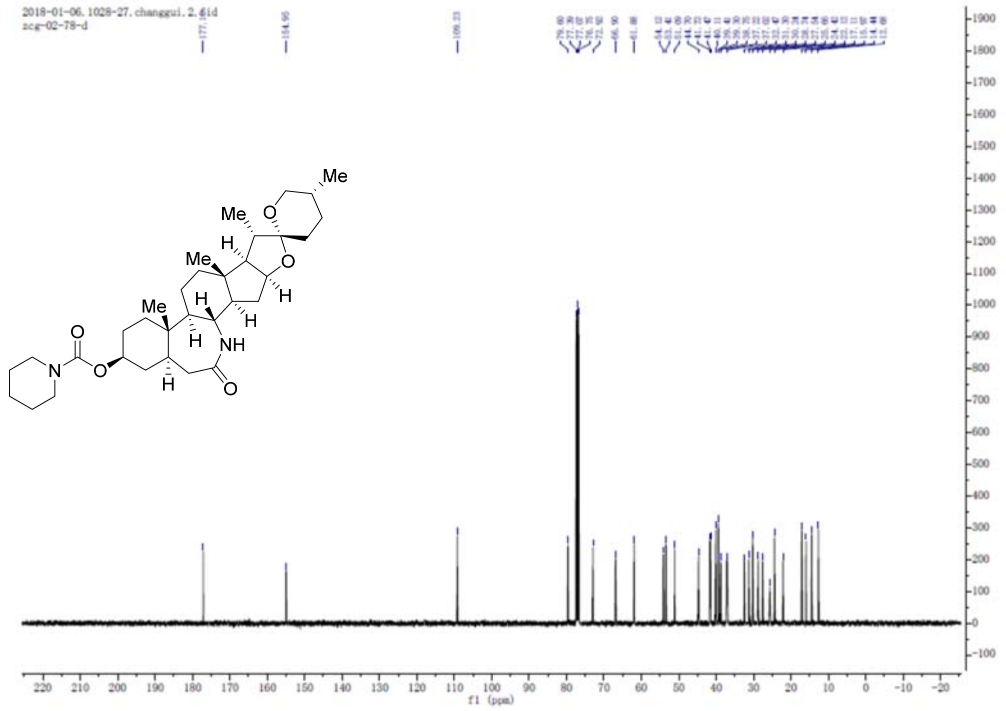

Supplementary Figure 138. ^{1}H and ^{13} C spectra of **41ce**.

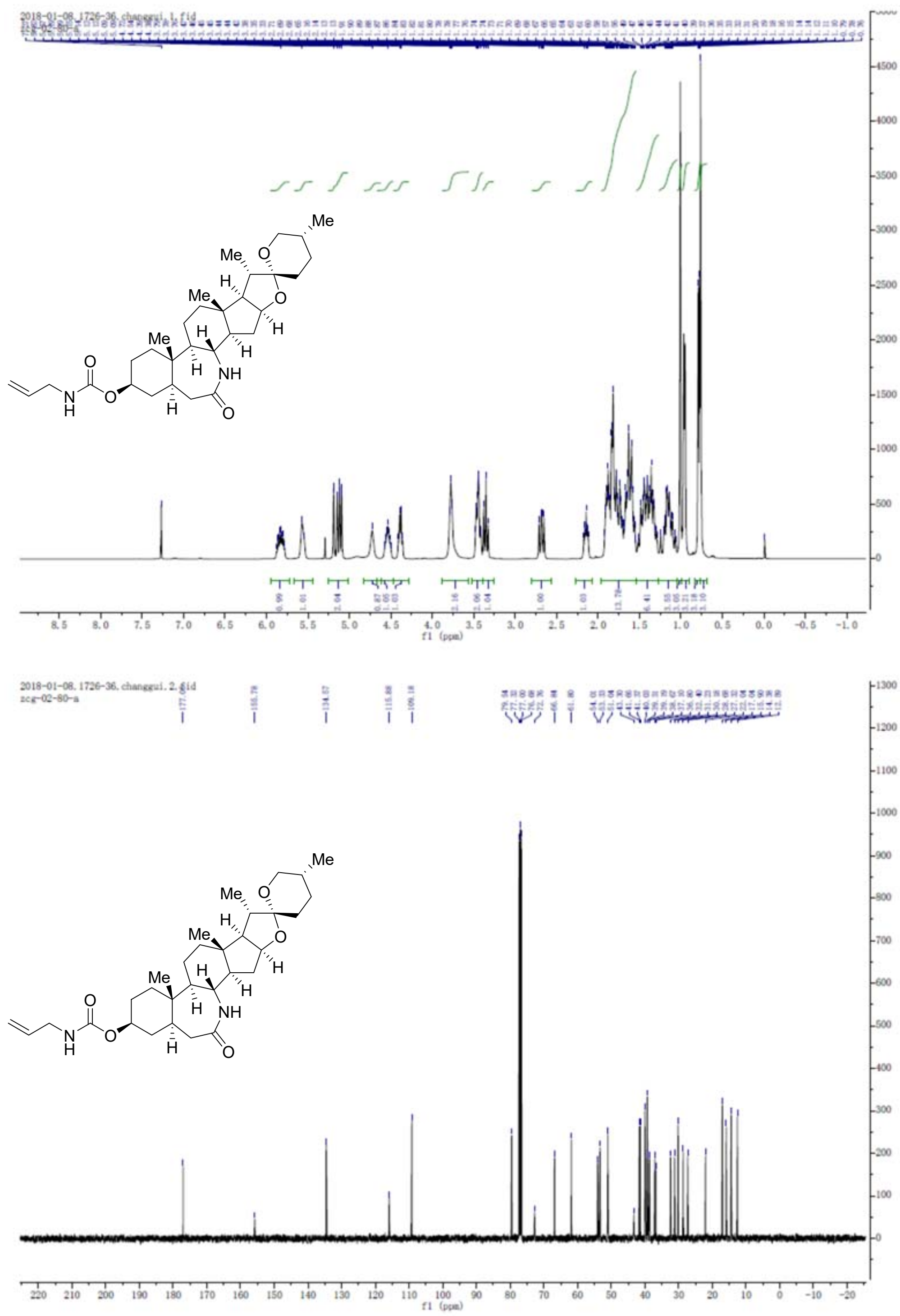

Supplementary Figure 139. ^{1}H and ^{13}C spectra of **41cf**.

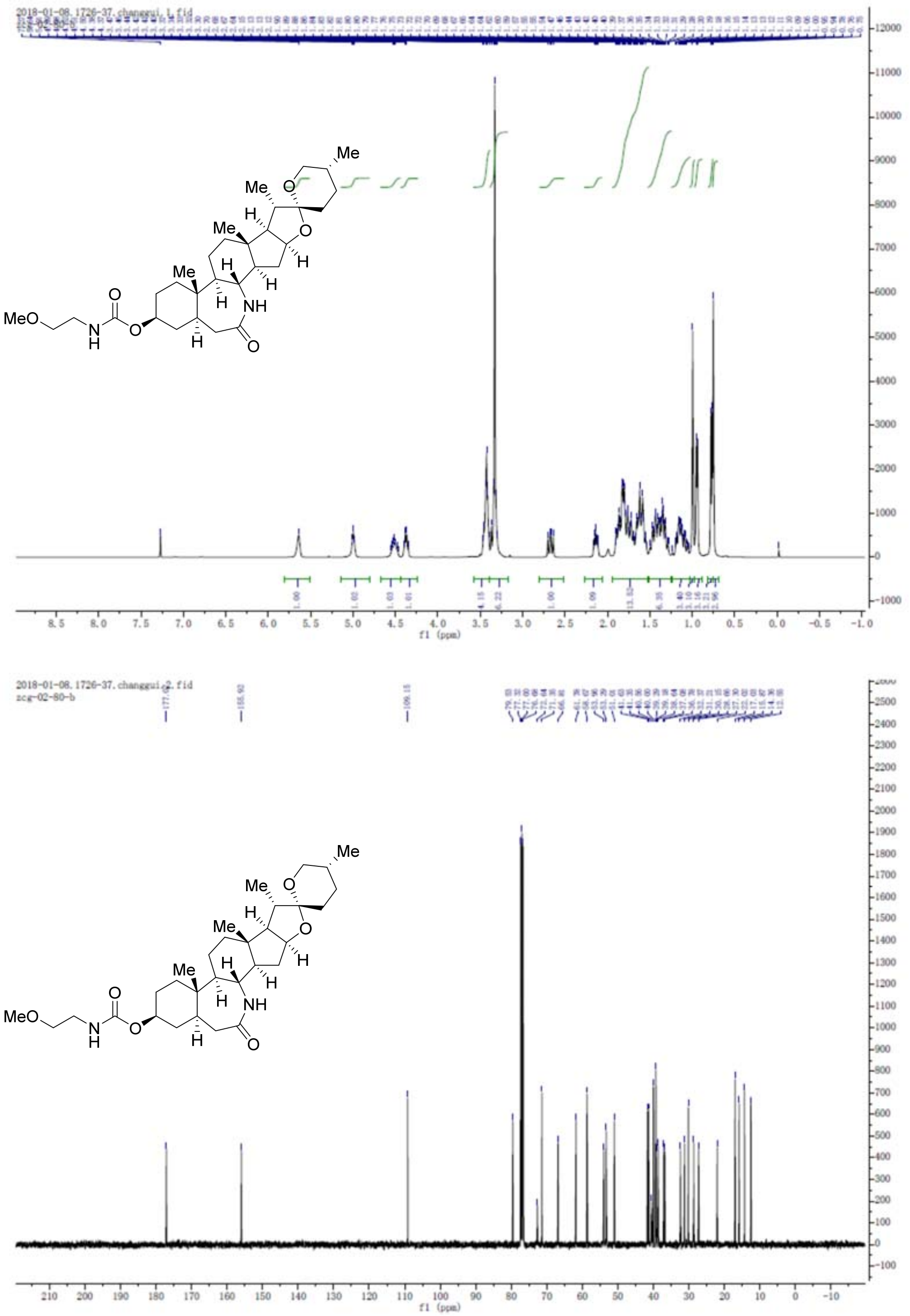

Supplementary Figure 140. ^{1}H and ^{13} C spectra of **41cg**.

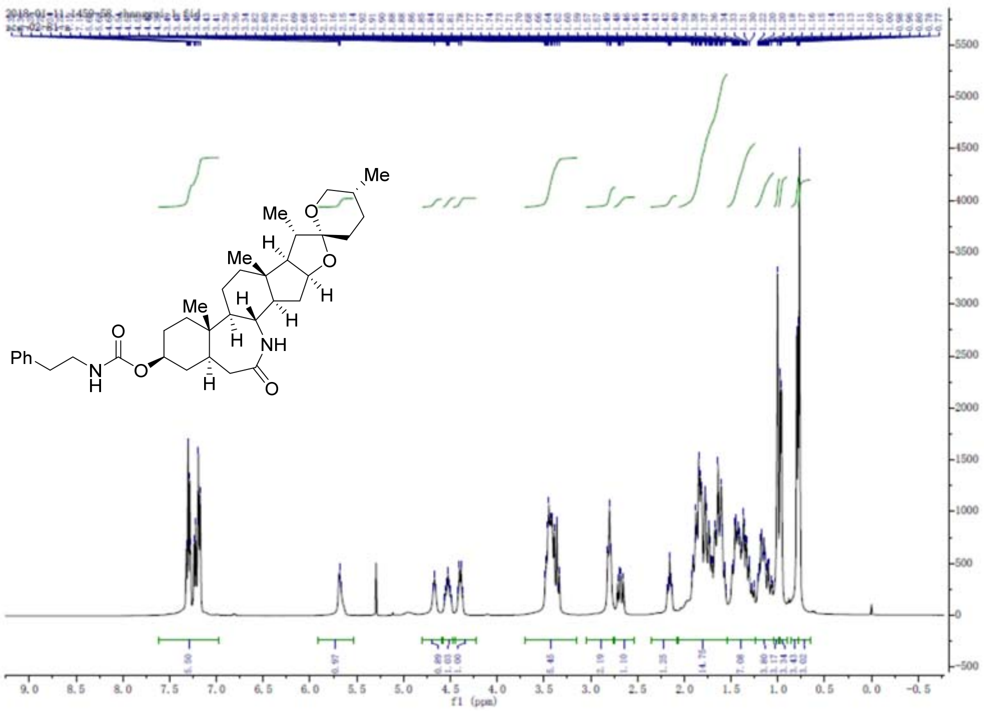

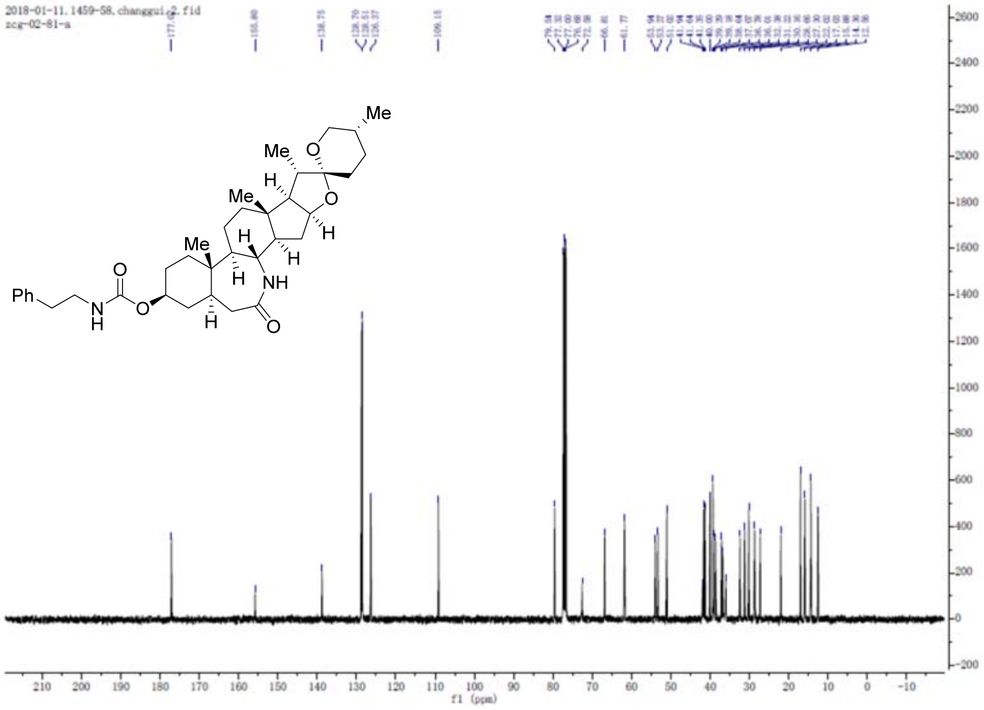

Supplementary Figure 141. ^{1}H and ^{13}C spectra of **41ch**.

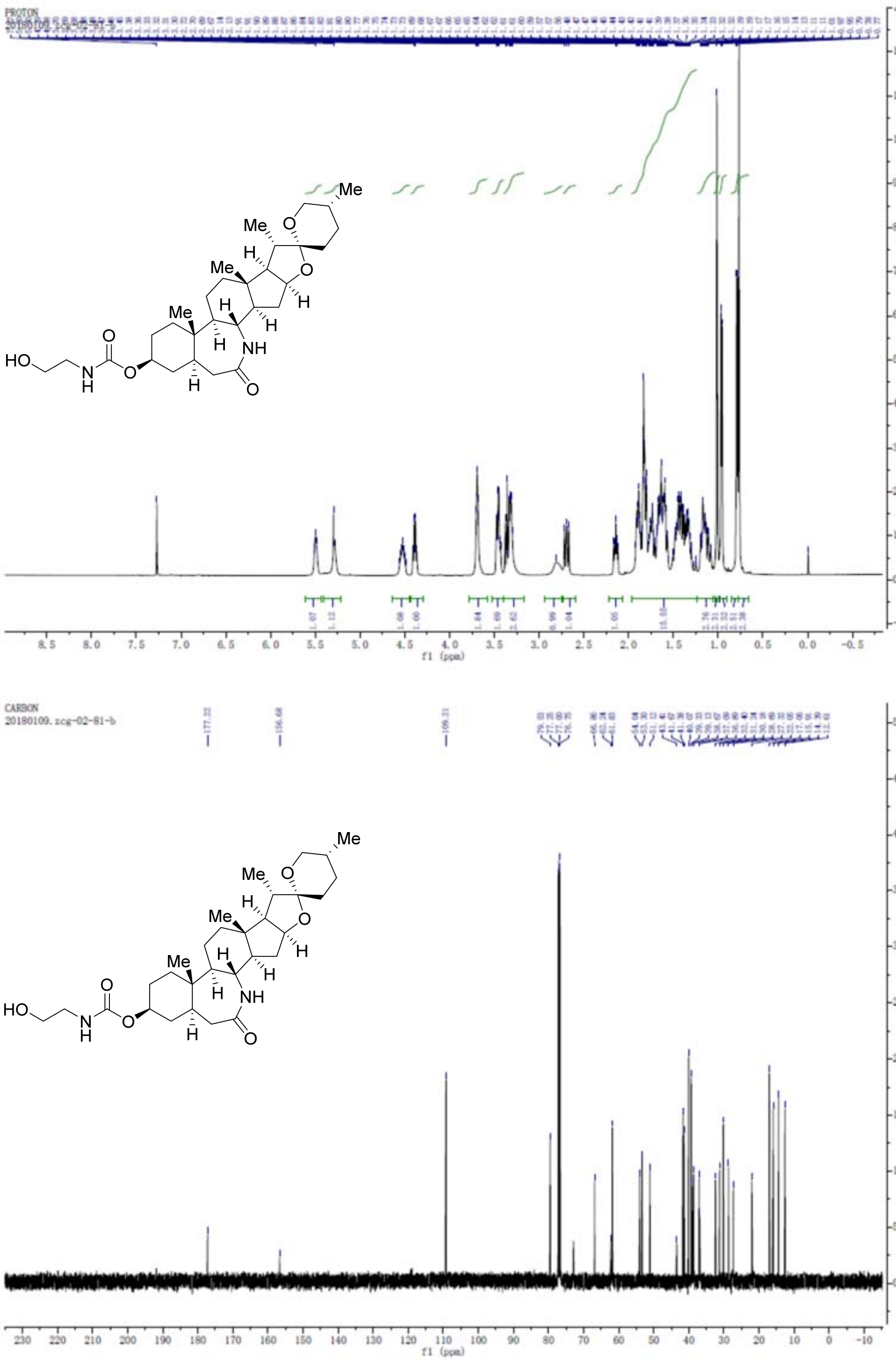

Supplementary Figure 142. ^{1}H and ^{13}C spectra of **41ci**.

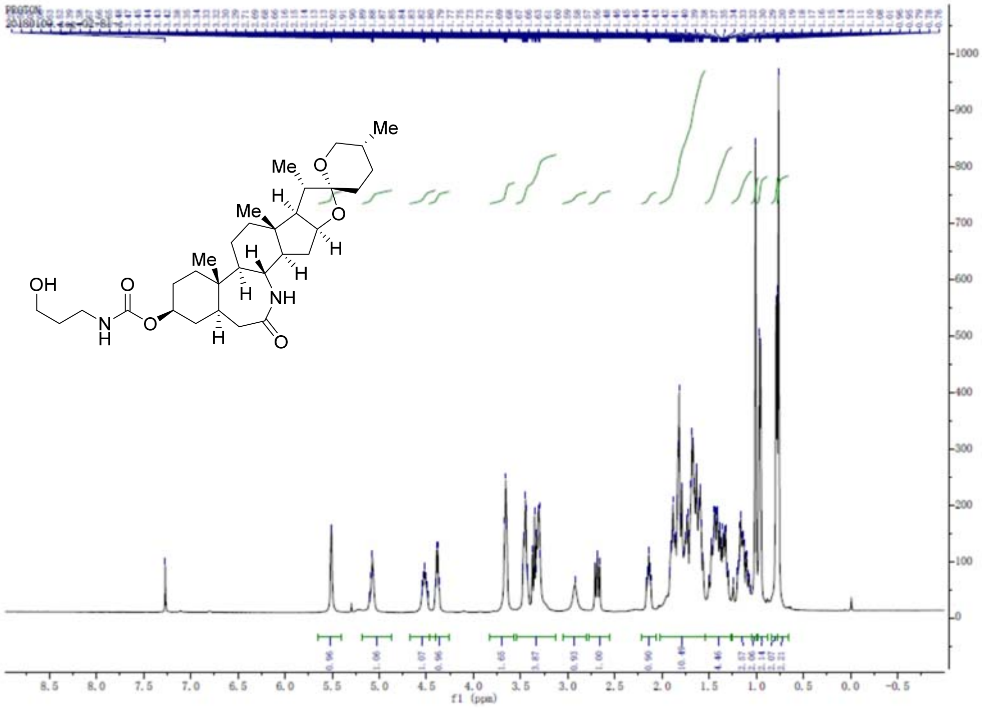

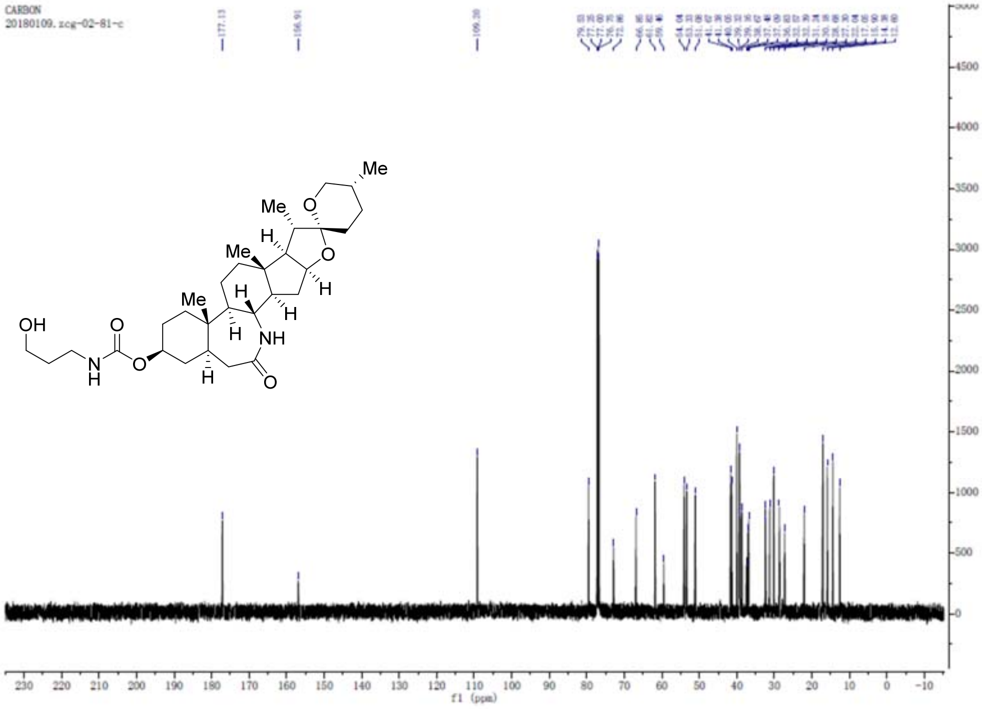

Supplementary Figure 143. ^{1}H and [13] C spectra of **41cj**.

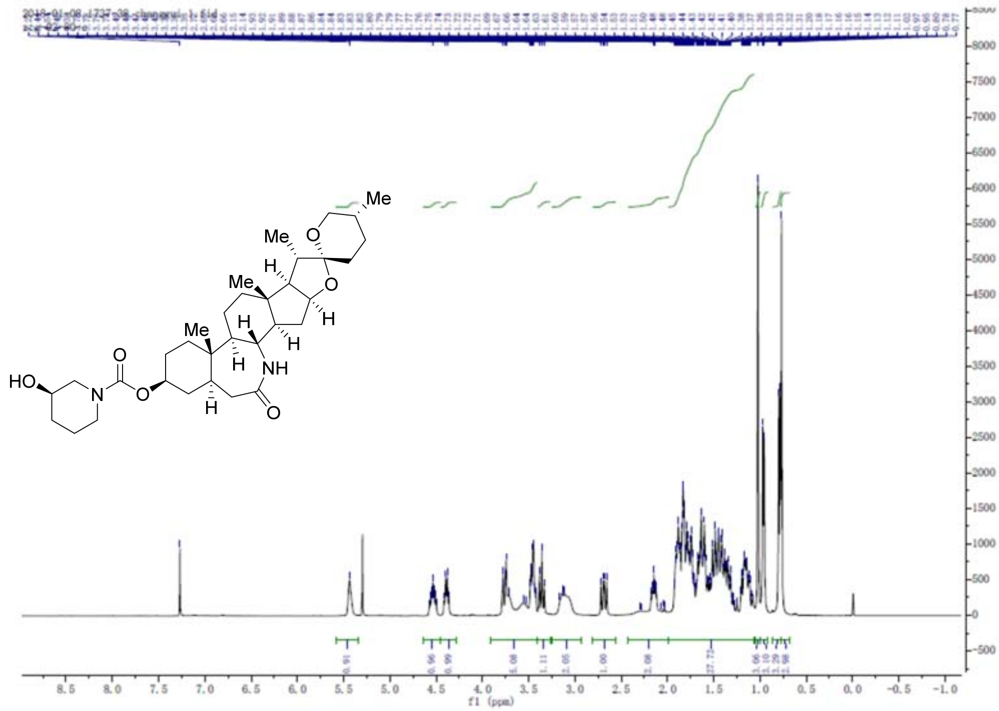

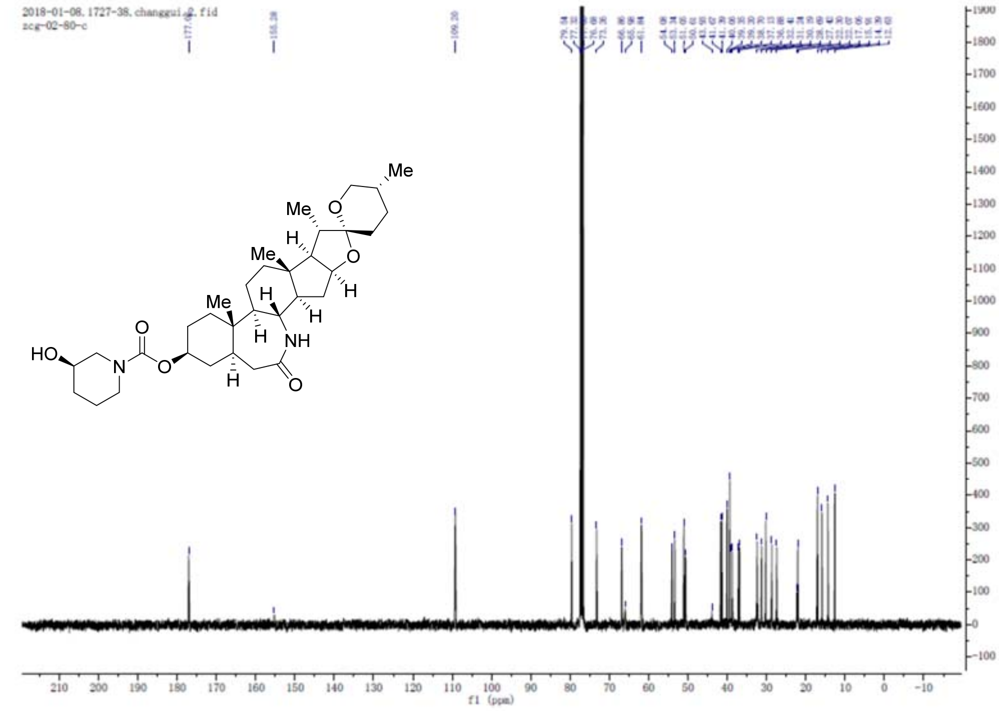

Supplementary Figure 144. ^{1}H and ^{13} C spectra of **41ck**.

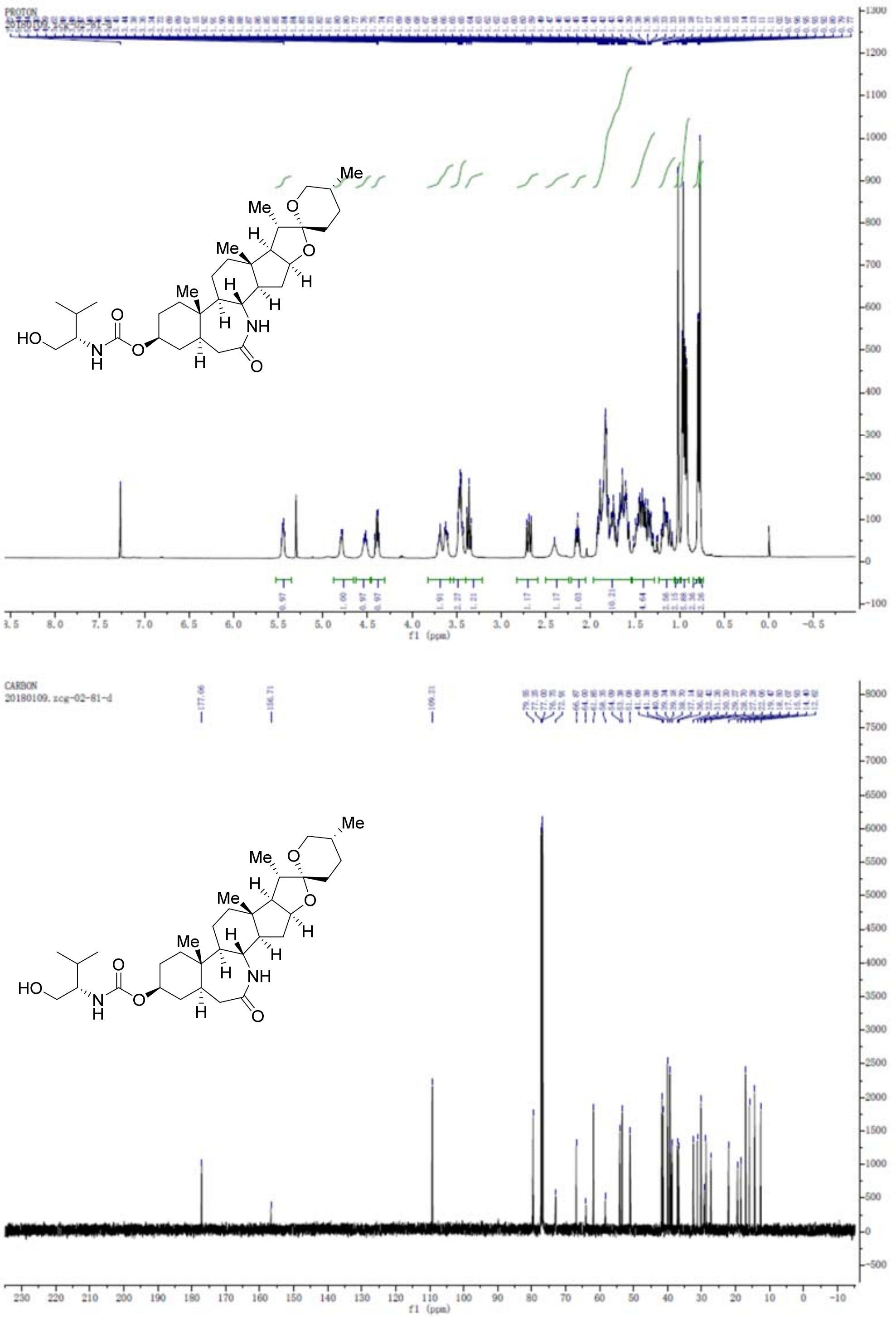

Supplementary Figure 145. ^{1}H and ^{13}C spectra of **41cl**.

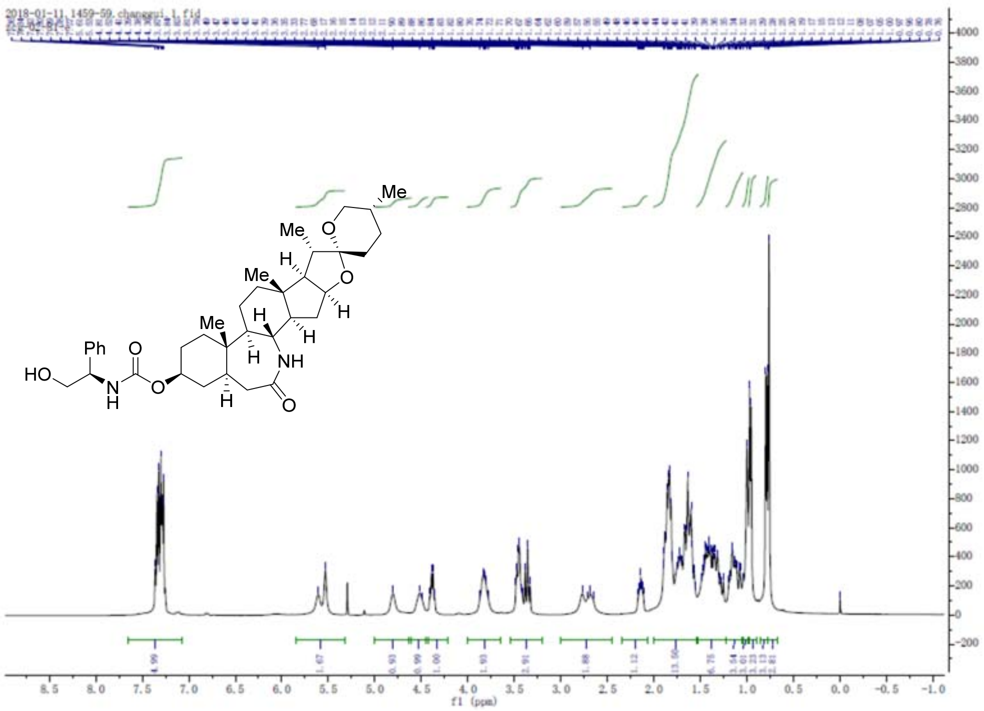

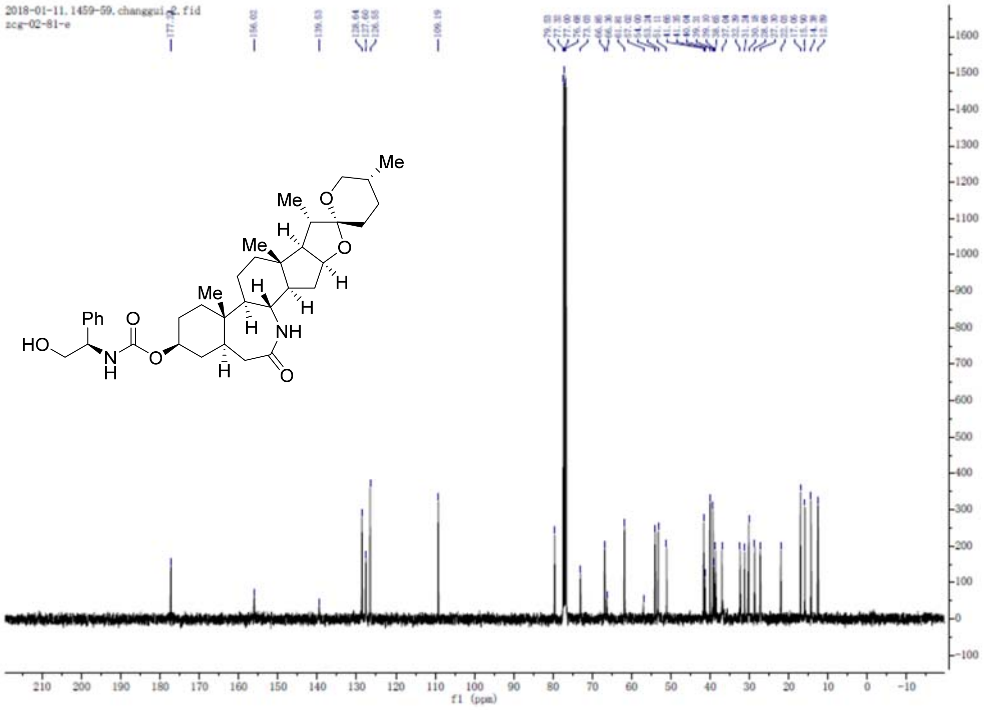

Supplementary Figure 146. ^{1}H and ^{13} C spectra of **41cm**.

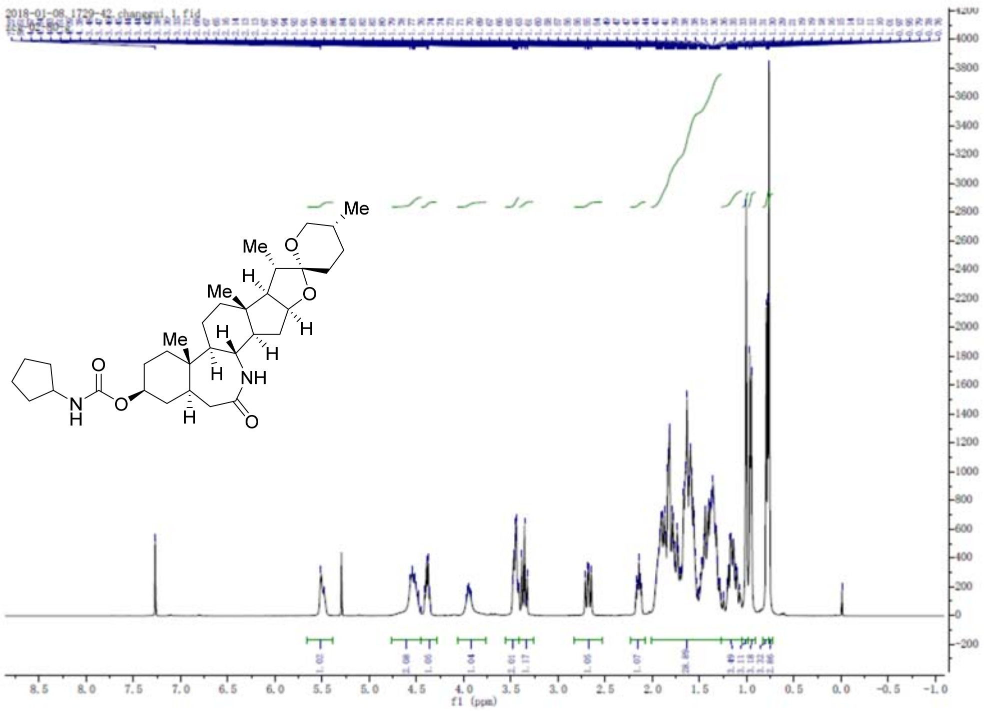

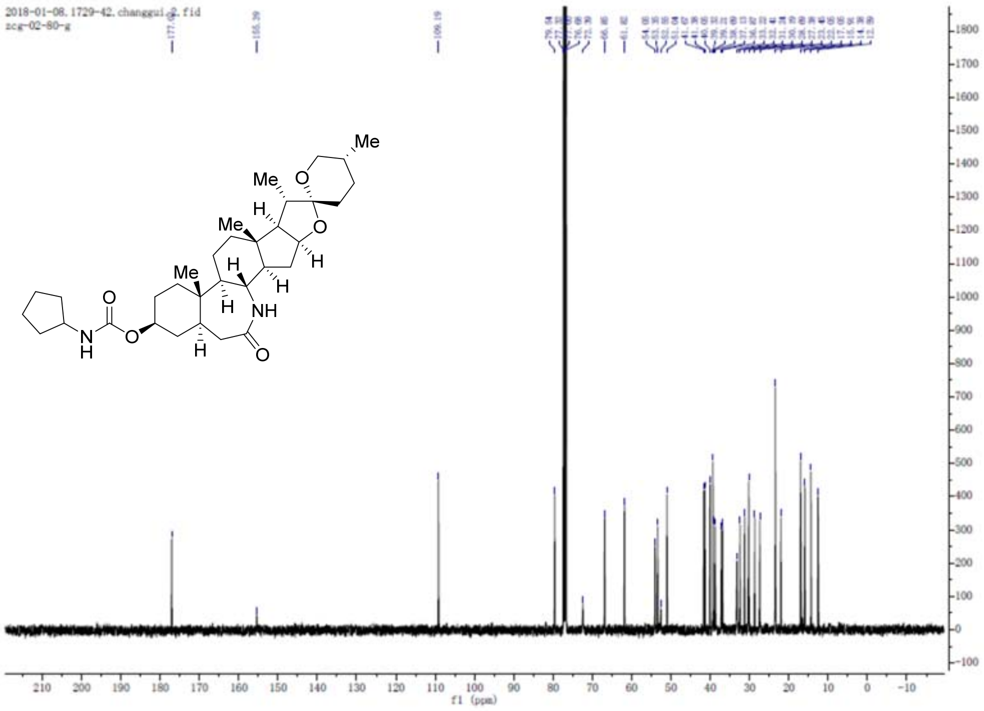

Supplementary Figure 147. ^{1}H and ^{13}C spectra of **41cn**.

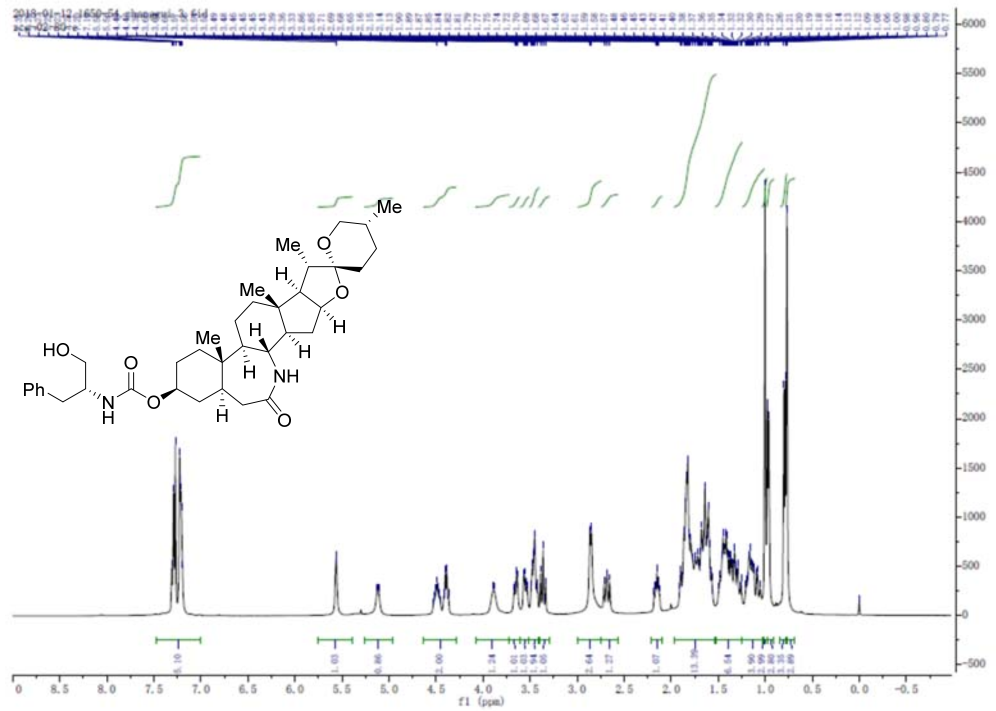

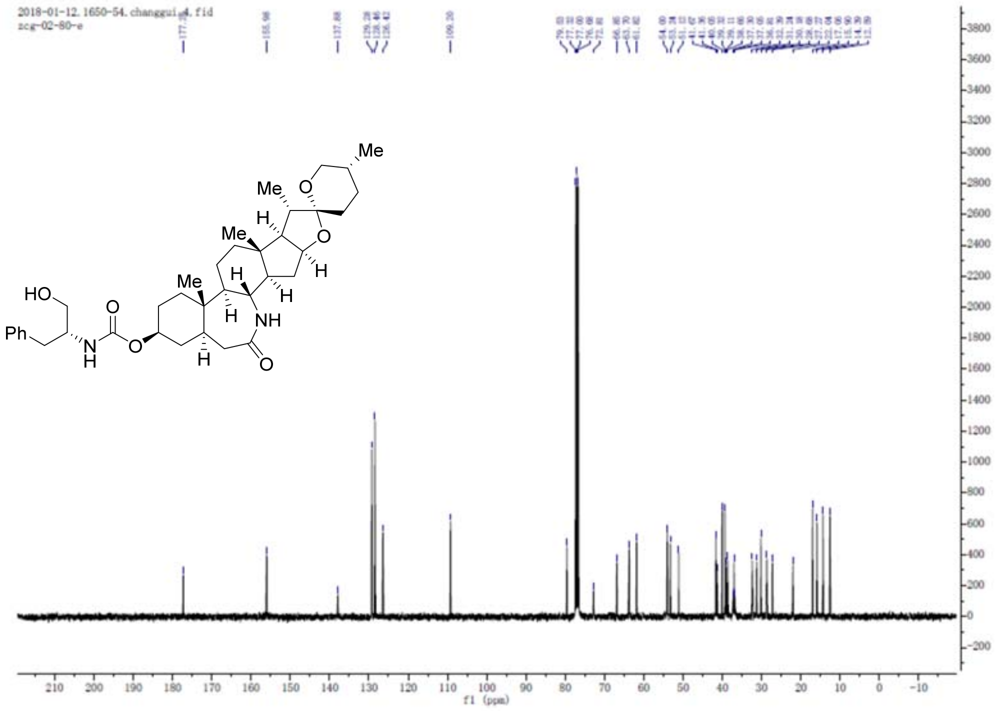

Supplementary Figure 148. ^{1}H and ^{13}C spectra of **41co**.

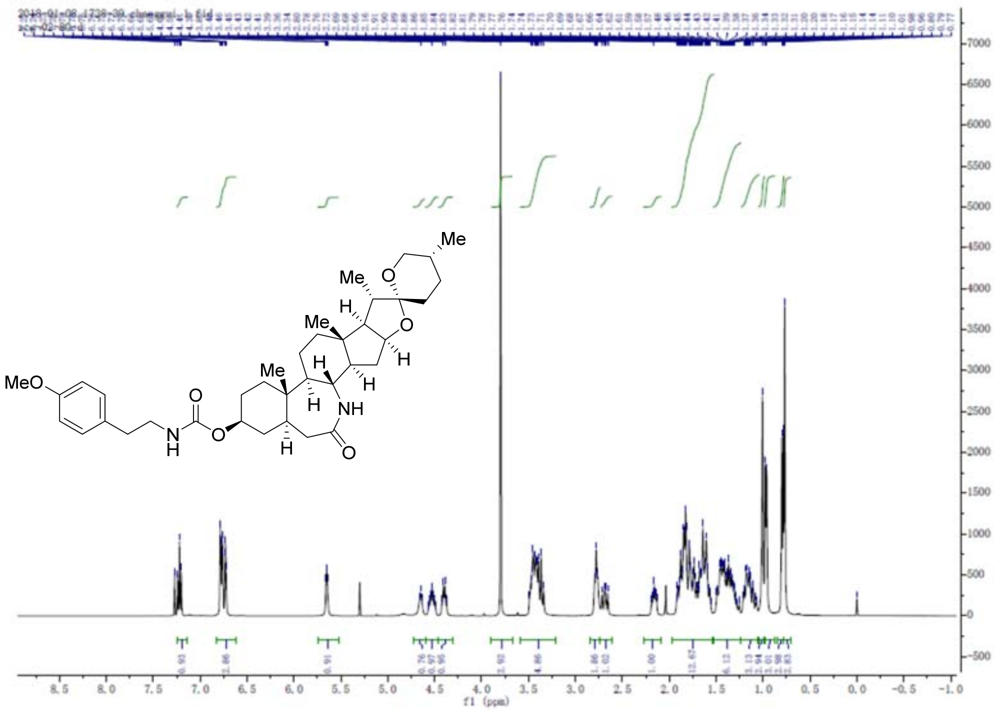

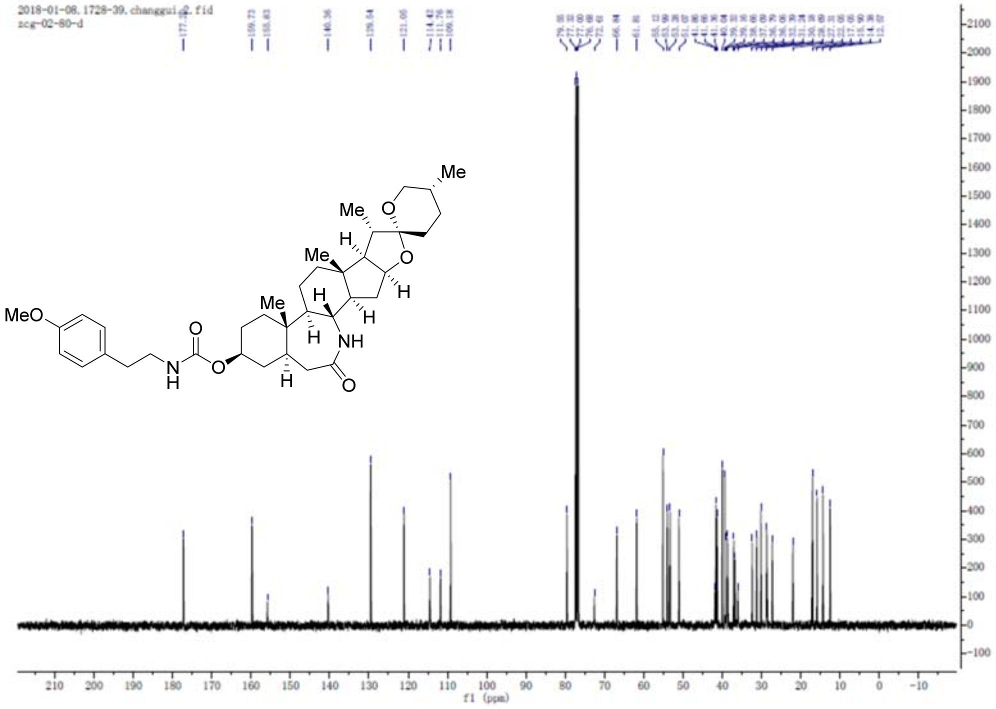

Supplementary Figure 149. ^{1}H and ^{13} C spectra of **41cp**.

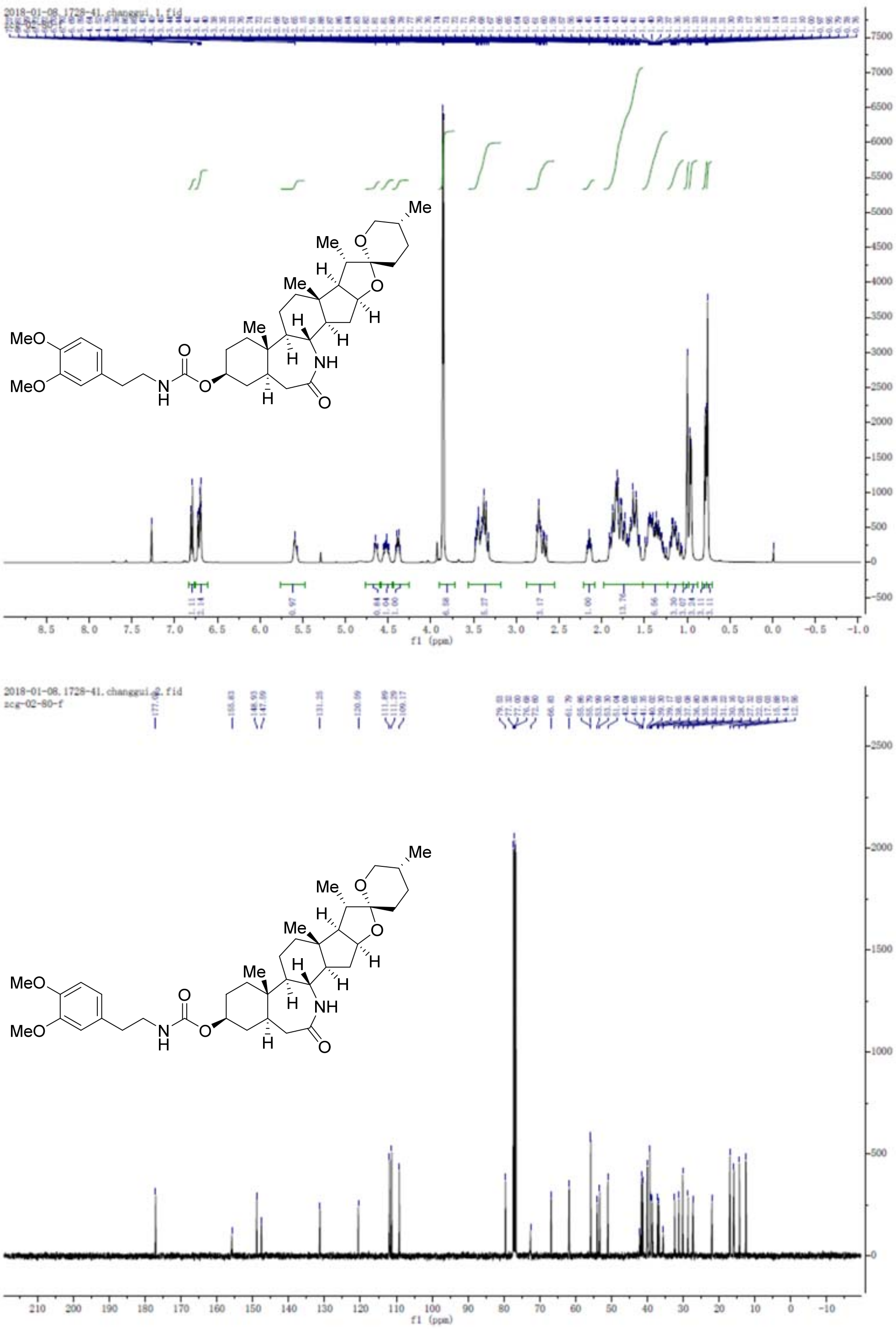

Supplementary Figure 150. ^{1}H and ^{13}C spectra of **41cq**.

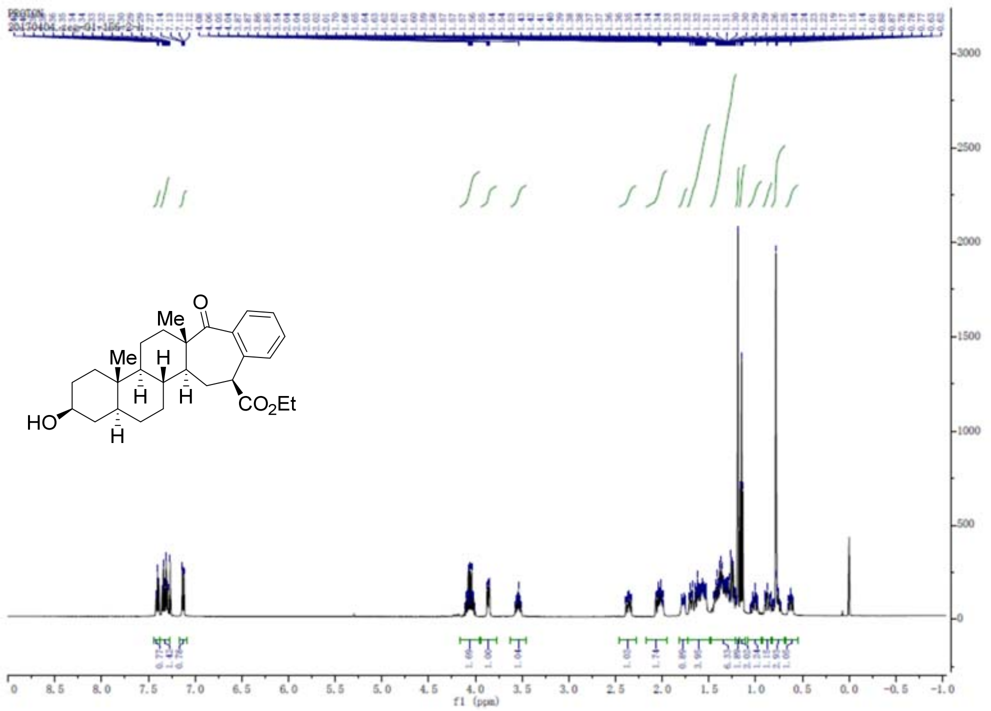

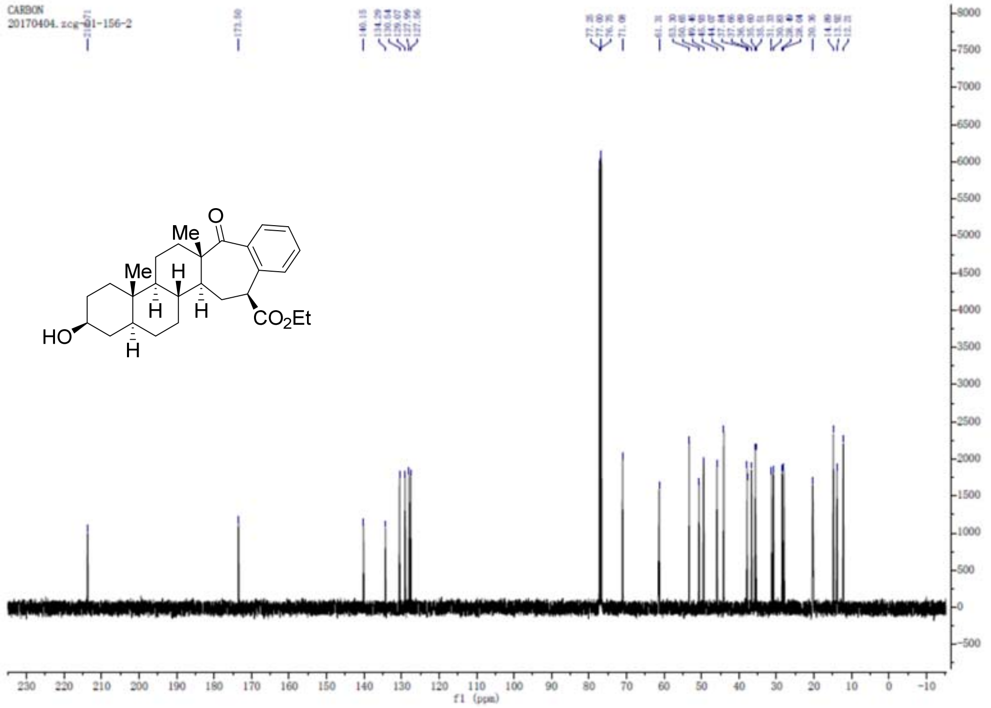

Supplementary Figure 151. ^{1}H and ^{13}C spectra of **42a**.

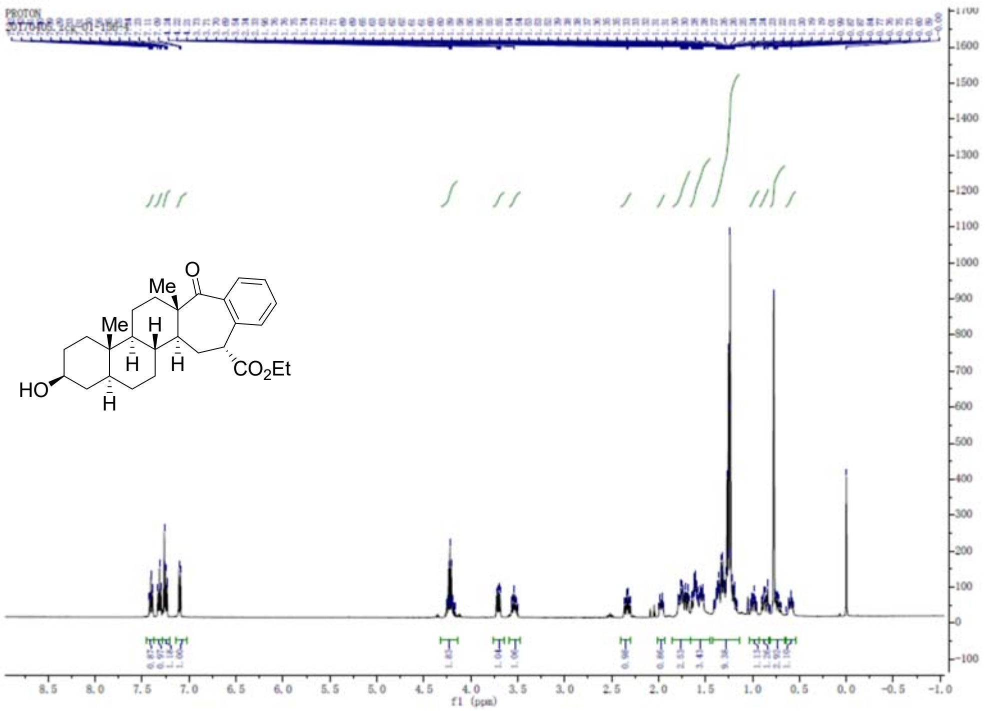

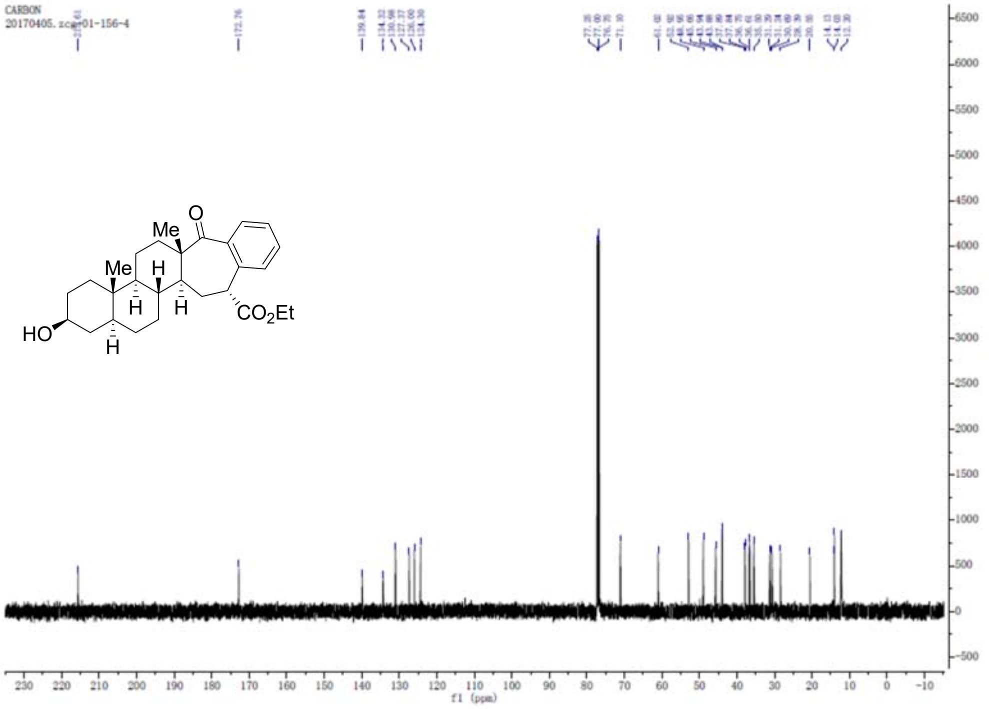

Supplementary Figure 152. ^{1}H and ^{13}C spectra of **42b**.

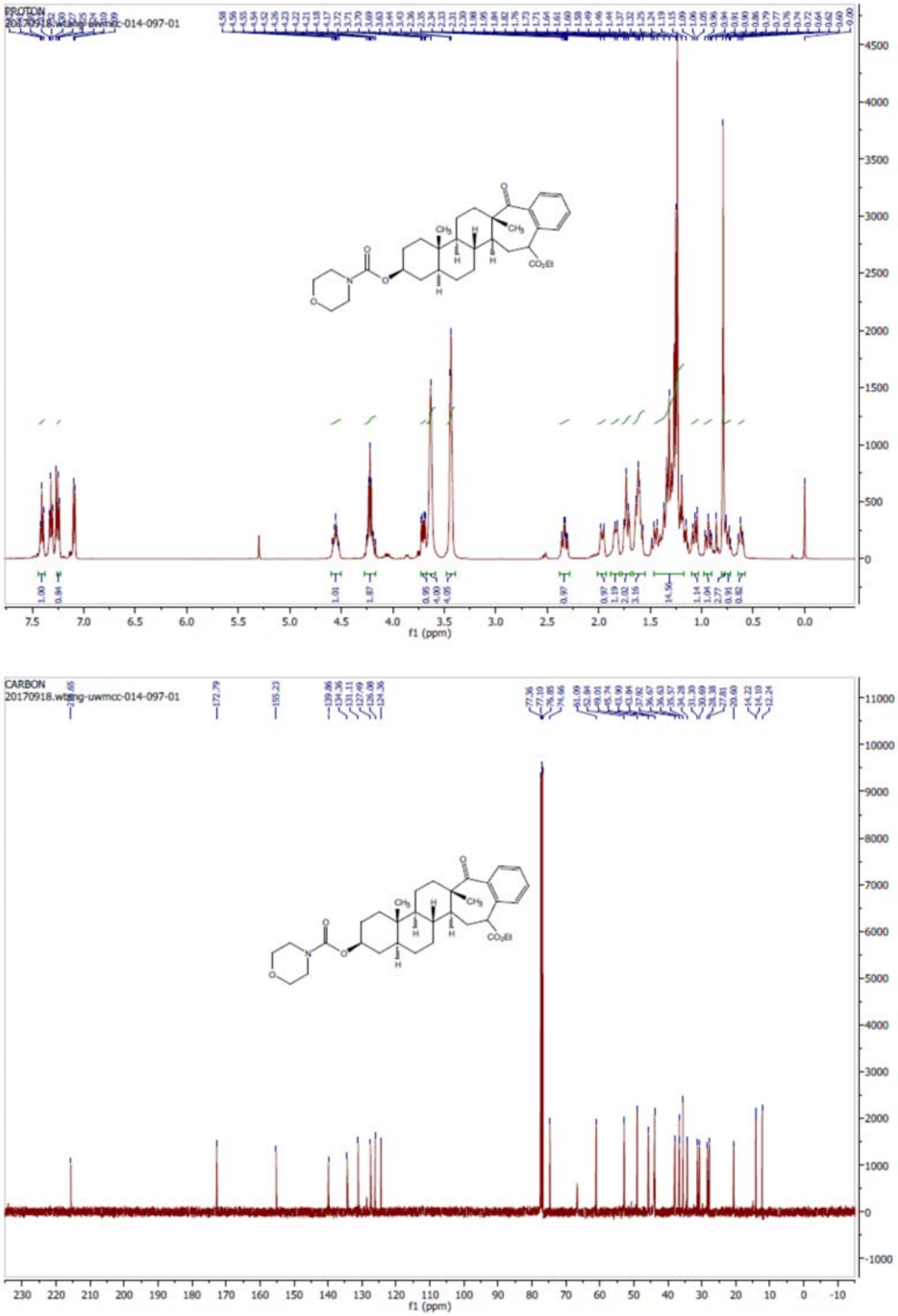

Supplementary Figure 153. ^{1}H and ^{13} C spectra of **42c**.

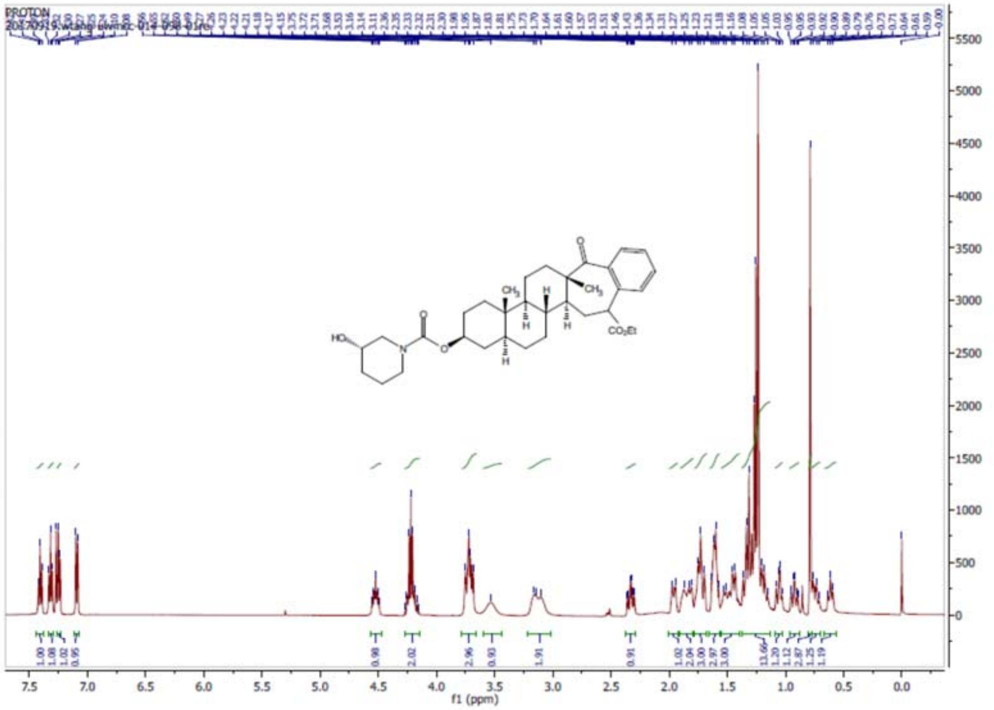

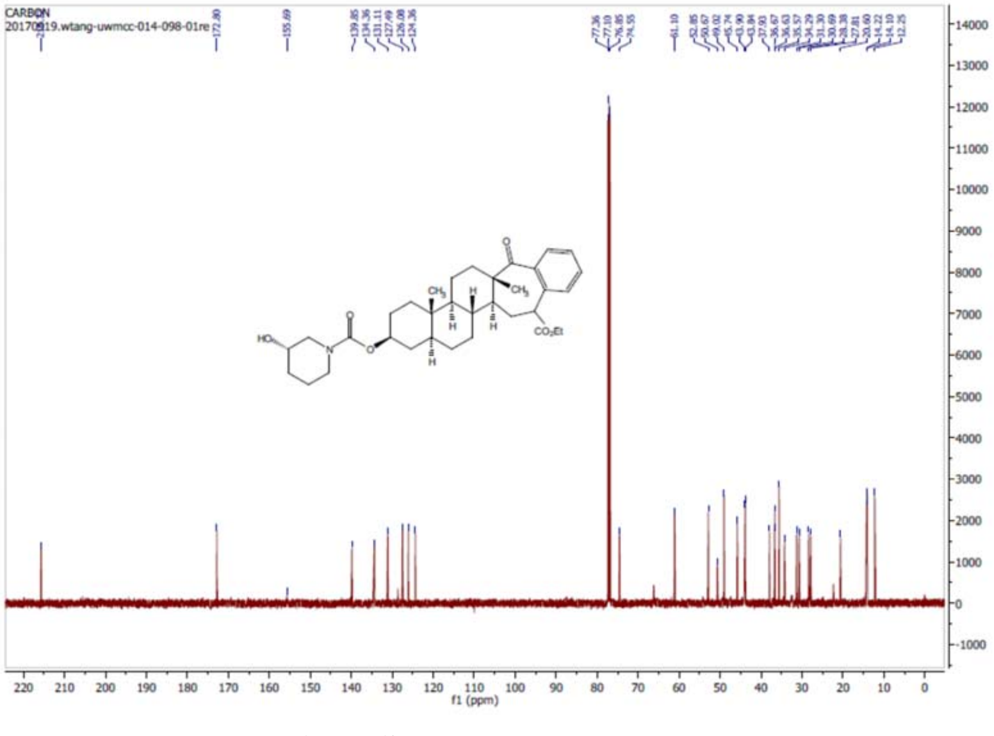

Supplementary Figure 154. ^{1}H and ^{13} C spectra of **42d**.

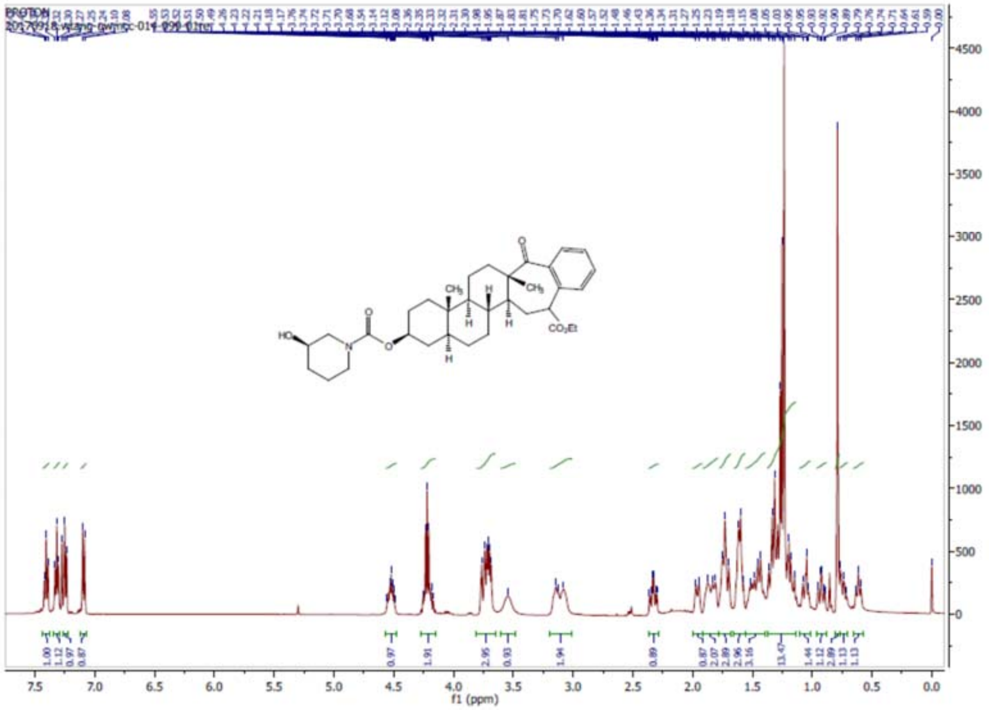

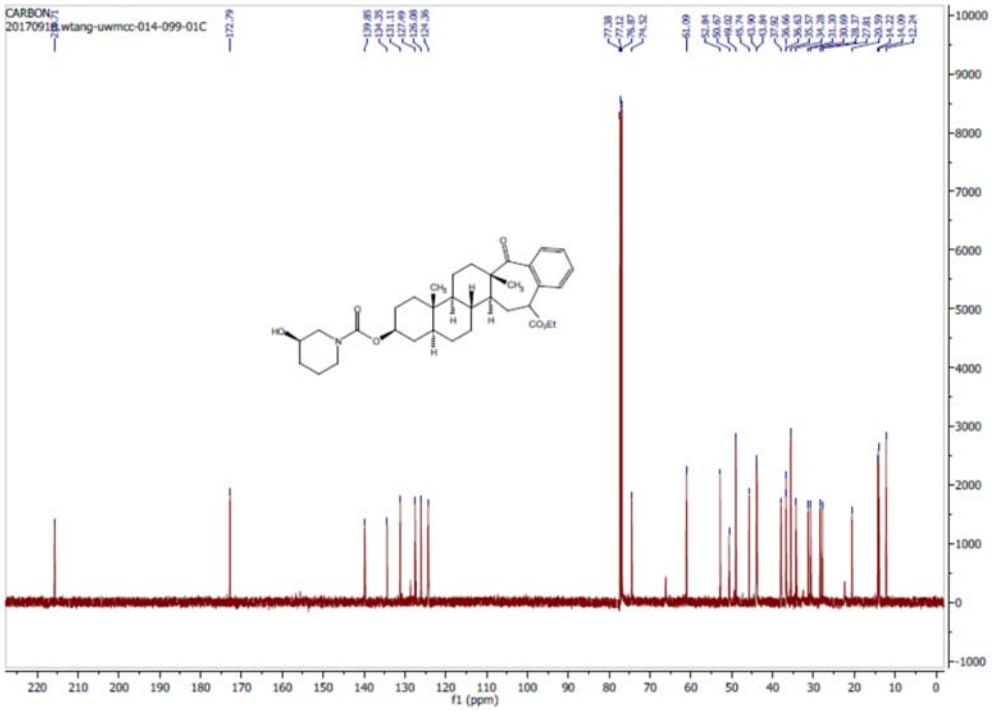

Supplementary Figure 155. ^{1}H and ^{13}C spectra of **42e**.

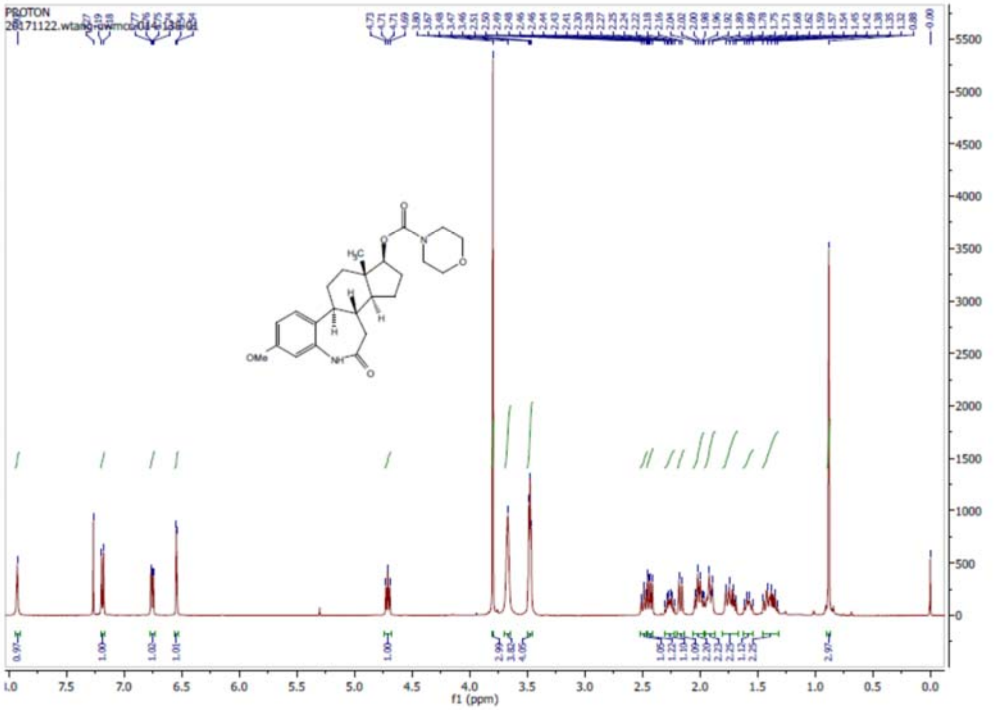

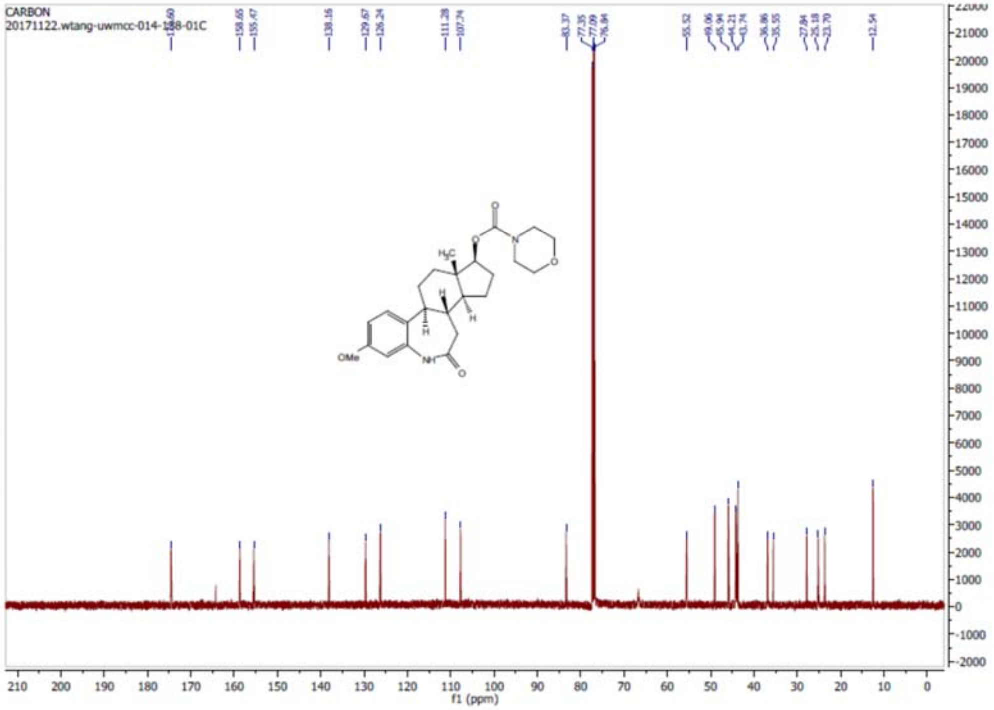

Supplementary Figure 156. ^{1}H and ^{13}C spectra of **43a**.

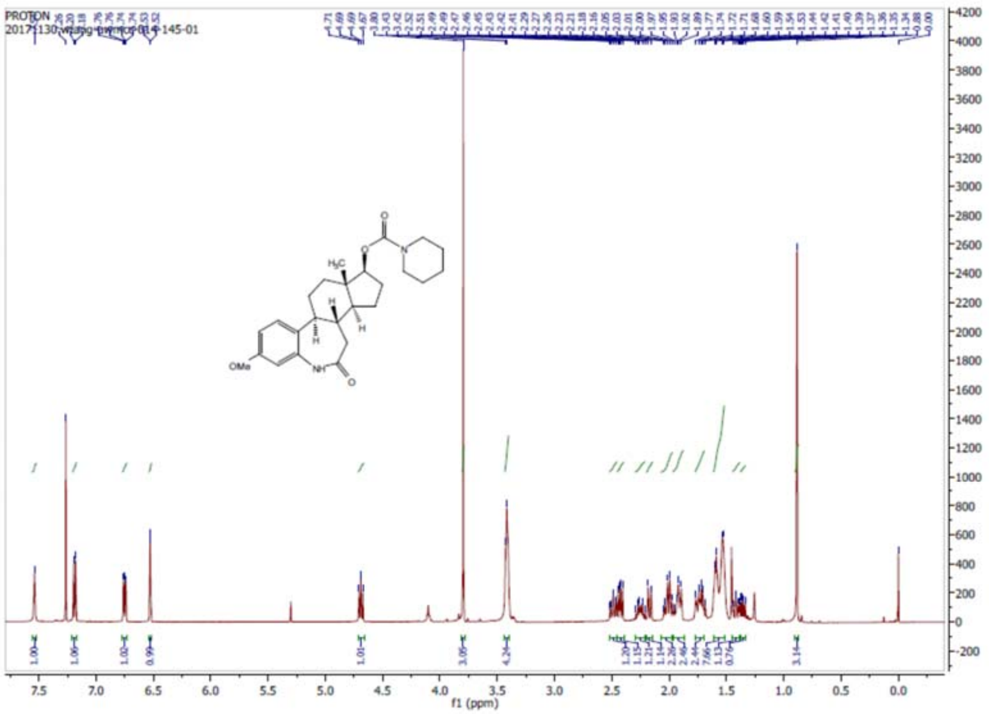

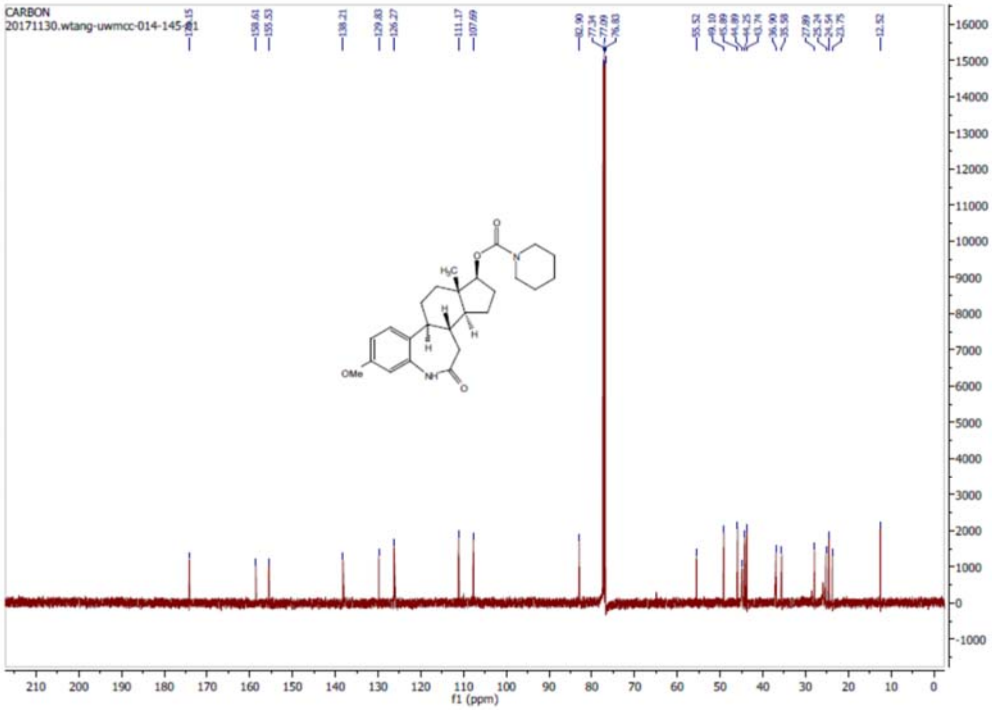

Supplementary Figure 157. ^{1}H and ^{13}C spectra of **43b**.

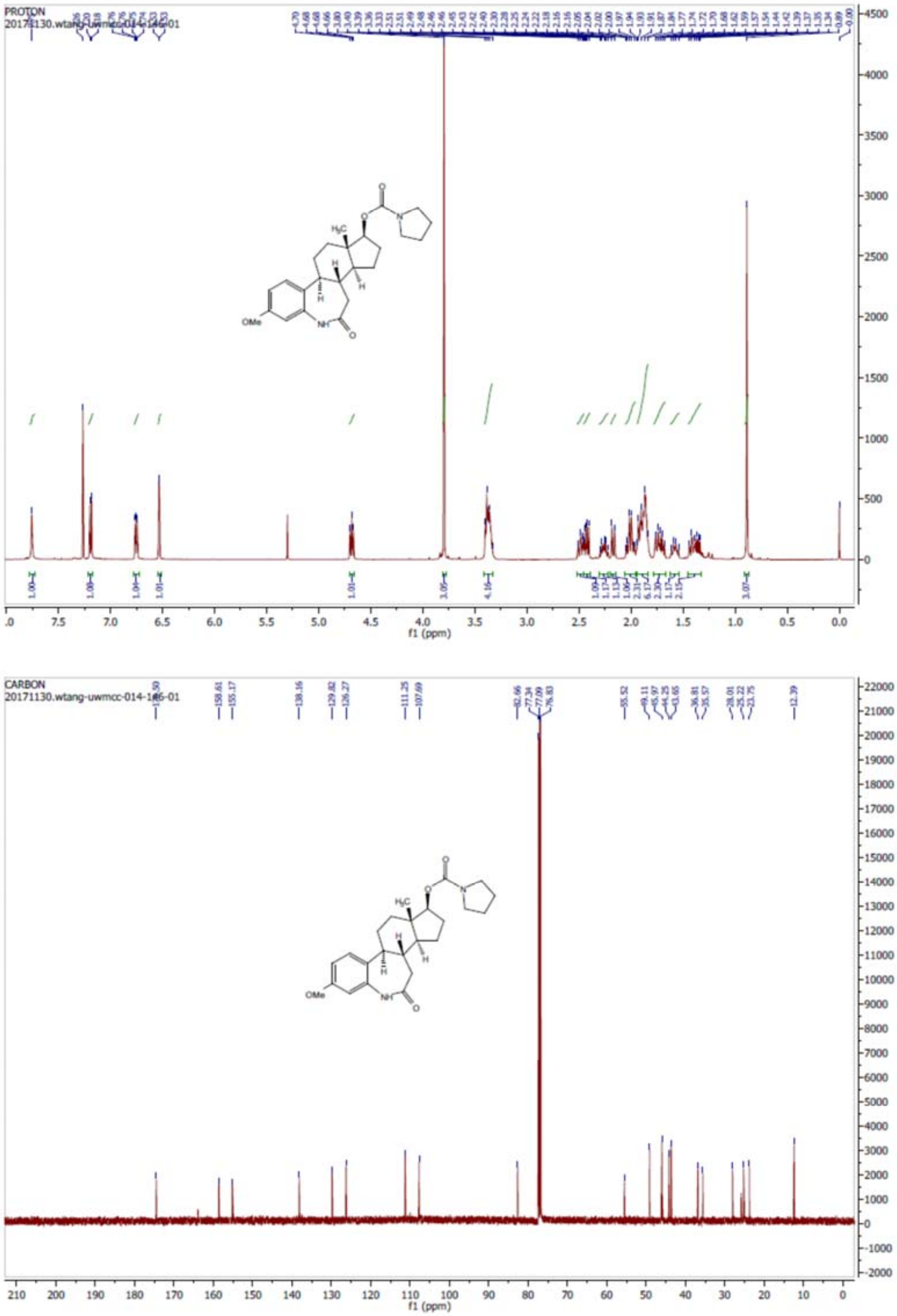

Supplementary Figure 158. ^{1}H and ^{13}C spectra of **43c**.

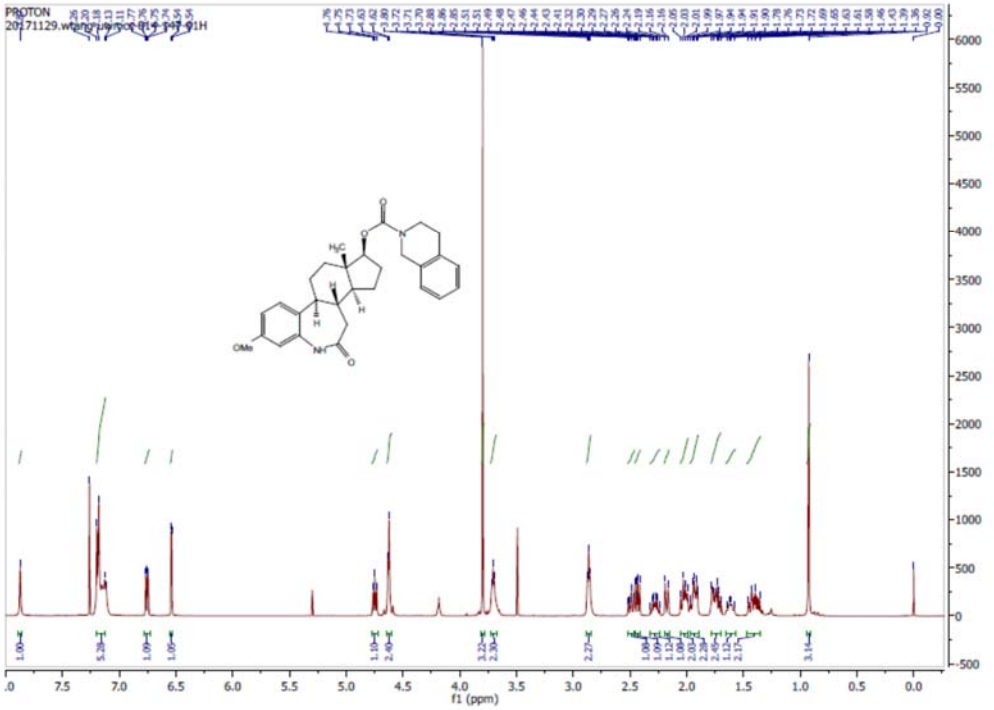

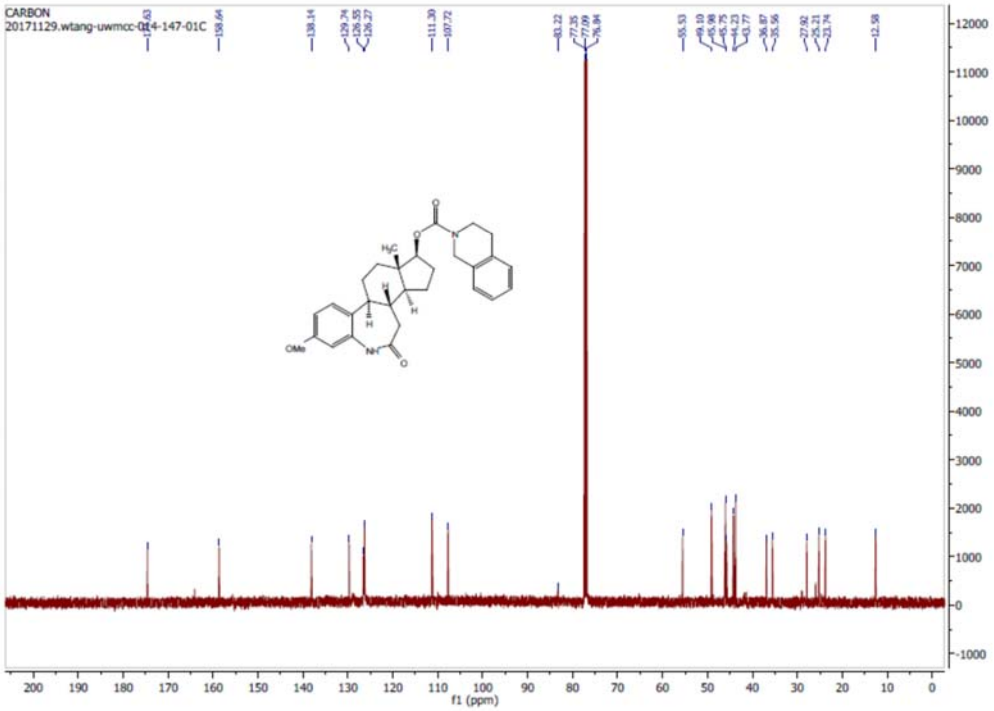

Supplementary Figure 159. ^{1}H and ^{13}C spectra of **43d**.

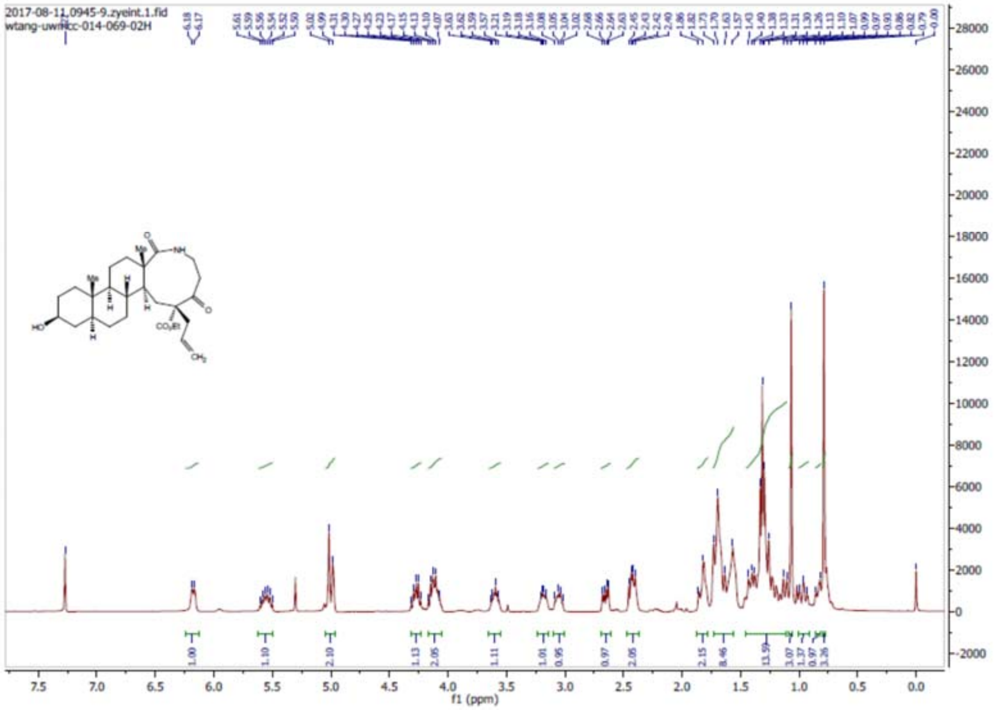

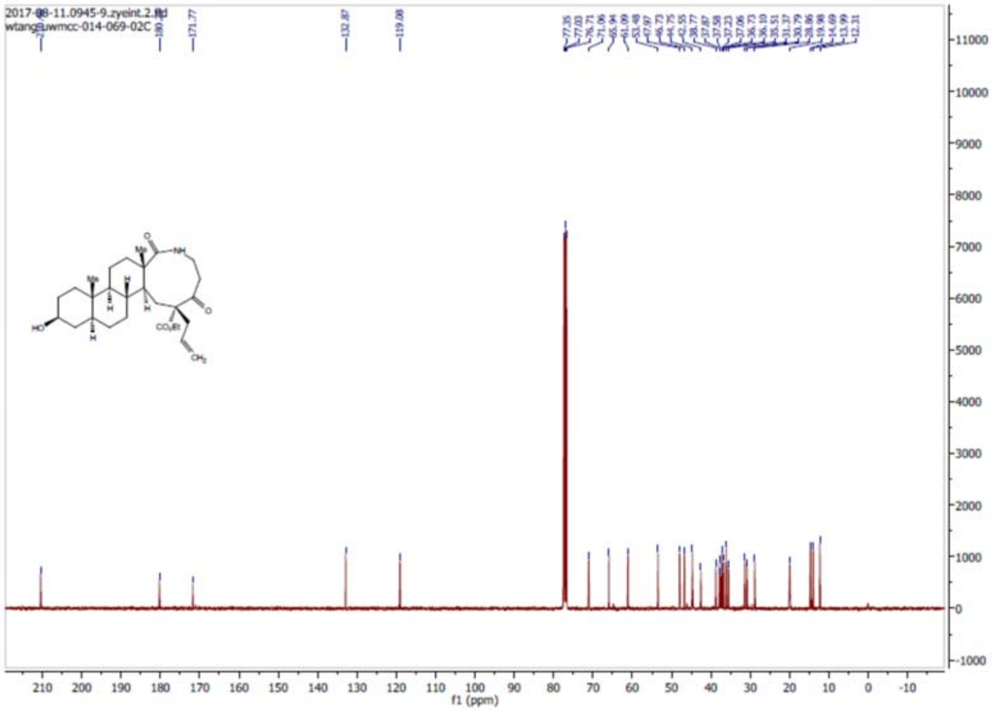

Supplementary Figure 160. ^{1}H and ^{13}C spectra of **S14a**.

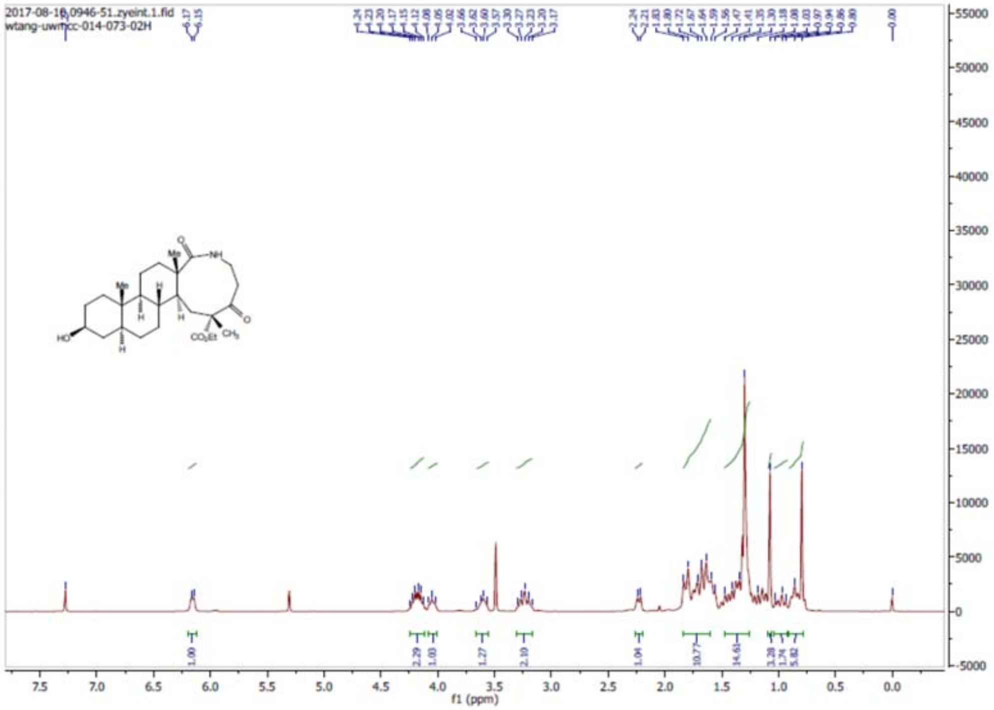

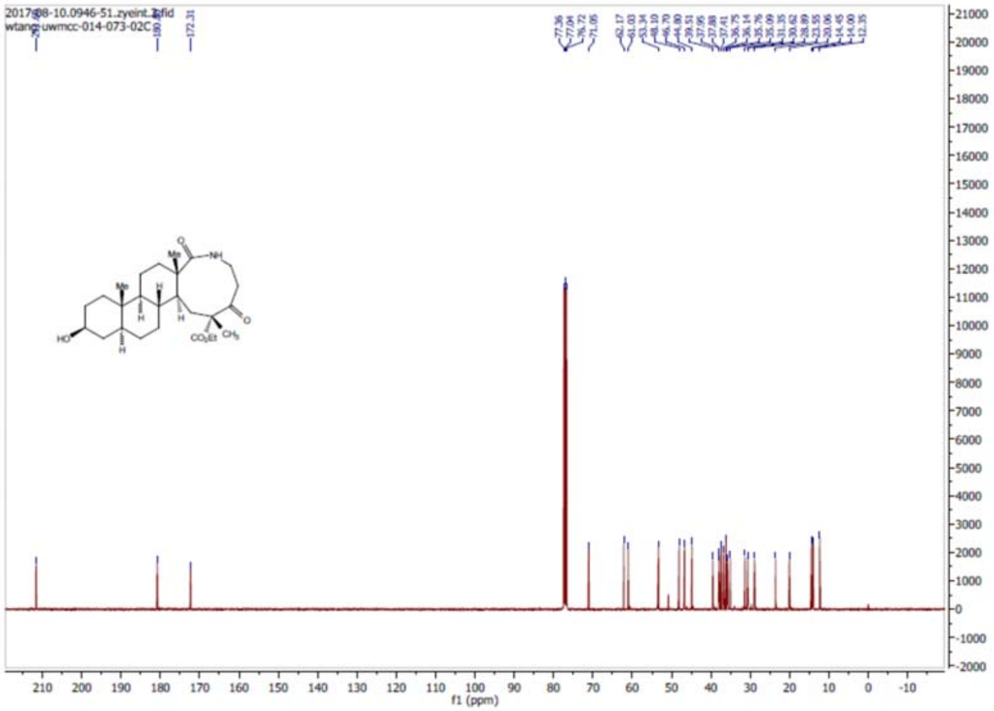

Supplementary Figure 161. ^{1}H and ^{13}C spectra of **S14b**.

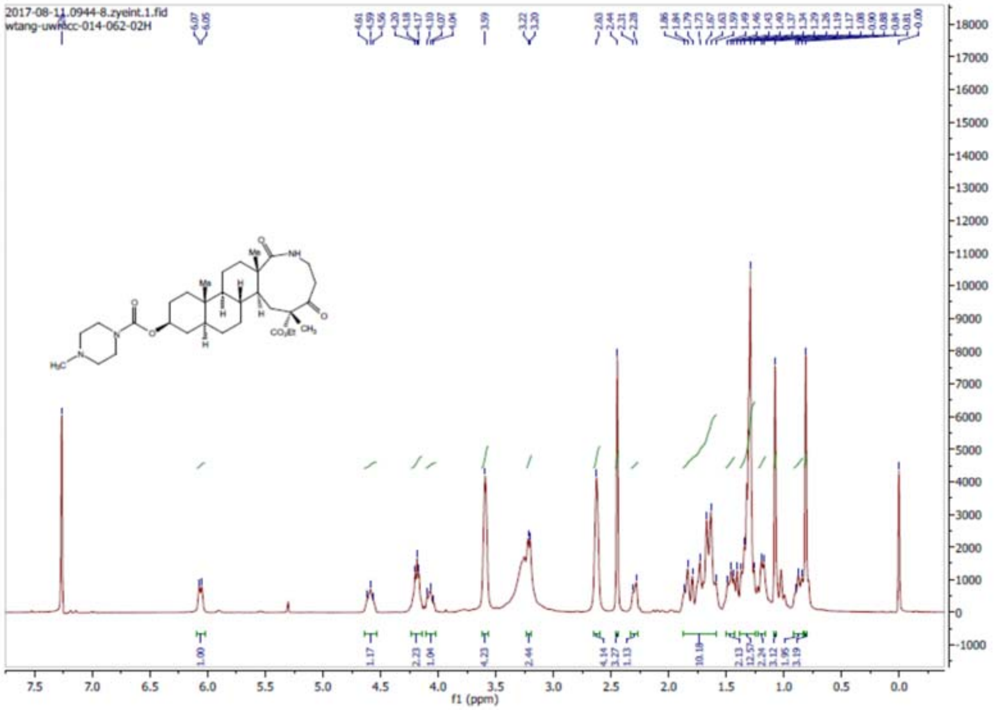

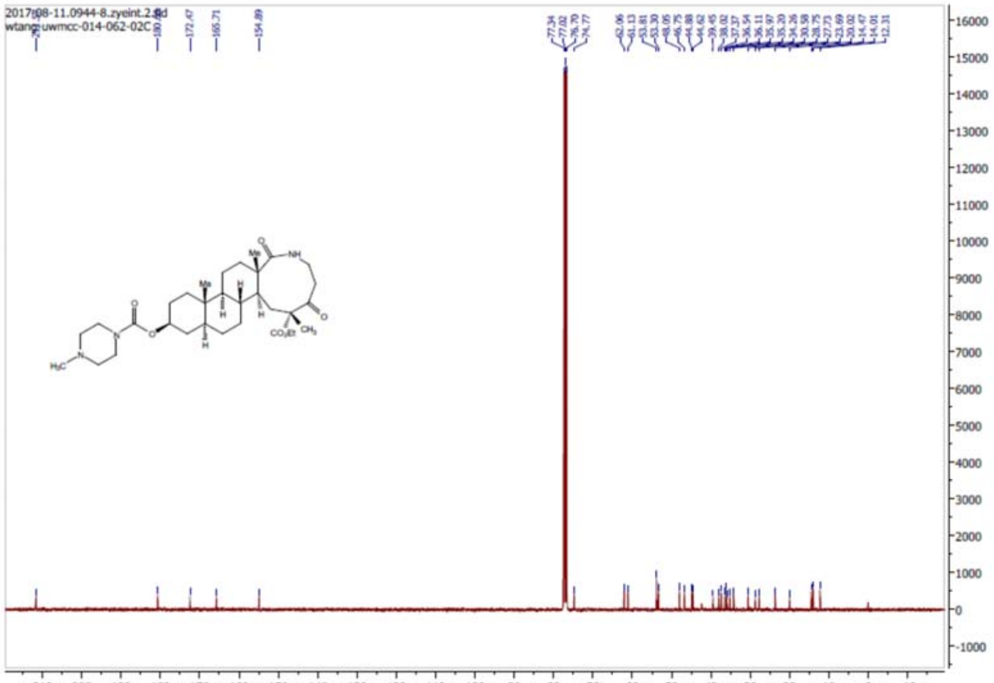

Supplementary Figure 162. ^{1}H and ^{13}C spectra of **45a**.

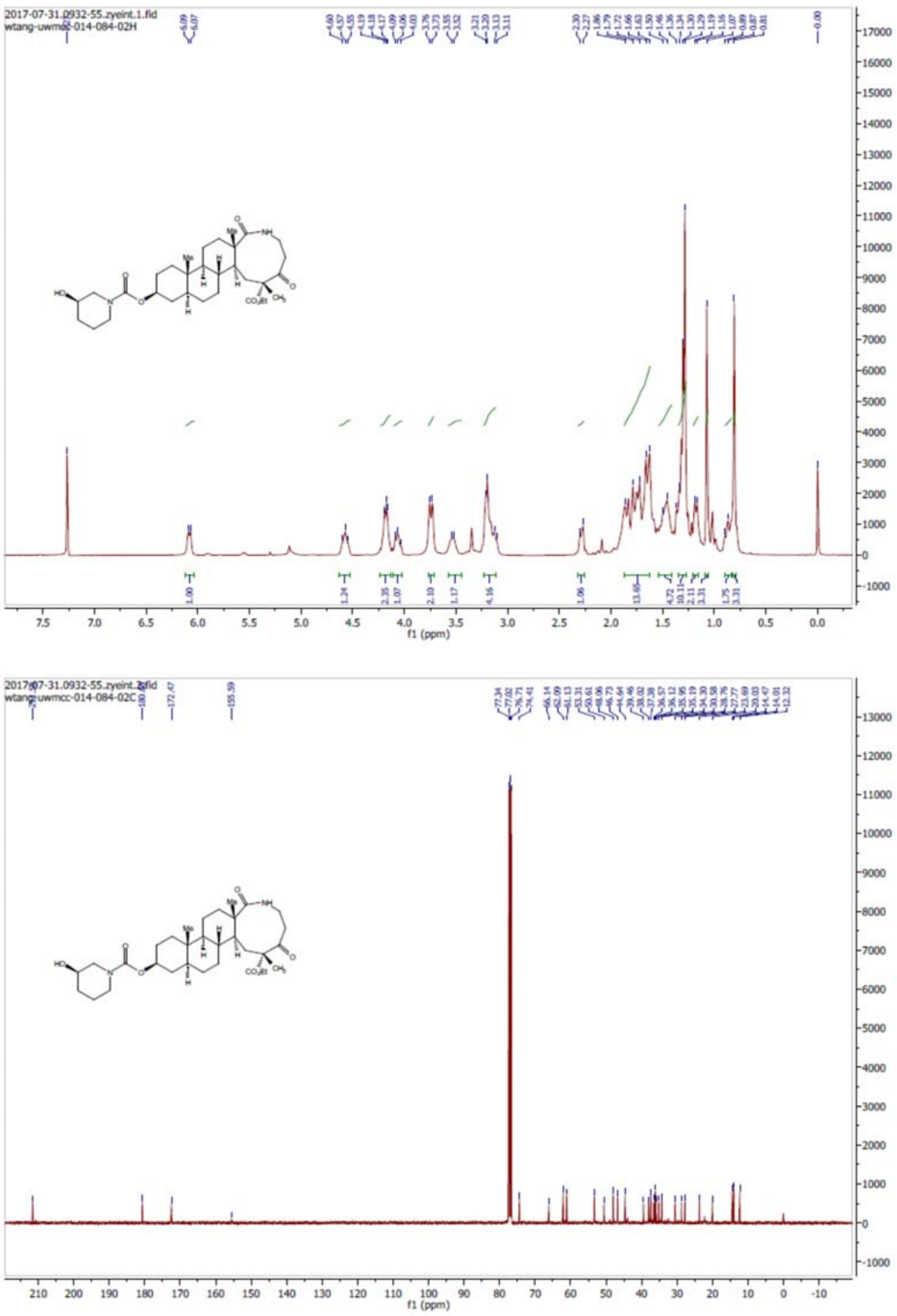

Supplementary Figure 163. ^{1}H and ^{13}C spectra of **45b**.

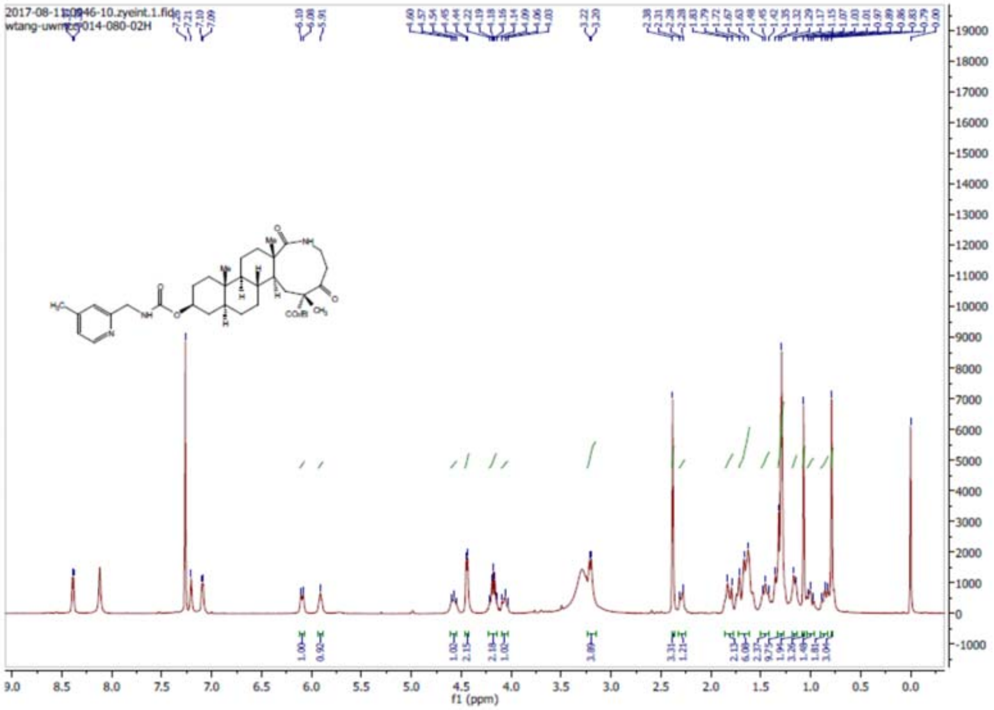

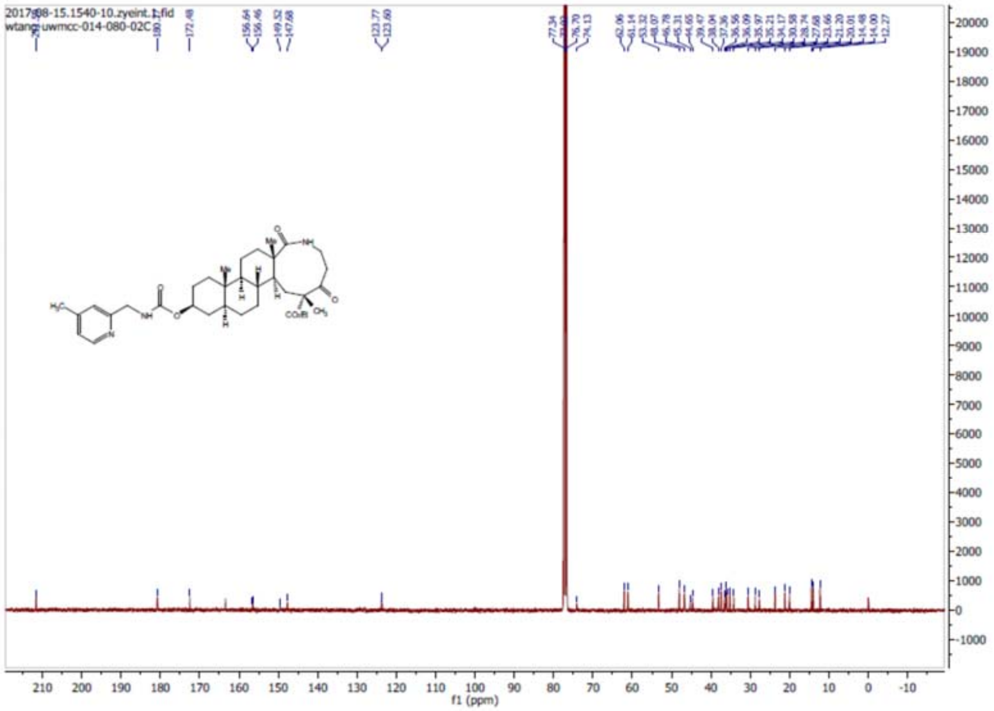

Supplementary Figure 164. ^{1}H and ^{13}C spectra of **45c**.

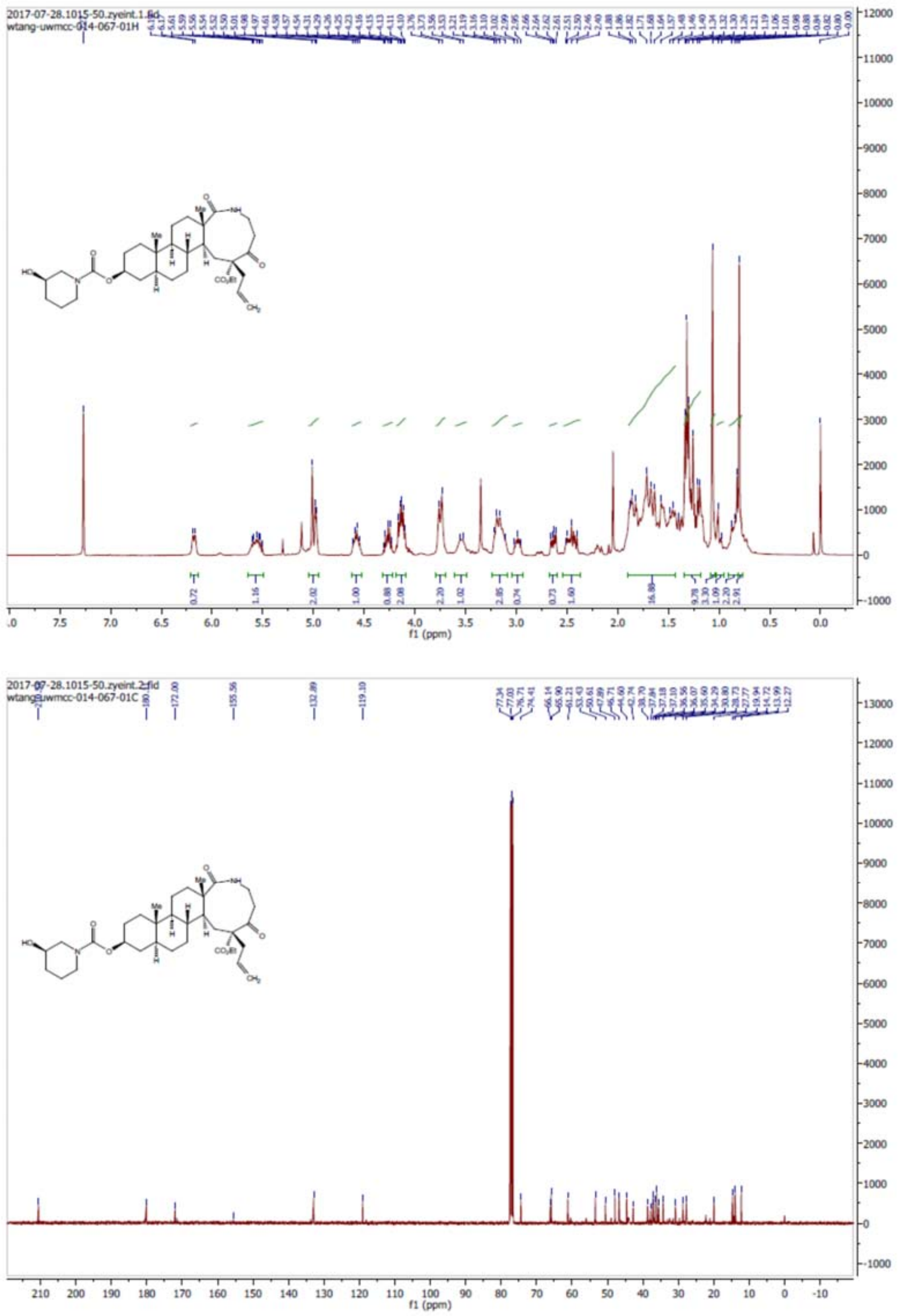

Supplementary Figure 165. ^{1}H and ^{13} C spectra of **45d**.

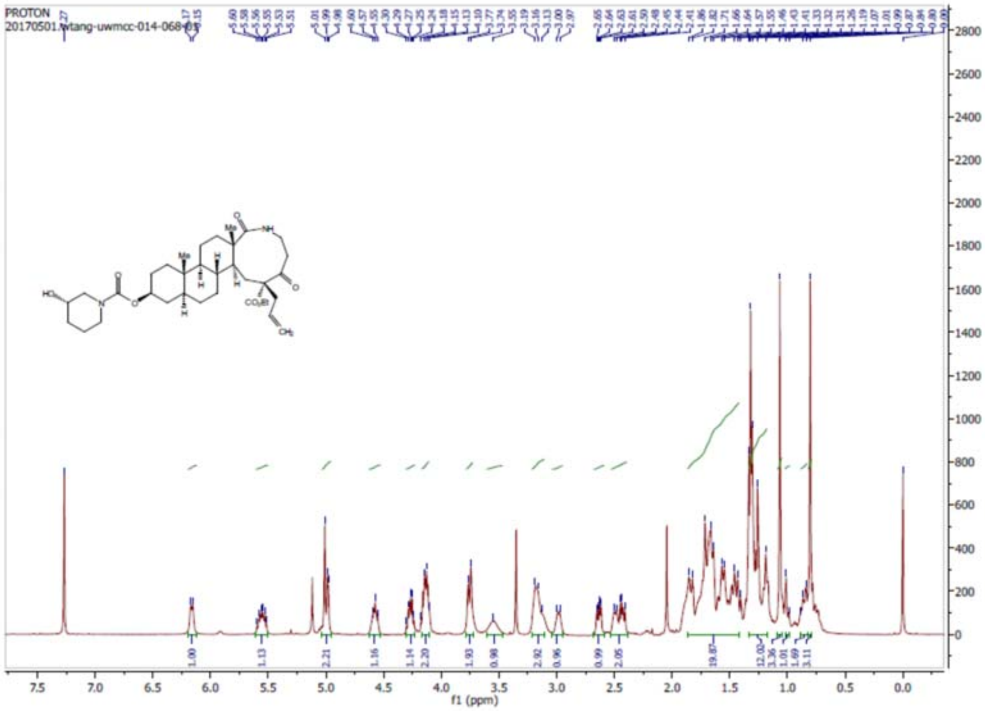

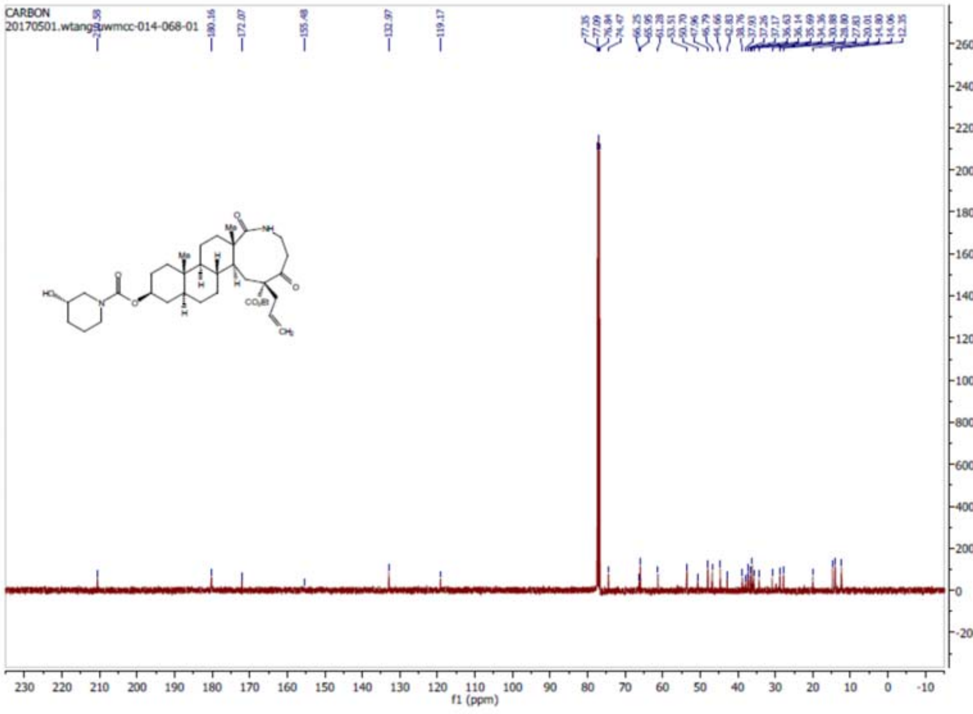

Supplementary Figure 166. ^{1}H and ^{13}C spectra of **45e**.

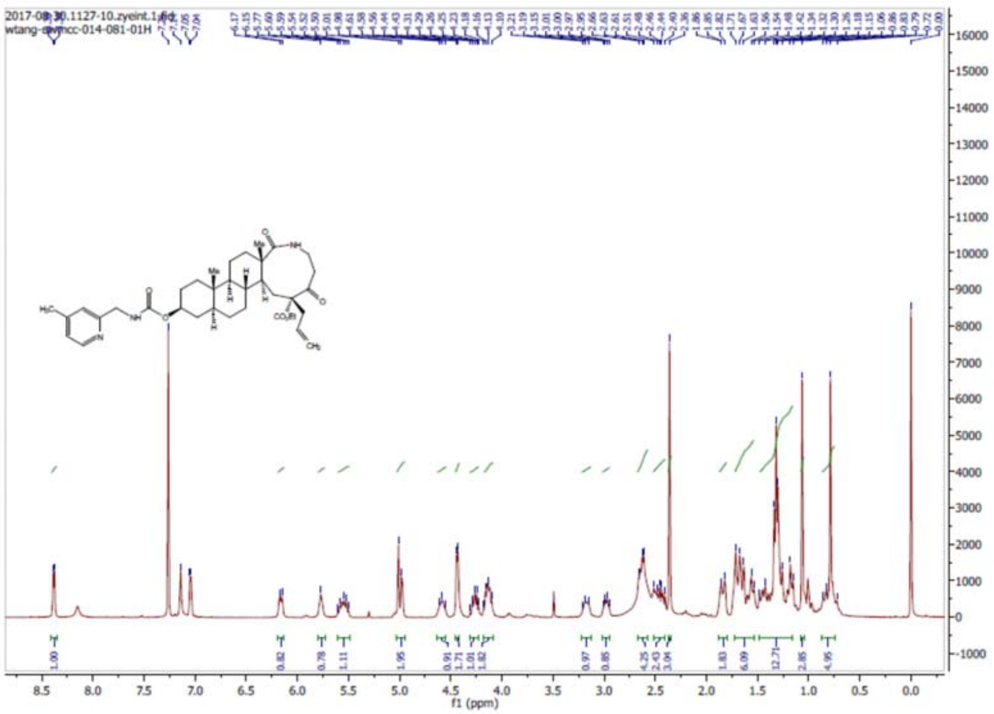

Supplementary Figure 167. ^{1}H and spectra of **45f**.

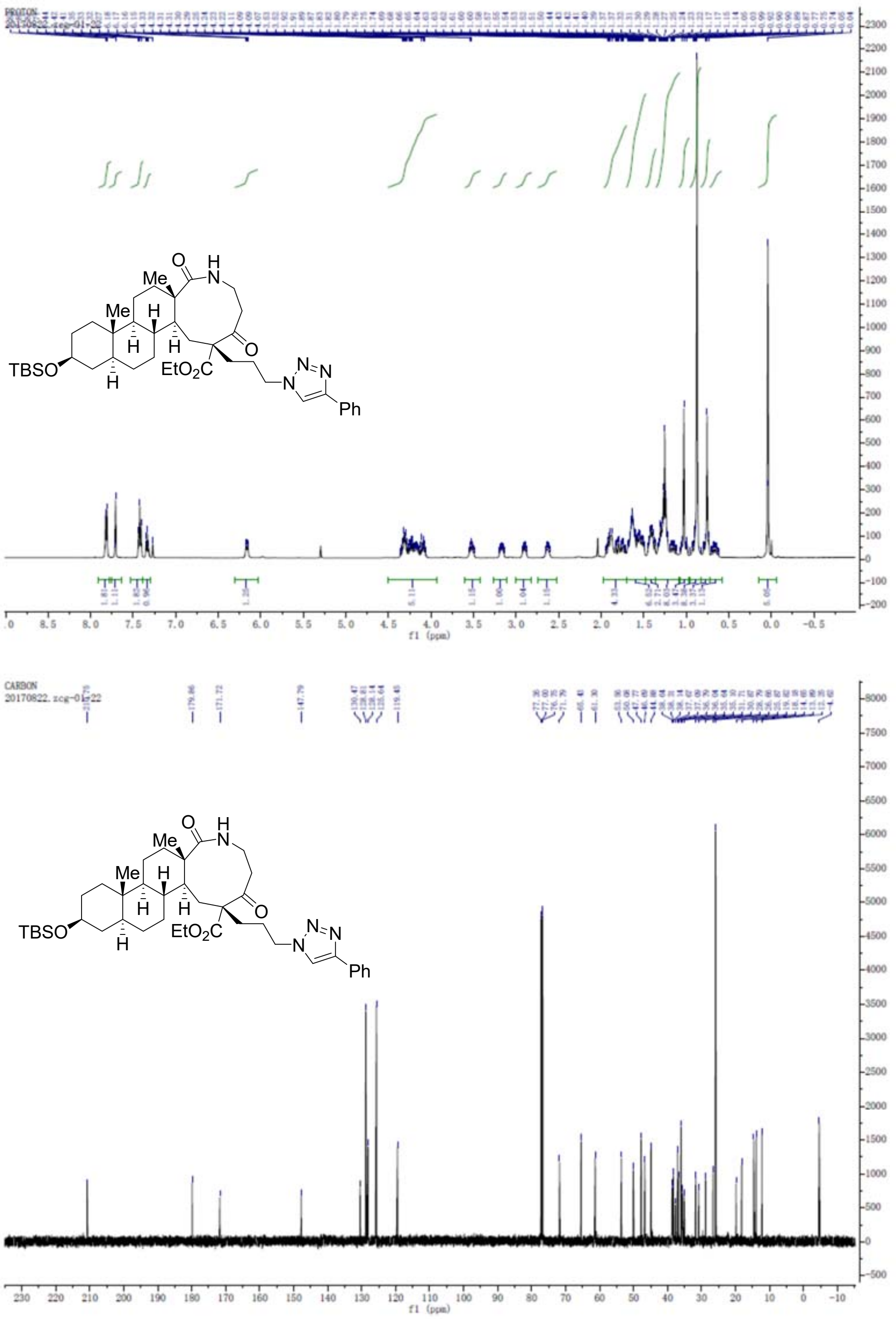

Supplementary Figure 168. ^{1}H and ^{13}C spectra of **S46a**.

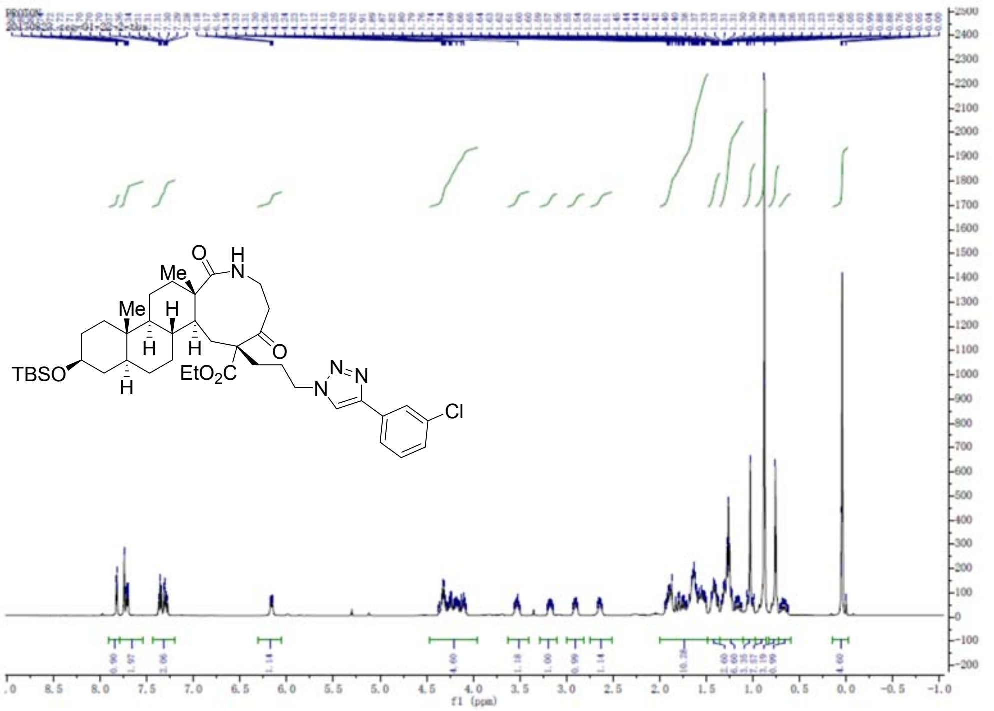

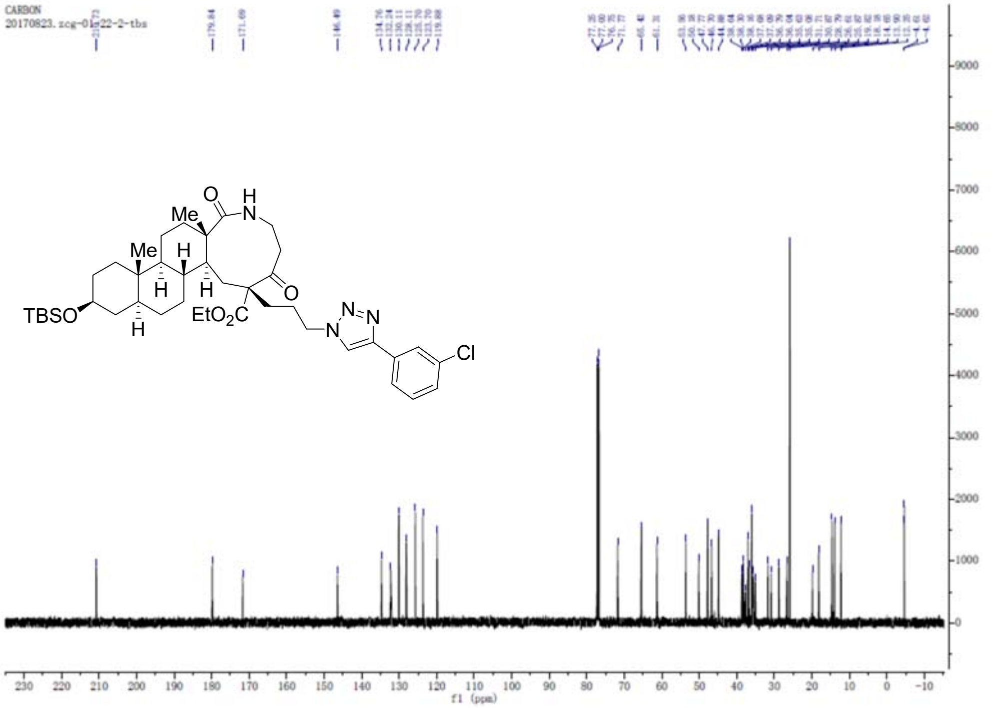

Supplementary Figure 169. ^{1}H and ^{13}C spectra of **S46b**.

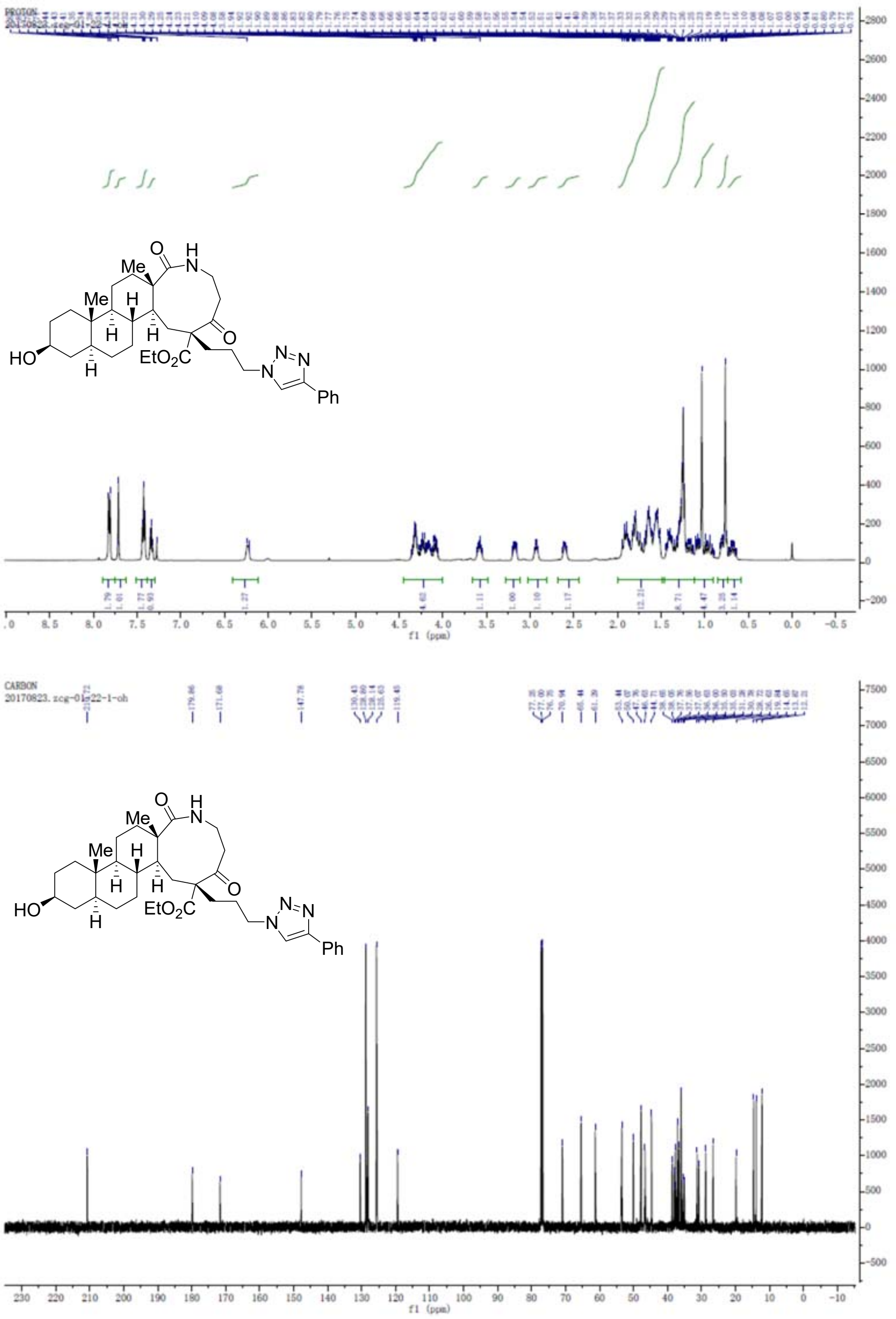

Supplementary Figure 170. ^{1}H and ^{13}C spectra of **46a**.

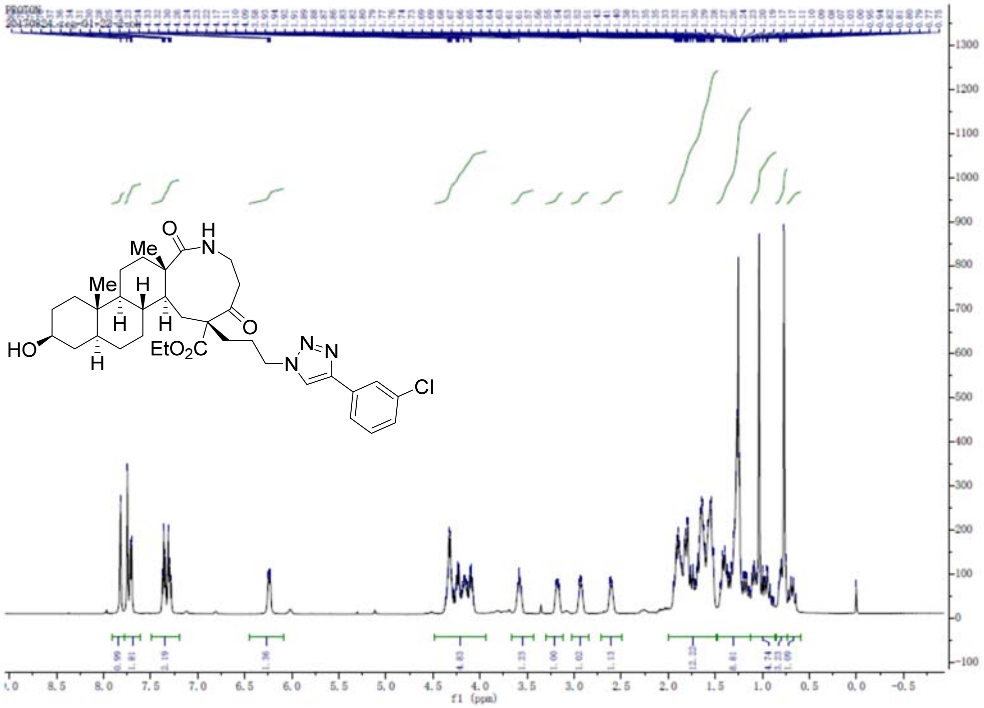

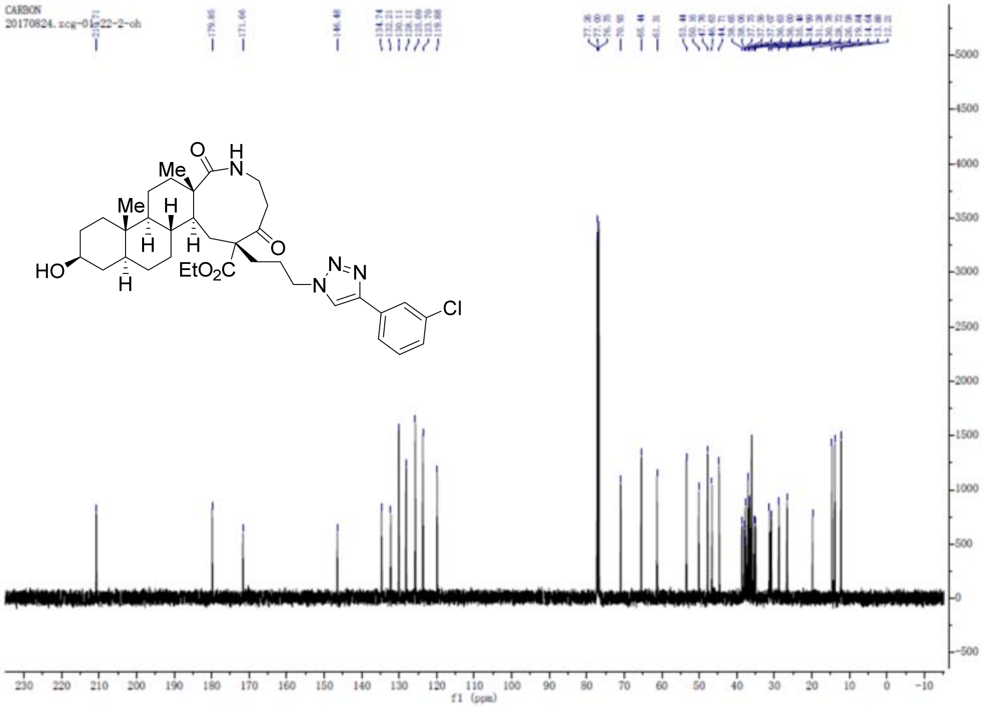

Supplementary Figure 171. ^{1}H and ^{13}C spectra of **46b**.

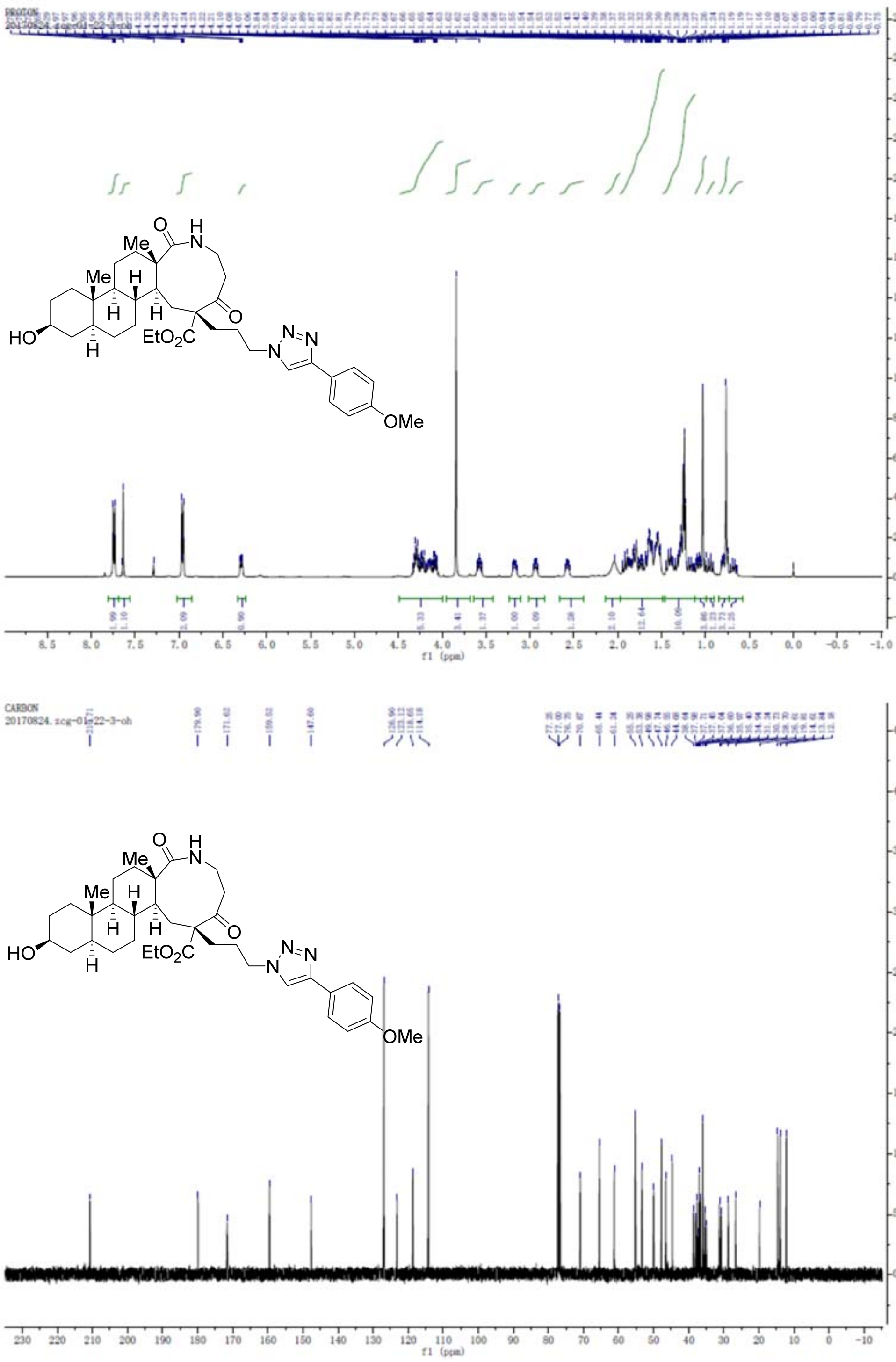

Supplementary Figure 172. ^{1}H and ^{13} C spectra of **46c**.

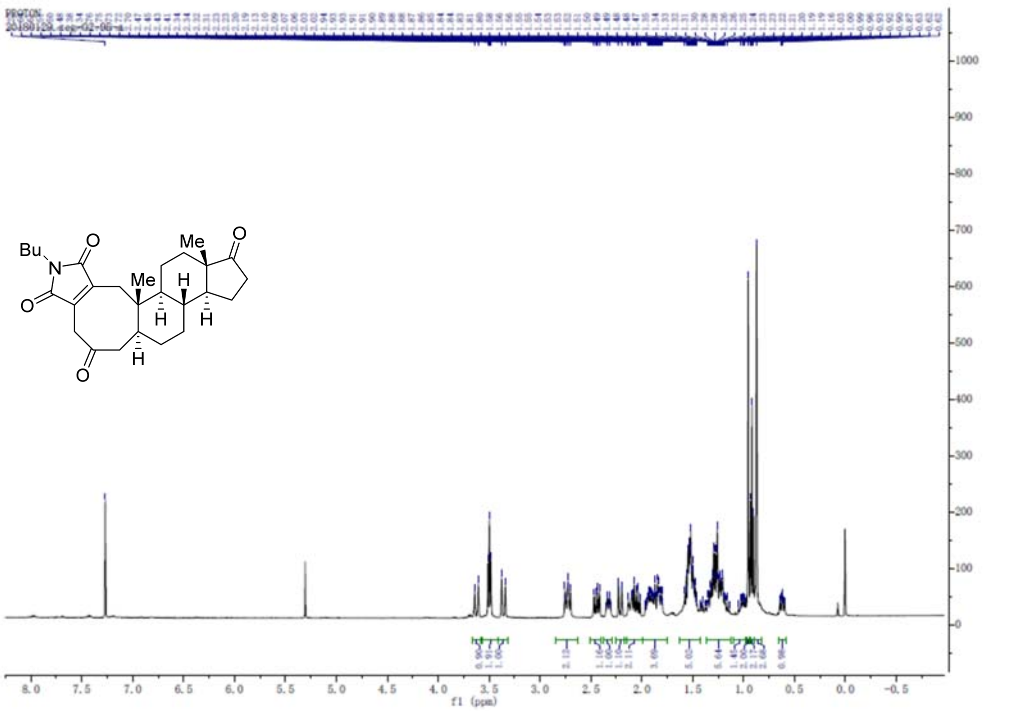

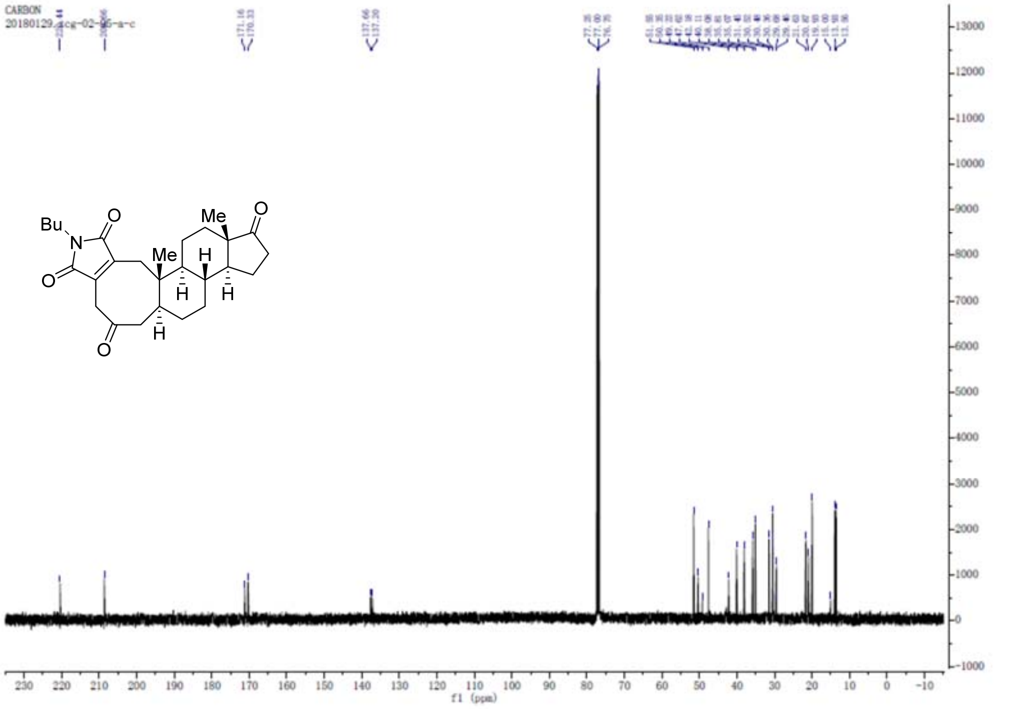

Supplementary Figure 173. ^{1}H and ^{13}C spectra of **48a**.

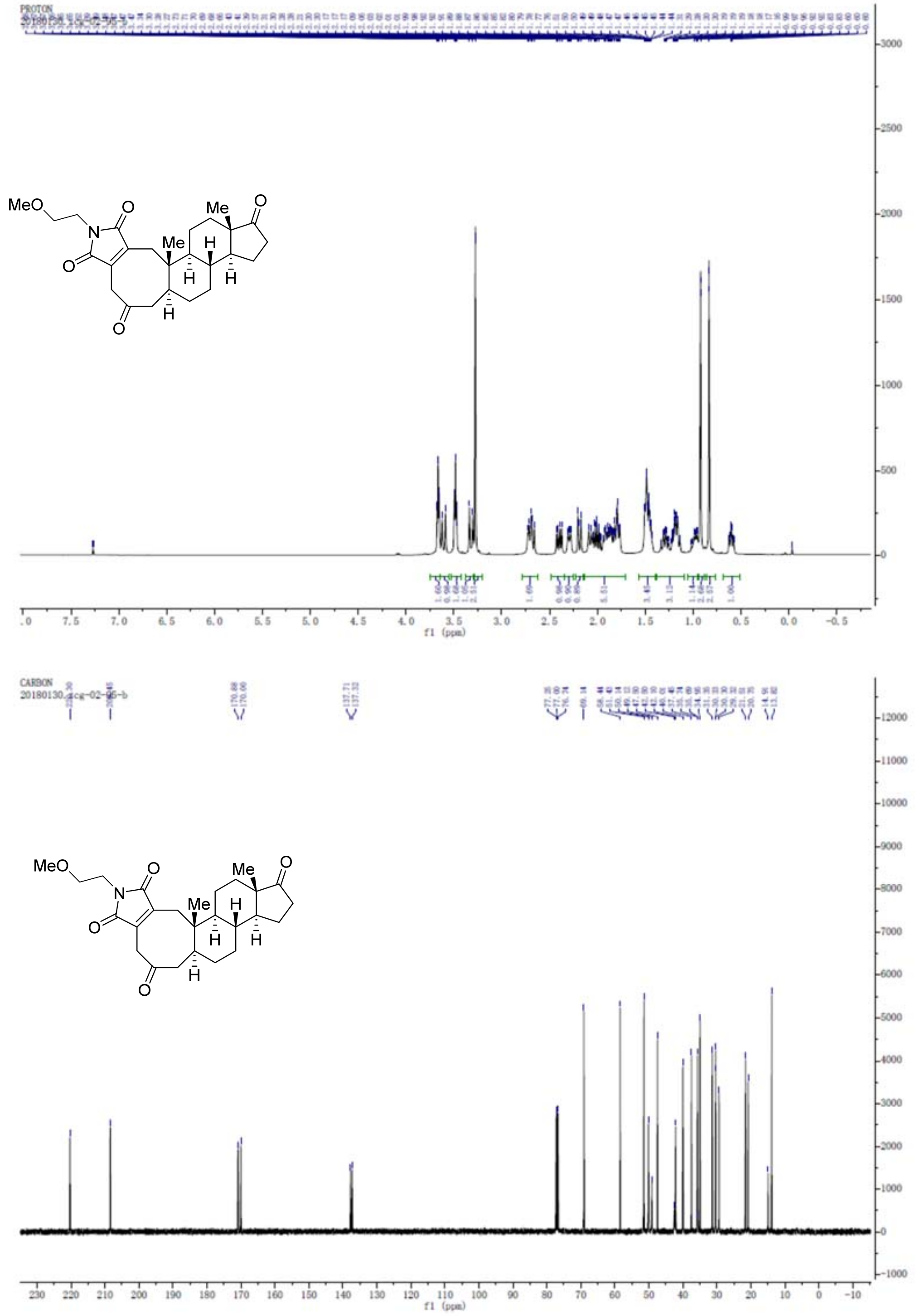

Supplementary Figure 174. ^{1}H and ^{13} C spectra of **48b**.

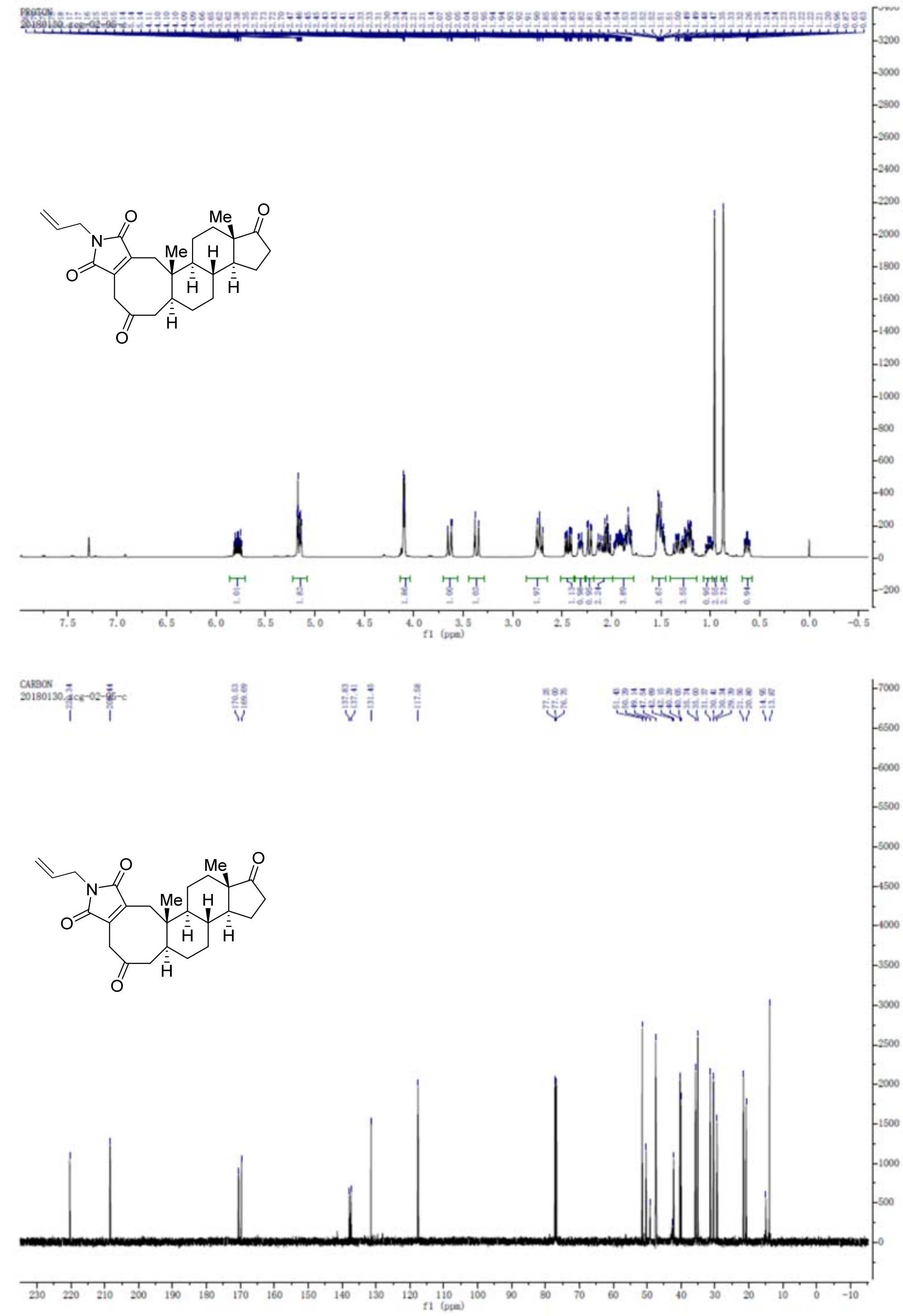

Supplementary Figure 175. ^{1}H and ^{13}C spectra of **48c**.

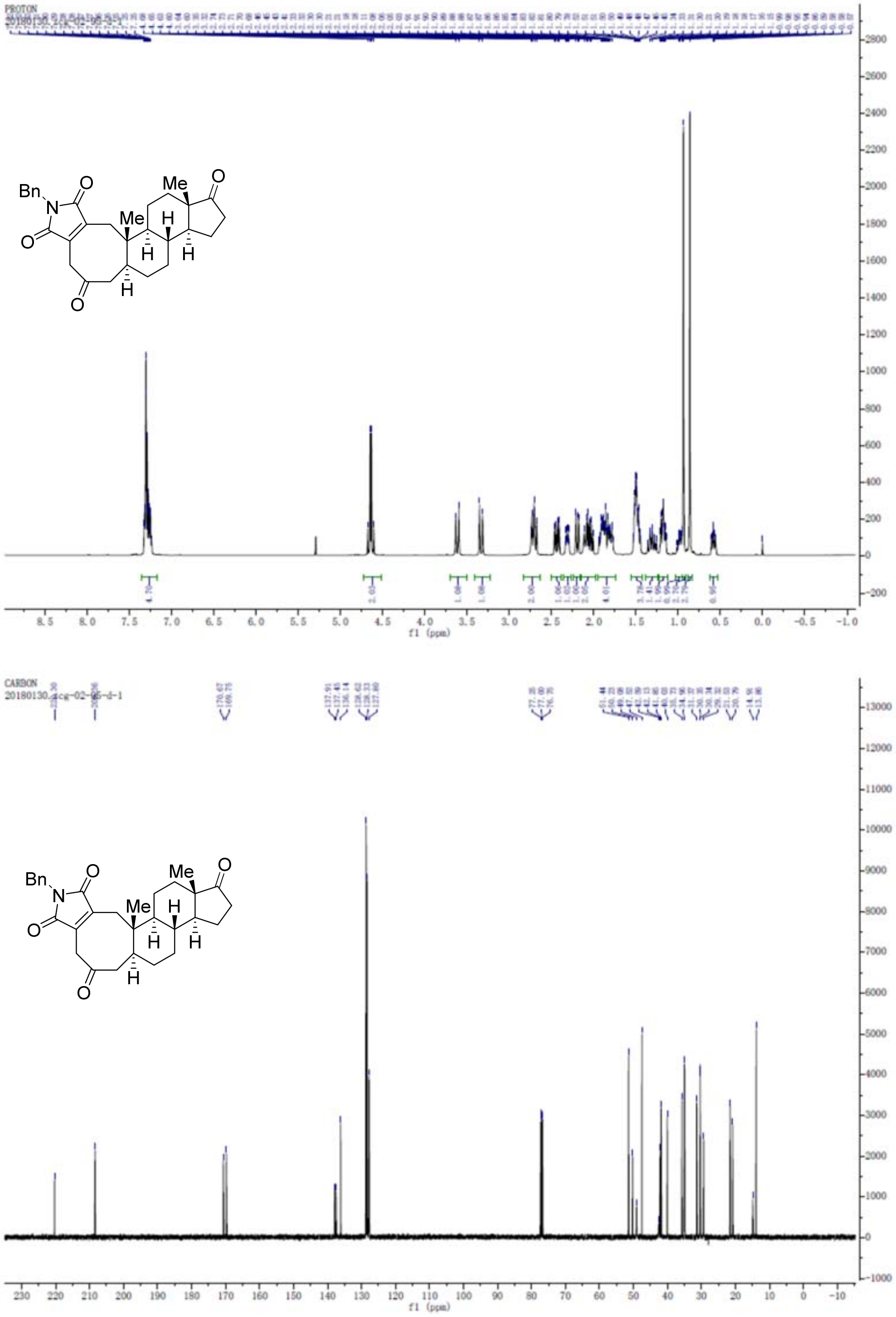

Supplementary Figure 176. ^{1}H and ^{13} C spectra of **48d**.

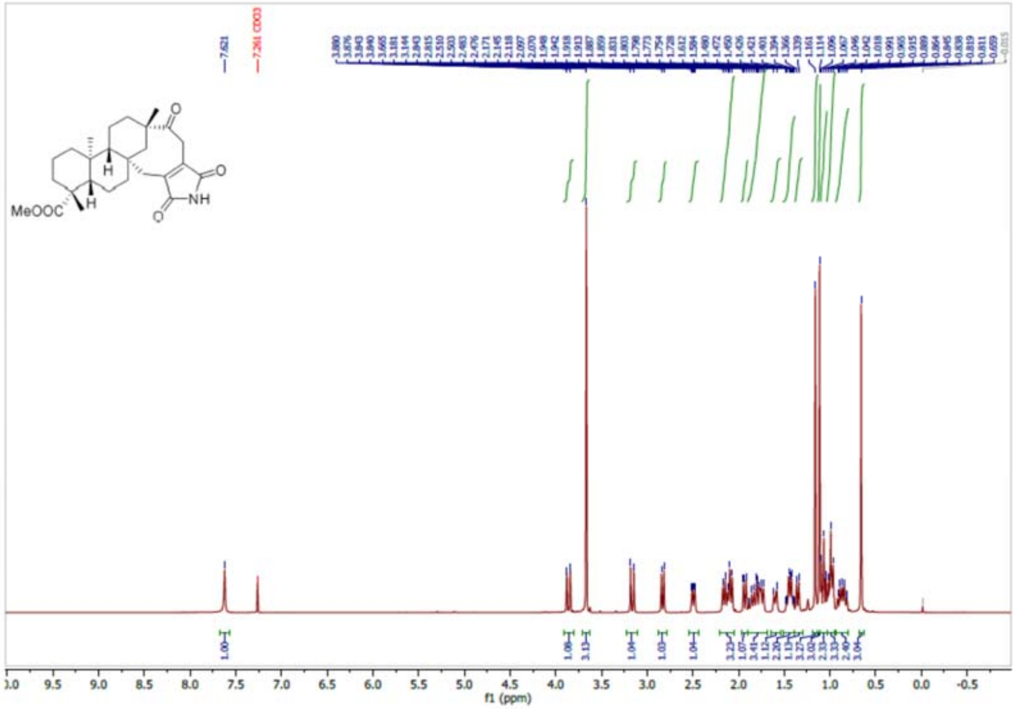

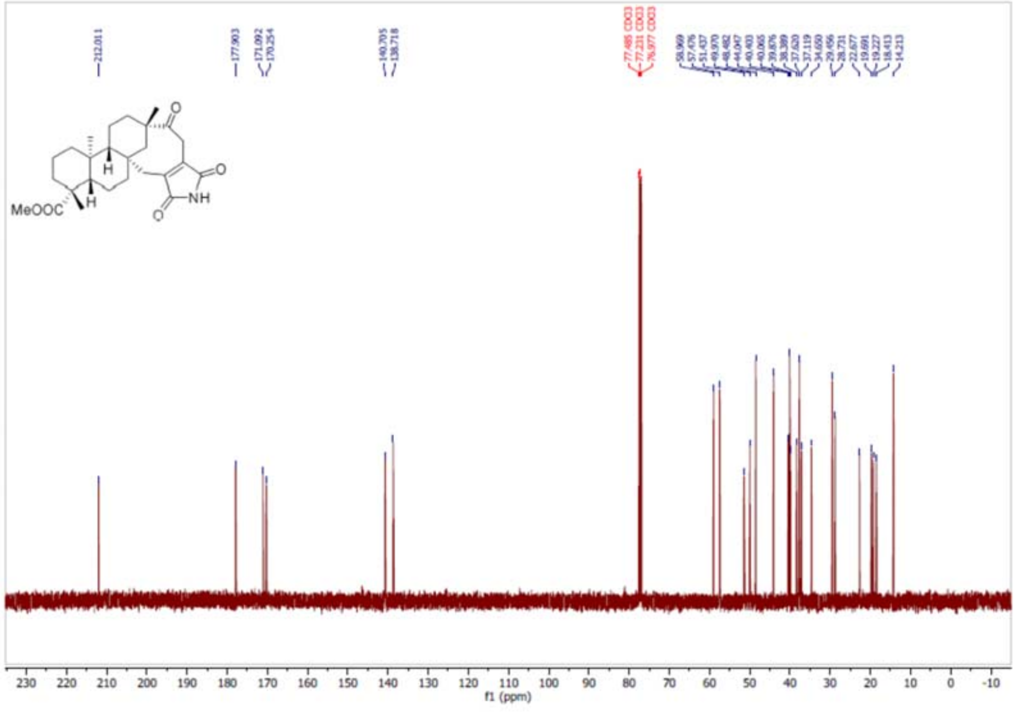

Supplementary Figure 177. ^{1}H and ^{13} C spectra of **47aa**.

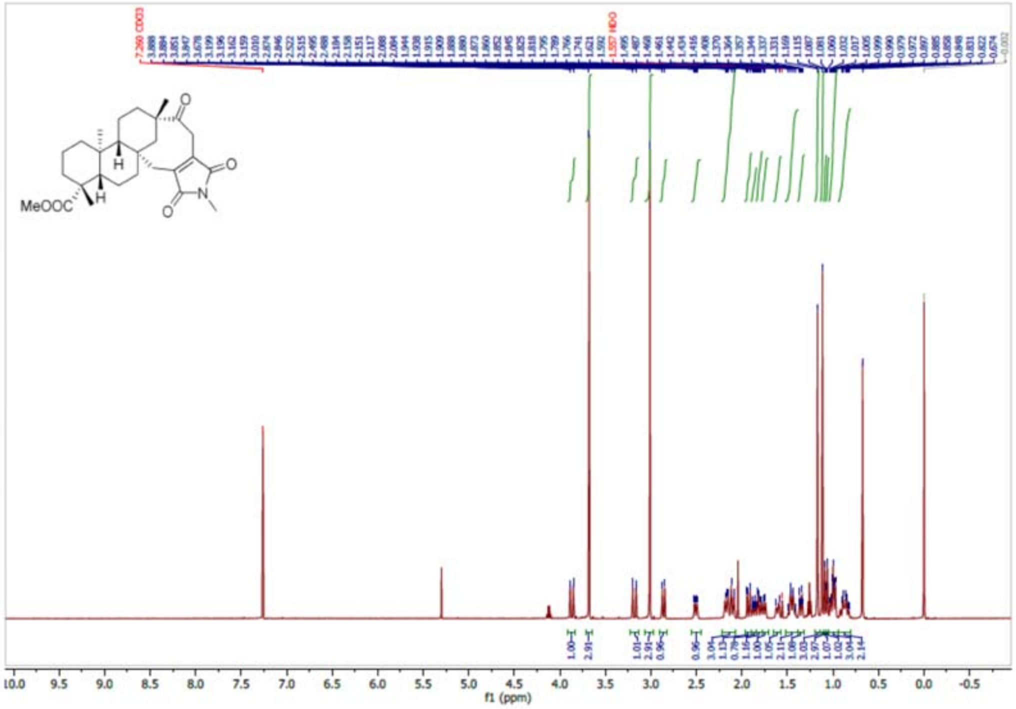

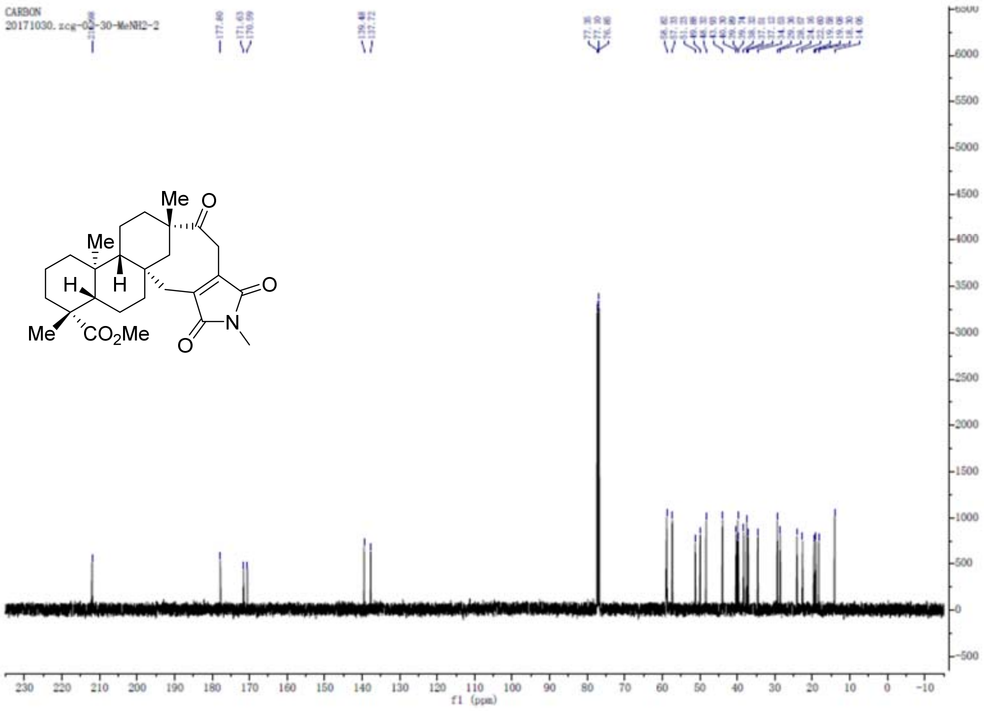

Supplementary Figure 178. ^{1}H and [13] C spectra of **47ab**.

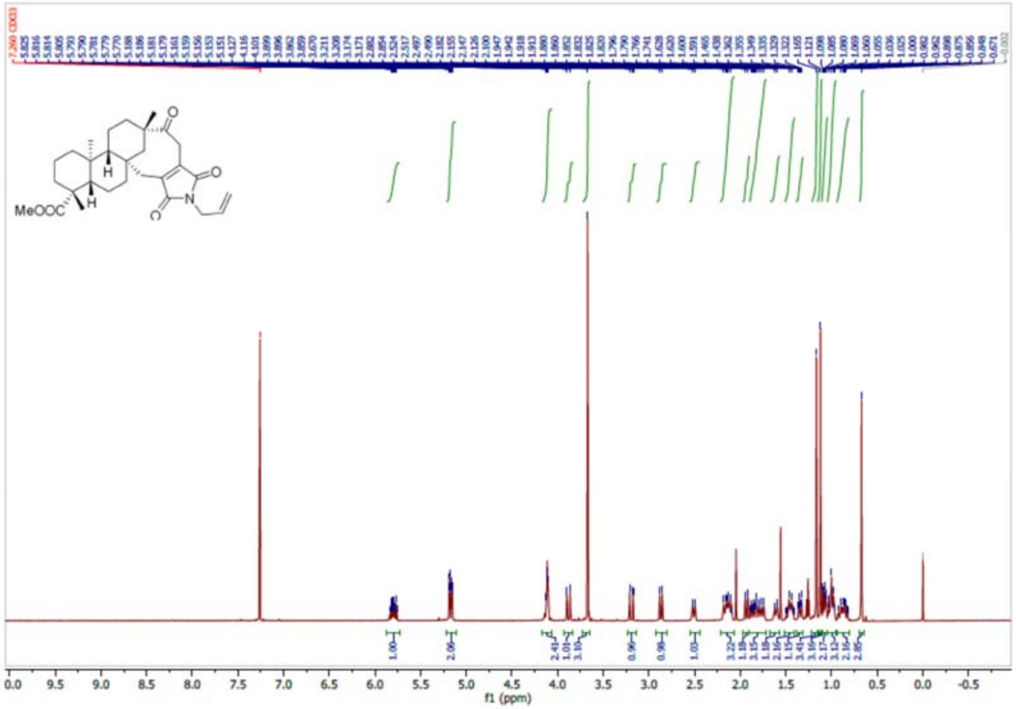

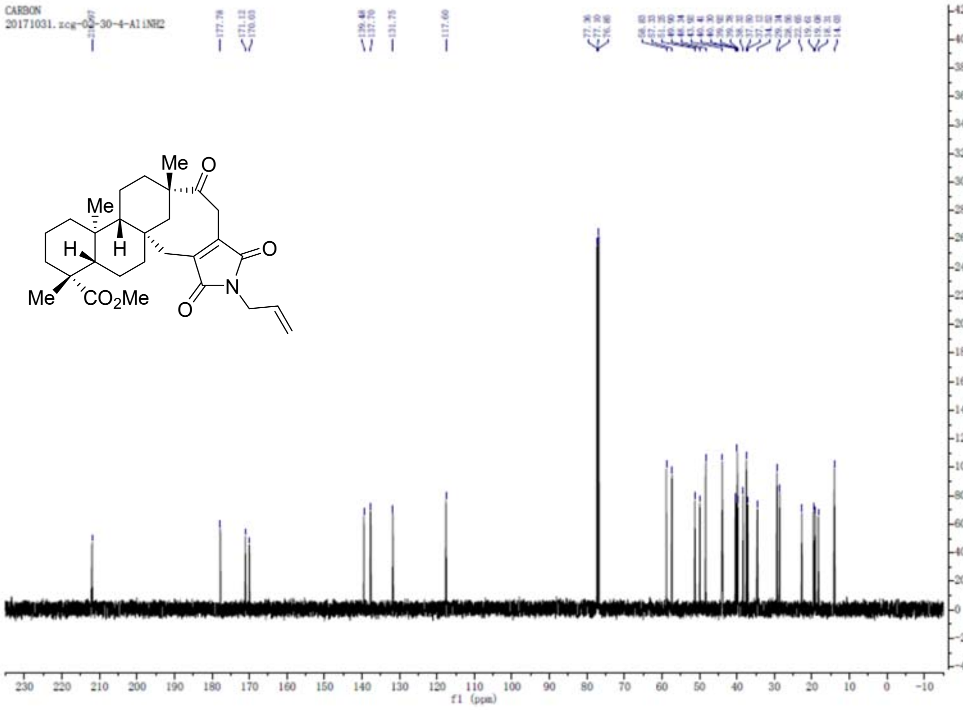

Supplementary Figure 179. ^{1}H and ^{13} C spectra of **47ac**.

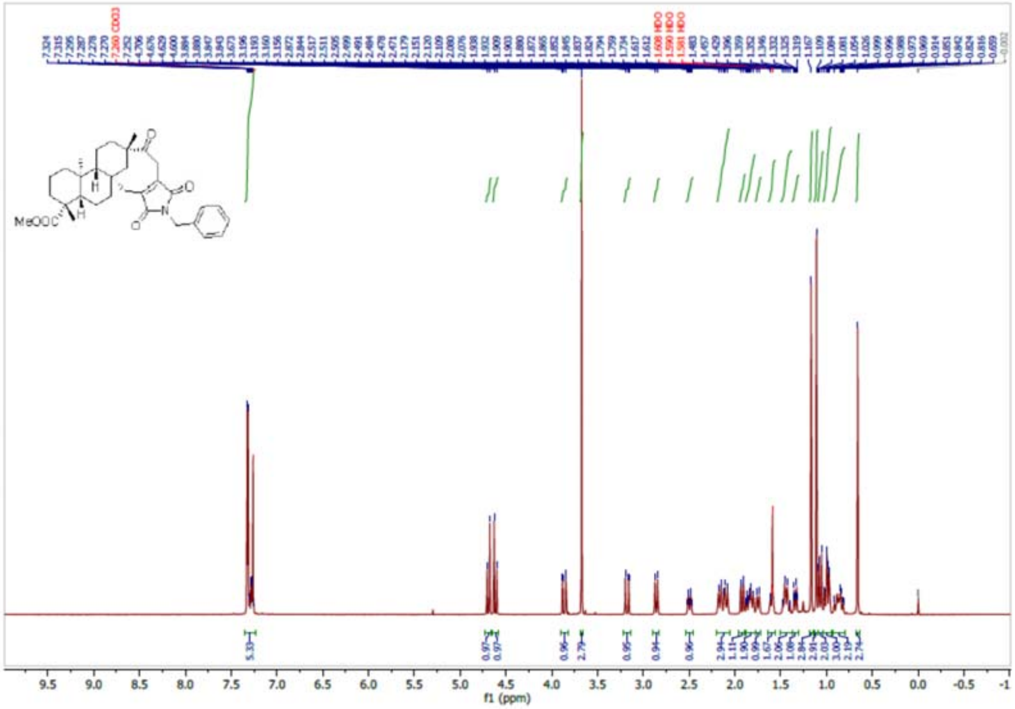

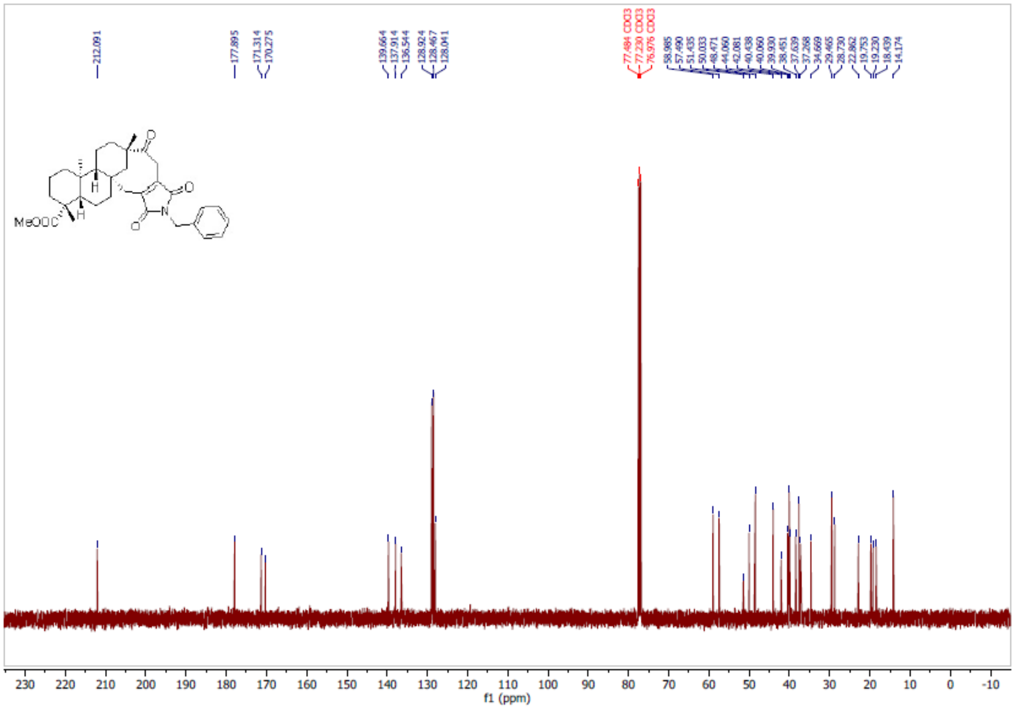

Supplementary Figure 180. ^{1}H and ^{13}C spectra of **47ad**.

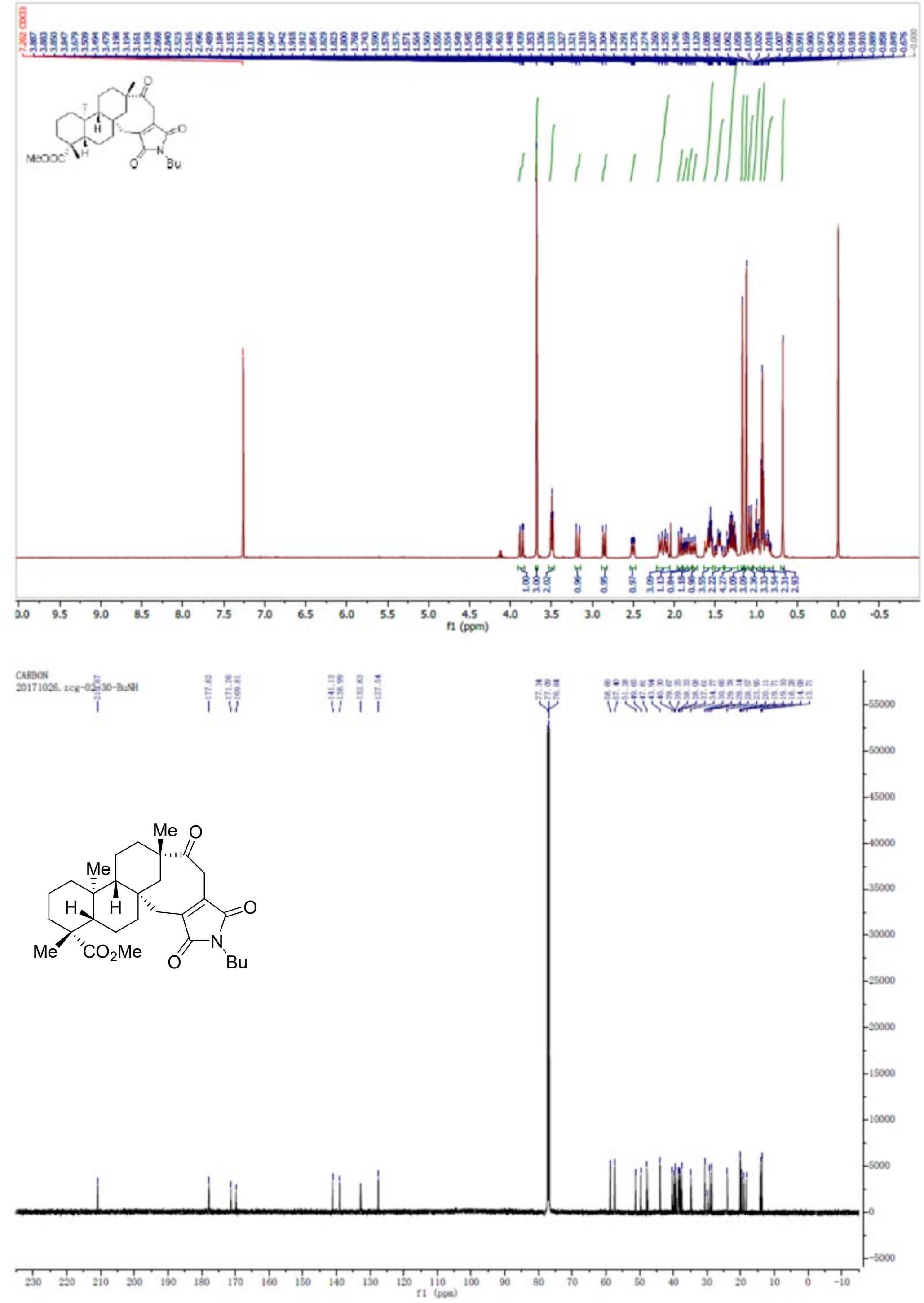

Supplementary Figure 181. ^{1}H and ^{13} C spectra of **47ae**.

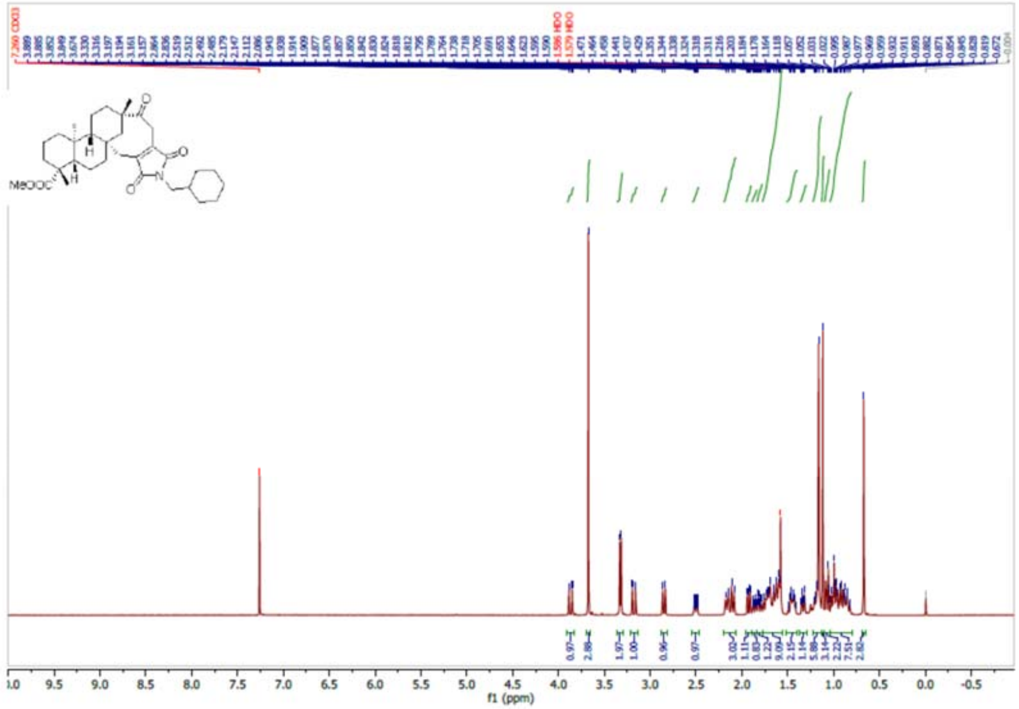

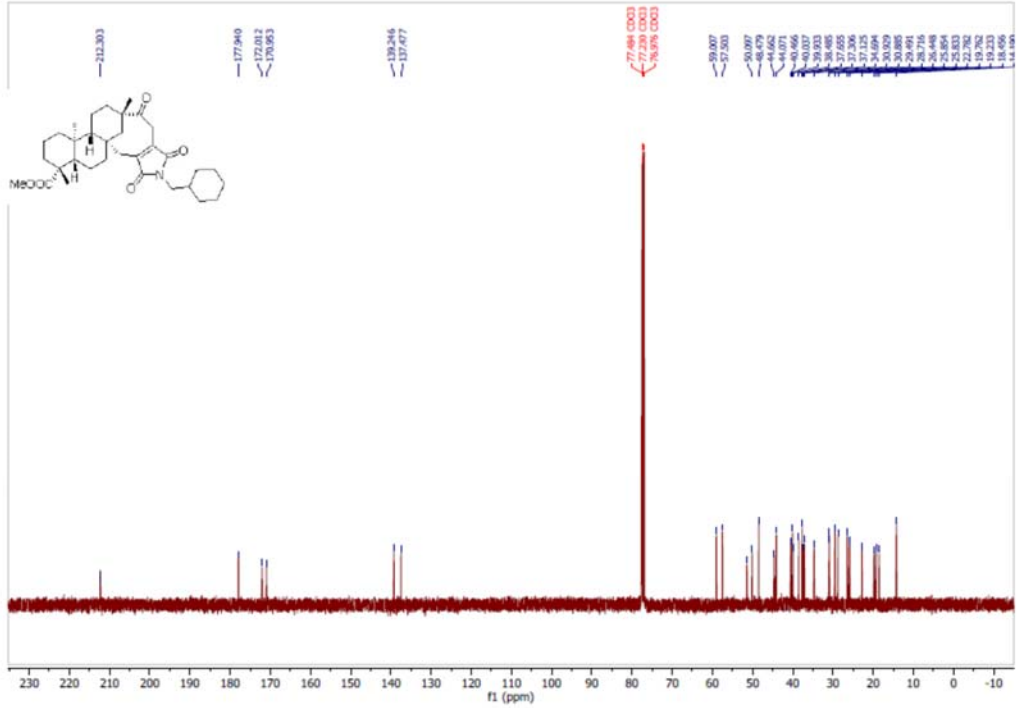

Supplementary Figure 182. ^{1}H and ^{13}C spectra of **47af**.

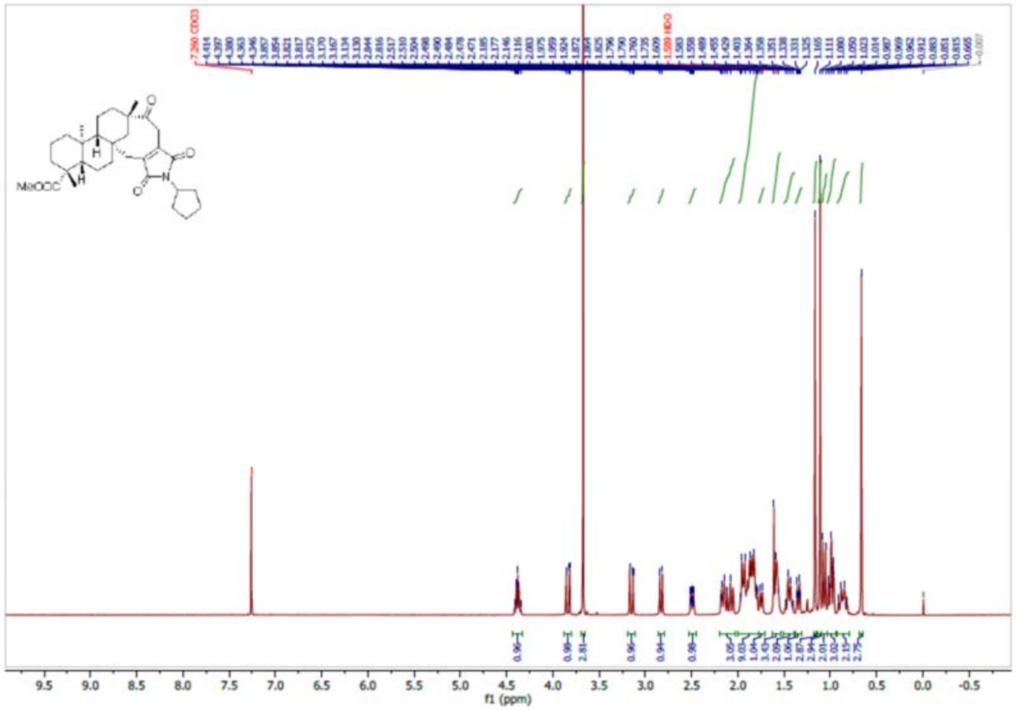

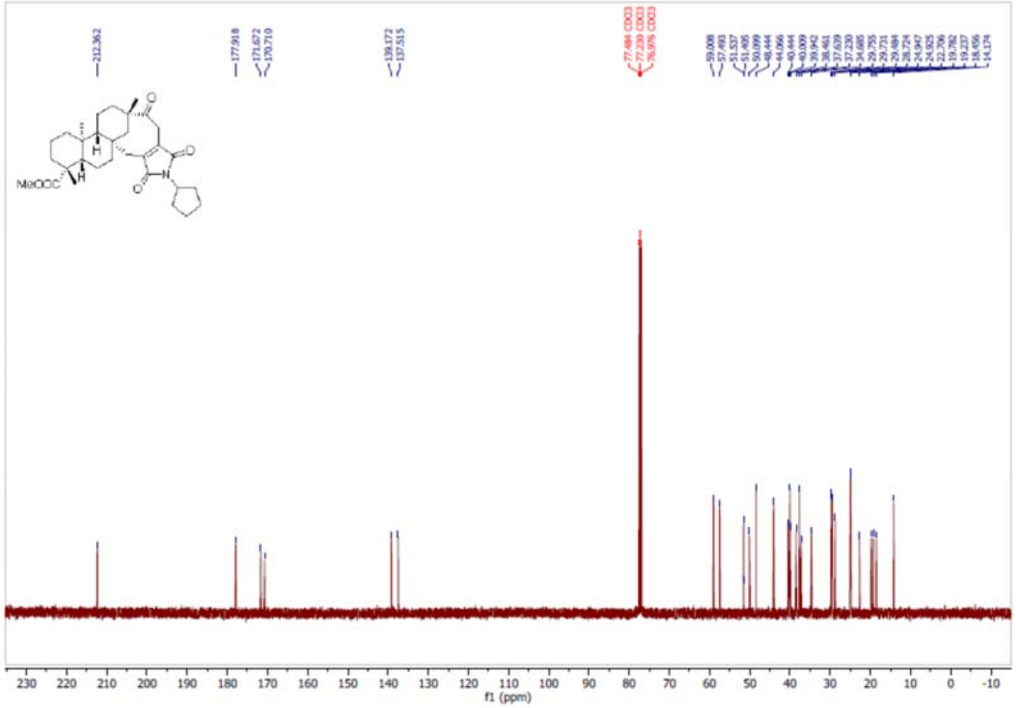

Supplementary Figure 183. ^{1}H and ^{13} C spectra of **47ag**.

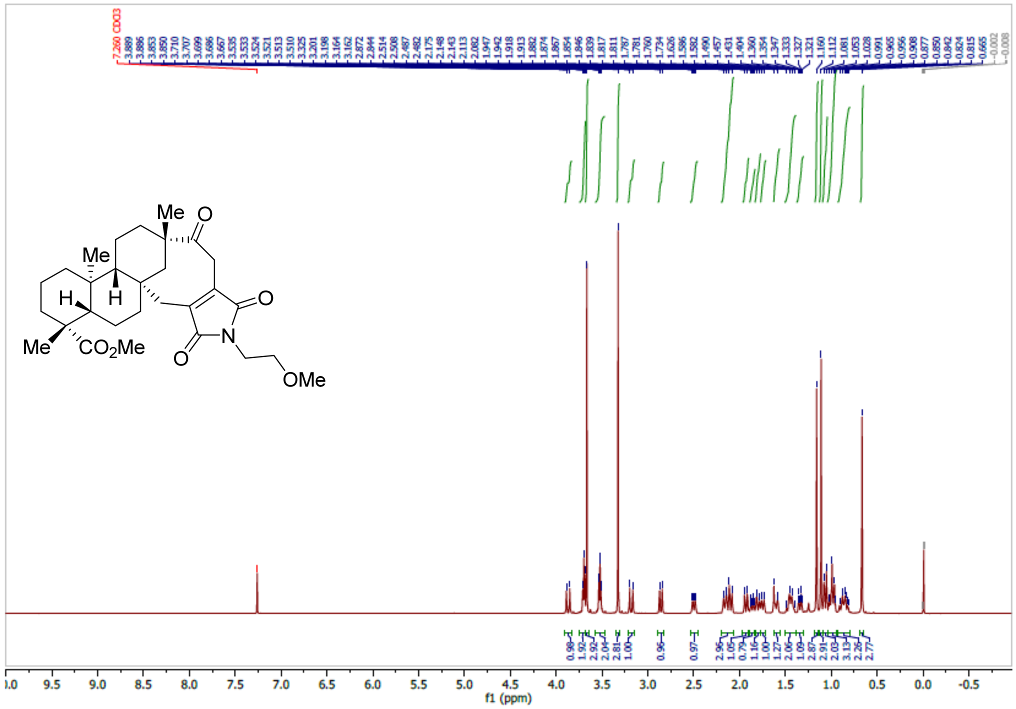

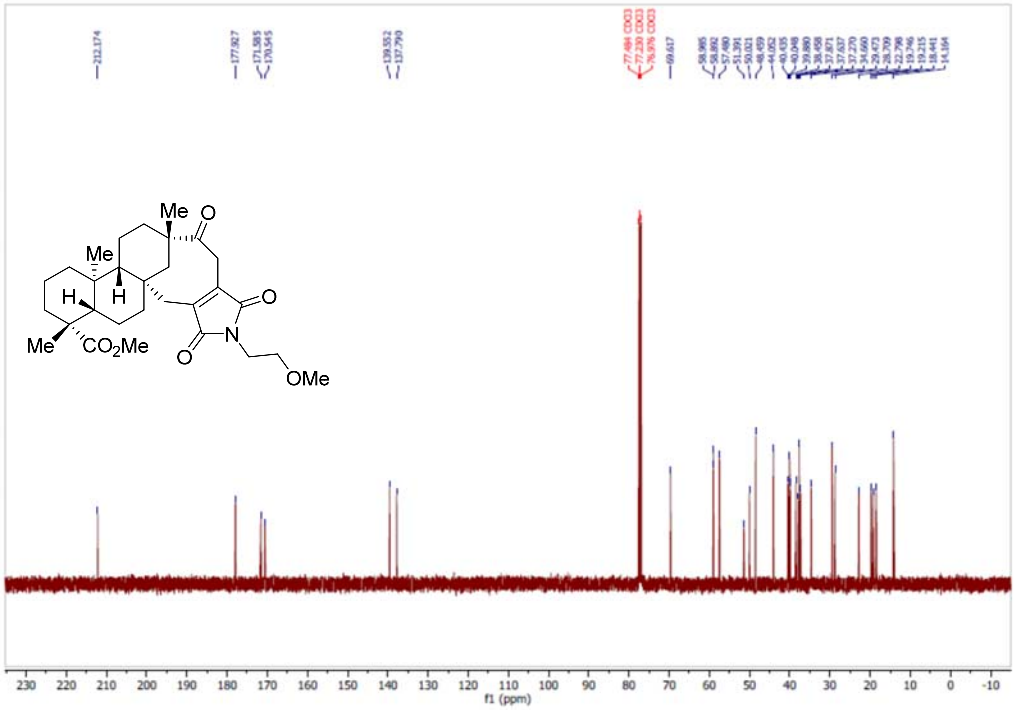

Supplementary Figure 184. ^{1}H and ^{13}C spectra of **47ah**.

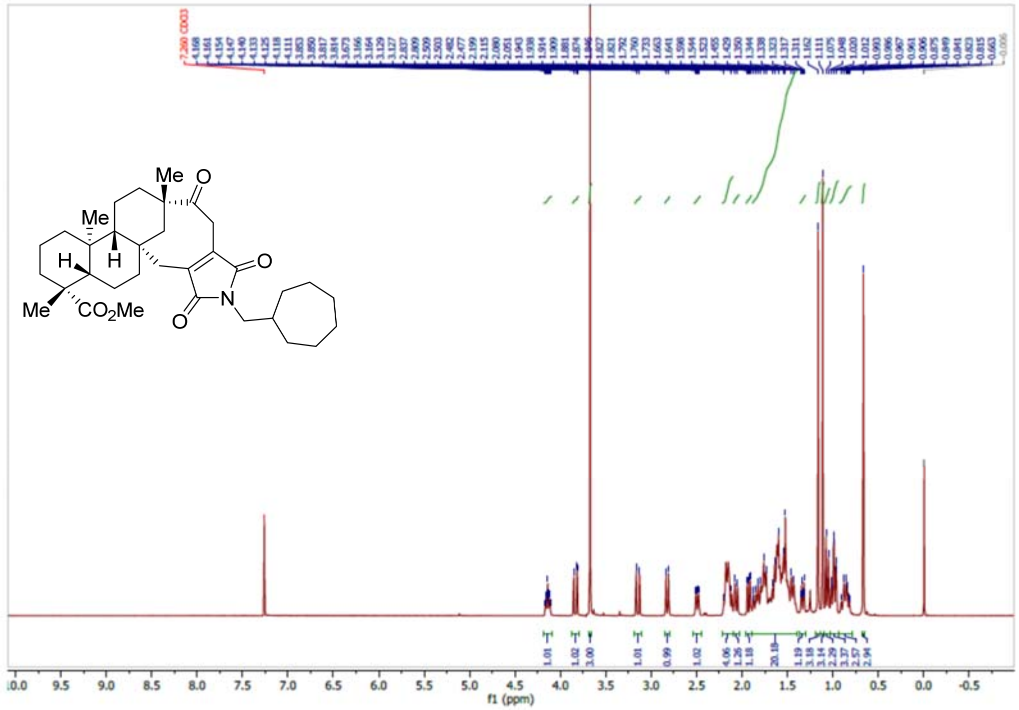

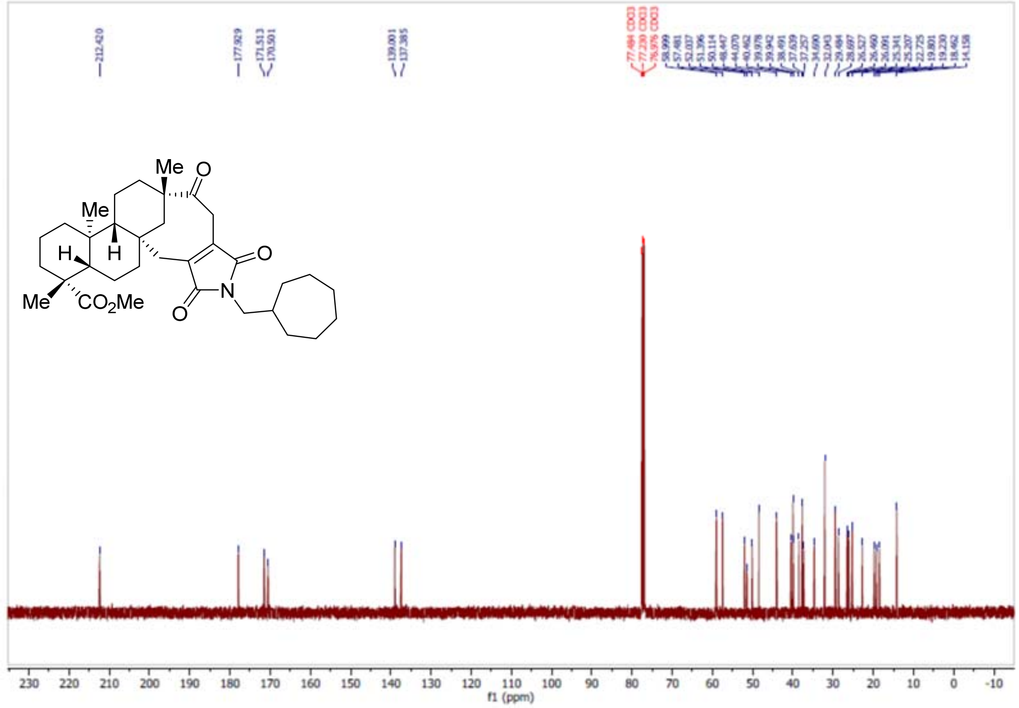

Supplementary Figure 185. ^{1}H and ^{13}C spectra of **47ai**.

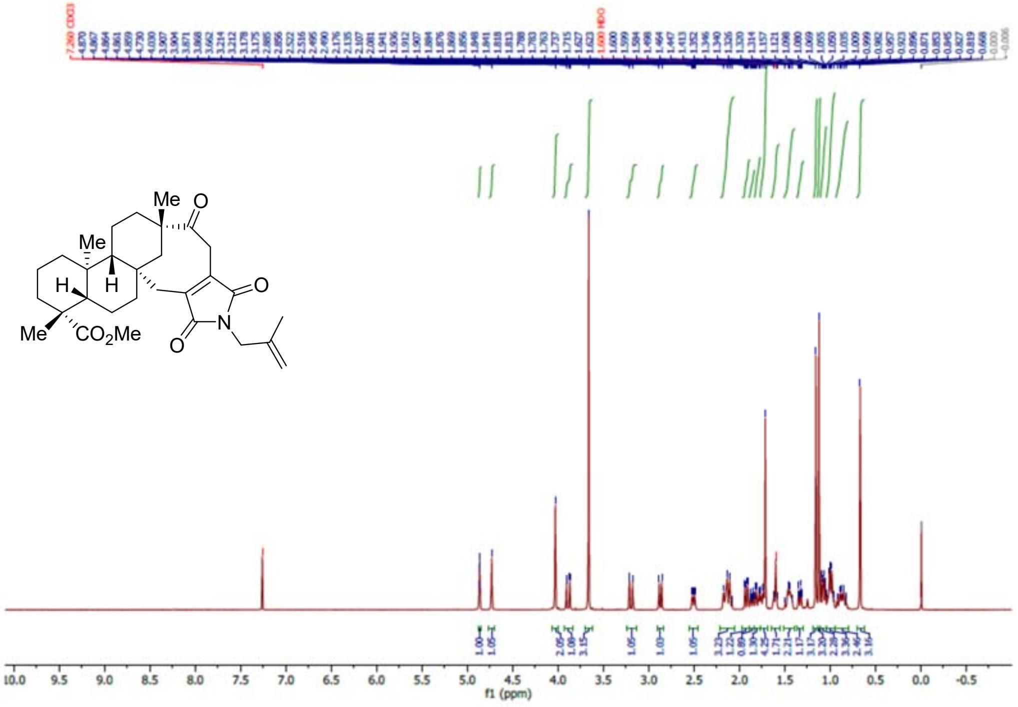

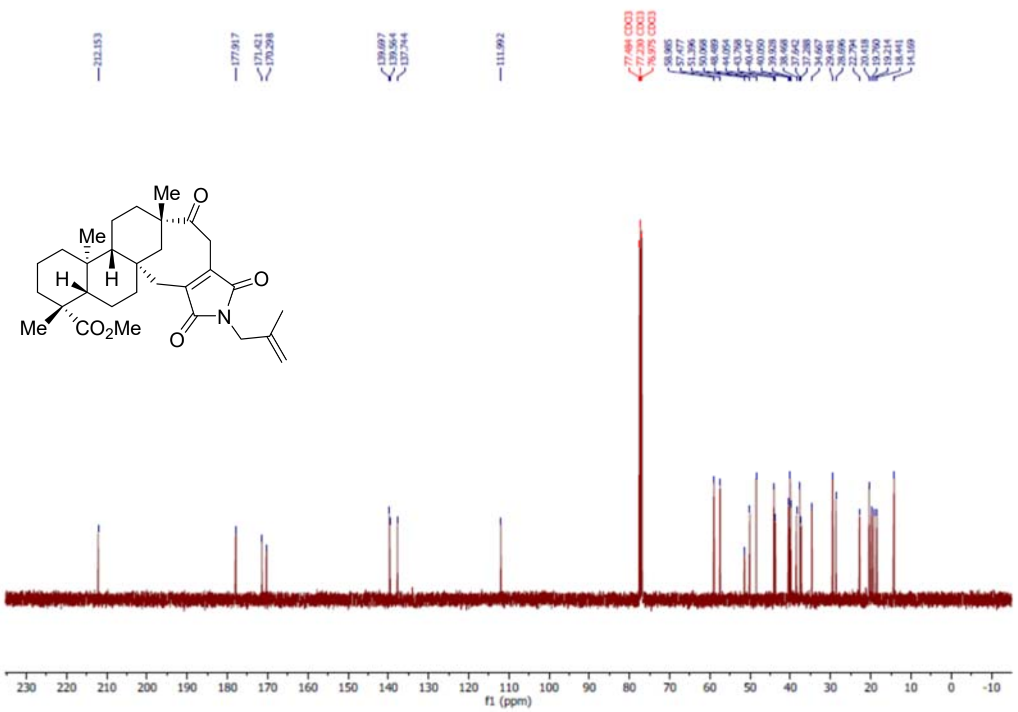

Supplementary Figure 186. ^{1}H and ^{13}C spectra of **47aj**.

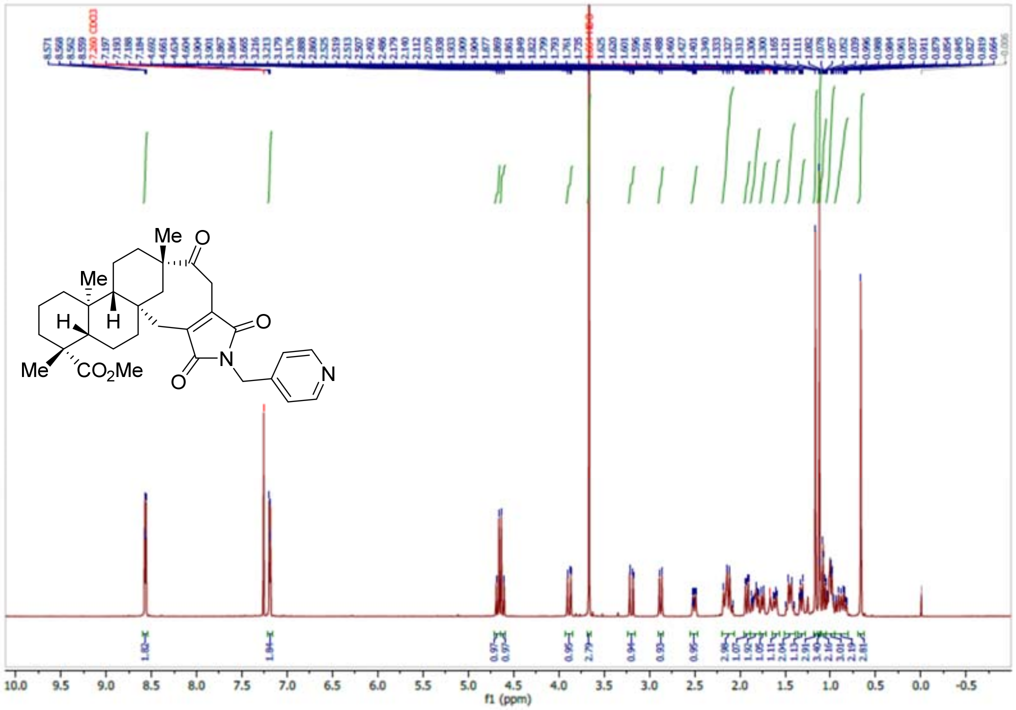

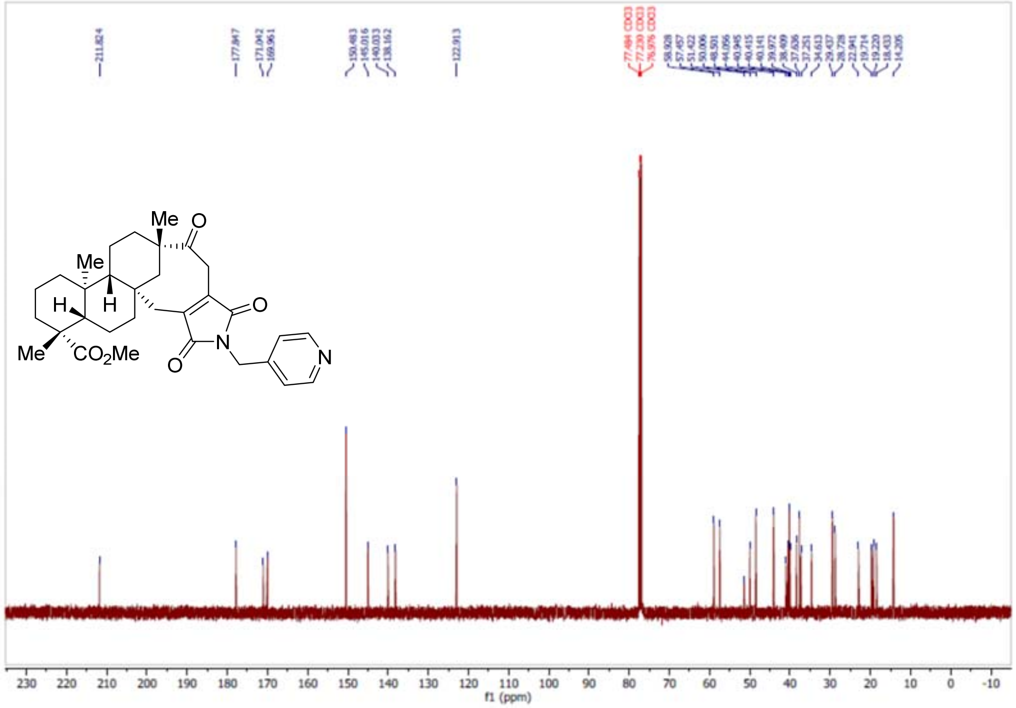

Supplementary Figure 187. ^{1}H and ^{13}C spectra of **47ak**.

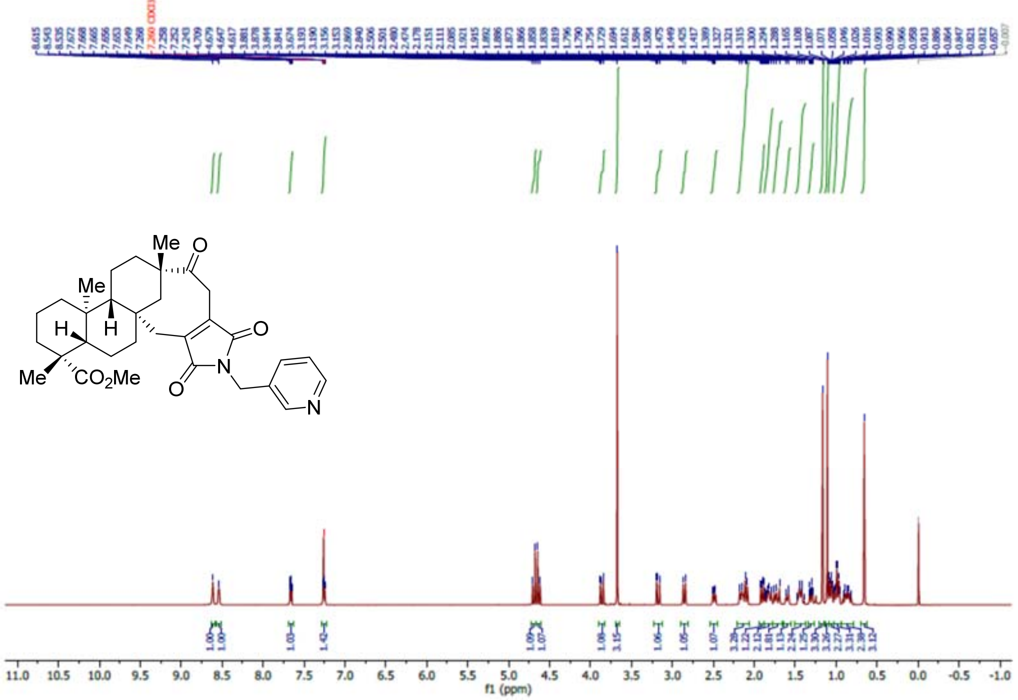

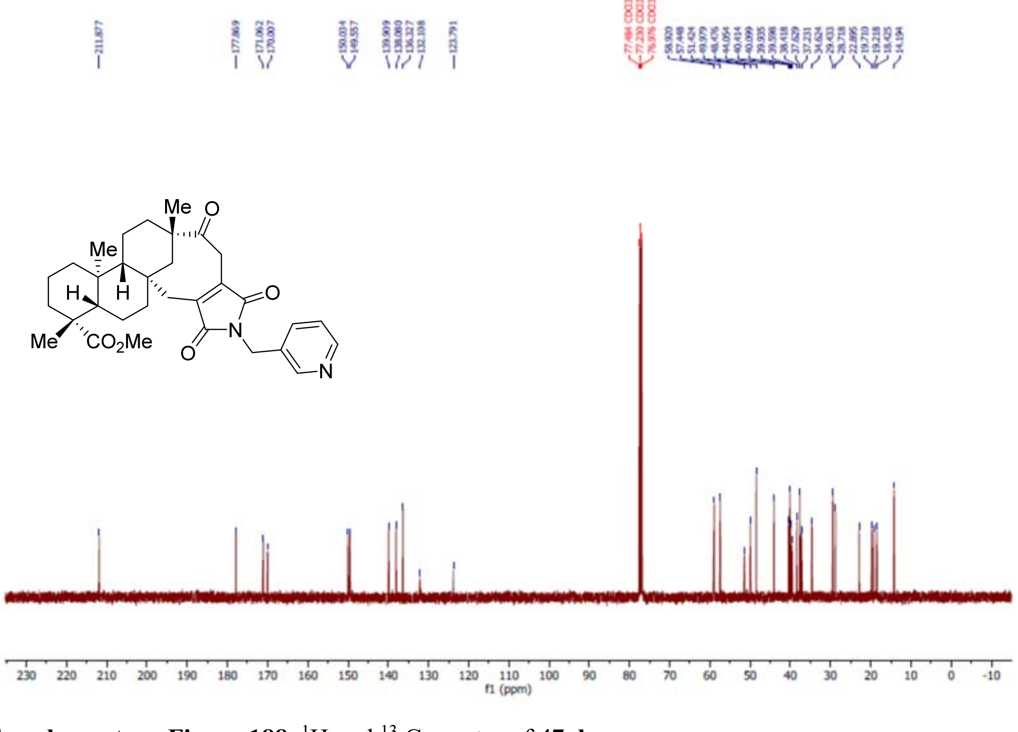

Supplementary Figure 188. ^{1}H and ^{13}C spectra of **47al**.

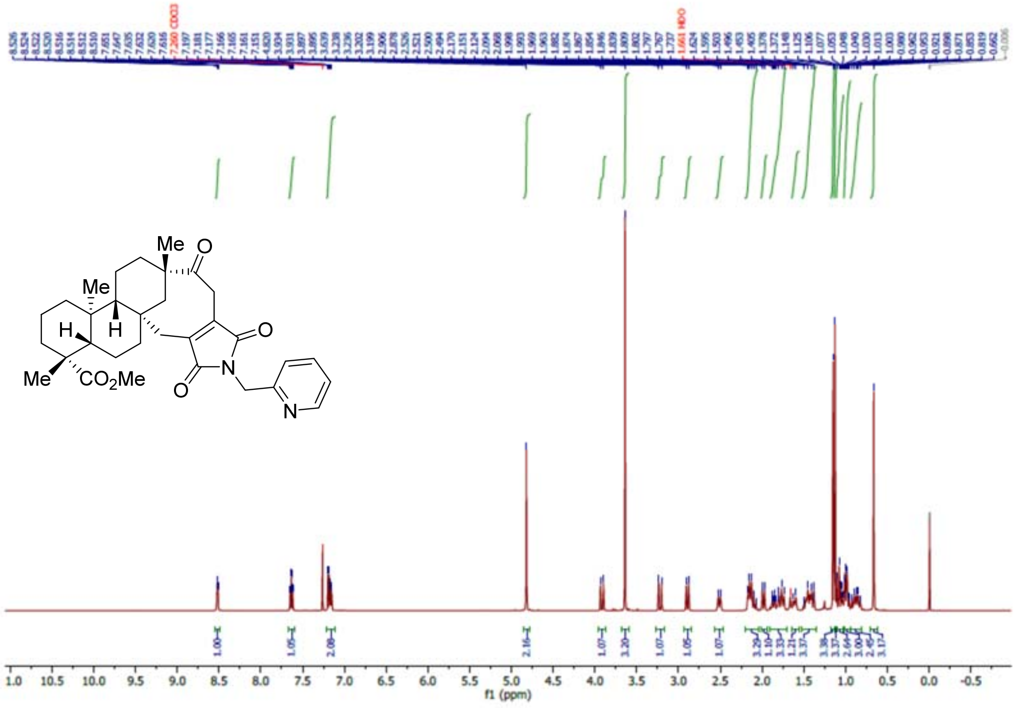

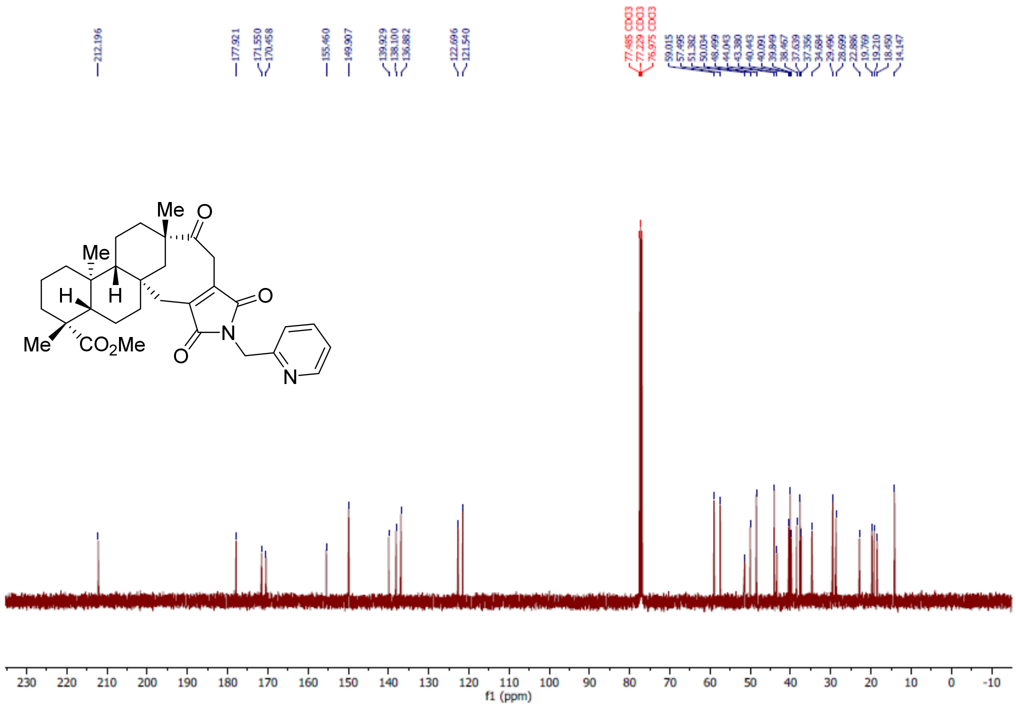

Supplementary Figure 189. ^{1}H and ^{13}C spectra of **47am**.

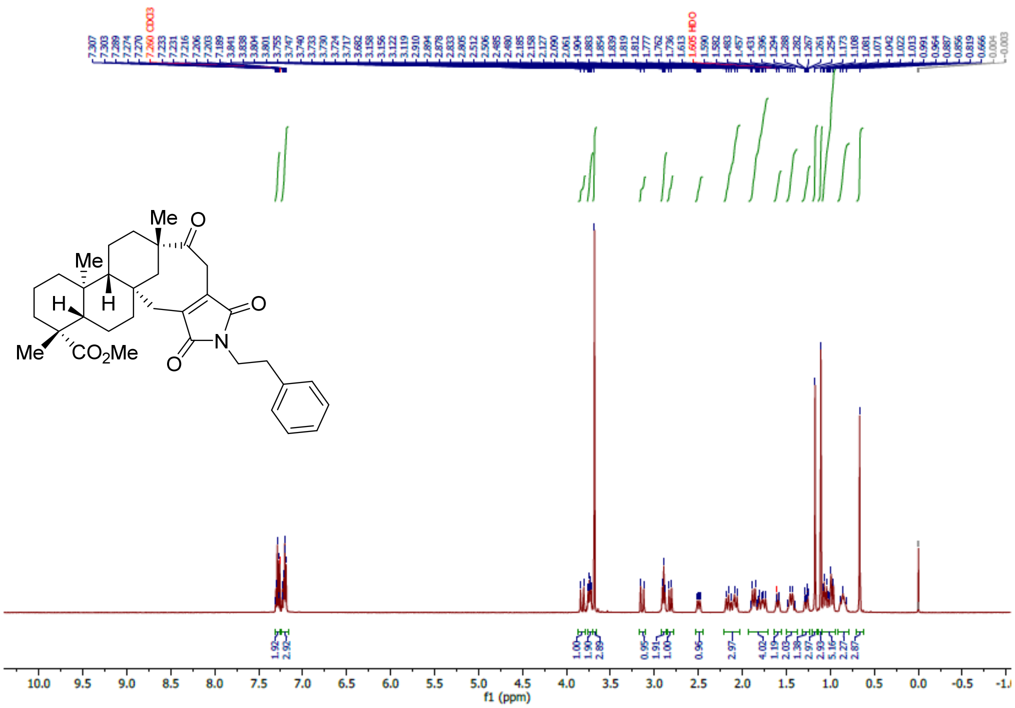

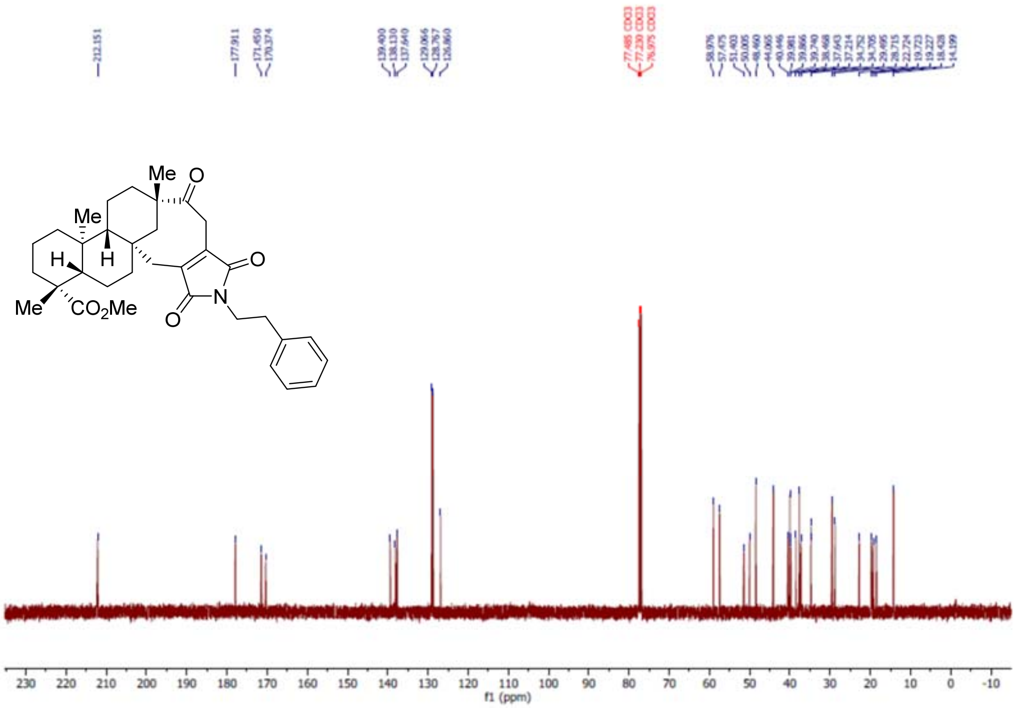

Supplementary Figure 190. ^{1}H and ^{13} C spectra of **47an**.

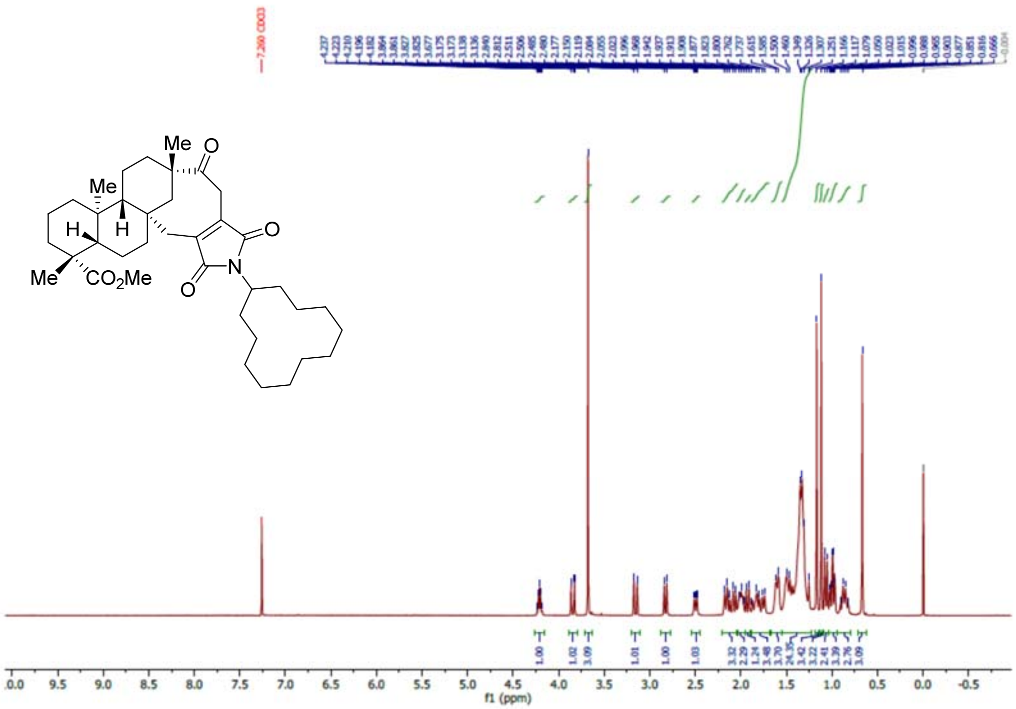

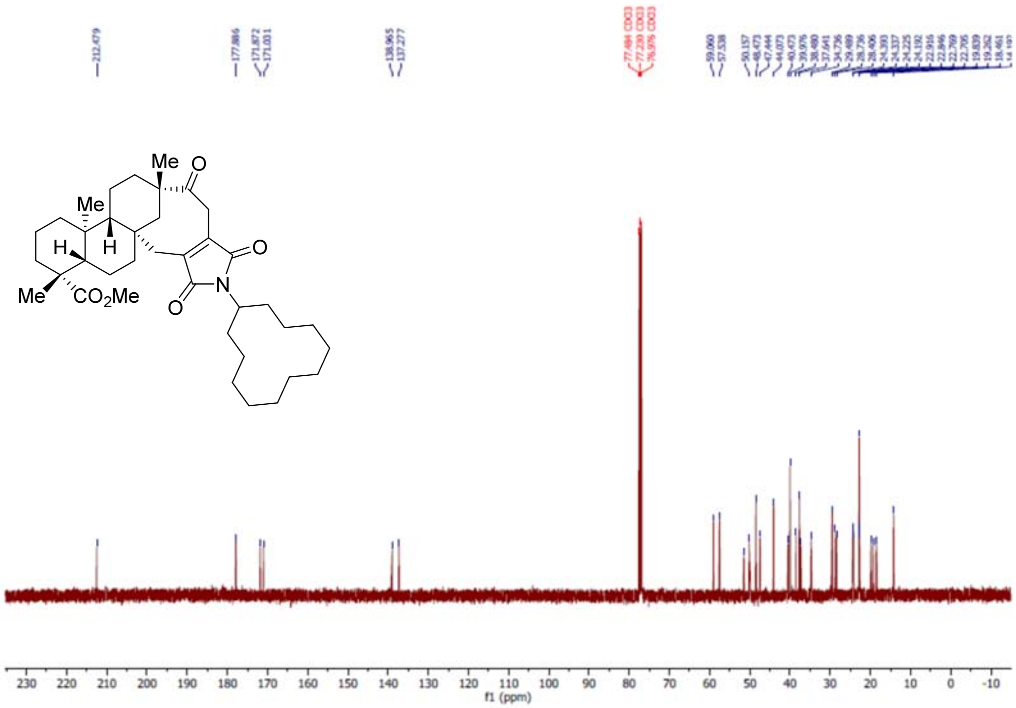

Supplementary Figure 191. ^{1}H and ^{13}C spectra of **47ao**.

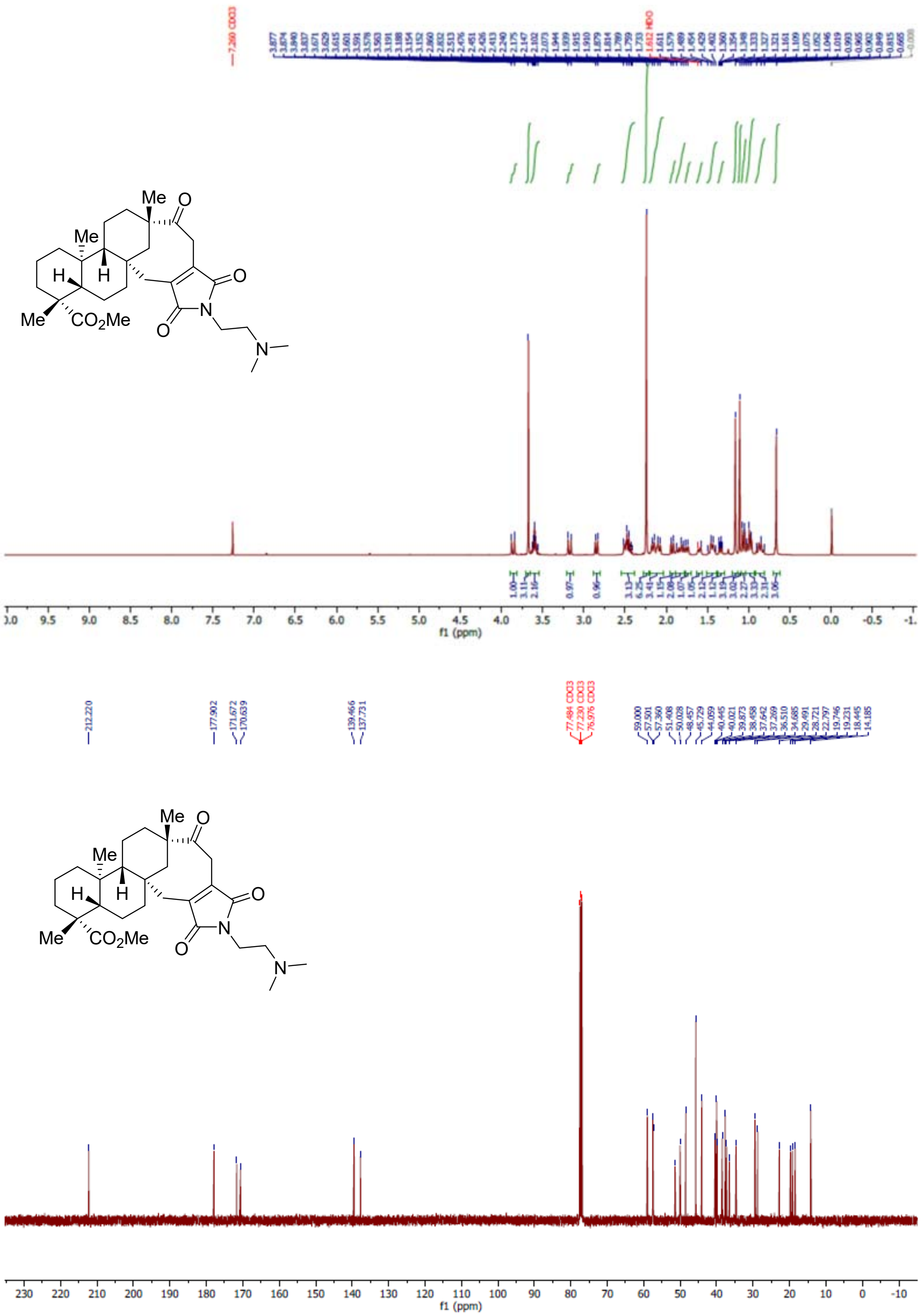

Supplementary Figure 192. ¹H and ¹³ C spectra of **47ap**.

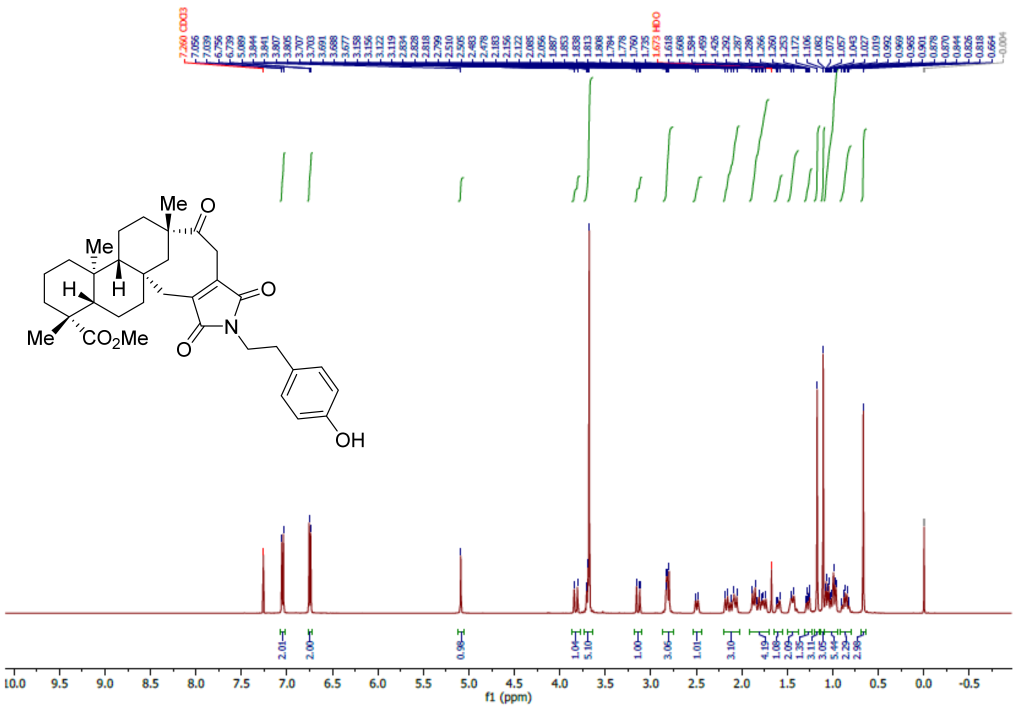

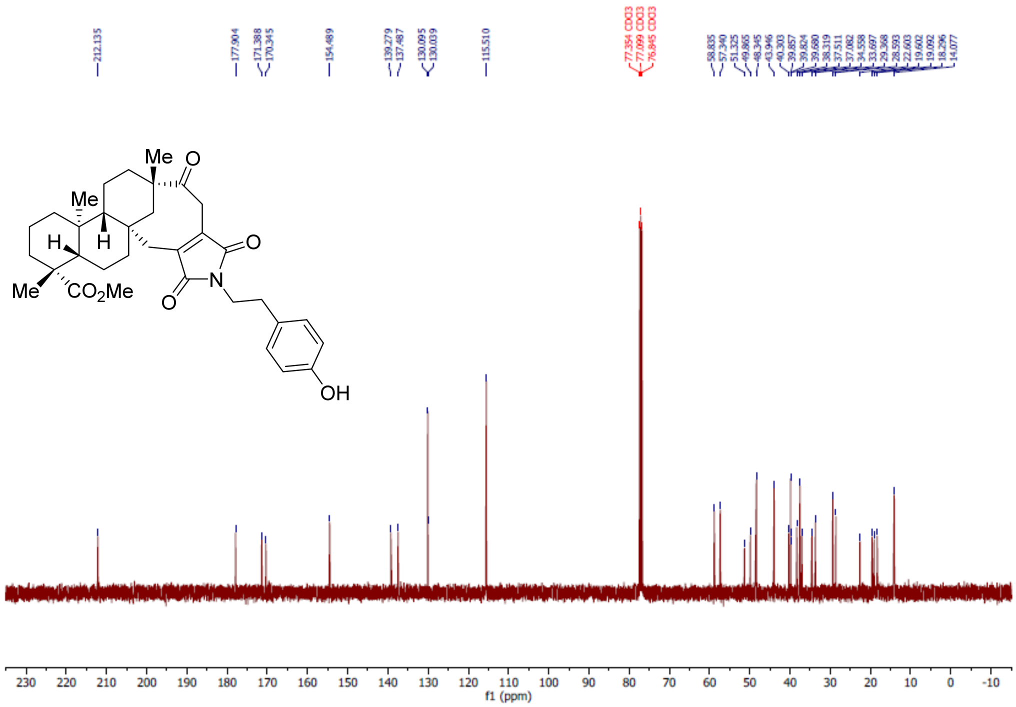

Supplementary Figure 193. ^{1}H and ^{13}C spectra of **47aq**.

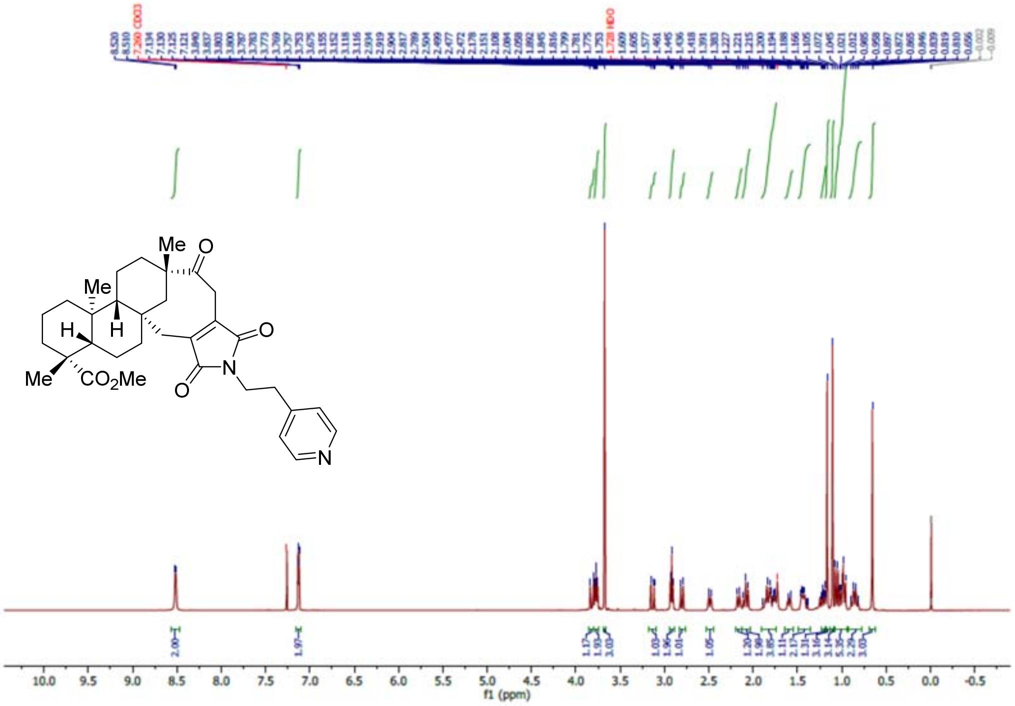

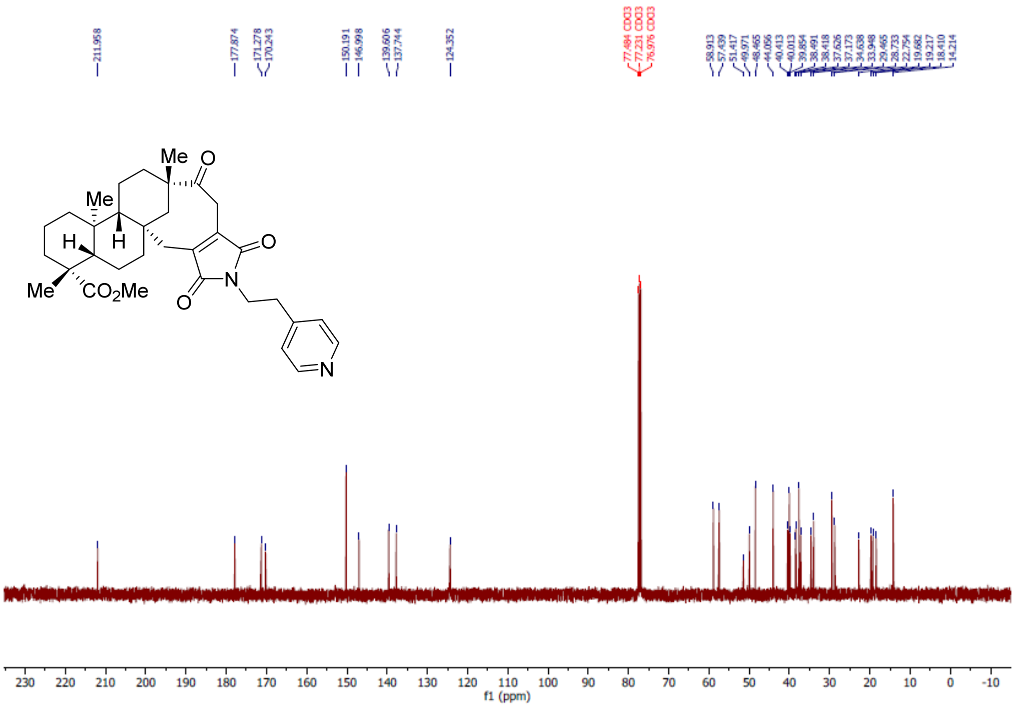

Supplementary Figure 194. ^{1}H and ^{13}C spectra of **47ar**.

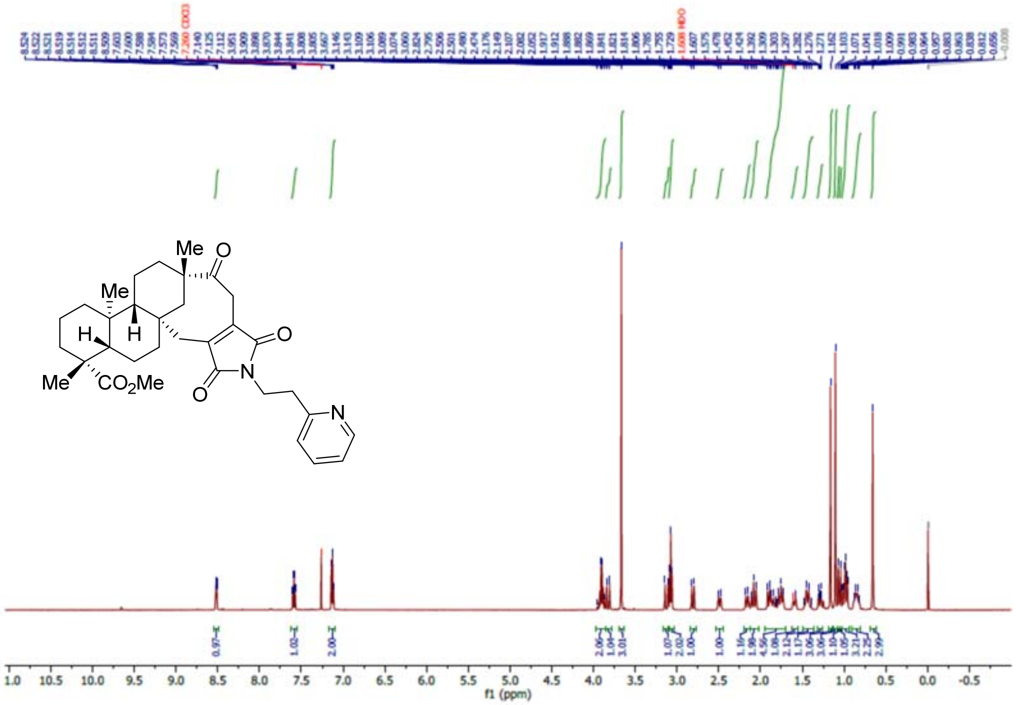

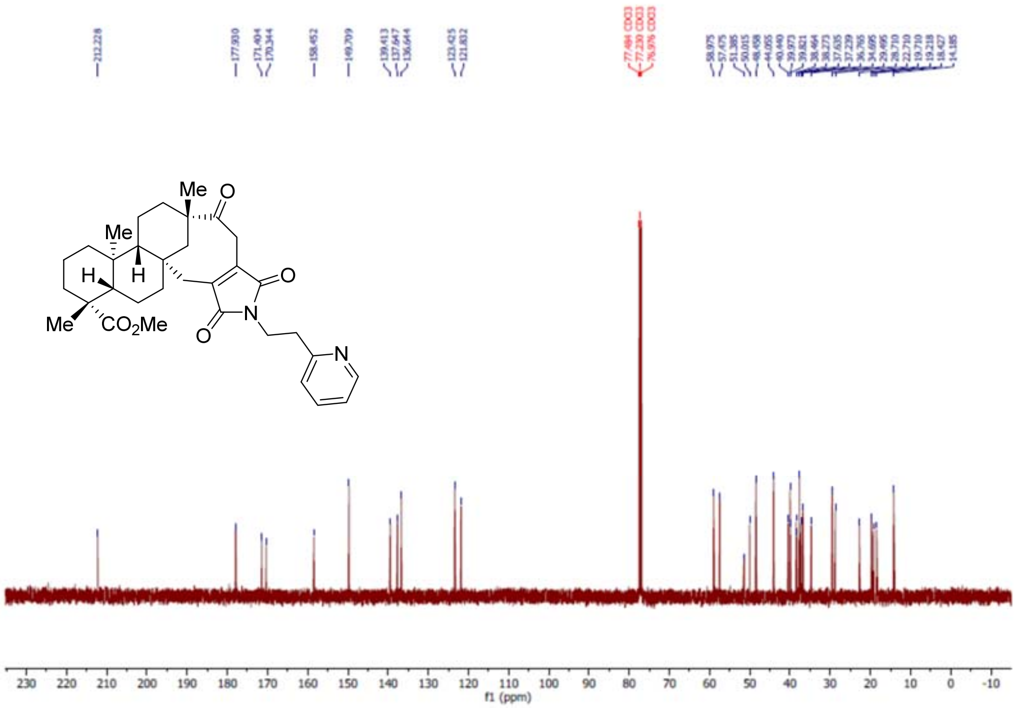

Supplementary Figure 195. ^{1}H and ^{13}C spectra of **47as**.

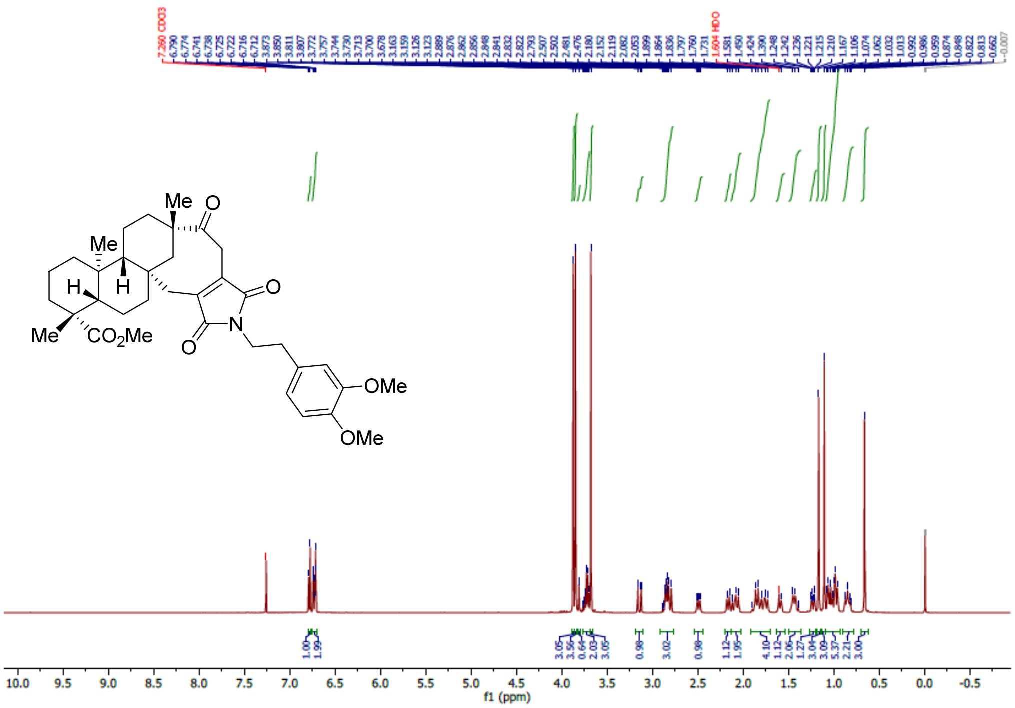

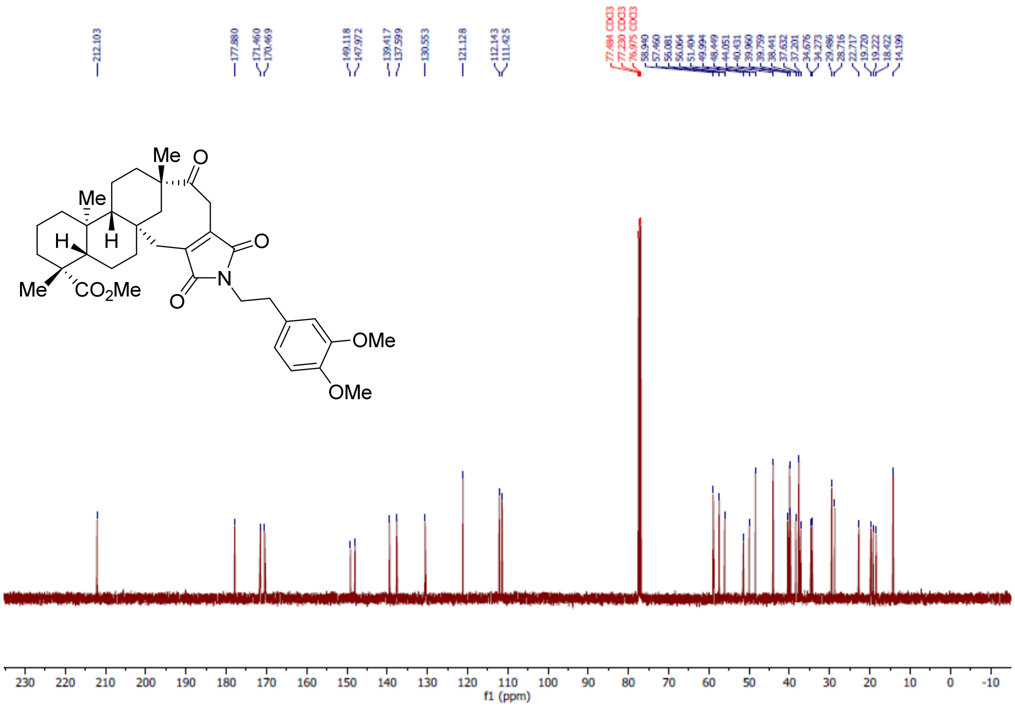

Supplementary Figure 196. ^{1}H and ^{13} C spectra of **47at**.

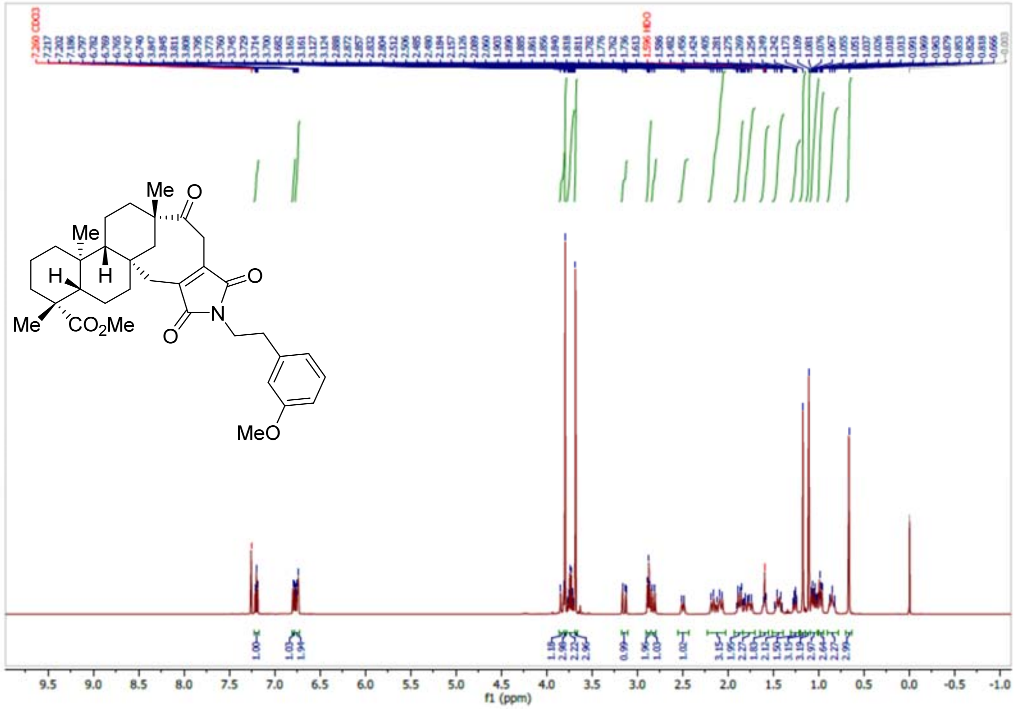

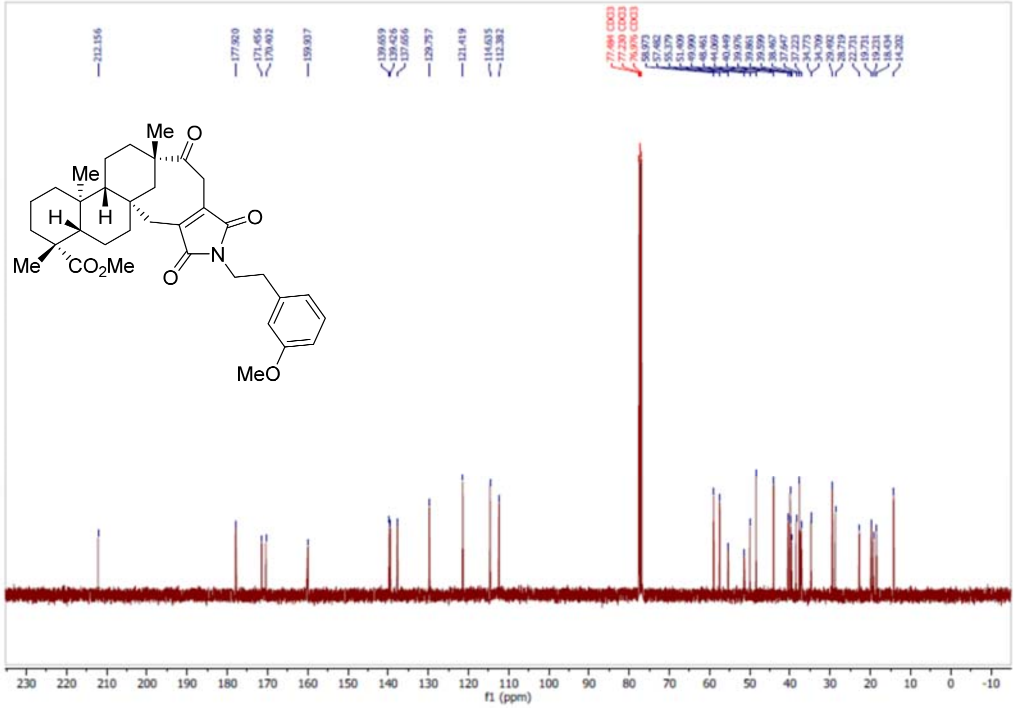

Supplementary Figure 197. ^{1}H and ^{13}C spectra of **47au**.

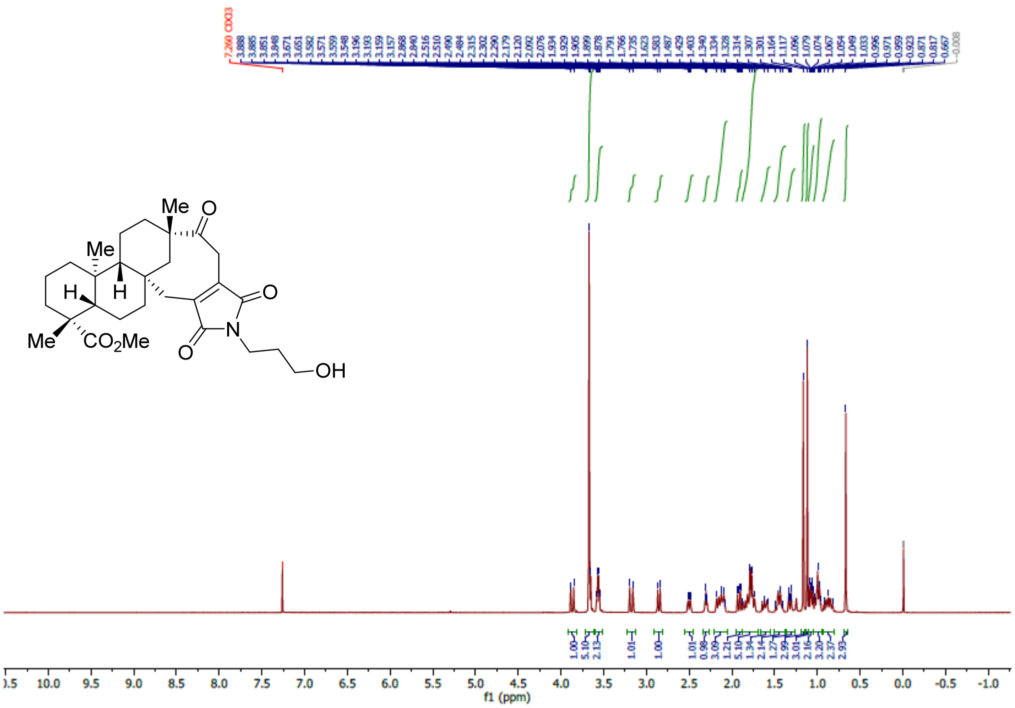

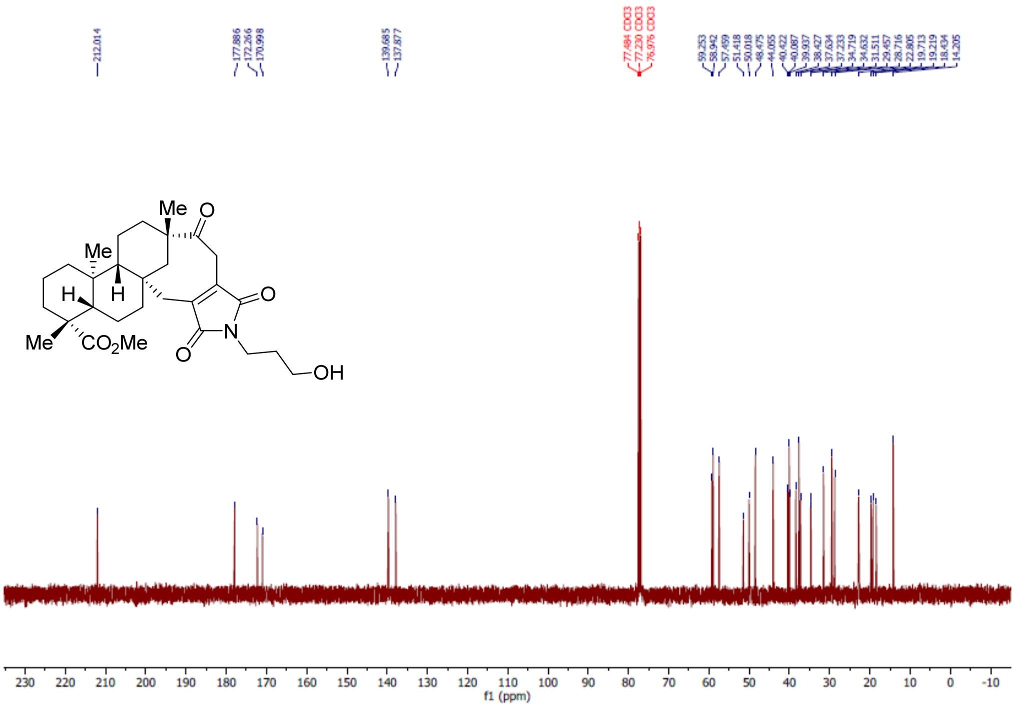

Supplementary Figure 198. ^{1}H and ^{13}C spectra of **47av**.

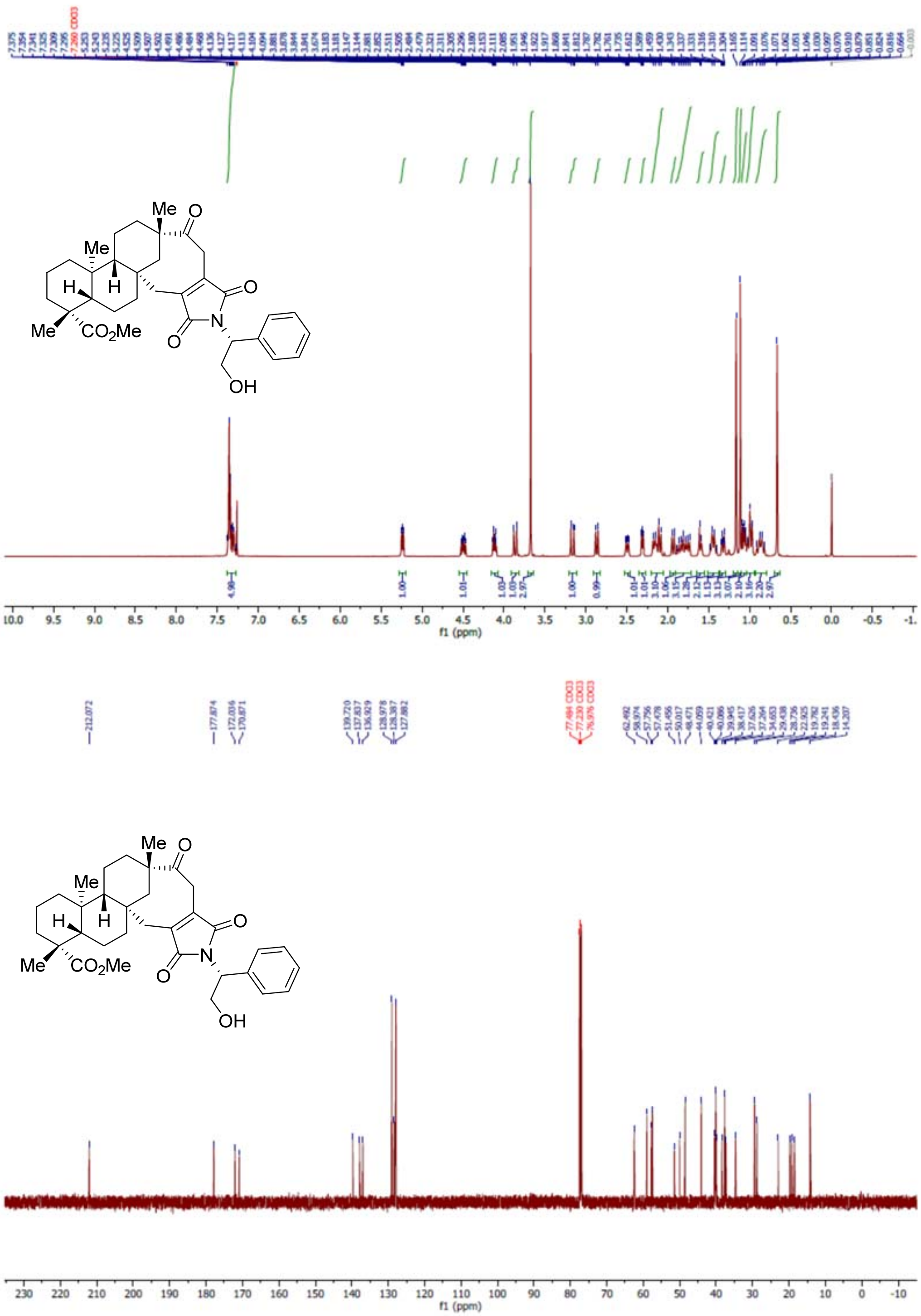

Supplementary Figure 199. ^{1}H and ^{13} C spectra of **47aw**.

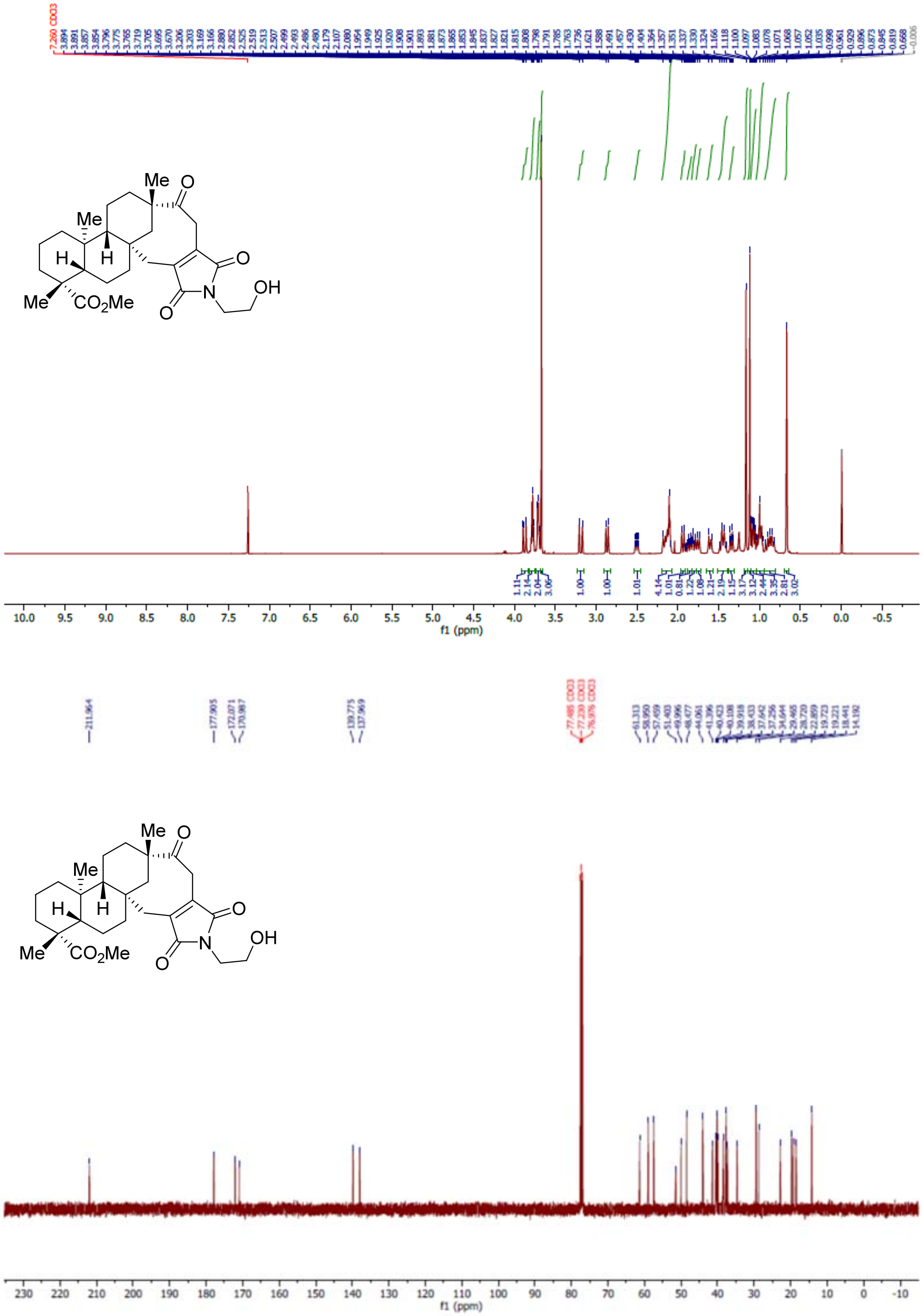

Supplementary Figure 200. ^{1}H and ^{13}C spectra of **47ax**.

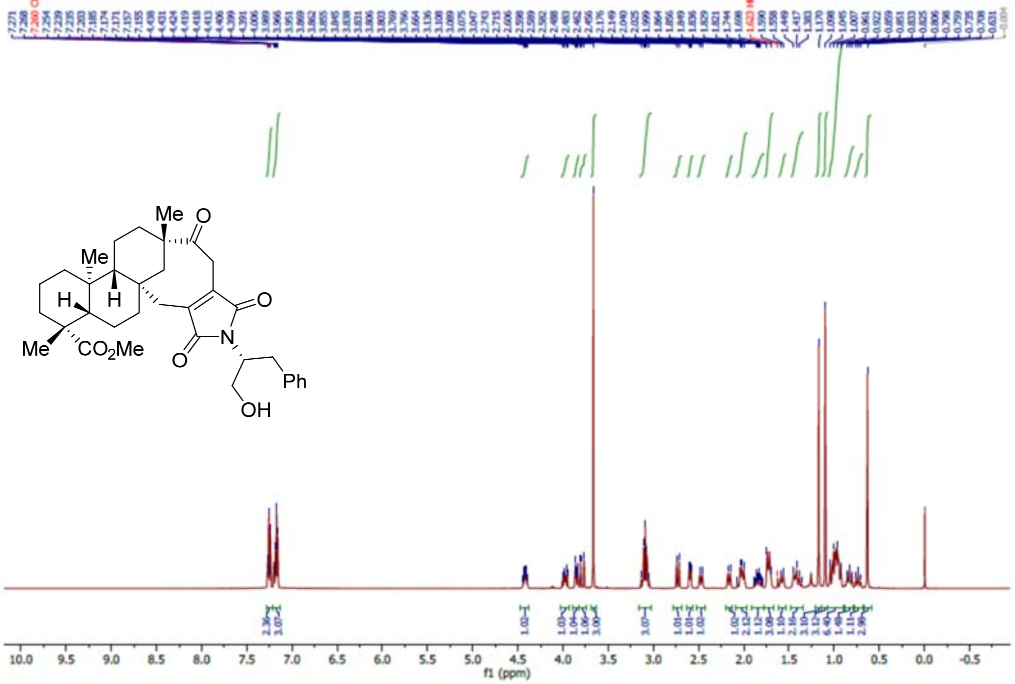

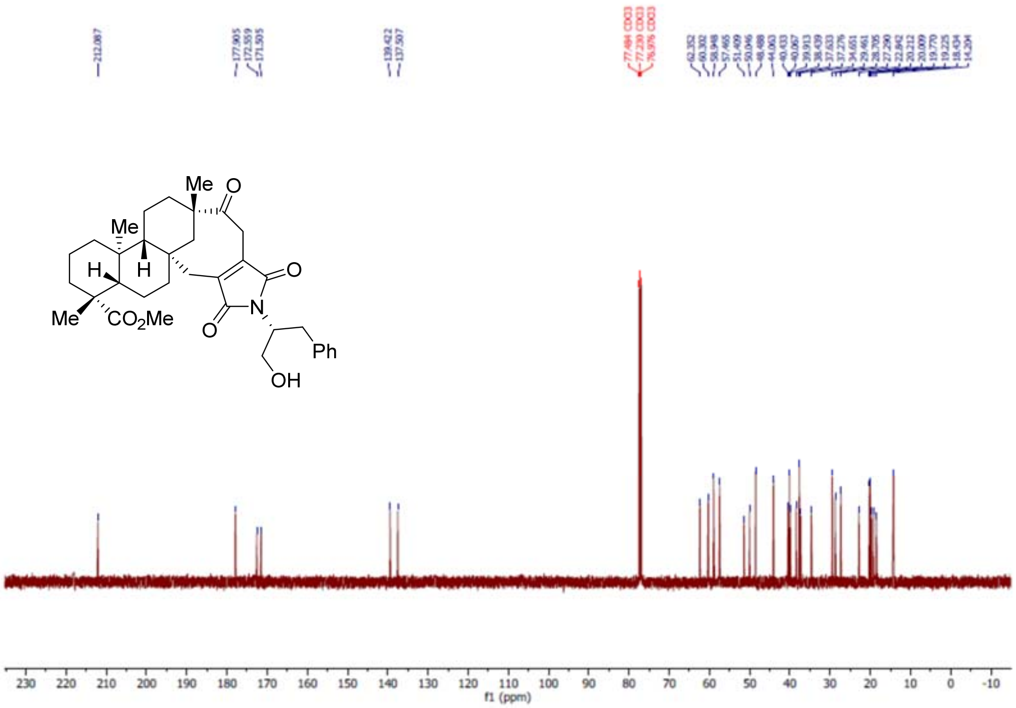

Supplementary Figure 201. ^{1}H and ^{13}C spectra of **47ay**.

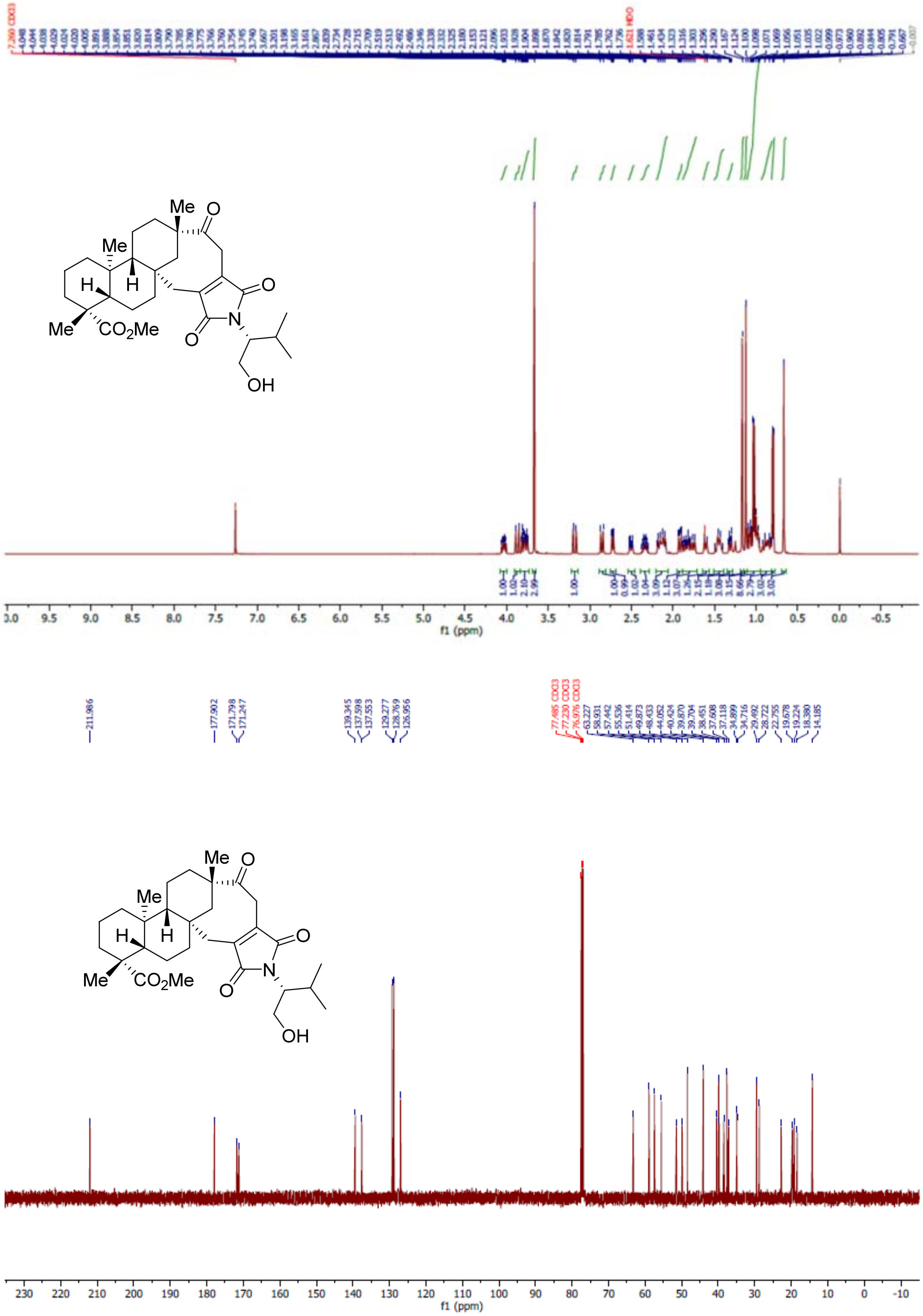

Supplementary Figure 202. ^{1}H and ^{13}C spectra of **47az**.

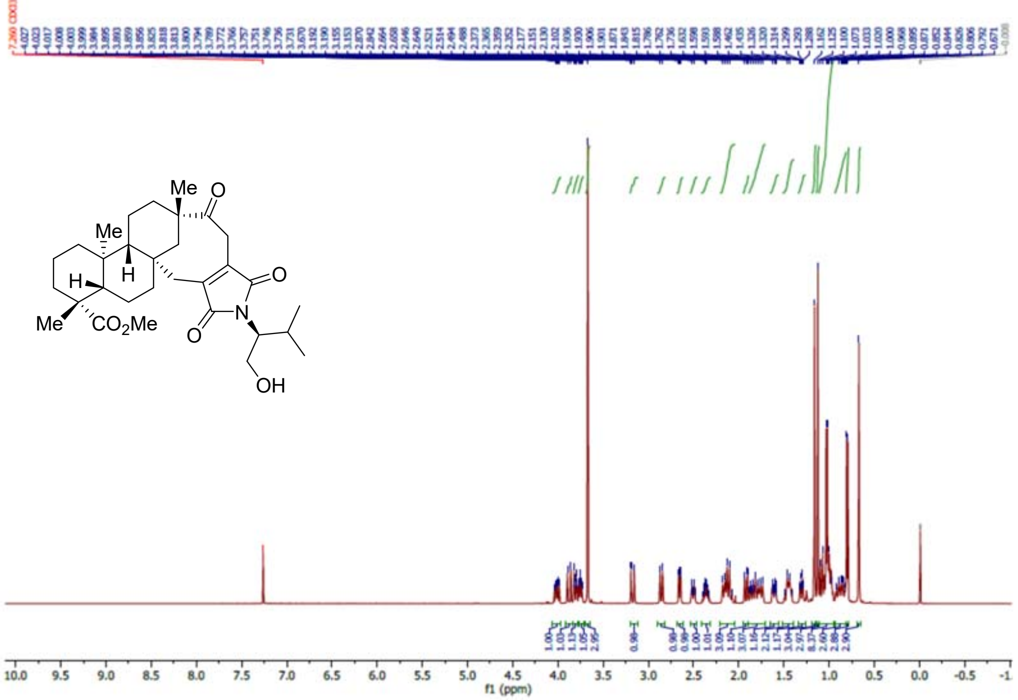

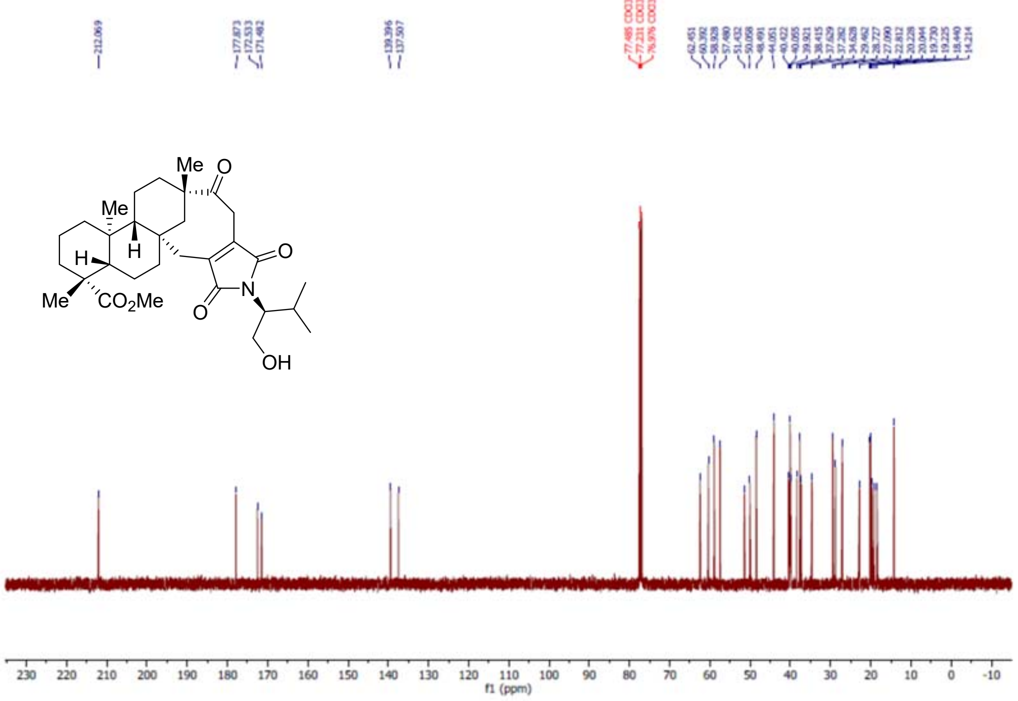

Supplementary Figure 203. ^{1}H and ^{13} C spectra of **47ba**.

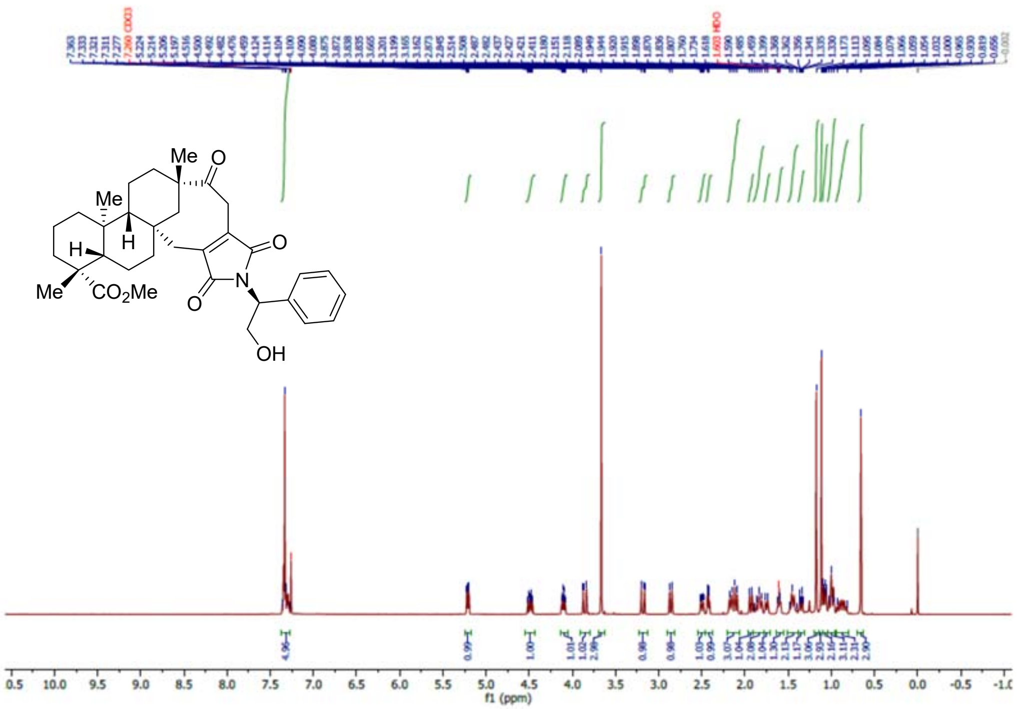

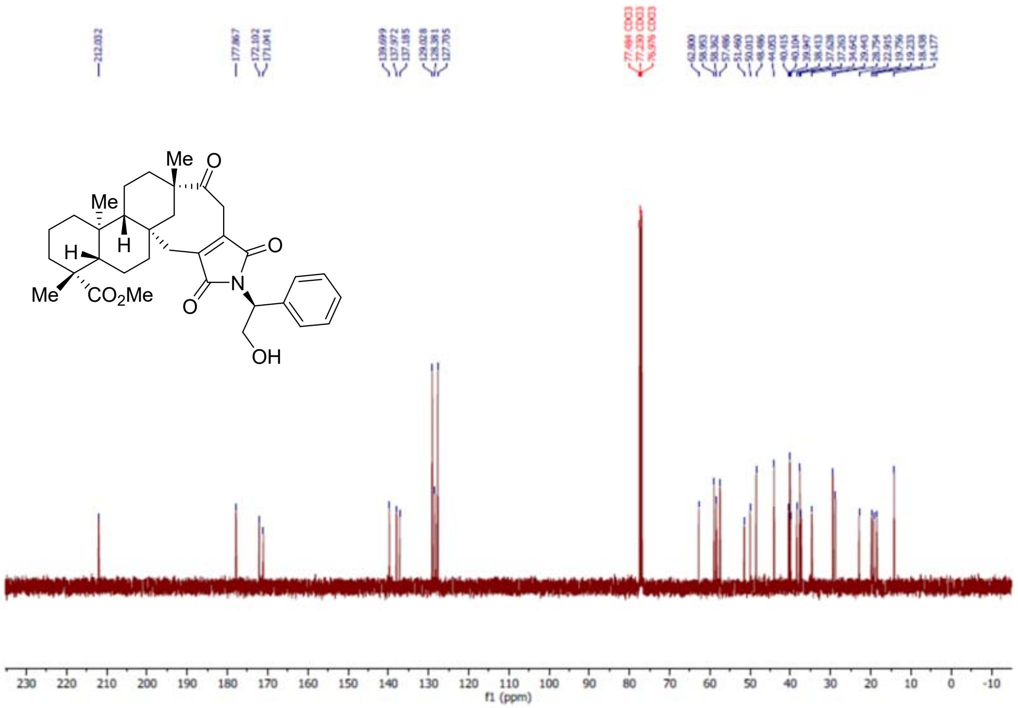

Supplementary Figure 204. ^{1}H and ^{13}C spectra of **47bb**.

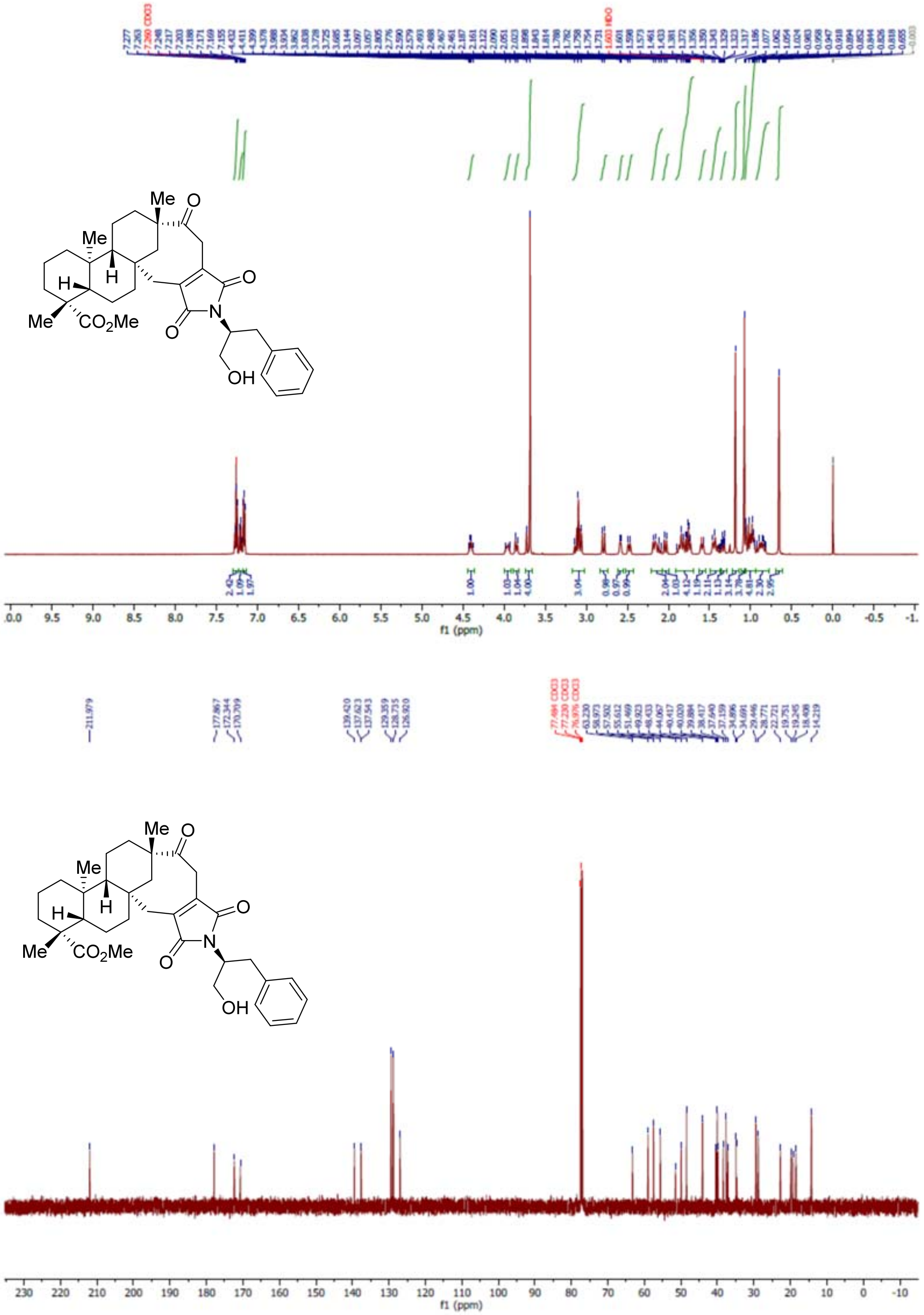

Supplementary Figure 205. ^{1}H and ^{13}C spectra of **47bc**.

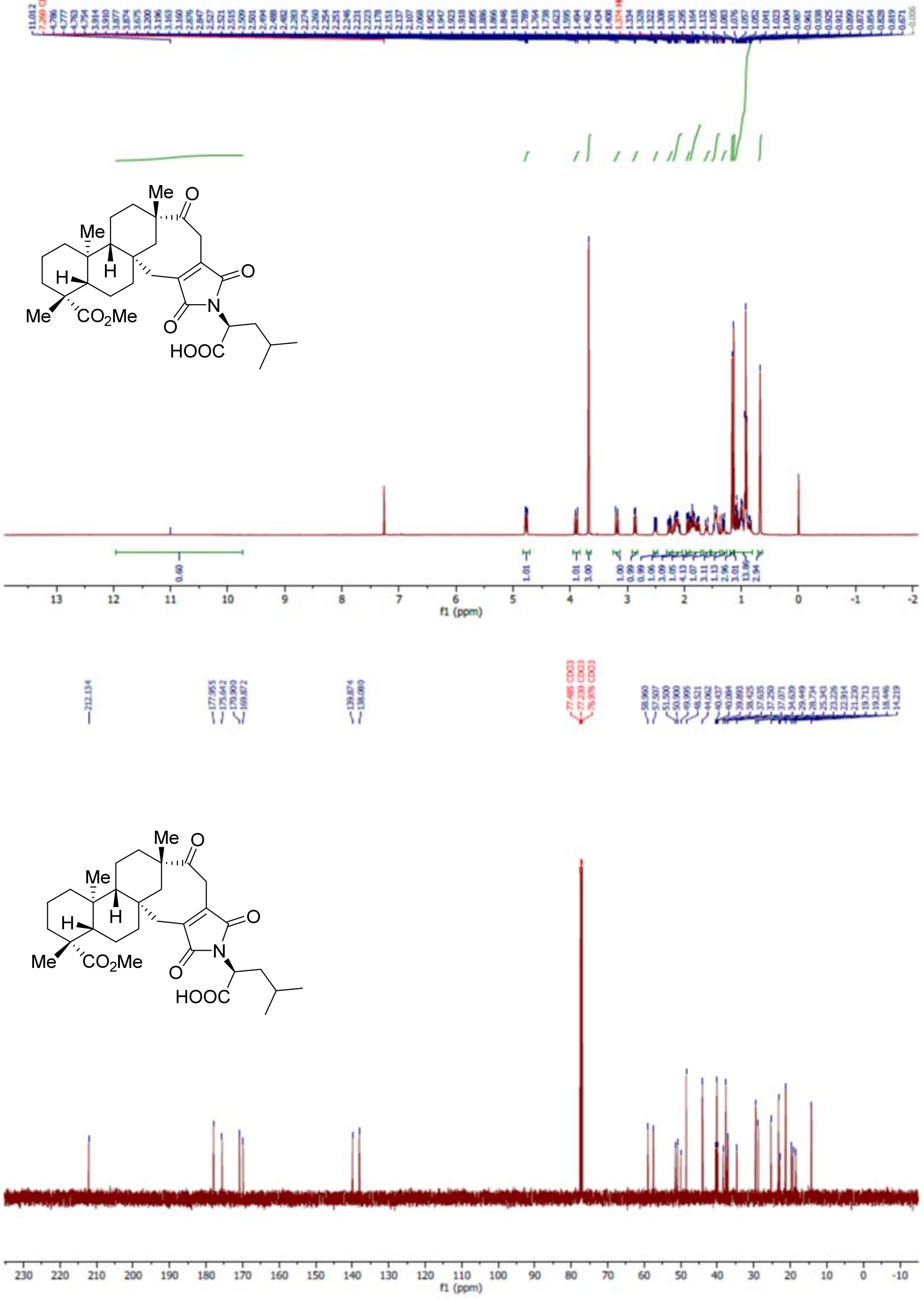

Supplementary Figure 206. ^{1}H and ^{13}C spectra of **47bd**.

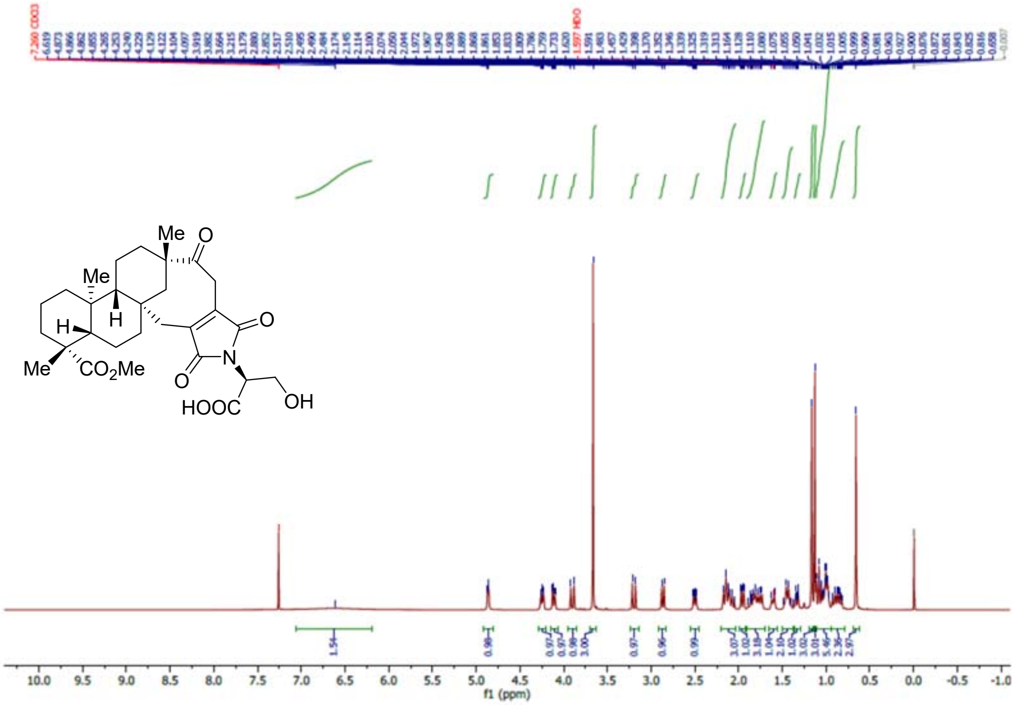

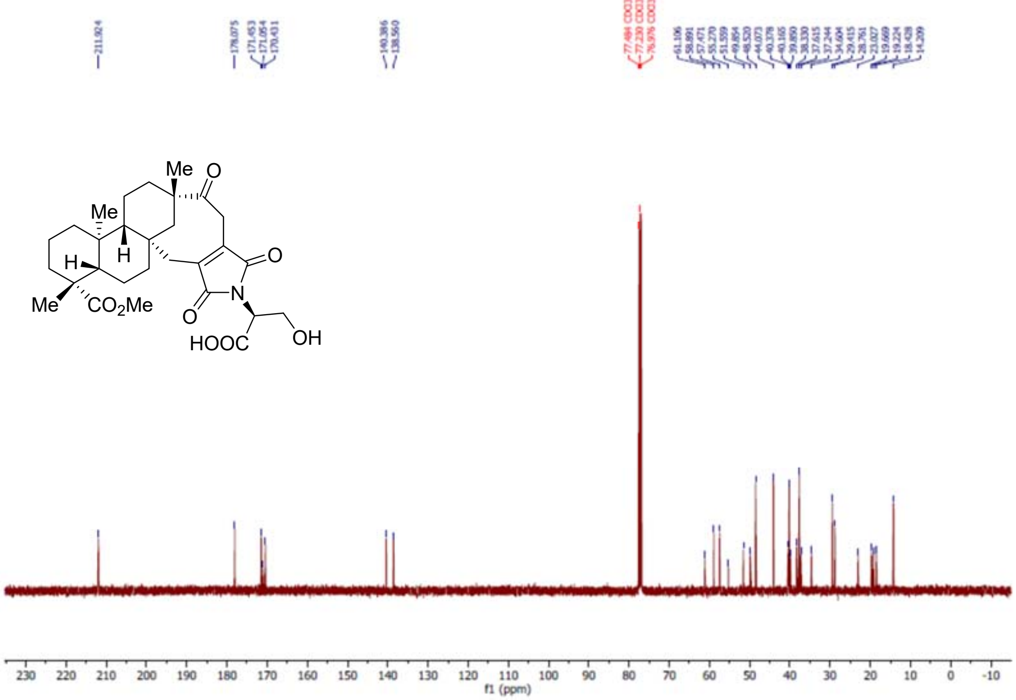

Supplementary Figure 207. ^{1}H and ^{13} C spectra of **47be**.

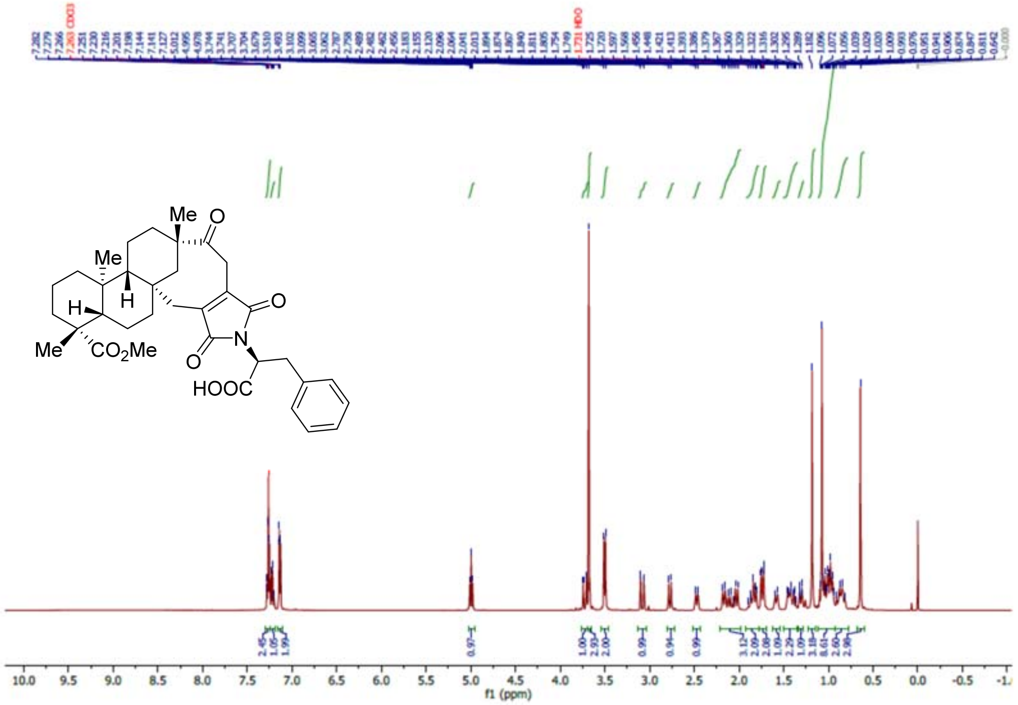

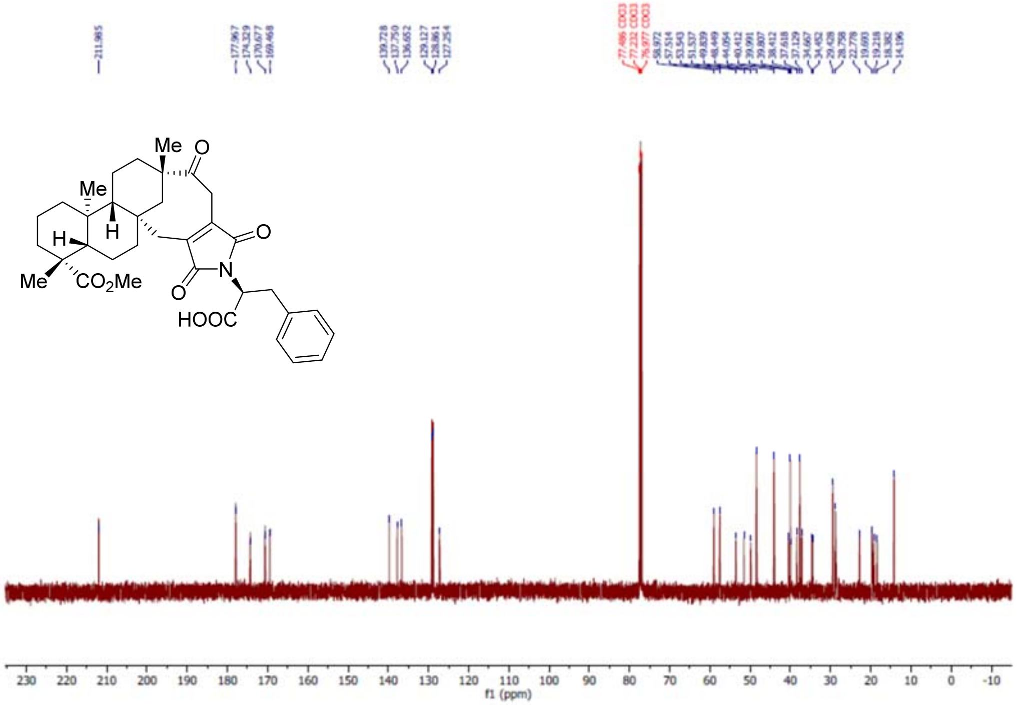

Supplementary Figure 208. ^{1}H and ^{13}C spectra of **47bf**.

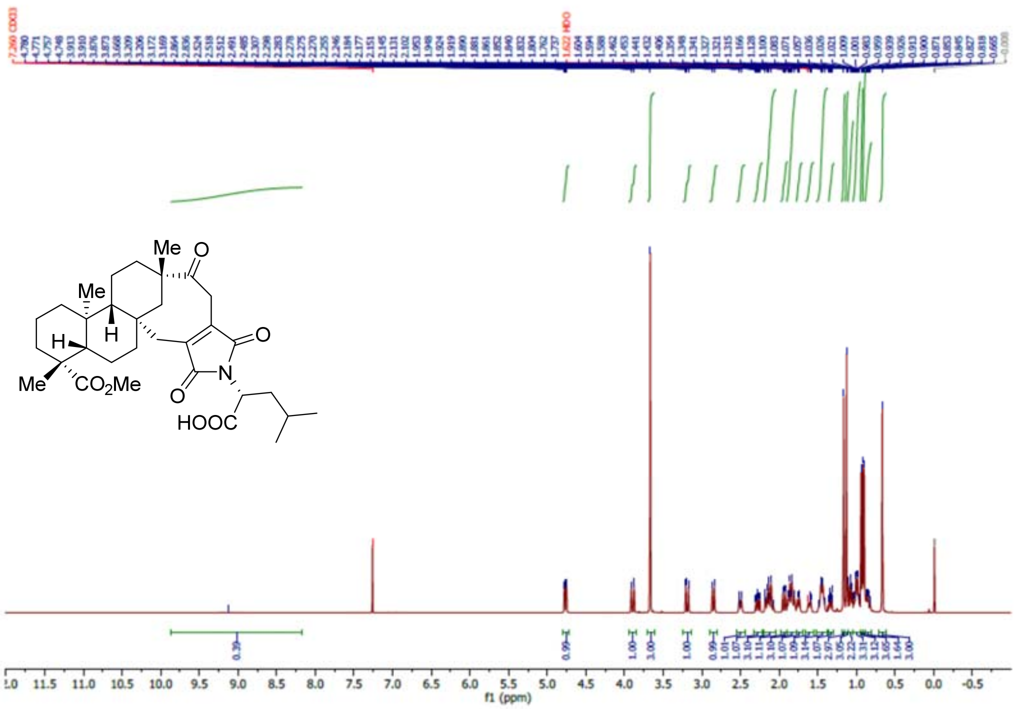

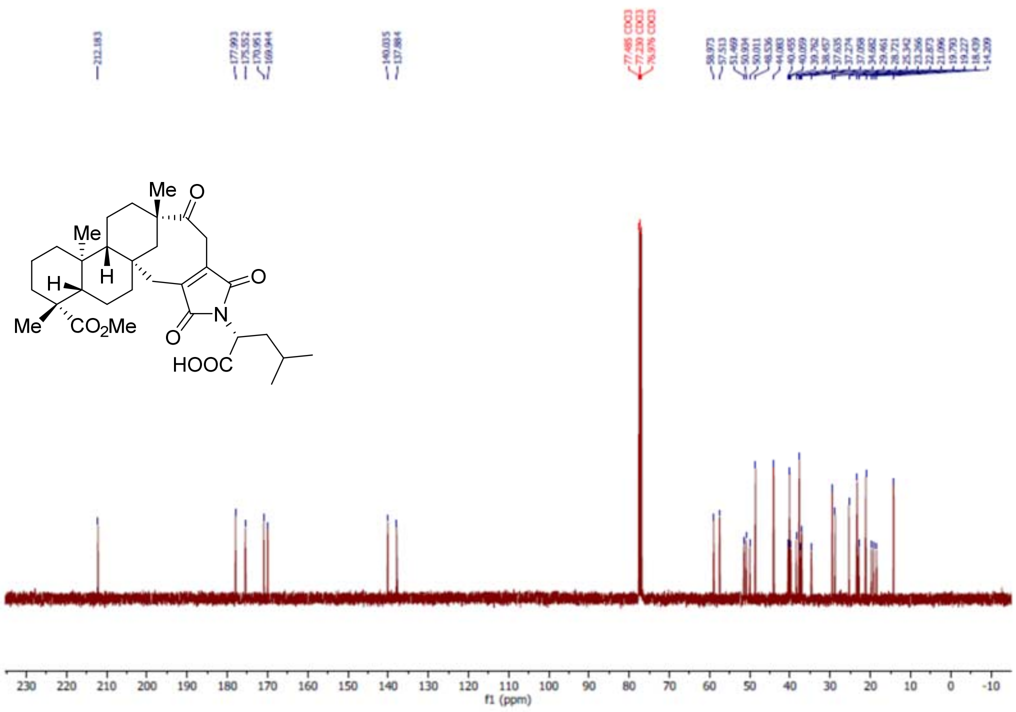

Supplementary Figure 209. ^{1}H and ^{13}C spectra of **47bg**.

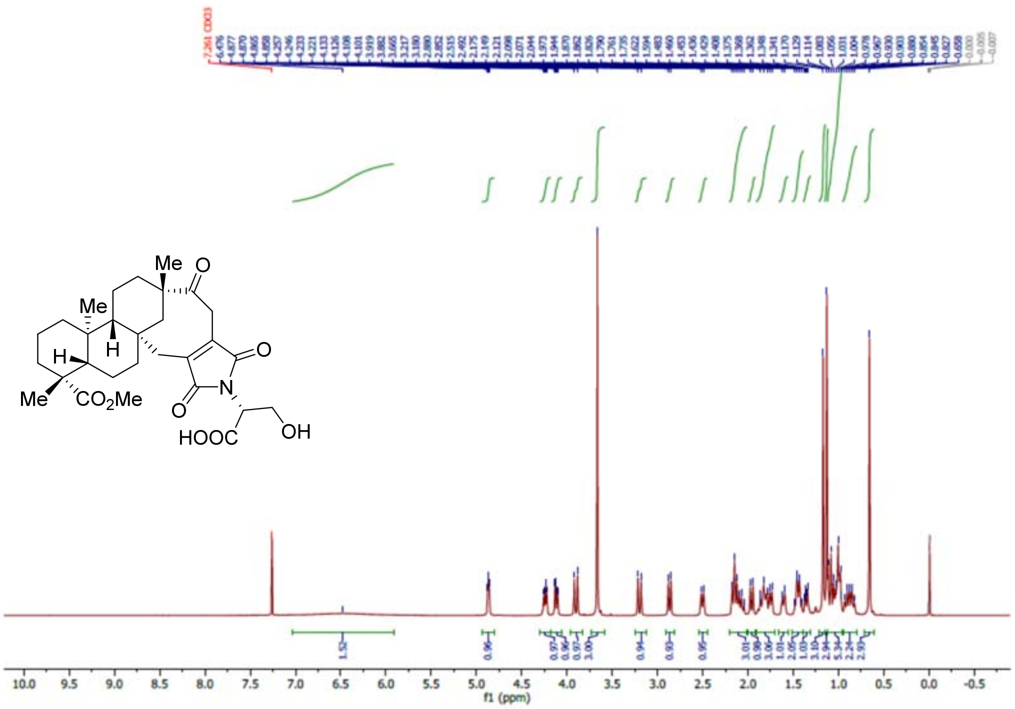

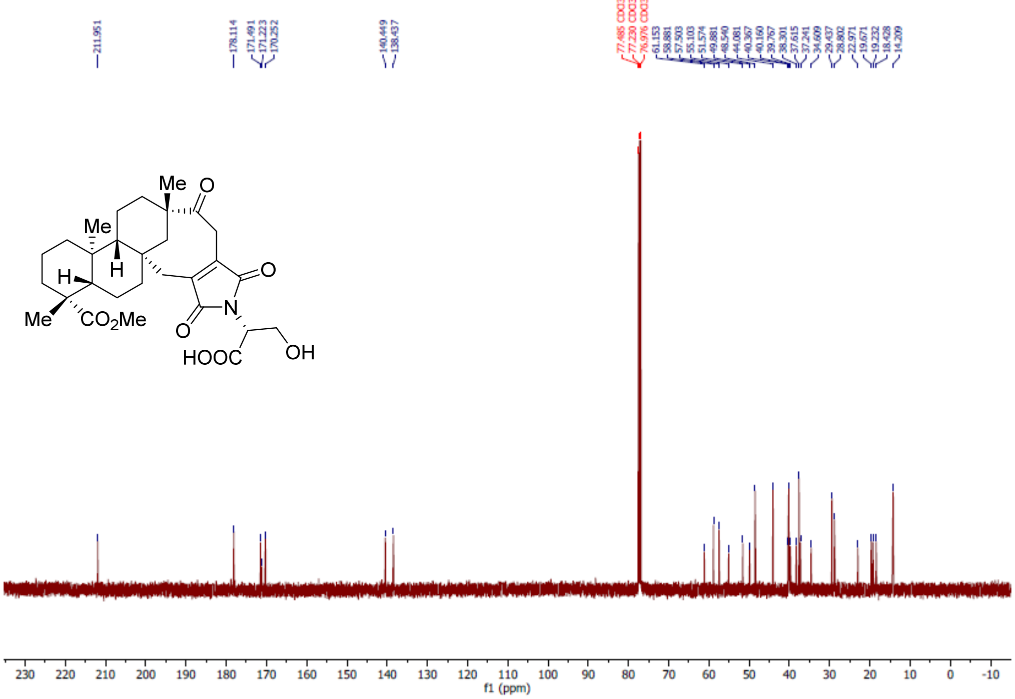

Supplementary Figure 210. ^{1}H and ^{13}C spectra of **47bh**.

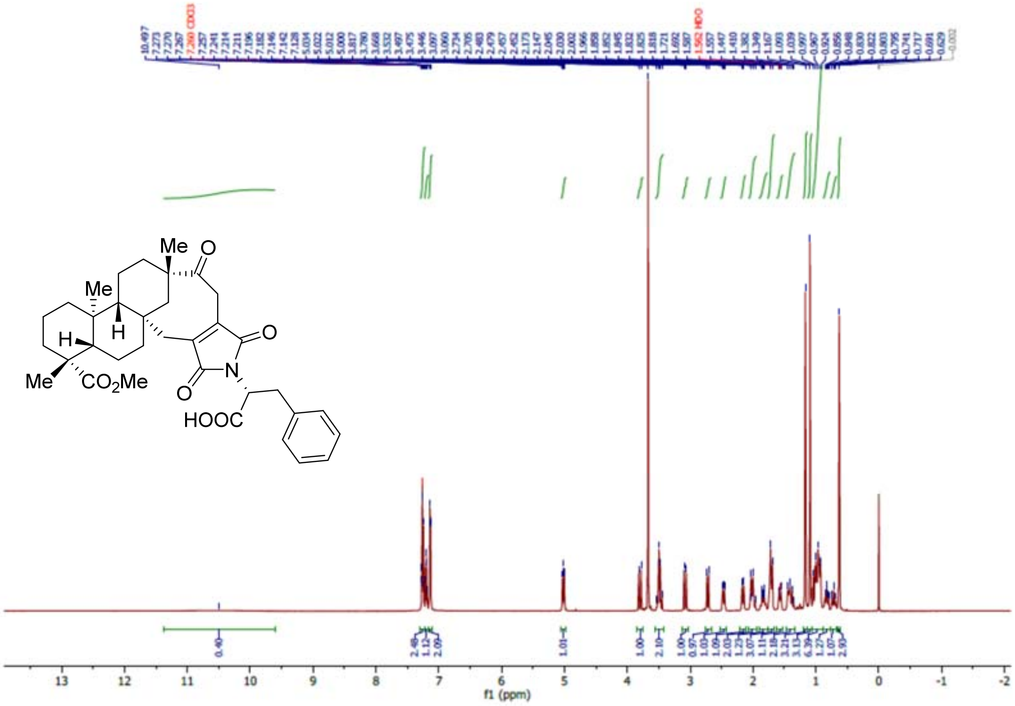

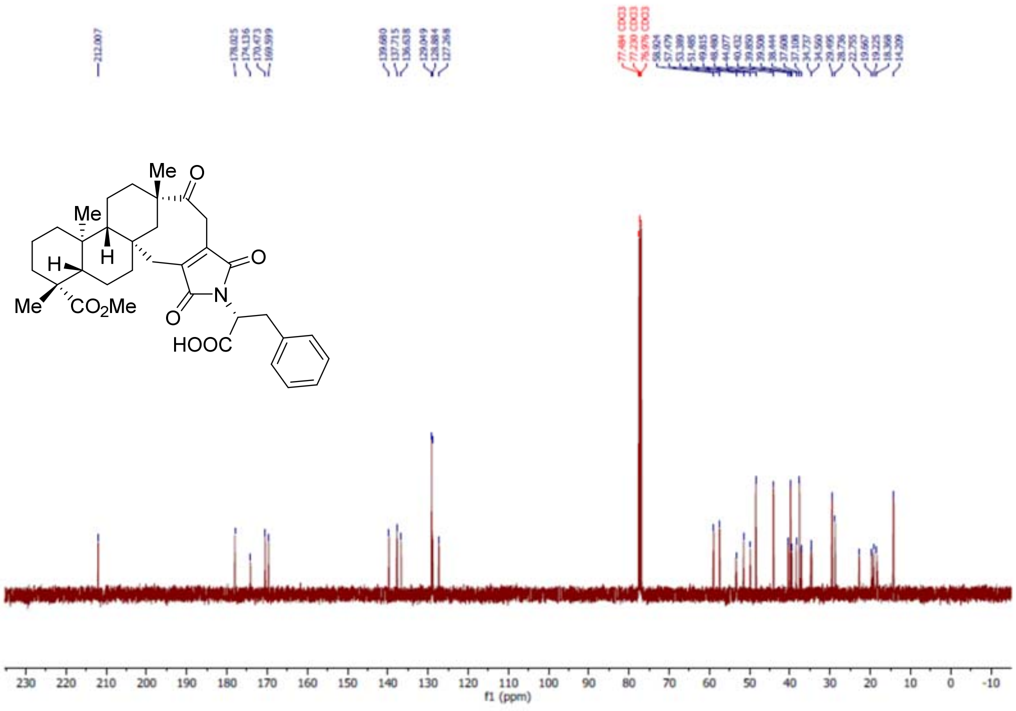

Supplementary Figure 211. ^{1}H and ^{13}C spectra of **47bi**.

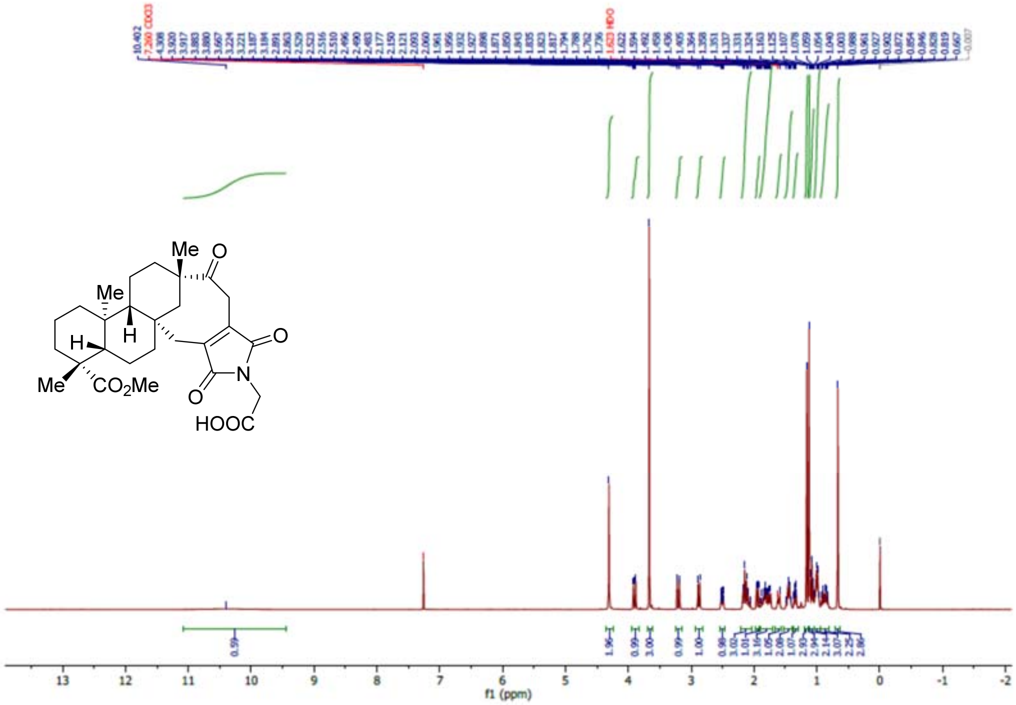

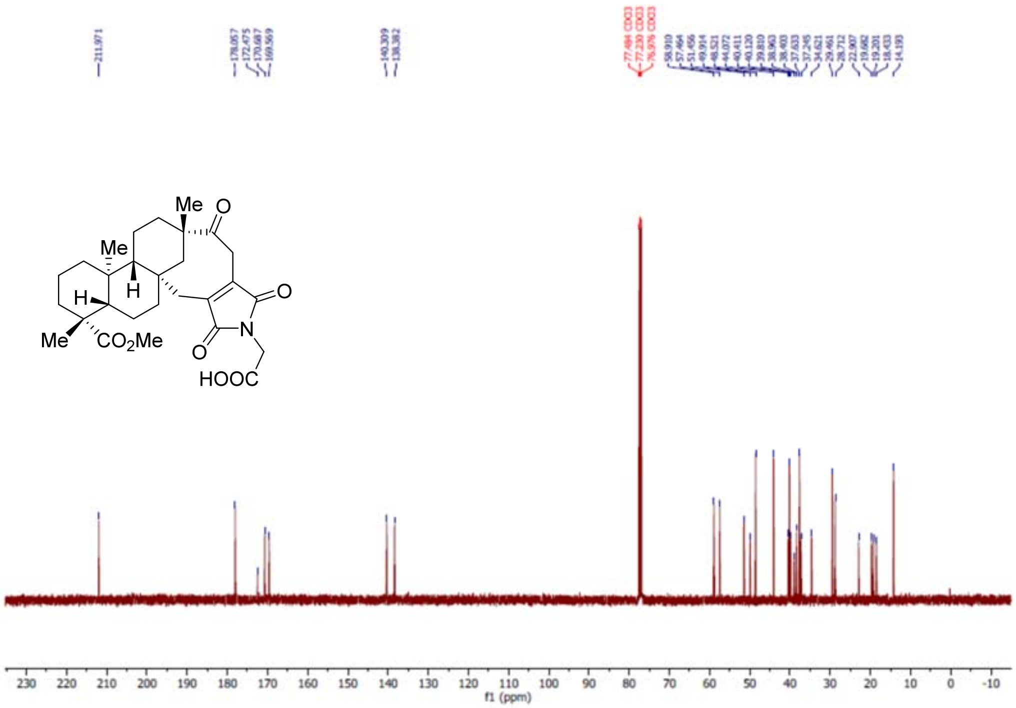

Supplementary Figure 212. ^{1}H and ^{13}C spectra of **47bj**.

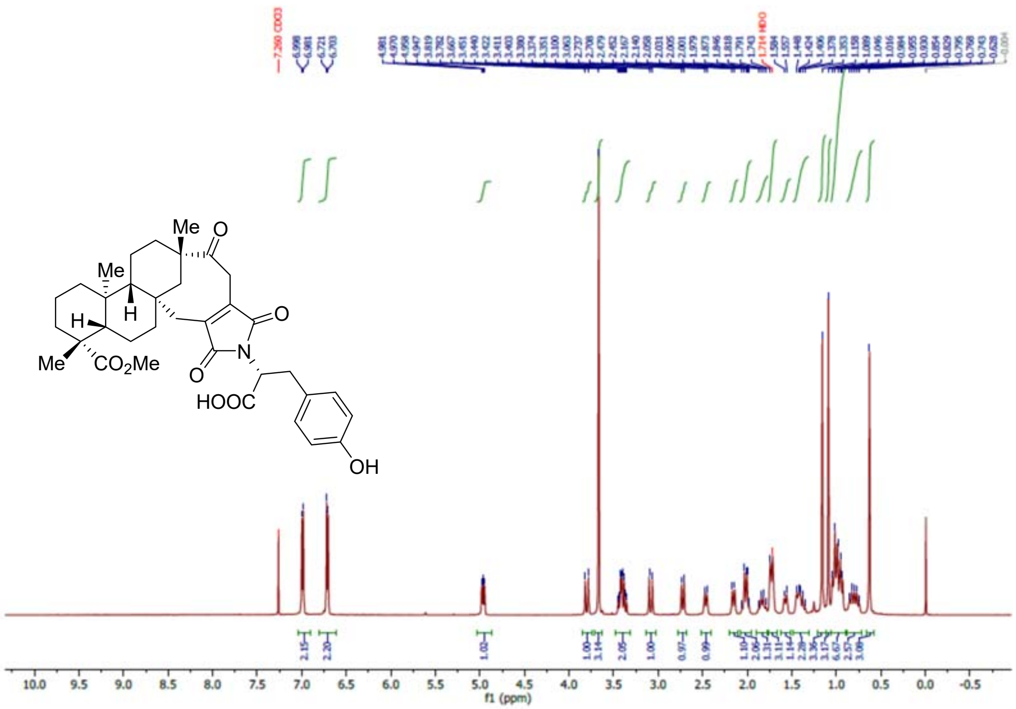

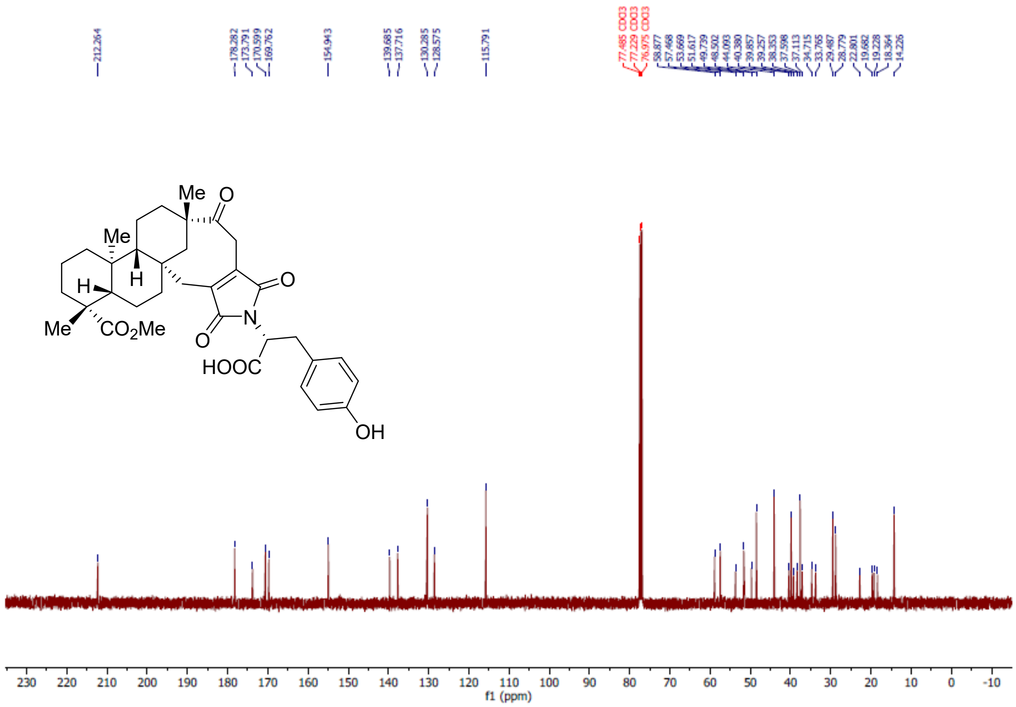

Supplementary Figure 213. ^{1}H and ^{13} C spectra of **47bk**.

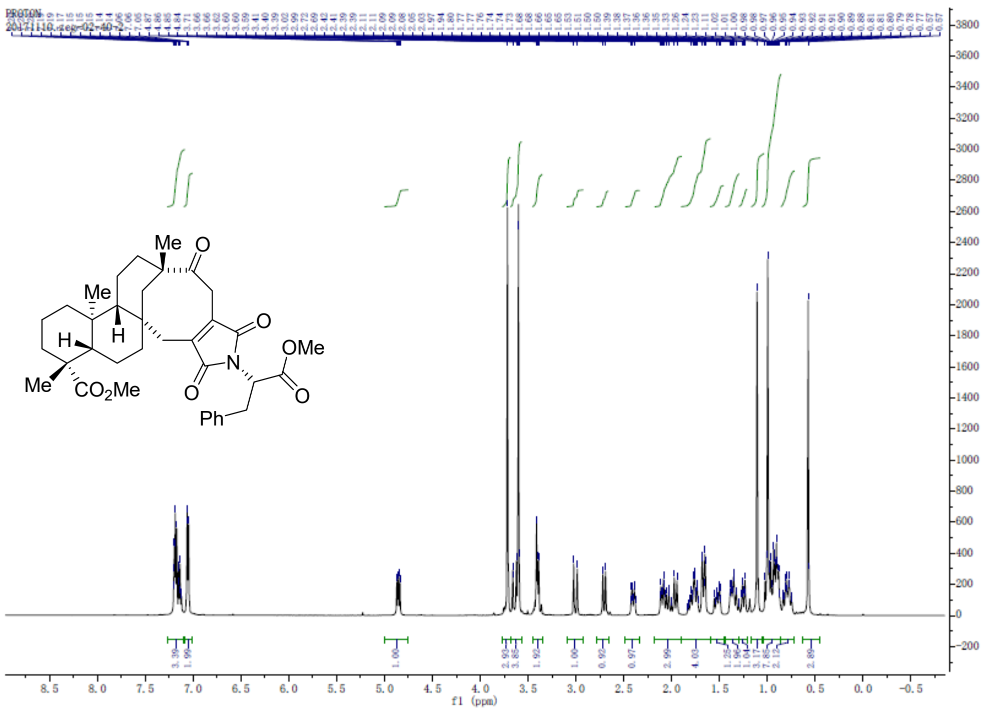

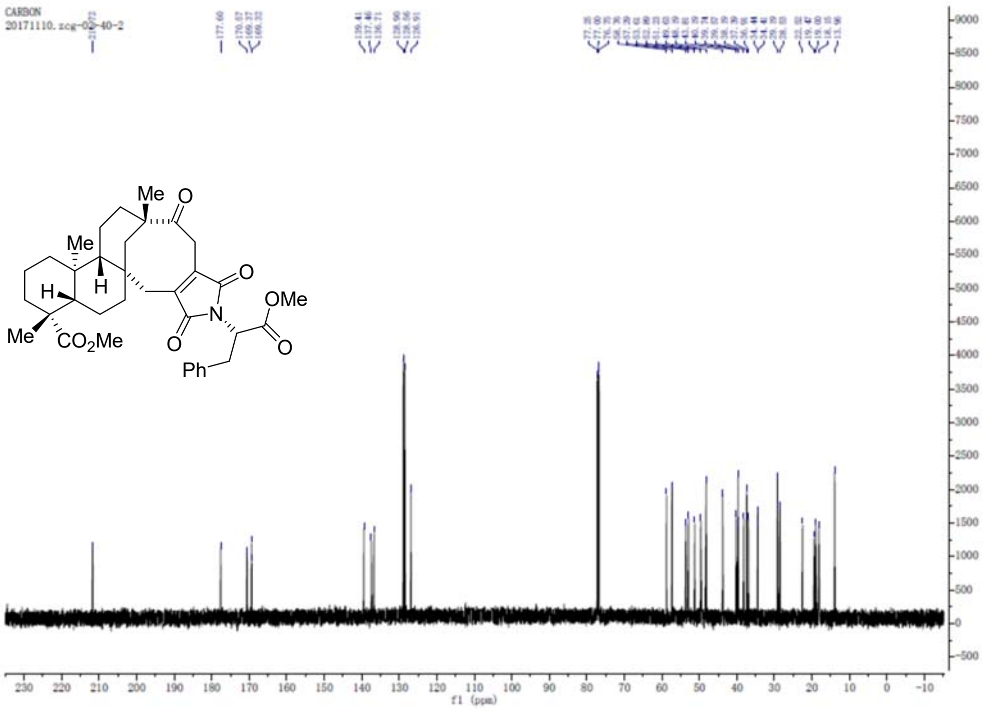

Supplementary Figure 214. ^{1}H and ^{13}C spectra of **47bl**.

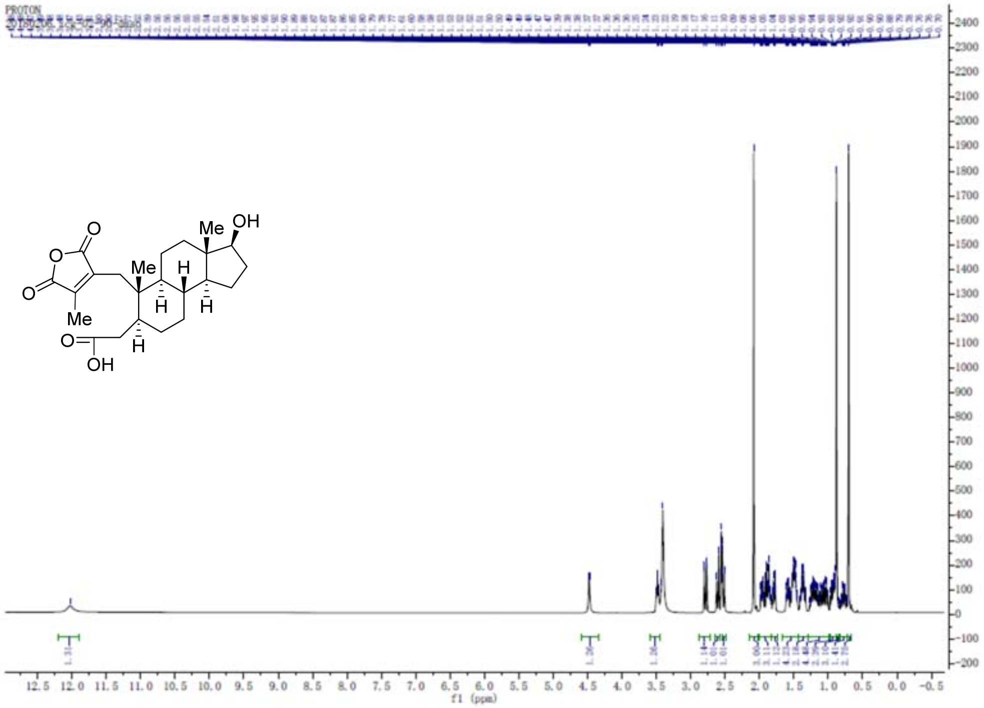

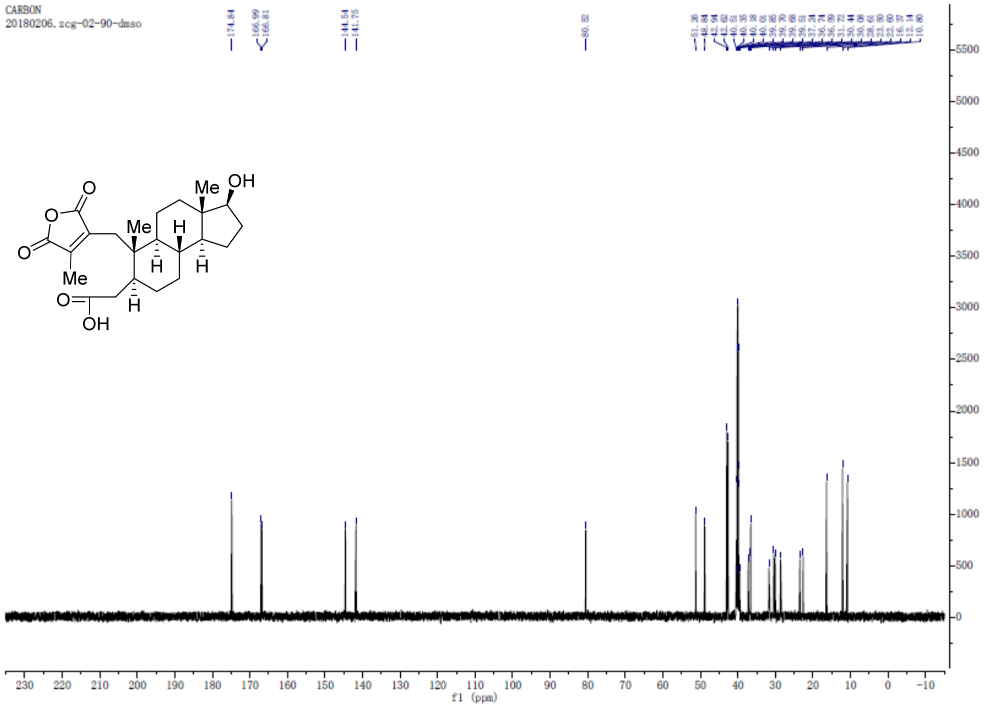

Supplementary Figure 215. ^{1}H and ^{13}C spectra of **49a**.

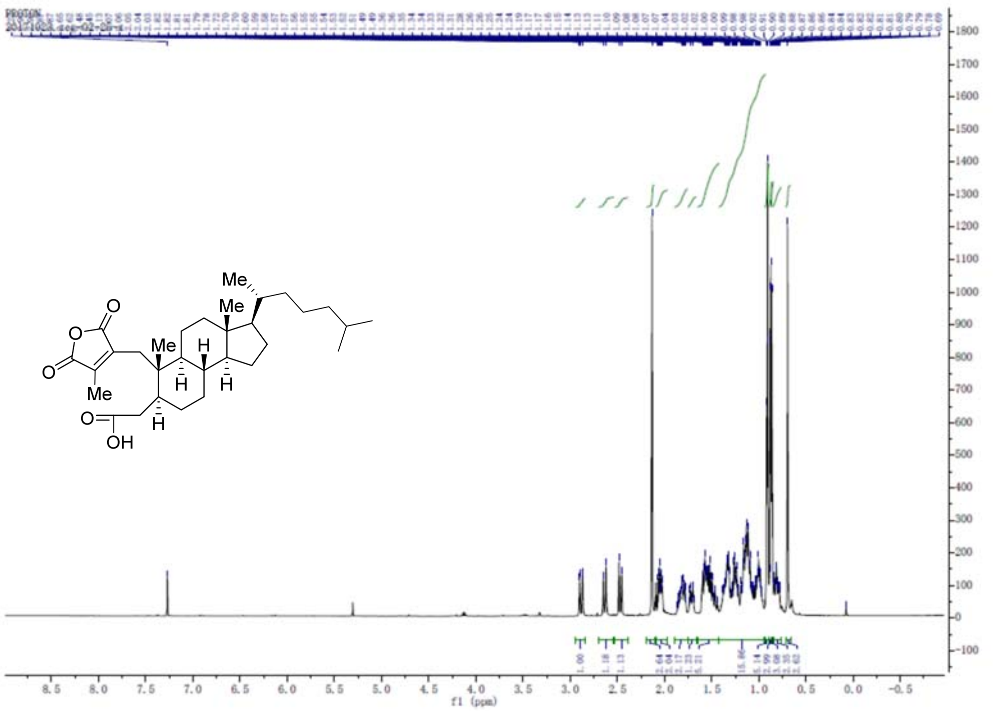

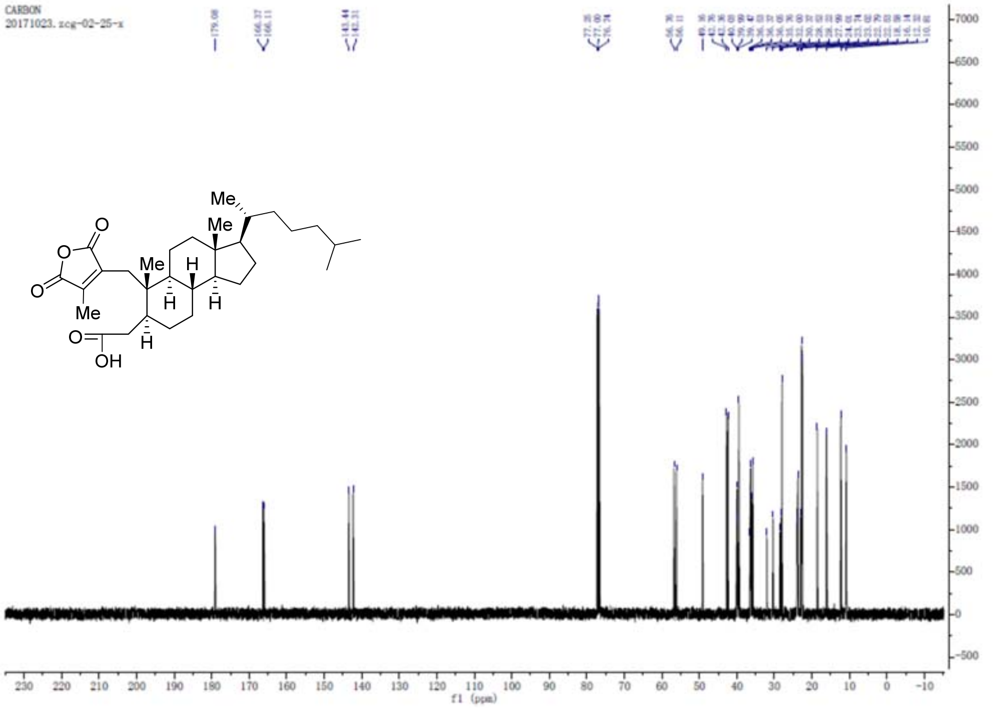

Supplementary Figure 216. ^{1}H and ^{13}C spectra of **49b**.

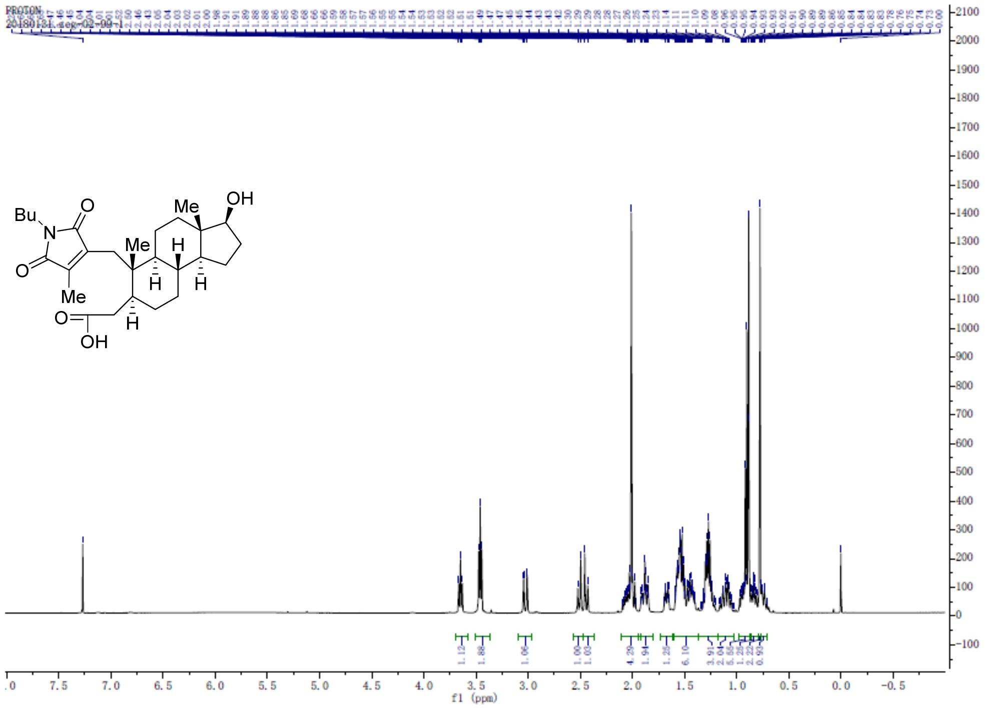

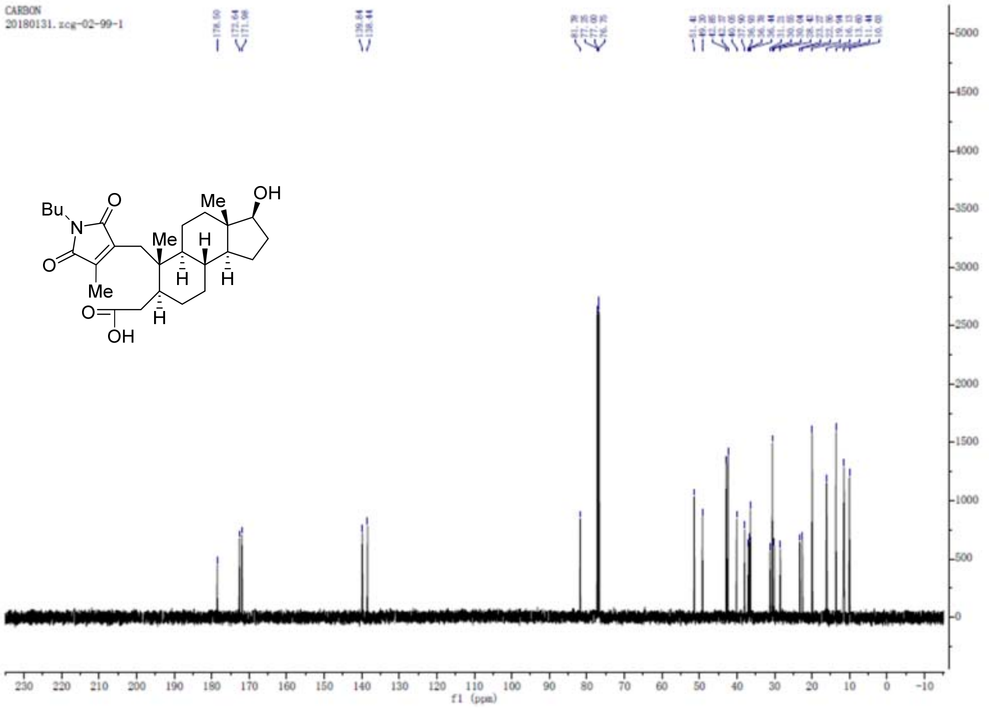

Supplementary Figure 217. ^{1}H and ^{13} C spectra of **50a**.

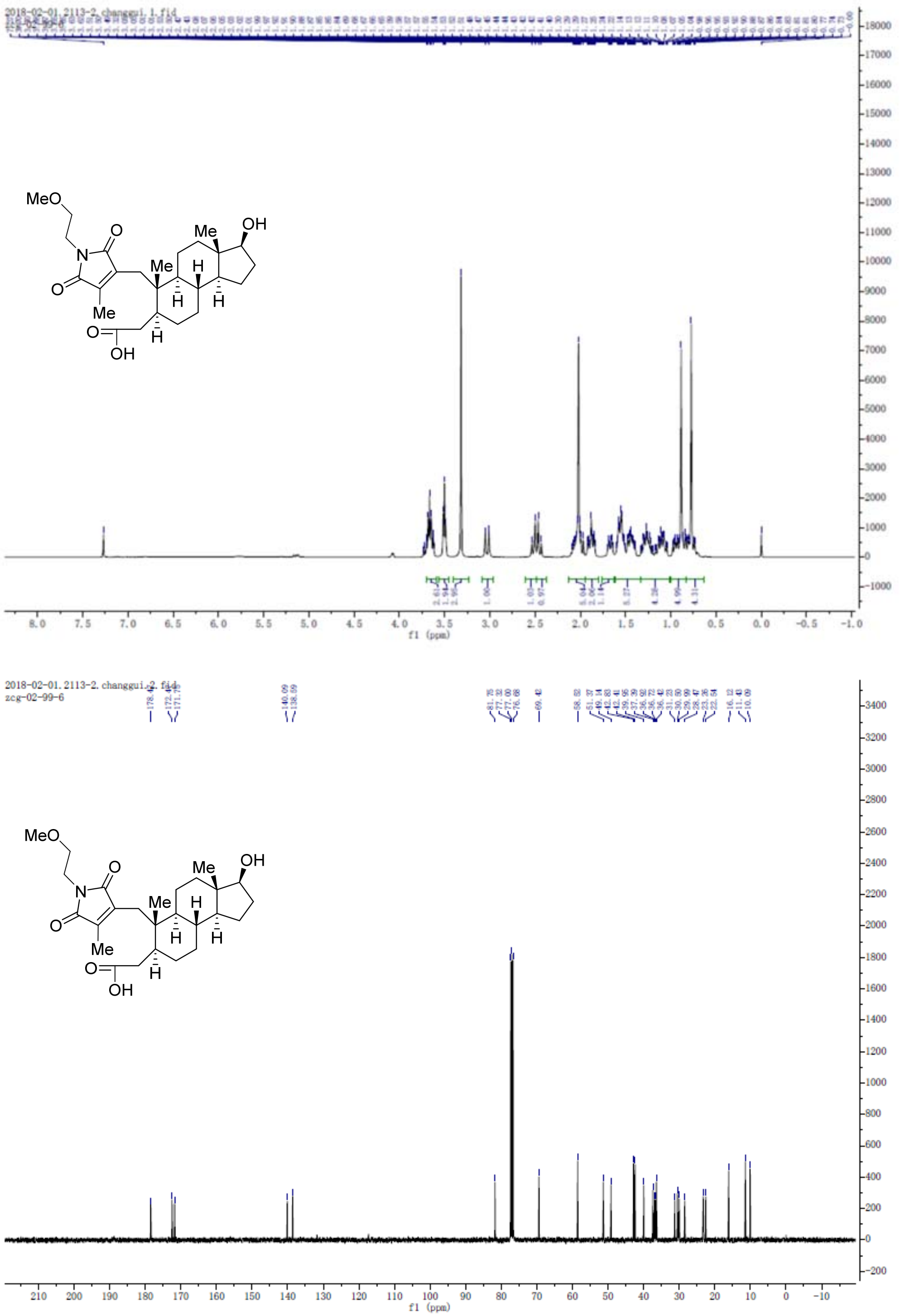

Supplementary Figure 218. ^{1}H and ^{13}C spectra of **50b**.

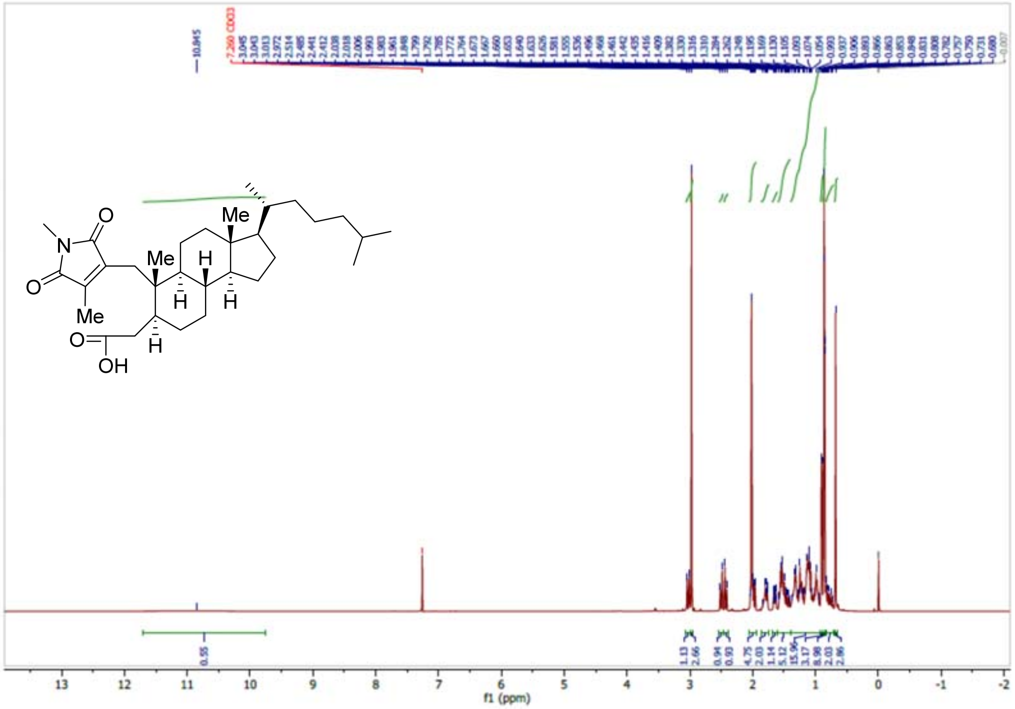

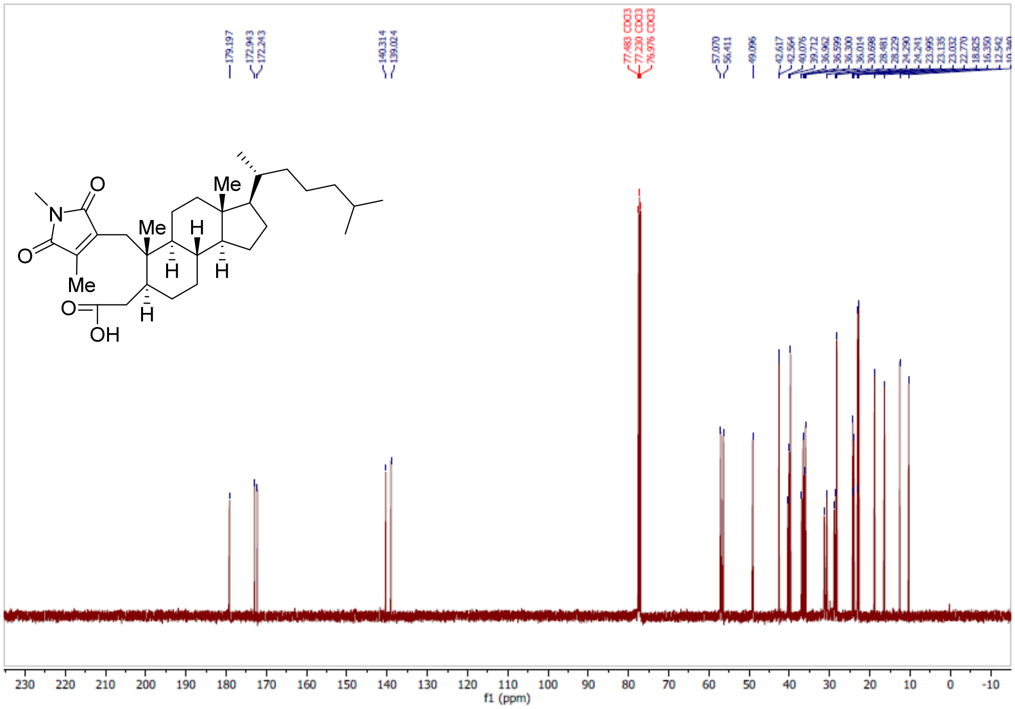

Supplementary Figure 219. ^{1}H and ^{13} C spectra of **50c**.

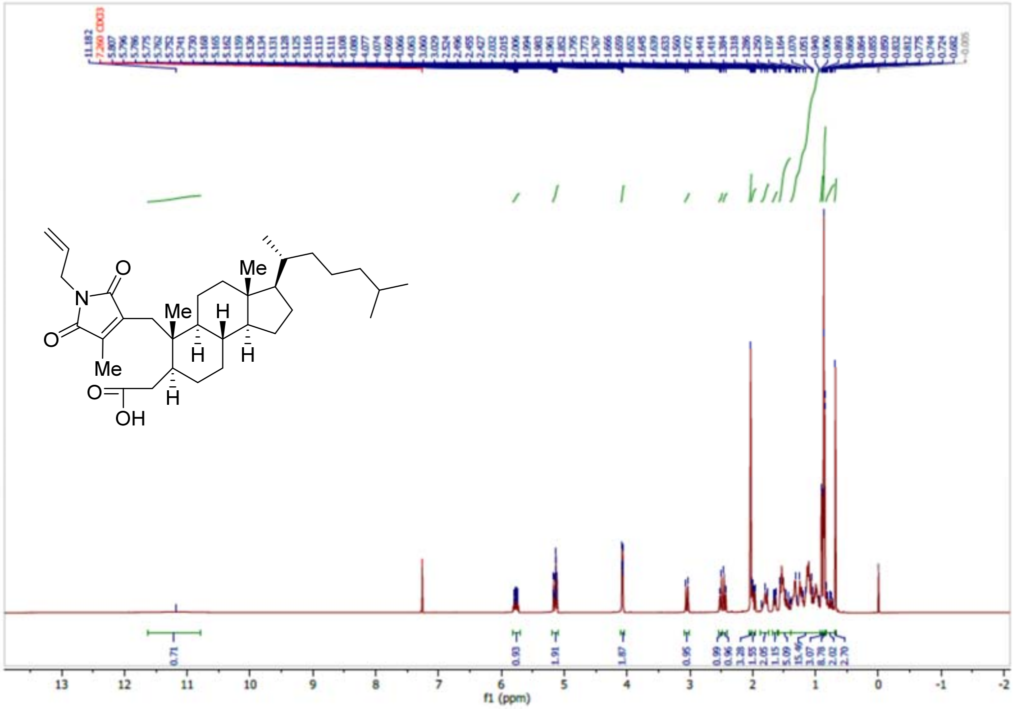

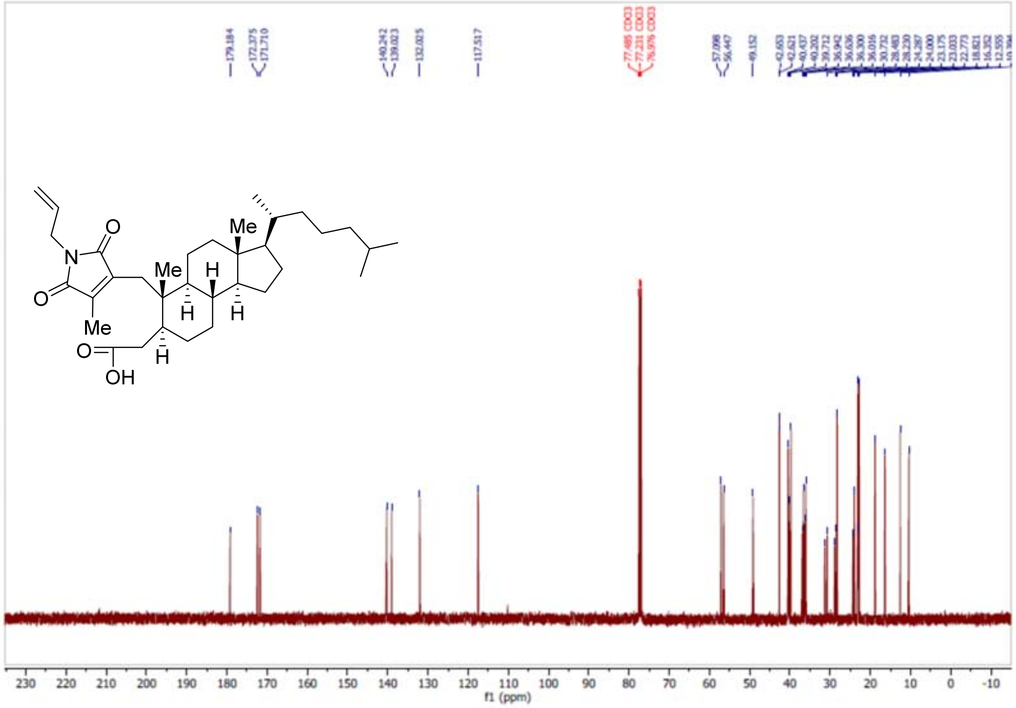

Supplementary Figure 220. ^{1}H and ^{13}C spectra of **50d**.

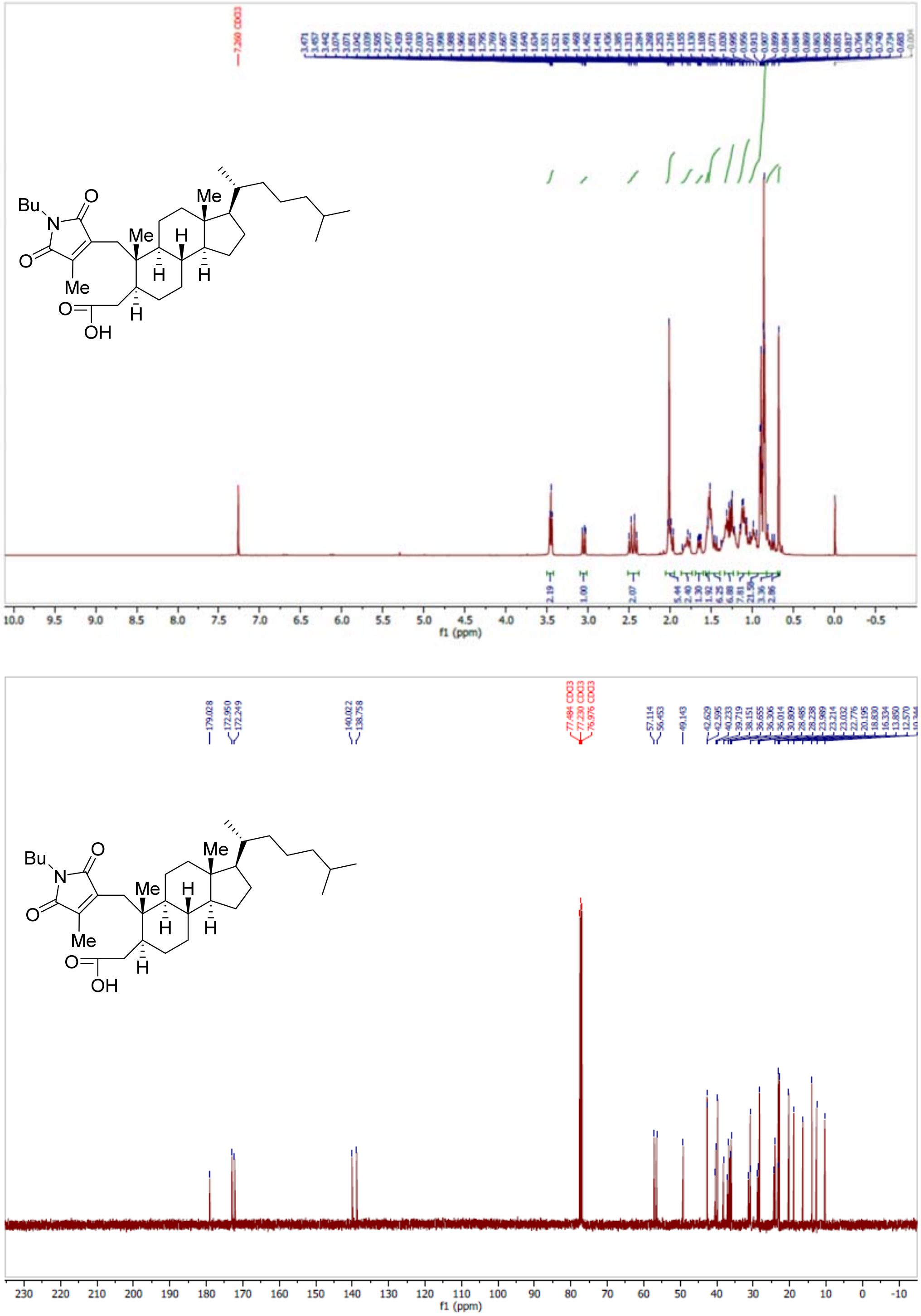

Supplementary Figure 221. ^{1}H and ^{13}C spectra of **50e**.

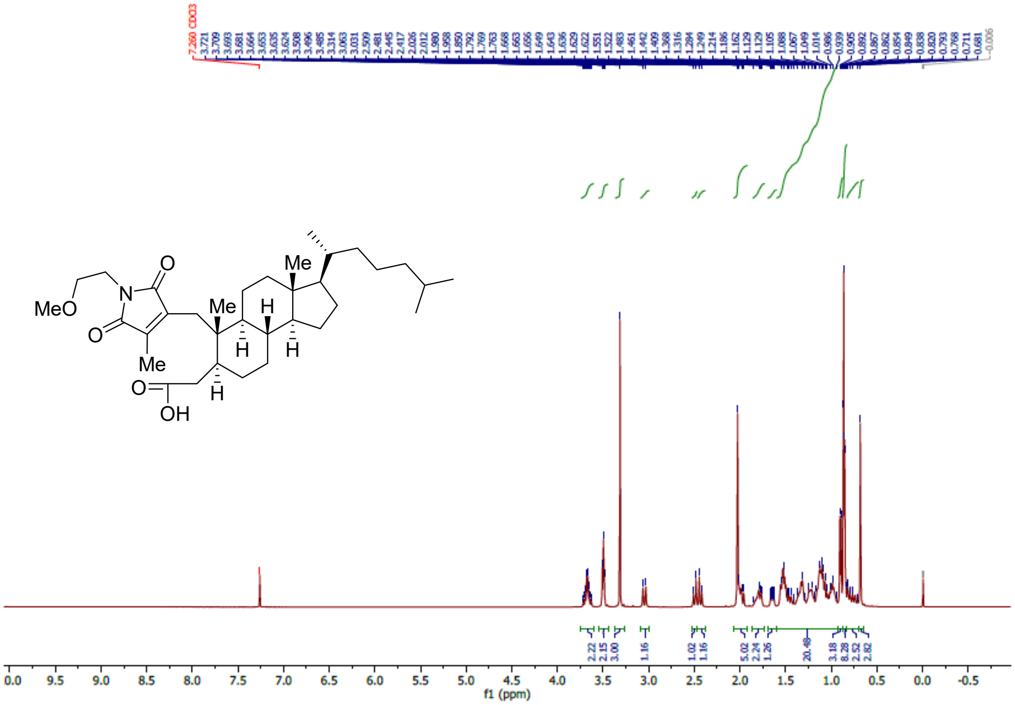

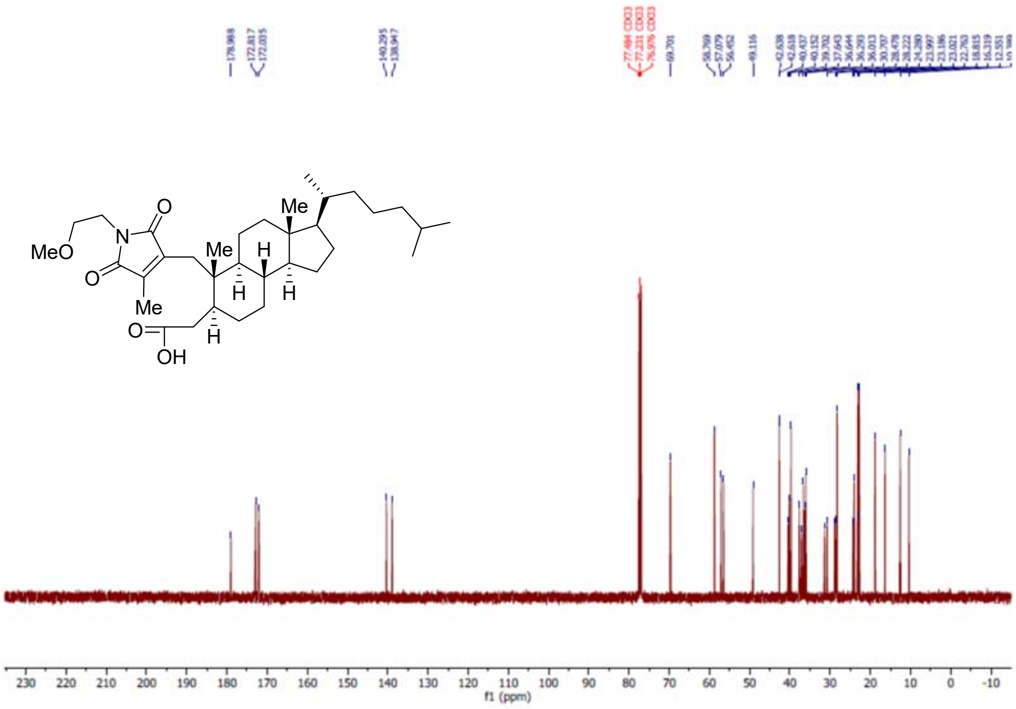

Supplementary Figure 222. ^{1}H and ^{13}C spectra of **50f**.

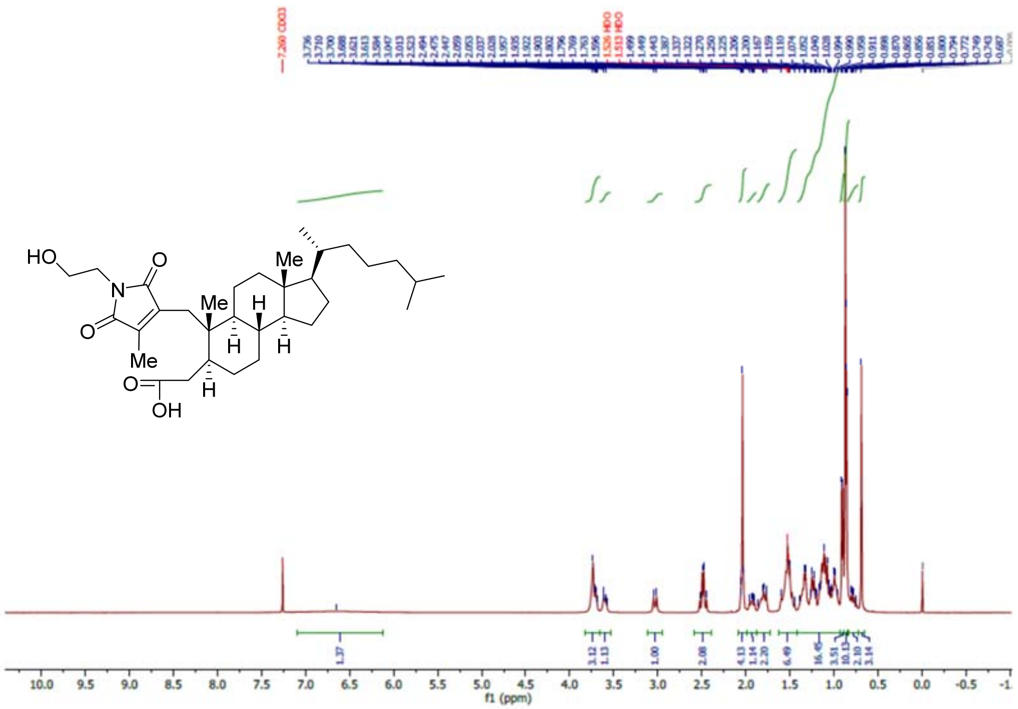

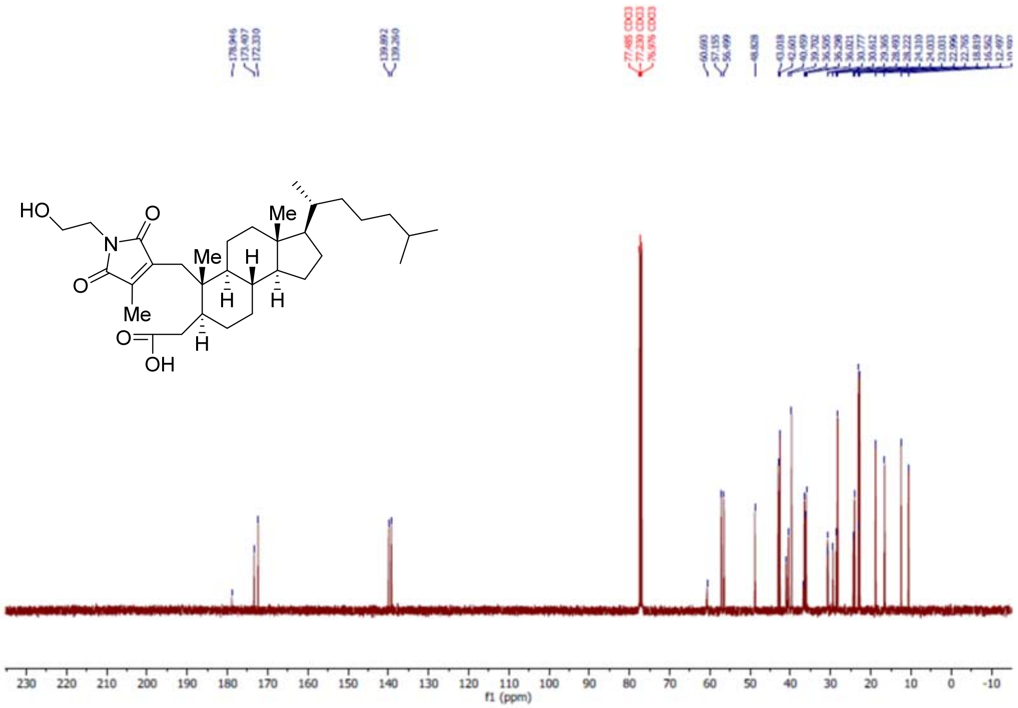

Supplementary Figure 223. ^{1}H and ^{13}C spectra of **50g**.

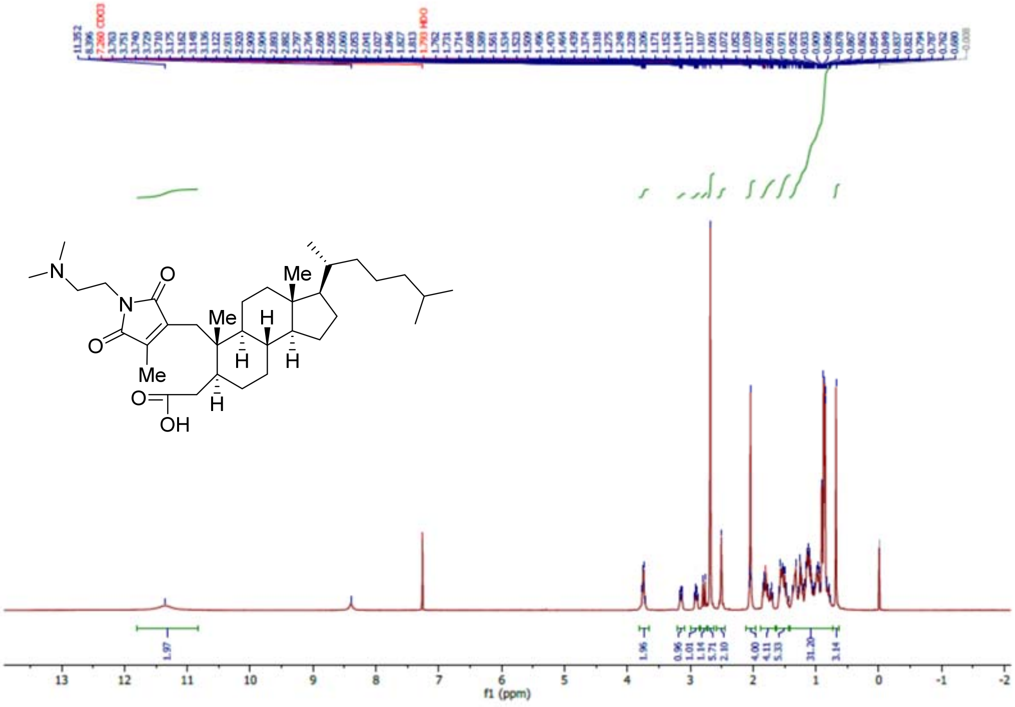

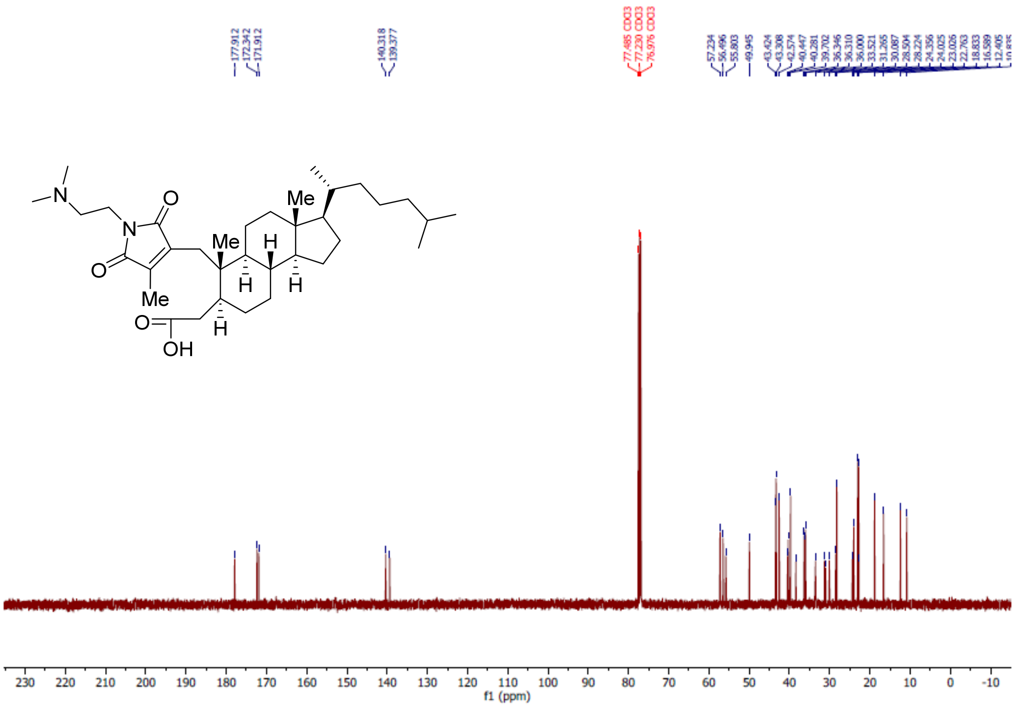

Supplementary Figure 224. ^{1}H and ^{13}C spectra of **50h**.

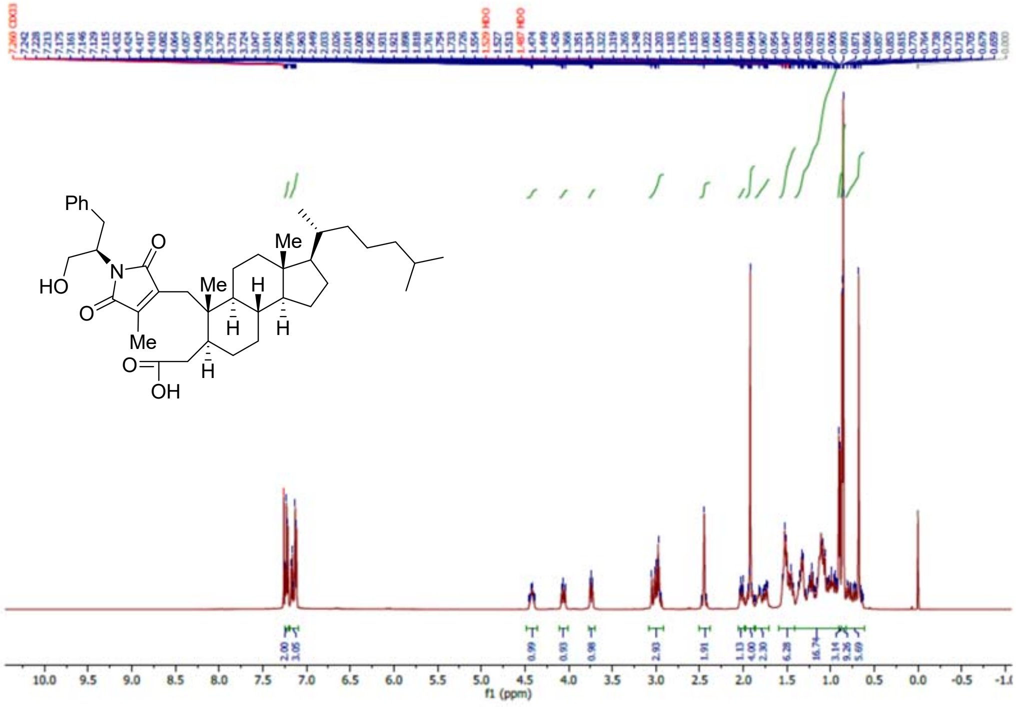

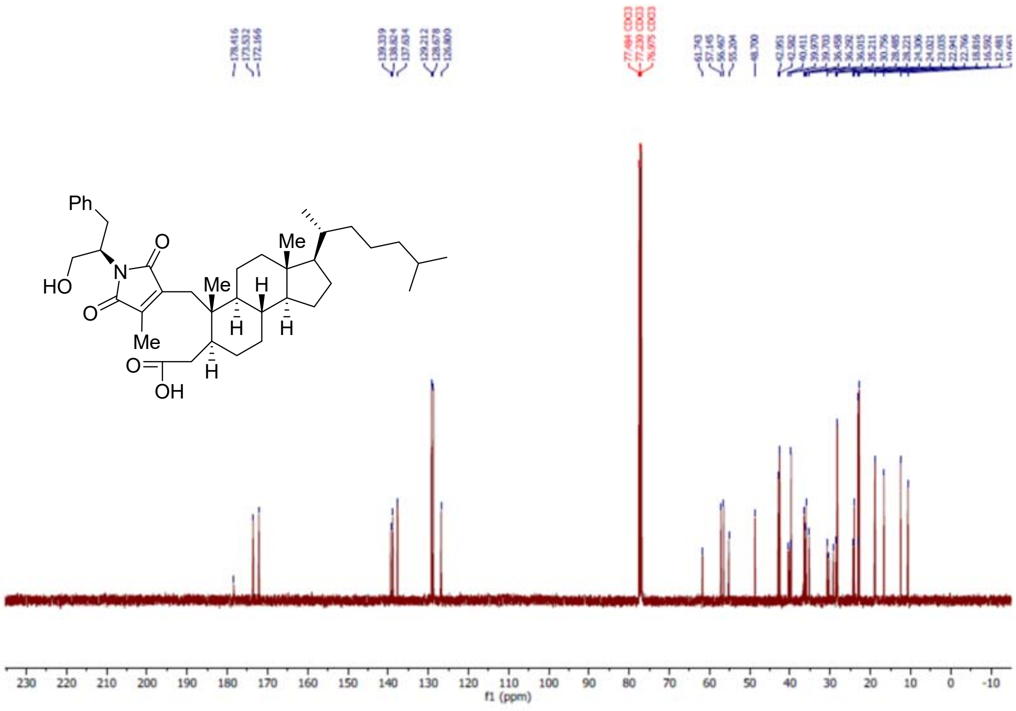

Supplementary Figure 225. ^{1}H and ^{13}C spectra of **50i**.

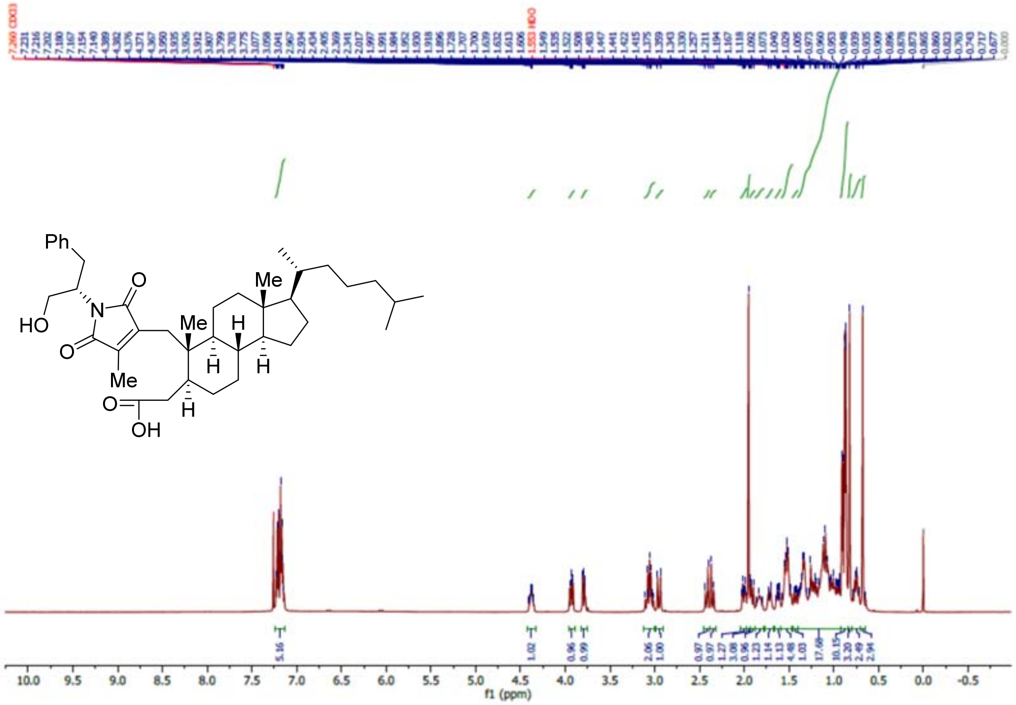

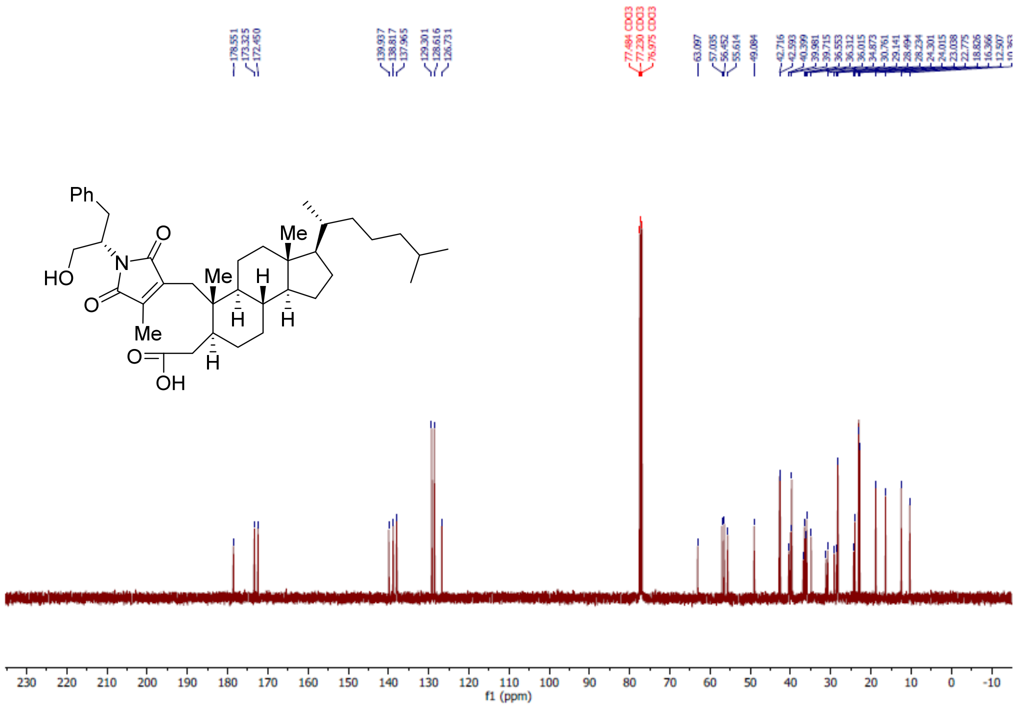

Supplementary Figure 226. ^{1}H and ^{13}C spectra of **50j**.

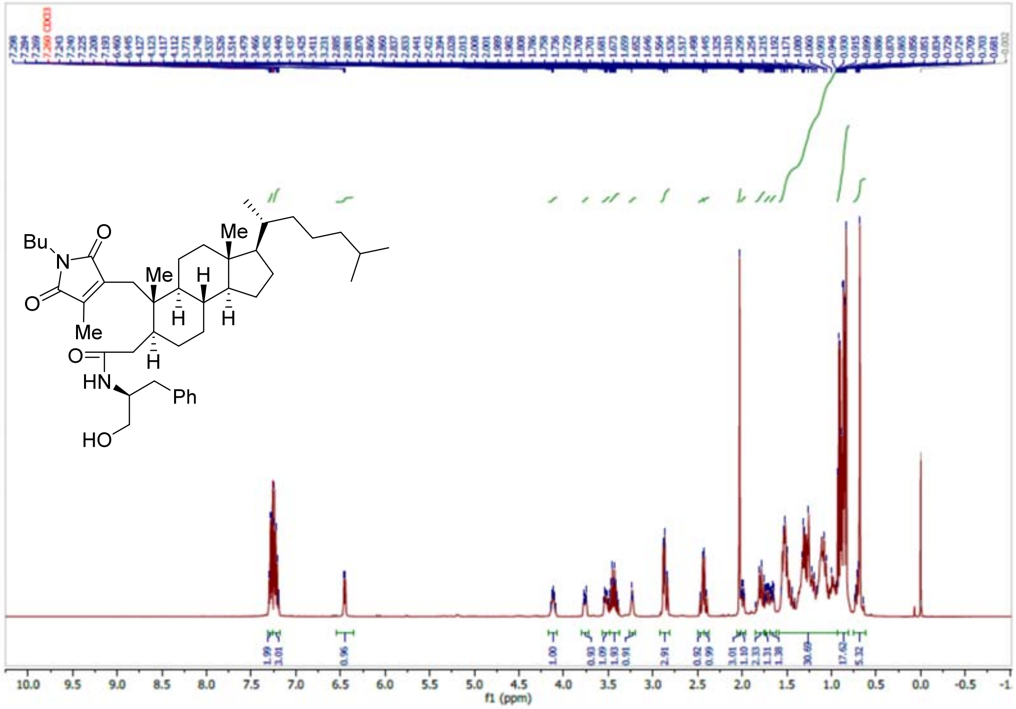

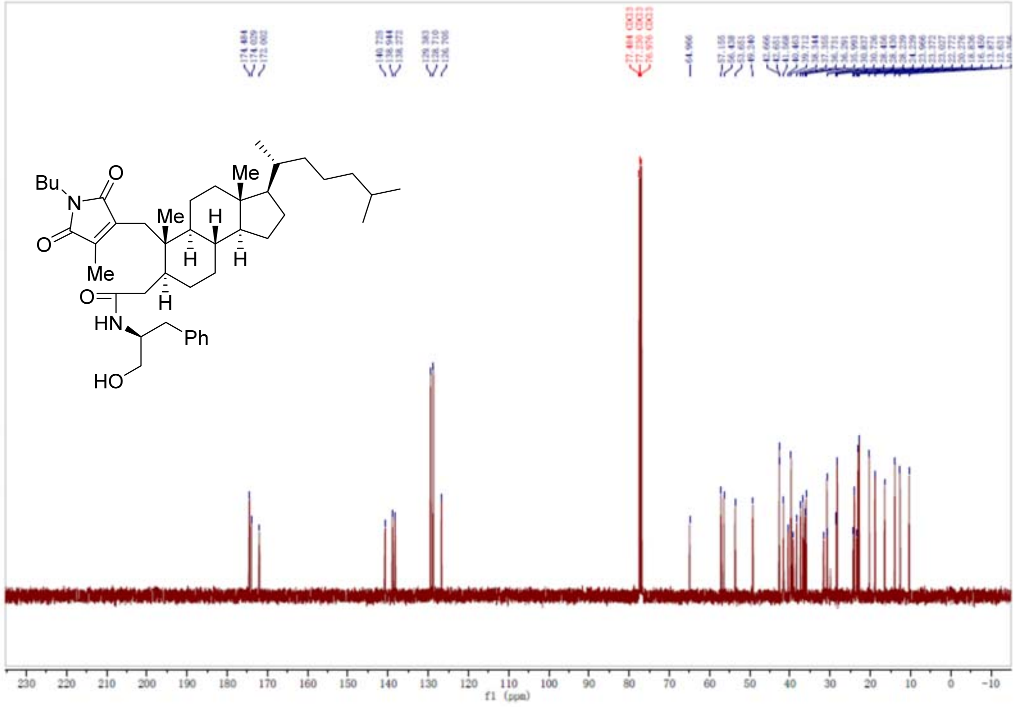

Supplementary Figure 227. ^{1}H and ^{13}C spectra of **51a**.

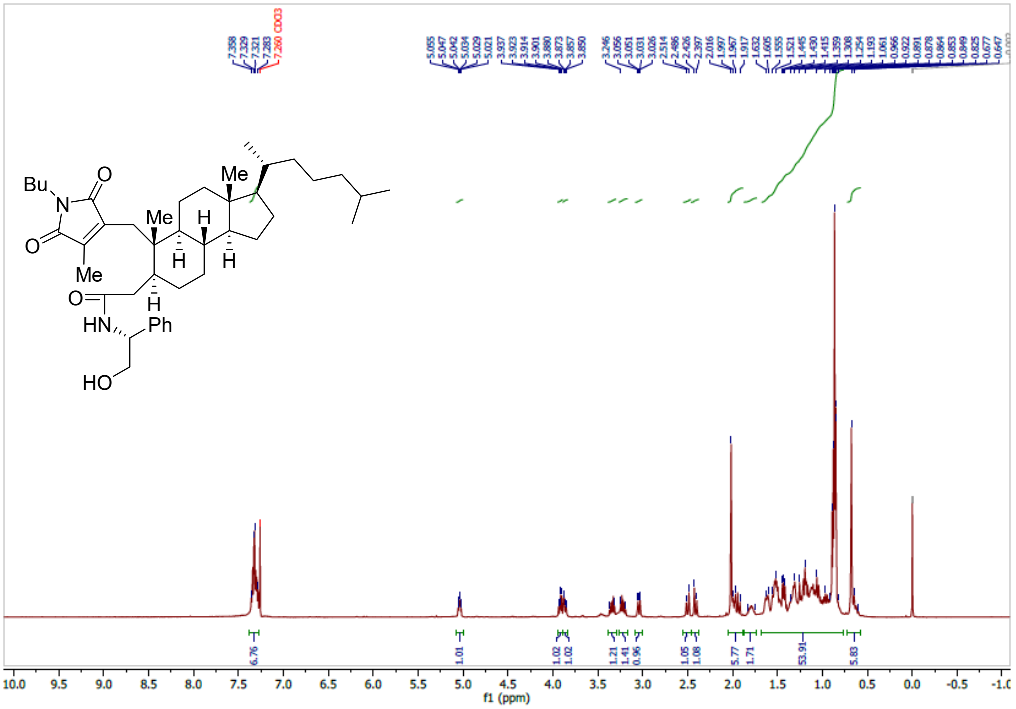

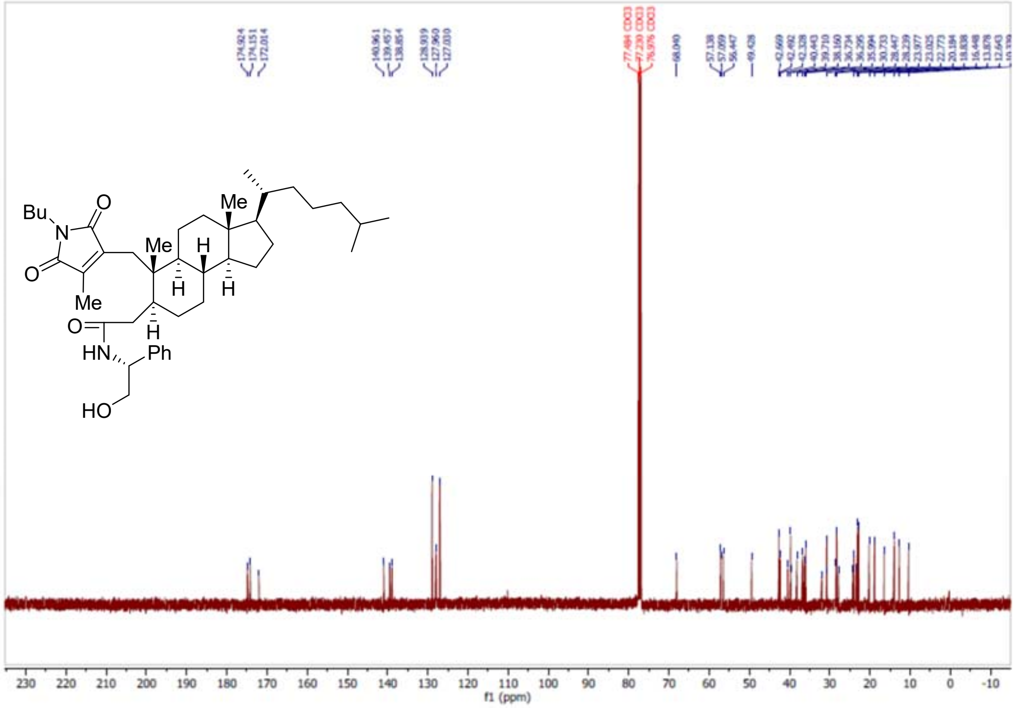

Supplementary Figure 228. ^{1}H and ^{13}C spectra of **51b**.

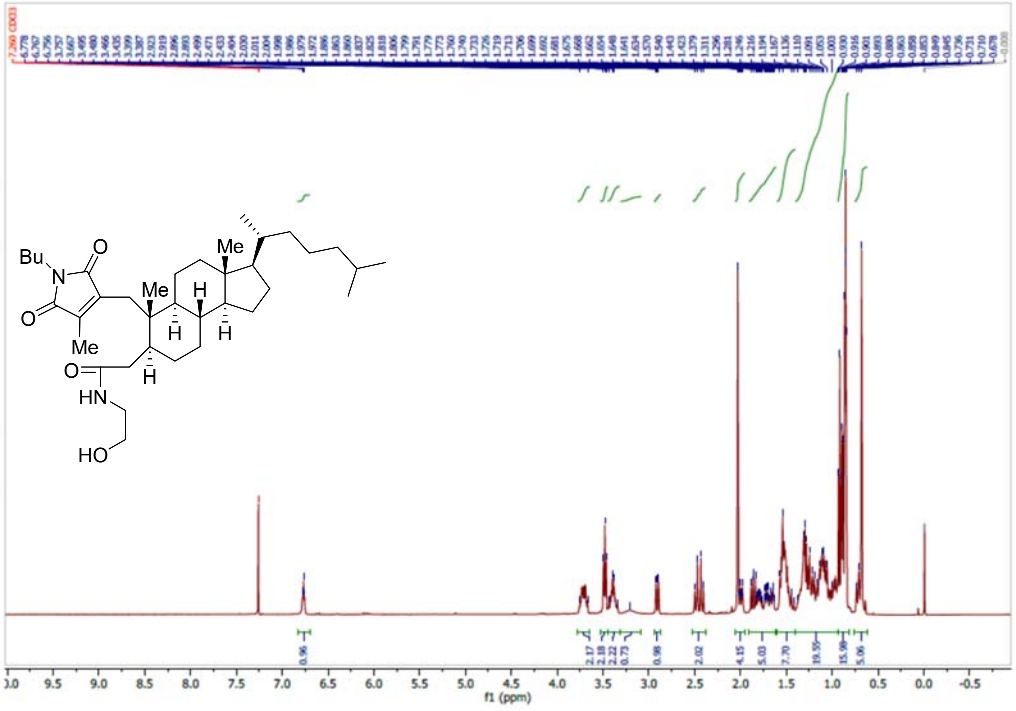

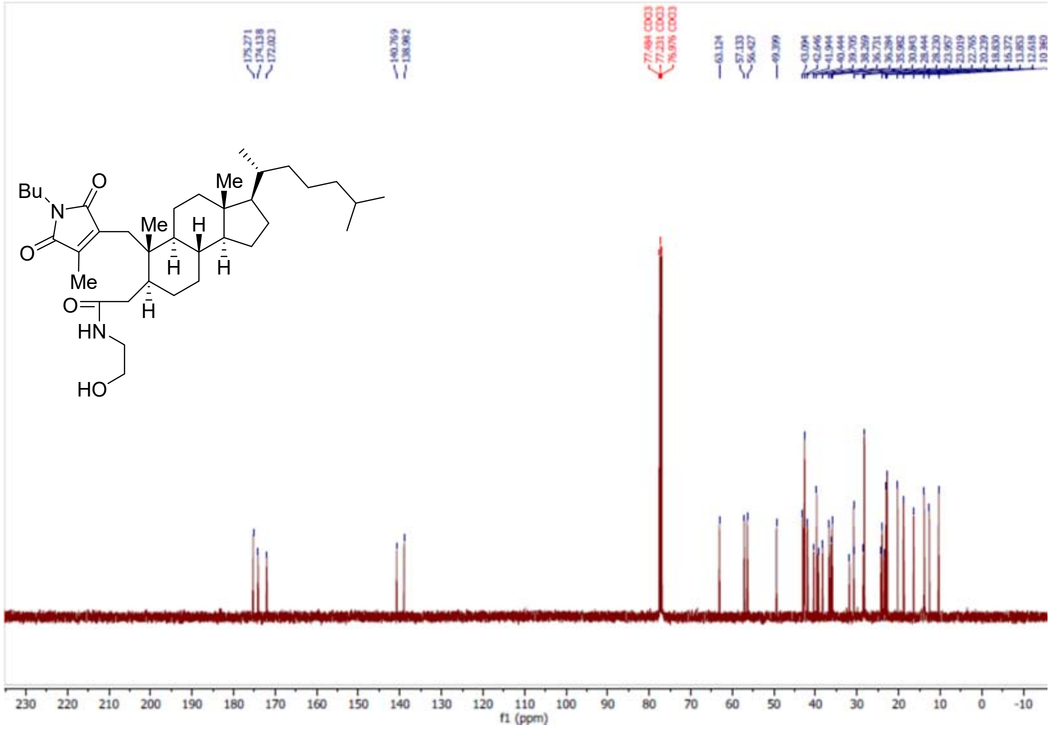

Supplementary Figure 229. ^{1}H and ^{13}C spectra of **51c**.

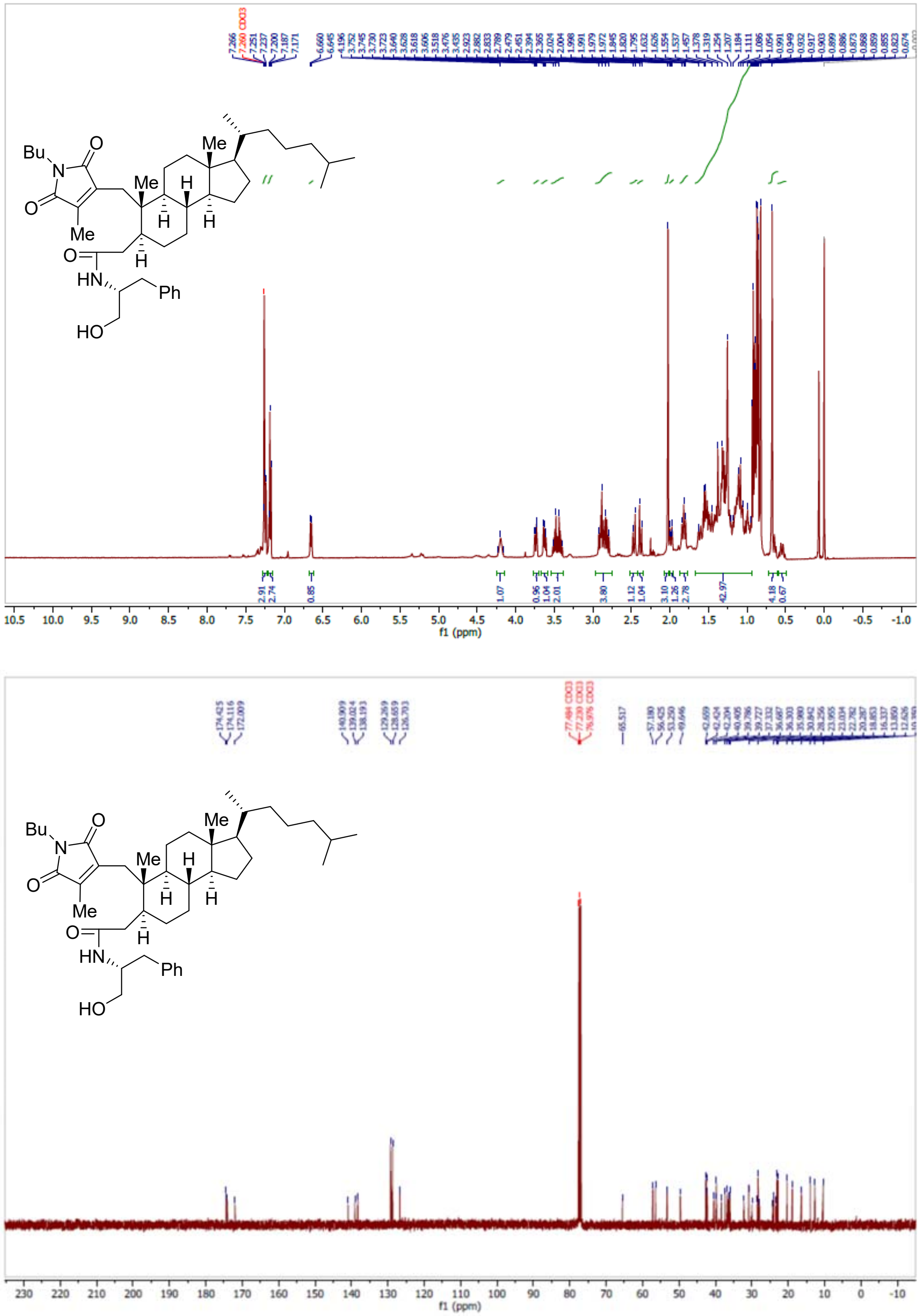

Supplementary Figure 230. ^{1}H and ^{13}C spectra of **51d**.

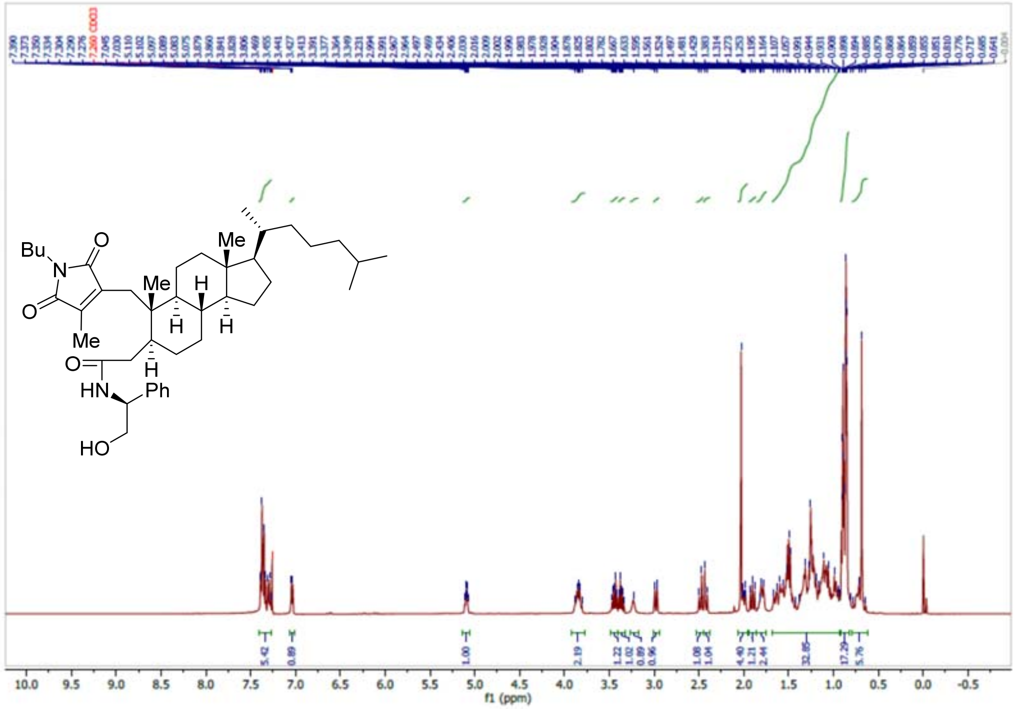

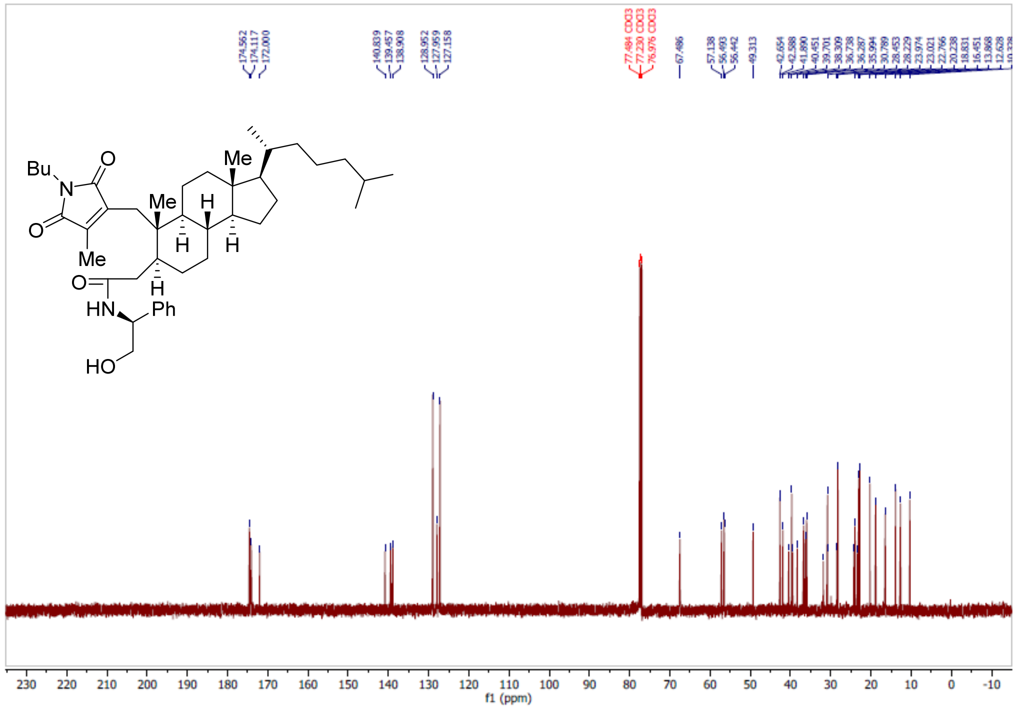

Supplementary Figure 231. ^{1}H and ^{13}C spectra of **51e**.

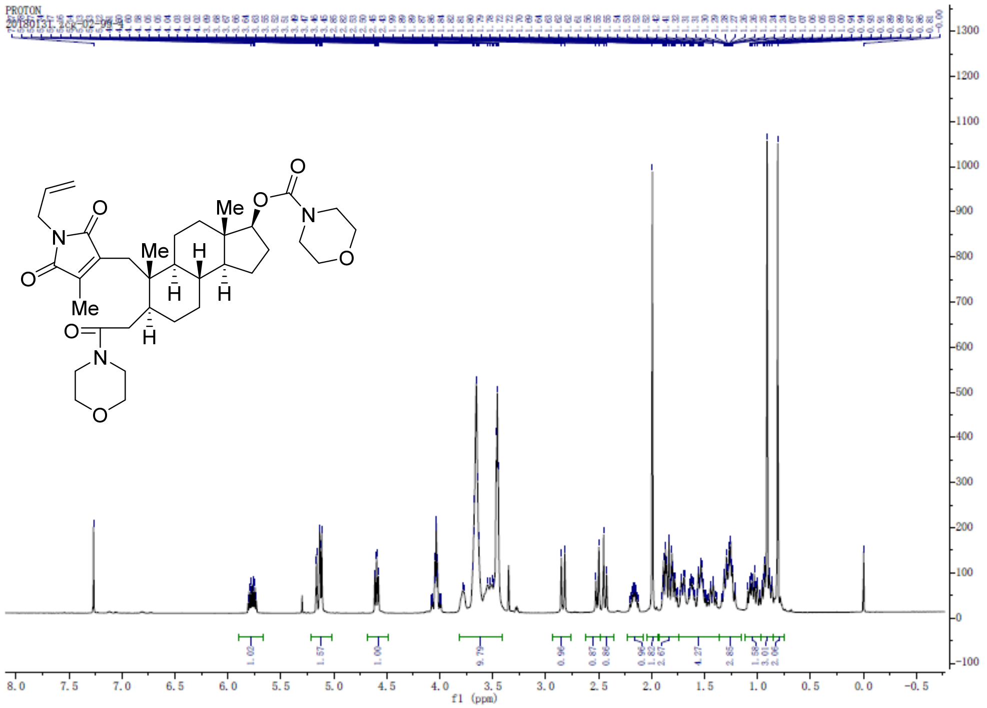

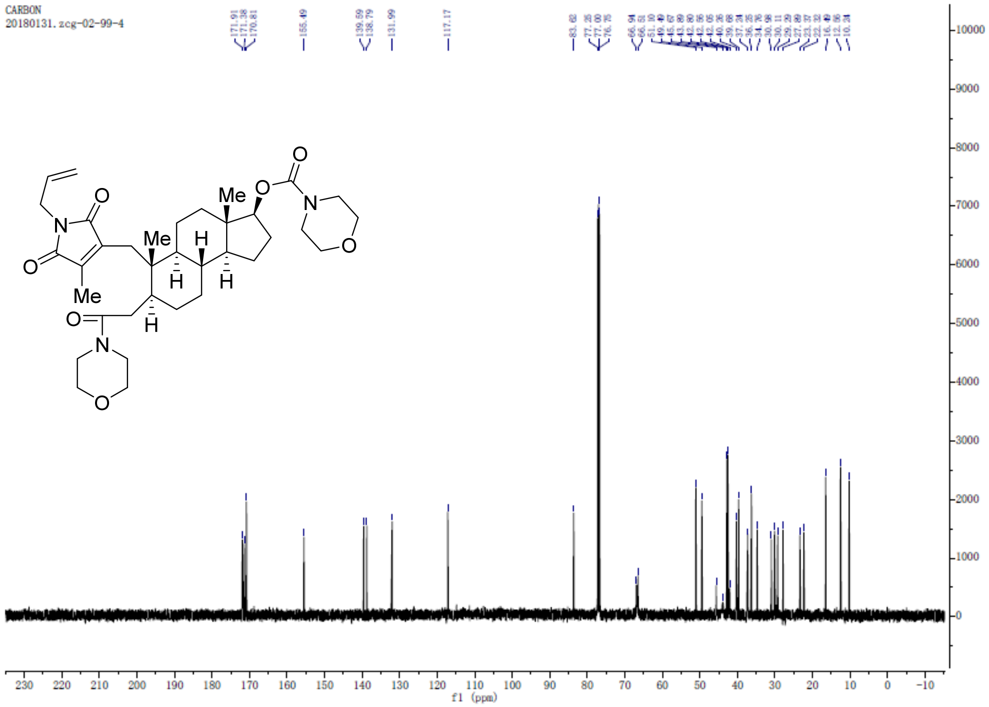

Supplementary Figure 232. ^{1}H and ^{13} C spectra of **53**.

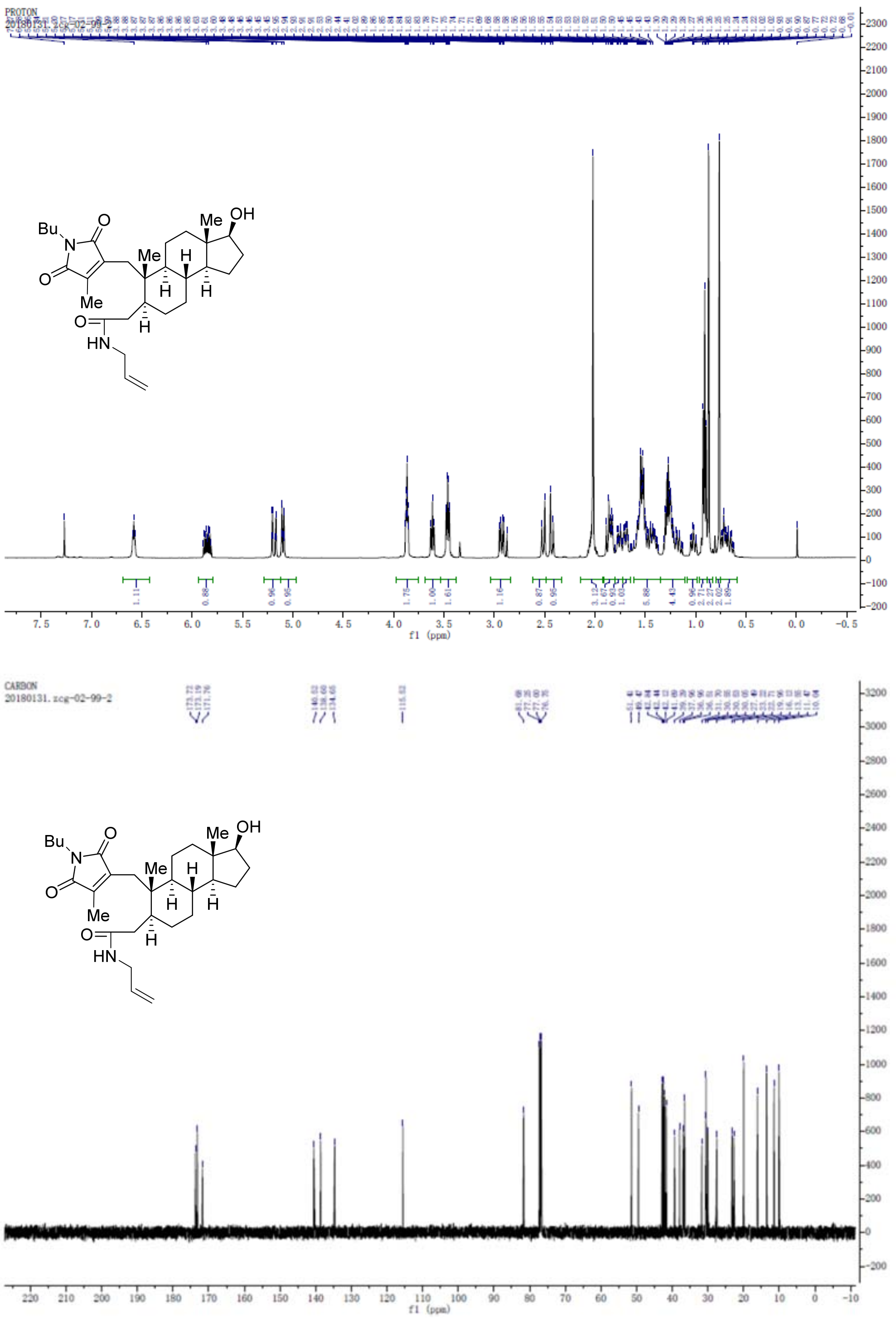

Supplementary Figure 233. ^{1}H and ^{13} C spectra of **52a**.

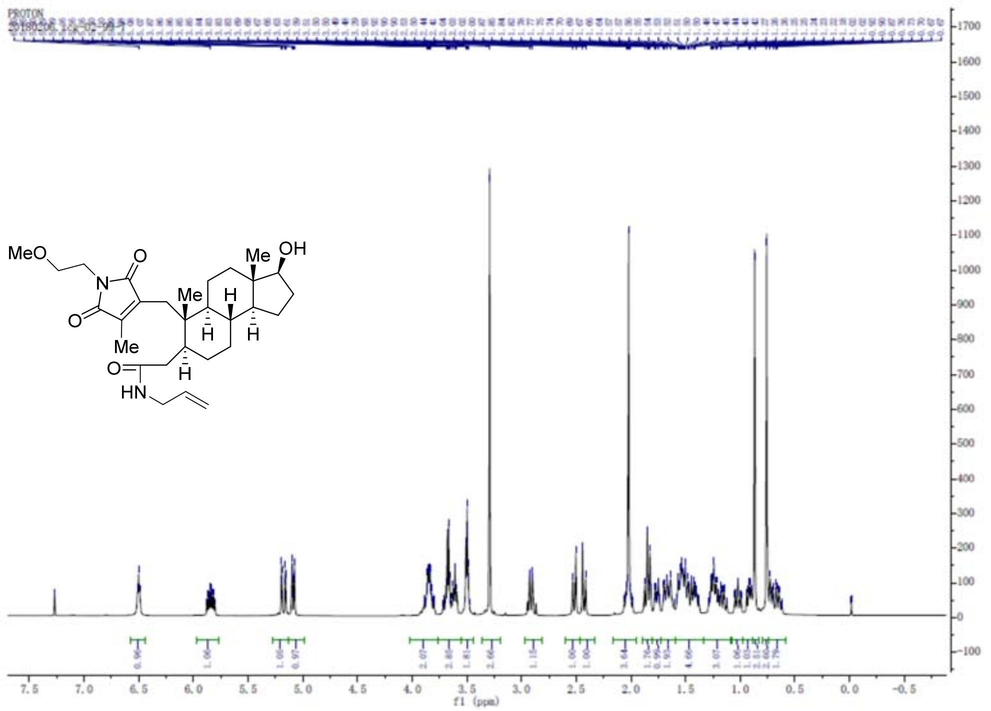

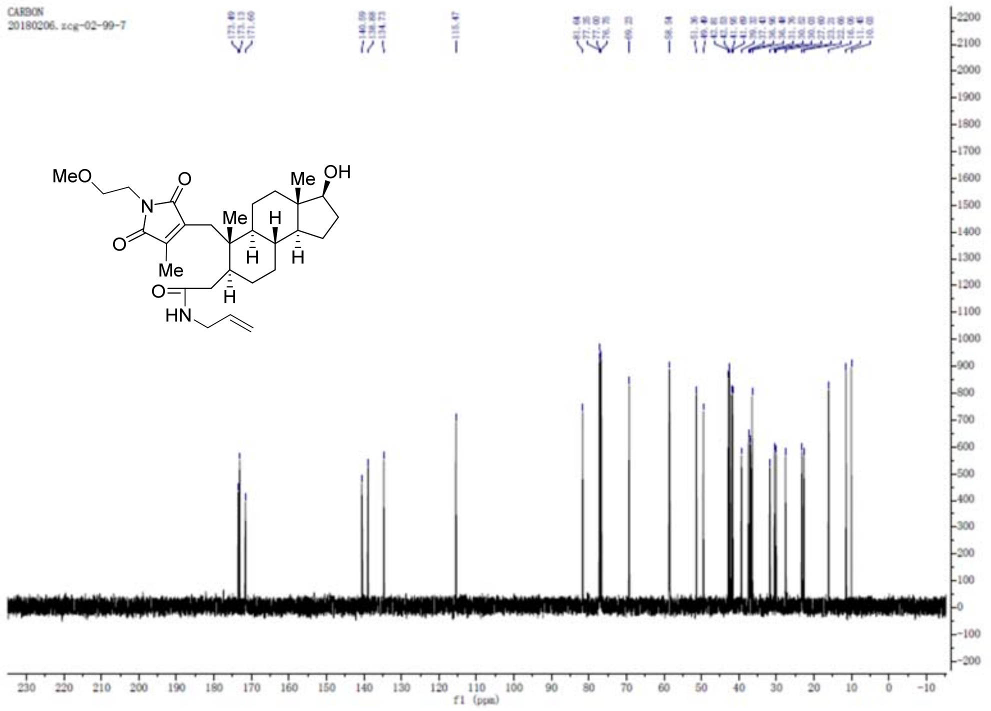

Supplementary Figure 234. ^{1}H and ^{13}C spectra of **52b**.

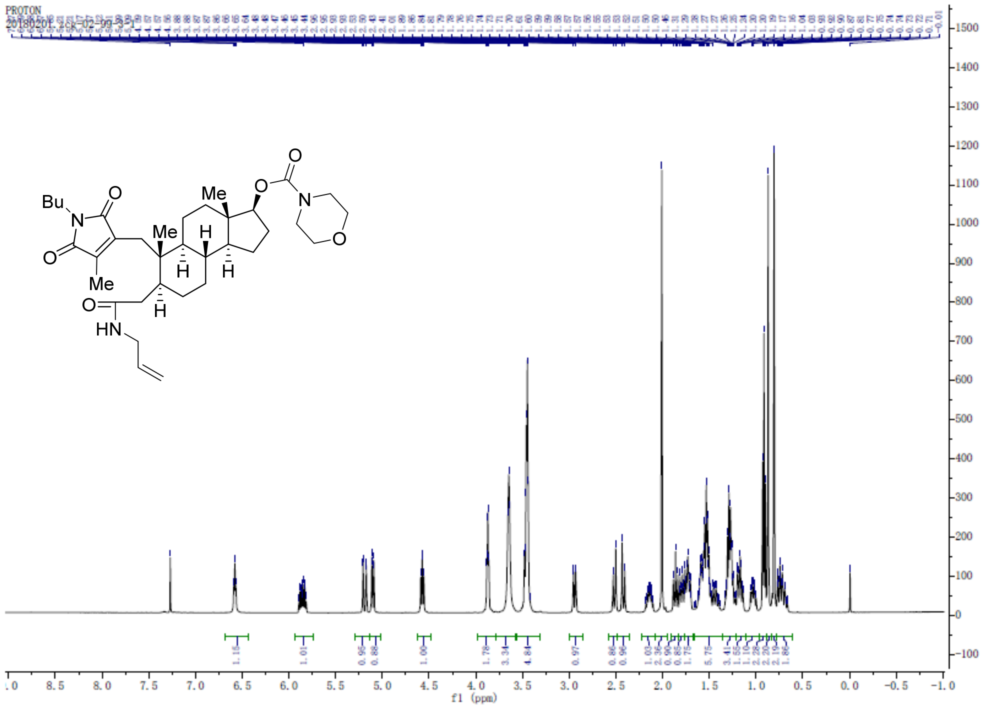

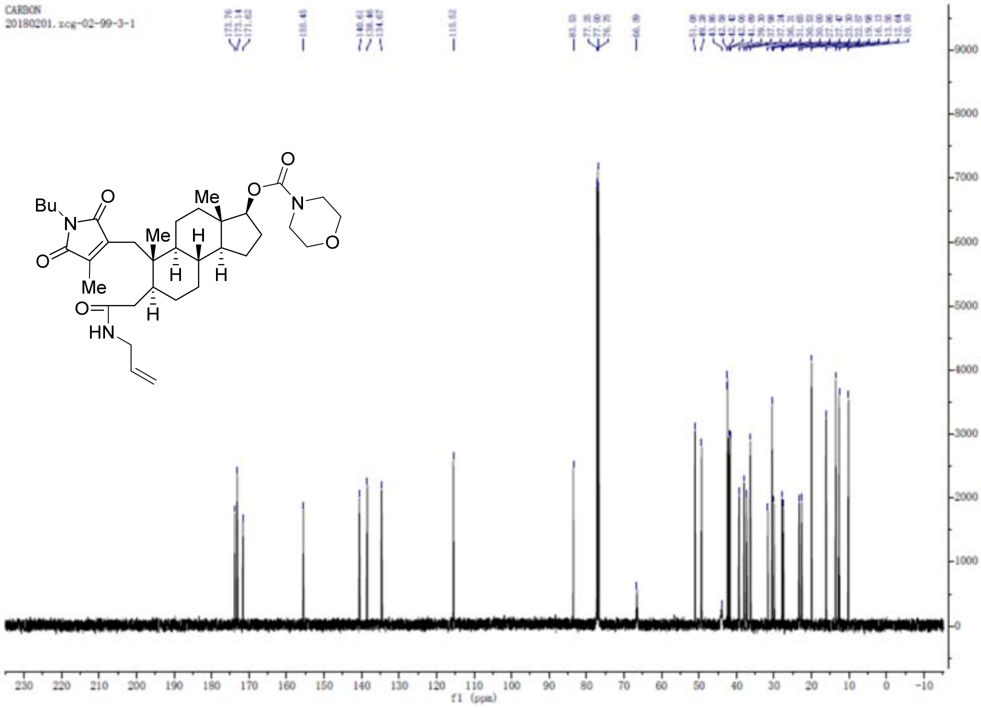

Supplementary Figure 235. ^{1}H and ^{13} C spectra of **54a**.

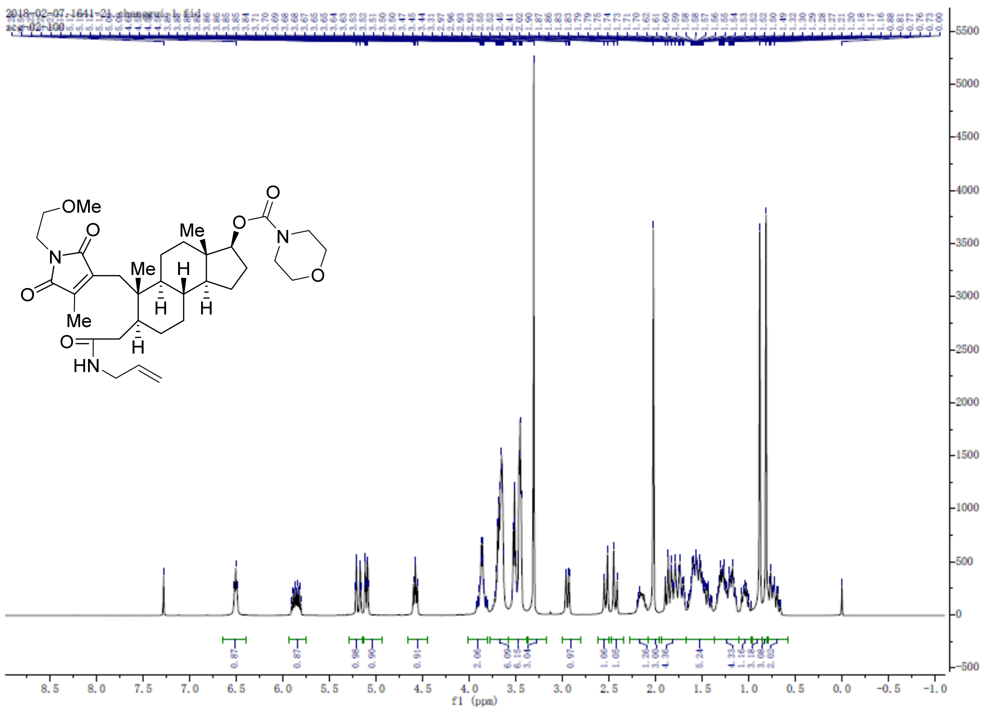

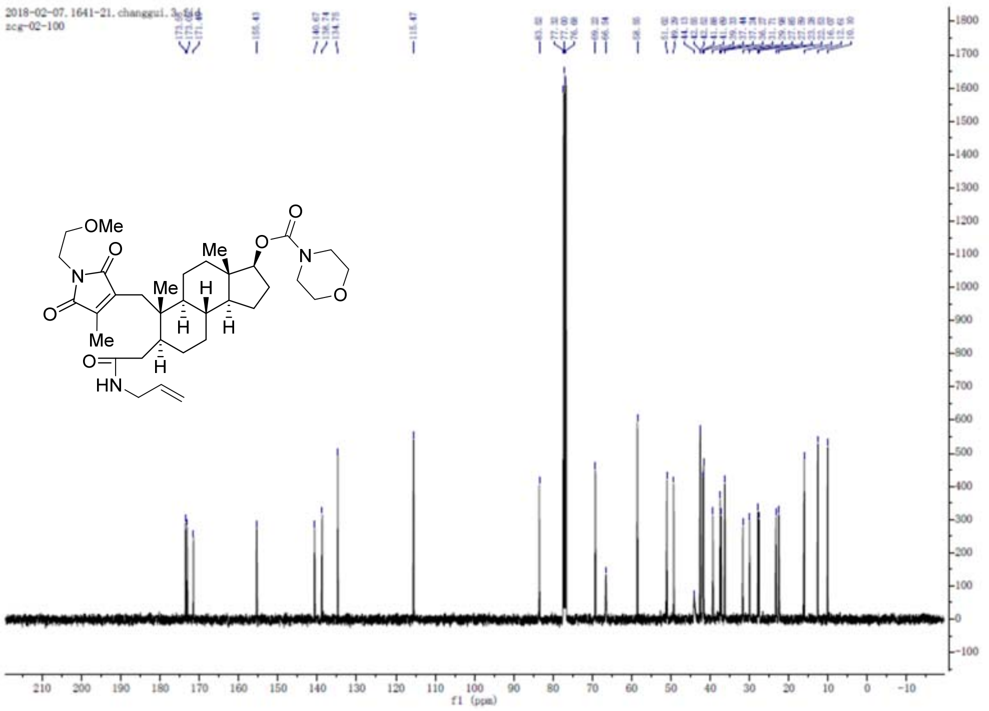

Supplementary Figure 236. ^{1}H and ^{13} C spectra of **54b**.

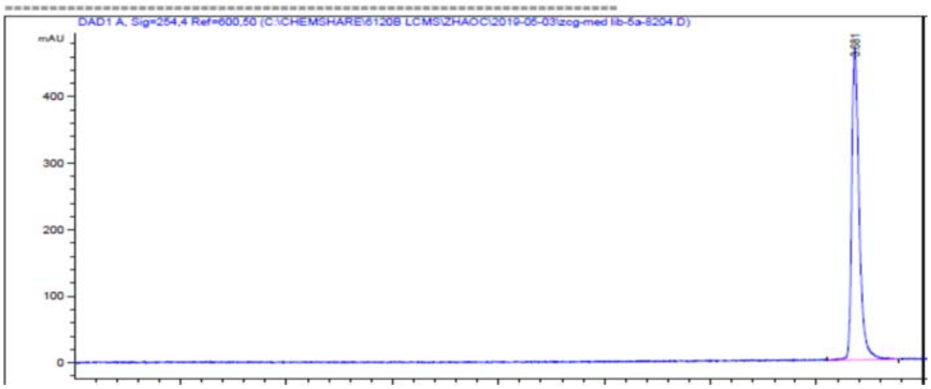

Signal 1: DAD1 A, Sig=254,4 Ref=600,50

Peak #	RetTime [min]	Type	Width [min]	Area [mAU*s]	Height [mAU]	Area %
1	3.681	BB	0.0373	1146.51184	467.73169	100.0000
Totals :				1146.51184	467.73169	

Supplementary Figure 237. LC-MS spectra of **5a**, MS: $(M+Na)^+$, found: 569.2.

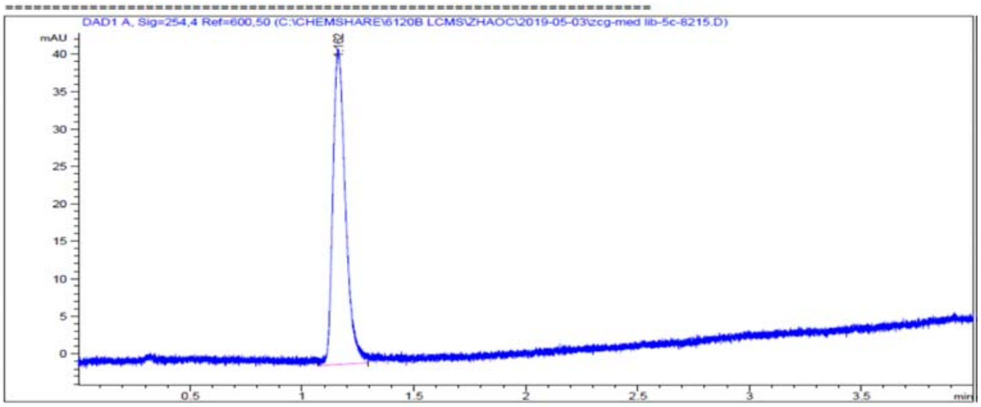

Signal 1: DAD1 A, Sig=254,4 Ref=600,50

Peak #	RetTime [min]	Type	Width [min]	Area [mAU*s]	Height [mAU]	Area %
1	1.162	BB	0.0577	165.32951	41.85899	100.0000
Totals :				165.32951	41.85899	

Supplementary Figure 238. LC-MS spectra of **5c**, MS: $(M+Na)^+$, found: 641.3.

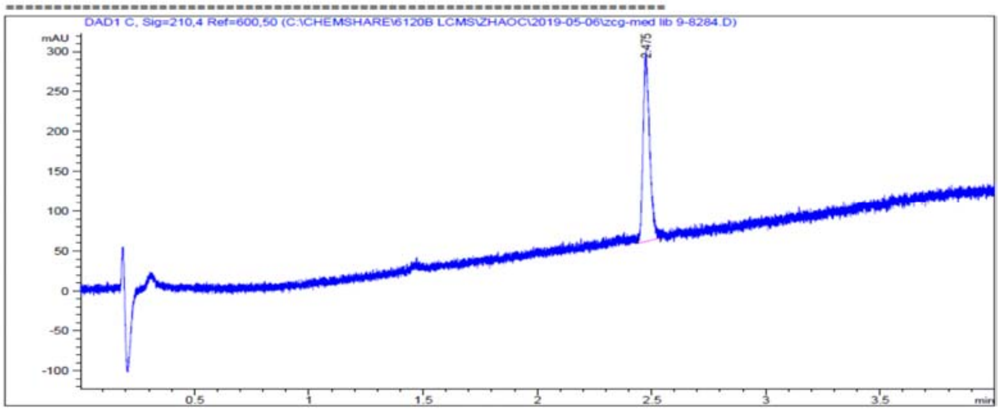

Signal 1: DAD1 C, Sig=210,4 Ref=600,50

Peak #	RetTime [min]	Type	Width [min]	Area [mAU*s]	Height [mAU]	Area %
1	2.475	BB	0.0314	474.60620	226.86649	100.0000
Totals :				474.60620	226.86649	

Supplementary Figure 239. LC-MS spectra of **9**, MS: $(M+H)^+$, found: 362.2.

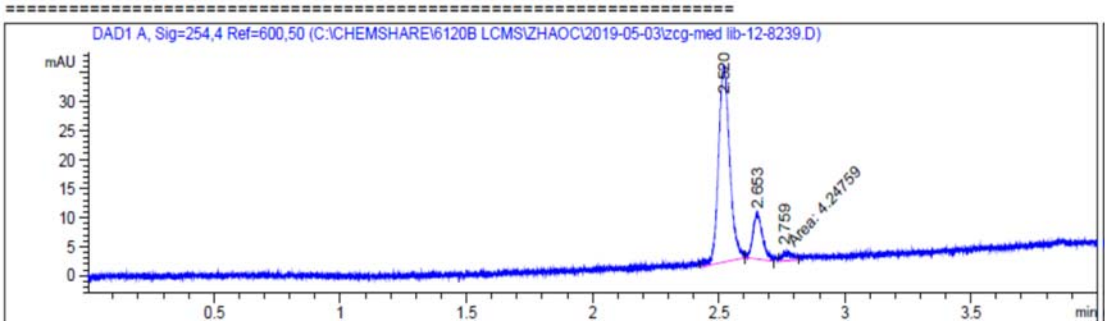

Signal 1: DAD1 A, Sig=254,4 Ref=600,50

Peak #	RetTime [min]	Type	Width [min]	Area [mAU*s]	Height [mAU]	Area %
1	2.520	BB	0.0459	104.70833	33.76105	80.4985
2	2.653	BB	0.0338	21.11897	7.83874	16.2360
3	2.759	MM	0.0381	4.24759	1.85686	3.2655
Totals :				130.07489	43.45665	

Supplementary Figure 240. LC-MS spectra of **12**, MS: $(M+H)^+$, found: 428.1.

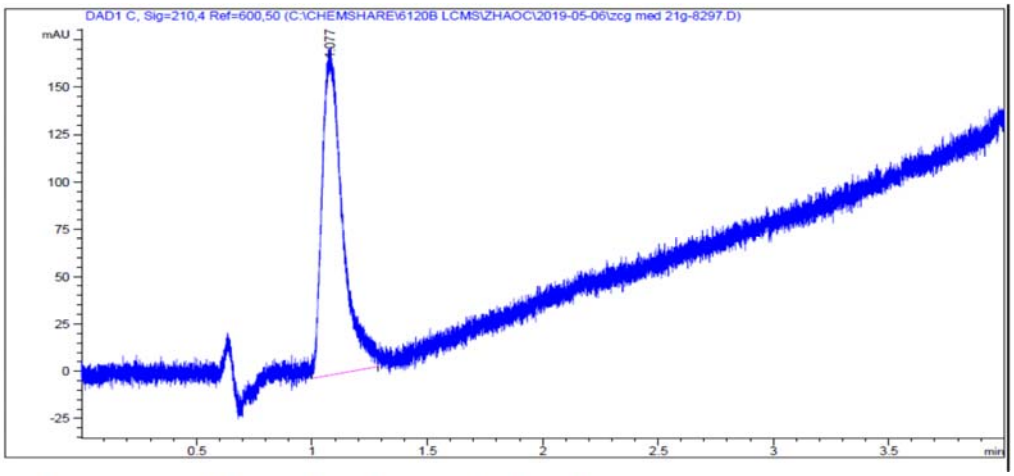

Signal 1: DAD1 C, Sig=210,4 Ref=600,50

Peak #	RetTime [min]	Type	Width [min]	Area [mAU*s]	Height [mAU]	Area %
1	1.077	BB	0.0760	1057.09326	165.84901	100.0000
Totals :				1057.09326	165.84901	

Supplementary Figure 241. LC-MS spectra of **21g**, MS: $(M+H)^+$, found: 320.1.

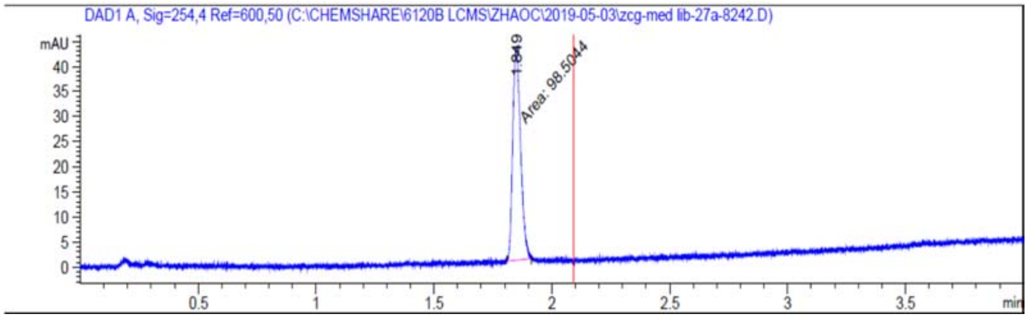

Signal 1: DAD1 A, Sig=254,4 Ref=600,50

Peak #	RetTime [min]	Type	Width [min]	Area [mAU*s]	Height [mAU]	Area %
1	1.849	MM	0.0389	98.50439	42.23854	100.0000
Totals :				98.50439	42.23854	

Supplementary Figure 242. LC-MS spectra of **27a**, MS: $(M+H)^+$, found: 358.1.

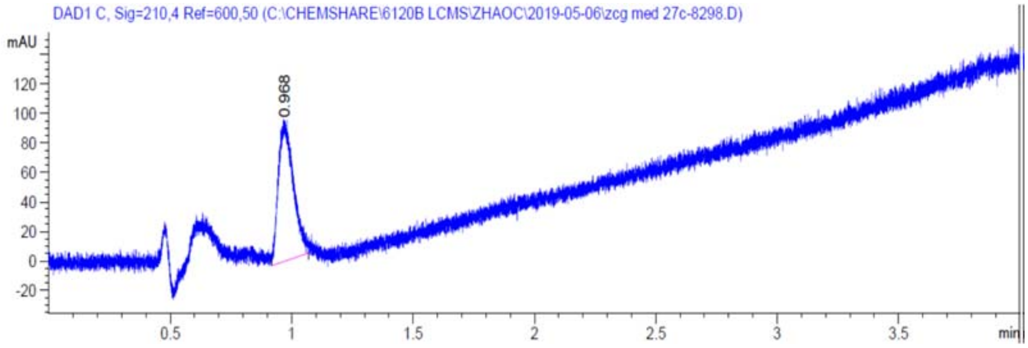

Peak #	RetTime [min]	Type	Width [min]	Area [mAU*s]	Height [mAU]	Area %
1	0.968	BB	0.0522	410.53693	94.15156	100.0000
Totals :				410.53693	94.15156	

Supplementary Figure 243. LC-MS spectra of **27c**, MS: $(M+H)^+$, found: 318.1.

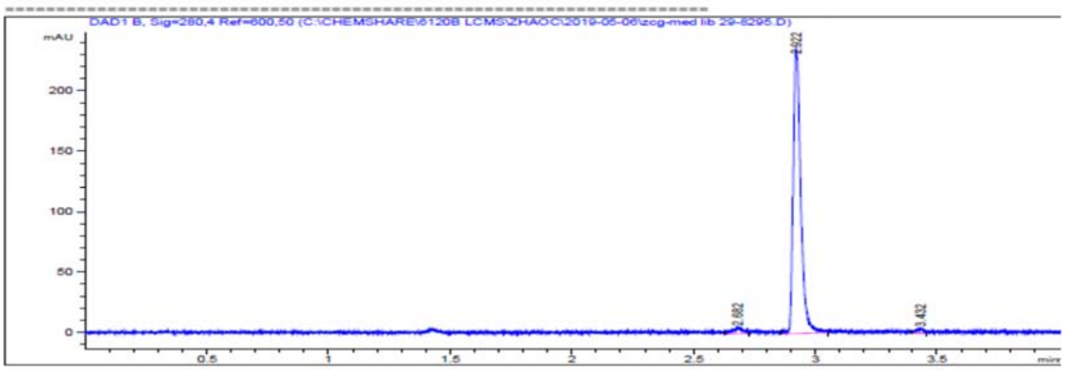

Signal 1: DAD1 B, Sig=280,4 Ref=600,50

Peak #	RetTime [min]	Type	Width [min]	Area [mAU*s]	Height [mAU]	Area %
1	2.682	BB	0.0395	16.22314	4.87850	3.0278
2	2.922	BB	0.0332	511.30566	236.34161	95.4272
3	3.432	BB	0.0298	8.27852	3.60452	1.5451
Totals :				535.80732	244.82463	

Supplementary Figure 245. LC-MS spectra of **29**, MS: $(M+H)^+$, found: 316.1.

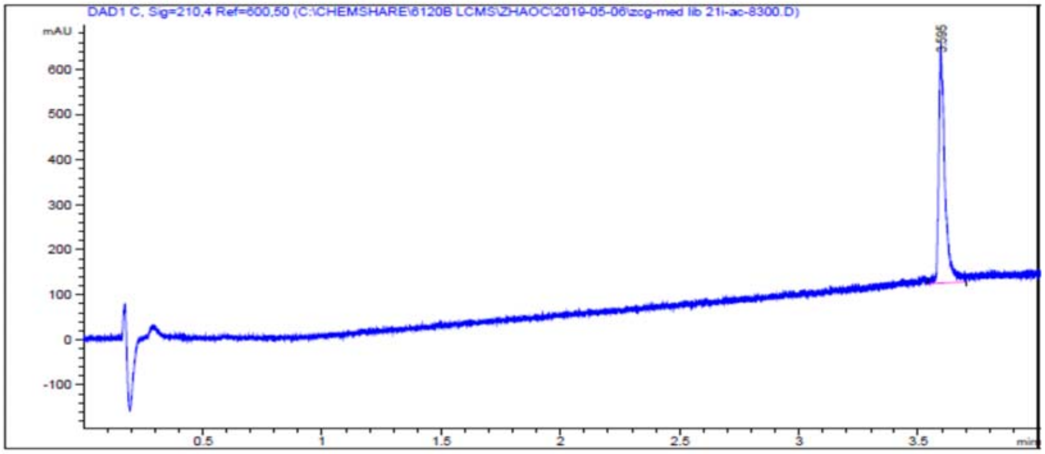

Signal 1: DAD1 C, Sig=210,4 Ref=600,50

Peak #	RetTime [min]	Type	Width [min]	Area [mAU*s]	Height [mAU]	Area %
1	3.595	BB	0.0273	950.97528	512.08533	100.0000
Totals :				950.97528	512.08533	

Supplementary Figure 246. LC-MS spectra of **41bb**, MS: (M+H)$^+$, found: 460.3.

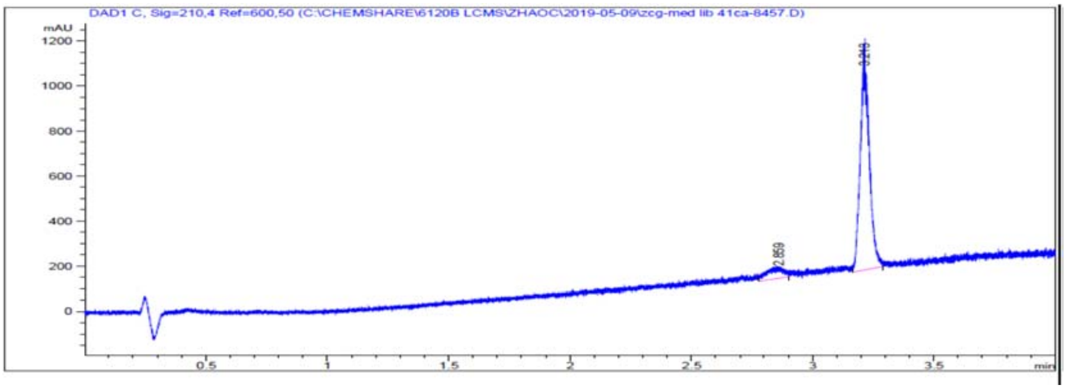

Signal 1: DAD1 C, Sig=210,4 Ref=600,50

Peak #	RetTime [min]	Type	Width [min]	Area [mAU*s]	Height [mAU]	Area %
1	2.859	BB	0.0635	238.23796	44.25521	8.8085
2	3.213	BB	0.0352	2466.40503	893.97723	91.1915
Totals :				2704.64299	938.23244	

Supplementary Figure 247. LC-MS spectra of **41ca**, MS: (M+H)$^+$, found: 559.2.

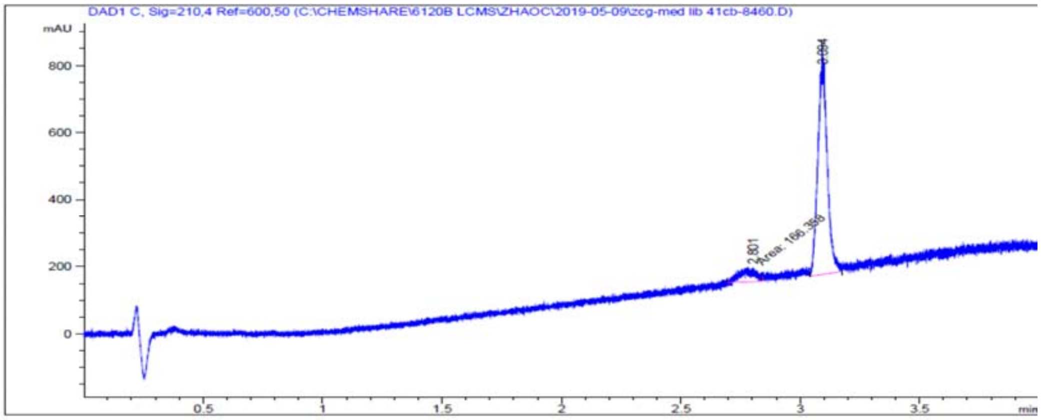

Signal 1: DAD1 C, Sig=210,4 Ref=600,50

Peak #	RetTime [min]	Type	Width [min]	Area [mAU*s]	Height [mAU]	Area %
1	2.801	MM	0.0637	166.35771	43.49775	8.6168
2	3.094	BB	0.0383	1764.27161	617.72681	91.3832
Totals :				1930.62932	661.22456	

Supplementary Figure 248. LC-MS spectra of **41cb**, MS: (M+H)$^{+}$, found: 573.2.

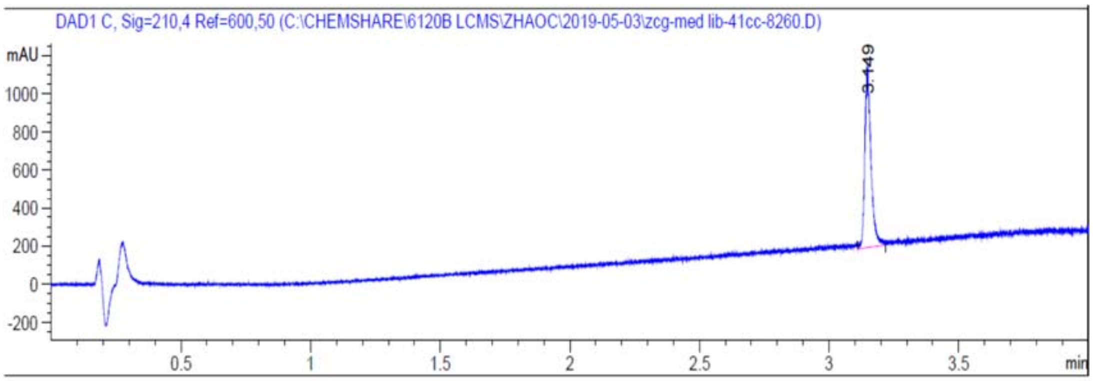

Signal 3: DAD1 C, Sig=210,4 Ref=600,50

Peak #	RetTime [min]	Type	Width [min]	Area [mAU*s]	Height [mAU]	Area %
1	3.149	BB	0.0263	1550.32410	892.52545	100.0000
Totals :				1550.32410	892.52545	

Supplementary Figure 249. LC-MS spectra of **41cc**, MS: (M+H)$^{+}$, found: 573.2.

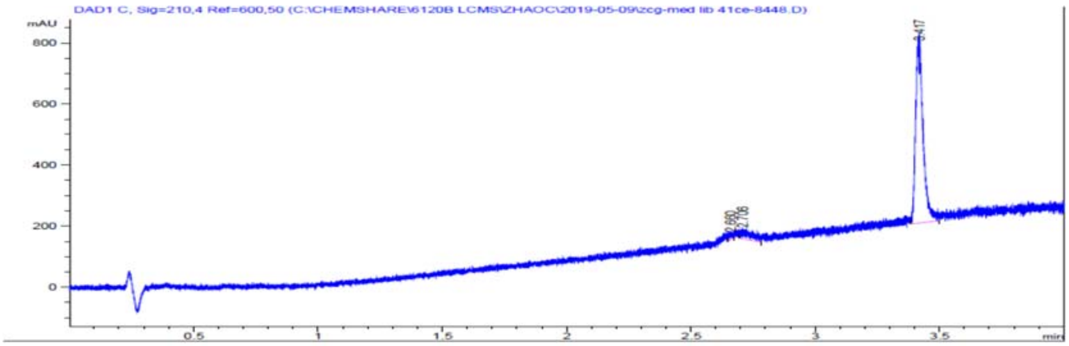

Signal 1: DAD1 C, Sig=210,4 Ref=600,50

Peak #	RetTime [min]	Type	Width [min]	Area [mAU*s]	Height [mAU]	Area %
1	2.660	BB	0.0164	21.96706	17.06423	1.6025
2	2.706	BB	0.0364	88.19725	29.34437	6.4338
3	3.417	BB	0.0299	1260.67590	591.05695	91.9637
Totals :				1370.84022	637.46554	

Supplementary Figure 250. LC-MS spectra of **41ce**, MS: (M+H)$^+$, found: 557.2.

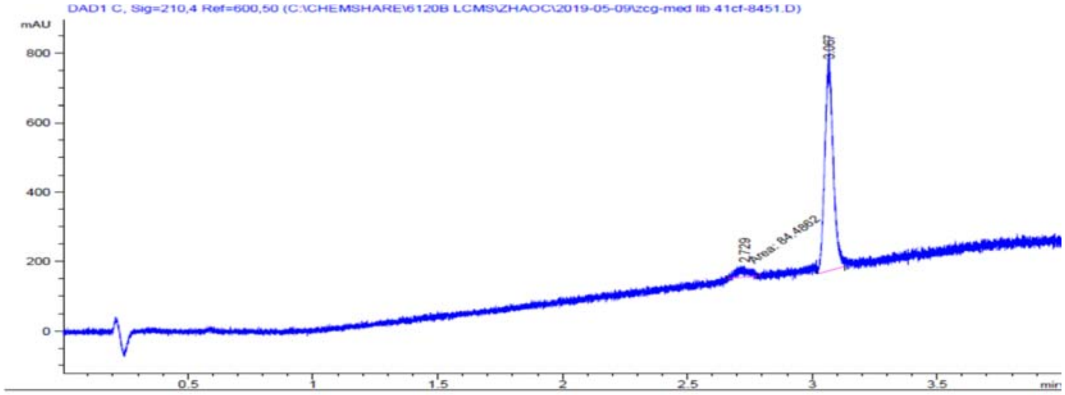

Signal 1: DAD1 C, Sig=210,4 Ref=600,50

Peak #	RetTime [min]	Type	Width [min]	Area [mAU*s]	Height [mAU]	Area %
1	2.729	MM	0.0460	84.48620	33.42954	5.5783
2	3.067	BB	0.0330	1430.05762	602.83209	94.4217
Totals :				1514.54382	636.26163	

Supplementary Figure 251. LC-MS spectra of **41cf**, MS: (M+H)$^+$, found: 529.3.

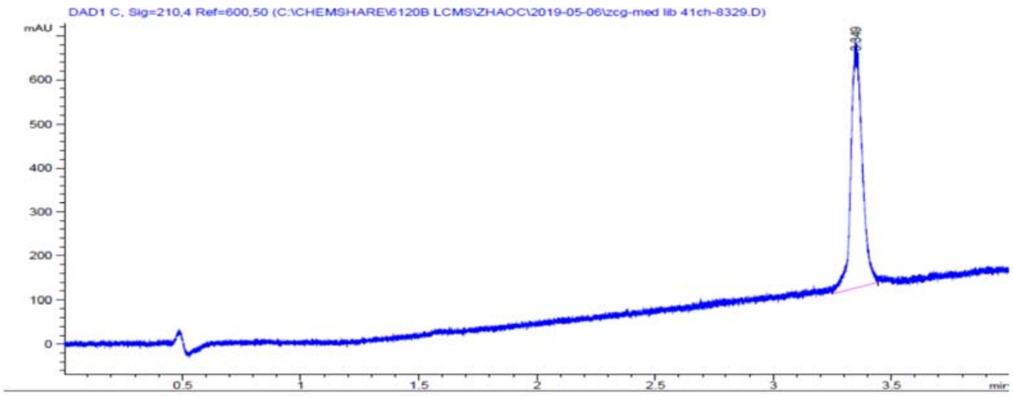

Signal 1: DAD1 C, Sig=210,4 Ref=600,50

Peak #	RetTime [min]	Type	Width [min]	Area [mAU*s]	Height [mAU]	Area %
1	3.349	BB	0.0471	1861.49243	532.77356	100.0000
Totals :				1861.49243	532.77356	

Supplementary Figure 252. LC-MS spectra of **41ch**, MS: $(M+H)^+$, found: 593.3.

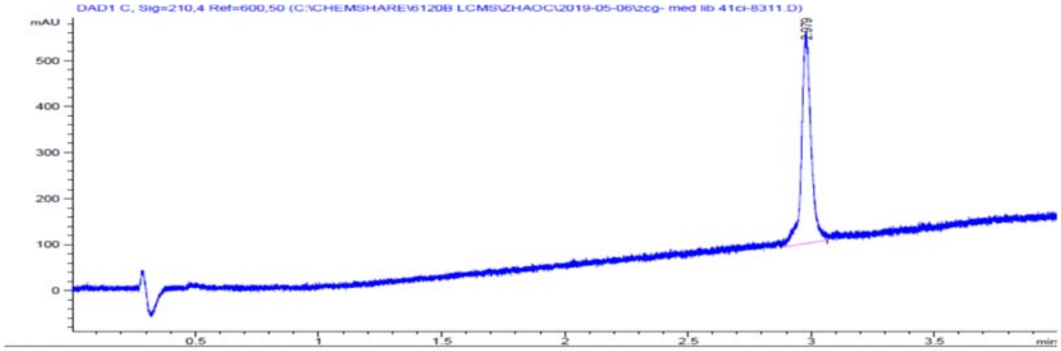

Signal 1: DAD1 C, Sig=210,4 Ref=600,50

Peak #	RetTime [min]	Type	Width [min]	Area [mAU*s]	Height [mAU]	Area %
1	2.979	BB	0.0408	1235.28418	439.42145	100.0000
Totals :				1235.28418	439.42145	

Supplementary Figure 253. LC-MS spectra of **41ci**, MS: $(M+H)^+$, found: 533.2.

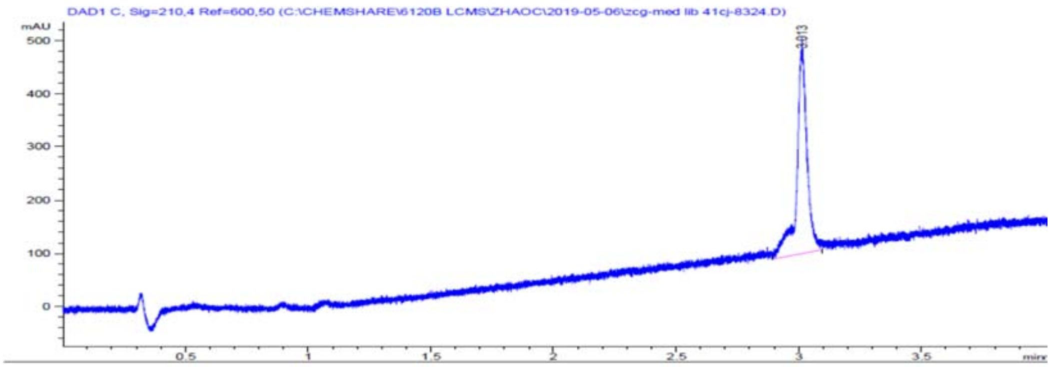

Signal 1: DAD1 C, Sig=210,4 Ref=600,50

Peak #	RetTime [min]	Type	Width [min]	Area [mAU*s]	Height [mAU]	Area %
1	3.013	BB	0.0419	1139.68005	380.77518	100.0000
Totals :				1139.68005	380.77518	

Supplementary Figure 254. LC-MS spectra of **41cj**, MS: $(M+H)^+$, found: 547.2.

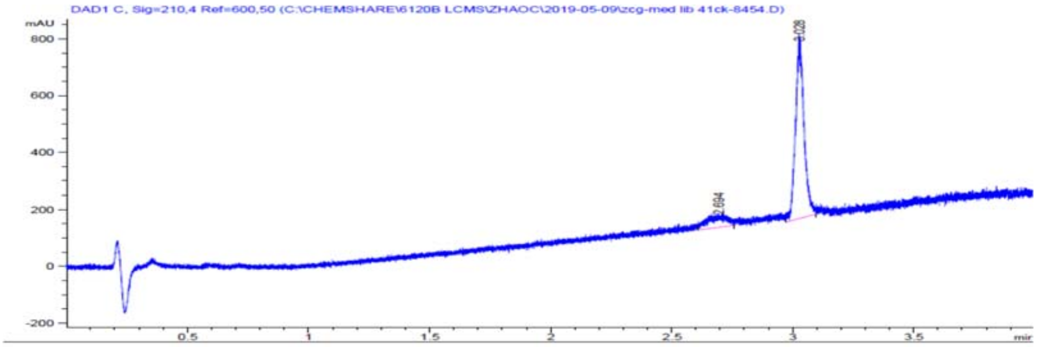

Signal 1: DAD1 C, Sig=210,4 Ref=600,50

Peak #	RetTime [min]	Type	Width [min]	Area [mAU*s]	Height [mAU]	Area %
1	2.694	BB	0.0752	231.61143	37.62191	13.0843
2	3.028	BB	0.0316	1538.53955	613.79236	86.9157
Totals :				1770.15099	651.41427	

Supplementary Figure 255. LC-MS spectra of **41ck**, MS: $(M+H)^+$, found: 573.2.

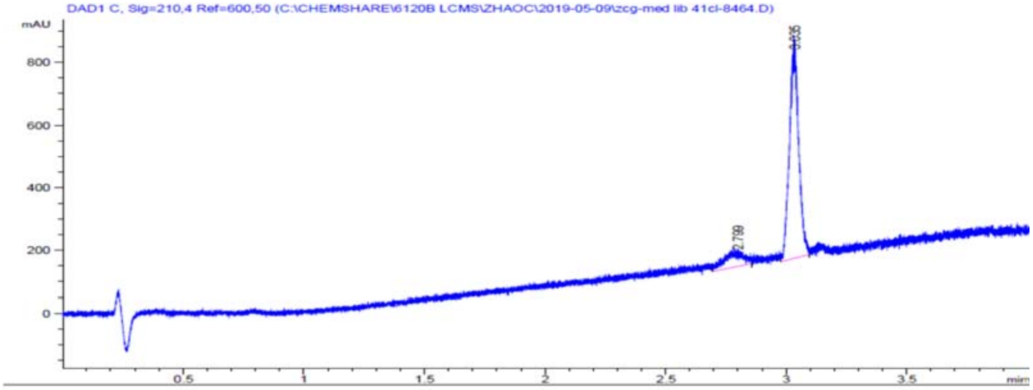

Signal 1: DAD1 C, Sig=210,4 Ref=600,50

Peak #	RetTime [min]	Type	Width [min]	Area [mAU*s]	Height [mAU]	Area %
1	2.799	BB	0.0744	280.05371	45.12654	13.3822
2	3.035	BB	0.0349	1812.67896	657.09686	86.6178

Totals : 2092.73267 702.22341

Supplementary Figure 256. LC-MS spectra of **41cl**, MS: $(M+H)^+$, found: 575.3.

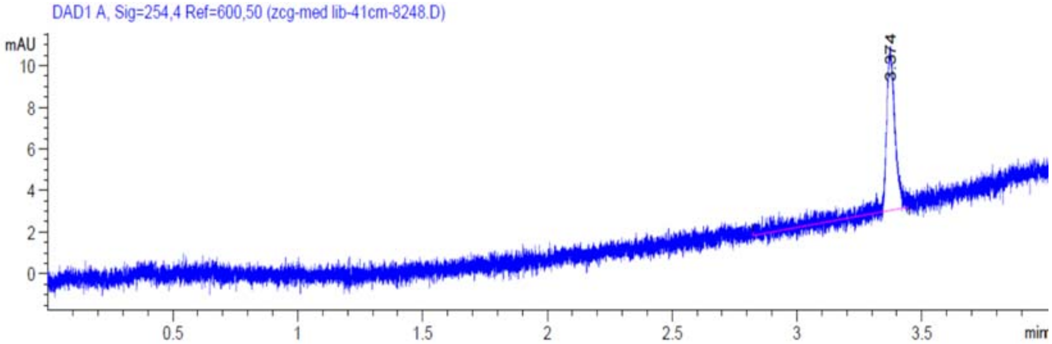

Signal 1: DAD1 A, Sig=254,4 Ref=600,50

Peak #	RetTime [min]	Type	Width [min]	Area [mAU*s]	Height [mAU]	Area %
1	3.373	BB	0.0387	20.24950	7.96697	100.0000

Totals : 20.24950 7.96697

Supplementary Figure 257. LC-MS spectra of **41cm**, MS: $(M+H)^+$, found: 609.2.

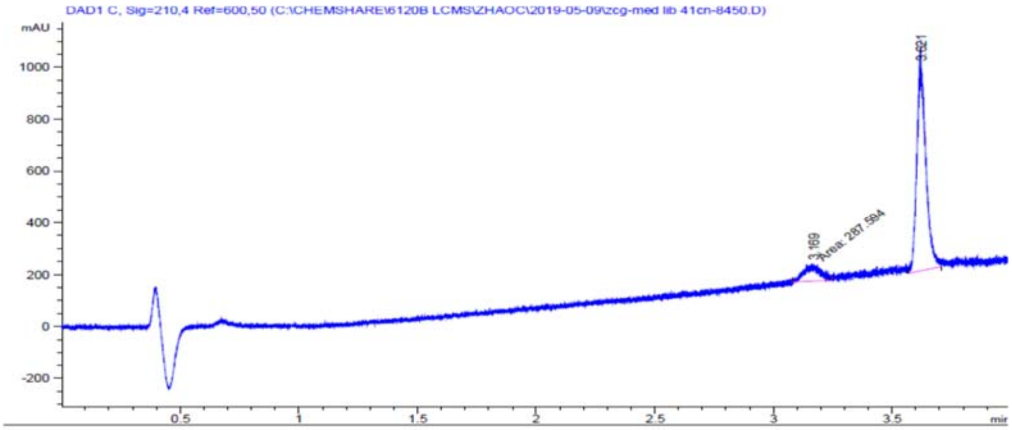

Signal 1: DAD1 C, Sig=210,4 Ref=600,50

Peak #	RetTime [min]	Type	Width [min]	Area [mAU*s]	Height [mAU]	Area %
1	3.169	MM	0.0691	287.59369	69.34865	11.8708
2	3.621	BB	0.0326	2135.10474	796.69647	88.1292
Totals :				2422.69843	866.04512	

Supplementary Figure 258. LC-MS spectra of **41cn**, MS: $(M+H)^+$, found: 557.2.

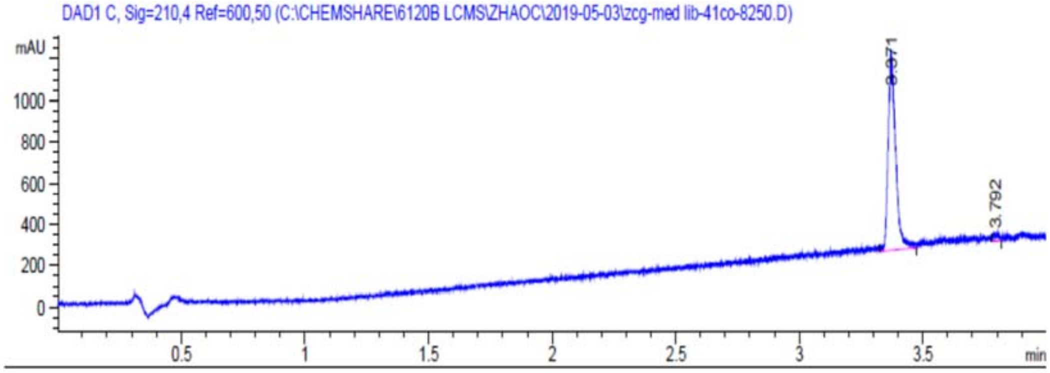

Signal 3: DAD1 C, Sig=210,4 Ref=600,50

Peak #	RetTime [min]	Type	Width [min]	Area [mAU*s]	Height [mAU]	Area %
1	3.371	BB	0.0305	1999.68420	879.60547	97.2583
2	3.792	BB	0.0242	56.37052	30.53646	2.7417
Totals :				2056.05472	910.14193	

Supplementary Figure 259. LC-MS spectra of **41co**, MS: $(M+H)^+$, found: 623.2.

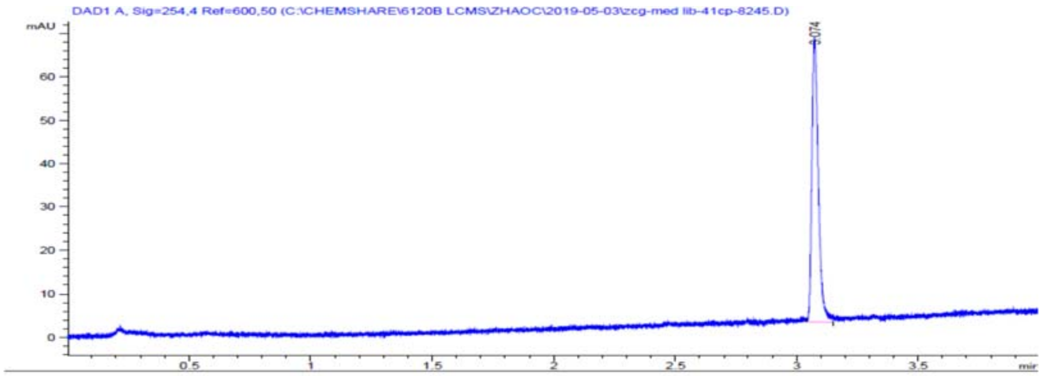

Signal 1: DAD1 A, Sig=254,4 Ref=600,50

Peak #	RetTime [min]	Type	Width [min]	Area [mAU*s]	Height [mAU]	Area %
1	3.074	BB	0.0287	121.62946	65.44843	100.0000
Totals :				121.62946	65.44843	

Supplementary Figure 260. LC-MS spectra of **41cp**, MS: $(M+H)^+$, found: 623.2.

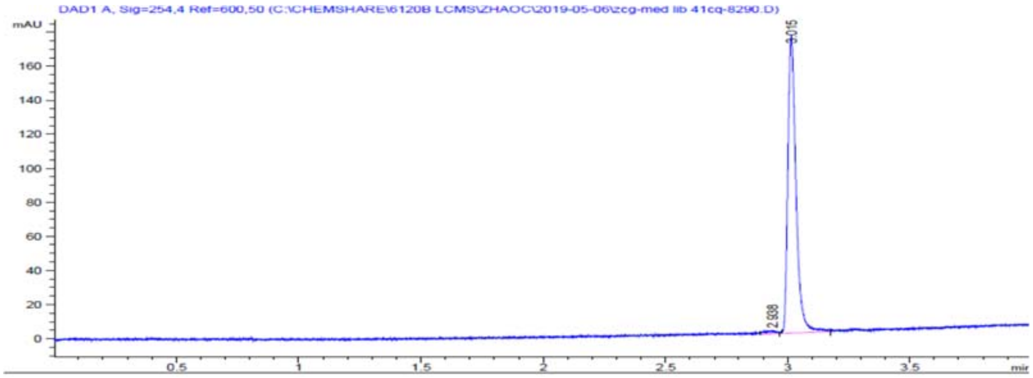

Signal 1: DAD1 A, Sig=254,4 Ref=600,50

Peak #	RetTime [min]	Type	Width [min]	Area [mAU*s]	Height [mAU]	Area %
1	2.938	BB	0.0412	5.94917	1.71070	1.4464
2	3.015	BB	0.0354	405.35452	174.59377	98.5536
Totals :				411.30370	176.30446	

Supplementary Figure 261. LC-MS spectra of **41cq**, MS: $(M+H)^+$, found: 653.3.

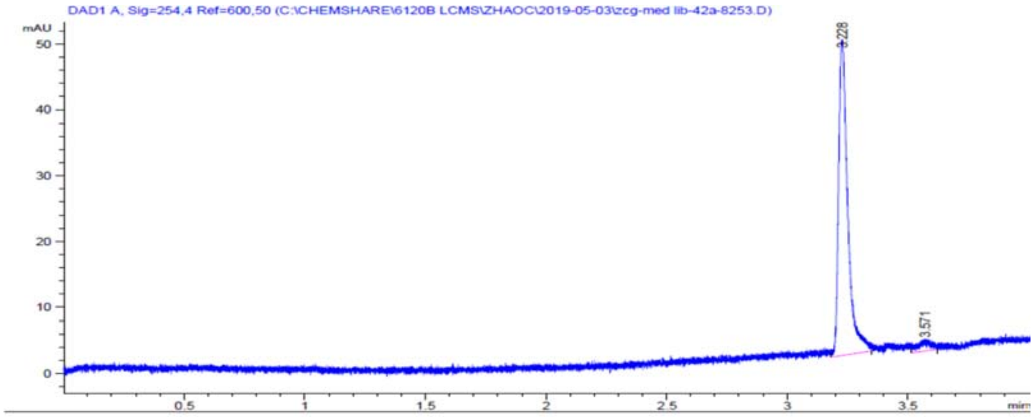

Peak #	RetTime [min]	Type	Width [min]	Area [mAU*s]	Height [mAU]	Area %
1	3.228	BB	0.0400	126.64327	47.66279	95.4249
2	3.571	BB	0.0452	6.07187	1.61405	4.5751
Totals :				132.71514	49.27685	

Supplementary Figure 262. LC-MS spectra of **42a**, MS: $(M+H)^+$, found: 439.2.

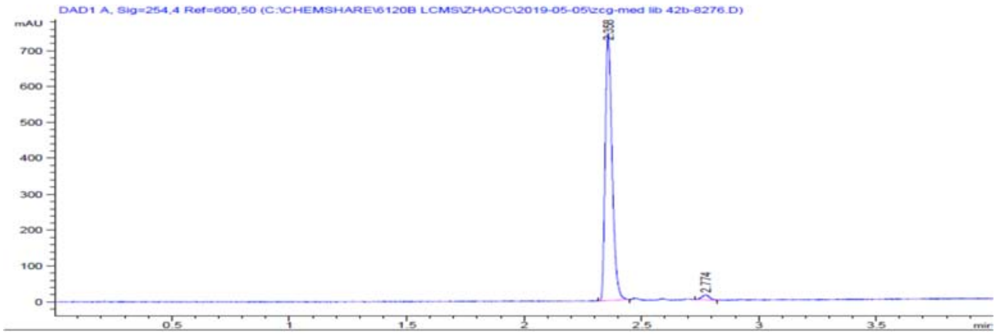

Signal 1: DAD1 A, Sig=254,4 Ref=600,50

Peak #	RetTime [min]	Type	Width [min]	Area [mAU*s]	Height [mAU]	Area %
1	2.358	BB	0.0315	1525.38269	745.35980	97.9674
2	2.774	BB	0.0345	31.64767	13.91463	2.0326
Totals :				1557.03036	759.27443	

Supplementary Figure 267. LC-MS spectra of **42b**, MS: $(M+H)^+$, found: 439.2.

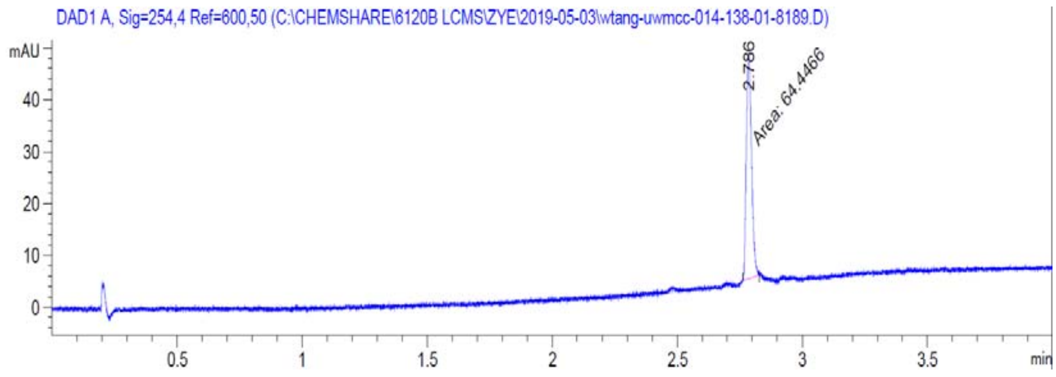

Signal 1: DAD1 A, Sig=254,4 Ref=600,50

Peak #	RetTime [min]	Type	Width [min]	Area [mAU*s]	Height [mAU]	Area %
1	2.786	MM	0.0250	64.44662	43.03405	100.0000
Totals :				64.44662	43.03405	

Supplementary Figure 268. LC-MS spectra of **43a**: retention time, 2.786 min; MS $(M+H)^+$ found 429.1

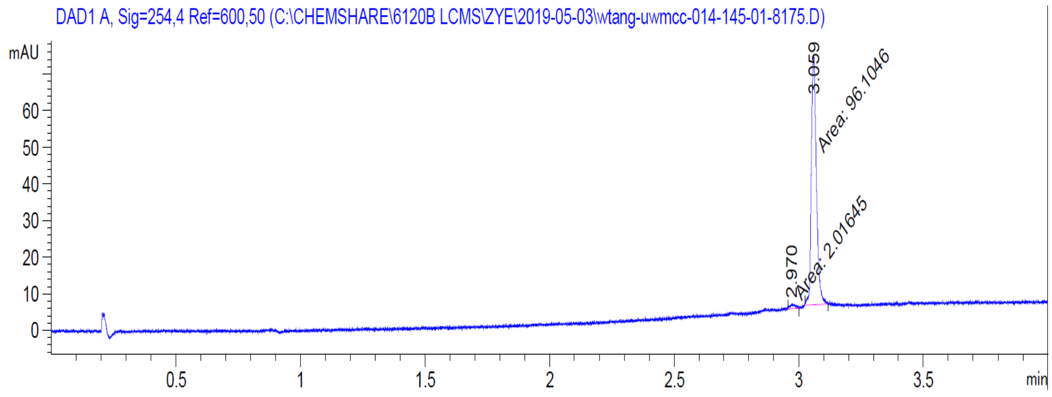

Signal 1: DAD1 A, Sig=254,4 Ref=600,50

Peak #	RetTime [min]	Type	Width [min]	Area [mAU*s]	Height [mAU]	Area %
1	2.970	MM	0.0236	2.01645	1.42702	2.0551
2	3.059	MM	0.0236	96.10456	67.79678	97.9449
Totals :				98.12101	69.22381	

Supplementary Figure 269. LC-MS spectra of **43b**: retention time, 3.059 min; MS $(M+H)^+$ found 427.2

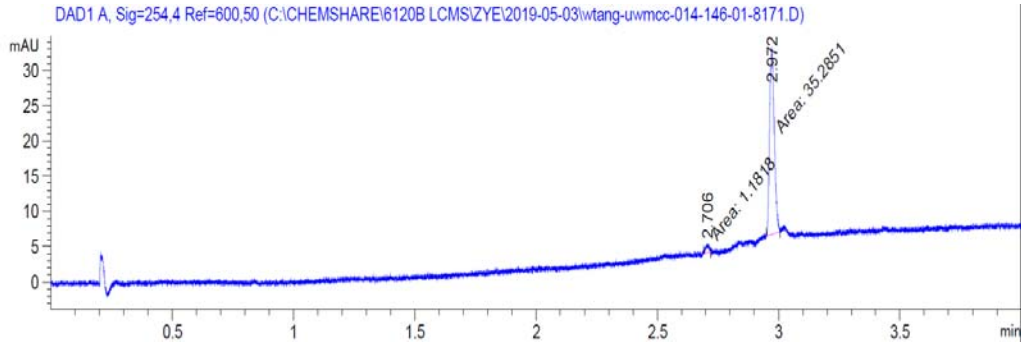

Signal 1: DAD1 A, Sig=254,4 Ref=600,50

Peak #	RetTime [min]	Type	Width [min]	Area [mAU*s]	Height [mAU]	Area %
1	2.706	MM	0.0163	1.18180	1.20795	3.2407
2	2.972	MM	0.0226	35.28513	26.02234	96.7593
Totals :				36.46693	27.23029	

Supplementary Figure 270. LC-MS spectra of **43c**: retention time, 2.972 min; MS $(M+H)^+$ found 413.1

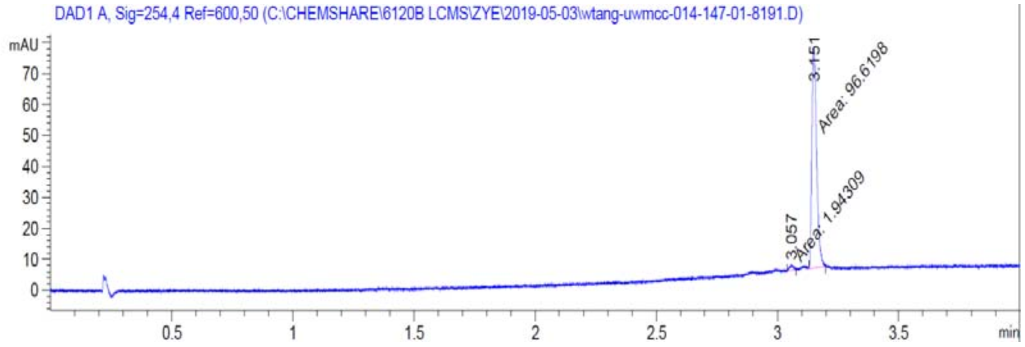

Signal 1: DAD1 A, Sig=254,4 Ref=600,50

Peak #	RetTime [min]	Type	Width [min]	Area [mAU*s]	Height [mAU]	Area %
1	3.057	MM	0.0200	1.94309	1.61833	1.9714
2	3.151	MM	0.0226	96.61982	71.15961	98.0286
Totals :				98.56291	72.77795	

Supplementary Figure 271. LC-MS spectra of **43d**: retention time, 3.151 min; MS $(M+H)^+$ found 475.1

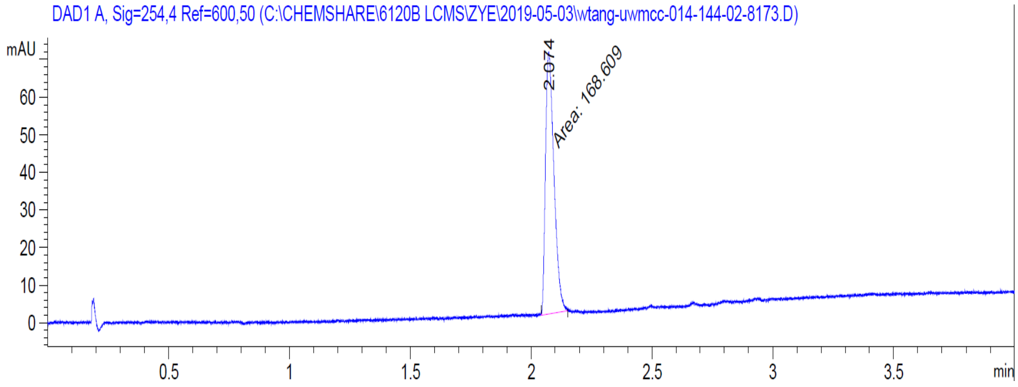

Signal 1: DAD1 A, Sig=254,4 Ref=600,50

Peak #	RetTime [min]	Type	Width [min]	Area [mAU*s]	Height [mAU]	Area %
1	2.074	MM	0.0405	168.60870	69.45885	100.0000
Totals :				168.60870	69.45885	

Supplementary Figure 272. LC-MS spectra of **43e**: retention time, 2.074 min; MS $(M+H)^+$ found 442.2

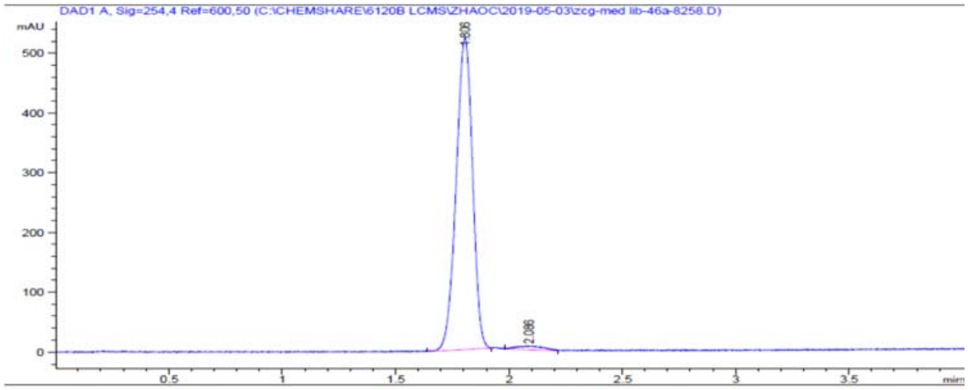

Signal 1: DAD1 A, Sig=254,4 Ref=600,50

Peak #	RetTime [min]	Type	Width [min]	Area [mAU*s]	Height [mAU]	Area %
1	1.806	BB	0.0788	2627.26758	521.23181	98.1199
2	2.086	BB	0.1020	50.34156	5.83639	1.8801
Totals :				2677.60914	527.06820	

Supplementary Figure 273. LC-MS spectra of **46a**, MS: $(M+H)^+$, found: 619.2.

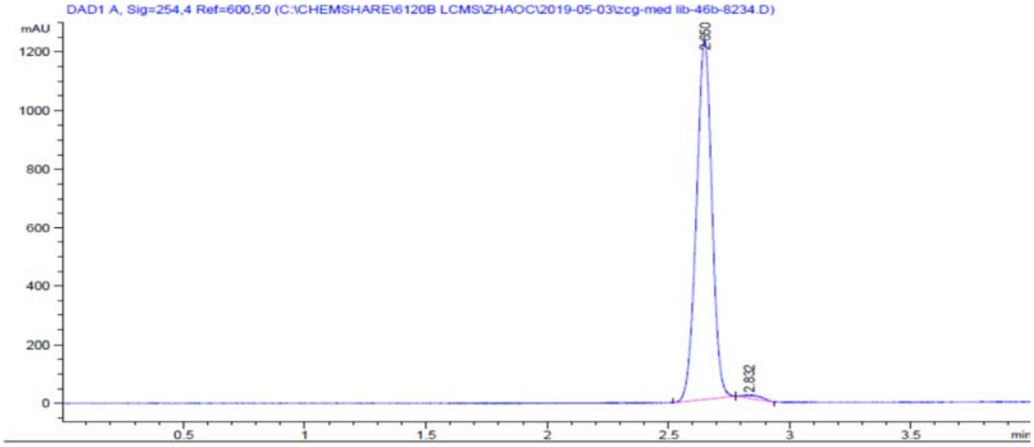

Peak #	RetTime [min]	Type	Width [min]	Area [mAU*s]	Height [mAU]	Area %
1	2.650	BB	0.0693	5447.17773	1222.09045	98.8819
2	2.832	BB	0.0762	61.59166	9.64379	1.1181
Totals :				5508.76939	1231.73425	

Supplementary Figure 274. LC-MS spectra of **46b**, MS: $(M+H)^+$, found: 653.2.

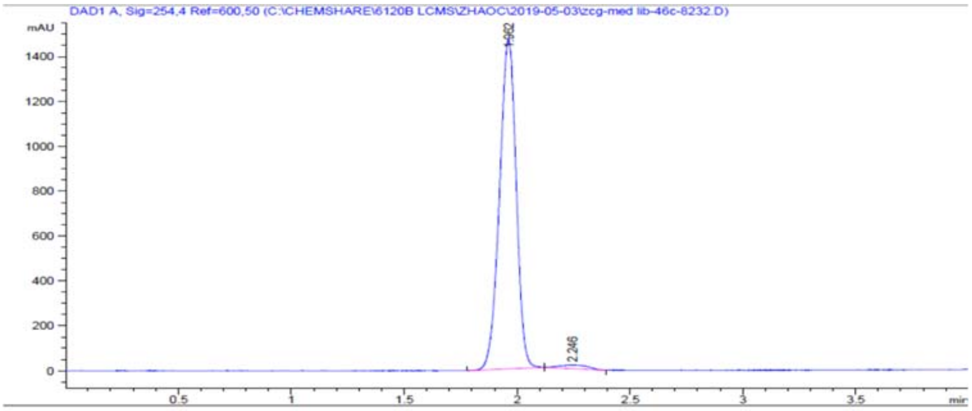

Peak #	RetTime [min]	Type	Width [min]	Area [mAU*s]	Height [mAU]	Area %
1	1.962	BB	0.0792	7535.39160	1461.24268	98.1576
2	2.246	BB	0.1032	141.43507	16.14976	1.8424
Totals :				7676.82668	1477.39243	

Supplementary Figure 275. LC-MS spectra of **46c**, MS: $(M+H)^+$, found: 649.2.

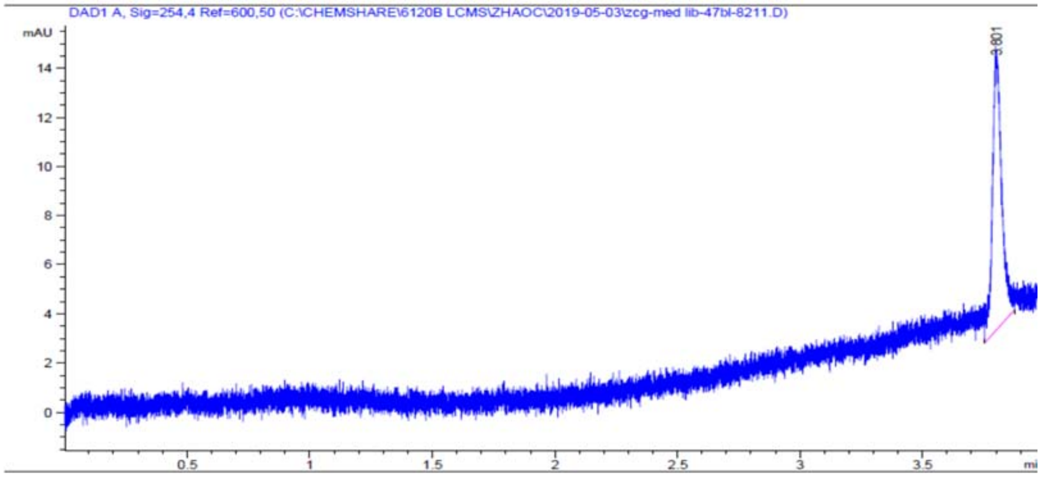

Peak #	RetTime [min]	Type	Width [min]	Area [mAU*s]	Height [mAU]	Area %
1	3.801	BB	0.0370	29.91258	11.23940	100.0000
Totals :				29.91258	11.23940	

Supplementary Figure 276. LC-MS spectra of **47bl**, MS: $(M+H)^+$, found: 604.2.

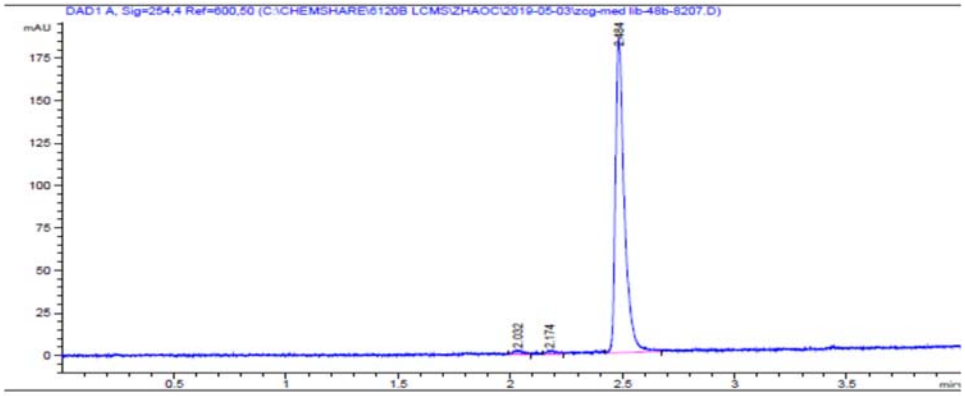

Signal 1: DAD1 A, Sig=254,4 Ref=600,50

Peak #	RetTime [min]	Type	Width [min]	Area [mAU*s]	Height [mAU]	Area %
1	2.032	BB	0.0394	8.10848	2.48651	1.5443
2	2.174	BB	0.0397	6.37945	1.95481	1.2150
3	2.484	BB	0.0409	510.57147	184.58342	97.2407
Totals :				525.05940	189.02474	

Supplementary Figure 277. LC-MS spectra of **48b**, MS: $(M+H)^+$, found: 442.1.

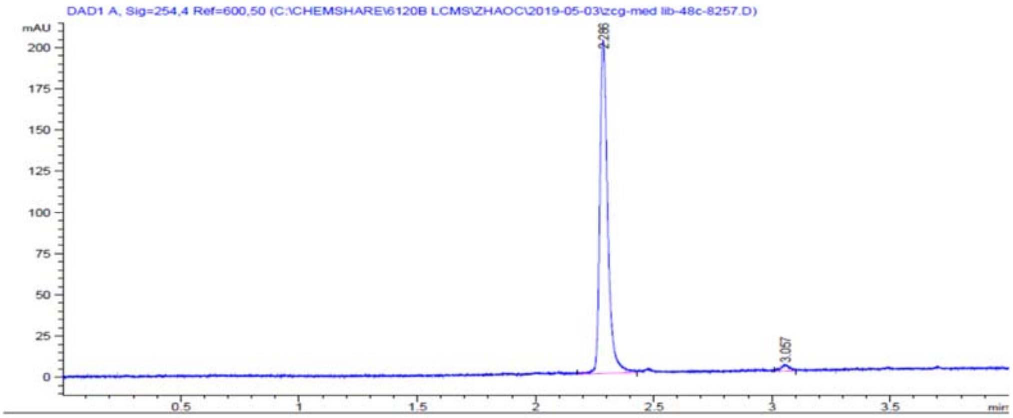

Signal 1: DAD1 A, Sig=254,4 Ref=600,50

Peak #	RetTime [min]	Type	Width [min]	Area [mAU*s]	Height [mAU]	Area %
1	2.286	BB	0.0347	468.67633	202.44060	97.9859
2	3.057	BB	0.0344	9.63371	3.50040	2.0141
Totals :				478.31004	205.94099	

Supplementary Figure 278. LC-MS spectra of **48c**, MS: $(M+H)^+$, found: 524.1.

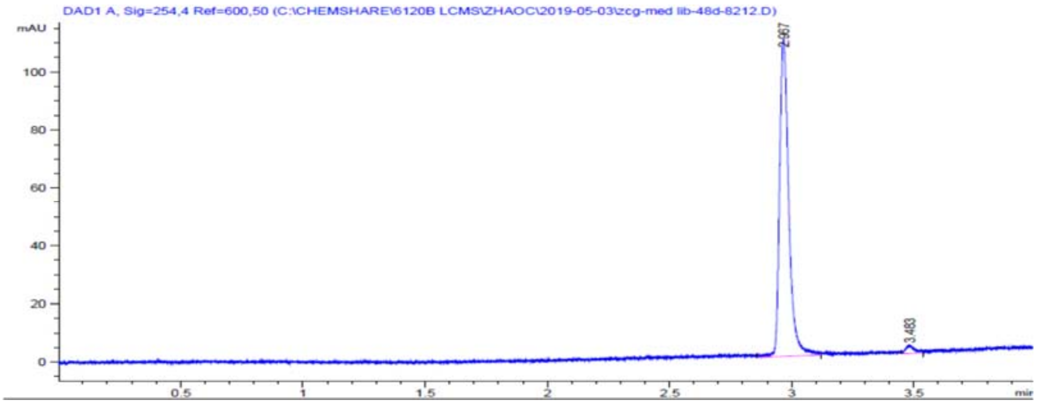

Peak #	RetTime [min]	Type	Width [min]	Area [mAU*s]	Height [mAU]	Area %
1	2.967	BB	0.0411	288.32559	109.45775	97.5198
2	3.483	BB	0.0353	7.33278	2.70836	2.4802
Totals :				295.65837	112.16611	

Supplementary Figure 279. LC-MS spectra of **48d**, MS: $(M+H)^+$, found: 474.1.

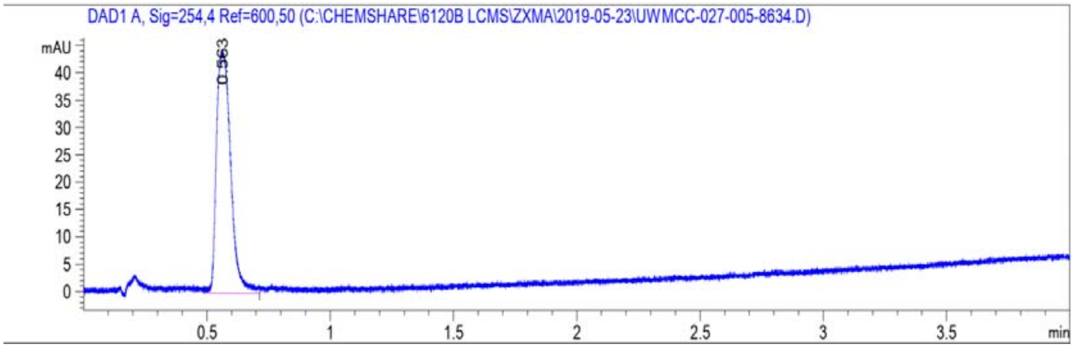

Signal 1: DAD1 A, Sig=254,4 Ref=600,50

Peak #	RetTime [min]	Type	Width [min]	Area [mAU*s]	Height [mAU]	Area %
1	0.563	BB	0.0635	177.16956	43.83995	100.0000
Totals :				177.16956	43.83995	

Supplementary Figure 280. LC-MS spectra of **47aa**, MS: $[M+Na]^+$, found: 464.1.

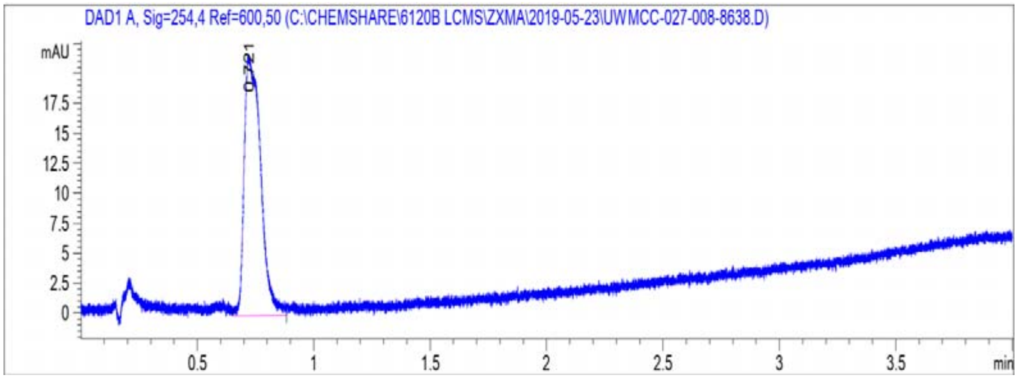

Signal 1: DAD1 A, Sig=254,4 Ref=600,50

Peak #	RetTime [min]	Type	Width [min]	Area [mAU*s]	Height [mAU]	Area %
1	0.721	BB	0.0672	106.88324	21.40781	100.0000
Totals :				106.88324	21.40781	

Supplementary Figure 281. LC-MS spectra of **47ab**, MS: $[M+Na]^+$, found: 478.1.

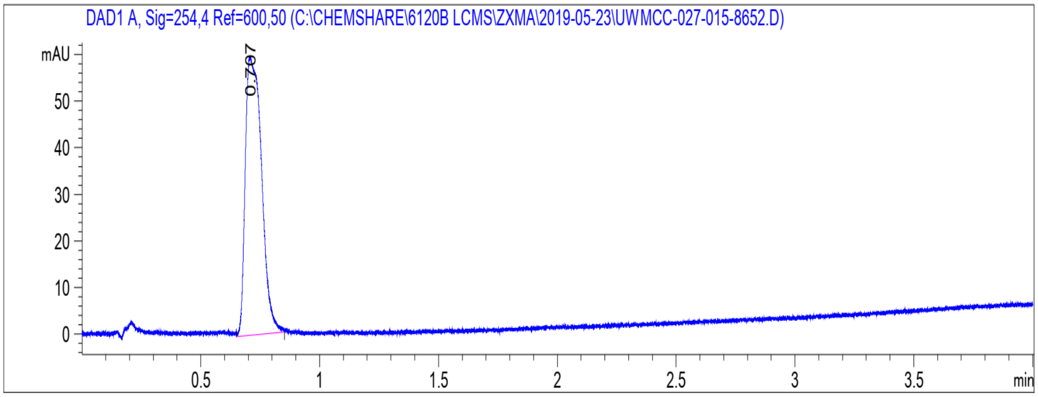

Signal 1: DAD1 A, Sig=254,4 Ref=600,50

Peak #	RetTime [min]	Type	Width [min]	Area [mAU*s]	Height [mAU]	Area %
1	0.707	BB	0.0644	284.24057	59.46888	100.0000
Totals :				284.24057	59.46888	

Supplementary Figure 282. LC-MS spectra of **47ah**, MS: [M+H]$^+$, found: 500.1.

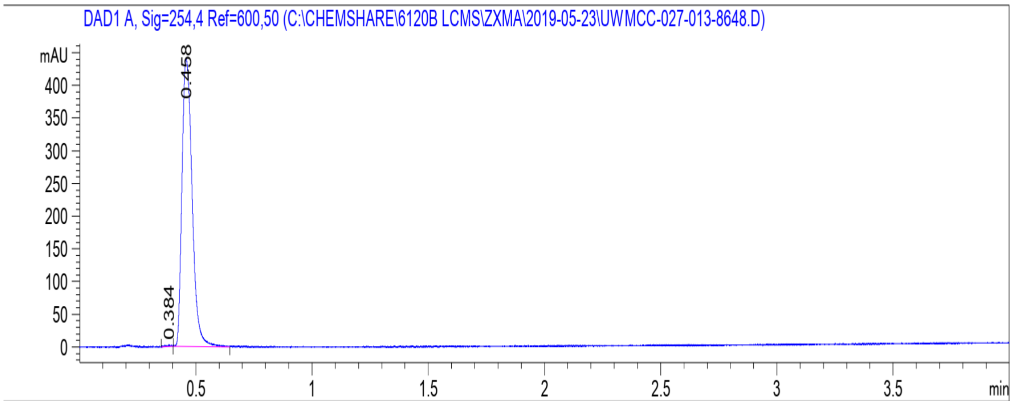

Signal 1: DAD1 A, Sig=254,4 Ref=600,50

Peak #	RetTime [min]	Type	Width [min]	Area [mAU*s]	Height [mAU]	Area %
1	0.384	BB	0.0265	3.53245	1.63467	0.2643
2	0.458	BB	0.0483	1332.84595	440.24777	99.7357
Totals :				1336.37840	441.88244	

Supplementary Figure 283. LC-MS spectra of **47ak**, MS: [M+H]$^+$, found: 533.2.

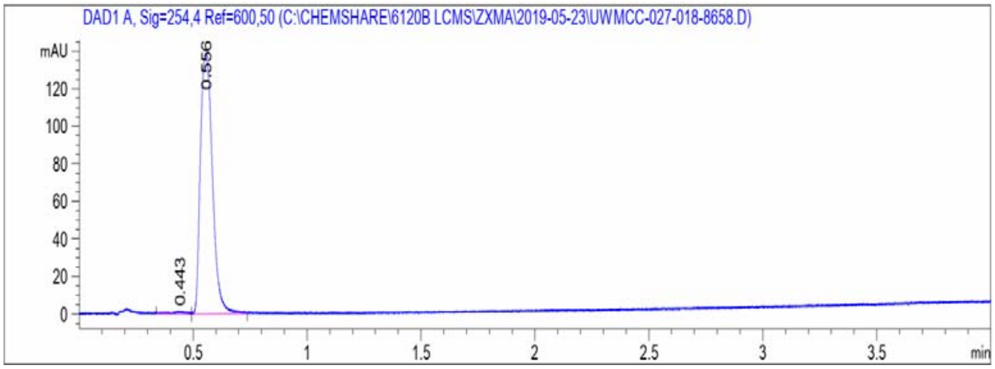

Signal 1: DAD1 A, Sig=254,4 Ref=600,50

Peak #	RetTime [min]	Type	Width [min]	Area [mAU*s]	Height [mAU]	Area %
1	0.443	BB	0.0611	6.85801	1.33867	1.3243
2	0.556	BB	0.0596	510.98947	138.94984	98.6757

Totals : 517.84748 140.28852

Supplementary Figure 284. LC-MS spectra of **47al**, MS: $[M+H]^+$, found: 533.2.

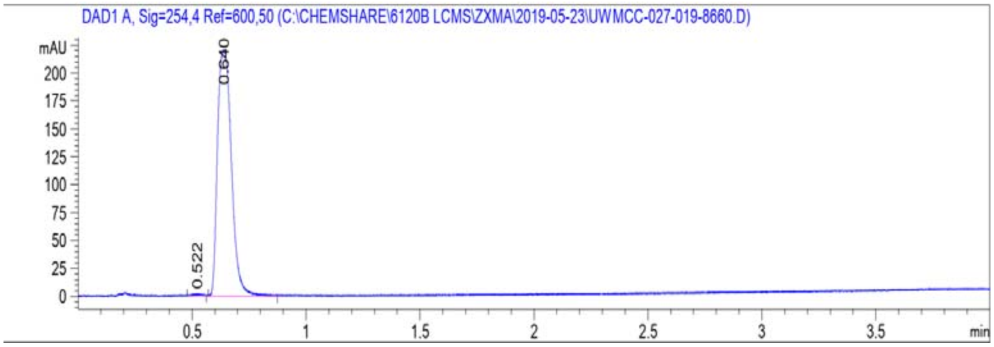

Signal 1: DAD1 A, Sig=254,4 Ref=600,50

Peak #	RetTime [min]	Type	Width [min]	Area [mAU*s]	Height [mAU]	Area %
1	0.522	BB	0.0400	5.08074	1.53367	0.5408
2	0.640	BB	0.0682	934.33435	219.93929	99.4592

Totals : 939.41509 221.47296

Supplementary Figure 285. LC-MS spectra of **47am**, MS: $[M+H]^+$, found: 533.2.

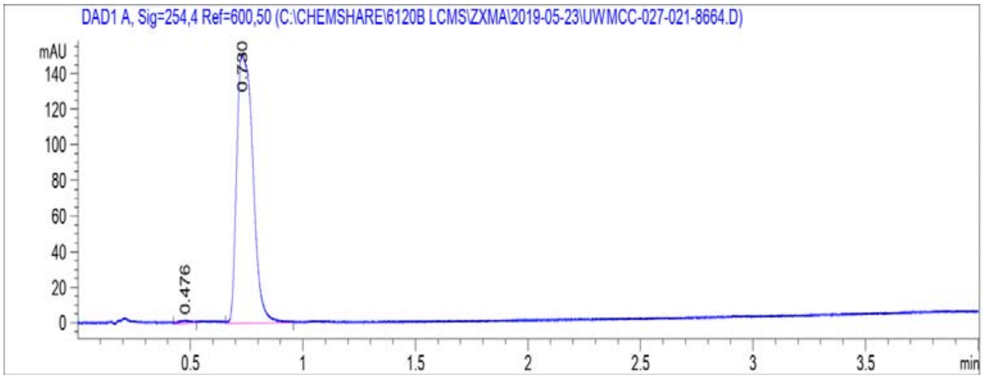

Signal 1: DAD1 A, Sig=254,4 Ref=600,50

Peak #	RetTime [min]	Type	Width [min]	Area [mAU*s]	Height [mAU]	Area %
1	0.476	BB	0.0457	5.45476	1.43561	0.7163
2	0.730	BB	0.0691	756.04346	150.90480	99.2837
Totals :				761.49822	152.34041	

Supplementary Figure 286. LC-MS spectra of **47aq**, MS: $[M+Na]^+$, found: 584.2.

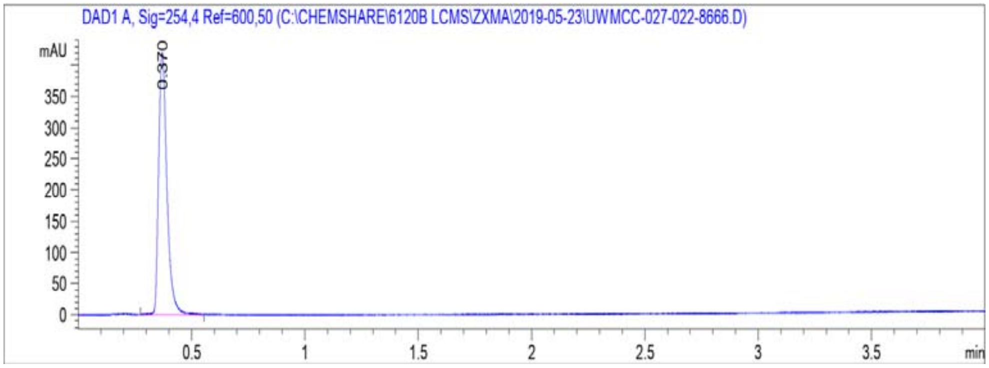

Signal 1: DAD1 A, Sig=254,4 Ref=600,50

Peak #	RetTime [min]	Type	Width [min]	Area [mAU*s]	Height [mAU]	Area %
1	0.370	BB	0.0394	1080.13354	419.19431	100.0000
Totals :				1080.13354	419.19431	

Supplementary Figure 287. LC-MS spectra of **47ar**, MS: $[M+H]^+$, found: 547.2.

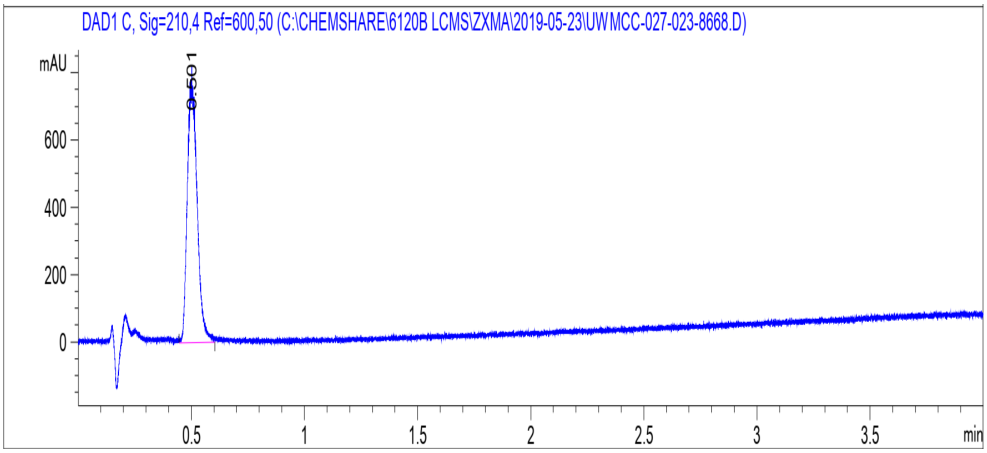

Signal 3: DAD1 C, Sig=210,4 Ref=600,50

Peak #	RetTime [min]	Type	Width [min]	Area [mAU*s]	Height [mAU]	Area %
1	0.501	BB	0.0469	2365.79199	755.49298	100.0000

Totals : 2365.79199 755.49298

Supplementary Figure 288. LC-MS spectra of **47as**, MS: $[M+H]^+$, found: 547.2.

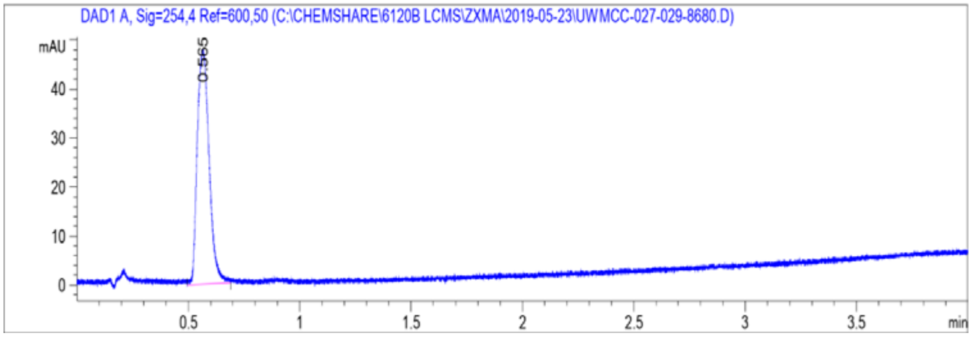

Signal 1: DAD1 A, Sig=254,4 Ref=600,50

Peak #	RetTime [min]	Type	Width [min]	Area [mAU*s]	Height [mAU]	Area %
1	0.565	BB	0.0593	177.92407	47.92444	100.0000

Totals : 177.92407 47.92444

Supplementary Figure 289. LC-MS spectra of **47av**, MS: $[M+Na]^+$, found: 522.2.

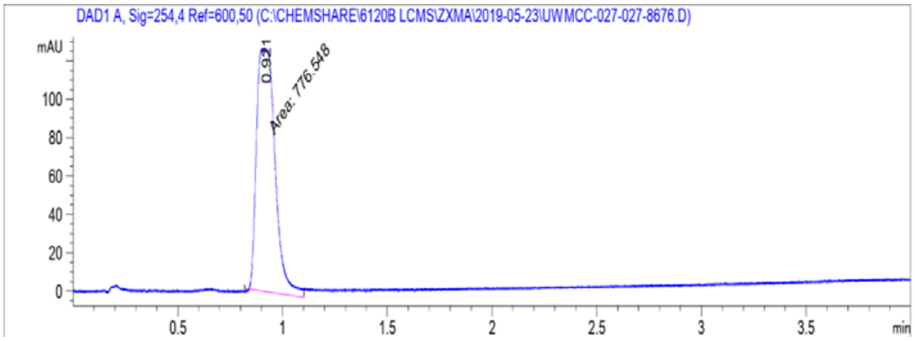

Signal 1: DAD1 A, Sig=254,4 Ref=600,50

Peak #	RetTime [min]	Type	Width [min]	Area [mAU*s]	Height [mAU]	Area %
1	0.921	MM	0.1025	776.54773	126.23119	100.0000
Totals :				776.54773	126.23119	

Supplementary Figure 290. LC-MS spectra of **47aw**, MS: $[M+H]^+$, found: 562.2.

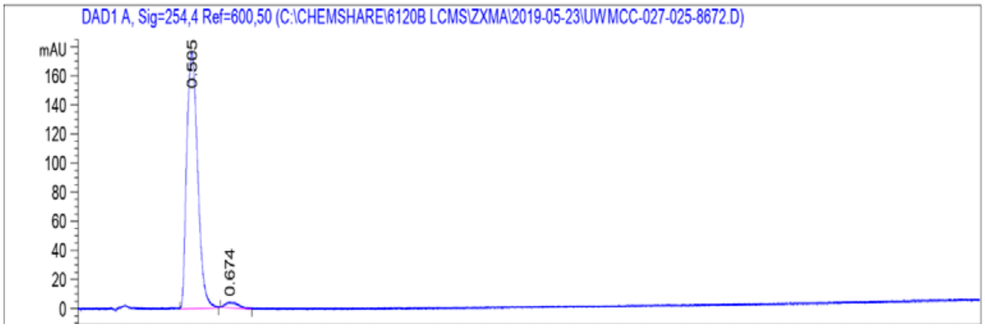

Signal 1: DAD1 A, Sig=254,4 Ref=600,50

Peak #	RetTime [min]	Type	Width [min]	Area [mAU*s]	Height [mAU]	Area %
1	0.505	BB	0.0544	593.88306	175.90561	97.2173
2	0.674	BB	0.0523	16.99913	3.89387	2.7827
Totals :				610.88219	179.79948	

Supplementary Figure 291. LC-MS spectra of **47ax**, MS: $[M+H]^+$, found: 486.1.

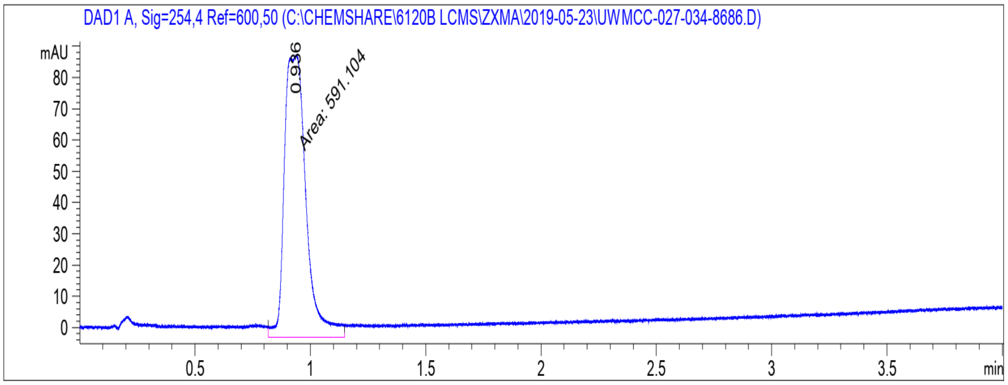

Signal 1: DAD1 A, Sig=254,4 Ref=600,50

Peak #	RetTime [min]	Type	Width [min]	Area [mAU*s]	Height [mAU]	Area %
1	0.936	MM	0.1094	591.10419	90.03129	100.0000
Totals :				591.10419	90.03129	

Supplementary Figure 292. LC-MS spectra of **47ay**, MS: $[M+H]^+$, found: 576.1.

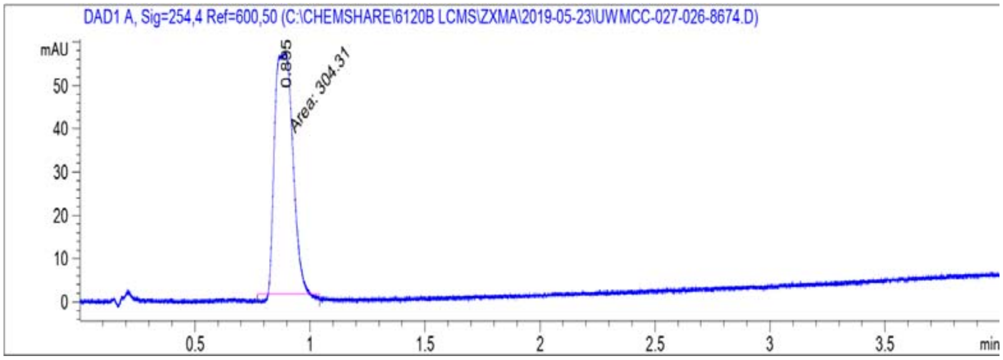

Signal 1: DAD1 A, Sig=254,4 Ref=600,50

Peak #	RetTime [min]	Type	Width [min]	Area [mAU*s]	Height [mAU]	Area %
1	0.895	MM	0.0914	304.30957	55.49830	100.0000
Totals :				304.30957	55.49830	

Supplementary Figure 293. LC-MS spectra of **47az**, MS: $[M+Na]^+$, found: 550.2.

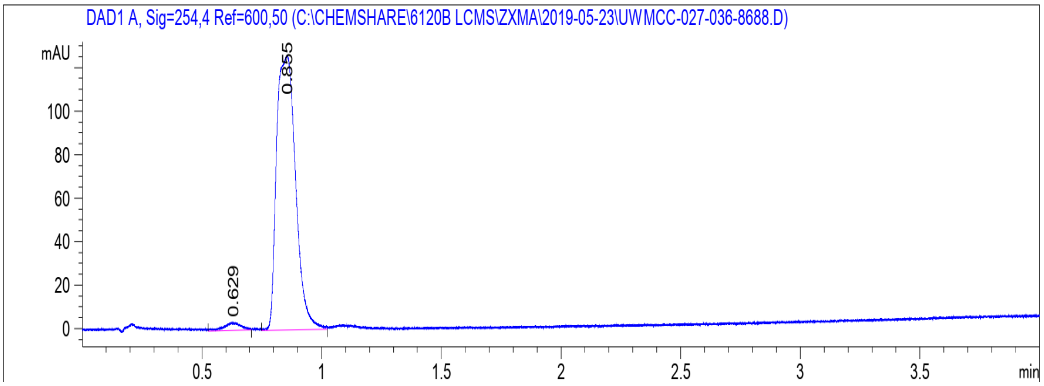

Signal 1: DAD1 A, Sig=254,4 Ref=600,50

Peak #	RetTime [min]	Type	Width [min]	Area [mAU*s]	Height [mAU]	Area %
1	0.629	BB	0.0667	18.19019	3.34867	2.5439
2	0.855	BB	0.0767	696.84882	125.70684	97.4561
Totals :				715.03901	129.05551	

Supplementary Figure 294. LC-MS spectra of **47ba**, MS: [M+H]$^+$, found: 528.2.

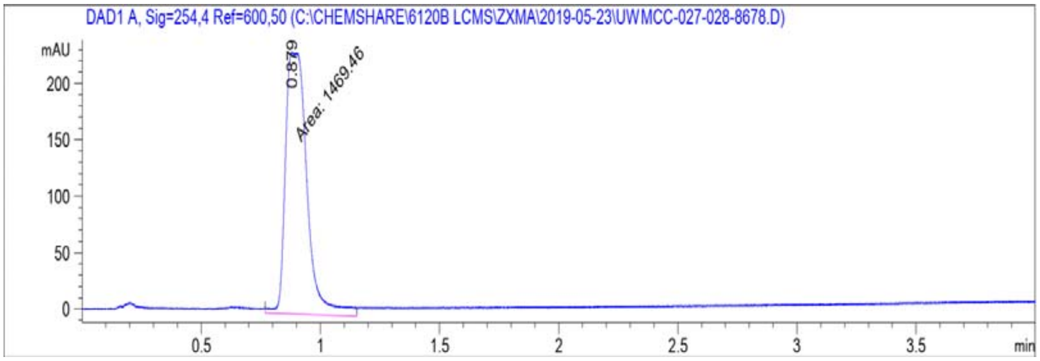

Signal 1: DAD1 A, Sig=254,4 Ref=600,50

Peak #	RetTime [min]	Type	Width [min]	Area [mAU*s]	Height [mAU]	Area %
1	0.879	MM	0.1056	1469.46326	231.90567	100.0000
Totals :				1469.46326	231.90567	

Supplementary Figure 295. LC-MS spectra of **47bb**, MS: [M+H]$^+$, found: 562.2.

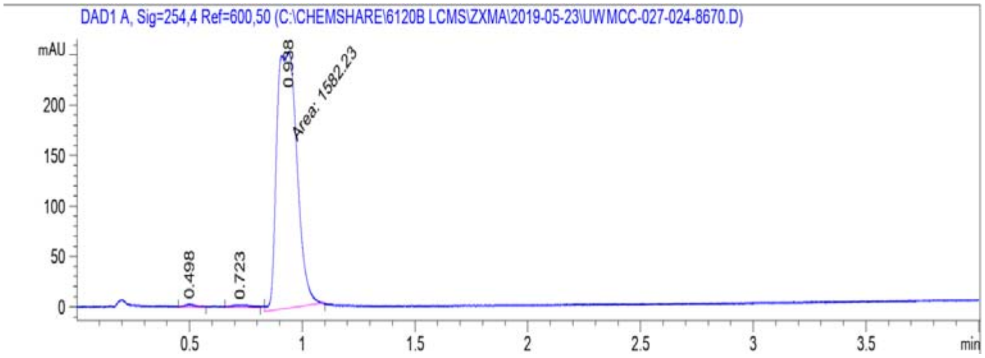

Signal 1: DAD1 A, Sig=254,4 Ref=600,50

Peak #	RetTime [min]	Type	Width [min]	Area [mAU*s]	Height [mAU]	Area %
1	0.498	BB	0.0471	8.58646	2.23702	0.5360
2	0.723	BB	0.0683	11.20103	1.94232	0.6992
3	0.938	MM	0.1037	1582.23193	254.23346	98.7648

Totals : 1602.01943 258.41279

Supplementary Figure 296. LC-MS spectra of **47bc**, MS: $[M+H]^+$, found: 576.2.

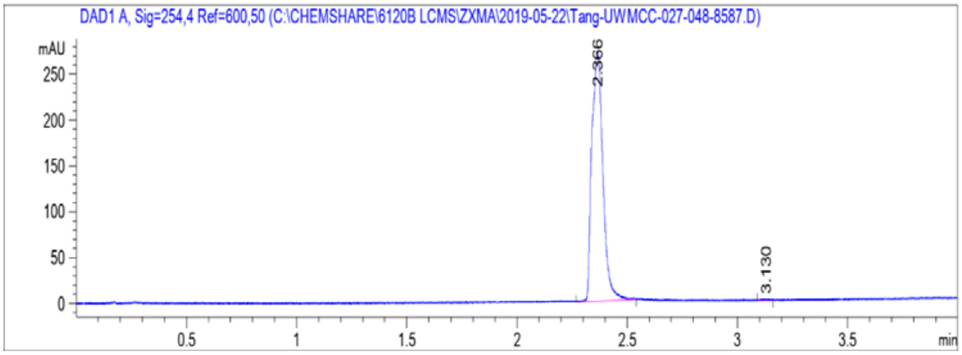

Signal 1: DAD1 A, Sig=254,4 Ref=600,50

Peak #	RetTime [min]	Type	Width [min]	Area [mAU*s]	Height [mAU]	Area %
1	2.366	BB	0.0492	954.99274	272.83850	99.5868
2	3.130	BB	0.0377	3.96280	1.28371	0.4132

Totals : 958.95553 274.12221

Supplementary Figure 297. LC-MS spectra of **47be**, MS: $[M+H]^+$, found: 530.1.

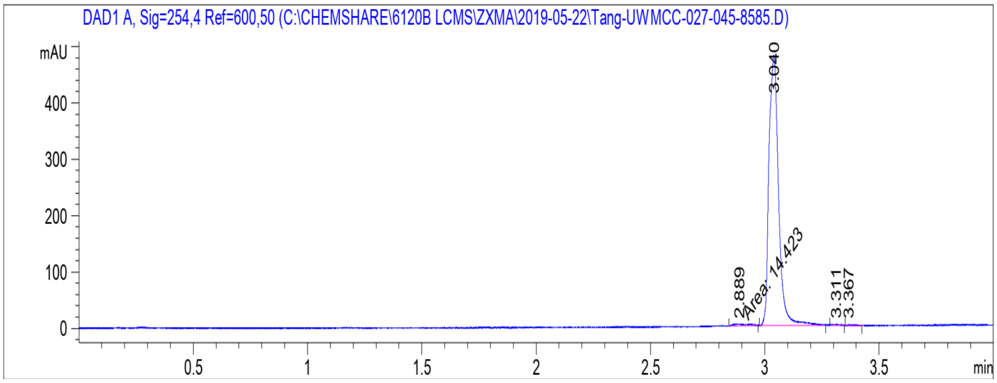

Signal 1: DAD1 A, Sig=254,4 Ref=600,50

Peak #	RetTime [min]	Type	Width [min]	Area [mAU*s]	Height [mAU]	Area %
1	2.889	MM	0.0720	14.42300	3.33998	1.0143
2	3.040	BB	0.0410	1399.37488	479.99518	98.4145
3	3.311	BB	0.0308	4.36962	1.69264	0.3073
4	3.367	BB	0.0393	3.75224	1.15378	0.2639
Totals :				1421.91974	486.18158	

Supplementary Figure 298. LC-MS spectra of **47bf**, MS: [M+H]$^+$, found: 590.2.

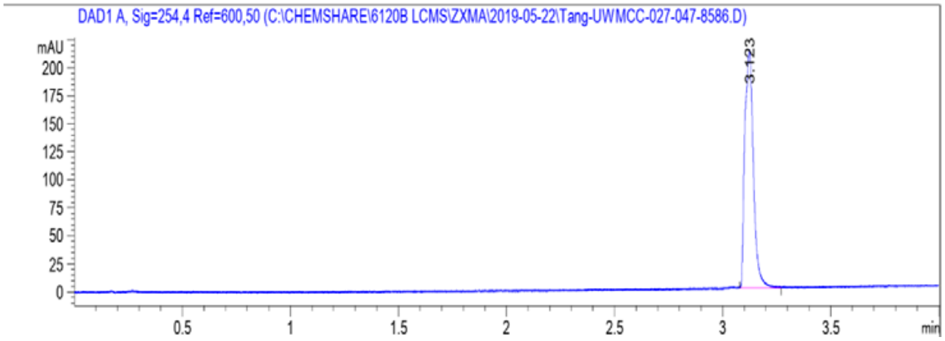

Signal 1: DAD1 A, Sig=254,4 Ref=600,50

Peak #	RetTime [min]	Type	Width [min]	Area [mAU*s]	Height [mAU]	Area %
1	3.123	BB	0.0390	588.54791	212.54967	100.0000
Totals :				588.54791	212.54967	

Supplementary Figure 299. LC-MS spectra of **47bg**, MS: [M+H]$^+$, found: 556.2.

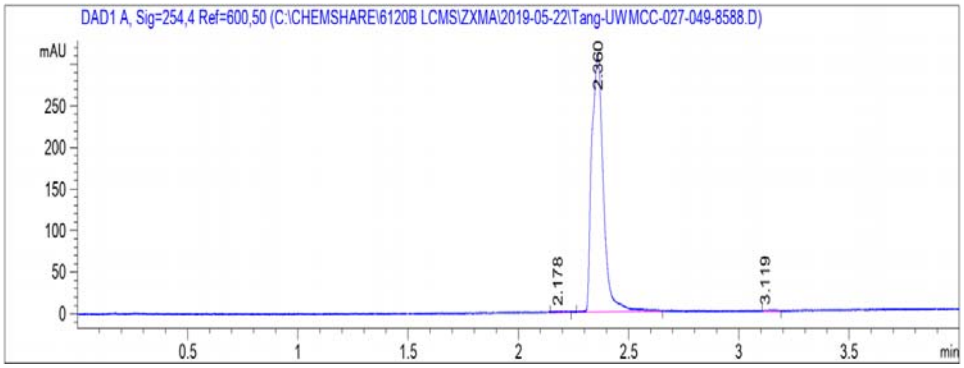

Signal 1: DAD1 A, Sig=254,4 Ref=600,50

Peak #	RetTime [min]	Type	Width [min]	Area [mAU*s]	Height [mAU]	Area %
1	2.178	BB	0.0490	4.91972	1.21348	0.4273
2	2.360	BB	0.0518	1141.61902	311.04358	99.1498
3	3.119	BB	0.0447	4.86955	1.32989	0.4229
Totals :				1151.40829	313.58694	

Supplementary Figure 300. LC-MS spectra of **47bh**, MS: $[M+H]^+$, found: 530.1.

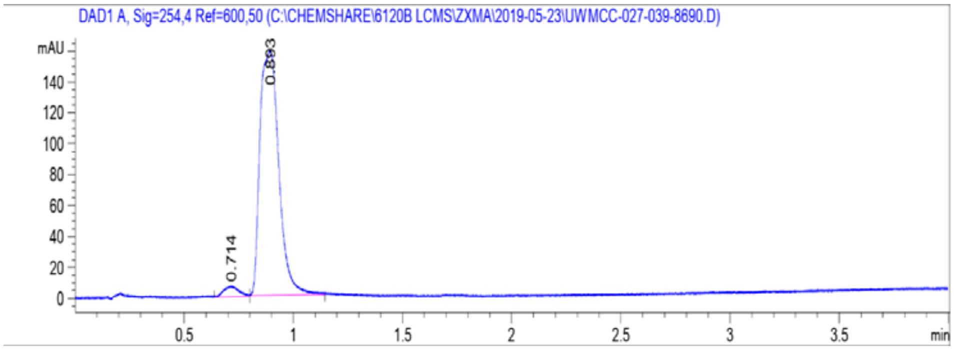

Signal 1: DAD1 A, Sig=254,4 Ref=600,50

Peak #	RetTime [min]	Type	Width [min]	Area [mAU*s]	Height [mAU]	Area %
1	0.714	BB	0.0608	32.51508	6.52767	3.3045
2	0.893	BB	0.0824	951.43506	158.31516	96.6955
Totals :				983.95014	164.84282	

Supplementary Figure 301. LC-MS spectra of **47bi**, MS: $[M+H]^+$, found: 590.2.

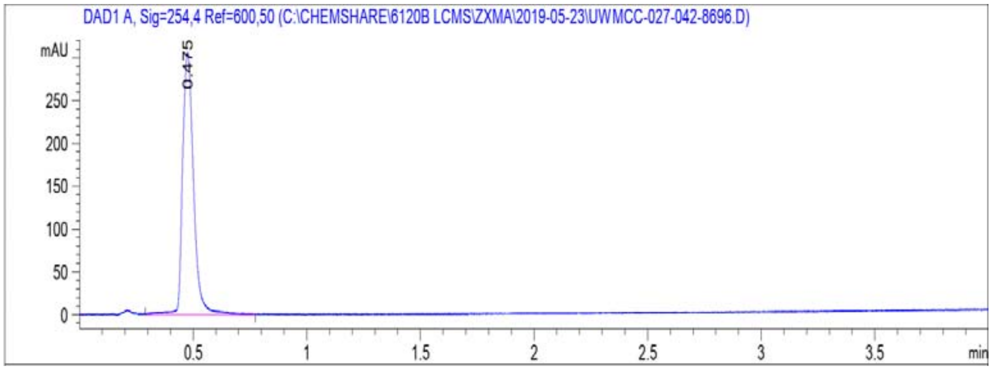

Signal 1: DAD1 A, Sig=254,4 Ref=600,50

Peak #	RetTime [min]	Type	Width [min]	Area [mAU*s]	Height [mAU]	Area %
1	0.475	BB	0.0537	1040.33667	305.77939	100.0000
Totals :				1040.33667	305.77939	

Supplementary Figure 302. LC-MS spectra of **47bj**, MS: $[M+Na]^+$, found: 522.1.

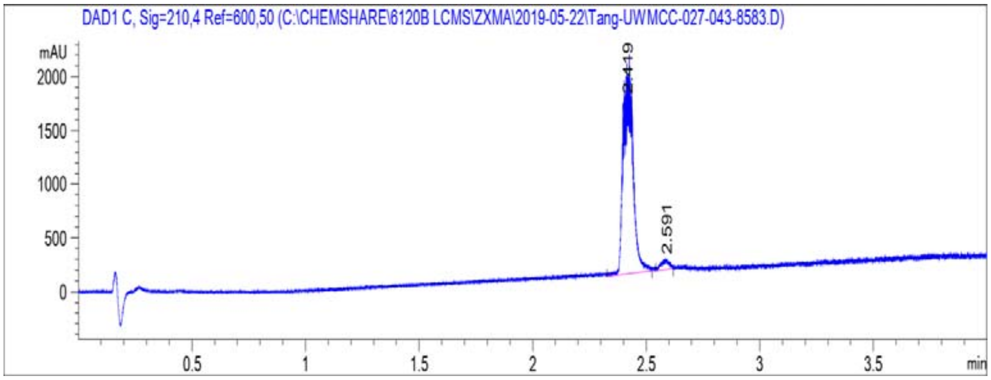

Signal 3: DAD1 C, Sig=210,4 Ref=600,50

Peak #	RetTime [min]	Type	Width [min]	Area [mAU*s]	Height [mAU]	Area %
1	2.419	BB	0.0413	5472.94043	1624.40723	95.7735
2	2.591	BB	0.0326	241.51938	92.14283	4.2265
Totals :				5714.45981	1716.55006	

Supplementary Figure 303. LC-MS spectra of **47bk**, MS: $[M+H]^+$, found: 606.2.

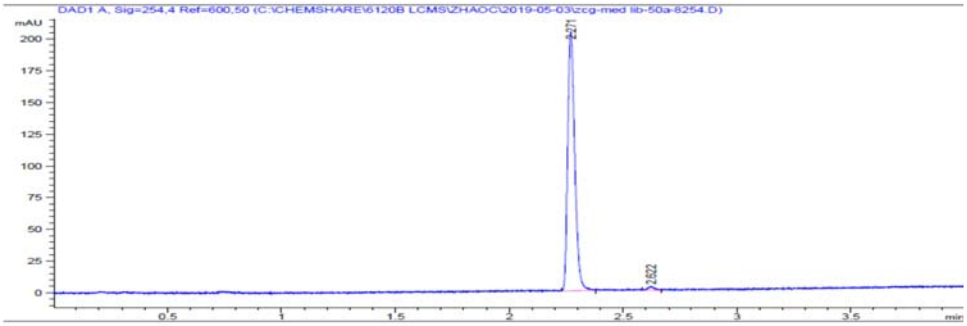

Signal 1: DAD1 A, Sig=254,4 Ref=600,50

Peak #	RetTime [min]	Type	Width [min]	Area [mAU*s]	Height [mAU]	Area %
1	2.271	BB	0.0336	441.59647	203.92654	98.5705
2	2.622	BB	0.0290	6.40434	2.63952	1.4295
Totals :				448.00081	206.56606	

Supplementary Figure 304. LC-MS spectra of **50a**, MS: $(M+H)^+$, found: 460.1.

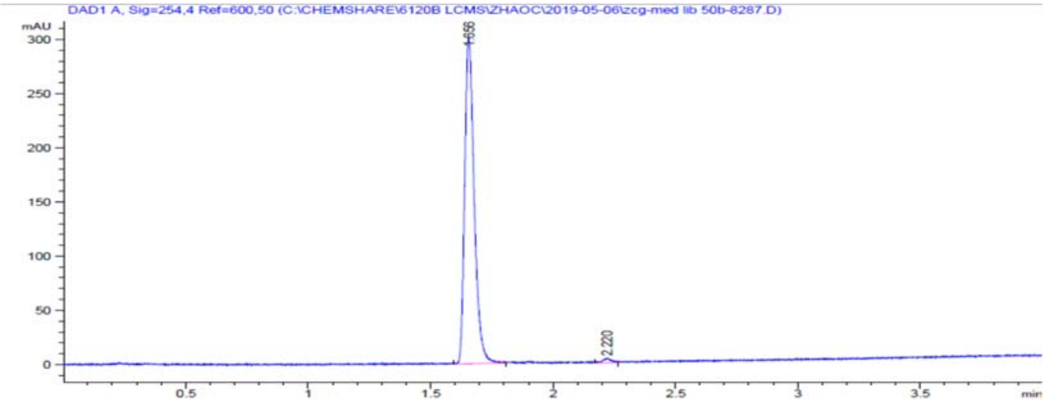

Signal 1: DAD1 A, Sig=254,4 Ref=600,50

Peak #	RetTime [min]	Type	Width [min]	Area [mAU*s]	Height [mAU]	Area %
1	1.656	BB	0.0417	815.90704	300.61743	98.7904
2	2.220	BB	0.0355	9.99033	3.66457	1.2096
Totals :				825.89738	304.28200	

Supplementary Figure 305. LC-MS spectra of **50b**, MS: $(M+H)^+$, found: 462.1.

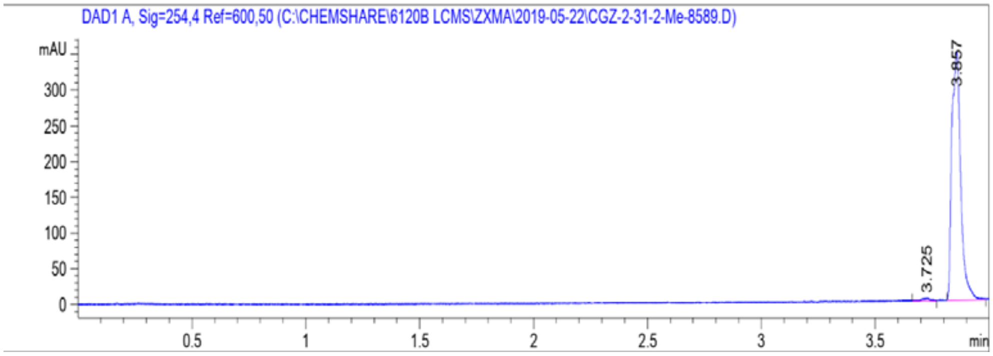

Signal 1: DAD1 A, Sig=254,4 Ref=600,50

Peak #	RetTime [min]	Type	Width [min]	Area [mAU*s]	Height [mAU]	Area %
1	3.725	BB	0.0375	9.93899	3.26708	1.0507
2	3.857	BB	0.0370	936.03607	348.18356	98.9493
Totals :				945.97506	351.45064	

Supplementary Figure 306. LC-MS spectra of **50c**, MS: $[M+Na]^+$, found: 536.2.

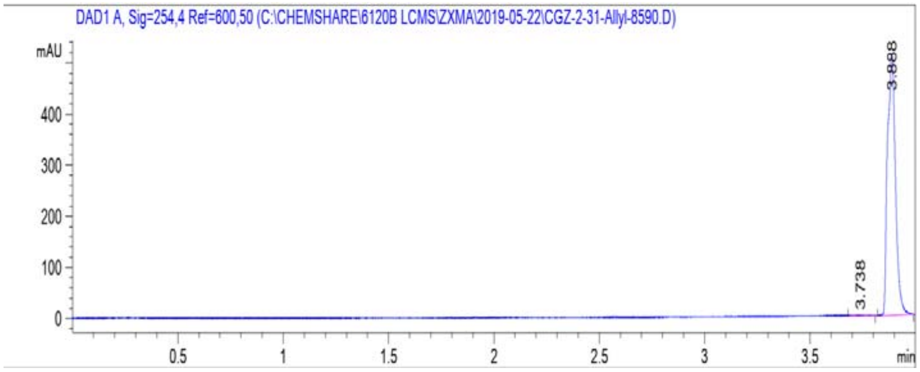

Signal 1: DAD1 A, Sig=254,4 Ref=600,50

Peak #	RetTime [min]	Type	Width [min]	Area [mAU*s]	Height [mAU]	Area %
1	3.738	BB	0.0591	6.15278	1.24347	0.4557
2	3.888	BB	0.0366	1344.00378	512.34650	99.5443
Totals :				1350.15657	513.58996	

Supplementary Figure 307. LC-MS spectra of **50d**, MS: $[M+Na]^+$, found: 562.2.

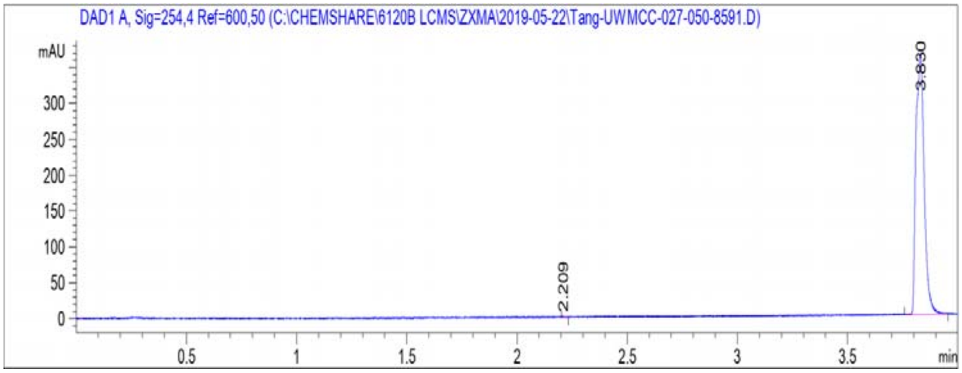

Signal 1: DAD1 A, Sig=254,4 Ref=600,50

Peak #	RetTime [min]	Type	Width [min]	Area [mAU*s]	Height [mAU]	Area %
1	2.209	BB	0.0155	1.48769	1.22734	0.1568
2	3.830	BB	0.0361	947.54669	363.68616	99.8432
Totals :				949.03438	364.91350	

Supplementary Figure 308. LC-MS spectra of **50f**, MS: $[M+Na]^+$, found: 580.2.

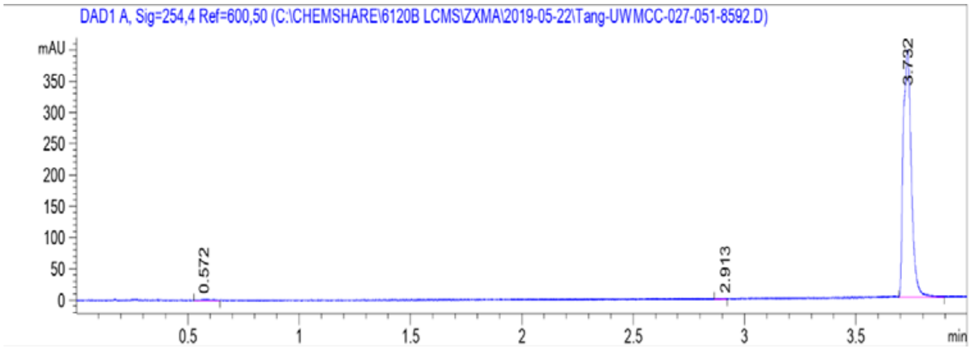

Signal 1: DAD1 A, Sig=254,4 Ref=600,50

Peak #	RetTime [min]	Type	Width [min]	Area [mAU*s]	Height [mAU]	Area %
1	0.572	BB	0.0565	6.65231	1.41561	0.6645
2	2.913	BB	0.0311	3.32227	1.30031	0.3319
3	3.732	BB	0.0350	991.12994	394.71170	99.0036
Totals :				1001.10452	397.42762	

Supplementary Figure 309. LC-MS spectra of **50g**, MS: $[M+Na]^+$, found: 566.2.

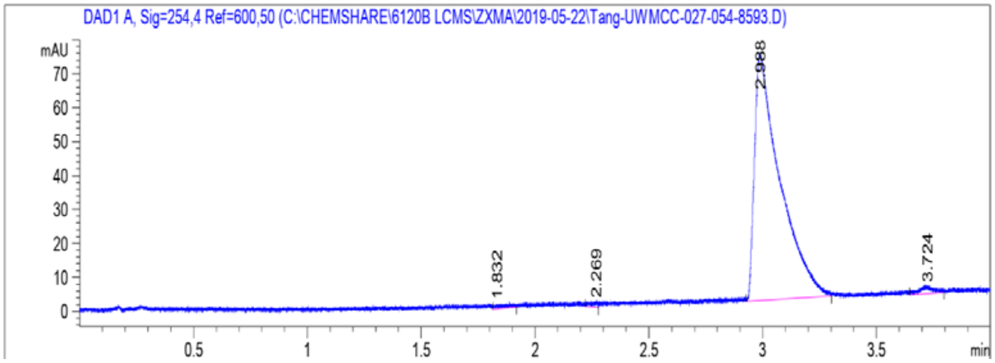

Signal 1: DAD1 A, Sig=254,4 Ref=600,50

Peak #	RetTime [min]	Type	Width [min]	Area [mAU*s]	Height [mAU]	Area %
1	1.832	BB	0.0451	4.38731	1.17824	0.7550
2	2.269	BB	0.0282	2.87784	1.21984	0.4952
3	2.988	BB	0.1000	563.86255	72.57236	97.0273
4	3.724	BB	0.0635	10.01015	1.92013	1.7225
Totals :				581.13785	76.89056	

Supplementary Figure 310. LC-MS spectra of **50h**, MS: $[M+H]^+$, found: 571.3.

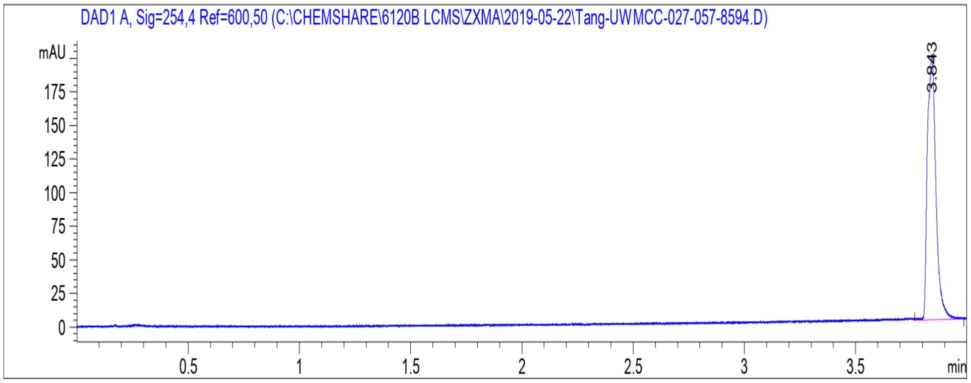

Signal 1: DAD1 A, Sig=254,4 Ref=600,50

Peak #	RetTime [min]	Type	Width [min]	Area [mAU*s]	Height [mAU]	Area %
1	3.843	BB	0.0367	524.32275	197.11107	100.0000
Totals :				524.32275	197.11107	

Supplementary Figure 311. LC-MS spectra of **50i**, MS: $[M+Na]^+$, found: 656.2.

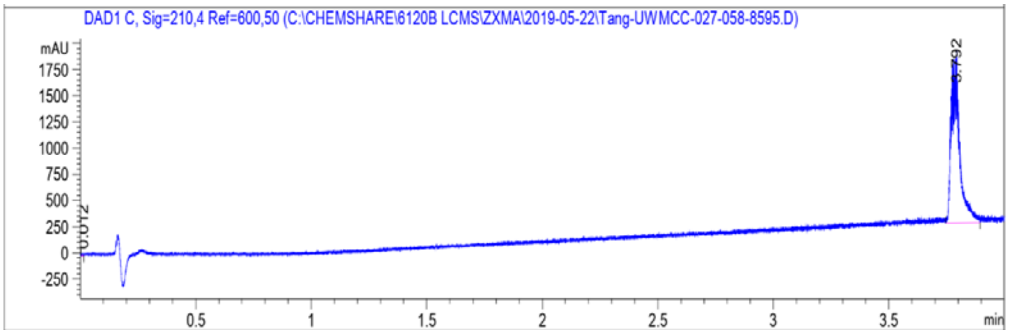

Signal 3: DAD1 C, Sig=210,4 Ref=600,50

Peak #	RetTime [min]	Type	Width [min]	Area [mAU*s]	Height [mAU]	Area %
1	0.012	BB	6.30e-3	6.68205	15.68771	0.1767
2	3.792	BB	0.0334	3774.82446	1371.34241	99.8233

Totals : 3781.50652 1387.03012

Supplementary Figure 312. LC-MS spectra of **50j**, MS: $[M+Na]^+$, found: 656.3.

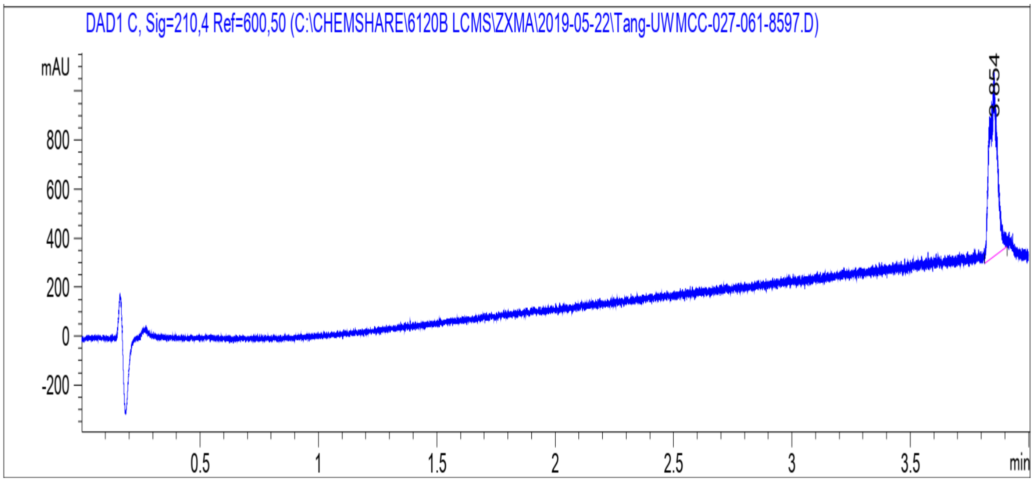

Signal 3: DAD1 C, Sig=210,4 Ref=600,50

Peak #	RetTime [min]	Type	Width [min]	Area [mAU*s]	Height [mAU]	Area %
1	3.854	BB	0.0334	1682.32092	646.49286	100.0000

Totals : 1682.32092 646.49286

Supplementary Figure 313. LC-MS spectra of **51c**, MS: $[M+H]^+$, found: 599.3.

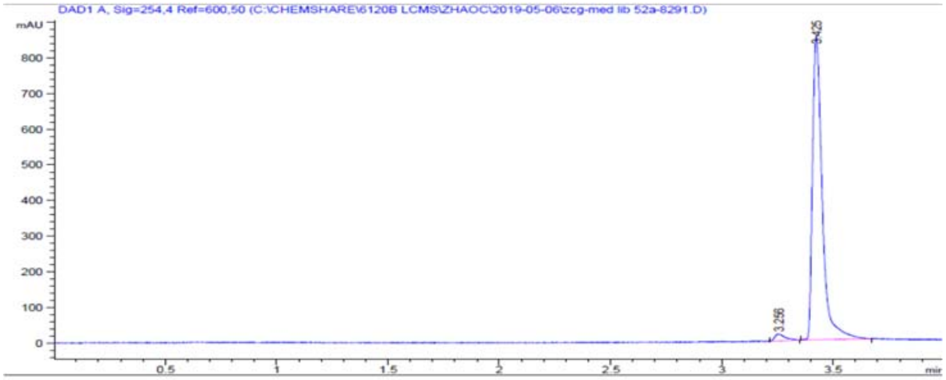

Signal 1: DAD1 A, Sig=254,4 Ref=600,50

Peak #	RetTime [min]	Type	Width [min]	Area [mAU*s]	Height [mAU]	Area %
1	3.256	BB	0.0481	65.47691	19.22167	2.4661
2	3.425	BB	0.0469	2589.55884	850.38123	97.5339
Totals :				2655.03575	869.60290	

Supplementary Figure 314. LC-MS spectra of **52a**, MS: $(M+H)^+$, found: 499.2.

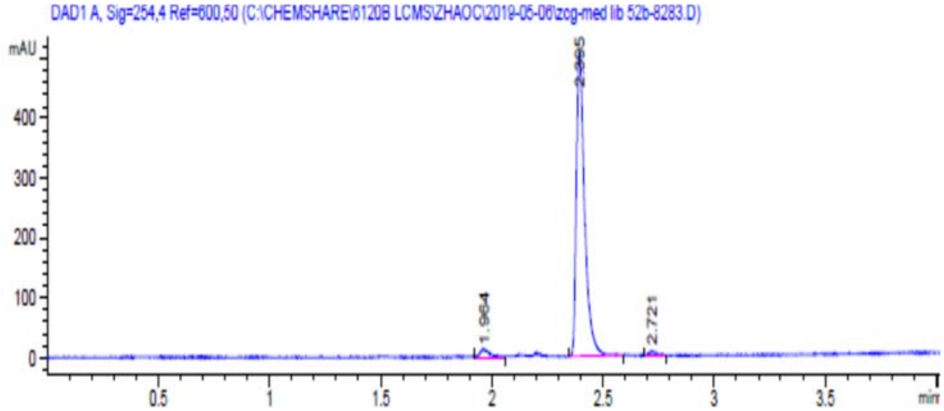

Peak #	RetTime [min]	Type	Width [min]	Area [mAU*s]	Height [mAU]	Area %
1	1.964	BB	0.0444	37.45743	12.45463	2.7150
2	2.395	BB	0.0390	1325.41602	509.18027	96.0676
3	2.721	BB	0.0392	16.79672	5.95884	1.2174
Totals :				1379.67017	527.59374	

Supplementary Figure 315. LC-MS spectra of **52b**, MS: $(M+H)^+$, found: 501.2.

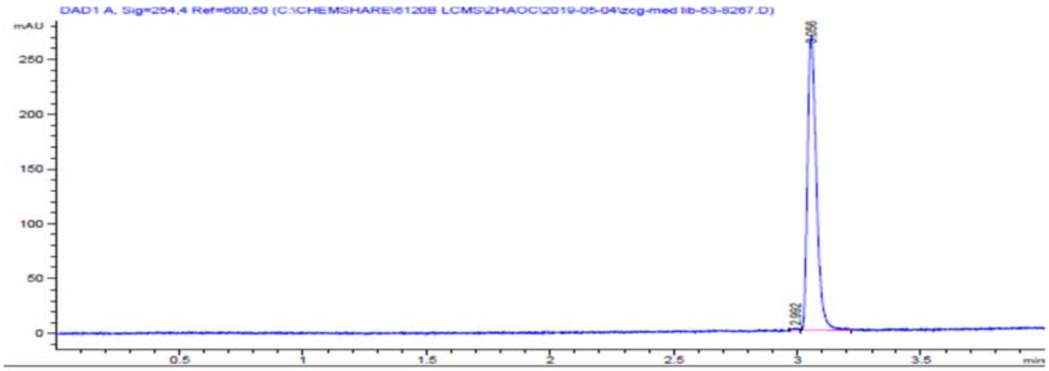

Signal 1: DAD1 A, Sig=254,4 Ref=600,50

Peak #	RetTime [min]	Type	Width [min]	Area [mAU*s]	Height [mAU]	Area %
1	2.992	BB	0.0240	3.47980	1.84466	0.5159
2	3.056	BB	0.0392	670.96979	267.96042	99.4841
Totals :				674.44959	269.80508	

Supplementary Figure 316. LC-MS spectra of **53**, MS: $(M+H)^+$, found: 626.2.

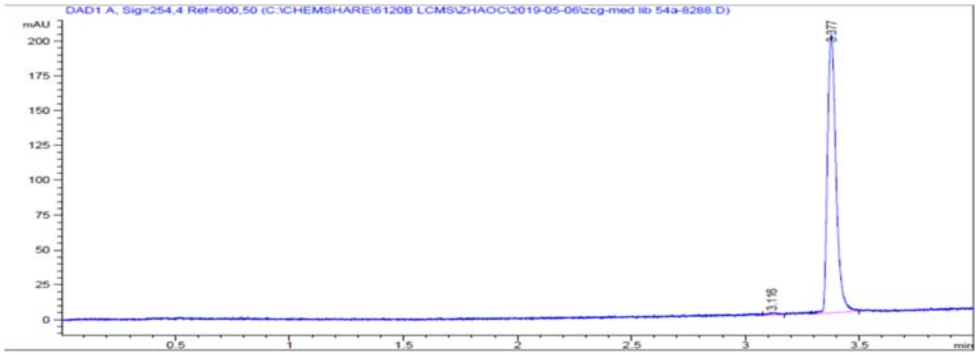

Signal 1: DAD1 A, Sig=254,4 Ref=600,50

Peak #	RetTime [min]	Type	Width [min]	Area [mAU*s]	Height [mAU]	Area %
1	3.116	BB	0.0384	5.09895	1.60649	0.9693
2	3.377	BB	0.0407	520.94727	199.82138	99.0307
Totals :				526.04622	201.42787	

Supplementary Figure 317. LC-MS spectra of **54a**, MS: $(M+H)^+$, found: 612.3.

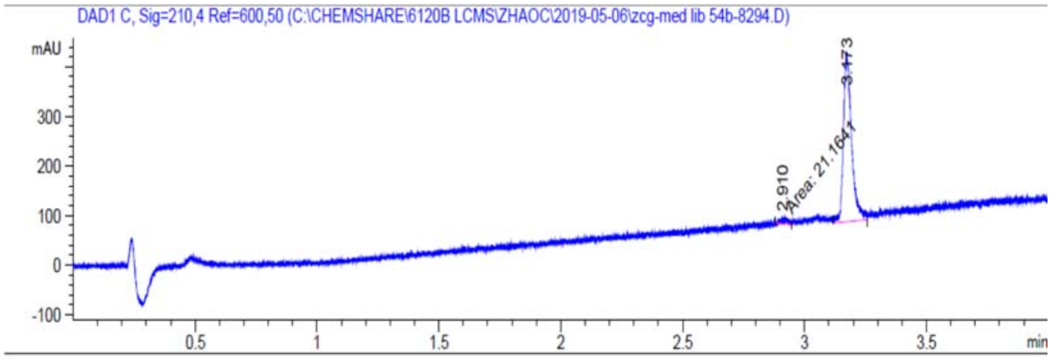

Signal 3: DAD1 C, Sig=210,4 Ref=600,50

Peak #	RetTime [min]	Type	Width [min]	Area [mAU*s]	Height [mAU]	Area %
1	2.910	MM	0.0264	21.16409	13.37608	2.6102
2	3.173	BB	0.0338	789.66327	335.02640	97.3898
Totals :				810.82736	348.40248	

Supplementary Figure 318. LC-MS spectra of **54b**, MS: $(M+H)^+$, found: 614.2.

III. Supplementary References

[1] Purushottamachar, P., Njar, V. C. O. A New Simple and High-Yield Synthesis of 5α-Dihydrotestosterone (DHT), a Potent Androgen Receptor Agonist**.** *Steroids,* **77**, 1530–1534 (2012).

[2] Sun, J., Dong, Y., Cao, L., Wang, X., Wang, S., Hu, Y. Highly Efficient Chemoselective Deprotection of O,O-Acetals and O,O-Ketals Catalyzed by Molecular Iodine in Acetone. *J. Org. Chem.,* **69**, 8932–8934 (2004).

[3] Mo, F., N. Lim, H., Dong, G. Bifunctional Ligand-Assisted Catalytic Ketone α-Alkenylation with Internal Alkynes: Controlled Synthesis of Enones and Mechanistic Studies. *J. Am. Chem. Soc.*, **137**, 15518–15527 (2015).

[4] Pérez-Estrada, S., Rodríguez-Molina, B., Xiao, L., Santillan, R., Jiménez-Osés, G., Houk, K. N. Garcia-Garibay, M. A. Thermodynamic Evaluation of Aromatic CH/π Interactions and Rotational Entropy in a Molecular Rotor. *J. Am. Chem. Soc.*, **137**, 2175–2178 (2015).

[5] Felzmanna, W., Gmeinerb G., Gärtnera, P. First synthesis of a pentadeuterated 3'-hydroxystanozolol--an internal standard in doping analysis. *Steroids* **70**, 103–110 (2005).

[6] Goliaszewski, A. et al. Specific allylic-allylic coupling procedures effected by ligand-induced elimination from di(allylic)palladium species. *Tetrahedron* **41**, 5779–5789 (1985).

[7] Furrow, M. E., Myers, A. G. Practical Procedures for the Preparation of N-tert-Butyldimethylsilylhydrazones and Their Use in Modified Wolff−Kishner Reductions and in the Synthesis of Vinyl Halides and gem-Dihalides, *J. Am. Chem. Soc.*, **126**, 5436–5445 (2004).

[8] Horn, E. J. et al. Scalable and sustainable electrochemical allylic C-H oxidation. *Nature* **533**, 77–81 (2016).

[9] See, Y. Y., Herrmann, A. T., Aihara, Y. Baran, P. S. Scalable C–H Oxidation with Copper: Synthesis of Polyoxypregnanes. *J. Am. Chem. Soc.* **137**, 13776−13779 (2015).

[10] Künzer, H., Thiel, M. A novel route to 3-alkylated estra-1,3,5(10)-trienes. *Tetrahedron Letters*

29*,* 1135−1136 (1988).
[11] Bauer, R. A., Wenderski, T. A. & Tan D. S. Biomimetic diversity-oriented synthesis of benzannulated medium rings via ring expansion. *Nat. Chem. Biol.,* **9**, 21–29, (2013).
[12] Bruker-AXS. *APEX3.* Version 2016.5-0. Madison, Wisconsin, USA, (2016).
[13] Krause, L., Herbst-Irmer, R., Sheldrick, G. M. & Stalke, D. *J. Appl. Cryst.* **48**, 3-10 (2015).
[14] Sheldrick, G. M. *XPREP*. Version 2013/1. Georg-August-Universität Göttingen, Göttingen, Germany (2013b).
[15] Sheldrick, G. M. (2013a). The *SHELX* homepage, http://shelx.uni-ac.gwdg.de/SHELX/.
[16] Sheldrick, G. M. *Acta Cryst. A*, **71**, 3-8 (2015a).
[17] Sheldrick, G. M. *Acta Cryst. C*, **71**, 3-8 (2015b).
[18] Dolomanov, O. V., Bourhis, L. J., Gildea, R. J., Howard, J. A. K. & Puschmann, H. *J. Appl. Crystallogr.* **42**, 339-341 (2009).
[19] Guzei, I. A. (2007-2013). Programs *Gn*. University of Wisconsin-Madison, Madison, Wisconsin, USA.
